# Proteomic Analysis of Quercetin-Treated K562 Cells

**DOI:** 10.3390/ijms21010032

**Published:** 2019-12-19

**Authors:** Fabrizia Brisdelli, Laura Di Francesco, Alessandra Giorgi, Anna Rita Lizzi, Carla Luzi, Giuseppina Mignogna, Argante Bozzi, M. Eugenia Schininà

**Affiliations:** 1 Department of Biotechnological and Applied Clinical Sciences, University of L’Aquila, 67100 L’Aquila, Italy; fabrizia.brisdelli@cc.univaq.it (F.B.); annarita.lizzi@cc.univaq.it (A.R.L.); carla.luzi@cc.univaq.it (C.L.); argante.bozzi@cc.univaq.it (A.B.); 2 Department of Biochemical Sciences, Sapienza, University of Rome, 00185 Rome, Italy; laura.difrancesco@uniroma1.it (L.D.F.); alessandra.giorgi@uniroma1.it (A.G.); pina.mignogna@uniroma1.it (G.M.)

**Keywords:** chronic myeloid leukemia, K562, quercetin, flavonoids, apoptosis, SILAC, quantitative proteomics, oxidative stress, lipid metabolism

## Abstract

Among natural products under investigation for their additive potential in cancer prevention and treatment, the flavonoid quercetin has received attention for its effects on the cell cycle arrest and apoptosis. In the past, we addressed this issue in K562 cells, a cellular model of the human chronic myeloid leukemia. Here, we applied stable isotope labeling by amino acids in cell culture (SILAC) proteomics with the aim to increase knowledge on the regulative and metabolic pathways modulated by quercetin in these cells. After 24 h of quercetin treatment, we observed that apoptosis was not completely established, thus we selected this time range to capture quantitative data. As a result, we were able to achieve a robust identification of 1703 proteins, and to measure fold changes between quercetin-treated and untreated cells for 1206 proteins. Through a bioinformatics functional analysis on a subset of 112 proteins, we propose that the apoptotic phenotype of K562 cells entails a significant modulation of the translational machinery, RNA metabolism, antioxidant defense systems, and enzymes involved in lipid metabolism. Finally, we selected eight differentially expressed proteins, validated their modulated expression in quercetin-treated K562 cells, and discussed their possible role in flavonoid cytotoxicity. This quantitative profiling, performed for the first time on this type of tumor cells upon treatment with a flavonoid, will contribute to revealing the molecular basis of the multiplicity of the effects selectively exerted by quercetin on K562 cells.

## 1. Introduction

Flavonoids are plant-derived compounds that are present in fruits, vegetables, legumes, red wine, and green tea. They are known to display antioxidant and anti-inflammatory effects, and to exhibit many antineoplastic properties, including inhibitory effects on cancer cell proliferation, tumor growth, angiogenesis, metastasis, as well as induction of apoptosis [1].

Quercetin (3,3′,4′,5,7-pentahydroxyflavone) is a flavonol widely found in the plant kingdom and is a component of most edible fruits and vegetables, with highest concentrations present in onions, apples, and red wine [2]. Great attention has been given to quercetin as proapoptotic agent with a specific activity on several cancer cell lines, without affecting normal cells [3,4]. Indeed, a significant increase in the survival rate and a marked reduction in tumor volume were observed in tumor-bearing animals treated with quercetin [5].

Quercetin is known to have multiple intracellular targets, including proteins involved in apoptosis and cell cycle [3]. It has been shown that quercetin can modulate signal proteins, like the NF-kappa-B transcription factor (NF-kB), the cytochrome c oxidase subunit 2 (Cox-2), the NAD-dependent protein deacetylase sirtuin-1 (SIRT-1), and the cellular tumor antigen p53. Moreover, downregulation of the antiapoptotic proteins coded by the *Bcl-xL* and *Bcl-2* genes and upregulation of proapoptotic proteins coded by *Bax* genes have also been reported [6,7,8].

It is well known that quercetin is able to mediate both intrinsic as well extrinsic apoptotic cell death in cancer cells [9,10]. In liver carcinoma HepG-2 and in human gastric cancer stem cells, quercetin can trigger apoptosis by inhibition of survival signal of PI-3-kinase/Akt pathway [11,12]. Evidence that quercetin-induced apoptosis is associated with downregulation of heat shock proteins, such as the heat shock proteins HSP-70 and HSP-90 in prostate cancer and in leukemic cells, have been also collected [13,14]. Quercetin exerts its anticancer activity also by modulating several proteins involved in the cell cycle regulation, such as p21, p53, cyclin B1, cyclin D1, and p27. Depending on the cell type, it can inhibit cell cycle progression, blocking cells at G2/M or at G1/S by regulating the expression of cyclin-dependent kinases (CDKs) [15,16,17,18].

The growth-suppressive and apoptotic effect of quercetin has also been described in leukemic cells. In K562 cells, an established model of the human chronic myeloid leukemia (CML), quercetin-induced apoptosis has been associated to a reduction of *HSP70*, *Bcl-xL*, and *FOXM1* transcripts [14]. In acute lymphoid leukemia (ALL) and CML cells, quercetin is able to suppress the activity of telomerase [19], while in T-lymphoblastic leukemia cell line, it causes a decrease of the level and activity of the protein *NOTCH1* gene products [20]. In human myelomonocytic cell line U-937, it has been reported that quercetin decreases the level of Induced myeloid leukemia cell differentiation Mcl-1 protein, a prosurvival member of the Bcl-2 family, inducing apoptosis at high concentration and sensitizing cells to apoptosis triggered by drugs or death receptor inducers at low concentrations [21,22].

In our previous studies we were able to prove that exposure of K562 cells to 25 µM quercetin caused an almost full block of growth, associated with a G2/M phase arrest and with a significant decrease of cell percentage in G1 and S phase of cell cycle. Moreover, a progressive increase of apoptosis from 24 h (10% of apoptotic cells) to 72 h (30–40% of apoptotic cells) was observed [23,24]. Since quercetin is a pleiotropic molecule and may exert its effects on different pathways, in the present work, we have moved to a genome-wide approach for unveiling the molecular landscape induced by this flavonoid on K562 cells. With this aim, we analyzed altered protein expression in K562 cells exposed for 24 h to the same concentration of quercetin as the previous works, but focusing our attention on all the proteomic pattern of treated cells. For a reliable quantitative comparative analysis of quercetin-treated and -untreated K562, we selected the stable isotope metabolic labeling of amino acid residues in cell culture (SILAC) approach [25]. Validation of our proteomic results was addressed on a number of down- or upregulated proteins, selected on the basis of their antioxidant activity, their involvement in cell proliferation and survival, and their role in the altered lipid metabolism in cancer cells. Their possible involvement in the K562 responsiveness to the quercetin dysregulation is discussed here. Moreover, the subset of 112 proteins proposed as putative biomarkers of the quercetin-induced effects on K562 could represent a robust starting point for future investigations on the effects of this flavonoid on human chronic myeloid leukemia cells.

## 2. Results

### 2.1. Effect of Quercetin on K562 Cell Growth and Apoptosis

**Figure 1:**
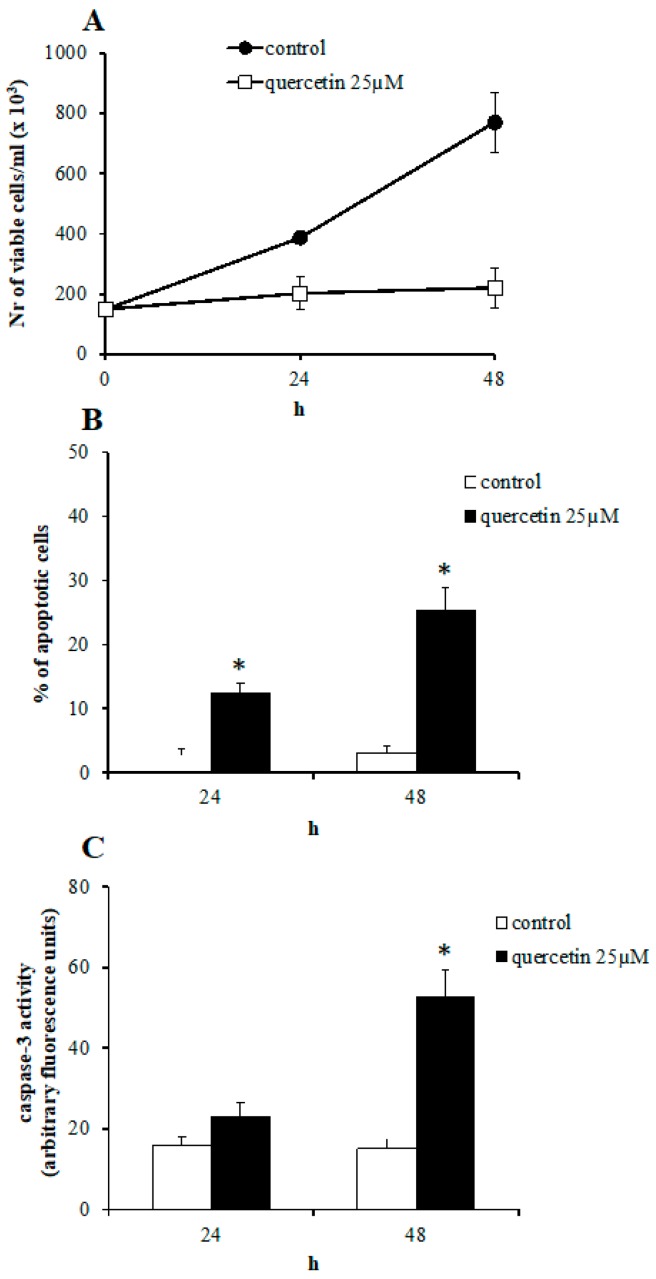
Effect of 25 µM quercetin on K562 cell growth (**A**), apoptosis (**B**), and caspase-3 activity (**C**). (**A**) Cells were counted and the number of trypan blue-negative cells was determined at the indicated times. (**B**) The percentage of condensed and fragmented nuclei was estimated by fluorescence microscope analysis of acridine orange and ethidium bromide double-stained cells at the indicated times. At least 400 cells were counted for each determination. (**C**) The caspase-3 activity was measured spectrofluorimetrically using DEVD-aminomethylcoumarin as substrate. Results represent the mean ± SD of three independent experiments. Statistical evaluation was achieved by Student’s *t*-test. *, data are significantly different from untreated cells (*p* < 0.05).

The effect of 25 µM quercetin treatment on proliferation and viability of K562 cells was evaluated after 24 and 48 h of treatment by trypan blue exclusion test and analysis of apoptotic markers, such as condensation and fragmentation of nuclear chromatin and activation of caspase-3. As previously described [23], quercetin significantly inhibited the K562 growth, with an almost full block of proliferation already after 24 h of treatment, as seen in Figure 1, panel A. The analysis of nuclear morphology showed that the percentage of apoptotic cells reached 25.3% after 48 h of quercetin treatment, as seen in Figure 1, panel B. A 3.5-fold increase of caspase-3 activity after 48 h of treatment, compared to untreated cells, confirmed the induction of apoptosis by quercetin, as seen in Figure 1, panel C.

### 2.2. Proteome Profile of Quercetin-Treated K562 Cells

In order to highlight early molecular events leading to quercetin cytotoxicity in K562 cells, proteome analysis was performed following the 24 h treatment, when cells were already not proliferating, but DNA fragmentation and caspase-3 activation were less marked.

**Figure 2:**
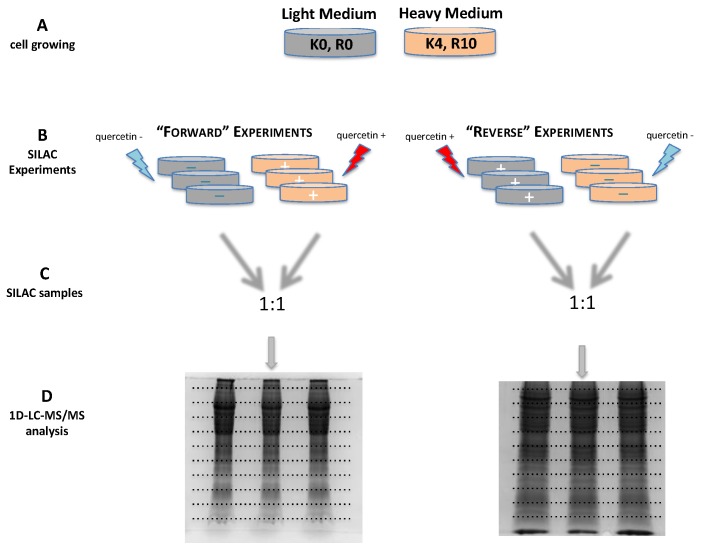
Schematics of the workflow developed in this work. The entire strategy employed in profiling protein fold changes in quercetin-treated K562 cells consists of four main steps (horizontal levels). (**A**) Cell growing. Orange culture dishes codes for K562 cells grown in a “heavy” medium supplemented with L-[4,4,5,5-D4]-lysine and L-[^13^C6^15^N4]-arginine (marked as K4, R10); grey culture dishes codes for K562 cells grown in a “light” medium supplemented with L-lysine and L-arginine (marked as K0, R0). (**B**) Treatment scheme in “forward” and “reverse” SILAC experiments. In “forward” experiments, heavy isotope labelled cells (in orange code) were treated for 24 h with 25 μM quercetin (red lightning bolt), whereas control cells (grey coded) where treated with a vehicle solution (DMSO; cyan lightning bolt). In “reverse” SILAC experiments, media were swapped. The three replicates for each status are also shown. (**C**) Scheme of the pairwise merging into the SILAC samples. For sake of clarity, merging of only a treated and control replicate over three for each SILAC experiment is shown. Arrows with a cyan tail refers to the aliquot from a control cultured cell, whereas the aliquot from a quercetin treated sample is coded by an arrow with a red tail; colors of the arrow heads code for the stable isotope labelling of the cell culture, as described in line A. (**D**) Summary of the proteomics strategy. Figures of the SDS-PAGE of the three replicates for each “forward” and “reverse” SILAC experiments are shown. Dashed lines refer to slice excision selection. At this final step, two further replicates (“technical”) were obtained by splitting each slice, and each submitted to LC-MSMS analysis.

In these conditions, small fold changes between the quercetin-treated and -untreated K562 proteomes were expected. As a quantitative approach reliable for a confident measurement of differential expression following a 24 h quercetin treatment, we selected the SILAC methodology, using stable isotope labeled lysine and arginine [25]. In order to achieve a robust measurement of the protein relative abundances, we introduced a label-swapping replication [26], three biological replicates, and two technical replicates in the experimental design. The entire proteomic experiment is schematically shown in Figure 2.

**Figure 3:**
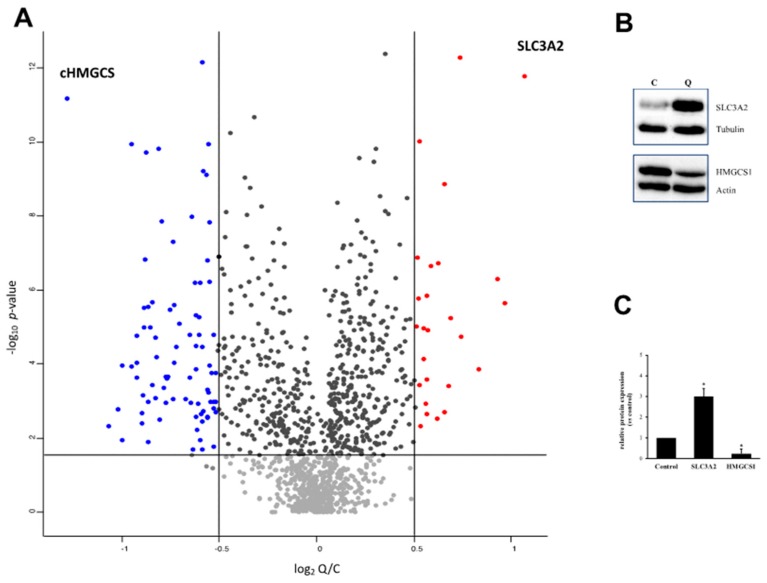
Summary of SILAC data. (**A**) Volcano plot of quantitative data. One sample *t*-test analysis was performed on the protein relative abundances measured among data from 12 replicates between quercetin treated and control cells (Q/C ratios; columns headed as “H/L normalized ratio” in Table S2, according to the original MaxQuant output). Log_2_ Q/C values were plotted against the −log_10_ of the FDR adjusted *p*-values (according to the Benjamini–Hochberg method; y axis, –log_10_ *p*-value). Vertical and horizontal lines mark Q/C and *p*-values used as arbitrary thresholds in DEPs selection. Blue dots show proteins upregulated in quercetin-treated cells, red dots those downregulated. (**B**) Western blotting for SLC3A2 and HMGCS1 proteins, employed in validating SILAC data. Immunoblots are representative of three independent experiments with similar results. C: Control, untreated cells; Q: Quercetin-treated cells. (**C**) Quantification of relative abundance. In the densitometric analysis, after normalization with tubulin or actin protein levels, values have been obtained by the ratio between the intensities of SLC3A2 and HMGCS1 bands in quercetin-treated and -untreated cells, assigning the value 1 to the control. Results represent the mean ± SD of three independent experiments. Statistical evaluation was achieved by Student’s *t*-test. *, data significantly different from untreated cells (*p* < 0.05).

Through this SILAC approach, we were able to determine a K562 proteome profile encompassing a total of 1703 protein groups, as seen in Table S1. Upon a stringent analysis of the quantitative mass spectrometric data, reliable fold changes between quercetin-treated and -untreated proteomes were calculated for 1206 identification hits, as seen in Table S2 and Figure 3, panel A.

Although quercetin-induced K562 proteomes were not found to be largely altered, we were able to measure, with statistical significance, abundance differences higher than 40–50% (log_2_ fold change > 0.5) for 112 proteins. Then, we arbitrarily considered them as differentially expressed proteins (DEPs). Among these, 27 were upmodulated in quercetin-treated cell cultures, as listed in Table 1, whereas 85 were downmodulated in the same cell status, as listed in Table 2.

As validation of our data, we submitted to Western blotting the two proteins for which highest fold changes were determined, and in a consistent manner among all replicates, as seen in Figure 3, panel B, i.e., the heavy chain of the 4F2 cell-surface antigen (SLC3A2; log_2_ Q/C ratios of 1.07) and the cytoplasmic isoform of the hydroxymethylglutaryl-CoA synthase (HMGCS1; log_2_ Q/C of −1.28).

With the aim to functionally characterize the quercetin-induced proteome profile, we analyzed the selected DEPs by Over-Representation Analysis (ORA) of Gene Ontology (GO) annotations [27]. As a starting point, we compared the number of coding genes of DEPs overlapping with the annotated genes in the nonredundant GO Slim subset for the three GO categories “Biological Process”, “Molecular Functions”, and “Cellular Compartment”, as seen in Figure S1. In accordance with this distribution, the 24 h quercetin treatment seems to induce in K562 cells: i) an evident turn-on of signaling activities (74% of upregulated proteins are comprised in the Biological Process term “response to stimulus” versus 34% of downregulated proteins); ii) a marked turn-off of transcriptional and translational processes (80% of downregulated proteins are encompassed in Molecular Function term “nucleic acid binding” versus 15% of the upregulated proteins; 46% of downregulated proteins are comprised in the Cellular Compartment term “ribosome”); and iii) a significant change in the cellular metabolism (according to the distinctive distributions of DEPs in the Molecular Function category).

**Figure 4:**
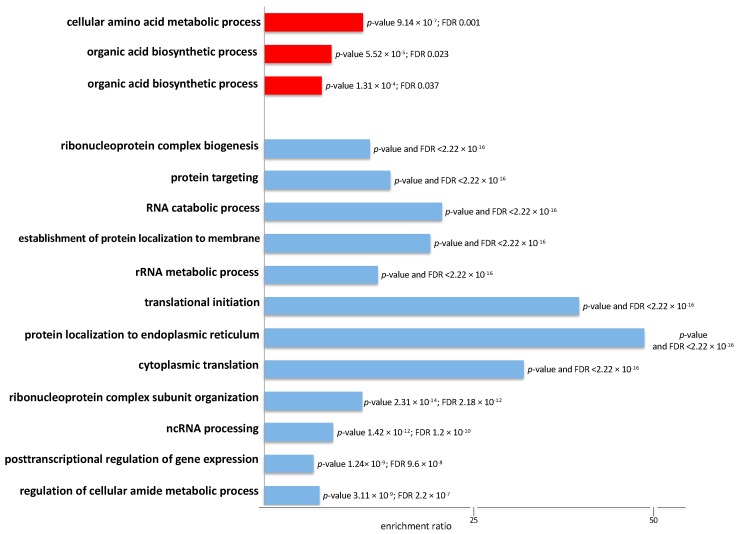
Enrichment analysis of the 112 differentially expressed proteins (DEPs) in K562 cells upon 24 h treatment with quercetin. GO terms found enriched among biological processes (ORA analysis by WebGestalt). X-axis—enrichment ratios. Process categories are listed on the left bar side. *p*-values and FDR values are also showed right next to each bar. The 2.22 × 10^−16^ value is the smallest positive floating-point number in the R platform. Blue bars refer to downregulated, and red bars to upregulated proteins, as listed in Table 1 and Table 2. Statistics are shown in Table S3.

Then, we moved to the ORA analysis using the biological process as a specific category of the selected database. Encoding genes for upregulated proteins were enriched in only three GO sets mainly related to oxidative metabolism. On the other hand, 12 categories, mainly related to protein biosynthetic processes, were identified as enriched by the downregulated DEPs. A graphical representation and statistical details of this analysis are shown in Figure 4 and Table S3, respectively.

The enrichment analysis of DEPs in terms of metabolic pathways confirmed that the early K562 response to the quercetin treatment is marked by the translation shut down, as seen in Table S4. Moreover, the overlap of three DEPs on the Reactome Gene Set R-HSA-2426168 provided an indication, although weak, of a specific alteration in the lipid metabolism in quercetin-treated cells.

### 2.3. Effects of Quercetin on Selected Pathways of K562 Cells

**Figure 5:**
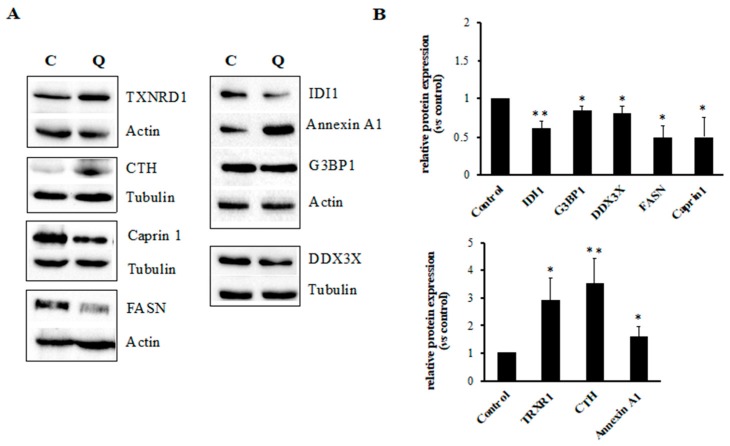
Western blotting analyses in K562 cells treated for 24 h with 25 µM quercetin. (**A**) Representative immunoblots of three independent experiments with similar results; C: control, untreated cells, Q: quercetin-treated cells. (**B**) In the densitometric analysis, after normalization with tubulin or actin protein levels, relative protein expression values have been determined as ratios between the intensities of protein bands in treated and untreated cells, assigning the value 1 to the control. Results represent the mean ± SD of three independent experiments. Statistical evaluation was achieved by Student’s *t*-test. *, *p* < 0.05 vs. control; **, *p* < 0.01 vs. control.

Following proteomic data collection, we used Western blotting to quantitatively determine the relative abundance of protein factors involved in selected cellular pathways, as seen in Figure 5.

First, we measured the expression level of six genes involved at different steps in the complex pathways of protein biosynthesis and cell cycle progression. Specifically, we were able to confirm the decreased expression of two critical factors for the mRNA metabolism (the DEAD-box helicase 3 X-linked or DDX3X, and the Ras GTPase-activating protein-binding protein 1 or G3BP1), and of the cell-cycle-associated protein Caprin1 (cytoplasmic activation/proliferation-associated protein-1). Overall, these data suggested that quercetin is able to induce a fast perturbation of the translational apparatus and of cell cycle progression. Moreover, we were able to confirm increased expression levels for three gene products involved in the amino acid metabolism, i.e., the 4F2 cell-surface antigen heavy chain membrane protein (SLC3A2), involved in the amino acid importing, and the proteins thioredoxin reductase 1 (TXNRD1) and cystathionine gamma-lyase (CTH). Particularly, these latter enzymes are involved not only in the specific metabolism of seleno-amino acids, but also in the cellular activity against reactive oxygen species and in the interconversion of nucleotide di- and triphosphates. Similarly, we were also able to validate proteomic data for the proposed upregulation of the expression of the *ANXA1* gene (annexin A1 protein), known to be involved in many pivotal biological processes, such as cellular transduction, membrane aggregation, inflammation, proliferation, differentiation, and apoptosis.

**Figure 6:**
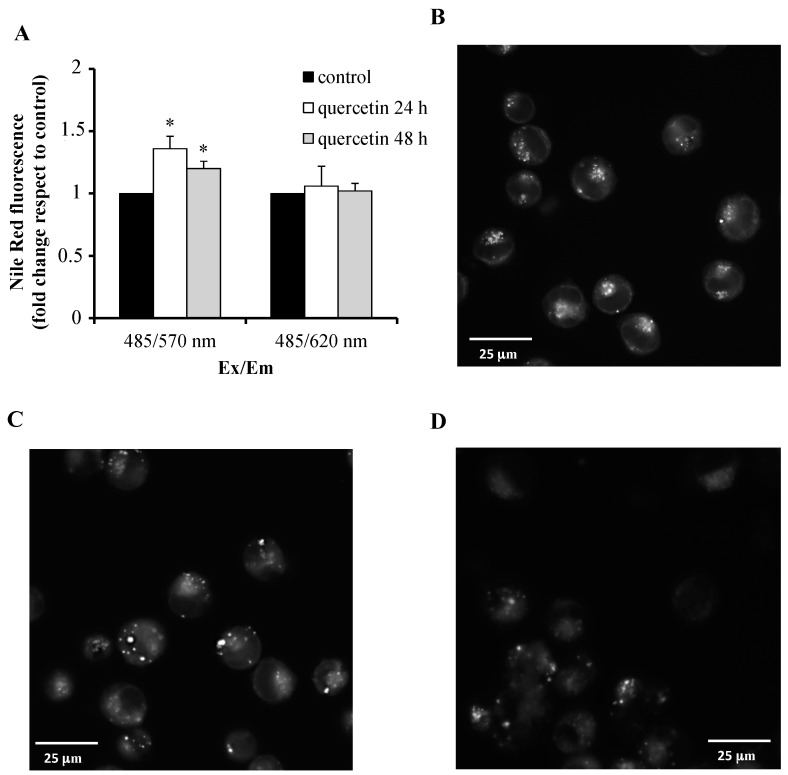
Analysis of lipid content in quercetin-treated K562 cells by Nile Red staining. (**A**) Intracellular lipid content was quantified after Nile Red staining by spectrofluorimetric analysis. Nile Red displays different emission maxima, depending on the hydrophobicity of the bound lipids; Ex/Em 485/570: Nile-Red-stained neutral lipids; Ex/Em 485/620: Nile-Red-stained polar lipids. Values have been obtained by the ratios between the fluorescence of treated and untreated cells, assigning the value 1 to the control. Results represent the mean ± SD of three independent experiments. Statistical evaluation was achieved by Student’s *t*-test. *, Data are significantly different from untreated cells (*p* < 0.05). (**B**–**D**) Neutral-Red-stained neutral lipids of untreated, quercetin-treated for 24 h and 48 h K562 cells, respectively, were visualized by fluorescence microscopy. Scale bars, 25 μm.

Finally, we also focused on lipid metabolism, whose dysregulation is known to negatively contribute to cell growth, proliferation, and survival. We were able to confirm by Western blotting the proteomic data on downregulation of *FASN*, *HMGCS1*, and *IDI1* genes, critical nodes of the lipid transformations in cells. Moreover, with the aim to evaluate the effect of quercetin on the cellular lipid content, we stained K562 cells with Nile Red, a lipophilic fluorescent dye. The fluorescence intensity of Nile-Red-stained cells is directly proportional to lipid content. As shown in panel A of the Figure 6, a mild increase (36% and 20% after 24 and 48 h of treatment, respectively) of fluorescence due to neutral lipid concentration was detected in quercetin-treated cells, compared to control, while polar lipid content did not change. Fluorescence microscopy showed a different morphology and localization of lipid droplets (mainly composed of neutral lipids) in quercetin-treated cells compared to untreated cells. In these last cell samples, many small lipid droplets of similar size, clustered and localized in a polarized manner, have been observed, as seen in Figure 6, panel B. On the other hand, in several quercetin-treated cells, lipid droplets appeared less numerous but increased in size and not clustered, as seen in Figure 6, panels C and D.

## 3. Discussion

K562 cells have been widely used as a model system for testing new synthetic and natural drugs for CML (chronic myeloid leukemia) [28]. In our previous studies, we showed that quercetin (3,3′,4′,5,7-pentahydroxyflavone), one of the main flavonoids widely distributed in the plant kingdom, is able to trigger apoptosis in cultured K562 cells [23,24]. However, the multiplicity of the effects selectively exerted by quercetin on cells [29] requires disclosing the molecular basis of its cytotoxicity by quantitative proteomics.

To our knowledge, quantitative proteomics by SILAC [25] has been applied to assess quercetin-induced alterations in protein expression only on hepatoma HepG2 cells [18,30]. The present study focused on differentially expressed proteins in K562 cells, when treated with quercetin before detecting DNA fragmentation and a marked caspase-3 activation (24 h). The final aim was to use quantitative information of the protein fold changes at the early stage of apoptosis for characterizing quercetin-induced dysregulation of critical K562 signaling, regulative, and metabolic pathways at the proteome level.

In our differential proteome profile following 24 h quercetin treatment, downregulated proteins are predominant, which suggests a fast responsiveness of the cell proteolytic machinery, as seen in Table 2. Bioinformatics referred a massive stop of the K562 translational machinery, as seen in Figure 4, otherwise measured by the viability assay, as seen in Figure 1. When the gene ontology (GO) terms significantly enriched from downregulated proteins were analyzed, we observed that these are mainly composed of ribosomal proteins (RPs). In fact, it is known that RPs may have additional extraribosomal functions unrelated to protein biosynthesis and involved in the regulation of different cellular processes [31]. Remarkably, it has been previously observed that suppression of specific RPs can induce apoptosis [32,33]. Dominance of RPs in the proteome profile induced by quercetin reported here (43% of downregulated proteins) may be used to confirm their role as sentinels for the self-evaluation of cellular health and in the responsiveness to flavonoid treatment.

Beside the biosynthetic machinery and RPs, we were able to prove that the quercetin treatment of K562 cells decreases the expression of the fatty acid synthase (FASN), hydroxymethylglutaryl-CoA synthase (HMGCS1), and isopentenyl-diphosphate delta-isomerase 1 (IDI1), key enzymes of lipid metabolism, as seen in Figure 5. FASN catalyzes the synthesis of long chain fatty acids, while HMGCS1 and IDI1 are two enzymes of the mevalonate pathway of cholesterol synthesis. FASN expression and the levels of cholesterol are much higher in cancer cells than in normal cells, promoting cell proliferation, drug resistance, and tumor progression [34,35]. FASN inhibitors and cholesterol-lowering agents are known to induce apoptosis in cancer cells; therefore, they are considered in anticancer drug development [36,37]. Previous studies have shown that quercetin has potent inhibitory effects on hepatic expression of FASN, with a significant decrease of fatty acid levels associated with induction of apoptosis [38,39]. Our results also confirm quercetin activity on the FASN expression in leukemic cells, as seen in Figure 5. Nevertheless, apoptosis in quercetin-treated K562 cells does not seem to be associated with a decrease of intracellular lipids. On the contrary, Nile Red staining evidenced a mild increase of neutral lipids. This increase, following the induction of apoptosis, could be a consequence of inhibition of mitochondrial fatty acid β-oxidation and storage of fatty acids into triacylglycerols. Then, the downregulation of FASN, HMGCS1, and IDI1 does not cause a significant alteration of lipid content that could promote apoptosis [40]. Because fatty acid and cholesterol synthesis pathways are coordinately regulated by a feedback mechanism mediated by SREBPs (sterol-regulatory element-binding proteins), at this stage of the study, we are not able to rule out that quercetin may also directly affect the K562 SREBPs [41].

Our SILAC results associated the antiproliferative activity of quercetin in K562 cells with the downregulation of three proteins involved in cell cycle progression, i.e., Caprin-1 (cytoplasmic activation/proliferation-associated protein-1), DDX3X (a helicase that contributes to the formation of cytoplasmic stress granules), and G3BP1 (Ras GTPase-activating protein-binding protein 1). In fact, it has been reported that Caprin-1 is involved in cell growth of MCF-7, HeLa, and hepatocellular carcinoma cells [42,43,44], and its suppression in B lymphocyte line DT40 resulted in a prolonged G1 phase and slower proliferation [45]. Moreover, previous studies on the colocalization of Caprin1 and G3BP1 within the cytoplasmic RNA granules in epithelial cells and fibroblasts suggested a role of the Caprin–G3BP1 complex in the translational regulation of protein involved in cell proliferation through a selective binding of the carboxyl-terminal region of Caprin-1 to the Myc proto-oncogene protein c-Myc or to the cyclin D2 mRNAs [46]. On the other hand, besides playing a role in stress granule formation [47], G3BP1 has been also reported as a protein involved in the control of cell proliferation, promoting S-phase entry in fibroblasts [48], in the regulation of apoptosis through interaction with p53 and its translocation [49], and, more recently, in the oncogenic pathways in several human cancers, including breast, gastric, colon, and liver carcinomas [50,51,52,53]. Data on the physical interaction and colocalization of DDX3X with Caprin-1 are also already available [54,55]. DDX3 is a multifunctional protein involved not only in the assembly of RNA–protein complexes during cellular stress, suggesting a role for DDX3 in translational control, but also in other aspects of RNA metabolism (transcriptional regulation of INFβ, p21waf1/cip1, E-cadherin promoters; splicing and nuclear export), in cell-cycle progression, proliferation, and apoptosis [56,57]. DDX3’s role in cancer development is rather complex; it has been described both as tumor suppressor gene by regulating p21 [58] and with oncogenic properties [59]. Data here reported on downexpression of these three proteins (Caprin-1, DDX3X, and G3BP1) suggest a regulation effect of quercetin on stress granule assembly.

Our K562 SILAC profiling traces for a low rate of upregulation after 24 h quercetin treatment. Indeed, we were able to list only a few gene products as potential biomarkers of molecular processes affected by the selective K562 responsiveness to quercetin, as seen in Table 2.

Specifically, the relative abundance of at least three proteins involved in the cellular redox homeostasis (the glutathione reductase or GSR, the peroxiredoxin-5 or PRDX5, and the thioredoxin reductase 1, or TXNRD1) increased in K562 following 24 h exposure to quercetin. The upregulation of these enzymes could not be directly implicated in the apoptotic process triggered by quercetin, but overexpression of antioxidant proteins could be due to endogenous protective response mechanisms. Alterations in the cellular environment induce adaptive mechanisms that lighten or eventually counteract the change. Genes that encode for heat shock proteins and thioredoxin, glutathione, and sirtuin systems are involved in preserving cellular homeostasis during stressful conditions [60]. Nevertheless, upregulation of antioxidant enzymes to restore redox homeostasis can also attenuate ROS signals necessary for physiological cellular activities and therefore indirectly contribute to cell death [61]. Moreover, upregulation of protein expression is not always related to an increased enzyme activity; even if this proteomic analysis showed an increase of GSR expression, in a previous study, quercetin treatment did not modulate GSR activity in K562 cells [23]. In addition, the overexpression of TXNRD1 could not necessarily lead to an increase in its enzymatic activity, as Lu et al. [62] observed that quercetin could irreversibly inhibit thioredoxin reductase.

As a further marker of a cellular responsiveness to stressful conditions, cystathionine gamma-lyase (CTH) was found to be upregulated in K562 cells upon quercetin treatment. CTH utilizes cystathionine generated by cystathionine β-synthase to synthetize cysteine, and it is one of the three enzymes responsible for endogenous sulphydric acid (H_2_S) production from cysteine. CTH is a highly inducible enzyme and is regulated by several factors depending on the cell type in response to a large variety of signals, such as oxidative stress, ER and Golgi stress, mitochondrial stress, inflammation, and starvation [63]. Overexpression of CTH in human melanoma cells induced apoptosis by suppressing the activity of nuclear factor-kB (NF-kB) and decreasing the expression of antiapoptotic proteins [64]. In human aorta smooth muscle cell, CTH upregulation increased H_2_S production and induced apoptosis [65]. Altered, both higher and lower, H_2_S levels can have damaging effects for cells. In fact, it has been shown that activation of the H_2_S pathway can exert both pro- and antiapoptotic activity in cultured cells [66].

Overexpression of CTH can produce, besides the increase of H_2_S, the depletion of cystathionine. Several studies indicated that cystathionine inhibited mitochondria-mediated apoptosis in macrophages by the inhibition of MPTP opening and could rescue liver cells from endoplasmic reticulum stress induced by apoptotic stimuli [67,68]. Cystathionine also protected human breast cancer cells against an excess of reactive oxygen species (ROS) and chemotherapeutic drug-induced apoptosis [69]. Depletion of cystathionine could cause mitochondrial and endoplasmic reticulum instability, which decreases the apoptotic threshold.

In our experiments on K562 cells, quercetin also induced the upregulation of annexin A1, a member of the annexin family, Ca^2+^-regulated, phospholipid-dependent, membrane-binding proteins. Annexin A1 is involved in many pivotal biological processes, such as cellular transduction, membrane aggregation, inflammation, proliferation, differentiation, and apoptosis. Several studies indicate that annexin A1 might function either as a tumor suppressor or a tumor promoter depending on the type of cancer cells [70]. In K562, annexin A1 could have a role as tumor suppressor: Zhu et al. observed annexin A1 downregulation in adriamycin-resistant K562 cells compared with nonresistant cells, and demonstrated that annexin A1 knockdown and overexpression decreased and increased, respectively, adriamycin sensitivity of K562 cells [71]. Likewise, in our study, an annexin A1 upregulation was observed in K562 cells undergoing apoptosis by quercetin. Annexin A1 upregulation has been also reported to be involved in resveratrol-induced apoptosis of human promyelocytic leukemia HL-60 cells [72].

## 4. Materials and Methods

### 4.1. Materials

RPMI 1640 medium and fetal calf serum were from Euroclone. RPMI medium without L-Arginine and L-Lysine (cat. n. R1780), L-Lysine, L-Arginine, L-[4,4,5,5-D4]-Lysine and L-[13C615N4]-Arginine were from Merck, Darmstadt, Germany.

Acridine orange, ethidium bromide, Nile red, quercetin, and polyclonal anti-Caprin1 antibody were purchased from Sigma Chemical Co (St. Louis, MO, USA). Stock solution of quercetin was prepared in DMSO. The DMSO concentration in treated cells was less than 0.01% (*v*/*v*). Monoclonal anti-actin, anti-tubulin, anti-CTH (F-1), anti-G3BP1 (H-10), anti-cHMGCS (A-6), anti-Annexin I (EH17a), anti-IDI1 (XY-7), anti-TrxR1 (B-2), anti-CD98 (E5), anti-DDX3 (2253C5a) and anti-FASN (A-5) antibodies were purchased from Santa Cruz Biotechnology (Santa Cruz, CA, USA). Peroxidase-conjugated secondary IgG antibodies were from Thermo Scientific Inc. (Hudson, NH). Reagents for enhanced chemiluminescence (ECL) detection were obtained from Advasta. Fluorogenic caspase-3 substrate, acetyl-Asp-Glu-Val-aspaminomethylcoumarin (Ac-DEVD-AMC) was from Alexis Biochemicals (San Diego, CA, USA).

All other chemicals were reagent grade.

### 4.2. Cell Culture

Human erythroleukemia cells (K562), obtained from the American Type Culture Collection (ATCC), were grown in RPMI 1640 medium supplemented with 10% dialyzed fetal bovine serum, 100 U/mL penicillin, 100 μg/mL streptomycin and 2 mM glutamine. Cells were maintained in a humidified atmosphere with 5% CO_2_ at 37 °C, with medium renewal every 2–3 days.

For SILAC experiments [25], complete growing media were constituted from medium without L-Arginine and L-Lysine, respectively supplemented with 40 mg/L L-Lysine and 40 mg/L L-Arginine (“light” medium) or 40 mg/L of L-[4,4,5,5-D4]-Lysine and 40 mg/L L-[^13^C6^15^N4]-Arginine (“heavy” medium). The two cell cultures were started in 35 mm culture dishes by suspending cells in fresh “heavy” or “light” media, respectively. Each starting cell culture was expanded for five doubling times to ensure a high level of labeled stable isotope amino acids incorporation in proteins—marked as “K0,R0” and “K4,R10”, respectively, in Figure 2, line A—as determined by MALDI-TOF MS analysis on the housekeeping protein beta-actin (data not shown).

### 4.3. Quercetin Treatment and Cell Lysate Preparation

In “forward” SILAC experiments, the cells cultured in the “heavy” and “light” media were respectively treated with 25 μM quercetin in DMSO or with same amount of the vehicle solution as control. In “reverse” SILAC experiments, media were swapped. Three biological replicates were performed for each label swapped experiment, as seen in Figure 2, line A.

After a 24 h treatment, as seen in Figure 2, line B, cells were collected by centrifugation at 300× *g* and washed three times with ice-cold PBS. The cell pellets were then suspended for 30 min at 4 °C in RIPA buffer containing a protease inhibitor cocktail, with six vortex cycles. Cell lysates were centrifuged 15 min at 12,000× *g* at 4 °C, and the resulting supernatants were collected. Protein concentrations of the cell lysates were determined by the Bradford assay.

### 4.4. SDS-PAGE Prefractionation

For the SILAC analysis, protein mixtures from each pair of differently labeled cells were merged 1:1 on the basis of protein amount, as seen in Figure 2, line C, and loaded on a 12.5% polyacrylamide gel (50 μg/lane). After electrophoresis, gels were stained using Coomassie G250 dye, as seen in Figure 2, line D.

### 4.5. Mass Spectrometry Protein Identification

From each SDS-PAGE lane, eleven slices were excised, washed with a solution of 50 mM ABC (ammonium bicarbonate) buffer containing 50% ACN (acetonitrile), dehydrated with 100% ACN, and speed-vac dried. Reduction on each dried gel piece was achieved with 50 μL 10 mM DTT (dithiothreitol) in a 50 mM ABC buffer by a 30 min incubation at 55 °C. At the end, the DTT solution was removed, 50 μL of 0.5 M IAA (iodoacetamide) were added, and each sample was kept for 15 min at room temperature in the dark. Before the proteolytic step, the reagent excess was washed with a solution containing a 1:1 (*v*/*v*) mixture of 50 mM ABC buffer and ACN, and samples were dried by ACN treatment and speed-vac. Rehydration was then achieved by adding 20 μL of 25 mM ABC buffer containing 5 ng/mL of trypsin at 4 °C, and gel pieces were then incubated for 16 h at 37 °C. Proteolytic peptide mixtures were extracted from the PAGE matrix, dried, and solubilized in 5% ACN/0.1% TFA. Desalting steps were carried out by solid phase extraction (SPE) according to Rappsilber et al. [73]. C18 reverse-phase loaded Empore™ SPE disks were purchased from Supelco (Bellefonte, PA, USA; cat. 66883-U). Prior to mass spectrometric analyses, each peptide mixture was dried, suspended in 100 μL of 0.1% formic acid (FA), and further split in two technical replicates.

All proteomic samples were then sequentially analyzed by nanoliquid chromatography tandem mass spectrometry (nanoLC-MS/MS). For this purpose, an Ultimate 3000 system (Dionex, Sunnyvale, CA, USA) was equipped with a splitting cartridge for nanoflows and connected on-line via a nanoelectrospray ion source (Thermo-Fisher Scientific, Waltham, MA, USA) to an LTQ-Orbitrap XL mass spectrometer (Thermo-Fisher Scientific). Each sample was automatically loaded from the autosampler module at a flow rate of 20 μL/min onto a trap column (Acclaim^®^PepMap™ μ-Precolumn, 300 μm × 1 mm, Dionex) in 4% ACN containing 0.1% TFA. After 4 min, peptides were eluted at 300 nL/min onto a 15 cm column (360 μm OD × 75 μm ID, 15 μm Tip ID; PicoFrit^®^, New Objective, Woburn, MA, USA), custom packed with a reverse phase (C18, 5 μm particle size, 200 Å pore size; Magic C18 AQ, Michrom), by a two-step gradient of ACN in 0.1% FA (from 5% to 40% in 120 min, and from 40% to 85% in 15 min). At the end of each run, eluent was set back to 4% ACN in 0.1% FA, and column left to equilibrate for 20 min.

As peptides eluted, they were on-line injected and analyzed by LTQ-Orbitrap as in Correani et al. [74]. In particular, tandem mass (MS/MS) spectra were acquired with a data-dependent top-five method, selecting the five most intense ions with ≥2 charge states detected per survey scan if they exceeded an intensity of at least 200 counts. To avoid redundant sequencing of the most abundant peptides, dynamic exclusion was enabled with repeat count of 1, repeat duration of 30 s, exclusion list size of 300, and exclusion duration of 90 s.

### 4.6. Proteomics Analysis

The raw files from the LC-MS/MS analysis were uploaded to the quantitative proteomics software package MaxQuant (version 1.6.0.16; Max Planck Institute of Biochemistry, Martinsried, DE) [75]. Specifically, the Andromeda search engine was configured for the SwissProt Human Protein Database (release 2017_09; 8,747,138 residues; 19,083 sequences; EMBL-EBI, Hinxton Cambridge, UK), and for two built-in databases in which, respectively, sequences have been reversed (decoy database) and a common contaminants list was encompassed. Carbamidomethylation of Cysteine residues was imposed as a fixed modification; oxidation of methionine residues and acetylation on protein N-terminus were accepted as variable modifications; doublets for natural and isotope stable labeled arginine (delta mass 10 amu) and lysine (delta mass 4 amu) residues were imposed in the identification parameters. Alignment between contiguous HPLC runs was activated. The peptide false discovery rate (FDR) was set to 0.01. For protein identification, the minimum peptide length considered was 7, FDR was set to 0.01, and validation was based on q-value. “Requantification” function was flagged. In quantification, unique and razor peptides were considered. All further identification and quantification parameters were set as default.

Identification data mining was performed on the Perseus computational platform (ver. 1.6.1.7; Max Planck Institute of Biochemistry, Martinsried, Germany) [76]. Firstly, proteins identified at least one time in the “forward” and “reverse” biological replicates gave rise to a starting matrix (2406 identification hits). Then, rows of this matrix have been further filtered for identifications found in the reverse decoy and in the contaminant databases (filtered matrix: 2331 identification hits). A more confident list was finally obtained accepting only proteins identified in both “forward” and “reverse” experiments with at least four peptides and a minimum of two unique peptides (final identification list: 1703 hits, Table S1).

### 4.7. Quantitative Data Analysis

As parameter of protein relative abundances between quercetin treated and control (quercetin untreated) cells we used the “H/L normalized” ratios of the MaxQuant output (Q/C ratios in this manuscript). Only a) all rows that showed less than two valid ratio values in each of the two groups of label-swapped replicates, and b) showing variability of the among all replicates higher than 30%, were accepted as the K562 quantified proteome (1206 quantified protein hits; Table S2).

One-sample *t*-test was selected as method to determine their statistical significance among the 12 replicas (i.e., two label swapped starting cell cultures × three independent biological replicates × two technical replicates for each sample set up for the LCMSMS analysis). Settings were: a) difference in log_2_ Q/C ratios among replicates equal to 0 as null hypothesis, b) multiple testing adjustment of *p*-values according to the Benjamini–Hochberg method, c) a FDR value of 0.05 as cut-off, and d) truncation of the outliers at both sides.

Proteins observed with log_2_ Q/C ≥|0.5| (approximately, a fold change of more than ±1.5 times), and with a statistical significance (−log_10_ *p*-value ≥1.54) were arbitrarily accepted as differentially expressed proteins (DEFs), as seen in Table 1 and Table 2.

### 4.8. Functional Analysis of DEPs

The WEB-based GEne SeT AnaLysis Toolkit (WebGestalt 2019; http://webgestalt.org/) [27] was employed for the enrichment analysis of the differentially expressed protein coding gene set we determined, as seen in Table 1 and Table 2. *Homo sapiens* was the selected organism, geneontology was the functional database (daily build at the WebGestalt site), and genome protein coding was the reference list for the Over-Representation Analysis (ORA). The GO SLIM database of the WebGestalt platform, including only the first levels below “Biological Process”, “Molecular Function” and “Cellular Process”, was first employed for determining the overlap (“category size”) between the two categories of the terms of the Gene Ontology (GO) annotations and the quercetin-induced DEPs, as seen in Figure S1. Then, in the ORA, we set a minimum number of five identifications accepted for “category size”, the Benjamini–Hochberg method for multiple test adjustment of the *p*-values from the hypergeometric test, and FDR (false discovery rate) value ≤ 0.05 for testing a significance level of “enrichments”. For the Enrichment Analysis of metabolic pathways-encompassed DEPs, we set i) the Reactome database (Release 88.2, 11/01/2018) as original source of the reference dataset, ii) the minimum number of identifications accepted for category to two, and iii) the mode “weighted set cover” to find top gene sets while maximizing gene coverage. All further parameters were set as default.

### 4.9. Analysis of Cell Proliferation and Viability

Cells were seeded at a density of 1 × 10^5^ per mL and incubated in the absence or in the presence of 25 µM quercetin. After 24 and 48 h, cells were counted and viability determined by trypan blue exclusion assay.

### 4.10. Apoptosis Evaluation

Nuclear morphology was assessed by acridine orange/ethidium bromide double staining assay. After washing with PBS, cells were stained with a fluorescent solution containing 100 µg/mL ethidium bromide and 100 µg/mL acridine orange in PBS and immediately observed with a fluorescence microscope. Cells showing condensed and fragmented chromatin were considered apoptotic. A minimum of 400 cells was counted for each determination.

### 4.11. Caspase-3 Activity

Cells were washed with PBS and then lysed in a buffer containing 50 mM Tris-HCl, pH 7.4, 10 mM EGTA, 1 mM EDTA, 10 mM DTT, 1% (*v*/*v*) Triton X-100, for 30 min at 4 °C. After centrifugation at 15,000× *g* for 15 min at 4 °C, supernatants were collected and used for detection of caspase activity.

Cell lysate (60 µg of proteins) was mixed with 20 μM fluorogenic caspase-3 peptide substrate, Ac-DEVD-AMC, in the reaction buffer (50 mM Tris–HCl, pH 7.4, 10 mM EGTA, 1 mM EDTA, 10 mM DTT). The reaction mixture was incubated for 30 min, at 37 °C.

Fluorescence was measured on a Perkin-Elmer LS-50B spectrofluorometer, with excitation at 380 nm and emission at 460 nm.

### 4.12. Western Blotting Analysis

The expression levels of targeted proteins were determined using Western blotting assays. Cells were collected after quercetin treatment (same conditions as above), washed with PBS, and lysed for 30 min at 4 °C in the RIPA buffer containing a suitable cocktail of protease inhibitors. Proteins were separated on a 12.5% SDS-PAGE (50 µg/lane) and transferred to polyvinylidene difluoride (PVDF) membranes. Membranes were blocked with 5% (*w*/*v*) nonfat dry milk and immunoblotted with suitably diluted primary antibodies followed by secondary antibodies (goat antirabbit or antimouse IgG and rabbit antigoat IgG) conjugated with horseradish peroxidase. Bands were visualized using a chemiluminescent detection system (Thermo Scientific, Rockford, IL, USA), quantified by ImageJ software (version 1.44; NIH, Bethesda, MD, USA) and normalized by internal reference. Assays were performed at least three times independently.

### 4.13. Lipid Content

Cells were washed with PBS and fixed in 4% paraformaldehyde for 30 min at 4 °C. Then, cells were washed with PBS and cellular lipids were stained with 0.5 µg/mL Nile Red for spectrofluorimetric analysis and with 0.1 µg/mL Nile Red for fluorescence microscopy. Fluorescence of Nile-Red-stained lipids was measured on a Perkin-Elmer LS-50B spectrofluorometer, setting at excitation 485 nm and emission 570 nm for neutral lipids and at excitation 485 nm and emission at 620 nm for polar lipids. Stained cells were observed on fluorescence microscope with both FITC and rhodamine filter.

## 5. Conclusions

On the whole, our proteomic analysis provides a robust list of up- and downregulations that quercetin treatment induces at 24 h in K562 cells, namely at the early stages of the apoptosis response. Even if the proteome changes we observed are relatively small (only few coding genes have been found with a relative abundance double or half than in control state), specific modulation of proteins involved in translational machinery, RNA metabolism, antioxidant defense systems, and lipid metabolism has been determined.

It is doubtless that further orthogonal approaches have to be employed to validate the proteins listed here as key elements of the quercetin effects on chronic myeloid leukemia cells in blast crisis. Nevertheless, the data reported in the present manuscript represent a preliminary but robust snapshot of the cell toxicity of this widely used flavonoid. In the future, high throughput screening of the expression of biomarkers provided here in K562-knockout cells will help in revealing the complete landscape of modulated pathways which push these cells into apoptosis. Moreover, quantitative comparison by multiplex analysis (i.e., iTRAQ) of the proteome signatures induced by quercetin among other CML cells expressing the fusion gene *BCR-ABL1* will help in highlighting the quercetin effects at its pivotal biological processes. Finally, the in-depth knowledge of the biochemical mechanism(s) of quercetin-induced cytotoxicity could drive precise drug design against this kind of human cancer.

## Figures and Tables

**Table 1:** Upregulated proteome in quercetin-treated K562 cells.

Protein Names	Gene Symbol	Unique Peptides ^a^	Sequence Coverage [%] ^b^	log_2.0_ Q/C ^c^	*p*-Value ^d^
4F2 cell-surface antigen heavy chain	*SLC3A2*	25	43.8	1.07	1.65 × 10^−12^
Glutamate–cysteine ligase regulatory subunit	*GCLM*	4	21.2	0.97	2.32 × 10^−6^
Thioredoxin reductase 1. Cytoplasmic	*TXNRD1*	16	43.8	0.93	5.17 × 10^−7^
Acid ceramidase; Acid ceramidase subunit alpha; Acid ceramidase subunit beta	*ASAH1*	9	30.1	0.84	1.37 × 10^−4^
Regulation of nuclear pre-mRNA domain-containing protein 1B	*RPRD1B*	7	32.8	0.74	1.80 × 10^−5^
Glutathione reductase. mitochondrial	*GSR*	17	57.5	0.74	5.40 × 10^−13^
Cystathionine gamma-lyase	*CTH*	10	40.2	0.69	5.88 × 10^−6^
Replication protein A 70 kDa DNA-binding subunit	*RPA1*	10	29.7	0.68	4.03 × 10^−4^
Synaptic vesicle membrane protein VAT-1 homolog	*VAT1*	18	68.4	0.66	1.40 × 10^−9^
Ferritin heavy chain	*FTH1*	7	45.9	0.66	1.96 × 10^−3^
Annexin A5	*ANXA5*	18	64.4	0.62	1.86 × 10^−7^
D-3-phosphoglycerate dehydrogenase	*PHGDH*	17	46.5	0.62	3.01 × 10^−3^
Carbonyl reductase [NADPH] 1	*CBR1*	8	53.1	0.59	2.23 × 10^−7^
Coronin-1C	*CORO1C*	13	36.5	0.57	1.22 × 10^−5^
Hexokinase-1	*HK1*	31	41.8	0.57	2.30 × 10^−3^
Ferrochelatase. Mitochondrial	*FECH*	11	46.8	0.57	2.62 × 10^−4^
Glycine–tRNA ligase	*GARS*	27	50.6	0.57	1.47 × 10^−6^
Sorting nexin-6; Sorting nexin-6. N-terminally processed	*SNX6*	7	27.3	0.56	1.19 × 10^−3^
Annexin A1	*ANXA1*	31	75.4	0.55	1.08 × 10^−5^
Adipocyte plasma membrane-associated protein	*APMAP*	7	24.0	0.55	7.53 × 10^−5^
Protein phosphatase 1F	*PPM1F*	15	53.1	0.53	4.63 × 10^−3^
Selenide. Water dikinase 1	*SEPHS1*	4	15.6	0.53	3.80 × 10^−4^
Glutamine–fructose-6-phosphate aminotransferase [isomerizing] 1	*GFPT1*	20	39.3	0.53	9.48 × 10^−11^
Transaldolase	*TALDO1*	22	52.5	0.52	1.76 × 10^−6^
Alanine–tRNA ligase. Cytoplasmic	*AARS*	43	57.3	0.52	1.32 × 10^−7^
Carbonic anhydrase-related protein	*CA8*	7	36.2	0.52	9.72 × 10^−6^
Porphobilinogen deaminase	*HMBS*	15	56.2	0.51	1.50 × 10^−3^

^a^ Total number of peptide sequences exclusively assigned to the protein group. The sequence coverage can be used to qualitatively estimate the level of confidence in protein identification. ^b^ Sequence coverage of the best protein sequence contained in the group by the identified peptides, expressed as %. ^c^ Q/C ratios stands for relative abundances, evaluated as ratios of the peptide MS intensity pairs between quercetin treated (Q) and untreated cells (C). It is expressed as log_2_. ^d^ *p*-value obtained by one sample test with Benjamini–Hochberg correction.

**Table 2:** Downregulated proteome in quercetin-treated K562 cells.

Protein Names	Gene Symbol	Unique Peptides ^a^	Sequence Coverage [%] ^b^	log_2_ Q/C ^c^	*p*-Value ^d^
Hydroxymethylglutaryl-CoA synthase. Cytoplasmic	*HMGCS1*	21	65.0	−1.28	6.70 × 10^−12^
Ribonucleoside-diphosphate reductase subunit M2	*RRM2*	5	22.4	−1.07	4.78 × 10^−3^
60S acidic ribosomal protein P2	*RPLP2*	10	97.4	−1.02	1.64 × 10^−3^
60S ribosomal protein L13a	*RPL13A*	14	49.8	−1.00	1.08 × 10^−4^
60S acidic ribosomal protein P1	*RPLP1*	5	95.6	−1.00	1.15 × 10^−2^
60S ribosomal protein L9	*RPL9*	15	72.4	−0.95	1.18 × 10^−4^
Cold shock domain-containing protein E1	*CSDE1*	10	16.3	−0.95	1.14 × 10^−10^
60S ribosomal protein L18	*RPL18*	9	35.1	−0.92	2.35 × 10^−4^
60S ribosomal protein L18a	*RPL18A*	19	61.9	−0.92	1.72 × 10^−5^
60S ribosomal protein L7	*RPL7*	18	48.4	−0.92	9.53 × 10^−5^
N-acetyltransferase 10	*NAT10*	18	25.2	−0.90	2.15 × 10^−3^
H/ACA ribonucleoprotein complex subunit 2	*NHP2*	4	31.4	−0.90	4.11 × 10^−3^
60S ribosomal protein L10	*RPL10*	13	46.7	−0.89	7.20 × 10^−4^
60S ribosomal protein L17	*RPL17*	13	50.5	−0.89	3.07 × 10^−6^
Nucleoplasmin-3	*NPM3*	5	36.5	−0.88	1.01 × 10^−5^
DnaJ homolog subfamily A member 1	*DNAJA1*	12	43.8	−0.88	1.50 × 10^−7^
Thymidylate synthase	*TYMS*	10	41.5	−0.87	1.92 × 10^−10^
60S ribosomal protein L4	*RPL4*	23	46.4	−0.87	1.30 × 10^−2^
Caprin-1	*CAPRIN1*	10	20.3	−0.86	2.81 × 10^−6^
Ribosome biogenesis protein BRX1 homolog	*BRIX1*	8	25.8	−0.86	1.08 × 10^−3^
60S ribosomal protein L7a	*RPL7A*	17	51.1	−0.85	1.02 × 10^−5^
60S ribosomal protein L27	*RPL27*	11	54.4	−0.84	3.65 × 10^−4^
1.2-dihydroxy-3-keto-5-methylthiopentene dioxygenase	*ADI1*	9	60.3	−0.84	2.13 × 10^−6^
60S ribosomal protein L21	*RPL21*	11	48.1	−0.83	1.93 × 10^−5^
40S ribosomal protein S7	*RPS7*	18	68.6	−0.82	8.50 × 10^−4^
60S ribosomal protein L30	*RPL30*	9	68.7	−0.82	6.54 × 10^−5^
Transcription factor BTF3	*BTF3*	9	63.1	−0.81	1.55 × 10^−10^
40S ribosomal protein S4. X isoform	*RPS4X*	22	60.8	−0.80	3.20 × 10^−3^
Polyadenylate-binding protein 1; Polyadenylate-binding protein 3	*PABPC1;* *PABPC3*	17	42.3	−0.79	1.43 × 10^−8^
60S ribosomal protein L3	*RPL3*	13	36.0	−0.79	4.37 × 10^−4^
40S ribosomal protein S13	*RPS13*	14	62.9	−0.77	1.13 × 10^−3^
60S ribosomal protein L23a	*RPL23A*	14	47.4	−0.77	2.17 × 10^−4^
40S ribosomal protein S8	*RPS8*	15	60.1	−0.77	2.45 × 10^−4^
Probable ATP-dependent RNA helicase DDX47	*DDX47*	4	14.1	−0.76	2.25 × 10^−4^
Probable ATP-dependent RNA helicase DDX5	*DDX5*	19	48.0	−0.75	3.39 × 10^−6^
40S ribosomal protein S28	*RPS28*	8	79.7	−0.74	8.64 × 10^−4^
Eukaryotic translation initiation factor 3 subunit K	*EIF3K*	10	53.2	−0.73	4.93 × 10^−8^
60S ribosomal protein L8	*RPL8*	11	42.4	−0.73	2.50 × 10^−6^
60S ribosomal protein L6	*RPL6*	18	52.8	−0.73	9.20 × 10^−5^
60S ribosomal protein L10a	*RPL10A*	12	42.4	−0.72	3.42 × 10^−5^
60S ribosomal protein L36	*RPL36*	6	33.3	−0.70	8.17 × 10^−6^
40S ribosomal protein S10	*RPS10*	13	65.5	−0.67	8.80 × 10^−4^
Plasminogen activator inhibitor 1 RNA-binding protein	*SERBP1*	9	25.2	−0.65	2.38 × 10^−4^
Eukaryotic translation initiation factor 3 subunit E	*EIF3E*	17	43.1	−0.65	1.67 × 10^−5^
40S ribosomal protein S25	*RPS25*	9	41.6	−0.65	1.13 × 10^−3^
Cytochrome c oxidase subunit 2	*MT-CO2*	6	32.2	−0.64	1.05 × 10^−8^
Eukaryotic translation initiation factor 4 gamma 2	*EIF4G2*	8	12.6	−0.63	2.89 × 10^−2^
Nascent polypeptide-associated complex subunit alpha	*NACA*	6	34.9	−0.62	1.98 × 10^−2^
Importin subunit alpha-1	*KPNA2*	22	68.1	−0.62	6.26 × 10^−7^
Eukaryotic translation initiation factor 3 subunit D	*EIF3D*	12	29.7	−0.62	4.93 × 10^−6^
Ubiquitin-conjugating enzyme E2 S	*UBE2S*	7	46.4	−0.62	3.32 × 10^−5^
40S ribosomal protein S6	*RPS6*	11	38.2	−0.62	1.37 × 10^−4^
60S ribosomal protein L35	*RPL35*	6	35.8	−0.61	2.68 × 10^−3^
40S ribosomal protein S15a	*RPS15A*	13	76.2	−0.61	6.03 × 10^−3^
Myosin light polypeptide 6	*MYL6*	8	55.6	−0.60	1.21 × 10^−3^
Fatty acid synthase	*FASN*	121	65.1	−0.60	5.50 × 10^−6^
Superkiller viralicidic activity 2-like 2	*SKIV2L2*	22	28.3	−0.60	1.64 × 10^−5^
40S ribosomal protein S20	*RPS20*	7	49.6	−0.59	6.47 × 10^−7^
Ras GTPase-activating protein-binding protein 2	*G3BP2*	6	22.4	−0.59	1.13 × 10^−2^
Nucleolar RNA helicase 2	*DDX21*	31	50.4	−0.59	2.20 × 10^−3^
40S ribosomal protein S3a	*RPS3A*	19	61.0	−0.59	2.01 × 10^−2^
Lamin-B1	*LMNB1*	12	30.4	−0.58	3.56 × 10^−5^
Elongation factor 2	*EEF2*	55	69.8	−0.58	3.54 × 10^−3^
Ribonucleoside-diphosphate reductase large subunit	*RRM1*	22	43.7	−0.58	7.04 × 10^−13^
60S ribosomal protein L27a	*RPL27A*	9	45.3	−0.58	6.26 × 10^−10^
Heat shock 70 kDa protein 4L	*HSPA4L*	15	30.0	−0.56	1.94 × 10^−3^
Nucleolin	*NCL*	32	36.1	−0.56	7.91 × 10^−10^
RNA-binding motif protein. X chromosome; RNA-binding motif protein. X chromosome. X-linked-like-1	*RBMX; RBMXL1*	6	24.0	−0.56	5.60 × 10^−2^
YrdC domain-containing protein. mitochondrial	*YRDC*	7	36.2	−0.56	2.86 × 10^−3^
60S ribosomal protein L5	*RPL5*	14	40.4	−0.56	2.72 × 10^−3^
40S ribosomal protein S3	*RPS3*	21	69.1	−0.56	1.60 × 10^−7^
Multifunctional methyltransferase subunit TRM112-like protein	*TRMT112*	6	55.2	−0.55	5.01 × 10^−4^
Isopentenyl-diphosphate Delta-isomerase 1	*IDI1*	8	37.9	−0.55	6.07 × 10^−4^
Inositol hexakisphosphate and diphosphoinositol-pentakisphosphate kinase 2	*PPIP5K2*	13	14.7	−0.55	1.16 × 10^−10^
Mitotic spindle assembly checkpoint protein MAD2A	*MAD2L1*	6	34.6	−0.55	1.50 × 10^−8^
Ras GTPase-activating protein-binding protein 1	*G3BP1*	10	38.4	−0.54	6.09 × 10^−7^
Phosducin-like protein 3	*PDCL3*	6	36.0	−0.54	1.10 × 10^−4^
DNA (cytosine-5)-methyltransferase 1	*DNMT1*	7	6.3	−0.53	1.07 × 10^−3^
Heterogeneous nuclear ribonucleoprotein A3	*HNRNPA3*	15	42.9	−0.53	1.79 × 10^−4^
ATP-dependent RNA helicase DDX3X	*DDX3X*	7	40.5	−0.53	6.37 × 10^−2^
40S ribosomal protein S19	*RPS19*	15	63.4	−0.53	1.61 × 10^−3^
Medium-chain specific acyl-CoA dehydrogenase. mitochondrial	*ACADM*	9	31.1	−0.52	1.66 × 10^−2^
Myb-binding protein 1A	*MYBBP1A*	24	26.8	−0.52	1.63 × 10^−5^
Enhancer of mRNA-decapping protein 4	*EDC4*	9	10.8	−0.51	1.05 × 10^−3^
60S ribosomal protein L35a	*RPL35A*	12	49.1	−0.51	1.07 × 10^−3^

^a^ Total number of peptide sequences exclusively assigned to the protein group. The sequence coverage can be used to qualitatively estimate the level of confidence in protein identification. ^b^ Sequence coverage of the best protein sequence contained in the group by the identified peptides, expressed as %. ^c^ Q/C ratios stand for relative abundances, evaluated as ratios of the peptide MS intensity pairs between quercetin treated (Q) and untreated cells (C). It is expressed as log_2_. ^d^ *p*-value obtained by one sample test with Benjamini–Hochberg correction.

## Supplementary Materials

Supplementary materials can be found at https://www.mdpi.com/1422-0067/21/1/32/s1.

## Abbreviations

ABC	Ammonium bicarbonate buffer
ABL	Abelson leukemia virus oncogene
ACN	Acetonitrile
ATCC	American Type Culture Collection
BCR	Break-point cluster region gene
CML	Chronic myeloid leukemia
DEPs	Differentially expressed proteins
DTT	Dithiothreitol
FA	Formic acid
FDR	False discovery rate
GO	Gene Ontology
IAA	Iodoacetamide
LC-MS/MS	Liquid chromatography tandem mass spectrometry
MS/MS	Tandem mass spectrometry
ORA	Over-Representation Analysis
Ph	Philadelphia chromosome
PVDF	Polyvinylidene difluoride
ROS	Reactive oxygen species
SD	Standard deviation
SILAC	Stable isotope metabolic labelling of amino acid
SPE	Solid phase extraction
TFA	Trifluoracetic acid
TKI	Tyrosine kinase inhibitor
WebGestalt	WEB-based GEne SeT AnaLysis Toolkit
