# Supplementary material for: Proteomic Analysis of Quercetin-Treated K562 Cells
Source: Int J Mol Sci. 2019 Dec 19;21(1):32. doi: 10.3390/ijms21010032 (PMC6981597; doi:10.3390/ijms21010032)
Supplement: Supplementary file 1 [file ijms-21-00032-s001.zip › jms-665671_Supplemetary materials/Figure S1 and captions.pdf]

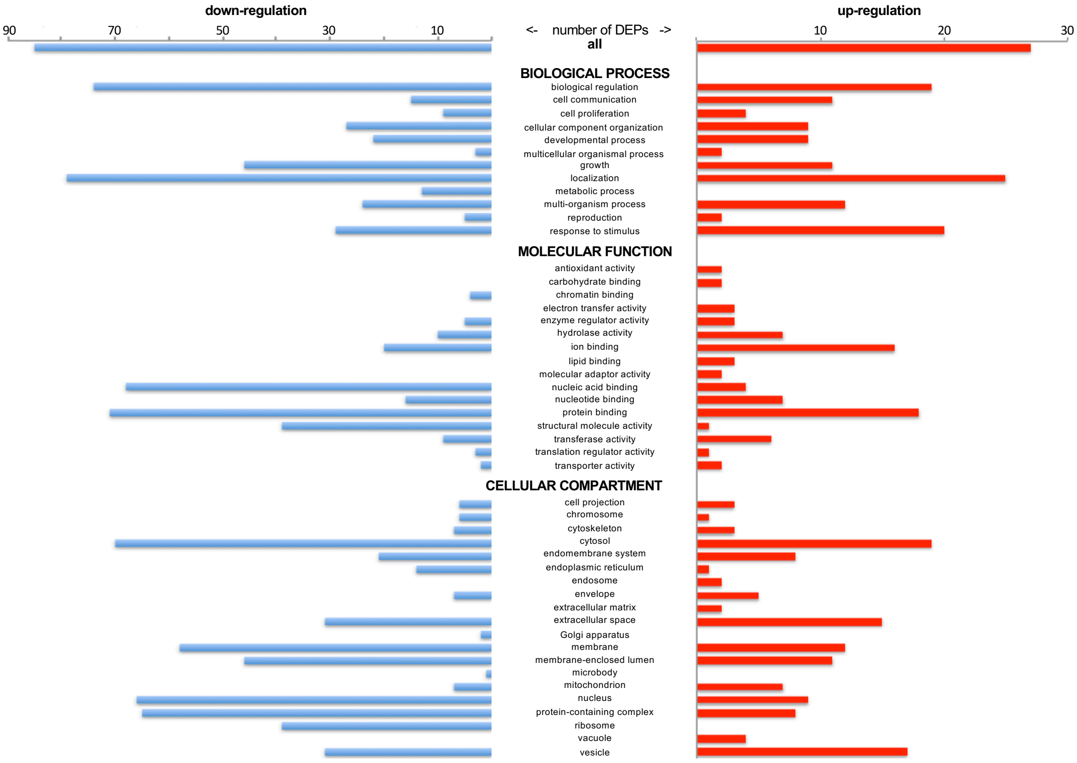

Figure S1. Two-side bar comparison of distribution among GO Slim categories of the 107 differentially expressed proteins (DEPs) in K562 cells upon 24 h treatment with quercetin. GO Slim terms from biological process (upper group), cellular component (medial group) and molecular function (lower group) ontologies overlapping with coding genes for DEPs are vertically ranked. Blue bars refer to down regulated, and red bars to up regulated proteins, as listed in Tables I and II. Bar length indicates the number of the overlapping genes ("category size" in the following "enrichment" analysis). For sake of comparison, the horizontal axes have been proportionally scaled on the basis of the global number of proteins compared.
