# Supplementary material for: Proteomic Analysis of Quercetin-Treated K562 Cells
Source: Int J Mol Sci. 2019 Dec 19;21(1):32. doi: 10.3390/ijms21010032 (PMC6981597; doi:10.3390/ijms21010032)
Supplement: Supplementary file 1 [file ijms-21-00032-s001.zip › jms-665671_Supplemetary materials/Table S1.pdf]

Table S1

Protein IDs[a]	Majority protein IDs[b]	Protein names	Gene names	Peptides[c]	Razor + unique peptides[d]	Unique peptides[e]	Identification type[f]												Sequence coverage [%][g]	Mol. weight [kDa][h]	Q-value[i]	Score[l]
							FRW1	FRW1a	FRW2	FRW2a	FRW3	FRW3a	REV1	REV1a	REV2	REV2a	REV3	REV3a				
P49588	P49588	Alanine--tRNA ligase, cytoplasmic	AARS	43	43	43	By MS/MS	By MS/MS	By MS/MS	By MS/MS	By MS/MS	By MS/MS	By MS/MS	By MS/MS	By MS/MS	By MS/MS	By MS/MS	By MS/MS	57,3	106,81	0	323,31
Q5JTZ9	Q5JTZ9	Alanine--tRNA ligase, mitochondrial	AARS2	7	7	7		By MS/MS	By MS/MS	By MS/MS		By MS/MS				By MS/MS	By MS/MS		13,9	107,34	0	16,36
Q9NRN7	Q9NRN7	L-aminoadipate-semialdehyde dehydrogenase-phosphopantetheinyl transferase	AASDHPPT	8	8	8	By MS/MS	By MS/MS	By MS/MS	By MS/MS	By MS/MS		By MS/MS	By MS/MS	By MS/MS	By MS/MS	By MS/MS	By MS/MS	24,9	35,78	0	103,68
P61221	P61221	ATP-binding cassette sub-family E member 1	ABCE1	22	22	22	By MS/MS	By MS/MS	By MS/MS	By MS/MS	By MS/MS	By MS/MS	By MS/MS	By MS/MS	By MS/MS	By MS/MS	By MS/MS	By MS/MS	42,6	67,31	0	323,31
Q8NE71	Q8NE71	ATP-binding cassette sub-family F member 1	ABCF1	14	14	14	By MS/MS	By MS/MS	By MS/MS	By MS/MS	By MS/MS	By MS/MS	By MS/MS	By MS/MS	By MS/MS	By MS/MS	By MS/MS	By MS/MS	18,5	95,93	0	226,51
Q9UG63	Q9UG63	ATP-binding cassette sub-family F member 2	ABCF2	7	7	7		By MS/MS		By MS/MS		By MS/MS	By MS/MS	By MS/MS	By MS/MS	By MS/MS	By MS/MS	By MS/MS	14,4	71,29	0	11,27
Q9NUJ1	Q9NUJ1	Mycophenolic acid acyl-glucuronide esterase, mitochondrial	ABHD10	4	4	4	By MS/MS	By MS/MS	By MS/MS	By MS/MS	By MS/MS	By MS/MS		By MS/MS	By MS/MS			By MS/MS	20,3	33,93	0	49,28
Q96IU4	Q96IU4	Alpha/beta hydrolase domain-containing protein 14B	ABHD14B	6	6	6	By MS/MS	By MS/MS	By MS/MS	By MS/MS	By MS/MS	By MS/MS	By MS/MS	By MS/MS	By MS/MS	By MS/MS	By MS/MS	By MS/MS	39,5	22,35	0	39,01
P09110	P09110	3-ketoacyl-CoA thiolase, peroxisomal	ACAA1	4	4	4	By MS/MS	By MS/MS	By MS/MS	By MS/MS	By MS/MS	By MS/MS	By MS/MS		By MS/MS	By MS/MS			18,6	44,29	0	31,01
Q13085	Q13085	Acetyl-CoA carboxylase 1;Biotin carboxylase	ACACA	48	48	41	By MS/MS	By MS/MS	By MS/MS	By MS/MS	By MS/MS	By MS/MS	By MS/MS	By MS/MS	By MS/MS	By MS/MS	By MS/MS	By MS/MS	30,9	265,55	0	132,96
P11310	P11310	Medium-chain specific acyl-CoA dehydrogenase, mitochondrial	ACADM	9	9	9	By MS/MS	By MS/MS	By MS/MS	By MS/MS	By MS/MS	By MS/MS	By MS/MS		By MS/MS	By MS/MS			31,1	46,59	0	323,31
P49748	P49748	Very long-chain specific acyl-CoA dehydrogenase, mitochondrial	ACADVL	13	13	13	By MS/MS	By MS/MS	By MS/MS	By MS/MS	By MS/MS	By MS/MS	By MS/MS	By MS/MS			By MS/MS	By MS/MS	30,2	70,39	0	69,83
P24752	P24752	Acetyl-CoA acetyltransferase, mitochondrial	ACAT1	24	24	24	By MS/MS	By MS/MS	By MS/MS	By MS/MS	By MS/MS	By MS/MS	By MS/MS	By MS/MS	By MS/MS	By MS/MS			61,8	45,20	0	323,31
Q9BWD1	Q9BWD1	Acetyl-CoA acetyltransferase, cytosolic	ACAT2	12	12	12	By MS/MS	By MS/MS	By MS/MS	By MS/MS	By MS/MS	By MS/MS	By MS/MS	By MS/MS	By MS/MS	By MS/MS			65,7	41,35	0	109,25
Q9H3P7	Q9H3P7	Golgi resident protein GCP60	ACBD3	5	5	5	By MS/MS	By MS/MS	By MS/MS	By MS/MS	By MS/MS	By MS/MS	By MS/MS	By MS/MS	By MS/MS	By MS/MS	By MS/MS	By MS/MS	12,9	60,59	0	142,92
P53396	P53396	ATP-citrate synthase	ACLY	49	49	49	By MS/MS	By MS/MS	By MS/MS	By MS/MS	By MS/MS	By MS/MS	By MS/MS	By MS/MS	By MS/MS	By MS/MS	By MS/MS	By MS/MS	53,4	120,84	0	323,31
P21399	P21399	Cytoplasmic aconitate hydratase	ACO1	14	14	14	By MS/MS	By MS/MS	By MS/MS	By MS/MS	By MS/MS	By MS/MS	By MS/MS	By MS/MS	By MS/MS	By MS/MS	By MS/MS	By MS/MS	27,7	98,40	0	199,29
Q99798	Q99798	Aconitate hydratase, mitochondrial	ACO2	24	24	24	By MS/MS	By MS/MS	By MS/MS	By MS/MS	By MS/MS	By MS/MS	By MS/MS	By MS/MS	By MS/MS	By MS/MS	By MS/MS	By MS/MS	40,9	85,42	0	210,75
Q9NPJ3	Q9NPJ3	Acyl-coenzyme A thioesterase 13;Acyl-coenzyme A thioesterase 13, N-terminally processed	ACOT13	6	6	6	By MS/MS	By MS/MS	By MS/MS	By MS/MS	By MS/MS	By MS/MS	By MS/MS	By MS/MS	By MS/MS	By MS/MS	By MS/MS	By MS/MS	46,4	14,96	0	78,28
O00154	O00154	Cytosolic acyl coenzyme A thioester hydrolase	ACOT7	7	7	7	By MS/MS	By MS/MS	By MS/MS	By MS/MS	By MS/MS	By MS/MS	By MS/MS	By MS/MS	By MS/MS	By MS/MS	By MS/MS	By MS/MS	25,8	41,80	0	17,09
Q9Y305	Q9Y305	Acyl-coenzyme A thioesterase 9, mitochondrial	ACOT9	4	4	4		By MS/MS		By MS/MS		By MS/MS	By MS/MS	By MS/MS	By MS/MS	By MS/MS			11,6	49,90	0	6,70
P24666	P24666	Low molecular weight phosphotyrosine protein phosphatase	ACP1	9	9	9	By MS/MS	By MS/MS	By MS/MS	By MS/MS	By MS/MS	By MS/MS	By MS/MS	By MS/MS	By MS/MS	By MS/MS	By MS/MS	By MS/MS	67,7	18,04	0	142,69
O95573	O95573	Long-chain-fatty-acid--CoA ligase 3	ACSL3	13	13	12	By MS/MS	By MS/MS	By MS/MS	By MS/MS	By MS/MS	By MS/MS	By MS/MS	By MS/MS	By MS/MS	By MS/MS	By MS/MS	By MS/MS	27,1	80,42	0	42,24
O60488	O60488	Long-chain-fatty-acid--CoA ligase 4	ACSL4	6	5	5	By MS/MS	By MS/MS	By MS/MS	By MS/MS	By MS/MS	By MS/MS	By matchin	By matchin	By MS/MS	By MS/MS	By MS/MS	By matchin	15,8	79,19	0	15,01
Q53FZ2	Q53FZ2	Acyl-coenzyme A synthetase ACSM3, mitochondrial	ACSM3	11	11	11	By MS/MS	By MS/MS	By MS/MS		By MS/MS	By MS/MS		By MS/MS			By MS/MS	By MS/MS	29,2	66,15	0	20,90
P68032;P63267;P68133;P62736	P68032;P63267;P68133;P62736	Actin, alpha cardiac muscle 1;Actin, gamma-enteric smooth muscle;Actin, alpha skeletal muscle;Actin, aortic smooth muscle	ACTC1;ACTG2;ACTA1;ACTA2	23	2	2	By MS/MS	By MS/MS	By MS/MS	By MS/MS	By MS/MS	By MS/MS	By MS/MS	By MS/MS	By MS/MS	By MS/MS	By matchin	By MS/MS	40,8	42,02	0	15,51
O96019	O96019	Actin-like protein 6A	ACTL6A	7	7	7	By MS/MS	By MS/MS	By MS/MS	By MS/MS	By MS/MS	By MS/MS	By MS/MS	By MS/MS			By MS/MS	By MS/MS	27,0	47,46	0	54,62
P12814	P12814	Alpha-actinin-1	ACTN1	32	12	10	By MS/MS	By MS/MS	By MS/MS	By MS/MS	By MS/MS	By MS/MS	By MS/MS	By MS/MS	By MS/MS	By MS/MS	By MS/MS	By MS/MS	42,7	103,06	0	143,51
O43707;Q9H254	O43707	Alpha-actinin-4	ACTN4	65	65	45	By MS/MS	By MS/MS	By MS/MS	By MS/MS	By MS/MS	By MS/MS	By MS/MS	By MS/MS	By MS/MS	By MS/MS	By MS/MS	By MS/MS	76,7	104,85	0	323,31
P61163;P42025	P61163;P42025	Alpha-centractin;Beta-centractin	ACTR1A;ACTR1B	9	9	9	By MS/MS	By MS/MS	By MS/MS	By MS/MS	By MS/MS	By MS/MS	By MS/MS				By MS/MS	By MS/MS	40,2	42,61	0	88,36
P61160	P61160	Actin-related protein 2	ACTR2	11	11	11	By MS/MS	By MS/MS	By MS/MS	By MS/MS	By MS/MS	By MS/MS	By MS/MS	By MS/MS	By MS/MS	By MS/MS			40,1	44,76	0	97,91
P61158;Q9C0K3;Q9P1U1	P61158	Actin-related protein 3	ACTR3	11	11	11	By MS/MS	By MS/MS	By MS/MS	By MS/MS	By MS/MS	By MS/MS	By MS/MS	By MS/MS			By MS/MS	By MS/MS	40,4	47,37	0	57,20
Q03154	Q03154	Aminoacylase-1	ACY1	6	6	6		By MS/MS	By MS/MS	By MS/MS	By MS/MS	By MS/MS				By MS/MS			24,8	45,88	0	36,72
P07311	P07311	Acylphosphatase-1	ACYP1	5	5	5	By MS/MS	By MS/MS	By MS/MS	By MS/MS	By MS/MS	By MS/MS	By MS/MS	By MS/MS	By MS/MS	By MS/MS	By MS/MS	By MS/MS	55,6	11,26	0	29,51
P35611	P35611	Alpha-adducin	ADD1	7	7	7	By MS/MS	By MS/MS	By MS/MS	By MS/MS	By MS/MS	By MS/MS					By MS/MS	By MS/MS	15,9	80,95	0	49,60
P11766	P11766	Alcohol dehydrogenase class-3	ADH5	10	10	10	By MS/MS	By MS/MS	By MS/MS	By MS/MS	By MS/MS	By MS/MS	By MS/MS	By MS/MS	By MS/MS	By MS/MS	By MS/MS		39,6	39,72	0	103,44
Q9BV57	Q9BV57	1,2-dihydroxy-3-keto-5-methylthiopentene dioxygenase	ADI1	9	9	9	By MS/MS	By MS/MS	By MS/MS	By MS/MS	By MS/MS	By MS/MS	By MS/MS		By MS/MS	By MS/MS	By MS/MS		60,3	21,50	0	31,20
P55263	P55263	Adenosine kinase	ADK	9	9	9	By MS/MS	By MS/MS	By MS/MS	By MS/MS	By MS/MS	By MS/MS	By MS/MS				By MS/MS	By MS/MS	43,6	40,55	0	111,68
P30566	P30566	Adenylosuccinate lyase	ADSL	10	10	10	By MS/MS	By MS/MS	By MS/MS	By MS/MS	By MS/MS	By MS/MS		By MS/MS			By MS/MS	By MS/MS	34,3	54,89	0	30,04
P30520	P30520	Adenylosuccinate synthetase isozyme 2	ADSS	11	11	11	By MS/MS	By MS/MS	By MS/MS	By MS/MS	By MS/MS	By MS/MS					By MS/MS	By MS/MS	33,8	50,10	0	180,55
Q9Y4W6	Q9Y4W6	AFG3-like protein 2	AFG3L2	12	12	11	By MS/MS	By MS/MS	By MS/MS	By MS/MS	By MS/MS	By MS/MS	By MS/MS	By MS/MS	By MS/MS	By MS/MS	By MS/MS	By MS/MS	23,3	88,58	0	221,02
Q53H12	Q53H12	Acylglycerol kinase, mitochondrial	AGK	4	4	4	By MS/MS	By MS/MS	By MS/MS	By MS/MS	By MS/MS	By MS/MS					By MS/MS	By MS/MS	15,6	47,14	0	10,35
P35573	P35573	Glycogen debranching enzyme;4-alpha-glucanotransferase;Amylo-alpha-1,6-glucosidase	AGL	8	8	8	By MS/MS	By MS/MS	By MS/MS	By MS/MS	By MS/MS	By MS/MS	By MS/MS	By MS/MS	By MS/MS	By MS/MS		By MS/MS	8,4	174,76	0	15,28
O00116	O00116	Alkyldihydroxyacetonephosphate synthase, peroxisomal	AGPS	14	14	14	By MS/MS	By MS/MS	By MS/MS	By MS/MS	By MS/MS	By MS/MS	By MS/MS	By MS/MS	By MS/MS	By MS/MS	By MS/MS	By MS/MS	28,9	72,91	0	145,80
P23526	P23526	Adenosylhomocysteinase	AHCY	19	19	19	By MS/MS	By MS/MS	By MS/MS	By MS/MS	By MS/MS	By MS/MS	By MS/MS	By MS/MS	By MS/MS	By MS/MS	By MS/MS	By MS/MS	42,4	47,72	0	198,20
O95433	O95433	Activator of 90 kDa heat shock protein ATPase homolog 1	AHSA1	7	7	7	By MS/MS	By MS/MS	By MS/MS	By MS/MS	By MS/MS	By MS/MS	By MS/MS	By MS/MS	By MS/MS	By MS/MS	By MS/MS	By MS/MS	22,8	38,27	0	19,20
O95831	O95831	Apoptosis-inducing factor 1, mitochondrial	AIFM1	14	14	14	By MS/MS	By MS/MS	By MS/MS	By MS/MS	By MS/MS	By MS/MS	By MS/MS	By MS/MS	By MS/MS	By MS/MS	By MS/MS	By MS/MS	35,1	66,90	0	138,42
Q12904	Q12904	Aminoacyl tRNA synthase complex-interacting multifunctional protein 1;Endothelial monocyte-activating polypeptide 2	AIMP1	11	11	11	By MS/MS	By MS/MS	By MS/MS	By MS/MS	By MS/MS	By MS/MS	By MS/MS	By MS/MS	By MS/MS	By MS/MS	By MS/MS	By MS/MS	58,3	34,35	0	57,02
Q13155	Q13155	Aminoacyl tRNA synthase complex-interacting multifunctional protein 2	AIMP2	9	9	9	By MS/MS	By MS/MS	By MS/MS	By MS/MS	By MS/MS	By MS/MS	By MS/MS	By MS/MS	By MS/MS	By MS/MS	By MS/MS	By MS/MS	46,6	35,35	0	105,75
O00170	O00170	AH receptor-interacting protein	AIP	5	5	5	By MS/MS	By MS/MS	By MS/MS	By MS/MS	By MS/MS	By MS/MS			By MS/MS		By MS/MS	By MS/MS	20,6	37,64	0	6,60
P00568;Q9Y6K8	P00568	Adenylate kinase isoenzyme 1	AK1	7	7	7	By MS/MS	By MS/MS	By MS/MS	By MS/MS	By MS/MS	By MS/MS	By MS/MS	By MS/MS	By MS/MS	By MS/MS	By MS/MS	By MS/MS	42,8	21,64	0	14,33
P54819	P54819	Adenylate kinase 2, mitochondrial;Adenylate kinase 2, mitochondrial, N-terminally processed	AK2	12	12	12	By MS/MS	By MS/MS	By MS/MS	By MS/MS	By MS/MS	By MS/MS	By MS/MS	By MS/MS	By MS/MS	By MS/MS	By MS/MS	By MS/MS	63,6	26,48	0	167,74
P27144	P27144	Adenylate kinase 4, mitochondrial	AK4	4	4	4	By MS/MS	By MS/MS	By MS/MS	By MS/MS	By MS/MS	By MS/MS			By MS/MS		By MS/MS	By MS/MS	34,1	25,27	0	9,31
P14550	P14550	Alcohol dehydrogenase [NADP(+)]	AKR1A1	7	7	7	By MS/MS	By MS/MS	By MS/MS	By MS/MS	By MS/MS	By MS/MS	By MS/MS	By MS/MS	By MS/MS	By MS/MS	By MS/MS		25,8	36,57	0	34,04

Q04828;P52895; P42330;P17516	Q04828;P52895	Aldo-keto reductase family 1 member C1;Aldo-keto reductase family 1 member C2	AKR1C1;AKR1C2	14	14	14	By MS/MS	By MS/MS	By MS/MS	By MS/MS	By MS/MS	By MS/MS	By MS/MS	By MS/MS	By MS/MS	By MS/MS	By MS/MS	By MS/MS	59,4	36,79	0	290,54
P54886	P54886	Delta-1-pyrroline-5-carboxylate synthase;Glutamate 5-kinase;Gamma-glutamyl phosphate reductase	ALDH18A1	13	13	13	By MS/MS	By MS/MS	By MS/MS	By MS/MS	By MS/MS	By MS/MS	By MS/MS	By MS/MS	By MS/MS	By MS/MS	By MS/MS	By MS/MS	23,3	87,30	0	323,31
P30837	P30837	Aldehyde dehydrogenase X, mitochondrial	ALDH1B1	11	11	11	By MS/MS	By MS/MS	By MS/MS	By MS/MS	By MS/MS	By MS/MS	By MS/MS	By MS/MS	By MS/MS		By MS/MS	By MS/MS	32,7	57,21	0	155,20
P05091	P05091	Aldehyde dehydrogenase, mitochondrial	ALDH2	8	8	8	By MS/MS	By MS/MS	By MS/MS		By MS/MS	By MS/MS					By MS/MS	By MS/MS	21,5	56,38	0	24,76
P49189	P49189	4-trimethylaminobutyraldehyde dehydrogenase	ALDH9A1	11	11	11	By MS/MS	By MS/MS		By MS/MS	By MS/MS	By MS/MS	By MS/MS	By MS/MS			By MS/MS	By MS/MS	28,3	53,80	0	35,90
P04075;P05062	P04075	Fructose-bisphosphate aldolase A	ALDOA	31	31	27	By MS/MS	By MS/MS	By MS/MS	By MS/MS	By MS/MS	By MS/MS	By MS/MS	By MS/MS	By MS/MS	By MS/MS	By MS/MS	By MS/MS	87,4	39,42	0	323,31
P09972	P09972	Fructose-bisphosphate aldolase C	ALDOC	14	10	10	By MS/MS	By MS/MS	By MS/MS	By MS/MS	By MS/MS	By MS/MS	By MS/MS	By MS/MS	By MS/MS	By MS/MS	By MS/MS	By MS/MS	47,5	39,46	0	323,31
Q86V81	Q86V81	THO complex subunit 4	ALYREF	5	5	5	By MS/MS	By MS/MS	By MS/MS	By MS/MS	By MS/MS	By MS/MS	By MS/MS	By MS/MS	By MS/MS	By MS/MS	By MS/MS	By MS/MS	26,1	26,89	0	280,40
Q9H1A4	Q9H1A4	Anaphase-promoting complex subunit 1	ANAPC1	6	6	6	By MS/MS	By MS/MS	By MS/MS	By MS/MS	By MS/MS	By MS/MS		By MS/MS			By MS/MS	By MS/MS	4,7	216,50	0	10,79
Q9UJX3	Q9UJX3	Anaphase-promoting complex subunit 7	ANAPC7	6	6	6	By MS/MS	By MS/MS	By MS/MS	By MS/MS	By MS/MS	By MS/MS						By MS/MS	12,4	66,86	0	104,40
P16157	P16157	Ankyrin-1	ANK1	30	30	30	By MS/MS	By MS/MS	By MS/MS	By MS/MS	By MS/MS	By MS/MS	By MS/MS	By MS/MS	By MS/MS		By MS/MS	By MS/MS	29,5	206,26	0	244,94
O15084;Q8N8A2	O15084	Serine/threonine-protein phosphatase 6 regulatory ankyrin repeat subunit A	ANKRD28	5	5	5	By MS/MS	By MS/MS		By MS/MS	By MS/MS	By MS/MS		By MS/MS			By MS/MS		8,0	112,96	0	5,89
P39687;O95626; O43423	P39687	Acidic leucine-rich nuclear phosphoprotein 32 family member A	ANP32A	12	12	9	By MS/MS	By MS/MS	By MS/MS	By MS/MS	By MS/MS	By MS/MS	By MS/MS	By MS/MS	By MS/MS	By MS/MS	By MS/MS	By MS/MS	41,0	28,59	0	323,31
Q92688	Q92688	Acidic leucine-rich nuclear phosphoprotein 32 family member B	ANP32B	11	8	8	By MS/MS	By MS/MS	By MS/MS	By MS/MS	By MS/MS	By MS/MS	By MS/MS	By MS/MS	By MS/MS	By MS/MS	By MS/MS	By MS/MS	37,1	28,79	0	56,40
Q9BTT0	Q9BTT0	Acidic leucine-rich nuclear phosphoprotein 32 family member E	ANP32E	6	6	6	By MS/MS	By MS/MS	By MS/MS	By MS/MS	By MS/MS	By MS/MS	By MS/MS	By MS/MS	By MS/MS	By MS/MS	By MS/MS	By MS/MS	23,1	30,69	0	154,24
P04083	P04083	Annexin A1	ANXA1	31	31	31	By MS/MS	By MS/MS	By MS/MS	By MS/MS	By MS/MS	By MS/MS	By MS/MS	By MS/MS	By MS/MS	By MS/MS	By MS/MS	By MS/MS	75,4	38,71	0	323,31
P50995	P50995	Annexin A11	ANXA11	15	15	15	By MS/MS	By MS/MS	By MS/MS	By MS/MS	By MS/MS	By MS/MS	By MS/MS	By MS/MS			By MS/MS	By MS/MS	33,1	54,39	0	179,81
P07355;A6NMY6	P07355;A6NMY6	Annexin A2;Putative annexin A2-like protein	ANXA2;ANXA2P2	27	27	27	By MS/MS	By MS/MS	By MS/MS	By MS/MS	By MS/MS	By MS/MS	By MS/MS	By MS/MS	By MS/MS	By MS/MS	By MS/MS	By MS/MS	66,4	38,60	0	323,31
P08758	P08758	Annexin A5	ANXA5	18	18	18	By MS/MS	By MS/MS	By MS/MS	By MS/MS	By MS/MS	By MS/MS	By MS/MS	By MS/MS	By MS/MS	By MS/MS	By MS/MS	By MS/MS	64,4	35,94	0	323,31
P20073	P20073	Annexin A7	ANXA7	14	14	14	By MS/MS	By MS/MS	By MS/MS	By MS/MS	By MS/MS	By MS/MS	By MS/MS	By MS/MS	By MS/MS	By MS/MS	By MS/MS	By MS/MS	30,1	52,74	0	266,37
Q10567	Q10567	AP-1 complex subunit beta-1	AP1B1	18	9	9	By MS/MS	By MS/MS	By MS/MS	By MS/MS	By MS/MS	By MS/MS	By MS/MS	By MS/MS	By MS/MS	By matchin	By MS/MS	By MS/MS	20,4	104,64	0	29,80
O43747	O43747	AP-1 complex subunit gamma-1	AP1G1	7	7	7	By MS/MS	By MS/MS	By MS/MS	By MS/MS	By MS/MS	By MS/MS	By MS/MS	By MS/MS	By MS/MS	By MS/MS	By MS/MS	By MS/MS	15,2	91,35	0	35,98
Q9BXS5	Q9BXS5	AP-1 complex subunit mu-1	AP1M1	5	5	5	By MS/MS	By MS/MS	By MS/MS	By MS/MS	By MS/MS	By MS/MS					By MS/MS	By MS/MS	20,1	48,59	0	9,34
O95782	O95782	AP-2 complex subunit alpha-1	AP2A1	15	15	11	By MS/MS	By MS/MS	By MS/MS	By MS/MS	By MS/MS	By MS/MS		By MS/MS	By MS/MS	By MS/MS	By MS/MS	By MS/MS	22,5	107,54	0	37,85
O94973	O94973	AP-2 complex subunit alpha-2	AP2A2	6	2	2	By matchin	By MS/MS	By matchin	By MS/MS	By matchin	By matching		By matchin	By matchin	By matching			10,6	103,96	0	17,31
P63010	P63010	AP-2 complex subunit beta	AP2B1	19	19	10	By MS/MS	By MS/MS	By MS/MS	By MS/MS	By MS/MS	By MS/MS	By MS/MS	By MS/MS	By MS/MS	By MS/MS	By MS/MS	By MS/MS	25,7	104,55	0	74,77
P13798	P13798	Acylamino-acid-releasing enzyme	APEH	8	8	8	By MS/MS	By MS/MS	By MS/MS	By MS/MS	By MS/MS	By MS/MS	By MS/MS	By MS/MS	By MS/MS	By MS/MS	By MS/MS	By MS/MS	19,3	81,22	0	44,00
P27695	P27695	DNA-(apurinic or apyrimidinic site) lyase;DNA-(apurinic or apyrimidinic site) lyase, mitochondrial	APEX1	10	10	10	By MS/MS	By MS/MS	By MS/MS	By MS/MS	By MS/MS	By MS/MS	By MS/MS	By MS/MS	By MS/MS	By MS/MS		By MS/MS	47,2	35,55	0	197,90
Q9BZZ5	Q9BZZ5	Apoptosis inhibitor 5	API5	12	12	12	By MS/MS	By MS/MS	By MS/MS	By MS/MS	By MS/MS	By MS/MS	By MS/MS	By MS/MS			By MS/MS	By MS/MS	29,4	59,00	0	276,03
Q9HDC9	Q9HDC9	Adipocyte plasma membrane-associated protein	APMAP	7	7	7	By MS/MS	By MS/MS	By MS/MS	By MS/MS	By MS/MS	By MS/MS		By MS/MS			By MS/MS	By MS/MS	24,0	46,48	0	15,19
Q8NCW5	Q8NCW5	NAD(P)H-hydrate epimerase	APOA1BP	4	4	4	By MS/MS	By MS/MS	By MS/MS	By MS/MS	By MS/MS	By MS/MS	By MS/MS	By MS/MS	By MS/MS	By MS/MS	By MS/MS	By MS/MS	22,6	31,67	0	45,68
Q9NRW3;Q8IUX4; Q96AK3	Q9NRW3	DNA dC->dU-editing enzyme APOBEC-3C	APOBEC3C	4	4	4	By MS/MS	By MS/MS		By MS/MS	By MS/MS	By MS/MS	By MS/MS	By MS/MS	By MS/MS	By MS/MS			29,5	22,83	0	24,07
P02649	P02649	Apolipoprotein E	APOE	4	4	4	By MS/MS		By MS/MS	By MS/MS	By MS/MS	By MS/MS	By MS/MS	By MS/MS	By MS/MS	By MS/MS	By MS/MS	By MS/MS	20,5	36,15	0	35,91
Q9BUR5	Q9BUR5	Apolipoprotein O	APOO	4	4	4	By MS/MS	By MS/MS	By MS/MS	By MS/MS	By MS/MS	By MS/MS	By MS/MS	By MS/MS	By MS/MS	By MS/MS	By MS/MS	By MS/MS	28,3	22,28	0	26,19
P07741	P07741	Adenine phosphoribosyltransferase	APRT	12	12	12	By MS/MS	By MS/MS	By MS/MS	By MS/MS	By MS/MS	By MS/MS	By MS/MS	By MS/MS	By MS/MS	By MS/MS	By MS/MS	By MS/MS	80,0	19,61	0	241,18
Q96P48	Q96P48	Arf-GAP with Rho-GAP domain, ANK repeat and PH domain-containing protein 1	ARAP1	5	5	5		By MS/MS	By MS/MS	By MS/MS	By MS/MS	By MS/MS		By MS/MS	By MS/MS	By MS/MS	By MS/MS	By MS/MS	5,2	162,19	0	8,66
P48444	P48444	Coatomer subunit delta	ARCN1	14	14	14	By MS/MS	By MS/MS	By MS/MS	By MS/MS	By MS/MS	By MS/MS		By MS/MS	By MS/MS	By MS/MS	By MS/MS		35,0	57,21	0	52,50
P84077;P61204	P84077;P61204	ADP-ribosylation factor 1;ADP-ribosylation factor 3	ARF1;ARF3	14	14	8	By MS/MS	By MS/MS	By MS/MS	By MS/MS	By MS/MS	By MS/MS	By MS/MS	By MS/MS	By MS/MS	By MS/MS	By MS/MS	By MS/MS	64,6	20,70	0	323,31
P18085	P18085	ADP-ribosylation factor 4	ARF4	11	6	6	By MS/MS	By MS/MS	By MS/MS	By MS/MS	By MS/MS	By MS/MS	By MS/MS	By MS/MS	By MS/MS	By MS/MS	By MS/MS	By MS/MS	65,6	20,51	0	17,02
P84085	P84085	ADP-ribosylation factor 5	ARF5	11	5	5	By MS/MS	By MS/MS	By MS/MS	By MS/MS	By MS/MS	By MS/MS	By MS/MS	By MS/MS	By MS/MS	By MS/MS	By matchin	By matchin	65,6	20,53	0	16,31
P62330	P62330	ADP-ribosylation factor 6	ARF6	7	7	7	By MS/MS	By MS/MS	By MS/MS	By MS/MS	By MS/MS	By MS/MS	By MS/MS	By MS/MS	By MS/MS			By MS/MS	54,3	20,08	0	21,02
Q07960	Q07960	Rho GTPase-activating protein 1	ARHGAP1	5	5	5	By MS/MS	By MS/MS	By MS/MS	By MS/MS	By MS/MS	By MS/MS					By MS/MS		23,0	50,44	0	33,73
P52565	P52565	Rho GDP-dissociation inhibitor 1	ARHGDIA	8	8	8	By MS/MS	By MS/MS	By MS/MS	By MS/MS	By MS/MS	By MS/MS	By MS/MS	By MS/MS	By MS/MS	By MS/MS	By MS/MS	By MS/MS	47,5	23,21	0	323,31
P52566	P52566	Rho GDP-dissociation inhibitor 2	ARHGDIB	5	5	5	By MS/MS	By MS/MS		By MS/MS	By MS/MS	By MS/MS			By MS/MS	By MS/MS	By MS/MS		44,3	22,99	0	16,25
P36404	P36404	ADP-ribosylation factor-like protein 2	ARL2	10	10	10	By MS/MS	By MS/MS	By MS/MS	By MS/MS	By MS/MS	By MS/MS	By MS/MS	By MS/MS	By MS/MS		By MS/MS	By MS/MS	50,0	20,88	0	37,62
P36405	P36405	ADP-ribosylation factor-like protein 3	ARL3	5	5	5	By MS/MS	By MS/MS			By MS/MS		By MS/MS	By MS/MS			By MS/MS	By MS/MS	39,0	20,46	0	7,57
Q96BM9	Q96BM9	ADP-ribosylation factor-like protein 8A	ARL8A	8	2	2	By MS/MS	By MS/MS	By matchin	By matchin	By MS/MS	By MS/MS	By matchin	By MS/MS	By MS/MS	By MS/MS	By MS/MS	By matchin	52,2	21,42	0	4,36
Q9NVJ2	Q9NVJ2	ADP-ribosylation factor-like protein 8B	ARL8B	11	11	5	By MS/MS	By MS/MS	By MS/MS	By MS/MS	By MS/MS	By MS/MS	By MS/MS	By MS/MS	By MS/MS	By MS/MS	By MS/MS	By MS/MS	59,7	21,54	0	53,83
Q9H993	Q9H993	Protein-glutamate O-methyltransferase	ARMT1	5	5	5	By MS/MS	By MS/MS	By MS/MS	By MS/MS	By MS/MS	By MS/MS					By MS/MS	By MS/MS	19,3	51,17	0	9,11
O15143;Q92747	O15143	Actin-related protein 2/3 complex subunit 1B	ARPC1B	5	5	5	By MS/MS	By MS/MS	By MS/MS	By MS/MS	By MS/MS	By MS/MS			By MS/MS	By MS/MS	By MS/MS		21,8	40,95	0	9,54
O15144	O15144	Actin-related protein 2/3 complex subunit 2	ARPC2	11	11	11	By MS/MS	By MS/MS	By MS/MS	By MS/MS	By MS/MS	By MS/MS			By MS/MS	By MS/MS		By MS/MS	42,0	34,33	0	17,47
O15145	O15145	Actin-related protein 2/3 complex subunit 3	ARPC3	6	6	6	By MS/MS	By MS/MS	By MS/MS	By MS/MS	By MS/MS	By MS/MS	By MS/MS	By MS/MS	By MS/MS	By MS/MS	By MS/MS	By MS/MS	32,6	20,55	0	31,76
P59998	P59998	Actin-related protein 2/3 complex subunit 4	ARPC4	9	9	9	By MS/MS	By MS/MS	By MS/MS	By MS/MS	By MS/MS	By MS/MS	By MS/MS	By MS/MS	By MS/MS	By MS/MS	By MS/MS	By MS/MS	60,1	19,67	0	61,07
Q13510	Q13510	Acid ceramidase;Acid ceramidase subunit alpha;Acid ceramidase subunit beta	ASAH1	9	9	9	By MS/MS	By MS/MS	By MS/MS		By MS/MS	By MS/MS	By MS/MS	By MS/MS	By MS/MS	By MS/MS	By MS/MS	By MS/MS	30,1	44,66	0	17,25
O43681	O43681	ATPase ASNA1	ASNA1	5	5	5	By MS/MS	By MS/MS		By MS/MS	By MS/MS	By MS/MS	By MS/MS	By MS/MS					16,4	38,79	0	11,57
P08243	P08243	Asparagine synthetase [glutamine-hydrolyzing]	ASNS	26	26	26	By MS/MS	By MS/MS	By MS/MS	By MS/MS	By MS/MS	By MS/MS	By MS/MS	By MS/MS			By MS/MS	By MS/MS	58,1	64,37	0	275,29
Q9NVI7;Q5T2N8	Q9NVI7	ATPase family AAA domain-containing protein 3A	ATAD3A	11	11	6	By MS/MS	By MS/MS	By MS/MS	By MS/MS	By MS/MS	By MS/MS	By MS/MS	By MS/MS			By MS/MS	By MS/MS	25,4	71,37	0	84,43
P31939	P31939	Bifunctional purine biosynthesis protein PURH;Phosphoribosylaminoimidazolecarboxamide formyltransferase;IMP cyclohydrolase	ATIC	39	39	39	By MS/MS	By MS/MS	By MS/MS	By MS/MS	By MS/MS	By MS/MS	By MS/MS	By MS/MS			By MS/MS	By MS/MS	73,5	64,62	0	323,31
Q6DD88	Q6DD88	Atlastin-3	ATL3	10	10	10	By MS/MS	By MS/MS	By MS/MS	By MS/MS	By MS/MS	By MS/MS					By MS/MS	By MS/MS	30,5	60,54	0	295,89
P05023;P13637; P50993;Q13733; P20648;P54707	P05023	Sodium/potassium-transporting ATPase subunit alpha-1	ATP1A1	22	22	22	By MS/MS	By MS/MS	By MS/MS	By MS/MS	By MS/MS	By MS/MS	By MS/MS	By MS/MS	By MS/MS	By MS/MS	By MS/MS	By MS/MS	28,1	112,89	0	323,31
P54709	P54709	Sodium/potassium-transporting ATPase subunit beta-3	ATP1B3	5	5	5	By MS/MS	By MS/MS	By MS/MS	By MS/MS	By MS/MS	By MS/MS	By MS/MS	By MS/MS	By MS/MS	By MS/MS			22,9	31,51	0	28,30
P16615;O14983	P16615	Sarcoplasmic/endoplasmic reticulum calcium ATPase 2	ATP2A2	18	18	15	By MS/MS	By MS/MS	By MS/MS	By MS/MS	By MS/MS	By MS/MS	By MS/MS	By MS/MS	By MS/MS	By MS/MS	By MS/MS	By MS/MS	23,3	114,76	0	173,36
P23634;P20020	P23634;P20020	Plasma membrane calcium-transporting ATPase 4;Plasma membrane calcium-transporting ATPase 1	ATP2B4;ATP2B1	6	6	3	By MS/MS	By MS/MS	By MS/MS	By MS/MS	By MS/MS	By MS/MS		By MS/MS	By MS/MS	By MS/MS			7,2	137,92	0	85,24

P25705	P25705	ATP synthase subunit alpha, mitochondrial	ATP5A1	33	33	33	By MS/MS	By MS/MS	By MS/MS	By MS/MS	By MS/MS	By MS/MS	By MS/MS	By MS/MS	By MS/MS	By MS/MS	By MS/MS	By MS/MS	66,0	59,75	0	323,31
P06576	P06576	ATP synthase subunit beta, mitochondrial	ATP5B	29	29	29	By MS/MS	By MS/MS	By MS/MS	By MS/MS	By MS/MS	By MS/MS	By MS/MS	By MS/MS	By MS/MS	By MS/MS	By MS/MS	By MS/MS	73,5	56,56	0	323,31
P36542	P36542	ATP synthase subunit gamma, mitochondrial	ATP5C1	8	8	8	By MS/MS	By MS/MS	By MS/MS	By MS/MS	By MS/MS	By MS/MS	By MS/MS	By MS/MS	By MS/MS	By MS/MS	By MS/MS	By MS/MS	28,5	33,00	0	88,05
P24539	P24539	ATP synthase subunit F(0) complex subunit B1, mitochondrial	ATP5F1	15	15	15	By MS/MS	By MS/MS	By MS/MS	By MS/MS	By MS/MS	By MS/MS	By MS/MS	By MS/MS	By MS/MS	By MS/MS	By MS/MS	By MS/MS	49,6	28,91	0	86,07
O75947	O75947	ATP synthase subunit d, mitochondrial	ATP5H	10	10	10	By MS/MS	By MS/MS	By MS/MS	By MS/MS	By MS/MS	By MS/MS	By MS/MS	By MS/MS	By MS/MS	By MS/MS	By MS/MS	By MS/MS	72,0	18,49	0	72,36
P56134	P56134	ATP synthase subunit f, mitochondrial	ATP5J2	6	6	6	By MS/MS	By MS/MS	By MS/MS	By MS/MS	By MS/MS	By MS/MS	By MS/MS	By MS/MS	By MS/MS	By MS/MS	By MS/MS	By MS/MS	52,1	10,92	0	52,21
O75964	O75964	ATP synthase subunit g, mitochondrial	ATP5L	7	7	7	By MS/MS	By MS/MS	By MS/MS	By MS/MS	By MS/MS	By MS/MS	By MS/MS	By MS/MS	By MS/MS	By MS/MS	By MS/MS	By MS/MS	59,2	11,43	0	35,56
P48047	P48047	ATP synthase subunit O, mitochondrial	ATP5O	13	13	13	By MS/MS	By MS/MS	By MS/MS	By MS/MS	By MS/MS	By MS/MS	By MS/MS	By MS/MS	By MS/MS	By MS/MS	By MS/MS	By MS/MS	67,6	23,28	0	223,90
P61421	P61421	V-type proton ATPase subunit d 1	ATP6V0D1	5	5	5					By MS/MS	By MS/MS			By MS/MS	By MS/MS			21,9	40,33	0	6,47
P38606	P38606	V-type proton ATPase catalytic subunit A	ATP6V1A	14	14	14	By MS/MS	By MS/MS	By MS/MS	By MS/MS	By MS/MS	By MS/MS	By MS/MS	By MS/MS	By MS/MS	By MS/MS	By MS/MS	By MS/MS	35,5	68,30	0	141,36
P21281;P15313	P21281	V-type proton ATPase subunit B, brain isoform	ATP6V1B2	14	14	14	By MS/MS	By MS/MS	By MS/MS	By MS/MS	By MS/MS	By MS/MS	By MS/MS	By MS/MS			By MS/MS	By MS/MS	44,2	56,50	0	127,74
P21283	P21283	V-type proton ATPase subunit C 1	ATP6V1C1	7	7	7	By MS/MS	By MS/MS	By MS/MS	By MS/MS	By MS/MS	By MS/MS	By MS/MS	By MS/MS	By MS/MS	By MS/MS			24,1	43,94	0	67,82
Q16864	Q16864	V-type proton ATPase subunit F	ATP6V1F	4	4	4	By MS/MS	By MS/MS			By MS/MS		By MS/MS	By MS/MS	By MS/MS	By MS/MS	By MS/MS	By MS/MS	42,9	13,37	0	15,19
O75348;O95670	O75348	V-type proton ATPase subunit G 1	ATP6V1G1	5	5	5	By MS/MS	By MS/MS	By MS/MS	By MS/MS	By MS/MS	By MS/MS	By MS/MS	By MS/MS	By MS/MS	By MS/MS	By MS/MS	By MS/MS	33,1	13,76	0	149,05
Q9UI12	Q9UI12	V-type proton ATPase subunit H	ATP6V1H	4	4	4	By MS/MS	By MS/MS	By MS/MS		By MS/MS	By MS/MS					By MS/MS	By MS/MS	12,4	55,88	0	15,31
Q9UBB4	Q9UBB4	Ataxin-10	ATXN10	10	10	10	By MS/MS	By MS/MS	By MS/MS	By MS/MS	By MS/MS	By MS/MS	By MS/MS	By MS/MS	By MS/MS	By MS/MS	By MS/MS	By MS/MS	32,8	53,49	0	323,31
Q8WWM7	Q8WWM7	Ataxin-2-like protein	ATXN2L	7	7	7	By MS/MS	By MS/MS	By MS/MS	By MS/MS	By MS/MS	By MS/MS	By MS/MS						10,3	113,37	0	21,26
O95816	O95816	BAG family molecular chaperone regulator 2	BAG2	10	10	10	By MS/MS	By MS/MS	By MS/MS	By MS/MS	By MS/MS	By MS/MS	By MS/MS	By MS/MS	By MS/MS	By MS/MS			55,9	23,77	0	88,73
P46379	P46379	Large proline-rich protein BAG6	BAG6	12	12	12	By MS/MS	By MS/MS	By MS/MS	By MS/MS	By MS/MS	By MS/MS	By MS/MS	By MS/MS	By MS/MS	By MS/MS	By MS/MS	By MS/MS	19,5	119,41	0	47,95
Q07812	Q07812	Apoptosis regulator BAX	BAX	7	7	7	By MS/MS	By MS/MS	By MS/MS	By MS/MS	By MS/MS	By MS/MS	By MS/MS	By MS/MS	By MS/MS	By MS/MS	By MS/MS	By MS/MS	43,2	21,18	0	134,08
P51572	P51572	B-cell receptor-associated protein 31	BCAP31	6	6	6	By MS/MS	By MS/MS	By MS/MS	By MS/MS	By MS/MS	By MS/MS	By MS/MS	By MS/MS	By MS/MS	By MS/MS	By MS/MS	By MS/MS	27,6	27,99	0	163,64
O75934	O75934	Pre-mRNA-splicing factor SPF27	BCAS2	6	6	6	By MS/MS	By MS/MS	By MS/MS	By MS/MS	By MS/MS	By MS/MS	By MS/MS	By MS/MS	By MS/MS	By MS/MS	By MS/MS	By MS/MS	44,4	26,13	0	76,31
P54687	P54687	Branched-chain-amino-acid aminotransferase, cytosolic	BCAT1	8	8	8	By MS/MS	By MS/MS	By MS/MS	By MS/MS	By MS/MS	By MS/MS	By MS/MS	By MS/MS	By MS/MS	By MS/MS			26,2	42,97	0	137,95
Q9P287	Q9P287	BRCA2 and CDKN1A-interacting protein	BCCIP	5	5	5	By MS/MS	By MS/MS	By MS/MS	By MS/MS	By MS/MS	By MS/MS	By MS/MS	By MS/MS	By MS/MS	By MS/MS	By MS/MS	By MS/MS	25,8	35,98	0	12,68
Q9BXK5	Q9BXK5	Bcl-2-like protein 13	BCL2L13	4	4	4	By MS/MS	By MS/MS	By MS/MS	By MS/MS	By MS/MS	By MS/MS	By MS/MS	By MS/MS	By MS/MS	By MS/MS			13,2	52,72	0	14,03
P55957	P55957	BH3-interacting domain death agonist;BH3-interacting domain death agonist p15;BH3-interacting domain death agonist p13;BH3-interacting domain death agonist p11	BID	10	10	10	By MS/MS	By MS/MS	By MS/MS	By MS/MS	By MS/MS	By MS/MS	By MS/MS	By MS/MS	By MS/MS	By MS/MS	By MS/MS	By MS/MS	61,0	21,99	0	161,22
Q9NR09	Q9NR09	Baculoviral IAP repeat-containing protein 6	BIRC6	5	5	5	By MS/MS		By MS/MS	By MS/MS	By MS/MS	By MS/MS	By MS/MS						1,8	530,25	0	18,99
Q13867	Q13867	Bleomycin hydrolase	BLMH	8	8	8	By MS/MS	By MS/MS	By MS/MS	By MS/MS	By MS/MS	By MS/MS					By MS/MS	By MS/MS	21,5	52,56	0	15,02
P53004	P53004	Biliverdin reductase A	BLVRA	13	13	13	By MS/MS	By MS/MS	By MS/MS	By MS/MS	By MS/MS	By MS/MS	By MS/MS	By MS/MS	By MS/MS	By MS/MS			51,7	33,43	0	59,81
P30043	P30043	Flavin reductase (NADPH)	BLVRB	17	17	17	By MS/MS	By MS/MS	By MS/MS	By MS/MS	By MS/MS	By MS/MS	By MS/MS	By MS/MS	By MS/MS	By MS/MS	By MS/MS	By MS/MS	74,3	22,12	0	323,31
Q9H3K6	Q9H3K6	BolA-like protein 2	BOLA2	6	6	6	By MS/MS	By MS/MS	By MS/MS	By MS/MS	By MS/MS	By MS/MS	By MS/MS	By MS/MS	By MS/MS	By MS/MS	By MS/MS	By MS/MS	74,4	10,12	0	19,46
O95861	O95861	3(2),5-bisphosphate nucleotidase 1	BPNT1	9	9	9	By MS/MS	By MS/MS	By MS/MS	By MS/MS	By MS/MS	By MS/MS	By MS/MS	By MS/MS	By MS/MS	By MS/MS			36,4	33,39	0	40,26
Q6PJG6	Q6PJG6	BRCA1-associated ATM activator 1	BRAT1	6	6	6	By MS/MS	By MS/MS	By MS/MS	By MS/MS	By MS/MS	By MS/MS				By MS/MS			13,0	88,12	0	29,66
Q8TDN6	Q8TDN6	Ribosome biogenesis protein BRX1 homolog	BRIX1	8	8	8	By MS/MS	By MS/MS	By MS/MS	By MS/MS	By MS/MS	By MS/MS	By MS/MS	By MS/MS	By MS/MS	By MS/MS			25,8	41,40	0	15,23
Q5VW32	Q5VW32	BRO1 domain-containing protein BROX	BROX	6	6	6	By MS/MS	By MS/MS	By MS/MS	By MS/MS	By MS/MS	By MS/MS	By MS/MS				By MS/MS	By MS/MS	23,8	46,48	0	63,67
P35613	P35613	Basigin	BSG	7	7	7	By MS/MS	By MS/MS	By MS/MS	By MS/MS	By MS/MS	By MS/MS	By MS/MS				By MS/MS	By MS/MS	23,4	42,20	0	229,71
P20290	P20290	Transcription factor BTF3	BTF3	9	9	9	By MS/MS	By MS/MS	By MS/MS	By MS/MS	By MS/MS	By MS/MS	By MS/MS	By MS/MS	By MS/MS	By MS/MS	By MS/MS	By MS/MS	63,1	22,17	0	147,84
O43684	O43684	Mitotic checkpoint protein BUB3	BUB3	11	11	11	By MS/MS	By MS/MS	By MS/MS	By MS/MS	By MS/MS	By MS/MS	By MS/MS	By MS/MS	By MS/MS	By MS/MS			44,8	37,15	0	97,24
P41223	P41223	Protein BUD31 homolog	BUD31	4	4	4	By MS/MS	By MS/MS	By MS/MS	By MS/MS	By MS/MS	By MS/MS	By MS/MS	By MS/MS			By MS/MS		33,3	17,00	0	5,33
Q13895	Q13895	Bystin	BYSL	8	8	8	By MS/MS	By MS/MS	By MS/MS	By MS/MS	By MS/MS	By MS/MS						By MS/MS	22,9	49,60	0	17,74
Q7L1Q6	Q7L1Q6	Basic leucine zipper and W2 domain-containing protein 1	BZW1	12	10	10	By MS/MS	By MS/MS	By MS/MS	By MS/MS	By MS/MS	By MS/MS		By MS/MS		By MS/MS	By MS/MS	By MS/MS	37,2	48,04	0	97,03
Q9Y6E2	Q9Y6E2	Basic leucine zipper and W2 domain-containing protein 2	BZW2	14	14	12	By MS/MS	By MS/MS	By MS/MS	By MS/MS	By MS/MS	By MS/MS	By MS/MS	By MS/MS	By MS/MS	By MS/MS	By MS/MS	By MS/MS	41,3	48,16	0	187,69
Q9H0W9	Q9H0W9	Ester hydrolase C11orf54	C11orf54	4	4	4	By MS/MS	By MS/MS	By MS/MS	By MS/MS	By MS/MS	By MS/MS			By MS/MS	By MS/MS	By MS/MS	By MS/MS	18,1	35,12	0	11,31
Q53FT3	Q53FT3	Protein Hikeshi	C11orf73	4	4	4	By MS/MS	By MS/MS	By MS/MS	By MS/MS	By MS/MS	By MS/MS	By MS/MS	By MS/MS	By MS/MS	By MS/MS	By MS/MS	By MS/MS	22,8	21,63	0	11,55
Q9Y224	Q9Y224	UPF0568 protein C14orf166	C14orf166	8	8	8	By MS/MS	By MS/MS	By MS/MS	By MS/MS	By MS/MS	By MS/MS	By MS/MS	By MS/MS	By MS/MS	By MS/MS	By MS/MS	By MS/MS	41,4	28,07	0	212,95
Q07021	Q07021	Complement component 1 Q subcomponent-binding protein, mitochondrial	C1QBP	10	10	10	By MS/MS	By MS/MS	By MS/MS	By MS/MS	By MS/MS	By MS/MS	By MS/MS	By MS/MS	By MS/MS	By MS/MS	By MS/MS	By MS/MS	43,3	31,36	0	323,31
P30042	P30042	ES1 protein homolog, mitochondrial	C21orf33	7	7	7	By MS/MS	By MS/MS	By MS/MS	By MS/MS	By MS/MS	By MS/MS		By MS/MS		By MS/MS	By MS/MS	By MS/MS	43,3	28,17	0	35,88
Q9BUH6	Q9BUH6	Protein PAXX	C9orf142	6	6	6		By MS/MS	By MS/MS	By MS/MS	By MS/MS	By MS/MS	By MS/MS	By MS/MS	By MS/MS			By MS/MS	41,7	21,64	0	13,36
Q5T6V5	Q5T6V5	UPF0553 protein C9orf64	C9orf64	5	5	5	By MS/MS	By MS/MS		By MS/MS		By MS/MS		By MS/MS	By MS/MS	By MS/MS		By MS/MS	19,9	39,03	0	12,30
P35219	P35219	Carbonic anhydrase-related protein	CA8	7	7	7	By MS/MS	By MS/MS	By MS/MS	By MS/MS	By MS/MS	By MS/MS	By MS/MS	By MS/MS	By MS/MS	By MS/MS			36,2	32,97	0	24,38
Q9Y376	Q9Y376	Calcium-binding protein 39	CAB39	12	12	11	By MS/MS	By MS/MS		By MS/MS	By MS/MS	By MS/MS	By MS/MS	By MS/MS	By MS/MS	By MS/MS			46,6	39,87	0	23,86
Q9HB71	Q9HB71	Calcyclin-binding protein	CACYBP	17	17	17	By MS/MS	By MS/MS	By MS/MS	By MS/MS	By MS/MS	By MS/MS	By MS/MS	By MS/MS	By MS/MS	By MS/MS	By MS/MS	By MS/MS	69,7	26,21	0	301,40
P27708	P27708	CAD protein;Glutamine-dependent carbamoyl-phosphate synthase;Aspartate carbamoyltransferase;Dihydroorotase	CAD	56	56	56	By MS/MS	By MS/MS	By MS/MS	By MS/MS	By MS/MS	By MS/MS	By MS/MS	By MS/MS	By MS/MS	By MS/MS	By MS/MS	By MS/MS	41,0	242,98	0	323,31
P05937;P22676	P05937	Calbindin	CALB1	5	5	5		By MS/MS	By MS/MS	By MS/MS	By MS/MS	By MS/MS			By MS/MS	By MS/MS		By MS/MS	24,9	30,03	0	33,86
P27797	P27797	Calreticulin	CALR	18	18	18	By MS/MS	By MS/MS	By MS/MS	By MS/MS	By MS/MS	By MS/MS	By MS/MS	By MS/MS			By MS/MS	By MS/MS	60,7	48,14	0	280,06
Q86VP6;O75155	Q86VP6	Cullin-associated NEDD8-dissociated protein 1	CAND1	33	33	33	By MS/MS	By MS/MS	By MS/MS	By MS/MS	By MS/MS	By MS/MS	By MS/MS	By MS/MS	By MS/MS	By MS/MS	By MS/MS	By MS/MS	37,3	136,37	0	297,49
P27824;O14967	P27824	Calnexin	CANX	26	26	26	By MS/MS	By MS/MS	By MS/MS	By MS/MS	By MS/MS	By MS/MS	By MS/MS	By MS/MS	By MS/MS	By MS/MS	By MS/MS	By MS/MS	47,6	67,57	0	216,56
Q01518;P40123	Q01518	Adenylyl cyclase-associated protein 1	CAP1	27	27	27	By MS/MS	By MS/MS	By MS/MS	By MS/MS	By MS/MS	By MS/MS	By MS/MS	By MS/MS	By MS/MS	By MS/MS	By MS/MS	By MS/MS	56,2	51,90	0	323,31
P40121	P40121	Macrophage-capping protein	CAPG	8	8	8	By MS/MS	By MS/MS	By MS/MS	By MS/MS	By MS/MS	By MS/MS		By MS/MS	By MS/MS	By MS/MS			31,0	38,50	0	75,50
P07384	P07384	Calpain-1 catalytic subunit	CAPN1	22	22	22	By MS/MS	By MS/MS	By MS/MS	By MS/MS	By MS/MS	By MS/MS	By MS/MS	By MS/MS	By MS/MS	By MS/MS	By MS/MS	By MS/MS	34,9	81,89	0	216,14
P17655	P17655	Calpain-2 catalytic subunit	CAPN2	8	8	8	By MS/MS	By MS/MS	By MS/MS	By MS/MS	By MS/MS	By MS/MS	By MS/MS	By MS/MS	By MS/MS	By MS/MS	By MS/MS	By MS/MS	18,9	79,99	0	47,46
P04632;Q96L46	P04632	Calpain small subunit 1	CAPNS1	15	15	15	By MS/MS	By MS/MS	By MS/MS	By MS/MS	By MS/MS	By MS/MS	By MS/MS	By MS/MS	By MS/MS	By MS/MS	By MS/MS	By MS/MS	70,9	28,32	0	320,60
Q14444	Q14444	Caprin-1	CAPRIN1	10	10	10	By MS/MS	By MS/MS	By MS/MS	By MS/MS	By MS/MS	By MS/MS	By MS/MS	By MS/MS	By MS/MS	By MS/MS	By MS/MS		20,3	78,37	0	111,70
P52907	P52907	F-actin-capping protein subunit alpha-1	CAPZA1	13	13	11	By MS/MS	By MS/MS	By MS/MS	By MS/MS	By MS/MS	By MS/MS	By MS/MS	By MS/MS	By MS/MS	By MS/MS	By MS/MS	By MS/MS	66,4	32,92	0	323,31
P47755	P47755	F-actin-capping protein subunit alpha-2	CAPZA2	7	5	5	By MS/MS	By MS/MS	By MS/MS	By MS/MS	By MS/MS	By MS/MS	By matchin	By matchin	By matchin	By matchin	By MS/MS		42,0	32,95	0	58,74
P47756	P47756	F-actin-capping protein subunit beta	CAPZB	15	15	15	By MS/MS	By MS/MS	By MS/MS	By MS/MS	By MS/MS	By MS/MS	By MS/MS	By MS/MS	By MS/MS	By MS/MS	By MS/MS	By MS/MS	55,6	31,35	0	98,06
Q86X55	Q86X55	Histone-arginine methyltransferase CARM1	CARM1	5	5	5	By MS/MS	By MS/MS	By MS/MS	By MS/MS	By MS/MS	By MS/MS	By MS/MS	By MS/MS	By MS/MS	By MS/MS	By MS/MS	By MS/MS	16,9	65,85	0	22,02
P49589	P49589	Cysteine--tRNA ligase, cytoplasmic	CARS	16	16	16	By MS/MS	By MS/MS	By MS/MS	By MS/MS	By MS/MS	By MS/MS	By MS/MS	By MS/MS	By MS/MS	By MS/MS	By MS/MS	By MS/MS	29,4	85,47	0	283,30
P04040	P04040	Catalase	CAT	23	23	23	By MS/MS	By MS/MS	By MS/MS	By MS/MS	By MS/MS	By MS/MS	By MS/MS	By MS/MS			By MS/MS	By MS/MS	54,3	59,76	0	76,30

Q13951	Q13951	Core-binding factor subunit beta	CBFB	8	8	8	By MS/MS	By MS/MS	By MS/MS	By MS/MS	By MS/MS	By MS/MS	By MS/MS	By MS/MS	By MS/MS	By MS/MS	By MS/MS	By MS/MS	39,0	21,51	0	190,52
P16152	P16152	Carbonyl reductase [NADPH] 1	CBR1	10	10	8	By MS/MS	By MS/MS	By MS/MS	By MS/MS	By MS/MS	By MS/MS	By MS/MS	By MS/MS	By MS/MS	By MS/MS	By MS/MS	By MS/MS	53,1	30,38	0	301,39
P0DN79;P35520	P0DN79;P35520	Cystathionine beta-synthase	CBS	16	16	16	By MS/MS	By MS/MS	By MS/MS	By MS/MS	By MS/MS	By MS/MS	By MS/MS	By MS/MS			By MS/MS	By MS/MS	47,9	60,59	0	98,83
Q13185	Q13185	Chromobox protein homolog 3	CBX3	8	8	6	By MS/MS	By MS/MS	By MS/MS	By MS/MS	By MS/MS	By MS/MS	By MS/MS	By MS/MS	By MS/MS	By MS/MS	By MS/MS	By MS/MS	42,6	20,81	0	244,80
Q8IX12	Q8IX12	Cell division cycle and apoptosis regulator protein 1	CCAR1	8	8	8	By MS/MS	By MS/MS	By MS/MS	By MS/MS	By MS/MS	By MS/MS			By MS/MS		By MS/MS		12,9	132,82	0	61,40
Q8N163	Q8N163	Cell cycle and apoptosis regulator protein 2	CCAR2	28	28	28	By MS/MS	By MS/MS	By MS/MS	By MS/MS	By MS/MS	By MS/MS	By MS/MS	By MS/MS	By MS/MS	By MS/MS	By MS/MS	By MS/MS	47,2	102,90	0	178,36
Q6YP21	Q6YP21	Kynurenine--oxoglutarate transaminase 3	CCBL2	4	4	4	By MS/MS	By MS/MS	By MS/MS	By MS/MS		By MS/MS		By MS/MS	By MS/MS	By MS/MS			14,3	51,40	0	16,23
Q96A33	Q96A33	Coiled-coil domain-containing protein 47	CCDC47	8	8	8	By MS/MS	By MS/MS	By MS/MS		By MS/MS	By MS/MS					By MS/MS	By MS/MS	22,2	55,87	0	23,10
Q4VC31	Q4VC31	Coiled-coil domain-containing protein 58	CCDC58	8	8	8	By MS/MS	By MS/MS		By MS/MS	By MS/MS	By MS/MS	By MS/MS	By MS/MS	By MS/MS	By MS/MS	By MS/MS	By MS/MS	63,9	16,62	0	41,34
P78371	P78371	T-complex protein 1 subunit beta	CCT2	39	39	39	By MS/MS	By MS/MS	By MS/MS	By MS/MS	By MS/MS	By MS/MS	By MS/MS	By MS/MS	By MS/MS	By MS/MS	By MS/MS	By MS/MS	75,7	57,49	0	323,31
P49368	P49368	T-complex protein 1 subunit gamma	CCT3	30	30	30	By MS/MS	By MS/MS	By MS/MS	By MS/MS	By MS/MS	By MS/MS	By MS/MS	By MS/MS	By MS/MS	By MS/MS	By MS/MS	By MS/MS	60,7	60,53	0	323,31
P50991	P50991	T-complex protein 1 subunit delta	CCT4	29	29	29	By MS/MS	By MS/MS	By MS/MS	By MS/MS	By MS/MS	By MS/MS	By MS/MS	By MS/MS	By MS/MS	By MS/MS	By MS/MS	By MS/MS	62,3	57,92	0	323,31
P48643	P48643	T-complex protein 1 subunit epsilon	CCT5	43	43	43	By MS/MS	By MS/MS	By MS/MS	By MS/MS	By MS/MS	By MS/MS	By MS/MS	By MS/MS	By MS/MS	By MS/MS	By MS/MS	By MS/MS	76,7	59,67	0	323,31
P40227	P40227	T-complex protein 1 subunit zeta	CCT6A	33	33	27	By MS/MS	By MS/MS	By MS/MS	By MS/MS	By MS/MS	By MS/MS	By MS/MS	By MS/MS	By MS/MS	By MS/MS	By MS/MS	By MS/MS	64,0	58,02	0	323,31
Q99832	Q99832	T-complex protein 1 subunit eta	CCT7	37	37	37	By MS/MS	By MS/MS	By MS/MS	By MS/MS	By MS/MS	By MS/MS	By MS/MS	By MS/MS			By MS/MS	By MS/MS	75,3	59,37	0	323,31
P50990	P50990	T-complex protein 1 subunit theta	CCT8	42	42	42	By MS/MS	By MS/MS	By MS/MS	By MS/MS	By MS/MS	By MS/MS	By MS/MS	By MS/MS	By MS/MS	By MS/MS	By MS/MS	By MS/MS	71,2	59,62	0	323,31
Q9Y5K6	Q9Y5K6	CD2-associated protein	CD2AP	8	8	8	By MS/MS	By MS/MS	By MS/MS	By MS/MS	By MS/MS	By MS/MS						By MS/MS	15,5	71,45	0	15,85
Q16543	Q16543	Hsp90 co-chaperone Cdc37;Hsp90 co-chaperone Cdc37, N-terminally processed	CDC37	9	9	9	By MS/MS	By MS/MS	By MS/MS	By MS/MS	By MS/MS	By MS/MS	By MS/MS	By MS/MS			By MS/MS	By MS/MS	29,6	44,47	0	107,90
P60953;P17081;Q9H4E5	P60953	Cell division control protein 42 homolog	CDC42	8	7	7	By MS/MS	By MS/MS	By MS/MS	By MS/MS	By MS/MS	By MS/MS	By MS/MS	By MS/MS	By MS/MS	By MS/MS	By MS/MS	By MS/MS	42,9	21,26	0	131,85
Q6DT37	Q6DT37	Serine/threonine-protein kinase MRCK gamma	CDC42BPG	4	4	4	By MS/MS	By MS/MS						By MS/MS	By MS/MS	By MS/MS	By MS/MS	By MS/MS	3,5	172,46	0	3,93
Q99459	Q99459	Cell division cycle 5-like protein	CDC5L	8	8	8	By MS/MS	By MS/MS		By MS/MS		By MS/MS	By MS/MS		By MS/MS	By MS/MS			14,2	92,25	0	85,60
Q6P1J9	Q6P1J9	Parafibromin	CDC73	7	7	7	By MS/MS	By MS/MS	By MS/MS		By MS/MS	By MS/MS	By MS/MS	By MS/MS	By MS/MS	By MS/MS	By MS/MS	By MS/MS	21,1	60,58	0	65,93
P06493;Q96Q40;O94921;Q07002;Q00536;Q00537;Q14004	P06493	Cyclin-dependent kinase 1	CDK1	22	22	20	By MS/MS	By MS/MS	By MS/MS	By MS/MS	By MS/MS	By MS/MS	By MS/MS	By MS/MS	By MS/MS	By MS/MS	By MS/MS	By MS/MS	80,8	34,10	0	323,31
P24941;Q00526	P24941	Cyclin-dependent kinase 2	CDK2	8	6	6	By MS/MS	By MS/MS	By MS/MS	By MS/MS	By MS/MS	By MS/MS	By MS/MS	By MS/MS	By MS/MS	By MS/MS		By MS/MS	38,9	33,93	0	19,52
Q00535	Q00535	Cyclin-dependent-like kinase 5	CDK5	4	3	3	By MS/MS	By MS/MS	By matchin	By MS/MS		By MS/MS	By matchin	By matchin	By MS/MS	By MS/MS		By MS/MS	15,8	33,30	0,001346	2,42
Q00534	Q00534	Cyclin-dependent kinase 6	CDK6	4	3	3	By MS/MS	By MS/MS	By MS/MS	By MS/MS	By MS/MS	By MS/MS	By matchin	By matchin	By matchin	By matching			15,3	36,94	0	9,71
Q9BXW7	Q9BXW7	Cat eye syndrome critical region protein 5	CECR5	7	7	7	By MS/MS	By MS/MS	By MS/MS	By MS/MS	By MS/MS	By MS/MS	By MS/MS	By MS/MS	By MS/MS	By MS/MS			28,6	46,32	0	13,15
Q9Y6A4	Q9Y6A4	Cilia- and flagella-associated protein 20	CFAP20	6	6	6	By MS/MS	By MS/MS	By MS/MS	By MS/MS	By MS/MS	By MS/MS		By MS/MS	By MS/MS	By MS/MS	By MS/MS	By MS/MS	26,9	22,77	0	15,28
P23528	P23528	Cofilin-1	CFL1	24	24	20	By MS/MS	By MS/MS	By MS/MS	By MS/MS	By MS/MS	By MS/MS	By MS/MS	By MS/MS	By MS/MS	By MS/MS	By MS/MS	By MS/MS	86,1	18,50	0	323,31
Q9Y281	Q9Y281	Cofilin-2	CFL2	6	2	2	By MS/MS	By matchin	By matchin	By matchin	By matchin	By MS/MS	By MS/MS	By MS/MS	By matchin	By matchin	By MS/MS	By matchin	38,0	18,74	0	5,63
Q9Y6H1;Q5T1J5	Q9Y6H1;Q5T1J5	Coiled-coil-helix-coiled-coil-helix domain-containing protein 2;Putative coiled-coil-helix-coiled-coil-helix domain-containing protein CHCHD2P9, mitochondrial	CHCHD2;CHCHD2P9	4	4	4	By MS/MS	By MS/MS	By MS/MS	By MS/MS	By MS/MS	By MS/MS						By MS/MS	33,8	15,51	0	128,10
Q9NX63	Q9NX63	MICOS complex subunit MIC19	CHCHD3	12	12	12	By MS/MS	By MS/MS	By MS/MS	By MS/MS	By MS/MS	By MS/MS	By MS/MS	By MS/MS	By MS/MS	By MS/MS	By MS/MS	By MS/MS	37,9	26,15	0	208,90
Q14839;Q8TDI0;Q12873	Q14839	Chromodomain-helicase-DNA-binding protein 4	CHD4	9	9	9	By MS/MS		By MS/MS	By MS/MS	By MS/MS	By MS/MS	By MS/MS	By MS/MS	By MS/MS	By MS/MS	By MS/MS	By MS/MS	6,9	218,00	0	24,00
Q9NZZ3	Q9NZZ3	Charged multivesicular body protein 5	CHMP5	4	4	4	By MS/MS	By MS/MS	By MS/MS	By MS/MS	By MS/MS	By MS/MS			By MS/MS	By MS/MS		By MS/MS	29,7	24,57	0	11,70
Q9UHD1	Q9UHD1	Cysteine and histidine-rich domain-containing protein 1	CHORDC1	9	9	9	By MS/MS	By MS/MS	By MS/MS	By MS/MS	By MS/MS	By MS/MS	By MS/MS	By MS/MS	By MS/MS	By MS/MS	By MS/MS	By MS/MS	42,5	37,49	0	114,32
Q99653	Q99653	Calcineurin B homologous protein 1	CHP1	9	9	9	By MS/MS	By MS/MS	By MS/MS	By MS/MS	By MS/MS	By MS/MS	By MS/MS	By MS/MS	By MS/MS	By MS/MS	By MS/MS	By MS/MS	48,7	22,46	0	63,47
Q6FI81	Q6FI81	Anamorsin	CIAPIN1	4	4	4	By MS/MS	By MS/MS	By MS/MS	By MS/MS	By MS/MS	By MS/MS		By MS/MS	By MS/MS			By MS/MS	20,5	33,58	0	21,56
Q14011	Q14011	Cold-inducible RNA-binding protein	CIRBP	8	8	8	By MS/MS	By MS/MS		By MS/MS		By MS/MS	By MS/MS	By MS/MS	By MS/MS	By MS/MS	By MS/MS	By MS/MS	45,9	18,65	0	111,37
Q9NZ45	Q9NZ45	CDGSH iron-sulfur domain-containing protein 1	CISD1	7	7	7	By MS/MS	By MS/MS	By MS/MS	By MS/MS	By MS/MS	By MS/MS	By MS/MS	By MS/MS	By MS/MS	By MS/MS	By MS/MS	By MS/MS	57,4	12,20	0	100,62
Q8N5K1	Q8N5K1	CDGSH iron-sulfur domain-containing protein 2	CISD2	7	7	7	By MS/MS	By MS/MS	By MS/MS	By MS/MS	By MS/MS	By MS/MS	By MS/MS	By MS/MS	By MS/MS	By MS/MS	By MS/MS	By MS/MS	42,2	15,28	0	134,72
Q14008	Q14008	Cytoskeleton-associated protein 5	CKAP5	23	23	23	By MS/MS	By MS/MS	By MS/MS	By MS/MS	By MS/MS	By MS/MS	By MS/MS	By MS/MS	By MS/MS	By MS/MS	By MS/MS	By MS/MS	16,5	225,49	0	37,02
P12277	P12277	Creatine kinase B-type	CKB	19	19	19	By MS/MS	By MS/MS	By MS/MS	By MS/MS	By MS/MS	By MS/MS	By MS/MS	By MS/MS	By MS/MS	By MS/MS	By MS/MS	By MS/MS	70,1	42,64	0	323,31
O75122	O75122	CLIP-associating protein 2	CLASP2	4	4	2	By MS/MS		By MS/MS	By MS/MS	By MS/MS	By MS/MS		By MS/MS	By MS/MS	By MS/MS		By MS/MS	4,2	141,13	0	15,05
O00299	O00299	Chloride intracellular channel protein 1	CLIC1	18	18	18	By MS/MS	By MS/MS	By MS/MS	By MS/MS	By MS/MS	By MS/MS	By MS/MS	By MS/MS	By MS/MS	By MS/MS	By MS/MS	By MS/MS	83,8	26,92	0	323,31
O15247	O15247	Chloride intracellular channel protein 2	CLIC2	7	6	6	By MS/MS	By MS/MS	By MS/MS	By MS/MS	By MS/MS	By MS/MS			By MS/MS	By MS/MS	By MS/MS	By MS/MS	43,3	28,36	0	65,21
Q9Y696	Q9Y696	Chloride intracellular channel protein 4	CLIC4	9	9	8	By MS/MS	By MS/MS	By MS/MS	By MS/MS	By MS/MS	By MS/MS			By MS/MS	By MS/MS	By MS/MS	By MS/MS	50,6	28,77	0	30,98
P54105	P54105	Methylosome subunit pICln	CLNS1A	5	5	5	By MS/MS	By MS/MS	By MS/MS	By MS/MS	By MS/MS	By MS/MS	By MS/MS	By MS/MS	By MS/MS	By MS/MS			43,9	26,22	0	119,01
Q16740	Q16740	ATP-dependent Clp protease proteolytic subunit, mitochondrial	CLPP	4	4	4	By MS/MS		By MS/MS		By MS/MS	By MS/MS						By MS/MS	23,1	30,18	0	10,34
O96005	O96005	Cleft lip and palate transmembrane protein 1	CLPTM1	4	4	4		By MS/MS			By MS/MS	By MS/MS			By MS/MS				10,8	76,10	0	8,65
Q00610	Q00610	Clathrin heavy chain 1	CLTC	86	86	69	By MS/MS	By MS/MS	By MS/MS	By MS/MS	By MS/MS	By MS/MS	By MS/MS	By MS/MS	By MS/MS	By MS/MS	By MS/MS	By MS/MS	63,3	191,61	0	323,31
P53675	P53675	Clathrin heavy chain 2	CLTCL1	19	2	2	By matchin	By MS/MS	By MS/MS	By MS/MS	By matchin	By matchin	By matchin	By matchin	By matchin	By MS/MS	By matchin	By matchin	11,7	187,03	0,005767	1,79
O75153;Q9Y5I4	O75153	Clustered mitochondria protein homolog	CLUH	15	15	15	By MS/MS	By MS/MS	By MS/MS	By MS/MS	By MS/MS	By MS/MS	By MS/MS	By MS/MS	By MS/MS	By MS/MS	By MS/MS	By MS/MS	14,5	146,67	0	45,97
Q96DG6	Q96DG6	Carboxymethylenebutenolidase homolog	CMBL	15	15	15	By MS/MS	By MS/MS	By MS/MS	By MS/MS	By MS/MS	By MS/MS	By MS/MS	By MS/MS	By MS/MS	By MS/MS	By MS/MS	By MS/MS	51,8	28,05	0	43,00
P30085	P30085	UMP-CMP kinase	CMPK1	15	15	15	By MS/MS	By MS/MS	By MS/MS	By MS/MS	By MS/MS	By MS/MS	By MS/MS	By MS/MS	By MS/MS	By MS/MS	By MS/MS	By MS/MS	64,8	22,22	0	323,31
P62633	P62633	Cellular nucleic acid-binding protein	CNBP	8	8	8	By MS/MS	By MS/MS	By MS/MS	By MS/MS	By MS/MS	By MS/MS	By MS/MS	By MS/MS	By MS/MS	By MS/MS	By MS/MS	By MS/MS	45,2	19,46	0	126,82
Q96KP4	Q96KP4	Cytosolic non-specific dipeptidase	CNDP2	14	14	14	By MS/MS	By MS/MS	By MS/MS	By MS/MS	By MS/MS	By MS/MS	By MS/MS	By MS/MS			By MS/MS	By MS/MS	40,6	52,88	0	43,67
A5YKK6	A5YKK6	CCR4-NOT transcription complex subunit 1	CNOT1	20	20	20	By MS/MS	By MS/MS	By MS/MS	By MS/MS	By MS/MS	By MS/MS				By MS/MS	By MS/MS	By MS/MS	12,8	266,94	0	81,95
Q9UKZ1	Q9UKZ1	CCR4-NOT transcription complex subunit 11	CNOT11	4	4	4		By MS/MS		By MS/MS	By MS/MS	By MS/MS				By MS/MS			12,2	55,22	0	15,41
Q9UIV1	Q9UIV1	CCR4-NOT transcription complex subunit 7	CNOT7	4	4	4	By MS/MS	By MS/MS	By MS/MS	By MS/MS	By MS/MS	By MS/MS	By MS/MS		By MS/MS		By MS/MS		18,9	32,75	0	4,84
P09543	P09543	2,3-cyclic-nucleotide 3-phosphodiesterase	CNP	4	4	4	By MS/MS	By MS/MS	By MS/MS	By MS/MS	By MS/MS	By MS/MS					By MS/MS		18,1	47,58	0	13,40
Q9Y2B0	Q9Y2B0	Protein canopy homolog 2	CNPY2	6	6	6	By MS/MS	By MS/MS	By MS/MS	By MS/MS	By MS/MS	By MS/MS	By MS/MS	By MS/MS	By MS/MS	By MS/MS	By MS/MS	By MS/MS	47,3	20,65	0	25,48
Q9Y2R0	Q9Y2R0	Cytochrome c oxidase assembly factor 3 homolog, mitochondrial	COA3	5	5	5	By MS/MS	By MS/MS	By MS/MS	By MS/MS	By MS/MS	By MS/MS			By MS/MS	By MS/MS			47,2	11,73	0	6,79
Q5JTJ3	Q5JTJ3	Cytochrome c oxidase assembly factor 6 homolog	COA6	6	6	6	By MS/MS	By MS/MS	By MS/MS	By MS/MS	By MS/MS	By MS/MS						By MS/MS	36,8	14,12	0	22,37
Q13057	Q13057	Bifunctional coenzyme A synthase;Phosphopantetheine adenylyltransferase;Dephospho-CoA kinase	COASY	5	5	5	By MS/MS	By MS/MS	By MS/MS	By MS/MS	By MS/MS	By MS/MS	By MS/MS	By MS/MS			By MS/MS	By MS/MS	14,5	62,33	0	14,39
Q8NBJ5	Q8NBJ5	Procollagen galactosyltransferase 1	COLGALT1	4	4	4	By MS/MS	By MS/MS	By MS/MS	By MS/MS	By MS/MS	By MS/MS	By MS/MS	By MS/MS	By MS/MS	By MS/MS			8,7	71,64	0	7,91
P21964	P21964	Catechol O-methyltransferase	COMT	15	15	15	By MS/MS	By MS/MS	By MS/MS	By MS/MS	By MS/MS	By MS/MS	By MS/MS	By MS/MS	By MS/MS	By MS/MS	By MS/MS	By MS/MS	67,5	30,04	0	148,01

P53621	P53621	Coatomer subunit alpha;Xenin;Proxenin	COPA	35	35	35	By MS/MS	By MS/MS	By MS/MS	By MS/MS	By MS/MS	By MS/MS	By MS/MS	By MS/MS	By MS/MS	By MS/MS	By MS/MS	By MS/MS	37,9	138,34	0	183,59
P53618	P53618	Coatomer subunit beta	COPB1	26	26	26	By MS/MS	By MS/MS	By MS/MS	By MS/MS	By MS/MS	By MS/MS	By MS/MS	By MS/MS	By MS/MS	By MS/MS	By MS/MS	By MS/MS	39,9	107,14	0	168,10
P35606	P35606	Coatomer subunit beta	COPB2	11	11	11	By MS/MS	By MS/MS	By MS/MS	By MS/MS	By MS/MS	By MS/MS	By MS/MS	By MS/MS	By MS/MS	By MS/MS	By MS/MS	By MS/MS	18,5	102,49	0	103,72
O14579	O14579	Coatomer subunit epsilon	COPE	11	11	11	By MS/MS	By MS/MS	By MS/MS	By MS/MS	By MS/MS	By MS/MS	By MS/MS	By MS/MS	By MS/MS	By MS/MS	By MS/MS	By MS/MS	45,1	34,48	0	197,62
Q9Y678	Q9Y678	Coatomer subunit gamma-1	COPG1	13	10	10	By MS/MS	By MS/MS	By MS/MS	By MS/MS	By MS/MS	By MS/MS	By matchin	By matchin	By MS/MS	By MS/MS	By MS/MS	By MS/MS	22,2	97,72	0	119,32
Q9UBF2	Q9UBF2	Coatomer subunit gamma-2	COPG2	23	23	20	By MS/MS	By MS/MS	By MS/MS	By MS/MS	By MS/MS	By MS/MS	By MS/MS	By MS/MS	By MS/MS	By MS/MS	By MS/MS	By MS/MS	39,6	97,62	0	294,65
Q9UNS2	Q9UNS2	COP9 signalosome complex subunit 3	COPS3	5	5	5	By MS/MS	By MS/MS	By MS/MS	By MS/MS	By MS/MS	By MS/MS	By MS/MS	By MS/MS	By MS/MS	By MS/MS			20,1	47,87	0	65,38
Q9BT78	Q9BT78	COP9 signalosome complex subunit 4	COPS4	11	11	11	By MS/MS	By MS/MS	By MS/MS	By MS/MS	By MS/MS	By MS/MS	By MS/MS	By MS/MS	By MS/MS	By MS/MS			43,1	46,27	0	119,92
Q92905	Q92905	COP9 signalosome complex subunit 5	COPS5	7	7	7	By MS/MS			By MS/MS	By MS/MS	By MS/MS	By MS/MS		By MS/MS		By MS/MS		27,8	37,58	0	20,17
Q7L5N1	Q7L5N1	COP9 signalosome complex subunit 6	COPS6	6	6	6	By MS/MS	By MS/MS	By MS/MS	By MS/MS	By MS/MS				By MS/MS	By MS/MS		By MS/MS	23,9	36,16	0	22,87
Q9H9Q2	Q9H9Q2	COP9 signalosome complex subunit 7b	COPS7B	4	4	4	By MS/MS	By MS/MS	By MS/MS	By MS/MS	By MS/MS	By MS/MS				By MS/MS		By MS/MS	23,9	29,62	0	30,04
Q99627	Q99627	COP9 signalosome complex subunit 8	COPS8	6	6	6	By MS/MS	By MS/MS	By MS/MS	By MS/MS	By MS/MS	By MS/MS	By MS/MS	By MS/MS	By MS/MS	By MS/MS	By MS/MS	By MS/MS	36,8	23,23	0	174,68
P61923	P61923	Coatomer subunit zeta-1	COPZ1	9	9	9	By MS/MS	By MS/MS	By MS/MS	By MS/MS	By MS/MS	By MS/MS	By MS/MS	By MS/MS	By MS/MS	By MS/MS	By MS/MS	By MS/MS	48,0	20,20	0	194,61
O75208	O75208	Ubiquinone biosynthesis protein COQ9, mitochondrial	COQ9	5	5	5	By MS/MS	By MS/MS	By MS/MS	By MS/MS	By MS/MS	By MS/MS			By MS/MS	By MS/MS	By MS/MS	By MS/MS	27,0	35,51	0	50,20
Q9BR76	Q9BR76	Coronin-1B	CORO1B	4	4	4		By MS/MS		By MS/MS		By MS/MS	By MS/MS			By MS/MS		By MS/MS	14,1	54,23	0	15,91
Q9ULV4	Q9ULV4	Coronin-1C	CORO1C	14	14	13	By MS/MS	By MS/MS	By MS/MS	By MS/MS	By MS/MS	By MS/MS	By MS/MS	By MS/MS	By MS/MS	By MS/MS	By MS/MS	By MS/MS	36,5	53,25	0	101,20
P57737	P57737	Coronin-7	CORO7	4	4	4	By MS/MS	By MS/MS	By MS/MS	By MS/MS	By MS/MS	By MS/MS					By MS/MS	By MS/MS	8,4	100,60	0	24,38
Q14019	Q14019	Coactosin-like protein	COTL1	9	9	9	By MS/MS	By MS/MS		By MS/MS			By MS/MS		By MS/MS			By MS/MS	67,6	15,95	0	14,40
Q5RI15	Q5RI15	Cytochrome c oxidase protein 20 homolog	COX20	4	4	4	By MS/MS	By MS/MS		By MS/MS	By MS/MS				By MS/MS	By MS/MS			33,1	13,29	0	10,90
P13073	P13073	Cytochrome c oxidase subunit 4 isoform 1, mitochondrial	COX4I1	11	11	11	By MS/MS	By MS/MS	By MS/MS	By MS/MS	By MS/MS	By MS/MS	By MS/MS	By MS/MS	By MS/MS	By MS/MS	By MS/MS	By MS/MS	51,5	19,58	0	32,48
P20674	P20674	Cytochrome c oxidase subunit 5A, mitochondrial	COX5A	4	4	4	By MS/MS	By MS/MS	By MS/MS	By MS/MS	By MS/MS	By MS/MS	By MS/MS	By MS/MS	By MS/MS	By MS/MS	By MS/MS	By MS/MS	21,3	16,76	0	107,27
P10606	P10606	Cytochrome c oxidase subunit 5B, mitochondrial	COX5B	6	6	6	By MS/MS	By MS/MS	By MS/MS	By MS/MS	By MS/MS	By MS/MS		By MS/MS	By MS/MS	By MS/MS	By MS/MS	By MS/MS	24,0	13,70	0	13,89
P14854	P14854	Cytochrome c oxidase subunit 6B1	COX6B1	4	4	4	By MS/MS	By MS/MS	By MS/MS	By MS/MS	By MS/MS	By MS/MS	By MS/MS		By MS/MS	By MS/MS			58,1	10,19	0	15,47
P09669	P09669	Cytochrome c oxidase subunit 6C	COX6C	5	5	5	By MS/MS	By MS/MS		By MS/MS	By MS/MS	By MS/MS	By MS/MS	By MS/MS	By MS/MS	By MS/MS	By MS/MS	By MS/MS	49,3	8,78	0	5,62
O14548	O14548	Cytochrome c oxidase subunit 7A-related protein, mitochondrial	COX7A2L	4	4	4	By MS/MS	By MS/MS	By MS/MS	By MS/MS	By MS/MS	By MS/MS	By MS/MS	By MS/MS	By MS/MS	By MS/MS	By MS/MS	By MS/MS	57,0	12,62	0	6,57
Q99829	Q99829	Copine-1	CPNE1	5	5	5	By MS/MS	By MS/MS	By MS/MS	By MS/MS	By MS/MS	By MS/MS						By MS/MS	18,4	59,06	0	50,50
O75131;Q96FN4;Q8IYJ1;Q96A23;O95741;Q86YQ8;Q9HCH3;Q9UBL6	O75131	Copine-3	CPNE3	12	12	12	By MS/MS	By MS/MS	By MS/MS	By MS/MS	By MS/MS	By MS/MS	By MS/MS	By MS/MS			By MS/MS	By MS/MS	25,7	60,13	0	41,07
P36551	P36551	Oxygen-dependent coproporphyrinogen-III oxidase, mitochondrial	CPOX	12	12	12	By MS/MS	By MS/MS	By MS/MS	By MS/MS	By MS/MS	By MS/MS	By MS/MS	By MS/MS	By MS/MS	By MS/MS	By MS/MS	By MS/MS	35,9	50,15	0	35,64
Q9BRF8	Q9BRF8	Serine/threonine-protein phosphatase CPPED1	CPPED1	5	5	5	By MS/MS	By MS/MS	By MS/MS	By MS/MS	By MS/MS	By MS/MS			By MS/MS	By MS/MS	By MS/MS	By MS/MS	25,8	35,55	0	50,23
Q10570	Q10570	Cleavage and polyadenylation specificity factor subunit 1	CPSF1	6	6	6		By MS/MS	By MS/MS	By MS/MS	By MS/MS	By MS/MS	By MS/MS	By MS/MS	By MS/MS	By MS/MS	By MS/MS	By MS/MS	5,9	160,88	0	118,36
Q16630	Q16630	Cleavage and polyadenylation specificity factor subunit 6	CPSF6	7	7	7	By MS/MS	By MS/MS	By MS/MS	By MS/MS	By MS/MS	By MS/MS		By MS/MS	By MS/MS	By MS/MS	By MS/MS	By MS/MS	18,0	59,21	0	134,03
Q8N684	Q8N684	Cleavage and polyadenylation specificity factor subunit 7	CPSF7	6	6	6	By MS/MS	By MS/MS	By MS/MS	By MS/MS	By MS/MS	By MS/MS	By MS/MS	By MS/MS	By MS/MS	By MS/MS	By MS/MS	By MS/MS	19,1	52,05	0	31,43
P46108	P46108	Adapter molecule crk	CRK	4	4	4	By MS/MS	By MS/MS	By MS/MS	By MS/MS	By MS/MS	By MS/MS	By MS/MS	By MS/MS	By MS/MS	By MS/MS	By MS/MS	By MS/MS	20,7	33,83	0	28,10
P46109	P46109	Crk-like protein	CRKL	16	16	16	By MS/MS	By MS/MS	By MS/MS	By MS/MS	By MS/MS	By MS/MS	By MS/MS	By MS/MS	By MS/MS	By MS/MS	By MS/MS	By MS/MS	55,4	33,78	0	256,29
Q08257	Q08257	Quinone oxidoreductase	CRYZ	13	13	13	By MS/MS	By MS/MS	By MS/MS	By MS/MS	By MS/MS	By MS/MS	By MS/MS	By MS/MS	By MS/MS	By MS/MS	By MS/MS	By MS/MS	55,3	35,21	0	31,92
O75390	O75390	Citrate synthase, mitochondrial	CS	16	16	16	By MS/MS	By MS/MS	By MS/MS	By MS/MS	By MS/MS	By MS/MS	By MS/MS	By MS/MS	By MS/MS	By MS/MS	By MS/MS	By MS/MS	43,8	51,71	0	139,85
O75534	O75534	Cold shock domain-containing protein E1	CSDE1	10	10	10	By MS/MS	By MS/MS	By MS/MS	By MS/MS	By MS/MS	By MS/MS	By MS/MS	By MS/MS	By MS/MS	By MS/MS	By MS/MS	By MS/MS	16,3	88,88	0	323,31
P55060	P55060	Exportin-2	CSE1L	40	40	40	By MS/MS	By MS/MS	By MS/MS	By MS/MS	By MS/MS	By MS/MS	By MS/MS	By MS/MS	By MS/MS	By MS/MS	By MS/MS	By MS/MS	56,1	110,42	0	323,31
P68400;Q8NEV1	P68400;Q8NEV1	Casein kinase II subunit alpha;Casein kinase II subunit alpha 3	CSNK2A1;CSNK2A3	12	12	12	By MS/MS	By MS/MS	By MS/MS	By MS/MS	By MS/MS	By MS/MS	By MS/MS	By MS/MS	By MS/MS	By MS/MS			37,1	45,14	0	42,96
P19784	P19784	Casein kinase II subunit alpha	CSNK2A2	9	9	9	By MS/MS	By MS/MS		By MS/MS	By MS/MS	By MS/MS	By MS/MS	By MS/MS	By MS/MS	By MS/MS			31,4	41,21	0	140,48
P67870	P67870	Casein kinase II subunit beta	CSNK2B	5	5	5	By MS/MS	By MS/MS	By MS/MS	By MS/MS	By MS/MS	By MS/MS	By MS/MS	By MS/MS	By MS/MS	By MS/MS	By MS/MS	By MS/MS	35,3	24,94	0	54,71
P04080	P04080	Cystatin-B	CSTB	5	5	5	By MS/MS	By MS/MS	By MS/MS	By MS/MS	By MS/MS	By MS/MS	By MS/MS	By MS/MS	By MS/MS	By MS/MS	By MS/MS	By MS/MS	79,6	11,14	0	152,75
Q12996	Q12996	Cleavage stimulation factor subunit 3	CSTF3	4	4	4	By MS/MS	By MS/MS	By MS/MS	By MS/MS		By MS/MS	By MS/MS	By MS/MS	By MS/MS	By MS/MS	By MS/MS	By MS/MS	7,9	82,92	0	81,02
Q13363;P56545	Q13363	C-terminal-binding protein 1	CTBP1	7	7	7		By MS/MS		By MS/MS	By MS/MS	By MS/MS	By MS/MS	By MS/MS	By MS/MS	By MS/MS			27,0	47,54	0	16,01
P32929	P32929	Cystathionine gamma-lyase	CTH	10	10	10	By MS/MS	By MS/MS	By MS/MS	By MS/MS	By MS/MS	By MS/MS	By MS/MS	By MS/MS	By MS/MS	By MS/MS			40,2	44,51	0	48,23
Q8WYA6	Q8WYA6	Beta-catenin-like protein 1	CTNNBL1	4	4	4			By MS/MS	By MS/MS	By MS/MS	By MS/MS			By MS/MS	By MS/MS	By MS/MS		7,8	65,17	0	5,48
P17812;Q9NRF8	P17812	CTP synthase 1	CTPS1	12	12	12	By MS/MS	By MS/MS	By MS/MS	By MS/MS	By MS/MS	By MS/MS			By MS/MS			By MS/MS	26,2	66,69	0	130,58
P53634	P53634	Dipeptidyl peptidase 1;Dipeptidyl peptidase 1 exclusion domain chain;Dipeptidyl peptidase 1 heavy chain;Dipeptidyl peptidase 1 light chain	CTSC	5	5	5	By MS/MS	By MS/MS	By MS/MS	By MS/MS	By MS/MS		By MS/MS	By MS/MS	By MS/MS	By MS/MS	By MS/MS	By MS/MS	18,8	51,85	0	28,67
P07339	P07339	Cathepsin D;Cathepsin D light chain;Cathepsin D heavy chain	CTSD	5	5	5	By MS/MS	By MS/MS	By MS/MS	By MS/MS	By MS/MS	By MS/MS	By MS/MS	By MS/MS	By MS/MS	By MS/MS	By MS/MS	By MS/MS	18,7	44,55	0	50,90
Q14247	Q14247	Src substrate cortactin	CTTN	5	5	5	By MS/MS	By MS/MS	By MS/MS	By MS/MS	By MS/MS				By MS/MS	By MS/MS		By MS/MS	13,5	61,59	0	10,64
Q7Z7A3	Q7Z7A3	Cytoplasmic tRNA 2-thiolation protein 1	CTU1	4	4	4	By MS/MS	By MS/MS	By MS/MS	By MS/MS					By MS/MS	By MS/MS		By MS/MS	14,4	36,45	0	5,20
Q13616	Q13616	Cullin-1	CUL1	9	9	9	By MS/MS	By MS/MS	By MS/MS	By MS/MS	By MS/MS	By MS/MS		By MS/MS	By MS/MS	By MS/MS	By MS/MS	By MS/MS	17,5	89,68	0	64,82
Q13620	Q13620	Cullin-4B	CUL4B	8	8	5	By MS/MS	By MS/MS		By MS/MS		By MS/MS	By MS/MS	By MS/MS					11,2	103,98	0	28,88
O60888	O60888	Protein CutA	CUTA	4	4	4	By MS/MS	By MS/MS	By MS/MS	By MS/MS	By MS/MS	By MS/MS	By MS/MS	By MS/MS	By MS/MS	By MS/MS	By MS/MS	By MS/MS	33,5	19,12	0	16,42
O43169	O43169	Cytochrome b5 type B	CYB5B	5	5	5	By MS/MS	By MS/MS	By MS/MS	By MS/MS	By MS/MS	By MS/MS	By MS/MS	By MS/MS	By MS/MS	By MS/MS	By MS/MS	By MS/MS	53,4	16,33	0	126,05
P00387	P00387	NADH-cytochrome b5 reductase 3;NADH-cytochrome b5 reductase 3 membrane-bound form;NADH-cytochrome b5 reductase 3 soluble form	CYB5R3	12	12	12	By MS/MS	By MS/MS	By MS/MS	By MS/MS	By MS/MS	By MS/MS	By MS/MS	By MS/MS	By MS/MS	By MS/MS	By MS/MS	By MS/MS	51,2	34,23	0	271,72
P08574	P08574	Cytochrome c1, heme protein, mitochondrial	CYC1	10	10	10	By MS/MS	By MS/MS	By MS/MS	By MS/MS	By MS/MS	By MS/MS	By MS/MS	By MS/MS	By MS/MS	By MS/MS	By MS/MS	By MS/MS	40,0	35,42	0	52,73
P99999	P99999	Cytochrome c	CYCS	13	13	13	By MS/MS	By MS/MS	By MS/MS	By MS/MS	By MS/MS	By MS/MS	By MS/MS	By MS/MS	By MS/MS	By MS/MS	By MS/MS	By MS/MS	63,8	11,75	0	246,53
Q7L576	Q7L576	Cytoplasmic FMR1-interacting protein 1	CYFIP1	15	15	8	By MS/MS	By MS/MS	By MS/MS	By MS/MS	By MS/MS	By MS/MS	By MS/MS	By MS/MS				By MS/MS	15,3	145,18	0	37,78
Q16850	Q16850	Lanosterol 14-alpha demethylase	CYP51A1	11	11	11	By MS/MS	By MS/MS	By MS/MS	By MS/MS	By MS/MS	By MS/MS	By MS/MS	By MS/MS			By MS/MS	By MS/MS	34,0	56,81	0	66,78
P61803	P61803	Dolichyl-diphosphooligosaccharide--protein glycosyltransferase subunit DAD1	DAD1	4	4	4	By MS/MS	By MS/MS	By MS/MS	By MS/MS	By MS/MS	By MS/MS	By MS/MS	By MS/MS	By MS/MS	By MS/MS	By MS/MS	By MS/MS	35,4	12,50	0	29,28
P51398	P51398	28S ribosomal protein S29, mitochondrial	DAP3	7	7	7	By MS/MS	By MS/MS	By MS/MS	By MS/MS	By MS/MS	By MS/MS	By MS/MS	By MS/MS	By MS/MS	By MS/MS			27,4	45,57	0	62,16

P14868	P14868	Aspartate--tRNA ligase, cytoplasmic	DARS	25	25	25	By MS/MS	By MS/MS	By MS/MS	By MS/MS	By MS/MS	By MS/MS	By MS/MS	By MS/MS	By MS/MS	By MS/MS	By MS/MS	By MS/MS	57,3	57,14	0	323,31
Q96EP5	Q96EP5	DAZ-associated protein 1	DAZAP1	5	5	5	By MS/MS	By MS/MS	By MS/MS	By MS/MS	By MS/MS	By MS/MS	By MS/MS	By MS/MS	By MS/MS	By MS/MS	By MS/MS	By MS/MS	20,4	43,38	0	42,97
P07108	P07108	Acyl-CoA-binding protein	DBI	5	5	5	By MS/MS	By MS/MS	By MS/MS	By MS/MS	By MS/MS	By MS/MS					By MS/MS	By MS/MS	69,0	10,04	0	15,86
Q8WVC6	Q8WVC6	Dephospho-CoA kinase domain-containing protein	DCAKD	8	8	8	By MS/MS	By MS/MS		By MS/MS	By MS/MS	By MS/MS	By MS/MS	By MS/MS			By MS/MS	By MS/MS	35,9	26,55	0	20,42
Q96C86	Q96C86	m7GpppX diphosphatase	DCPS	6	6	6	By MS/MS	By MS/MS	By MS/MS	By MS/MS	By MS/MS	By MS/MS	By MS/MS	By MS/MS	By MS/MS	By MS/MS			27,0	38,61	0	128,60
P32321	P32321	Deoxycytidylate deaminase	DCTD	5	5	5	By MS/MS	By MS/MS	By MS/MS	By MS/MS	By MS/MS	By MS/MS	By MS/MS	By MS/MS	By MS/MS	By MS/MS			29,2	20,02	0	14,44
Q14203	Q14203	Dynactin subunit 1	DCTN1	14	14	14	By MS/MS	By MS/MS	By MS/MS	By MS/MS	By MS/MS	By MS/MS	By MS/MS	By MS/MS		By MS/MS	By MS/MS		17,0	141,69	0	104,58
Q13561	Q13561	Dynactin subunit 2	DCTN2	6	6	6	By MS/MS	By MS/MS	By MS/MS	By MS/MS	By MS/MS	By MS/MS	By MS/MS				By MS/MS	By MS/MS	21,7	44,23	0	279,12
Q9H773	Q9H773	dCTP pyrophosphatase 1	DCTPP1	9	9	9	By MS/MS	By MS/MS	By MS/MS	By MS/MS	By MS/MS	By MS/MS	By MS/MS	By MS/MS	By MS/MS	By MS/MS	By MS/MS	By MS/MS	64,1	18,68	0	94,54
Q9BTE7	Q9BTE7	DCN1-like protein 5	DCUN1D5	5	5	5	By MS/MS	By MS/MS	By MS/MS	By MS/MS	By MS/MS	By MS/MS	By MS/MS	By MS/MS					19,0	27,51	0	7,55
Q7Z4W1	Q7Z4W1	L-xylulose reductase	DCXR	8	8	8	By MS/MS	By MS/MS	By MS/MS	By MS/MS	By MS/MS	By MS/MS	By MS/MS		By MS/MS	By MS/MS	By MS/MS	By MS/MS	42,2	25,91	0	21,30
Q16531	Q16531	DNA damage-binding protein 1	DDB1	27	27	27	By MS/MS	By MS/MS	By MS/MS	By MS/MS	By MS/MS	By MS/MS	By MS/MS	By MS/MS	By MS/MS	By MS/MS	By MS/MS	By MS/MS	32,4	126,97	0	101,35
P39656	P39656	Dolichyl-diphosphooligosaccharide--protein glycosyltransferase 48 kDa subunit	DDOST	13	13	13	By MS/MS	By MS/MS	By MS/MS	By MS/MS	By MS/MS	By MS/MS					By MS/MS	By MS/MS	48,9	50,80	0	70,71
Q96HY6	Q96HY6	DDRGK domain-containing protein 1	DDRGK1	5	5	5	By MS/MS	By MS/MS	By MS/MS	By MS/MS	By MS/MS	By MS/MS						By MS/MS	25,8	35,61	0	19,16
P30046;A6NHG4	P30046;A6NHG4	D-dopachrome decarboxylase;D-dopachrome decarboxylase-like protein	DDT;DDTL	9	9	9	By MS/MS	By MS/MS	By MS/MS	By MS/MS	By MS/MS	By MS/MS	By MS/MS	By MS/MS	By MS/MS	By MS/MS	By MS/MS	By MS/MS	56,8	12,71	0	295,34
Q92499	Q92499	ATP-dependent RNA helicase DDX1	DDX1	20	20	20	By MS/MS	By MS/MS	By MS/MS	By MS/MS	By MS/MS	By MS/MS	By MS/MS	By MS/MS	By MS/MS	By MS/MS	By MS/MS	By MS/MS	36,4	82,43	0	103,24
Q92841	Q92841	Probable ATP-dependent RNA helicase DDX17	DDX17	18	11	11	By MS/MS	By MS/MS	By MS/MS	By MS/MS	By MS/MS	By MS/MS	By MS/MS	By MS/MS	By MS/MS	By MS/MS	By MS/MS	By MS/MS	31,3	80,27	0	161,10
Q9NVP1	Q9NVP1	ATP-dependent RNA helicase DDX18	DDX18	12	12	12	By MS/MS	By MS/MS	By MS/MS	By MS/MS	By MS/MS	By MS/MS	By MS/MS	By MS/MS	By MS/MS	By MS/MS	By MS/MS	By MS/MS	22,2	75,41	0	23,89
Q9NUU7;Q9UHL0	Q9NUU7	ATP-dependent RNA helicase DDX19A	DDX19A	10	10	3	By MS/MS	By MS/MS	By MS/MS	By MS/MS	By MS/MS	By MS/MS	By MS/MS	By MS/MS			By MS/MS	By MS/MS	36,0	53,97	0	179,51
Q9NR30	Q9NR30	Nucleolar RNA helicase 2	DDX21	33	33	31	By MS/MS	By MS/MS	By MS/MS	By MS/MS	By MS/MS	By MS/MS	By MS/MS	By MS/MS	By MS/MS	By MS/MS	By MS/MS	By MS/MS	50,4	87,34	0	323,31
Q9BUQ8	Q9BUQ8	Probable ATP-dependent RNA helicase DDX23	DDX23	10	10	10	By MS/MS	By MS/MS		By MS/MS		By MS/MS	By MS/MS	By MS/MS	By MS/MS	By MS/MS	By MS/MS	By MS/MS	15,5	95,58	0	17,39
O00148	O00148	ATP-dependent RNA helicase DDX39A	DDX39A	16	4	4	By matchin	By matchin	By MS/MS	By matchin	By matchin	By MS/MS	By matchin	By MS/MS	By matchin	By MS/MS	By MS/MS	By MS/MS	47,5	49,13	0	36,40
Q13838	Q13838	Spliceosome RNA helicase DDX39B	DDX39B	21	21	9	By MS/MS	By MS/MS	By MS/MS	By MS/MS	By MS/MS	By MS/MS	By MS/MS	By MS/MS	By MS/MS	By MS/MS	By MS/MS	By MS/MS	62,9	48,99	0	88,26
O00571;Q9NQI0	O00571	ATP-dependent RNA helicase DDX3X	DDX3X	21	20	7	By MS/MS	By MS/MS	By MS/MS	By MS/MS	By MS/MS	By MS/MS	By MS/MS	By MS/MS	By MS/MS	By MS/MS	By MS/MS	By MS/MS	40,5	73,24	0	303,71
Q86XP3	Q86XP3	ATP-dependent RNA helicase DDX42	DDX42	5	5	5	By MS/MS	By MS/MS	By MS/MS	By MS/MS	By MS/MS	By MS/MS	By MS/MS			By MS/MS	By MS/MS	By MS/MS	9,8	102,97	0	9,73
Q7L014	Q7L014	Probable ATP-dependent RNA helicase DDX46	DDX46	11	11	11	By MS/MS	By MS/MS	By MS/MS	By MS/MS	By MS/MS	By MS/MS		By MS/MS			By MS/MS		11,3	117,36	0	34,36
Q9H0S4	Q9H0S4	Probable ATP-dependent RNA helicase DDX47	DDX47	4	4	4	By MS/MS	By MS/MS	By MS/MS	By MS/MS	By MS/MS	By MS/MS					By MS/MS	By MS/MS	14,1	50,65	0	80,65
P17844	P17844	Probable ATP-dependent RNA helicase DDX5	DDX5	26	26	19	By MS/MS	By MS/MS	By MS/MS	By MS/MS	By MS/MS	By MS/MS	By MS/MS	By MS/MS	By MS/MS	By MS/MS	By MS/MS	By MS/MS	48,0	69,15	0	230,59
Q9BQ39	Q9BQ39	ATP-dependent RNA helicase DDX50	DDX50	4	2	2	By MS/MS	By MS/MS	By MS/MS	By MS/MS	By matchin	By MS/MS	By matchin	By matchin	By matchin	By matchin	By matchin	By matchin	8,1	82,56	0	3,85
P26196	P26196	Probable ATP-dependent RNA helicase DDX6	DDX6	15	15	15	By MS/MS	By MS/MS	By MS/MS	By MS/MS	By MS/MS	By MS/MS	By MS/MS	By MS/MS			By MS/MS	By MS/MS	50,1	54,42	0	128,70
Q16698	Q16698	2,4-dienoyl-CoA reductase, mitochondrial	DECR1	9	9	9	By MS/MS	By MS/MS	By MS/MS	By MS/MS	By MS/MS	By MS/MS	By MS/MS	By MS/MS	By MS/MS	By MS/MS	By MS/MS	By MS/MS	41,8	36,07	0	57,03
P35659	P35659	Protein DEK	DEK	6	6	6		By MS/MS		By MS/MS		By MS/MS	By MS/MS	By MS/MS	By MS/MS	By MS/MS	By MS/MS	By MS/MS	21,6	42,67	0	13,09
O43583	O43583	Density-regulated protein	DENR	5	5	5	By MS/MS	By MS/MS	By MS/MS		By MS/MS	By MS/MS	By MS/MS	By MS/MS	By MS/MS	By MS/MS	By MS/MS	By MS/MS	34,3	22,09	0	15,10
P00374;Q86XF0	P00374	Dihydrofolate reductase	DHFR	8	8	8	By MS/MS	By MS/MS	By MS/MS	By MS/MS	By MS/MS	By MS/MS	By MS/MS	By MS/MS	By MS/MS	By MS/MS	By MS/MS	By MS/MS	55,1	21,45	0	52,99
Q6UWP2	Q6UWP2	Dehydrogenase/reductase SDR family member 11	DHRS11	7	7	7	By MS/MS	By MS/MS	By MS/MS	By MS/MS	By MS/MS	By MS/MS	By MS/MS	By MS/MS	By MS/MS		By MS/MS	By MS/MS	38,5	28,31	0	29,20
O43143	O43143	Pre-mRNA-splicing factor ATP-dependent RNA helicase DHX15	DHX15	20	20	20	By MS/MS	By MS/MS	By MS/MS	By MS/MS	By MS/MS	By MS/MS	By MS/MS	By MS/MS	By MS/MS	By MS/MS	By MS/MS	By MS/MS	32,8	90,93	0	238,65
Q7L2E3	Q7L2E3	Putative ATP-dependent RNA helicase DHX30	DHX30	22	22	22	By MS/MS	By MS/MS	By MS/MS	By MS/MS	By MS/MS	By MS/MS	By MS/MS		By MS/MS	By MS/MS	By MS/MS	By MS/MS	26,5	133,94	0	59,70
Q08211	Q08211	ATP-dependent RNA helicase A	DHX9	41	41	41	By MS/MS	By MS/MS	By MS/MS	By MS/MS	By MS/MS	By MS/MS	By MS/MS	By MS/MS	By MS/MS	By MS/MS	By MS/MS	By MS/MS	38,6	140,96	0	323,31
Q9NR28	Q9NR28	Diablo homolog, mitochondrial	DIABLO	10	10	10	By MS/MS	By MS/MS	By MS/MS	By MS/MS	By MS/MS	By MS/MS	By MS/MS	By MS/MS	By MS/MS	By MS/MS	By MS/MS	By MS/MS	51,0	27,13	0	92,73
O60610	O60610	Protein diaphanous homolog 1	DIAPH1	40	40	40	By MS/MS	By MS/MS	By MS/MS	By MS/MS	By MS/MS	By MS/MS	By MS/MS	By MS/MS	By MS/MS	By MS/MS	By MS/MS	By MS/MS	38,4	141,35	0	323,31
Q9UNQ2	Q9UNQ2	Probable dimethyladenosine transferase	DIMT1	6	6	6	By MS/MS	By MS/MS	By MS/MS	By MS/MS	By MS/MS	By MS/MS	By MS/MS	By MS/MS	By MS/MS	By MS/MS	By MS/MS	By MS/MS	23,3	35,24	0	14,36
P10515	P10515	Dihydrolipoyllysine-residue acetyltransferase component of pyruvate dehydrogenase complex, mitochondrial	DLAT	17	17	17	By MS/MS	By MS/MS	By MS/MS	By MS/MS	By MS/MS	By MS/MS	By MS/MS	By MS/MS	By MS/MS	By MS/MS	By MS/MS	By MS/MS	35,5	69,00	0	63,47
P09622	P09622	Dihydrolipoyl dehydrogenase, mitochondrial	DLD	15	15	15	By MS/MS	By MS/MS	By MS/MS	By MS/MS	By MS/MS	By MS/MS	By MS/MS	By MS/MS			By MS/MS	By MS/MS	40,7	54,18	0	323,31
P36957	P36957	Dihydrolipoyllysine-residue succinyltransferase component of 2-oxoglutarate dehydrogenase complex, mitochondrial	DLST	6	6	6	By MS/MS	By MS/MS			By MS/MS	By MS/MS	By MS/MS	By MS/MS	By MS/MS	By MS/MS	By MS/MS	By MS/MS	15,2	48,76	0	244,90
Q86Y56	Q86Y56	Dynein assembly factor 5, axonemal	DNAAF5	6	6	6	By MS/MS	By MS/MS	By MS/MS	By MS/MS	By MS/MS	By MS/MS	By MS/MS	By MS/MS	By MS/MS	By MS/MS	By MS/MS	By MS/MS	13,2	93,52	0	128,64
P31689	P31689	DnaJ homolog subfamily A member 1	DNAJA1	12	12	12	By MS/MS	By MS/MS	By MS/MS	By MS/MS	By MS/MS	By MS/MS	By MS/MS	By MS/MS		By MS/MS	By MS/MS	By MS/MS	43,8	44,87	0	60,33
O60884	O60884	DnaJ homolog subfamily A member 2	DNAJA2	7	7	7	By MS/MS	By MS/MS		By MS/MS	By MS/MS	By MS/MS	By MS/MS	By MS/MS	By MS/MS	By MS/MS	By MS/MS	By MS/MS	28,6	45,75	0	38,82
Q96EY1	Q96EY1	DnaJ homolog subfamily A member 3, mitochondrial	DNAJA3	5	5	5		By MS/MS				By MS/MS	By MS/MS	By MS/MS	By MS/MS	By MS/MS			13,3	52,49	0	24,88
P25685	P25685	DnaJ homolog subfamily B member 1	DNAJB1	5	5	4	By MS/MS	By MS/MS	By MS/MS	By MS/MS	By MS/MS	By MS/MS	By MS/MS	By MS/MS	By MS/MS	By MS/MS	By MS/MS	By MS/MS	19,1	38,04	0	16,92
Q9UBS4	Q9UBS4	DnaJ homolog subfamily B member 11	DNAJB11	4	4	4		By MS/MS		By MS/MS	By MS/MS	By MS/MS	By MS/MS	By MS/MS	By MS/MS	By MS/MS			13,4	40,51	0	9,58
Q9NVH1	Q9NVH1	DnaJ homolog subfamily C member 11	DNAJC11	4	4	4	By MS/MS	By MS/MS				By MS/MS			By MS/MS	By MS/MS			10,7	63,28	0	47,66
Q96DA6	Q96DA6	Mitochondrial import inner membrane translocase subunit TIM14	DNAJC19	8	8	8	By MS/MS	By MS/MS	By MS/MS	By MS/MS	By MS/MS	By MS/MS	By MS/MS	By MS/MS	By MS/MS	By MS/MS	By MS/MS	By MS/MS	71,6	12,50	0	58,47
Q99543	Q99543	DnaJ homolog subfamily C member 2;DnaJ homolog subfamily C member 2, N-terminally processed	DNAJC2	6	6	6	By MS/MS	By MS/MS	By MS/MS	By MS/MS	By MS/MS	By MS/MS	By MS/MS	By MS/MS	By MS/MS	By MS/MS	By MS/MS	By MS/MS	15,5	72,00	0	21,73
Q99615	Q99615	DnaJ homolog subfamily C member 7	DNAJC7	9	9	9	By MS/MS	By MS/MS	By MS/MS	By MS/MS	By MS/MS	By MS/MS	By MS/MS	By MS/MS			By MS/MS	By MS/MS	22,1	56,44	0	33,27
O75937	O75937	DnaJ homolog subfamily C member 8	DNAJC8	4	4	4	By MS/MS		By MS/MS	By MS/MS	By MS/MS	By MS/MS		By MS/MS	By MS/MS	By MS/MS		By MS/MS	34,0	29,84	0	20,31
Q8WXX5	Q8WXX5	DnaJ homolog subfamily C member 9	DNAJC9	11	11	11	By MS/MS	By MS/MS	By MS/MS	By MS/MS	By MS/MS	By MS/MS	By MS/MS		By MS/MS	By MS/MS	By MS/MS	By MS/MS	46,9	29,91	0	323,31
O00429	O00429	Dynamin-1-like protein	DNM1L	16	16	16	By MS/MS	By MS/MS	By MS/MS	By MS/MS	By MS/MS	By MS/MS	By MS/MS	By MS/MS		By MS/MS	By MS/MS	By MS/MS	37,5	81,88	0	218,75
P50570;Q05193;Q9UQ16	P50570	Dynamin-2	DNM2	11	11	11	By MS/MS	By MS/MS	By MS/MS	By MS/MS	By MS/MS	By MS/MS	By MS/MS	By MS/MS	By MS/MS	By MS/MS	By MS/MS	By MS/MS	17,5	98,06	0	30,35
P26358	P26358	DNA (cytosine-5)-methyltransferase 1	DNMT1	7	7	7		By MS/MS	By MS/MS	By MS/MS	By MS/MS	By MS/MS	By MS/MS	By MS/MS	By MS/MS	By MS/MS	By MS/MS	By MS/MS	6,3	183,16	0	12,74
O43598	O43598	2-deoxynucleoside 5-phosphate N-hydrolase 1	DNPH1	7	7	7	By MS/MS	By MS/MS	By MS/MS	By MS/MS	By MS/MS	By MS/MS	By MS/MS	By MS/MS	By MS/MS	By MS/MS	By MS/MS	By MS/MS	49,4	19,11	0	126,40
O60762	O60762	Dolichol-phosphate mannosyltransferase subunit 1	DPM1	9	9	9	By MS/MS	By MS/MS	By MS/MS	By MS/MS	By MS/MS	By MS/MS	By MS/MS	By MS/MS	By MS/MS	By MS/MS	By MS/MS	By MS/MS	44,2	29,63	0	63,23
Q9NY33	Q9NY33	Dipeptidyl peptidase 3	DPP3	11	11	11	By MS/MS	By MS/MS	By MS/MS	By MS/MS	By MS/MS	By MS/MS	By MS/MS	By MS/MS	By MS/MS	By MS/MS	By MS/MS	By MS/MS	26,6	82,59	0	323,31
Q9Y295	Q9Y295	Developmentally-regulated GTP-binding protein 1	DRG1	9	9	9	By MS/MS	By MS/MS	By MS/MS	By MS/MS	By MS/MS	By MS/MS	By MS/MS	By MS/MS	By MS/MS	By MS/MS			35,1	40,54	0	323,31
Q02413	Q02413	Desmoglein-1	DSG1	10	10	10	By MS/MS	By MS/MS		By MS/MS			By MS/MS	By MS/MS	By MS/MS	By MS/MS	By MS/MS	By MS/MS	16,5	113,75	0	157,80
P15924	P15924	Desmoplakin	DSP	19	19	19	By MS/MS	By MS/MS		By MS/MS		By MS/MS	By MS/MS	By MS/MS	By MS/MS	By MS/MS	By MS/MS	By MS/MS	11,0	331,77	0	149,56
P60981	P60981	Destrin	DSTN	8	8	8	By MS/MS	By MS/MS	By MS/MS	By MS/MS	By MS/MS	By MS/MS	By MS/MS	By MS/MS	By MS/MS	By MS/MS	By MS/MS	By MS/MS	55,8	18,51	0	19,04
P23919	P23919	Thymidylate kinase	DTYMK	8	8	8	By MS/MS	By MS/MS	By MS/MS	By MS/MS	By MS/MS	By MS/MS	By MS/MS		By MS/MS			By MS/MS	35,8	23,82	0	18,05

P51452	P51452	Dual specificity protein phosphatase 3	DUSP3	5	5	5	By MS/MS	By MS/MS	By MS/MS	By MS/MS		By MS/MS			By MS/MS			By MS/MS	38,9	20,48	0	16,16
P33316	P33316	Deoxyuridine 5-triphosphate nucleotidohydrolase, mitochondrial	DUT	10	10	10	By MS/MS	By MS/MS	By MS/MS	By MS/MS	By MS/MS	By MS/MS	By MS/MS	By MS/MS	By MS/MS	By MS/MS	By MS/MS	By MS/MS	54,4	26,56	0	188,78
Q14204	Q14204	Cytoplasmic dynein 1 heavy chain 1	DYNC1H1	117	117	117	By MS/MS	By MS/MS	By MS/MS	By MS/MS	By MS/MS	By MS/MS	By MS/MS	By MS/MS	By MS/MS	By MS/MS	By MS/MS	By MS/MS	35,6	532,40	0	323,31
Q13409	Q13409	Cytoplasmic dynein 1 intermediate chain 2	DYNC1I2	4	4	4	By MS/MS	By MS/MS		By MS/MS		By MS/MS	By MS/MS	By MS/MS	By MS/MS	By MS/MS	By MS/MS	By MS/MS	11,9	71,46	0	12,52
Q9Y6G9	Q9Y6G9	Cytoplasmic dynein 1 light intermediate chain 1	DYNC1LI1	11	11	11	By MS/MS	By MS/MS	By MS/MS	By MS/MS	By MS/MS	By MS/MS					By MS/MS	By MS/MS	31,5	56,58	0	95,75
P63167	P63167	Dynein light chain 1, cytoplasmic	DYNLL1	6	6	3	By MS/MS	By MS/MS	By MS/MS	By MS/MS	By MS/MS	By MS/MS	By MS/MS	By MS/MS	By MS/MS	By MS/MS	By MS/MS	By MS/MS	65,2	10,37	0	73,07
Q96FJ2	Q96FJ2	Dynein light chain 2, cytoplasmic	DYNLL2	6	3	3	By MS/MS	By MS/MS	By MS/MS	By MS/MS	By MS/MS	By MS/MS	By MS/MS	By MS/MS	By MS/MS	By matchin	By MS/MS	By matchin	65,2	10,35	0	17,14
Q9NP97;Q8TF09	Q9NP97;Q8TF09	Dynein light chain roadblock-type 1;Dynein light chain roadblock-type 2	DYNLRB1;DYNLRB2	5	5	5	By MS/MS	By MS/MS			By MS/MS		By MS/MS	By MS/MS	By MS/MS	By MS/MS		By MS/MS	64,6	10,92	0	33,03
P63172	P63172	Dynein light chain Tctex-type 1	DYNLT1	4	4	4	By MS/MS	By MS/MS	By MS/MS	By MS/MS	By MS/MS	By MS/MS	By MS/MS	By MS/MS	By MS/MS	By MS/MS	By MS/MS	By MS/MS	58,4	12,45	0	216,82
Q99848	Q99848	Probable rRNA-processing protein EBP2	EBNA1BP2	4	4	4				By MS/MS	By MS/MS			By MS/MS	By MS/MS				18,6	34,85	0	6,88
Q13011	Q13011	Delta(3,5)-Delta(2,4)-dienoyl-CoA isomerase, mitochondrial	ECH1	8	8	8	By MS/MS	By MS/MS	By MS/MS	By MS/MS	By MS/MS	By MS/MS	By MS/MS	By MS/MS	By MS/MS	By MS/MS	By MS/MS	By MS/MS	34,1	35,82	0	249,82
P30084	P30084	Enoyl-CoA hydratase, mitochondrial	ECHS1	7	7	7	By MS/MS	By MS/MS	By MS/MS	By MS/MS	By MS/MS	By MS/MS	By MS/MS	By MS/MS	By MS/MS	By MS/MS	By MS/MS	By MS/MS	33,1	31,39	0	130,68
P42126	P42126	Enoyl-CoA delta isomerase 1, mitochondrial	ECI1	8	8	8	By MS/MS	By MS/MS	By MS/MS	By MS/MS	By MS/MS	By MS/MS	By MS/MS	By MS/MS	By MS/MS	By MS/MS	By MS/MS	By MS/MS	30,8	32,82	0	42,15
O75521	O75521	Enoyl-CoA delta isomerase 2, mitochondrial	ECI2	6	6	6	By MS/MS	By MS/MS	By MS/MS	By MS/MS	By MS/MS	By MS/MS	By MS/MS	By MS/MS	By MS/MS	By MS/MS			26,4	43,59	0	124,90
Q5VYK3	Q5VYK3	Proteasome-associated protein ECM29 homolog	ECM29	38	38	38	By MS/MS	By MS/MS	By MS/MS	By MS/MS	By MS/MS	By MS/MS	By MS/MS	By MS/MS		By MS/MS	By MS/MS		31,6	204,29	0	143,93
Q6P2E9	Q6P2E9	Enhancer of mRNA-decapping protein 4	EDC4	9	9	9	By MS/MS			By MS/MS	By MS/MS	By MS/MS					By MS/MS	By MS/MS	10,8	151,66	0	30,52
O60869	O60869	Endothelial differentiation-related factor 1	EDF1	4	4	4	By MS/MS	By MS/MS	By MS/MS	By MS/MS	By MS/MS	By MS/MS				By MS/MS		By MS/MS	29,7	16,37	0	14,88
P68104;Q5VTE0;Q05639	P68104;Q5VTE0	Elongation factor 1-alpha 1;Putative elongation factor 1-alpha-like 3	EEF1A1;EEF1A1P5	28	28	28	By MS/MS	By MS/MS	By MS/MS	By MS/MS	By MS/MS	By MS/MS	By MS/MS	By MS/MS	By MS/MS	By MS/MS	By MS/MS	By MS/MS	72,3	50,14	0	323,31
P24534	P24534	Elongation factor 1-beta	EEF1B2	8	8	8	By MS/MS	By MS/MS	By MS/MS	By MS/MS	By MS/MS	By MS/MS	By MS/MS	By MS/MS	By MS/MS	By MS/MS	By MS/MS	By MS/MS	52,0	24,76	0	299,93
P29692	P29692	Elongation factor 1-delta	EEF1D	9	9	9	By MS/MS	By MS/MS	By MS/MS	By MS/MS	By MS/MS	By MS/MS	By MS/MS	By MS/MS	By MS/MS	By MS/MS	By MS/MS	By MS/MS	34,2	31,12	0	202,67
O43324	O43324	Eukaryotic translation elongation factor 1 epsilon-1	EEF1E1	11	11	11	By MS/MS	By MS/MS	By MS/MS	By MS/MS	By MS/MS	By MS/MS	By MS/MS	By MS/MS	By MS/MS	By MS/MS	By MS/MS	By MS/MS	64,4	19,81	0	24,16
P26641	P26641	Elongation factor 1-gamma	EEF1G	18	18	18	By MS/MS	By MS/MS	By MS/MS	By MS/MS	By MS/MS	By MS/MS	By MS/MS	By MS/MS	By MS/MS	By MS/MS	By MS/MS	By MS/MS	46,5	50,12	0	217,83
P13639	P13639	Elongation factor 2	EEF2	56	56	55	By MS/MS	By MS/MS	By MS/MS	By MS/MS	By MS/MS	By MS/MS	By MS/MS	By MS/MS	By MS/MS	By MS/MS	By MS/MS	By MS/MS	69,8	95,34	0	323,31
Q96C19;Q9BUP0	Q96C19	EF-hand domain-containing protein D2	EFHD2	6	6	6	By MS/MS	By MS/MS	By MS/MS	By MS/MS	By MS/MS	By MS/MS	By MS/MS		By MS/MS	By MS/MS		By MS/MS	31,2	26,70	0	24,45
Q15029	Q15029	116 kDa U5 small nuclear ribonucleoprotein component	EFTUD2	35	34	34	By MS/MS	By MS/MS	By MS/MS	By MS/MS	By MS/MS	By MS/MS	By MS/MS	By MS/MS	By MS/MS	By MS/MS	By MS/MS	By MS/MS	51,9	109,43	0	323,31
Q9H4M9;Q9NZN3	Q9H4M9	EH domain-containing protein 1	EHD1	18	18	18	By MS/MS	By MS/MS	By MS/MS	By MS/MS	By MS/MS	By MS/MS					By MS/MS	By MS/MS	44,4	60,63	0	44,96
P41567	P41567	Eukaryotic translation initiation factor 1	EIF1	8	8	2	By MS/MS	By MS/MS	By MS/MS	By MS/MS	By MS/MS	By MS/MS	By MS/MS	By MS/MS	By MS/MS	By MS/MS	By MS/MS	By MS/MS	77,0	12,73	0	233,85
P47813;O14602	P47813;O14602	Eukaryotic translation initiation factor 1A, X-chromosomal;Eukaryotic translation initiation factor 1A, Y-chromosomal	EIF1AX;EIF1AY	9	9	9	By MS/MS	By MS/MS	By MS/MS	By MS/MS	By MS/MS	By MS/MS	By MS/MS	By MS/MS	By MS/MS	By MS/MS	By MS/MS	By MS/MS	71,5	16,46	0	90,62
Q9BY44	Q9BY44	Eukaryotic translation initiation factor 2A;Eukaryotic translation initiation factor 2A, N-terminally processed	EIF2A	7	7	7		By MS/MS	By MS/MS	By MS/MS	By MS/MS	By MS/MS					By MS/MS		19,5	64,99	0	8,04
Q14232	Q14232	Translation initiation factor eIF-2B subunit alpha	EIF2B1	13	13	13	By MS/MS	By MS/MS	By MS/MS	By MS/MS	By MS/MS	By MS/MS	By MS/MS	By MS/MS		By MS/MS	By MS/MS	By MS/MS	60,0	33,71	0	243,50
Q9UI10	Q9UI10	Translation initiation factor eIF-2B subunit delta	EIF2B4	7	7	7	By MS/MS	By MS/MS	By MS/MS	By MS/MS	By MS/MS	By MS/MS	By MS/MS	By MS/MS	By MS/MS	By MS/MS	By MS/MS	By MS/MS	20,1	57,56	0	26,34
Q13144	Q13144	Translation initiation factor eIF-2B subunit epsilon	EIF2B5	5	5	5	By MS/MS	By MS/MS	By MS/MS	By MS/MS	By MS/MS	By MS/MS	By MS/MS	By MS/MS	By MS/MS	By MS/MS	By MS/MS	By MS/MS	11,2	80,38	0	21,82
P05198	P05198	Eukaryotic translation initiation factor 2 subunit 1	EIF2S1	20	20	20	By MS/MS	By MS/MS	By MS/MS	By MS/MS	By MS/MS	By MS/MS	By MS/MS	By MS/MS	By MS/MS	By MS/MS	By MS/MS	By MS/MS	60,6	36,11	0	323,31
P20042	P20042	Eukaryotic translation initiation factor 2 subunit 2	EIF2S2	9	9	9		By MS/MS	By MS/MS	By MS/MS	By MS/MS	By MS/MS	By MS/MS	By MS/MS	By MS/MS	By MS/MS	By MS/MS	By MS/MS	36,3	38,39	0	116,16
P41091;Q2VIR3	P41091;Q2VIR3	Eukaryotic translation initiation factor 2 subunit 3;Putative eukaryotic translation initiation factor 2 subunit 3-like protein	EIF2S3;EIF2S3L	14	14	14	By MS/MS	By MS/MS	By MS/MS	By MS/MS	By MS/MS	By MS/MS	By MS/MS	By MS/MS			By MS/MS	By MS/MS	43,2	51,11	0	53,93
Q14152	Q14152	Eukaryotic translation initiation factor 3 subunit A	EIF3A	30	30	30	By MS/MS	By MS/MS	By MS/MS	By MS/MS	By MS/MS	By MS/MS	By MS/MS	By MS/MS	By MS/MS	By MS/MS	By MS/MS	By MS/MS	25,4	166,57	0	190,17
P55884	P55884	Eukaryotic translation initiation factor 3 subunit B	EIF3B	28	28	28	By MS/MS	By MS/MS	By MS/MS	By MS/MS	By MS/MS	By MS/MS	By MS/MS	By MS/MS	By MS/MS	By MS/MS	By MS/MS	By MS/MS	47,4	92,48	0	192,13
Q99613;B5ME19	Q99613;B5ME19	Eukaryotic translation initiation factor 3 subunit C;Eukaryotic translation initiation factor 3 subunit C-like protein	EIF3C;EIF3CL	13	13	13	By MS/MS	By MS/MS	By MS/MS	By MS/MS	By MS/MS	By MS/MS	By MS/MS	By MS/MS	By MS/MS	By MS/MS	By MS/MS	By MS/MS	17,2	105,34	0	300,60
O15371	O15371	Eukaryotic translation initiation factor 3 subunit D	EIF3D	12	12	12	By MS/MS	By MS/MS	By MS/MS	By MS/MS	By MS/MS	By MS/MS	By MS/MS				By MS/MS	By MS/MS	29,7	63,97	0	291,41
P60228	P60228	Eukaryotic translation initiation factor 3 subunit E	EIF3E	17	17	17	By MS/MS	By MS/MS	By MS/MS	By MS/MS	By MS/MS	By MS/MS	By MS/MS	By MS/MS			By MS/MS	By MS/MS	43,1	52,22	0	153,28
O00303	O00303	Eukaryotic translation initiation factor 3 subunit F	EIF3F	9	9	9	By MS/MS	By MS/MS	By MS/MS	By MS/MS	By MS/MS	By MS/MS	By MS/MS	By MS/MS	By MS/MS	By MS/MS	By MS/MS	By MS/MS	40,9	37,56	0	128,29
O75821	O75821	Eukaryotic translation initiation factor 3 subunit G	EIF3G	10	10	10	By MS/MS	By MS/MS	By MS/MS	By MS/MS	By MS/MS	By MS/MS	By MS/MS	By MS/MS	By MS/MS	By MS/MS	By MS/MS	By MS/MS	34,4	35,61	0	124,37
O15372	O15372	Eukaryotic translation initiation factor 3 subunit H	EIF3H	8	8	8	By MS/MS	By MS/MS	By MS/MS	By MS/MS	By MS/MS	By MS/MS	By MS/MS	By MS/MS	By MS/MS	By MS/MS			30,4	39,93	0	32,15
Q13347	Q13347	Eukaryotic translation initiation factor 3 subunit I	EIF3I	12	12	12	By MS/MS	By MS/MS	By MS/MS	By MS/MS	By MS/MS	By MS/MS	By MS/MS	By MS/MS	By MS/MS	By MS/MS	By MS/MS	By MS/MS	57,8	36,50	0	80,41
O75822	O75822	Eukaryotic translation initiation factor 3 subunit J	EIF3J	9	9	9	By MS/MS	By MS/MS	By MS/MS		By MS/MS	By MS/MS	By MS/MS	By MS/MS	By MS/MS	By MS/MS	By MS/MS	By MS/MS	39,9	29,06	0	125,90
Q9UBQ5	Q9UBQ5	Eukaryotic translation initiation factor 3 subunit K	EIF3K	10	10	10	By MS/MS	By MS/MS	By MS/MS	By MS/MS	By MS/MS	By MS/MS	By MS/MS	By MS/MS	By MS/MS	By MS/MS	By MS/MS	By MS/MS	53,2	25,06	0	183,81
Q9Y262	Q9Y262	Eukaryotic translation initiation factor 3 subunit L	EIF3L	16	16	16	By MS/MS	By MS/MS	By MS/MS	By MS/MS	By MS/MS	By MS/MS	By MS/MS	By MS/MS			By MS/MS	By MS/MS	37,6	66,73	0	323,31
Q7L2H7	Q7L2H7	Eukaryotic translation initiation factor 3 subunit M	EIF3M	17	17	17	By MS/MS	By MS/MS	By MS/MS	By MS/MS	By MS/MS	By MS/MS	By MS/MS	By MS/MS	By MS/MS	By MS/MS	By MS/MS	By MS/MS	58,3	42,50	0	89,41
P60842	P60842	Eukaryotic initiation factor 4A-I	EIF4A1	26	26	15	By MS/MS	By MS/MS	By MS/MS	By MS/MS	By MS/MS	By MS/MS	By MS/MS	By MS/MS	By MS/MS	By MS/MS	By MS/MS	By MS/MS	67,7	46,15	0	323,31
Q14240	Q14240	Eukaryotic initiation factor 4A-II;Eukaryotic initiation factor 4A-II, N-terminally processed	EIF4A2	18	7	7	By MS/MS	By MS/MS	By MS/MS	By MS/MS	By MS/MS	By MS/MS	By MS/MS	By MS/MS	By matchin	By matchin	By MS/MS	By MS/MS	51,4	46,40	0	23,94
P38919	P38919	Eukaryotic initiation factor 4A-III;Eukaryotic initiation factor 4A-III, N-terminally processed	EIF4A3	12	10	10	By MS/MS	By MS/MS	By MS/MS	By MS/MS	By MS/MS	By MS/MS	By MS/MS	By MS/MS			By MS/MS	By MS/MS	37,7	46,87	0	70,12
P23588	P23588	Eukaryotic translation initiation factor 4B	EIF4B	5	5	5	By MS/MS	By MS/MS	By MS/MS	By MS/MS	By MS/MS	By MS/MS	By MS/MS	By MS/MS	By MS/MS	By MS/MS			11,6	69,15	0	43,20
P06730;A6NMX2	P06730	Eukaryotic translation initiation factor 4E	EIF4E	7	7	7	By MS/MS	By MS/MS	By MS/MS	By MS/MS	By MS/MS	By MS/MS	By MS/MS	By MS/MS	By MS/MS	By MS/MS	By MS/MS	By MS/MS	34,1	25,10	0	34,69
Q04637	Q04637	Eukaryotic translation initiation factor 4 gamma 1	EIF4G1	32	32	29	By MS/MS	By MS/MS	By MS/MS	By MS/MS	By MS/MS	By MS/MS	By MS/MS	By MS/MS	By MS/MS	By MS/MS	By MS/MS	By MS/MS	29,6	175,49	0	214,91
P78344	P78344	Eukaryotic translation initiation factor 4 gamma 2	EIF4G2	8	8	8	By MS/MS	By MS/MS	By MS/MS	By MS/MS	By MS/MS	By MS/MS			By MS/MS	By MS/MS	By MS/MS		12,6	102,36	0	37,66
Q15056	Q15056	Eukaryotic translation initiation factor 4H	EIF4H	11	11	11	By MS/MS	By MS/MS	By MS/MS	By MS/MS	By MS/MS	By MS/MS	By MS/MS	By MS/MS	By MS/MS	By MS/MS	By MS/MS	By MS/MS	52,0	27,39	0	129,92
P55010	P55010	Eukaryotic translation initiation factor 5	EIF5	10	10	10	By MS/MS	By MS/MS	By MS/MS	By MS/MS	By MS/MS	By MS/MS					By MS/MS	By MS/MS	22,7	49,22	0	24,41
P63241;Q6IS14;Q9GZV4	P63241;Q6IS14;Q9GZV4	Eukaryotic translation initiation factor 5A-1;Eukaryotic translation initiation factor 5A-1-like;Eukaryotic translation initiation factor 5A-2	EIF5A;EIF5AL1;EIF5A2	18	18	18	By MS/MS	By MS/MS	By MS/MS	By MS/MS	By MS/MS	By MS/MS	By MS/MS	By MS/MS	By MS/MS	By MS/MS	By MS/MS	By MS/MS	88,3	16,83	0	323,31
O60841	O60841	Eukaryotic translation initiation factor 5B	EIF5B	17	17	17	By MS/MS	By MS/MS	By MS/MS	By MS/MS	By MS/MS	By MS/MS	By MS/MS	By MS/MS	By MS/MS	By MS/MS	By MS/MS	By MS/MS	23,9	138,83	0	62,56
P56537	P56537	Eukaryotic translation initiation factor 6	EIF6	9	9	9	By MS/MS	By MS/MS	By MS/MS	By MS/MS	By MS/MS	By MS/MS	By MS/MS	By MS/MS	By MS/MS	By MS/MS	By MS/MS	By MS/MS	60,0	26,60	0	240,56
Q9BQ52	Q9BQ52	Zinc phosphodiesterase ELAC protein 2	ELAC2	8	8	8	By MS/MS	By MS/MS	By MS/MS	By MS/MS	By MS/MS	By MS/MS	By MS/MS	By MS/MS	By MS/MS	By MS/MS	By MS/MS	By MS/MS	14,5	92,22	0	19,46
Q15717	Q15717	ELAV-like protein 1	ELAVL1	10	10	10	By MS/MS	By MS/MS	By MS/MS	By MS/MS	By MS/MS	By MS/MS	By MS/MS	By MS/MS	By MS/MS	By MS/MS	By MS/MS	By MS/MS	40,2	36,09	0	90,11

Q8N766	Q8N766	ER membrane protein complex subunit 1	EMC1	10	10	10	By MS/MS	By MS/MS	By MS/MS	By MS/MS	By MS/MS	By MS/MS				By MS/MS			15,8	111,76	0	59,03
Q15006	Q15006	ER membrane protein complex subunit 2	EMC2	5	5	5	By MS/MS	By MS/MS	By MS/MS	By MS/MS	By MS/MS	By MS/MS	By MS/MS	By MS/MS			By MS/MS	By MS/MS	23,2	34,83	0	14,42
Q9P0I2	Q9P0I2	ER membrane protein complex subunit 3	EMC3	4	4	4		By MS/MS	By MS/MS	By MS/MS		By MS/MS				By MS/MS	By MS/MS	By MS/MS	19,5	29,95	0	71,34
Q9NPA0	Q9NPA0	ER membrane protein complex subunit 7	EMC7	5	5	5	By MS/MS	By MS/MS			By MS/MS	By MS/MS		By MS/MS					26,9	26,47	0	5,95
P50402	P50402	Emerin	EMD	6	6	6	By MS/MS	By MS/MS	By MS/MS	By MS/MS	By MS/MS	By MS/MS				By MS/MS	By MS/MS		33,1	28,99	0	323,31
Q92979	Q92979	Ribosomal RNA small subunit methyltransferase NEP1	EMG1	6	6	6	By MS/MS		By MS/MS	By MS/MS	By MS/MS	By MS/MS		By MS/MS	By MS/MS	By MS/MS	By MS/MS	By MS/MS	36,1	26,72	0	12,32
P06733;P13929	P06733	Alpha-enolase	ENO1	38	38	35	By MS/MS	By MS/MS	By MS/MS	By MS/MS	By MS/MS	By MS/MS	By MS/MS	By MS/MS	By MS/MS	By MS/MS	By MS/MS	By MS/MS	79,3	47,17	0	323,31
P09104	P09104	Gamma-enolase	ENO2	9	6	6	By MS/MS	By MS/MS	By MS/MS	By MS/MS	By MS/MS	By MS/MS	By MS/MS	By MS/MS	By matchin	By matchin	By MS/MS	By matchin	35,7	47,27	0	19,07
Q9UHY7	Q9UHY7	Enolase-phosphatase E1	ENOPH1	5	5	5	By MS/MS	By MS/MS	By MS/MS	By MS/MS	By MS/MS	By MS/MS			By MS/MS	By MS/MS	By MS/MS	By MS/MS	30,3	28,93	0	137,65
P11171	P11171	Protein 4.1	EPB41	6	6	6	By MS/MS	By MS/MS	By MS/MS	By MS/MS	By MS/MS	By MS/MS			By MS/MS		By MS/MS	By MS/MS	8,9	97,02	0	66,06
P34913	P34913	Bifunctional epoxide hydrolase 2;Cytosolic epoxide hydrolase 2;Lipid-phosphate phosphatase	EPHX2	5	5	5	By MS/MS	By MS/MS	By MS/MS	By MS/MS	By MS/MS	By MS/MS		By MS/MS				By MS/MS	14,2	62,62	0	11,22
P07814	P07814	Bifunctional glutamate/proline--tRNA ligase;Glutamate--tRNA ligase;Proline--tRNA ligase	EPRS	62	62	62	By MS/MS	By MS/MS	By MS/MS	By MS/MS	By MS/MS	By MS/MS	By MS/MS	By MS/MS	By MS/MS	By MS/MS	By MS/MS	By MS/MS	51,9	170,59	0	323,31
P42566	P42566	Epidermal growth factor receptor substrate 15	EPS15	8	8	8	By MS/MS	By MS/MS	By MS/MS	By MS/MS	By MS/MS	By MS/MS	By MS/MS				By MS/MS		13,3	98,66	0	77,90
P84090	P84090	Enhancer of rudimentary homolog	ERH	5	5	5	By MS/MS	By MS/MS	By MS/MS	By MS/MS	By MS/MS	By MS/MS	By MS/MS	By MS/MS	By MS/MS	By MS/MS	By MS/MS	By MS/MS	46,2	12,26	0	323,31
O75477	O75477	Erlin-1	ERLIN1	5	3	3	By matchin	By matchin	By matchin	By MS/MS	By MS/MS	By MS/MS	By MS/MS	By MS/MS	By MS/MS	By matching			22,8	38,93	0	4,84
O94905	O94905	Erlin-2	ERLIN2	10	10	8	By MS/MS	By MS/MS	By MS/MS	By MS/MS	By MS/MS	By MS/MS	By MS/MS	By MS/MS	By MS/MS	By MS/MS			41,0	37,84	0	35,78
Q96HE7;Q86YB8	Q96HE7	ERO1-like protein alpha	ERO1L	7	7	7	By MS/MS	By MS/MS	By MS/MS	By MS/MS	By MS/MS	By MS/MS	By MS/MS				By MS/MS	By MS/MS	21,6	54,39	0	25,66
P30040	P30040	Endoplasmic reticulum resident protein 29	ERP29	13	13	13	By MS/MS	By MS/MS	By MS/MS	By MS/MS	By MS/MS	By MS/MS	By MS/MS	By MS/MS	By MS/MS	By MS/MS	By MS/MS	By MS/MS	55,9	28,99	0	187,06
Q9BS26	Q9BS26	Endoplasmic reticulum resident protein 44	ERP44	12	12	12	By MS/MS	By MS/MS	By MS/MS	By MS/MS	By MS/MS	By MS/MS	By MS/MS	By MS/MS	By MS/MS	By MS/MS			39,9	46,97	0	121,53
P10768	P10768	S-formylglutathione hydrolase	ESD	10	10	10	By MS/MS	By MS/MS	By MS/MS	By MS/MS	By MS/MS	By MS/MS	By MS/MS	By MS/MS	By MS/MS	By MS/MS	By MS/MS	By MS/MS	55,3	31,46	0	308,84
Q9BSJ8	Q9BSJ8	Extended synaptotagmin-1	ESYT1	35	35	35	By MS/MS	By MS/MS	By MS/MS	By MS/MS	By MS/MS	By MS/MS	By MS/MS	By MS/MS	By MS/MS	By MS/MS	By MS/MS	By MS/MS	43,1	122,85	0	323,31
P62495	P62495	Eukaryotic peptide chain release factor subunit 1	ETF1	14	14	14	By MS/MS	By MS/MS	By MS/MS	By MS/MS	By MS/MS	By MS/MS	By MS/MS	By MS/MS			By MS/MS	By MS/MS	41,4	49,03	0	88,72
P13804	P13804	Electron transfer flavoprotein subunit alpha, mitochondrial	ETFA	14	14	14	By MS/MS	By MS/MS	By MS/MS	By MS/MS	By MS/MS	By MS/MS	By MS/MS	By MS/MS	By MS/MS	By MS/MS	By MS/MS	By MS/MS	65,2	35,08	0	200,18
P38117	P38117	Electron transfer flavoprotein subunit beta	ETFB	18	18	18	By MS/MS	By MS/MS	By MS/MS	By MS/MS	By MS/MS	By MS/MS	By MS/MS	By MS/MS	By MS/MS	By MS/MS	By MS/MS	By MS/MS	58,0	27,84	0	246,94
Q9Y3B2	Q9Y3B2	Exosome complex component CSL4	EXOSC1	4	4	4	By MS/MS	By MS/MS	By MS/MS	By MS/MS	By MS/MS	By MS/MS	By MS/MS	By MS/MS		By MS/MS	By MS/MS	By MS/MS	29,7	21,45	0	323,31
Q13868	Q13868	Exosome complex component RRP4	EXOSC2	4	4	4	By MS/MS	By MS/MS	By MS/MS	By MS/MS	By MS/MS	By MS/MS	By MS/MS	By MS/MS	By MS/MS	By MS/MS	By MS/MS	By MS/MS	19,1	32,79	0	10,00
Q9NPD3	Q9NPD3	Exosome complex component RRP41	EXOSC4	5	5	5	By MS/MS	By MS/MS	By MS/MS	By MS/MS	By MS/MS		By MS/MS	By MS/MS	By MS/MS	By MS/MS	By MS/MS	By MS/MS	26,5	26,38	0	69,35
Q9NQT4	Q9NQT4	Exosome complex component RRP46	EXOSC5	4	4	4	By MS/MS	By MS/MS		By MS/MS		By MS/MS	By MS/MS		By MS/MS	By MS/MS			29,4	25,25	0	9,67
Q5RKV6	Q5RKV6	Exosome complex component MTR3	EXOSC6	4	4	4						By MS/MS	By MS/MS	By MS/MS	By MS/MS	By MS/MS		By MS/MS	23,5	28,24	0	4,19
Q15024	Q15024	Exosome complex component RRP42	EXOSC7	6	6	6	By MS/MS	By MS/MS	By MS/MS		By MS/MS	By MS/MS	By MS/MS	By MS/MS	By MS/MS	By MS/MS			38,5	31,82	0	40,45
Q96B26	Q96B26	Exosome complex component RRP43	EXOSC8	5	5	5			By MS/MS			By MS/MS	By MS/MS	By MS/MS	By MS/MS	By MS/MS	By MS/MS	By MS/MS	31,2	30,04	0	30,42
Q99504	Q99504	Eyes absent homolog 3	EYA3	4	4	4	By MS/MS	By MS/MS	By MS/MS	By MS/MS	By MS/MS	By MS/MS				By MS/MS			10,5	62,66	0	14,21
P15311	P15311	Ezrin	EZR	25	17	16	By MS/MS	By MS/MS	By MS/MS	By MS/MS	By MS/MS	By MS/MS	By MS/MS	By MS/MS	By MS/MS	By MS/MS	By MS/MS	By MS/MS	48,5	69,41	0	323,31
Q01469;A8MUU1	Q01469	Fatty acid-binding protein, epidermal	FABP5	13	13	13	By MS/MS	By MS/MS	By MS/MS	By MS/MS	By MS/MS	By MS/MS	By MS/MS	By MS/MS	By MS/MS	By MS/MS	By MS/MS	By MS/MS	85,2	15,16	0	199,59
Q96CS3	Q96CS3	FAS-associated factor 2	FAF2	13	13	13	By MS/MS	By MS/MS	By MS/MS	By MS/MS	By MS/MS	By MS/MS	By MS/MS					By MS/MS	44,3	52,62	0	53,70
P16930	P16930	Fumarylacetoacetase	FAH	12	12	12	By MS/MS	By MS/MS	By MS/MS	By MS/MS	By MS/MS	By MS/MS	By MS/MS	By MS/MS	By MS/MS	By MS/MS			39,9	46,37	0	67,29
Q96TA1	Q96TA1	Niban-like protein 1	FAM129B	6	6	6	By MS/MS	By MS/MS	By MS/MS	By MS/MS	By MS/MS	By MS/MS		By MS/MS	By MS/MS	By MS/MS		By MS/MS	14,1	84,14	0	23,70
Q96C01	Q96C01	Protein FAM136A	FAM136A	5	5	5	By MS/MS	By MS/MS	By MS/MS	By MS/MS	By MS/MS	By MS/MS		By MS/MS	By MS/MS	By MS/MS	By MS/MS	By MS/MS	44,9	15,64	0	10,79
Q96A26	Q96A26	Protein FAM162A	FAM162A	6	6	6	By MS/MS	By MS/MS	By MS/MS	By MS/MS	By MS/MS	By MS/MS			By MS/MS	By MS/MS	By MS/MS	By MS/MS	37,0	17,34	0	10,58
Q9NSI2	Q9NSI2	Protein FAM207A	FAM207A	4	4	4		By MS/MS					By MS/MS	By MS/MS		By MS/MS	By MS/MS	By MS/MS	27,0	25,46	0	5,21
Q9NUQ9;Q9H0Q0	Q9NUQ9	Protein FAM49B	FAM49B	15	15	15	By MS/MS	By MS/MS	By MS/MS	By MS/MS	By MS/MS	By MS/MS	By MS/MS	By MS/MS	By MS/MS	By MS/MS	By MS/MS	By MS/MS	65,1	36,75	0	297,47
Q8NCA5	Q8NCA5	Protein FAM98A	FAM98A	4	4	4		By MS/MS				By MS/MS	By MS/MS	By MS/MS				By MS/MS	13,1	55,40	0	12,14
Q9NVI1	Q9NVI1	Fanconi anemia group I protein	FANCI	7	7	7	By MS/MS	By MS/MS	By MS/MS	By MS/MS		By MS/MS		By MS/MS					7,4	149,32	0	13,49
Q9Y285	Q9Y285	Phenylalanine--tRNA ligase alpha subunit	FARSA	17	17	17	By MS/MS	By MS/MS	By MS/MS	By MS/MS	By MS/MS	By MS/MS	By MS/MS	By MS/MS			By MS/MS	By MS/MS	45,7	57,56	0	172,70
Q9NSD9	Q9NSD9	Phenylalanine--tRNA ligase beta subunit	FARSB	15	15	15	By MS/MS	By MS/MS	By MS/MS	By MS/MS	By MS/MS	By MS/MS	By MS/MS	By MS/MS	By MS/MS	By MS/MS	By MS/MS	By MS/MS	29,2	66,12	0	63,34
P49327	P49327	Fatty acid synthase	FASN	121	121	121	By MS/MS	By MS/MS	By MS/MS	By MS/MS	By MS/MS	By MS/MS	By MS/MS	By MS/MS	By MS/MS	By MS/MS	By MS/MS	By MS/MS	65,1	273,42	0	323,31
P22087	P22087	rRNA 2-O-methyltransferase fibrillarin	FBL	10	10	9	By MS/MS	By MS/MS	By MS/MS	By MS/MS	By MS/MS	By MS/MS	By MS/MS	By MS/MS	By MS/MS	By MS/MS	By MS/MS	By MS/MS	43,3	33,78	0	81,00
P14324	P14324	Farnesyl pyrophosphate synthase	FDPS	9	9	9	By MS/MS	By MS/MS	By MS/MS	By MS/MS	By MS/MS	By MS/MS	By MS/MS	By MS/MS	By MS/MS	By MS/MS			31,3	48,28	0	17,21
P22570	P22570	NADPH:adrenodoxin oxidoreductase, mitochondrial	FDXR	6	6	6	By MS/MS	By MS/MS		By MS/MS	By MS/MS	By MS/MS		By MS/MS			By MS/MS	By MS/MS	14,9	53,84	0	8,31
P22830	P22830	Ferrochelatase, mitochondrial	FECH	11	11	11	By MS/MS	By MS/MS	By MS/MS	By MS/MS	By MS/MS	By MS/MS	By MS/MS	By MS/MS	By MS/MS	By MS/MS			46,8	47,86	0	24,83
P39748	P39748	Flap endonuclease 1	FEN1	11	11	11	By MS/MS	By MS/MS	By MS/MS	By MS/MS	By MS/MS	By MS/MS	By MS/MS	By MS/MS	By MS/MS	By MS/MS	By MS/MS	By MS/MS	41,1	42,59	0	224,90
Q86UX7	Q86UX7	Fermitin family homolog 3	FERMT3	19	19	19	By MS/MS	By MS/MS	By MS/MS	By MS/MS	By MS/MS	By MS/MS	By MS/MS	By MS/MS	By MS/MS	By MS/MS	By MS/MS	By MS/MS	45,1	75,95	0	212,58
P07954	P07954	Fumarate hydratase, mitochondrial	FH	20	20	20	By MS/MS	By MS/MS	By MS/MS	By MS/MS	By MS/MS	By MS/MS	By MS/MS	By MS/MS	By MS/MS	By MS/MS	By MS/MS	By MS/MS	55,9	54,64	0	323,31
Q9Y613	Q9Y613	FH1/FH2 domain-containing protein 1	FHOD1	8	8	8	By MS/MS	By MS/MS	By MS/MS	By MS/MS	By MS/MS	By MS/MS		By MS/MS					12,8	126,55	0	46,89
Q9Y3D6	Q9Y3D6	Mitochondrial fission 1 protein	FIS1	5	5	5	By MS/MS	By MS/MS		By MS/MS	By MS/MS	By MS/MS	By MS/MS	By MS/MS	By MS/MS	By MS/MS	By MS/MS	By MS/MS	34,9	16,94	0	80,89
P62942	P62942	Peptidyl-prolyl cis-trans isomerase FKBP1A	FKBP1A	6	6	6	By MS/MS	By MS/MS	By MS/MS	By MS/MS	By MS/MS	By MS/MS	By MS/MS	By MS/MS	By MS/MS	By MS/MS	By MS/MS	By MS/MS	50,0	11,95	0	108,12
P26885	P26885	Peptidyl-prolyl cis-trans isomerase FKBP2	FKBP2	4	4	4	By MS/MS	By MS/MS	By MS/MS	By MS/MS			By MS/MS	By MS/MS			By MS/MS	By MS/MS	39,4	15,65	0	20,59
Q00688	Q00688	Peptidyl-prolyl cis-trans isomerase FKBP3	FKBP3	6	6	6	By MS/MS	By MS/MS	By MS/MS	By MS/MS	By MS/MS	By MS/MS	By MS/MS	By MS/MS			By MS/MS	By MS/MS	31,2	25,18	0	12,36
Q02790	Q02790	Peptidyl-prolyl cis-trans isomerase FKBP4;Peptidyl-prolyl cis-trans isomerase FKBP4, N-terminally processed	FKBP4	29	29	29	By MS/MS	By MS/MS	By MS/MS	By MS/MS	By MS/MS	By MS/MS	By MS/MS	By MS/MS			By MS/MS	By MS/MS	73,9	51,80	0	323,31
Q13451	Q13451	Peptidyl-prolyl cis-trans isomerase FKBP5;Peptidyl-prolyl cis-trans isomerase FKBP5, N-terminally processed	FKBP5	18	18	18	By MS/MS	By MS/MS	By MS/MS	By MS/MS	By MS/MS	By MS/MS	By MS/MS	By MS/MS	By MS/MS	By MS/MS		By MS/MS	54,9	51,21	0	323,31
Q14318	Q14318	Peptidyl-prolyl cis-trans isomerase FKBP8	FKBP8	8	8	8	By MS/MS	By MS/MS	By MS/MS	By MS/MS	By MS/MS	By MS/MS					By MS/MS	By MS/MS	31,6	44,56	0	49,00
P21333	P21333	Filamin-A	FLNA	101	101	94	By MS/MS	By MS/MS	By MS/MS	By MS/MS	By MS/MS	By MS/MS	By MS/MS	By MS/MS	By MS/MS	By MS/MS	By MS/MS	By MS/MS	52,3	280,74	0	323,31
O75369	O75369	Filamin-B	FLNB	68	62	59	By MS/MS	By MS/MS	By MS/MS	By MS/MS	By MS/MS	By MS/MS	By MS/MS	By MS/MS	By MS/MS	By MS/MS	By MS/MS	By MS/MS	40,6	278,16	0	323,31
Q14315	Q14315	Filamin-C	FLNC	67	59	59	By MS/MS	By MS/MS	By MS/MS	By MS/MS	By MS/MS	By MS/MS	By MS/MS	By MS/MS	By MS/MS	By MS/MS	By MS/MS	By MS/MS	34,8	291,02	0	289,06
P49354	P49354	Protein farnesyltransferase/geranylgeranyltransferase type-1 subunit alpha	FNTA	4	4	4	By MS/MS	By MS/MS		By MS/MS	By MS/MS	By MS/MS		By MS/MS			By MS/MS		16,6	44,41	0	105,64
Q16658	Q16658	Fascin	FSCN1	31	31	31	By MS/MS	By MS/MS	By MS/MS	By MS/MS	By MS/MS	By MS/MS	By MS/MS	By MS/MS			By MS/MS	By MS/MS	66,3	54,53	0	323,31
P02794	P02794	Ferritin heavy chain;Ferritin heavy chain, N-terminally processed	FTH1	7	7	7	By MS/MS	By MS/MS	By MS/MS	By MS/MS	By MS/MS	By MS/MS	By MS/MS	By MS/MS	By MS/MS	By MS/MS	By MS/MS	By MS/MS	45,9	21,23	0	10,80
P02792	P02792	Ferritin light chain	FTL	7	7	7	By MS/MS	By MS/MS	By MS/MS	By MS/MS	By MS/MS	By MS/MS	By MS/MS	By MS/MS	By MS/MS	By MS/MS	By MS/MS	By MS/MS	48,6	20,02	0	17,47
Q96AE4	Q96AE4	Far upstream element-binding protein 1	FUBP1	11	11	10	By MS/MS	By MS/MS	By MS/MS	By MS/MS	By MS/MS	By MS/MS	By MS/MS	By MS/MS	By MS/MS	By MS/MS	By MS/MS	By MS/MS	24,1	67,56	0	118,67
Q96I24	Q96I24	Far upstream element-binding protein 3	FUBP3	8	7	7	By MS/MS	By MS/MS	By MS/MS	By MS/MS	By MS/MS	By MS/MS		By MS/MS			By MS/MS	By MS/MS	21,5	61,64	0	22,62

P35637	P35637	RNA-binding protein FUS	FUS	7	7	5	By MS/MS	By MS/MS	By MS/MS	By MS/MS	By MS/MS	By MS/MS	By MS/MS	By MS/MS	By MS/MS	By MS/MS	By MS/MS	By MS/MS	15,8	53,43	0	48,12
Q13283	Q13283	Ras GTPase-activating protein-binding protein 1	G3BP1	11	11	10	By MS/MS	By MS/MS	By MS/MS	By MS/MS	By MS/MS	By MS/MS	By MS/MS	By MS/MS	By MS/MS	By MS/MS	By MS/MS	By MS/MS	38,4	52,16	0	111,20
Q9UN86	Q9UN86	Ras GTPase-activating protein-binding protein 2	G3BP2	7	6	6	By MS/MS	By MS/MS	By MS/MS	By MS/MS	By MS/MS	By MS/MS					By MS/MS	By MS/MS	22,4	54,12	0	41,55
P11413	P11413	Glucose-6-phosphate 1-dehydrogenase	G6PD	30	30	30	By MS/MS	By MS/MS	By MS/MS	By MS/MS	By MS/MS	By MS/MS	By MS/MS	By MS/MS			By MS/MS	By MS/MS	75,3	59,26	0	323,31
P60520;Q9H0R8;O95166	P60520	Gamma-aminobutyric acid receptor-associated protein-like 2	GABARAPL2	7	7	7	By MS/MS	By MS/MS	By MS/MS	By MS/MS	By MS/MS	By MS/MS	By MS/MS	By MS/MS	By MS/MS	By MS/MS	By MS/MS	By MS/MS	41,9	13,67	0	19,28
Q8TAE8	Q8TAE8	Growth arrest and DNA damage-inducible proteins-interacting protein 1	GADD45GIP1	6	6	6	By MS/MS	By MS/MS	By MS/MS	By MS/MS	By MS/MS	By MS/MS	By MS/MS	By MS/MS	By MS/MS	By MS/MS	By MS/MS	By MS/MS	35,1	25,38	0	92,57
Q14376	Q14376	UDP-glucose 4-epimerase	GALE	5	5	5		By MS/MS		By MS/MS	By MS/MS	By MS/MS	By MS/MS		By MS/MS		By MS/MS	By MS/MS	24,4	38,28	0	101,99
P51570	P51570	Galactokinase	GALK1	5	5	5		By MS/MS			By MS/MS	By MS/MS	By MS/MS	By MS/MS	By MS/MS	By MS/MS			16,8	42,27	0	4,42
Q14697	Q14697	Neutral alpha-glucosidase AB	GANAB	43	43	43	By MS/MS	By MS/MS	By MS/MS	By MS/MS	By MS/MS	By MS/MS	By MS/MS	By MS/MS	By MS/MS	By MS/MS	By MS/MS	By MS/MS	56,8	106,87	0	323,31
P04406;O14556	P04406	Glyceraldehyde-3-phosphate dehydrogenase	GAPDH	22	22	22	By MS/MS	By MS/MS	By MS/MS	By MS/MS	By MS/MS	By MS/MS	By MS/MS	By MS/MS	By MS/MS	By MS/MS	By MS/MS	By MS/MS	77,6	36,05	0	323,31
P41250	P41250	Glycine--tRNA ligase	GARS	27	27	27	By MS/MS	By MS/MS	By MS/MS	By MS/MS	By MS/MS	By MS/MS	By MS/MS	By MS/MS	By MS/MS	By MS/MS	By MS/MS	By MS/MS	50,6	83,17	0	323,31
P22102	P22102	Trifunctional purine biosynthetic protein adenosine-3;Phosphoribosylamine--glycine ligase;Phosphoribosylformylglycinamidine cyclo-ligase;Phosphoribosylglycinamide formyltransferase	GART	35	35	35	By MS/MS	By MS/MS	By MS/MS	By MS/MS	By MS/MS	By MS/MS	By MS/MS	By MS/MS	By MS/MS	By MS/MS	By MS/MS	By MS/MS	50,2	107,77	0	323,31
O75323	O75323	Protein NipSnap homolog 2	GBAS	5	5	5	By MS/MS	By MS/MS	By MS/MS	By MS/MS	By MS/MS	By MS/MS	By MS/MS	By MS/MS				By MS/MS	22,7	33,74	0	9,77
P48507	P48507	Glutamate--cysteine ligase regulatory subunit	GCLM	4	4	4	By MS/MS	By MS/MS	By MS/MS	By MS/MS	By MS/MS	By MS/MS	By MS/MS	By MS/MS	By MS/MS		By MS/MS	By MS/MS	21,2	30,73	0	181,46
Q92616	Q92616	Translational activator GCN1	GCN1L1	88	88	88	By MS/MS	By MS/MS	By MS/MS	By MS/MS	By MS/MS	By MS/MS	By MS/MS	By MS/MS	By MS/MS	By MS/MS	By MS/MS	By MS/MS	45,6	292,75	0	323,31
P31150	P31150	Rab GDP dissociation inhibitor alpha	GDI1	16	6	6	By MS/MS	By MS/MS	By matchin	By MS/MS	By MS/MS	By MS/MS	By matchin	By matching			By MS/MS	By MS/MS	43,2	50,58	0	81,12
P50395	P50395	Rab GDP dissociation inhibitor beta	GDI2	43	43	33	By MS/MS	By MS/MS	By MS/MS	By MS/MS	By MS/MS	By MS/MS	By MS/MS	By MS/MS			By MS/MS	By MS/MS	89,4	50,66	0	323,31
P57678	P57678	Gem-associated protein 4	GEMIN4	4	4	4	By MS/MS	By MS/MS	By MS/MS		By MS/MS		By MS/MS					By MS/MS	5,9	120,04	0	7,02
Q8TEQ6	Q8TEQ6	Gem-associated protein 5	GEMIN5	11	11	11	By MS/MS	By MS/MS	By MS/MS	By MS/MS	By MS/MS	By MS/MS			By MS/MS	By MS/MS	By MS/MS	By MS/MS	9,8	168,59	0	90,61
Q7L5D6	Q7L5D6	Golgi to ER traffic protein 4 homolog	GET4	5	5	5	By MS/MS	By MS/MS	By MS/MS	By MS/MS	By MS/MS	By MS/MS	By MS/MS	By MS/MS	By MS/MS	By MS/MS	By MS/MS	By MS/MS	20,8	36,50	0	24,90
Q96RP9	Q96RP9	Elongation factor G, mitochondrial	GFM1	8	8	8	By MS/MS	By MS/MS		By MS/MS	By MS/MS	By MS/MS	By MS/MS	By MS/MS	By MS/MS	By MS/MS	By MS/MS	By MS/MS	12,6	83,47	0	22,09
Q06210;O94808	Q06210	Glutamine--fructose-6-phosphate aminotransferase [isomerizing] 1	GFPT1	20	20	20	By MS/MS	By MS/MS	By MS/MS	By MS/MS	By MS/MS	By MS/MS	By MS/MS	By MS/MS	By MS/MS	By MS/MS	By MS/MS	By MS/MS	39,3	78,81	0	220,77
O75223	O75223	Gamma-glutamylcyclotransferase	GGCT	8	8	8	By MS/MS	By MS/MS	By MS/MS	By MS/MS	By MS/MS	By MS/MS	By MS/MS	By MS/MS	By MS/MS	By MS/MS	By MS/MS	By MS/MS	46,8	21,01	0	244,37
O95749	O95749	Geranylgeranyl pyrophosphate synthase	GGPS1	6	6	6	By MS/MS	By MS/MS		By MS/MS	By MS/MS	By MS/MS				By MS/MS			28,7	34,87	0	7,78
Q14691	Q14691	DNA replication complex GINS protein PSF1	GINS1	5	5	5	By MS/MS	By MS/MS	By MS/MS	By MS/MS	By MS/MS	By MS/MS		By MS/MS			By MS/MS	By MS/MS	29,1	22,99	0	18,99
Q9BRX5	Q9BRX5	DNA replication complex GINS protein PSF3	GINS3	5	5	5	By MS/MS	By MS/MS	By MS/MS	By MS/MS	By MS/MS	By MS/MS	By MS/MS		By MS/MS				25,0	24,53	0	5,02
P16278	P16278	Beta-galactosidase	GLB1	4	4	4	By MS/MS	By MS/MS	By MS/MS	By MS/MS	By MS/MS	By MS/MS					By MS/MS	By MS/MS	8,1	76,07	0	19,28
Q04760	Q04760	Lactoylglutathione lyase	GLO1	18	18	18	By MS/MS	By MS/MS	By MS/MS	By MS/MS	By MS/MS	By MS/MS	By MS/MS	By MS/MS	By MS/MS	By MS/MS	By MS/MS	By MS/MS	87,5	20,78	0	179,15
Q9HC38	Q9HC38	Glyoxalase domain-containing protein 4	GLOD4	10	10	10	By MS/MS	By MS/MS	By MS/MS	By MS/MS	By MS/MS	By MS/MS	By MS/MS	By MS/MS	By MS/MS	By MS/MS	By MS/MS	By MS/MS	45,0	34,79	0	157,49
O76003	O76003	Glutaredoxin-3	GLRX3	16	16	16	By MS/MS	By MS/MS	By MS/MS	By MS/MS	By MS/MS	By MS/MS	By MS/MS	By MS/MS	By MS/MS	By MS/MS	By MS/MS	By MS/MS	57,3	37,43	0	152,07
O94925	O94925	Glutaminase kidney isoform, mitochondrial	GLS	4	4	4	By MS/MS	By MS/MS		By MS/MS	By MS/MS	By MS/MS						By MS/MS	9,3	73,46	0	10,38
P00367;P49448	P00367;P49448	Glutamate dehydrogenase 1, mitochondrial;Glutamate dehydrogenase 2, mitochondrial	GLUD1;GLUD2	20	20	20	By MS/MS	By MS/MS	By MS/MS	By MS/MS	By MS/MS	By MS/MS	By MS/MS	By MS/MS			By MS/MS	By MS/MS	47,7	61,40	0	121,72
P49915	P49915	GMP synthase [glutamine-hydrolyzing]	GMPS	27	27	27	By MS/MS	By MS/MS	By MS/MS	By MS/MS	By MS/MS	By MS/MS	By MS/MS	By MS/MS	By MS/MS	By MS/MS	By MS/MS	By MS/MS	53,8	76,72	0	323,31
P04899	P04899	Guanine nucleotide-binding protein G(i) subunit alpha-2	GNAI2	4	2	2	By matchin	By matchin	By MS/MS	By matchin	By MS/MS	By MS/MS		By matchin	By MS/MS	By MS/MS			14,4	40,45	0	8,30
P08754;P63096;P09471;P11488;P19087;A8MTJ3;P38405	P08754	Guanine nucleotide-binding protein G(k) subunit alpha	GNAI3	8	8	6	By MS/MS	By MS/MS	By MS/MS	By MS/MS	By MS/MS	By MS/MS	By MS/MS	By MS/MS	By MS/MS	By MS/MS			27,4	40,53	0	72,60
P63092;Q5JWF2	P63092;Q5JWF2	Guanine nucleotide-binding protein G(s) subunit alpha isoforms short;Guanine nucleotide-binding protein G(s) subunit alpha isoforms XLas	GNAS	4	3	3	By MS/MS	By MS/MS	By MS/MS	By MS/MS	By MS/MS	By MS/MS	By MS/MS	By MS/MS	By MS/MS	By MS/MS			15,5	45,66	0	9,42
P62873	P62873	Guanine nucleotide-binding protein G(I)/G(S)/G(T) subunit beta-1	GNB1	12	12	7	By MS/MS	By MS/MS	By MS/MS	By MS/MS	By MS/MS	By MS/MS	By MS/MS		By MS/MS	By MS/MS	By MS/MS	By MS/MS	46,2	37,38	0	53,19
P62879;Q9HAV0;P16520	P62879	Guanine nucleotide-binding protein G(I)/G(S)/G(T) subunit beta-2	GNB2	10	5	5	By MS/MS	By MS/MS	By MS/MS	By MS/MS	By MS/MS	By MS/MS	By matching		By MS/MS	By MS/MS	By matchin	By MS/MS	39,7	37,33	0	62,48
P63244	P63244	Guanine nucleotide-binding protein subunit beta-2-like 1;Guanine nucleotide-binding protein subunit beta-2-like 1, N-terminally processed	GNB2L1	20	20	20	By MS/MS	By MS/MS	By MS/MS	By MS/MS	By MS/MS	By MS/MS	By MS/MS	By MS/MS	By MS/MS	By MS/MS	By MS/MS	By MS/MS	81,4	35,08	0	323,31
P46926;Q8TDQ7	P46926	Glucosamine-6-phosphate isomerase 1	GNPDA1	12	12	12	By MS/MS	By MS/MS	By MS/MS	By MS/MS	By MS/MS	By MS/MS	By MS/MS	By MS/MS	By MS/MS	By MS/MS	By MS/MS	By MS/MS	54,3	32,67	0	90,30
Q96EK6	Q96EK6	Glucosamine 6-phosphate N-acetyltransferase	GNPNAT1	6	6	6	By MS/MS	By MS/MS	By MS/MS	By MS/MS	By MS/MS	By MS/MS	By MS/MS	By MS/MS	By MS/MS	By MS/MS	By MS/MS	By MS/MS	37,5	20,75	0	50,86
Q7Z5G4	Q7Z5G4	Golgin subfamily A member 7	GOLGA7	4	4	4	By MS/MS	By MS/MS	By MS/MS	By MS/MS	By MS/MS	By MS/MS				By MS/MS	By MS/MS	By MS/MS	32,8	15,82	0	10,05
Q9H4A6	Q9H4A6	Golgi phosphoprotein 3	GOLPH3	4	4	3	By MS/MS	By MS/MS	By MS/MS	By MS/MS	By MS/MS						By MS/MS		15,4	33,81	0	63,55
Q9H8Y8	Q9H8Y8	Golgi reassembly-stacking protein 2	GORASP2	7	7	7	By MS/MS	By MS/MS	By MS/MS	By MS/MS	By MS/MS	By MS/MS			By MS/MS	By MS/MS			27,2	47,15	0	77,30
P17174	P17174	Aspartate aminotransferase, cytoplasmic	GOT1	21	21	21	By MS/MS	By MS/MS	By MS/MS	By MS/MS	By MS/MS	By MS/MS	By MS/MS	By MS/MS	By MS/MS	By MS/MS			64,2	46,25	0	139,03
P00505	P00505	Aspartate aminotransferase, mitochondrial	GOT2	24	24	24	By MS/MS	By MS/MS	By MS/MS	By MS/MS	By MS/MS	By MS/MS	By MS/MS	By MS/MS	By MS/MS	By MS/MS			66,5	47,52	0	323,31
P43304	P43304	Glycerol-3-phosphate dehydrogenase, mitochondrial	GPD2	9	9	9	By MS/MS	By MS/MS	By MS/MS	By MS/MS	By MS/MS	By MS/MS	By MS/MS	By MS/MS			By MS/MS	By MS/MS	20,4	80,85	0	17,50
P06744;Q8N196	P06744	Glucose-6-phosphate isomerase	GPI	26	26	20	By MS/MS	By MS/MS	By MS/MS	By MS/MS	By MS/MS	By MS/MS	By MS/MS	By MS/MS			By MS/MS	By MS/MS	62,5	63,15	0	323,31
Q13098	Q13098	COP9 signalosome complex subunit 1	GPS1	4	4	4		By MS/MS		By MS/MS	By MS/MS	By MS/MS		By MS/MS					14,1	55,54	0	4,77
P36969	P36969	Phospholipid hydroperoxide glutathione peroxidase, mitochondrial	GPX4	5	5	5	By MS/MS	By MS/MS	By MS/MS	By MS/MS	By MS/MS	By MS/MS	By MS/MS	By MS/MS	By MS/MS	By MS/MS			35,0	22,17	0	16,44
O75791	O75791	GRB2-related adapter protein 2	GRAP2	5	5	5	By MS/MS	By MS/MS		By MS/MS	By MS/MS	By MS/MS		By MS/MS	By MS/MS		By MS/MS	By MS/MS	17,3	37,91	0	6,04
P62993	P62993	Growth factor receptor-bound protein 2	GRB2	8	8	8	By MS/MS	By MS/MS	By MS/MS	By MS/MS	By MS/MS	By MS/MS	By MS/MS	By MS/MS	By MS/MS	By MS/MS	By MS/MS	By MS/MS	43,3	25,21	0	32,34
Q9UBQ7	Q9UBQ7	Glyoxylate reductase/hydroxypyruvate reductase	GRHPR	12	12	12	By MS/MS	By MS/MS	By MS/MS	By MS/MS	By MS/MS	By MS/MS	By MS/MS	By MS/MS	By MS/MS	By MS/MS	By MS/MS	By MS/MS	52,4	35,67	0	323,31
Q9HAV7	Q9HAV7	GrpE protein homolog 1, mitochondrial	GRPEL1	10	10	10	By MS/MS	By MS/MS	By MS/MS	By MS/MS	By MS/MS	By MS/MS	By MS/MS	By MS/MS	By MS/MS	By MS/MS	By MS/MS	By MS/MS	52,1	24,28	0	50,66
Q9BQ67	Q9BQ67	Glutamate-rich WD repeat-containing protein 1	GRWD1	5	5	5	By MS/MS	By MS/MS	By MS/MS	By MS/MS	By MS/MS	By MS/MS					By MS/MS	By MS/MS	23,3	49,42	0	38,61
P15170	P15170	Eukaryotic peptide chain release factor GTP-binding subunit ERF3A	GSPT1	9	9	6	By MS/MS	By MS/MS	By MS/MS	By MS/MS	By MS/MS	By MS/MS	By MS/MS	By MS/MS		By MS/MS	By MS/MS	By MS/MS	27,9	55,76	0	17,93
P00390	P00390	Glutathione reductase, mitochondrial	GSR	17	17	17	By MS/MS	By MS/MS	By MS/MS	By MS/MS	By MS/MS	By MS/MS	By MS/MS	By MS/MS			By MS/MS	By MS/MS	57,5	56,26	0	256,59
P48637	P48637	Glutathione synthetase	GSS	13	13	13	By MS/MS	By MS/MS	By MS/MS	By MS/MS	By MS/MS	By MS/MS					By MS/MS	By MS/MS	39,9	52,38	0	206,24
Q9Y2Q3	Q9Y2Q3	Glutathione S-transferase kappa 1	GSTK1	6	6	6	By MS/MS	By MS/MS	By MS/MS	By MS/MS	By MS/MS	By MS/MS	By MS/MS	By MS/MS	By MS/MS	By MS/MS	By MS/MS	By MS/MS	34,1	25,50	0	49,78
P78417	P78417	Glutathione S-transferase omega-1	GSTO1	18	18	18	By MS/MS	By MS/MS	By MS/MS	By MS/MS	By MS/MS	By MS/MS	By MS/MS	By MS/MS	By MS/MS	By MS/MS	By MS/MS	By MS/MS	50,2	27,57	0	261,41

P09211	P09211	Glutathione S-transferase P	GSTP1	17	17	17	By MS/MS	By MS/MS	By MS/MS	By MS/MS	By MS/MS	By MS/MS	By MS/MS	By MS/MS	By MS/MS	By MS/MS	By MS/MS	By MS/MS	71,9	23,36	0	323,31
P35269	P35269	General transcription factor IIF subunit 1	GTF2F1	4	4	4	By MS/MS		By MS/MS		By MS/MS		By MS/MS	By MS/MS	By MS/MS	By MS/MS	By MS/MS	By MS/MS	9,7	58,24	0	17,66
P78347	P78347	General transcription factor II-I	GTF2I	12	12	12	By MS/MS	By MS/MS	By MS/MS	By MS/MS	By MS/MS	By MS/MS	By MS/MS	By MS/MS	By MS/MS	By MS/MS	By MS/MS	By MS/MS	19,7	112,42	0	43,00
Q71UI9;P0C0S5	Q71UI9;P0C0S5	Histone H2A.V;Histone H2A.Z	H2AFV;H2AFZ	6	4	4	By MS/MS	By MS/MS	By matchin	By matchin	By matchin	By matchin	By MS/MS	By MS/MS	By MS/MS	By MS/MS	By MS/MS	By MS/MS	53,9	13,51	0	183,87
Q9P035	Q9P035	Very-long-chain (3R)-3-hydroxyacyl-CoA dehydratase 3	HACD3	7	7	7	By MS/MS	By MS/MS	By MS/MS	By MS/MS	By MS/MS	By MS/MS	By MS/MS	By MS/MS	By MS/MS	By MS/MS	By MS/MS	By MS/MS	26,2	43,16	0	20,93
Q16836	Q16836	Hydroxyacyl-coenzyme A dehydrogenase, mitochondrial	HADH	10	10	10	By MS/MS	By MS/MS	By MS/MS	By MS/MS	By MS/MS	By MS/MS	By MS/MS	By MS/MS	By MS/MS	By MS/MS	By MS/MS		61,5	34,29	0	48,09
P40939	P40939	Trifunctional enzyme subunit alpha, mitochondrial;Long-chain enoyl-CoA hydratase;Long chain 3-hydroxyacyl-CoA dehydrogenase	HADHA	22	22	22	By MS/MS	By MS/MS	By MS/MS	By MS/MS	By MS/MS	By MS/MS	By MS/MS	By MS/MS	By MS/MS	By MS/MS	By MS/MS	By MS/MS	39,4	83,00	0	323,31
P55084	P55084	Trifunctional enzyme subunit beta, mitochondrial;3-ketoacyl-CoA thiolase	HADHB	18	18	18	By MS/MS	By MS/MS	By MS/MS	By MS/MS	By MS/MS	By MS/MS	By MS/MS	By MS/MS			By MS/MS	By MS/MS	49,4	51,29	0	108,73
P12081;P49590	P12081	Histidine--tRNA ligase, cytoplasmic	HARS	10	10	10	By MS/MS	By MS/MS	By MS/MS	By MS/MS	By MS/MS	By MS/MS	By MS/MS	By MS/MS			By MS/MS	By MS/MS	26,7	57,41	0	50,04
O14929	O14929	Histone acetyltransferase type B catalytic subunit	HAT1	11	11	11	By MS/MS	By MS/MS	By MS/MS	By MS/MS	By MS/MS	By MS/MS	By MS/MS	By MS/MS	By MS/MS	By MS/MS	By MS/MS	By MS/MS	39,9	49,51	0	323,31
P69905	P69905	Hemoglobin subunit alpha	HBA1	8	4	4	By MS/MS	By MS/MS	By MS/MS	By MS/MS	By MS/MS	By MS/MS	By MS/MS	By MS/MS	By MS/MS	By MS/MS	By MS/MS	By MS/MS	83,1	15,26	0	19,29
P02100	P02100	Hemoglobin subunit epsilon	HBE1	7	5	5	By MS/MS	By MS/MS	By MS/MS	By MS/MS	By MS/MS	By MS/MS	By MS/MS	By MS/MS	By MS/MS	By MS/MS	By MS/MS	By MS/MS	57,8	16,20	0	21,43
P69891	P69891	Hemoglobin subunit gamma-1	HBG1	16	2	2	By MS/MS	By MS/MS	By MS/MS	By MS/MS	By MS/MS	By MS/MS	By MS/MS	By MS/MS	By MS/MS	By MS/MS	By MS/MS	By MS/MS	95,2	16,14	0	133,05
P69892	P69892	Hemoglobin subunit gamma-2	HBG2	16	16	2	By MS/MS	By MS/MS	By MS/MS	By MS/MS	By MS/MS	By MS/MS	By MS/MS	By MS/MS	By MS/MS	By MS/MS	By MS/MS	By MS/MS	95,2	16,13	0	323,31
P09105	P09105	Hemoglobin subunit theta-1	HBQ1	7	7	7	By MS/MS	By MS/MS	By MS/MS	By MS/MS	By MS/MS	By MS/MS	By MS/MS	By MS/MS	By MS/MS	By MS/MS	By MS/MS	By MS/MS	64,1	15,51	0	18,55
Q9Y450	Q9Y450	HBS1-like protein	HBS1L	8	8	8	By MS/MS	By MS/MS	By MS/MS	By MS/MS	By MS/MS	By MS/MS	By MS/MS	By MS/MS	By MS/MS	By MS/MS	By MS/MS	By MS/MS	20,3	75,47	0	39,39
P02008	P02008	Hemoglobin subunit zeta	HBZ	12	12	11	By MS/MS	By MS/MS	By MS/MS	By MS/MS	By MS/MS	By MS/MS	By MS/MS	By MS/MS	By MS/MS	By MS/MS	By MS/MS	By MS/MS	93,0	15,64	0	323,31
P51610	P51610	Host cell factor 1;HCF N-terminal chain 1;HCF N-terminal chain 2;HCF N-terminal chain 3;HCF N-terminal chain 4;HCF N-terminal chain 5;HCF N-terminal chain 6;HCF C-terminal chain 1;HCF C-terminal chain 2;HCF C-terminal chain 3;HCF C-terminal chain 4;HCF C-terminal chain 5;HCF C-terminal chain 6	HCFC1	12	12	12	By MS/MS	By MS/MS	By MS/MS	By MS/MS	By MS/MS	By MS/MS	By MS/MS	By MS/MS			By MS/MS	By MS/MS	10,5	208,73	0	24,49
Q13547	Q13547	Histone deacetylase 1	HDAC1	6	4	4	By matchin	By matchin	By MS/MS	By MS/MS	By MS/MS	By MS/MS	By matchin	By matchin	By matchin	By matchin	By MS/MS	By matchin	20,7	55,10	0	12,73
Q92769	Q92769	Histone deacetylase 2	HDAC2	7	7	5	By MS/MS	By MS/MS	By MS/MS	By MS/MS	By MS/MS	By MS/MS	By MS/MS	By MS/MS	By MS/MS	By MS/MS	By MS/MS	By MS/MS	19,9	55,36	0	40,07
Q7Z4H3	Q7Z4H3	HD domain-containing protein 2	HDDC2	6	6	6	By MS/MS	By MS/MS	By MS/MS	By MS/MS	By MS/MS	By MS/MS	By MS/MS	By MS/MS	By MS/MS	By MS/MS	By MS/MS	By MS/MS	35,8	23,39	0	25,54
Q8N4P3	Q8N4P3	Guanosine-3,5-bis(diphosphate) 3-pyrophosphohydrolase MESH1	HDDC3	5	5	5	By MS/MS	By MS/MS	By MS/MS	By MS/MS	By MS/MS	By MS/MS			By MS/MS				31,8	20,33	0	26,98
P51858	P51858	Hepatoma-derived growth factor	HDGF	12	12	12	By MS/MS	By MS/MS	By MS/MS	By MS/MS	By MS/MS	By MS/MS	By MS/MS	By MS/MS	By MS/MS	By MS/MS			64,2	26,79	0	125,75
P07686	P07686	Beta-hexosaminidase subunit beta;Beta-hexosaminidase subunit beta chain B;Beta-hexosaminidase subunit beta chain A	HEXB	8	8	8	By MS/MS	By MS/MS	By MS/MS	By MS/MS	By MS/MS	By MS/MS	By MS/MS	By MS/MS	By MS/MS	By MS/MS	By MS/MS	By MS/MS	21,9	63,11	0	46,95
Q9BTY7	Q9BTY7	Protein HGH1 homolog	HGH1	6	6	6	By MS/MS	By MS/MS	By MS/MS	By MS/MS	By MS/MS	By MS/MS	By MS/MS	By MS/MS			By MS/MS		31,0	42,13	0	14,16
P31937	P31937	3-hydroxyisobutyrate dehydrogenase, mitochondrial	HIBADH	4	4	4	By MS/MS		By MS/MS	By MS/MS	By MS/MS	By MS/MS	By MS/MS	By MS/MS	By MS/MS			By MS/MS	19,9	35,33	0	190,51
Q6NVY1	Q6NVY1	3-hydroxyisobutyryl-CoA hydrolase, mitochondrial	HIBCH	8	8	8	By MS/MS	By MS/MS		By MS/MS	By MS/MS	By MS/MS	By MS/MS	By MS/MS	By MS/MS	By MS/MS			27,5	43,48	0	14,49
P49773	P49773	Histidine triad nucleotide-binding protein 1	HINT1	10	10	10	By MS/MS	By MS/MS	By MS/MS	By MS/MS	By MS/MS	By MS/MS	By MS/MS	By MS/MS	By MS/MS	By MS/MS	By MS/MS	By MS/MS	87,3	13,80	0	40,59
Q9BX68	Q9BX68	Histidine triad nucleotide-binding protein 2, mitochondrial	HINT2	5	5	5	By MS/MS	By MS/MS	By MS/MS	By MS/MS	By MS/MS	By MS/MS						By MS/MS	52,1	17,16	0	19,80
P16402	P16402	Histone H1.3	HIST1H1D	10	3	3	By MS/MS	By matchin	By MS/MS	By MS/MS	By MS/MS	By MS/MS	By MS/MS	By MS/MS	By MS/MS	By MS/MS	By MS/MS	By MS/MS	30,3	22,35	0	13,54
P10412;P22492;Q02539	P10412	Histone H1.4	HIST1H1E	10	10	3	By MS/MS	By MS/MS	By MS/MS	By MS/MS	By MS/MS	By MS/MS	By MS/MS	By MS/MS	By MS/MS	By MS/MS	By MS/MS	By MS/MS	26,9	21,87	0	62,57
Q99879;Q99877;Q93079;Q5QNW6;P62807;P58876;O60814;Q99880;P57053;Q96A08	Q99879;Q99877;Q93079;Q5QNW6;P62807;P58876;O60814;Q99880;P57053	Histone H2B type 1-M;Histone H2B type 1-N;Histone H2B type 1-H;Histone H2B type 2-F;Histone H2B type 1-C/E/F/G/I;Histone H2B type 1-D;Histone H2B type 1-K;Histone H2B type 1-L;Histone H2B type F-S	HIST1H2BM;HIST1H2BN;HIST1H2BH;HIST2H2BF;HIST1H2BC;HIST1H2BD;HIST1H2BK;HIST1H2BL;H2BFS	11	11	3	By MS/MS	By MS/MS	By MS/MS	By MS/MS	By MS/MS	By MS/MS	By MS/MS	By MS/MS	By MS/MS	By MS/MS	By MS/MS	By MS/MS	65,1	13,99	0	323,31
P62805	P62805	Histone H4	HIST1H4A	17	17	17	By MS/MS	By MS/MS	By MS/MS	By MS/MS	By MS/MS	By MS/MS	By MS/MS	By MS/MS	By MS/MS	By MS/MS	By MS/MS	By MS/MS	68,0	11,37	0	323,31
Q71DI3;Q16695;P84243;P68431;Q6NXT2	Q71DI3;Q16695;P84243;P68431;Q6NXT2	Histone H3.2;Histone H3.1t;Histone H3.3;Histone H3.1;Histone H3.3C	HIST2H3A;HIST3H3;H3F3A;HIST1H3A;H3F3C	4	4	4	By MS/MS	By MS/MS	By MS/MS	By MS/MS	By MS/MS	By MS/MS	By MS/MS	By MS/MS	By MS/MS	By MS/MS	By MS/MS	By MS/MS	19,9	15,39	0	8,79
P19367;Q2TB90	P19367	Hexokinase-1	HK1	34	34	31	By MS/MS	By MS/MS	By MS/MS	By MS/MS	By MS/MS	By MS/MS	By MS/MS	By MS/MS	By MS/MS	By MS/MS	By MS/MS	By MS/MS	41,8	102,48	0	248,70
P52789;P52790	P52789	Hexokinase-2	HK2	20	17	17	By MS/MS	By MS/MS	By MS/MS	By MS/MS	By MS/MS	By MS/MS			By matching		By MS/MS		27,5	102,38	0	44,60
Q8TCT9	Q8TCT9	Minor histocompatibility antigen H13	HM13	6	6	6	By MS/MS			By MS/MS	By MS/MS	By MS/MS	By MS/MS	By MS/MS	By MS/MS	By MS/MS			26,3	41,49	0	8,93
P08397	P08397	Porphobilinogen deaminase	HMBS	15	15	15	By MS/MS	By MS/MS	By MS/MS	By MS/MS	By MS/MS	By MS/MS	By MS/MS	By MS/MS	By MS/MS	By MS/MS	By MS/MS	By MS/MS	56,2	39,33	0	323,31
P09429;B2RPK0;P23497	P09429	High mobility group protein B1	HMGB1	19	19	16	By MS/MS	By MS/MS	By MS/MS	By MS/MS	By MS/MS	By MS/MS	By MS/MS	By MS/MS	By MS/MS	By MS/MS	By MS/MS	By MS/MS	55,8	24,89	0	115,84
P26583	P26583	High mobility group protein B2	HMGB2	13	10	10	By MS/MS	By MS/MS	By MS/MS	By MS/MS	By MS/MS	By MS/MS	By MS/MS	By MS/MS	By MS/MS	By MS/MS	By MS/MS	By MS/MS	41,1	24,03	0	31,24
O15347	O15347	High mobility group protein B3	HMGB3	4	4	4	By MS/MS	By MS/MS					By MS/MS		By MS/MS		By MS/MS	By MS/MS	27,0	22,98	0	3,91
Q01581;P54868	Q01581	Hydroxymethylglutaryl-CoA synthase, cytoplasmic	HMGCS1	21	21	21	By MS/MS	By MS/MS	By MS/MS	By MS/MS	By MS/MS	By MS/MS	By MS/MS	By MS/MS			By MS/MS	By MS/MS	65,0	57,29	0	321,40
P30519	P30519	Heme oxygenase 2	HMOX2	7	7	7	By MS/MS	By MS/MS	By MS/MS	By MS/MS	By MS/MS	By MS/MS	By MS/MS	By MS/MS	By MS/MS	By MS/MS		By MS/MS	33,9	36,03	0	61,08
Q13151	Q13151	Heterogeneous nuclear ribonucleoprotein A0	HNRNPA0	9	9	9	By MS/MS	By MS/MS	By MS/MS	By MS/MS	By MS/MS	By MS/MS	By MS/MS	By MS/MS	By MS/MS	By MS/MS	By MS/MS	By MS/MS	32,1	30,84	0	98,07
P09651;Q32P51	P09651;Q32P51	Heterogeneous nuclear ribonucleoprotein A1;Heterogeneous nuclear ribonucleoprotein A1, N-terminally processed;Heterogeneous nuclear ribonucleoprotein A1-like 2	HNRNPA1;HNRNPA1L2	22	22	21	By MS/MS	By MS/MS	By MS/MS	By MS/MS	By MS/MS	By MS/MS	By MS/MS	By MS/MS	By MS/MS	By MS/MS	By MS/MS	By MS/MS	47,0	38,75	0	323,31
P22626	P22626	Heterogeneous nuclear ribonucleoproteins A2/B1	HNRNPA2B1	20	19	19	By MS/MS	By MS/MS	By MS/MS	By MS/MS	By MS/MS	By MS/MS	By MS/MS	By MS/MS	By MS/MS	By MS/MS	By MS/MS	By MS/MS	63,7	37,43	0	288,88
P51991	P51991	Heterogeneous nuclear ribonucleoprotein A3	HNRNPA3	15	15	15	By MS/MS	By MS/MS	By MS/MS	By MS/MS	By MS/MS	By MS/MS	By MS/MS	By MS/MS	By MS/MS	By MS/MS			42,9	39,59	0	139,82
Q99729	Q99729	Heterogeneous nuclear ribonucleoprotein A/B	HNRNPAB	5	5	5	By MS/MS	By MS/MS	By MS/MS	By MS/MS	By MS/MS	By MS/MS	By MS/MS		By MS/MS	By MS/MS			17,2	36,22	0	10,75
P07910;P0DMR1;O60812;B7ZW38;B2RXH8;Q86SE5	P07910;P0DMR1;O60812;B7ZW38;B2RXH8	Heterogeneous nuclear ribonucleoproteins C1/C2;Heterogeneous nuclear ribonucleoprotein C-like 4;Heterogeneous nuclear ribonucleoprotein C-like 1;Heterogeneous nuclear ribonucleoprotein C-like 3;Heterogeneous nuclear ribonucleoprotein C-like 2	HNRNPC;HNRNPCL4;HNRNPCL1;HNRNPCL3;HNRNPCL2	12	12	12	By MS/MS	By MS/MS	By MS/MS	By MS/MS	By MS/MS	By MS/MS	By MS/MS	By MS/MS	By MS/MS	By MS/MS	By MS/MS	By MS/MS	29,1	33,67	0	58,89
Q14103	Q14103	Heterogeneous nuclear ribonucleoprotein D0	HNRNPD	17	17	15	By MS/MS	By MS/MS	By MS/MS	By MS/MS	By MS/MS	By MS/MS	By MS/MS	By MS/MS	By MS/MS	By MS/MS	By MS/MS	By MS/MS	34,4	38,43	0	276,88
O14979	O14979	Heterogeneous nuclear ribonucleoprotein D-like	HNRNPDL	7	5	5	By MS/MS	By MS/MS	By MS/MS	By MS/MS	By MS/MS	By MS/MS	By MS/MS	By MS/MS	By MS/MS	By MS/MS	By matching		16,0	46,44	0	46,90

P52597	P52597	Heterogeneous nuclear ribonucleoprotein F;Heterogeneous nuclear ribonucleoprotein F, N-terminally processed	HNRNPF	14	12	12	By MS/MS	By MS/MS	By MS/MS	By MS/MS	By MS/MS	By MS/MS	By MS/MS	By MS/MS	By MS/MS	By MS/MS	By MS/MS	By MS/MS	54,9	45,67	0	323,31
P31943	P31943	Heterogeneous nuclear ribonucleoprotein H;Heterogeneous nuclear ribonucleoprotein H, N-terminally processed	HNRNPH1	14	14	9	By MS/MS	By MS/MS	By MS/MS	By MS/MS	By MS/MS	By MS/MS	By MS/MS	By MS/MS	By MS/MS	By MS/MS	By MS/MS	By MS/MS	42,3	49,23	0	323,31
P55795	P55795	Heterogeneous nuclear ribonucleoprotein H2	HNRNPH2	8	4	4	By matchin	By matchin	By MS/MS	By matchin	By MS/MS	By MS/MS	By MS/MS	By MS/MS	By matchin	By MS/MS	By MS/MS	By MS/MS	27,6	49,26	0	12,68
P31942	P31942	Heterogeneous nuclear ribonucleoprotein H3	HNRNPH3	7	7	7	By MS/MS	By MS/MS	By MS/MS	By MS/MS	By MS/MS	By MS/MS	By MS/MS	By MS/MS	By MS/MS	By MS/MS	By MS/MS	By MS/MS	34,7	36,93	0	83,51
P61978	P61978	Heterogeneous nuclear ribonucleoprotein K	HNRNPK	24	24	24	By MS/MS	By MS/MS	By MS/MS	By MS/MS	By MS/MS	By MS/MS	By MS/MS	By MS/MS	By MS/MS	By MS/MS	By MS/MS	By MS/MS	56,4	50,98	0	323,31
P14866	P14866	Heterogeneous nuclear ribonucleoprotein L	HNRNPL	16	16	16	By MS/MS	By MS/MS	By MS/MS	By MS/MS	By MS/MS	By MS/MS	By MS/MS	By MS/MS	By MS/MS	By MS/MS	By MS/MS	By MS/MS	42,1	64,13	0	205,42
P52272	P52272	Heterogeneous nuclear ribonucleoprotein M	HNRNPM	24	24	24	By MS/MS	By MS/MS	By MS/MS	By MS/MS	By MS/MS	By MS/MS	By MS/MS	By MS/MS	By MS/MS	By MS/MS	By MS/MS	By MS/MS	42,7	77,52	0	323,31
O43390	O43390	Heterogeneous nuclear ribonucleoprotein R	HNRNPR	21	16	16	By MS/MS	By MS/MS	By MS/MS	By MS/MS	By MS/MS	By MS/MS	By MS/MS	By MS/MS	By MS/MS	By MS/MS	By MS/MS	By MS/MS	36,3	70,94	0	109,56
Q00839	Q00839	Heterogeneous nuclear ribonucleoprotein U	HNRNPU	26	26	26	By MS/MS	By MS/MS	By MS/MS	By MS/MS	By MS/MS	By MS/MS	By MS/MS	By MS/MS	By MS/MS	By MS/MS	By MS/MS	By MS/MS	35,0	90,58	0	323,31
Q9BUJ2	Q9BUJ2	Heterogeneous nuclear ribonucleoprotein U-like protein 1	HNRNPUL1	10	10	10	By MS/MS	By MS/MS	By MS/MS	By MS/MS	By MS/MS	By MS/MS	By MS/MS	By MS/MS			By MS/MS	By MS/MS	18,2	95,74	0	161,23
Q1KMD3	Q1KMD3	Heterogeneous nuclear ribonucleoprotein U-like protein 2	HNRNPUL2	11	11	11	By MS/MS	By MS/MS	By MS/MS	By MS/MS	By MS/MS	By MS/MS	By MS/MS	By MS/MS	By MS/MS	By MS/MS	By MS/MS	By MS/MS	19,7	85,10	0	225,25
Q5SSJ5	Q5SSJ5	Heterochromatin protein 1-binding protein 3	HP1BP3	6	6	6	By MS/MS	By MS/MS	By MS/MS	By MS/MS	By MS/MS	By MS/MS					By MS/MS		15,6	61,21	0	8,27
P37235;P84074	P37235;P84074	Hippocalcin-like protein 1;Neuron-specific calcium-binding protein hippocalcin	HPCAL1;HPCA	5	5	4	By MS/MS	By MS/MS	By MS/MS	By MS/MS		By MS/MS	By MS/MS	By MS/MS	By MS/MS	By MS/MS	By MS/MS	By MS/MS	30,1	22,31	0	8,64
P00492	P00492	Hypoxanthine-guanine phosphoribosyltransferase	HPRT1	8	8	8	By MS/MS	By MS/MS	By MS/MS	By MS/MS	By MS/MS	By MS/MS	By MS/MS	By MS/MS	By MS/MS	By MS/MS	By MS/MS	By MS/MS	39,4	24,58	0	66,12
P52758	P52758	Ribonuclease UK114	HRSP12	5	5	5	By MS/MS	By MS/MS	By MS/MS	By MS/MS	By MS/MS	By MS/MS	By MS/MS	By MS/MS	By MS/MS	By MS/MS	By MS/MS	By MS/MS	53,3	14,49	0	128,77
Q99714	Q99714	3-hydroxyacyl-CoA dehydrogenase type-2	HSD17B10	16	16	16	By MS/MS	By MS/MS	By MS/MS	By MS/MS	By MS/MS	By MS/MS	By MS/MS	By MS/MS	By MS/MS	By MS/MS	By MS/MS	By MS/MS	78,9	26,92	0	323,31
Q53GQ0	Q53GQ0	Very-long-chain 3-oxoacyl-CoA reductase	HSD17B12	9	9	9	By MS/MS	By MS/MS	By MS/MS	By MS/MS	By MS/MS	By MS/MS	By MS/MS	By MS/MS	By MS/MS	By MS/MS	By MS/MS	By MS/MS	43,3	34,32	0	28,21
P51659	P51659	Peroxisomal multifunctional enzyme type 2;(3R)-hydroxyacyl-CoA dehydrogenase;Enoyl-CoA hydratase 2	HSD17B4	12	12	12	By MS/MS	By MS/MS	By MS/MS	By MS/MS	By MS/MS	By MS/MS	By MS/MS	By MS/MS	By MS/MS	By MS/MS	By MS/MS	By MS/MS	28,5	79,69	0	115,31
P07900;Q58FG0	P07900	Heat shock protein HSP 90-alpha	HSP90AA1	61	43	41	By MS/MS	By MS/MS	By MS/MS	By MS/MS	By MS/MS	By MS/MS	By MS/MS	By MS/MS	By MS/MS	By MS/MS	By MS/MS	By MS/MS	62,3	84,66	0	323,31
P08238;Q58FF7	P08238	Heat shock protein HSP 90-beta	HSP90AB1	61	61	38	By MS/MS	By MS/MS	By MS/MS	By MS/MS	By MS/MS	By MS/MS	By MS/MS	By MS/MS	By MS/MS	By MS/MS	By MS/MS	By MS/MS	71,8	83,26	0	323,31
Q58FF8	Q58FF8	Putative heat shock protein HSP 90-beta 2	HSP90AB2P	13	2	2	By MS/MS	By MS/MS	By MS/MS	By MS/MS	By MS/MS	By MS/MS	By MS/MS	By MS/MS	By MS/MS	By MS/MS	By MS/MS	By MS/MS	21,5	44,35	0	300,02
Q58FF6	Q58FF6	Putative heat shock protein HSP 90-beta 4	HSP90AB4P	9	3	3	By matchin	By matchin	By matchin	By matchin	By MS/MS	By matchin	By MS/MS	By MS/MS	By MS/MS	By MS/MS	By MS/MS	By MS/MS	17,0	58,26	0	37,05
P14625;Q58FF3	P14625	Endoplasmin	HSP90B1	36	34	34	By MS/MS	By MS/MS	By MS/MS	By MS/MS	By MS/MS	By MS/MS	By MS/MS	By MS/MS	By MS/MS	By MS/MS	By MS/MS	By MS/MS	48,9	92,47	0	323,31
Q0VDF9	Q0VDF9	Heat shock 70 kDa protein 14	HSPA14	5	5	5	By MS/MS	By MS/MS	By MS/MS	By MS/MS	By MS/MS	By MS/MS					By MS/MS		18,1	54,79	0	17,56
P0DMV9;P0DMV8	P0DMV9;P0DMV8	Heat shock 70 kDa protein 1B;Heat shock 70 kDa protein 1A	HSPA1B;HSPA1A	31	30	21	By MS/MS	By MS/MS	By MS/MS	By MS/MS	By MS/MS	By MS/MS	By MS/MS	By MS/MS	By MS/MS	By MS/MS	By MS/MS	By MS/MS	58,2	70,05	0	323,31
P34932	P34932	Heat shock 70 kDa protein 4	HSPA4	48	48	45	By MS/MS	By MS/MS	By MS/MS	By MS/MS	By MS/MS	By MS/MS	By MS/MS	By MS/MS	By MS/MS	By MS/MS	By MS/MS	By MS/MS	66,0	94,33	0	323,31
O95757	O95757	Heat shock 70 kDa protein 4L	HSPA4L	18	15	15	By MS/MS	By MS/MS	By MS/MS	By MS/MS	By MS/MS	By MS/MS	By MS/MS	By MS/MS	By MS/MS	By MS/MS	By MS/MS	By MS/MS	30,0	94,51	0	77,78
P11021	P11021	78 kDa glucose-regulated protein	HSPA5	33	32	32	By MS/MS	By MS/MS	By MS/MS	By MS/MS	By MS/MS	By MS/MS	By MS/MS	By MS/MS	By MS/MS	By MS/MS	By MS/MS	By MS/MS	48,5	72,33	0	323,31
P11142	P11142	Heat shock cognate 71 kDa protein	HSPA8	40	40	30	By MS/MS	By MS/MS	By MS/MS	By MS/MS	By MS/MS	By MS/MS	By MS/MS	By MS/MS	By MS/MS	By MS/MS	By MS/MS	By MS/MS	64,9	70,90	0	323,31
P38646	P38646	Stress-70 protein, mitochondrial	HSPA9	35	35	35	By MS/MS	By MS/MS	By MS/MS	By MS/MS	By MS/MS	By MS/MS	By MS/MS	By MS/MS	By MS/MS	By MS/MS	By MS/MS	By MS/MS	54,1	73,68	0	323,31
P04792	P04792	Heat shock protein beta-1	HSPB1	12	12	12	By MS/MS	By MS/MS	By MS/MS	By MS/MS	By MS/MS	By MS/MS	By MS/MS	By MS/MS	By MS/MS	By MS/MS	By MS/MS	By MS/MS	72,2	22,78	0	190,39
Q9NZL4	Q9NZL4	Hsp70-binding protein 1	HSPBP1	5	5	5	By MS/MS	By MS/MS	By MS/MS	By MS/MS	By MS/MS	By MS/MS	By MS/MS	By MS/MS	By MS/MS	By MS/MS		By MS/MS	18,2	39,47	0	25,86
P10809	P10809	60 kDa heat shock protein, mitochondrial	HSPD1	47	47	47	By MS/MS	By MS/MS	By MS/MS	By MS/MS	By MS/MS	By MS/MS	By MS/MS	By MS/MS	By MS/MS	By MS/MS	By MS/MS	By MS/MS	79,6	61,05	0	323,31
P61604	P61604	10 kDa heat shock protein, mitochondrial	HSPE1	14	14	14	By MS/MS	By MS/MS	By MS/MS	By MS/MS	By MS/MS	By MS/MS	By MS/MS	By MS/MS	By MS/MS	By MS/MS	By MS/MS	By MS/MS	88,2	10,93	0	186,60
Q92598	Q92598	Heat shock protein 105 kDa	HSPH1	33	31	30	By MS/MS	By MS/MS	By MS/MS	By MS/MS	By MS/MS	By MS/MS	By MS/MS	By MS/MS	By MS/MS	By MS/MS	By MS/MS	By MS/MS	45,8	96,86	0	323,31
Q9BUP3	Q9BUP3	Oxidoreductase HTATIP2	HTATIP2	4	4	4	By MS/MS	By MS/MS	By MS/MS	By MS/MS		By MS/MS	By MS/MS	By MS/MS					20,7	27,05	0	74,25
Q7Z6Z7	Q7Z6Z7	E3 ubiquitin-protein ligase HUWE1	HUWE1	44	44	44	By MS/MS	By MS/MS	By MS/MS	By MS/MS	By MS/MS	By MS/MS	By MS/MS	By MS/MS	By MS/MS	By MS/MS	By MS/MS	By MS/MS	17,8	481,89	0	274,49
Q9Y4L1;Q9BXT5	Q9Y4L1	Hypoxia up-regulated protein 1	HYOU1	46	46	46	By MS/MS	By MS/MS	By MS/MS	By MS/MS	By MS/MS	By MS/MS	By MS/MS	By MS/MS	By MS/MS	By MS/MS	By MS/MS	By MS/MS	50,6	111,33	0	323,31
P41252	P41252	Isoleucine--tRNA ligase, cytoplasmic	IARS	52	52	52	By MS/MS	By MS/MS	By MS/MS	By MS/MS	By MS/MS	By MS/MS	By MS/MS	By MS/MS	By MS/MS	By MS/MS	By MS/MS	By MS/MS	49,7	144,50	0	323,31
Q9NSE4	Q9NSE4	Isoleucine--tRNA ligase, mitochondrial	IARS2	19	19	19	By MS/MS	By MS/MS	By MS/MS	By MS/MS	By MS/MS	By MS/MS		By MS/MS			By MS/MS	By MS/MS	20,9	113,79	0	76,17
Q5T440	Q5T440	Putative transferase CAF17, mitochondrial	IBA57	4	4	4	By MS/MS	By MS/MS		By MS/MS	By MS/MS	By MS/MS				By MS/MS		By MS/MS	20,8	38,16	0	21,88
P14735	P14735	Insulin-degrading enzyme	IDE	8	8	8	By MS/MS	By MS/MS	By MS/MS	By MS/MS	By MS/MS	By MS/MS	By MS/MS	By MS/MS	By MS/MS	By MS/MS	By MS/MS	By MS/MS	9,6	117,97	0	15,18
O75874	O75874	Isocitrate dehydrogenase [NADP] cytoplasmic	IDH1	13	12	12	By MS/MS	By MS/MS	By MS/MS	By MS/MS	By MS/MS	By MS/MS	By MS/MS	By MS/MS	By MS/MS	By MS/MS	By MS/MS	By MS/MS	33,1	46,66	0	91,75
P48735	P48735	Isocitrate dehydrogenase [NADP], mitochondrial	IDH2	18	18	17	By MS/MS	By MS/MS	By MS/MS	By MS/MS	By MS/MS	By MS/MS	By MS/MS	By MS/MS	By MS/MS	By MS/MS	By MS/MS	By MS/MS	41,2	50,91	0	305,49
P50213	P50213	Isocitrate dehydrogenase [NAD] subunit alpha, mitochondrial	IDH3A	7	7	7	By MS/MS	By MS/MS	By MS/MS	By MS/MS	By MS/MS	By MS/MS	By MS/MS	By MS/MS	By MS/MS	By MS/MS			26,5	39,59	0	16,85
O43837	O43837	Isocitrate dehydrogenase [NAD] subunit beta, mitochondrial	IDH3B	6	6	6	By MS/MS	By MS/MS	By MS/MS	By MS/MS	By MS/MS	By MS/MS		By MS/MS	By MS/MS	By MS/MS			19,5	42,18	0	34,94
Q13907	Q13907	Isopentenyl-diphosphate Delta-isomerase 1	IDI1	8	8	8	By MS/MS	By MS/MS	By MS/MS	By MS/MS	By MS/MS	By MS/MS	By MS/MS	By MS/MS	By MS/MS	By MS/MS	By MS/MS	By MS/MS	37,9	26,32	0	36,50
Q9NZI8;Q9Y6M1	Q9NZI8	Insulin-like growth factor 2 mRNA-binding protein 1	IGF2BP1	12	10	10	By MS/MS	By MS/MS	By MS/MS	By MS/MS	By MS/MS	By MS/MS	By MS/MS	By MS/MS	By MS/MS	By MS/MS	By MS/MS	By MS/MS	26,7	63,48	0	209,09
O00425	O00425	Insulin-like growth factor 2 mRNA-binding protein 3	IGF2BP3	13	13	11	By MS/MS	By MS/MS	By MS/MS	By MS/MS	By MS/MS	By MS/MS	By MS/MS	By MS/MS	By MS/MS	By MS/MS	By MS/MS	By MS/MS	30,7	63,70	0	47,46
P11717	P11717	Cation-independent mannose-6-phosphate receptor	IGF2R	25	25	25	By MS/MS	By MS/MS	By MS/MS	By MS/MS	By MS/MS	By MS/MS	By MS/MS	By MS/MS	By MS/MS	By MS/MS	By MS/MS	By MS/MS	14,2	274,37	0	184,02
O95163	O95163	Elongator complex protein 1	IKBKAP	17	17	17	By MS/MS	By MS/MS	By MS/MS	By MS/MS	By MS/MS	By MS/MS	By MS/MS	By MS/MS	By MS/MS	By MS/MS	By MS/MS	By MS/MS	21,5	150,25	0	103,50
Q12905	Q12905	Interleukin enhancer-binding factor 2	ILF2	17	17	17	By MS/MS	By MS/MS	By MS/MS	By MS/MS	By MS/MS	By MS/MS	By MS/MS	By MS/MS	By MS/MS	By MS/MS			58,5	43,06	0	257,23
Q12906	Q12906	Interleukin enhancer-binding factor 3	ILF3	16	16	15	By MS/MS	By MS/MS	By MS/MS	By MS/MS	By MS/MS	By MS/MS	By MS/MS	By MS/MS	By MS/MS	By MS/MS	By MS/MS	By MS/MS	24,8	95,34	0	134,19
Q16891	Q16891	MICOS complex subunit MIC60	IMMT	19	19	19	By MS/MS	By MS/MS	By MS/MS	By MS/MS	By MS/MS	By MS/MS	By MS/MS	By MS/MS	By MS/MS	By MS/MS	By MS/MS	By MS/MS	35,5	83,68	0	323,31
P29218	P29218	Inositol monophosphatase 1	IMPA1	6	6	6	By MS/MS		By MS/MS	By MS/MS	By MS/MS	By MS/MS		By MS/MS			By MS/MS	By MS/MS	30,3	30,19	0	19,92
P12268	P12268	Inosine-5-monophosphate dehydrogenase 2	IMPDH2	22	22	22	By MS/MS	By MS/MS	By MS/MS	By MS/MS	By MS/MS	By MS/MS	By MS/MS	By MS/MS			By MS/MS	By MS/MS	53,1	55,80	0	323,31
Q9UI26	Q9UI26	Importin-11	IPO11	20	20	20	By MS/MS	By MS/MS	By MS/MS	By MS/MS	By MS/MS	By MS/MS	By MS/MS	By MS/MS	By MS/MS	By MS/MS	By MS/MS	By MS/MS	32,0	112,53	0	212,41
Q8TEX9	Q8TEX9	Importin-4	IPO4	24	24	24	By MS/MS	By MS/MS	By MS/MS	By MS/MS	By MS/MS	By MS/MS	By MS/MS	By MS/MS	By MS/MS	By MS/MS	By MS/MS	By MS/MS	32,1	118,71	0	215,67
O00410;O60518	O00410	Importin-5	IPO5	36	36	36	By MS/MS	By MS/MS	By MS/MS	By MS/MS	By MS/MS	By MS/MS	By MS/MS	By MS/MS	By MS/MS	By MS/MS	By MS/MS	By MS/MS	46,9	123,63	0	323,31
O95373	O95373	Importin-7	IPO7	30	30	29	By MS/MS	By MS/MS	By MS/MS	By MS/MS	By MS/MS	By MS/MS	By MS/MS	By MS/MS	By MS/MS	By MS/MS	By MS/MS	By MS/MS	37,1	119,52	0	323,31
Q96P70	Q96P70	Importin-9	IPO9	14	14	14	By MS/MS	By MS/MS	By MS/MS	By MS/MS	By MS/MS	By MS/MS	By MS/MS	By MS/MS	By MS/MS	By MS/MS	By MS/MS	By MS/MS	22,0	115,96	0	252,32
P46940	P46940	Ras GTPase-activating-like protein IQGAP1	IQGAP1	48	48	47	By MS/MS	By MS/MS	By MS/MS	By MS/MS	By MS/MS	By MS/MS	By MS/MS	By MS/MS	By MS/MS	By MS/MS	By MS/MS	By MS/MS	41,5	189,25	0	323,31
Q96CN7	Q96CN7	Isochorismatase domain-containing protein 1	ISOC1	9	9	9	By MS/MS	By MS/MS	By MS/MS	By MS/MS	By MS/MS	By MS/MS	By MS/MS	By MS/MS	By MS/MS	By MS/MS	By MS/MS	By MS/MS	44,6	32,24	0	38,81
Q96AB3	Q96AB3	Isochorismatase domain-containing protein 2, mitochondrial	ISOC2	6	6	6	By MS/MS	By MS/MS	By MS/MS	By MS/MS	By MS/MS	By MS/MS	By MS/MS	By MS/MS	By MS/MS	By MS/MS	By MS/MS	By MS/MS	37,1	22,34	0	49,62
Q9NPH2	Q9NPH2	Inositol-3-phosphate synthase 1	ISYNA1	13	13	13	By MS/MS	By MS/MS	By MS/MS	By MS/MS	By MS/MS	By MS/MS	By MS/MS	By MS/MS			By MS/MS	By MS/MS	35,7	61,07	0	136,72
P05556	P05556	Integrin beta-1	ITGB1	4	4	4	By MS/MS	By MS/MS	By MS/MS	By MS/MS		By MS/MS					By MS/MS	By MS/MS	8,3	88,41	0	21,10

Q9BY32	Q9BY32	Inosine triphosphate pyrophosphatase	ITPA	6	6	6	By MS/MS	By MS/MS	By MS/MS	By MS/MS	By MS/MS	By MS/MS	By MS/MS	By MS/MS	By MS/MS	By MS/MS	By MS/MS	By MS/MS	45,4	21,45	0	309,88
P14923;P35222	P14923	Junction plakoglobin	JUP	16	16	16	By MS/MS	By MS/MS	By MS/MS	By MS/MS		By MS/MS		By MS/MS	By MS/MS		By MS/MS		36,8	81,74	0	97,21
Q15046	Q15046	Lysine--tRNA ligase	KARS	22	22	22	By MS/MS	By MS/MS	By MS/MS	By MS/MS	By MS/MS	By MS/MS	By MS/MS	By MS/MS	By MS/MS	By MS/MS	By MS/MS	By MS/MS	43,7	68,05	0	284,17
O60341	O60341	Lysine-specific histone demethylase 1A	KDM1A	14	14	14	By MS/MS	By MS/MS	By MS/MS	By MS/MS	By MS/MS	By MS/MS	By MS/MS	By MS/MS	By MS/MS	By MS/MS	By MS/MS	By MS/MS	24,9	92,90	0	69,93
Q7LBC6	Q7LBC6	Lysine-specific demethylase 3B	KDM3B	10	10	9		By MS/MS	By MS/MS	By MS/MS	By MS/MS	By MS/MS	By MS/MS	By MS/MS	By MS/MS	By MS/MS	By MS/MS	By MS/MS	9,9	191,58	0	10,61
Q07666;Q5VWX1	Q07666	KH domain-containing, RNA-binding, signal transduction-associated protein 1	KHDRBS1	6	6	6	By MS/MS	By MS/MS	By MS/MS	By MS/MS	By MS/MS	By MS/MS	By MS/MS	By MS/MS	By MS/MS	By MS/MS	By MS/MS	By MS/MS	22,1	48,23	0	22,67
Q92945	Q92945	Far upstream element-binding protein 2	KHSRP	21	21	21	By MS/MS	By MS/MS	By MS/MS	By MS/MS	By MS/MS	By MS/MS	By MS/MS	By MS/MS	By MS/MS	By MS/MS	By MS/MS	By MS/MS	44,0	73,11	0	176,33
Q15397	Q15397	Pumilio domain-containing protein KIAA0020	KIAA0020	5	5	5	By MS/MS	By MS/MS		By MS/MS	By MS/MS	By MS/MS					By MS/MS	By MS/MS	11,9	73,58	0	14,53
Q99661;Q8N4N8	Q99661	Kinesin-like protein KIF2C	KIF2C	4	4	4		By MS/MS		By MS/MS		By MS/MS	By MS/MS				By MS/MS		6,8	81,31	0	7,59
P33176	P33176	Kinesin-1 heavy chain	KIF5B	32	32	27	By MS/MS	By MS/MS	By MS/MS	By MS/MS	By MS/MS	By MS/MS	By MS/MS	By MS/MS	By MS/MS	By MS/MS	By MS/MS	By MS/MS	45,9	109,68	0	183,47
P52294	P52294	Importin subunit alpha-5;Importin subunit alpha-5, N-terminally processed	KPNA1	6	3	3	By MS/MS	By MS/MS	By matching		By matchin	By MS/MS		By MS/MS			By matchin	By matchin	16,5	60,22	0	15,50
P52292	P52292	Importin subunit alpha-1	KPNA2	22	22	22	By MS/MS	By MS/MS	By MS/MS	By MS/MS	By MS/MS	By MS/MS	By MS/MS	By MS/MS	By MS/MS	By MS/MS	By MS/MS	By MS/MS	68,1	57,86	0	323,31
O00505	O00505	Importin subunit alpha-4	KPNA3	12	12	7	By MS/MS	By MS/MS	By MS/MS	By MS/MS	By MS/MS	By MS/MS		By MS/MS			By MS/MS	By MS/MS	33,2	57,81	0	56,03
O00629	O00629	Importin subunit alpha-3	KPNA4	11	6	6	By MS/MS	By MS/MS	By MS/MS	By MS/MS	By MS/MS	By MS/MS		By MS/MS			By MS/MS	By MS/MS	31,9	57,89	0	323,31
O60684;O15131	O60684	Importin subunit alpha-7	KPNA6	10	10	7	By MS/MS	By MS/MS	By MS/MS	By MS/MS	By MS/MS	By MS/MS					By MS/MS	By MS/MS	33,8	60,03	0	228,01
Q14974	Q14974	Importin subunit beta-1	KPNB1	36	36	36	By MS/MS	By MS/MS	By MS/MS	By MS/MS	By MS/MS	By MS/MS	By MS/MS	By MS/MS	By MS/MS	By MS/MS	By MS/MS	By MS/MS	54,8	97,17	0	323,31
P05783;CON__H-INV:HIT000015463	P05783	Keratin, type I cytoskeletal 18	KRT18	20	18	13	By MS/MS	By MS/MS	By MS/MS	By MS/MS	By MS/MS	By MS/MS	By MS/MS	By MS/MS	By MS/MS	By MS/MS	By MS/MS	By MS/MS	58,8	48,06	0	323,31
Q86UP2	Q86UP2	Kinectin	KTN1	8	8	8	By MS/MS	By MS/MS	By MS/MS	By MS/MS	By MS/MS	By MS/MS				By MS/MS	By MS/MS	By MS/MS	8,2	156,27	0	39,57
Q53H82	Q53H82	Beta-lactamase-like protein 2	LACTB2	4	4	4	By MS/MS	By MS/MS	By MS/MS	By MS/MS	By MS/MS	By MS/MS			By MS/MS	By MS/MS	By MS/MS	By MS/MS	24,0	32,81	0	10,24
P11279	P11279	Lysosome-associated membrane glycoprotein 1	LAMP1	4	4	4	By MS/MS	By MS/MS	By MS/MS	By MS/MS	By MS/MS	By MS/MS						By MS/MS	9,1	44,88	0	16,14
Q6IAA8	Q6IAA8	Ragulator complex protein LAMTOR1	LAMTOR1	7	7	7	By MS/MS	By MS/MS	By MS/MS	By MS/MS	By MS/MS	By MS/MS			By MS/MS	By MS/MS			55,9	17,75	0	57,62
Q9Y2Q5	Q9Y2Q5	Ragulator complex protein LAMTOR2	LAMTOR2	7	7	7	By MS/MS	By MS/MS	By MS/MS	By MS/MS	By MS/MS	By MS/MS	By MS/MS	By MS/MS	By MS/MS	By MS/MS	By MS/MS	By MS/MS	90,4	13,51	0	97,12
Q9UHA4	Q9UHA4	Ragulator complex protein LAMTOR3	LAMTOR3	4	4	4	By MS/MS	By MS/MS			By MS/MS		By MS/MS		By MS/MS		By MS/MS		49,2	13,62	0	7,99
P28838	P28838	Cytosol aminopeptidase	LAP3	22	22	22	By MS/MS	By MS/MS	By MS/MS	By MS/MS	By MS/MS	By MS/MS	By MS/MS	By MS/MS			By MS/MS	By MS/MS	56,8	56,17	0	297,66
Q9P2J5	Q9P2J5	Leucine--tRNA ligase, cytoplasmic	LARS	42	42	42	By MS/MS	By MS/MS	By MS/MS	By MS/MS	By MS/MS	By MS/MS	By MS/MS	By MS/MS	By MS/MS	By MS/MS	By MS/MS	By MS/MS	45,5	134,46	0	323,31
Q9Y4W2	Q9Y4W2	Ribosomal biogenesis protein LAS1L	LAS1L	4	4	4		By MS/MS	By MS/MS		By MS/MS		By MS/MS	By MS/MS	By MS/MS	By MS/MS			9,7	83,06	0	8,33
Q14739	Q14739	Lamin-B receptor	LBR	9	9	9	By MS/MS	By MS/MS	By MS/MS	By MS/MS	By MS/MS	By MS/MS	By MS/MS	By MS/MS	By MS/MS	By MS/MS	By MS/MS	By MS/MS	19,2	70,70	0	103,21
P13796;P13797	P13796	Plastin-2	LCP1	21	21	21	By MS/MS	By MS/MS	By MS/MS	By MS/MS	By MS/MS	By MS/MS			By MS/MS		By MS/MS	By MS/MS	51,7	70,29	0	240,12
P00338	P00338	L-lactate dehydrogenase A chain	LDHA	31	31	30	By MS/MS	By MS/MS	By MS/MS	By MS/MS	By MS/MS	By MS/MS	By MS/MS	By MS/MS	By MS/MS	By MS/MS	By MS/MS	By MS/MS	75,6	36,69	0	323,31
P07195;Q6ZMR3;P07864	P07195	L-lactate dehydrogenase B chain	LDHB	22	21	21	By MS/MS	By MS/MS	By MS/MS	By MS/MS	By MS/MS	By MS/MS	By MS/MS	By MS/MS	By MS/MS	By MS/MS	By MS/MS	By MS/MS	65,3	36,64	0	323,31
O95202	O95202	LETM1 and EF-hand domain-containing protein 1, mitochondrial	LETM1	9	9	9	By MS/MS	By MS/MS	By MS/MS	By MS/MS	By MS/MS	By MS/MS		By MS/MS			By MS/MS	By MS/MS	18,0	83,35	0	30,15
P09382	P09382	Galectin-1	LGALS1	7	7	7	By MS/MS	By MS/MS	By MS/MS	By MS/MS	By MS/MS	By MS/MS	By MS/MS	By MS/MS	By MS/MS	By MS/MS	By MS/MS	By MS/MS	60,0	14,72	0	129,13
P18858	P18858	DNA ligase 1	LIG1	8	8	8	By MS/MS	By MS/MS	By MS/MS	By MS/MS		By MS/MS					By MS/MS	By MS/MS	11,6	101,73	0	11,60
P49257	P49257	Protein ERGIC-53	LMAN1	6	6	6	By MS/MS	By MS/MS	By MS/MS	By MS/MS	By MS/MS	By MS/MS	By MS/MS	By MS/MS			By MS/MS	By MS/MS	17,5	57,55	0	34,71
Q12907	Q12907	Vesicular integral-membrane protein VIP36	LMAN2	7	7	7	By MS/MS	By MS/MS	By MS/MS	By MS/MS	By MS/MS	By MS/MS	By MS/MS	By MS/MS	By MS/MS	By MS/MS			26,4	40,23	0	60,94
P02545	P02545	Prelamin-A/C;Lamin-A/C	LMNA	12	12	12	By MS/MS		By MS/MS	By MS/MS	By MS/MS	By MS/MS	By MS/MS	By MS/MS	By MS/MS		By MS/MS	By MS/MS	22,3	74,14	0	97,89
P20700	P20700	Lamin-B1	LMNB1	13	13	12	By MS/MS	By MS/MS		By MS/MS	By MS/MS	By MS/MS	By MS/MS	By MS/MS	By MS/MS	By MS/MS	By MS/MS	By MS/MS	30,4	66,41	0	202,20
P36776	P36776	Lon protease homolog, mitochondrial	LONP1	28	28	28	By MS/MS	By MS/MS	By MS/MS	By MS/MS	By MS/MS	By MS/MS	By MS/MS	By MS/MS	By MS/MS	By MS/MS	By MS/MS	By MS/MS	35,8	106,49	0	171,14
P50851;Q8NFP9	P50851	Lipopolysaccharide-responsive and beige-like anchor protein	LRBA	12	12	12	By MS/MS	By MS/MS	By MS/MS	By MS/MS	By MS/MS	By MS/MS		By MS/MS	By MS/MS	By MS/MS			6,4	319,10	0	43,63
P42704	P42704	Leucine-rich PPR motif-containing protein, mitochondrial	LRPPRC	90	90	90	By MS/MS	By MS/MS	By MS/MS	By MS/MS	By MS/MS	By MS/MS	By MS/MS	By MS/MS	By MS/MS	By MS/MS	By MS/MS	By MS/MS	69,0	157,90	0	323,31
Q8N1G4	Q8N1G4	Leucine-rich repeat-containing protein 47	LRRC47	11	11	11	By MS/MS	By MS/MS	By MS/MS	By MS/MS	By MS/MS	By MS/MS	By MS/MS	By MS/MS	By MS/MS	By MS/MS	By MS/MS	By MS/MS	28,8	63,47	0	137,94
Q8N9N7	Q8N9N7	Leucine-rich repeat-containing protein 57	LRRC57	4	4	4				By MS/MS		By MS/MS						By MS/MS	29,7	26,75	0	6,28
Q96AG4	Q96AG4	Leucine-rich repeat-containing protein 59	LRRC59	15	15	15	By MS/MS	By MS/MS	By MS/MS	By MS/MS	By MS/MS	By MS/MS	By MS/MS	By MS/MS	By MS/MS	By MS/MS	By MS/MS	By MS/MS	47,6	34,93	0	323,31
Q3MHD2	Q3MHD2	Protein LSM12 homolog	LSM12	4	4	4	By MS/MS	By MS/MS	By MS/MS	By MS/MS	By MS/MS	By MS/MS	By MS/MS	By MS/MS	By MS/MS	By MS/MS	By MS/MS	By MS/MS	34,9	21,70	0	22,82
Q9Y333	Q9Y333	U6 snRNA-associated Sm-like protein LSm2	LSM2	7	7	7	By MS/MS	By MS/MS	By MS/MS	By MS/MS	By MS/MS	By MS/MS	By MS/MS	By MS/MS	By MS/MS	By MS/MS	By MS/MS	By MS/MS	82,1	10,83	0	105,64
Q9Y4Z0	Q9Y4Z0	U6 snRNA-associated Sm-like protein LSm4	LSM4	5	5	5	By MS/MS	By MS/MS	By MS/MS	By MS/MS	By MS/MS	By MS/MS	By MS/MS	By MS/MS			By MS/MS	By MS/MS	30,9	15,35	0	9,38
O95777	O95777	U6 snRNA-associated Sm-like protein LSm8	LSM8	5	5	5	By MS/MS	By MS/MS	By MS/MS	By MS/MS	By MS/MS	By MS/MS	By MS/MS	By MS/MS	By MS/MS	By MS/MS	By MS/MS	By MS/MS	82,3	10,40	0	134,74
P48449	P48449	Lanosterol synthase	LSS	7	7	7	By MS/MS	By MS/MS	By MS/MS	By MS/MS	By MS/MS	By MS/MS	By MS/MS	By MS/MS	By MS/MS	By MS/MS		By MS/MS	12,4	83,31	0	8,52
P09960	P09960	Leukotriene A-4 hydrolase	LTA4H	6	6	6	By MS/MS	By MS/MS	By MS/MS	By MS/MS	By MS/MS		By MS/MS	By MS/MS	By MS/MS	By MS/MS		By MS/MS	14,2	69,28	0	53,59
Q9Y383;Q9NQ29	Q9Y383	Putative RNA-binding protein Luc7-like 2	LUC7L2	12	12	12	By MS/MS	By MS/MS	By MS/MS	By MS/MS	By MS/MS	By MS/MS	By MS/MS	By MS/MS	By MS/MS	By MS/MS	By MS/MS	By MS/MS	33,9	46,51	0	155,21
O95232	O95232	Luc7-like protein 3	LUC7L3	8	8	8	By MS/MS	By MS/MS		By MS/MS	By MS/MS	By MS/MS	By MS/MS	By MS/MS			By MS/MS	By MS/MS	30,1	51,47	0	25,78
Q9NX58	Q9NX58	Cell growth-regulating nucleolar protein	LYAR	8	8	8		By MS/MS	By MS/MS	By MS/MS	By MS/MS	By MS/MS	By MS/MS	By MS/MS	By MS/MS	By MS/MS	By MS/MS	By MS/MS	28,0	43,61	0	19,90
P07948	P07948	Tyrosine-protein kinase Lyn	LYN	4	3	3		By MS/MS		By MS/MS		By MS/MS					By MS/MS		12,7	58,57	0,000348	2,78
O75608	O75608	Acyl-protein thioesterase 1	LYPLA1	8	8	8	By MS/MS	By MS/MS	By MS/MS	By MS/MS	By MS/MS	By MS/MS	By MS/MS	By MS/MS	By MS/MS	By MS/MS	By MS/MS	By MS/MS	55,2	24,67	0	35,56
Q5VWZ2	Q5VWZ2	Lysophospholipase-like protein 1	LYPLAL1	6	6	6	By MS/MS	By MS/MS	By MS/MS	By MS/MS	By MS/MS	By MS/MS			By MS/MS	By MS/MS	By MS/MS		32,9	26,32	0	11,91
Q5U5X0	Q5U5X0	Complex III assembly factor LYRM7	LYRM7	4	4	4	By MS/MS	By MS/MS	By MS/MS		By MS/MS	By MS/MS	By MS/MS	By MS/MS	By MS/MS			By MS/MS	41,3	11,96	0	10,00
Q8WZA0	Q8WZA0	Protein LZIC	LZIC	7	7	7	By MS/MS	By MS/MS	By MS/MS	By MS/MS	By MS/MS	By MS/MS	By MS/MS	By MS/MS	By MS/MS	By MS/MS	By MS/MS	By MS/MS	39,5	21,49	0	134,12
Q9BQ69	Q9BQ69	O-acetyl-ADP-ribose deacetylase MACROD1	MACROD1	4	4	4	By MS/MS	By MS/MS	By MS/MS	By MS/MS	By MS/MS	By MS/MS		By MS/MS	By MS/MS		By MS/MS		20,9	35,51	0	12,15
Q13257	Q13257	Mitotic spindle assembly checkpoint protein MAD2A	MAD2L1	6	6	6	By MS/MS	By MS/MS	By MS/MS	By MS/MS	By MS/MS	By MS/MS	By MS/MS	By MS/MS		By MS/MS	By MS/MS	By MS/MS	34,6	23,51	0	69,09
P36507	P36507	Dual specificity mitogen-activated protein kinase kinase 2	MAP2K2	9	9	5	By MS/MS	By MS/MS	By MS/MS	By MS/MS	By MS/MS	By MS/MS					By MS/MS	By MS/MS	32,8	44,42	0	15,73
P28482;P27361;P31152;Q16659	P28482	Mitogen-activated protein kinase 1	MAPK1	18	18	18	By MS/MS	By MS/MS	By MS/MS	By MS/MS	By MS/MS	By MS/MS	By MS/MS	By MS/MS	By MS/MS	By MS/MS			61,1	41,39	0	59,73
Q16539	Q16539	Mitogen-activated protein kinase 14	MAPK14	8	8	8	By MS/MS	By MS/MS	By MS/MS	By MS/MS	By MS/MS	By MS/MS		By MS/MS	By MS/MS	By MS/MS			38,3	41,29	0	30,96
Q15691	Q15691	Microtubule-associated protein RP/EB family member 1	MAPRE1	20	20	19	By MS/MS	By MS/MS	By MS/MS	By MS/MS	By MS/MS	By MS/MS	By MS/MS	By MS/MS	By MS/MS	By MS/MS	By MS/MS	By MS/MS	82,8	30,00	0	323,31
Q9NX47	Q9NX47	E3 ubiquitin-protein ligase MARCH5	MARCH5	4	4	4	By MS/MS	By MS/MS	By MS/MS	By MS/MS	By MS/MS	By MS/MS				By MS/MS			22,7	31,23	0	10,00
P56192	P56192	Methionine--tRNA ligase, cytoplasmic	MARS	28	28	28	By MS/MS	By MS/MS	By MS/MS	By MS/MS	By MS/MS	By MS/MS	By MS/MS	By MS/MS	By MS/MS	By MS/MS	By MS/MS	By MS/MS	49,6	101,11	0	323,31
P31153;Q00266	P31153	S-adenosylmethionine synthase isoform type-2	MAT2A	9	9	9	By MS/MS	By MS/MS	By MS/MS	By MS/MS	By MS/MS	By MS/MS	By MS/MS	By MS/MS			By MS/MS	By MS/MS	28,4	43,66	0	39,21
Q9NZL9	Q9NZL9	Methionine adenosyltransferase 2 subunit beta	MAT2B	4	4	4	By MS/MS	By MS/MS	By MS/MS	By MS/MS	By MS/MS	By MS/MS	By MS/MS	By MS/MS	By MS/MS	By MS/MS			17,7	37,55	0	35,28
P43243	P43243	Matrin-3	MATR3	15	15	15	By MS/MS	By MS/MS	By MS/MS	By MS/MS	By MS/MS	By MS/MS	By MS/MS	By MS/MS	By MS/MS	By MS/MS	By MS/MS	By MS/MS	26,4	94,62	0	284,68

Q68D91	Q68D91	Metallo-beta-lactamase domain-containing protein 2	MBLAC2	4	4	4			By MS/MS	By MS/MS	By MS/MS		By MS/MS						20,4	31,37	0	8,45
Q9HCC0	Q9HCC0	Methylcrotonoyl-CoA carboxylase beta chain, mitochondrial	MCCC2	8	8	8	By MS/MS	By MS/MS	By MS/MS	By MS/MS	By MS/MS	By MS/MS					By MS/MS	By MS/MS	23,3	61,33	0	164,11
P49736	P49736	DNA replication licensing factor MCM2	MCM2	32	32	32	By MS/MS	By MS/MS	By MS/MS	By MS/MS	By MS/MS	By MS/MS	By MS/MS	By MS/MS	By MS/MS	By MS/MS	By MS/MS	By MS/MS	47,1	101,89	0	323,31
P25205	P25205	DNA replication licensing factor MCM3	MCM3	34	34	34	By MS/MS	By MS/MS	By MS/MS	By MS/MS	By MS/MS	By MS/MS	By MS/MS	By MS/MS	By MS/MS	By MS/MS	By MS/MS	By MS/MS	45,2	90,98	0	323,31
P33991	P33991	DNA replication licensing factor MCM4	MCM4	23	23	23	By MS/MS	By MS/MS	By MS/MS	By MS/MS	By MS/MS	By MS/MS	By MS/MS	By MS/MS	By MS/MS	By MS/MS	By MS/MS	By MS/MS	34,8	96,56	0	323,31
P33992	P33992	DNA replication licensing factor MCM5	MCM5	15	15	15	By MS/MS	By MS/MS	By MS/MS	By MS/MS	By MS/MS	By MS/MS	By MS/MS	By MS/MS	By MS/MS	By MS/MS	By MS/MS	By MS/MS	28,9	82,29	0	117,90
Q14566	Q14566	DNA replication licensing factor MCM6	MCM6	31	31	31	By MS/MS	By MS/MS	By MS/MS	By MS/MS	By MS/MS	By MS/MS	By MS/MS	By MS/MS	By MS/MS	By MS/MS	By MS/MS	By MS/MS	49,2	92,89	0	323,31
P33993	P33993	DNA replication licensing factor MCM7	MCM7	29	29	29	By MS/MS	By MS/MS	By MS/MS	By MS/MS	By MS/MS	By MS/MS	By MS/MS	By MS/MS	By MS/MS	By MS/MS	By MS/MS	By MS/MS	50,9	81,31	0	197,83
Q9ULC4	Q9ULC4	Malignant T-cell-amplified sequence 1	MCTS1	10	10	10	By MS/MS	By MS/MS	By MS/MS	By MS/MS	By MS/MS	By MS/MS	By MS/MS	By MS/MS	By MS/MS	By MS/MS	By MS/MS	By MS/MS	57,5	20,56	0	259,40
P40925	P40925	Malate dehydrogenase, cytoplasmic	MDH1	15	15	15	By MS/MS	By MS/MS	By MS/MS	By MS/MS	By MS/MS	By MS/MS	By MS/MS	By MS/MS	By MS/MS	By MS/MS	By MS/MS	By MS/MS	49,7	36,43	0	278,56
P40926	P40926	Malate dehydrogenase, mitochondrial	MDH2	23	23	23	By MS/MS	By MS/MS	By MS/MS	By MS/MS	By MS/MS	By MS/MS	By MS/MS	By MS/MS	By MS/MS	By MS/MS	By MS/MS	By MS/MS	66,6	35,50	0	323,31
Q9NU22	Q9NU22	Midasin	MDN1	12	12	12	By MS/MS	By MS/MS	By MS/MS	By MS/MS	By MS/MS	By MS/MS	By MS/MS		By MS/MS		By MS/MS		3,4	632,81	0	22,68
P48163	P48163	NADP-dependent malic enzyme	ME1	5	5	5	By MS/MS	By MS/MS	By MS/MS	By MS/MS	By MS/MS	By MS/MS						By MS/MS	17,7	64,15	0	22,20
P23368	P23368	NAD-dependent malic enzyme, mitochondrial	ME2	5	5	5				By MS/MS	By MS/MS	By MS/MS						By MS/MS	21,2	65,44	0	59,56
Q9Y316	Q9Y316	Protein MEMO1	MEMO1	10	10	10	By MS/MS	By MS/MS	By MS/MS	By MS/MS	By MS/MS	By MS/MS	By MS/MS	By MS/MS	By MS/MS	By MS/MS			45,8	33,73	0	99,85
P50579	P50579	Methionine aminopeptidase 2	METAP2	10	10	10	By MS/MS	By MS/MS	By MS/MS		By MS/MS	By MS/MS	By MS/MS	By MS/MS	By MS/MS	By MS/MS	By MS/MS	By MS/MS	31,2	52,89	0	74,07
O14880	O14880	Microsomal glutathione S-transferase 3	MGST3	5	5	5	By MS/MS	By MS/MS	By MS/MS	By MS/MS	By MS/MS	By MS/MS	By MS/MS	By MS/MS	By MS/MS	By MS/MS		By MS/MS	52,6	16,52	0	47,59
P14174	P14174	Macrophage migration inhibitory factor	MIF	4	4	4	By MS/MS	By MS/MS	By MS/MS	By MS/MS	By MS/MS	By MS/MS	By MS/MS	By MS/MS	By MS/MS	By MS/MS	By MS/MS	By MS/MS	36,5	12,48	0	316,21
Q14165	Q14165	Malectin	MLEC	8	8	8	By MS/MS	By MS/MS	By MS/MS	By MS/MS	By MS/MS	By MS/MS			By MS/MS	By MS/MS	By MS/MS	By MS/MS	34,9	32,23	0	29,51
Q9H8S9;Q7L9L4	Q9H8S9;Q7L9L4	MOB kinase activator 1A;MOB kinase activator 1B	MOB1A;MOB1B	5	5	5	By MS/MS	By MS/MS	By MS/MS	By MS/MS	By MS/MS	By MS/MS	By MS/MS	By MS/MS	By MS/MS	By MS/MS			23,6	25,08	0	14,09
Q13724	Q13724	Mannosyl-oligosaccharide glucosidase	MOGS	4	4	4	By MS/MS	By MS/MS	By MS/MS	By MS/MS	By MS/MS	By MS/MS	By MS/MS	By MS/MS	By MS/MS	By MS/MS	By MS/MS	By MS/MS	7,4	91,92	0	32,32
Q9Y5U8	Q9Y5U8	Mitochondrial pyruvate carrier 1	MPC1	4	4	4	By MS/MS	By MS/MS					By MS/MS			By MS/MS	By MS/MS		63,3	12,35	0	7,89
O95563	O95563	Mitochondrial pyruvate carrier 2	MPC2	6	6	6	By MS/MS	By MS/MS		By MS/MS	By MS/MS		By MS/MS		By MS/MS		By MS/MS	By MS/MS	53,5	14,28	0	14,65
Q00013	Q00013	55 kDa erythrocyte membrane protein	MPP1	13	13	13	By MS/MS	By MS/MS	By MS/MS	By MS/MS	By MS/MS	By MS/MS	By MS/MS	By MS/MS	By MS/MS	By MS/MS	By MS/MS	By MS/MS	43,1	52,30	0	223,17
P25325	P25325	3-mercaptopyruvate sulfurtransferase	MPST	4	4	4	By MS/MS	By MS/MS	By MS/MS	By MS/MS	By MS/MS	By MS/MS	By MS/MS	By MS/MS	By MS/MS	By MS/MS	By MS/MS	By MS/MS	26,9	33,18	0	65,29
P49959	P49959	Double-strand break repair protein MRE11A	MRE11A	7	7	7		By MS/MS		By MS/MS		By MS/MS	By MS/MS	By MS/MS	By MS/MS	By MS/MS	By MS/MS	By MS/MS	14,4	80,59	0	10,39
Q9BYD6	Q9BYD6	39S ribosomal protein L1, mitochondrial	MRPL1	7	7	7	By MS/MS	By MS/MS	By MS/MS	By MS/MS	By MS/MS	By MS/MS	By MS/MS	By MS/MS	By MS/MS	By MS/MS	By MS/MS	By MS/MS	35,4	36,91	0	49,93
Q9Y3B7	Q9Y3B7	39S ribosomal protein L11, mitochondrial	MRPL11	11	11	11	By MS/MS	By MS/MS	By MS/MS	By MS/MS	By MS/MS	By MS/MS	By MS/MS	By MS/MS	By MS/MS	By MS/MS	By MS/MS	By MS/MS	57,8	20,68	0	74,09
P52815	P52815	39S ribosomal protein L12, mitochondrial	MRPL12	7	7	7	By MS/MS	By MS/MS	By MS/MS	By MS/MS	By MS/MS	By MS/MS	By MS/MS	By MS/MS	By MS/MS	By MS/MS	By MS/MS	By MS/MS	52,0	21,35	0	85,98
Q9BYD1	Q9BYD1	39S ribosomal protein L13, mitochondrial	MRPL13	8	8	8	By MS/MS	By MS/MS	By MS/MS	By MS/MS	By MS/MS	By MS/MS			By MS/MS	By MS/MS	By MS/MS		52,8	20,69	0	13,42
Q6P1L8	Q6P1L8	39S ribosomal protein L14, mitochondrial	MRPL14	5	5	5	By MS/MS	By MS/MS	By MS/MS	By MS/MS	By MS/MS	By MS/MS		By MS/MS	By MS/MS	By MS/MS	By MS/MS		42,8	15,95	0	96,08
Q9P015	Q9P015	39S ribosomal protein L15, mitochondrial	MRPL15	11	11	11	By MS/MS	By MS/MS	By MS/MS	By MS/MS	By MS/MS	By MS/MS	By MS/MS	By MS/MS		By MS/MS	By MS/MS	By MS/MS	45,3	33,42	0	82,61
Q9NRX2	Q9NRX2	39S ribosomal protein L17, mitochondrial	MRPL17	7	7	7	By MS/MS	By MS/MS	By MS/MS	By MS/MS		By MS/MS			By MS/MS	By MS/MS			36,6	20,05	0	12,45
P49406	P49406	39S ribosomal protein L19, mitochondrial	MRPL19	4	4	4	By MS/MS	By MS/MS		By MS/MS		By MS/MS			By MS/MS	By MS/MS			14,4	33,54	0	5,90
Q9BYC9	Q9BYC9	39S ribosomal protein L20, mitochondrial	MRPL20	5	5	5	By MS/MS	By MS/MS	By MS/MS	By MS/MS	By MS/MS	By MS/MS		By MS/MS	By MS/MS		By MS/MS		34,9	17,44	0	6,97
Q9NWU5	Q9NWU5	39S ribosomal protein L22, mitochondrial	MRPL22	6	6	6	By MS/MS	By MS/MS	By MS/MS	By MS/MS	By MS/MS	By MS/MS	By MS/MS	By MS/MS	By MS/MS	By MS/MS	By MS/MS	By MS/MS	32,0	23,64	0	25,46
Q16540	Q16540	39S ribosomal protein L23, mitochondrial	MRPL23	4	4	4	By MS/MS	By MS/MS	By MS/MS	By MS/MS	By MS/MS	By MS/MS	By MS/MS			By MS/MS		By MS/MS	35,3	17,78	0	31,77
Q96A35	Q96A35	39S ribosomal protein L24, mitochondrial	MRPL24	6	6	6	By MS/MS	By MS/MS	By MS/MS	By MS/MS	By MS/MS			By MS/MS	By MS/MS	By MS/MS			38,9	24,92	0	8,63
Q9P0M9	Q9P0M9	39S ribosomal protein L27, mitochondrial	MRPL27	4	4	4	By MS/MS	By MS/MS	By MS/MS	By MS/MS	By MS/MS	By MS/MS						By MS/MS	35,1	16,07	0	19,00
Q13084	Q13084	39S ribosomal protein L28, mitochondrial	MRPL28	4	4	4		By MS/MS	By MS/MS	By MS/MS	By MS/MS	By MS/MS		By MS/MS	By MS/MS	By MS/MS	By MS/MS	By MS/MS	21,5	30,16	0	7,98
Q9BZE1	Q9BZE1	39S ribosomal protein L37, mitochondrial	MRPL37	6	6	6	By MS/MS	By MS/MS	By MS/MS	By MS/MS	By MS/MS	By MS/MS	By MS/MS	By MS/MS		By MS/MS	By MS/MS		23,2	48,12	0	16,80
Q9NQ50	Q9NQ50	39S ribosomal protein L40, mitochondrial	MRPL40	5	5	5	By MS/MS	By MS/MS	By MS/MS	By MS/MS	By MS/MS	By MS/MS	By MS/MS	By MS/MS	By MS/MS	By MS/MS	By MS/MS	By MS/MS	34,5	24,49	0	11,21
Q8IXM3	Q8IXM3	39S ribosomal protein L41, mitochondrial	MRPL41	5	5	5	By MS/MS	By MS/MS	By MS/MS	By MS/MS	By MS/MS	By MS/MS		By MS/MS		By MS/MS	By MS/MS	By MS/MS	33,6	15,38	0	9,57
Q8N983	Q8N983	39S ribosomal protein L43, mitochondrial	MRPL43	5	5	5	By MS/MS	By MS/MS	By MS/MS	By MS/MS	By MS/MS	By MS/MS	By MS/MS				By MS/MS	By MS/MS	30,7	23,43	0	19,67
Q9H9J2	Q9H9J2	39S ribosomal protein L44, mitochondrial	MRPL44	5	5	5	By MS/MS	By MS/MS	By MS/MS	By MS/MS	By MS/MS	By MS/MS	By MS/MS	By MS/MS	By MS/MS	By MS/MS	By MS/MS	By MS/MS	23,8	37,54	0	78,58
Q9HD33	Q9HD33	39S ribosomal protein L47, mitochondrial	MRPL47	7	7	7	By MS/MS	By MS/MS	By MS/MS	By MS/MS	By MS/MS	By MS/MS	By MS/MS	By MS/MS	By MS/MS	By MS/MS	By MS/MS	By MS/MS	24,0	29,45	0	20,49
Q8N5N7	Q8N5N7	39S ribosomal protein L50, mitochondrial	MRPL50	4	4	4	By MS/MS	By MS/MS	By MS/MS	By MS/MS	By MS/MS	By MS/MS	By MS/MS				By MS/MS	By MS/MS	24,7	18,33	0	9,75
Q96EL3	Q96EL3	39S ribosomal protein L53, mitochondrial	MRPL53	4	4	4	By MS/MS	By MS/MS	By MS/MS	By MS/MS	By MS/MS	By MS/MS	By MS/MS	By MS/MS	By MS/MS	By MS/MS			37,5	12,11	0	20,61
Q9BYD2	Q9BYD2	39S ribosomal protein L9, mitochondrial	MRPL9	4	4	4	By MS/MS	By MS/MS	By MS/MS	By MS/MS	By MS/MS	By MS/MS	By MS/MS	By MS/MS	By MS/MS		By MS/MS	By MS/MS	21,0	30,24	0	112,15
P82664	P82664	28S ribosomal protein S10, mitochondrial	MRPS10	7	7	7	By MS/MS	By MS/MS		By MS/MS					By MS/MS	By MS/MS			30,8	23,00	0	44,33
Q9Y2R5	Q9Y2R5	28S ribosomal protein S17, mitochondrial	MRPS17	4	4	4	By MS/MS	By MS/MS	By MS/MS	By MS/MS	By MS/MS	By MS/MS		By MS/MS		By MS/MS		By MS/MS	51,5	14,50	0	45,98
Q9Y676	Q9Y676	28S ribosomal protein S18b, mitochondrial	MRPS18B	5	5	5	By MS/MS	By MS/MS	By MS/MS	By MS/MS	By MS/MS	By MS/MS	By MS/MS		By MS/MS	By MS/MS	By MS/MS	By MS/MS	32,9	29,40	0	27,92
P82650	P82650	28S ribosomal protein S22, mitochondrial	MRPS22	13	13	13	By MS/MS	By MS/MS	By MS/MS	By MS/MS	By MS/MS	By MS/MS	By MS/MS	By MS/MS	By MS/MS	By MS/MS			41,4	41,28	0	33,34
P82663	P82663	28S ribosomal protein S25, mitochondrial	MRPS25	7	7	7	By MS/MS	By MS/MS	By MS/MS	By MS/MS	By MS/MS	By MS/MS	By MS/MS	By MS/MS	By MS/MS	By MS/MS			41,6	20,12	0	26,68
Q92552	Q92552	28S ribosomal protein S27, mitochondrial	MRPS27	7	7	7	By MS/MS	By MS/MS	By MS/MS	By MS/MS	By MS/MS	By MS/MS	By MS/MS	By MS/MS	By MS/MS	By MS/MS			21,7	47,61	0	34,85
Q9Y2Q9	Q9Y2Q9	28S ribosomal protein S28, mitochondrial	MRPS28	5	5	5	By MS/MS	By MS/MS	By MS/MS	By MS/MS	By MS/MS	By MS/MS		By MS/MS			By MS/MS	By MS/MS	29,9	20,84	0	24,39
Q92665	Q92665	28S ribosomal protein S31, mitochondrial	MRPS31	4	4	4	By MS/MS		By MS/MS		By MS/MS		By MS/MS	By MS/MS	By MS/MS	By MS/MS			15,2	45,32	0	51,18
P82930	P82930	28S ribosomal protein S34, mitochondrial	MRPS34	5	5	5	By MS/MS	By MS/MS	By MS/MS	By MS/MS	By MS/MS	By MS/MS	By MS/MS	By MS/MS	By MS/MS			By MS/MS	28,0	25,65	0	8,28
P82673	P82673	28S ribosomal protein S35, mitochondrial	MRPS35	6	6	6		By MS/MS		By MS/MS	By MS/MS	By MS/MS		By MS/MS	By MS/MS	By MS/MS			25,1	36,84	0	13,43
P82675	P82675	28S ribosomal protein S5, mitochondrial	MRPS5	5	5	5	By MS/MS	By MS/MS	By MS/MS	By MS/MS	By MS/MS	By MS/MS	By MS/MS	By MS/MS	By MS/MS	By MS/MS			17,0	48,01	0	50,78
P82932	P82932	28S ribosomal protein S6, mitochondrial	MRPS6	7	7	7	By MS/MS	By MS/MS				By MS/MS		By MS/MS	By MS/MS	By MS/MS	By MS/MS	By MS/MS	39,2	14,23	0	9,68
Q9Y2R9	Q9Y2R9	28S ribosomal protein S7, mitochondrial	MRPS7	12	12	12	By MS/MS	By MS/MS	By MS/MS	By MS/MS	By MS/MS	By MS/MS	By MS/MS	By MS/MS	By MS/MS	By MS/MS		By MS/MS	48,8	28,13	0	32,13
Q9UKD2	Q9UKD2	mRNA turnover protein 4 homolog	MRTO4	7	7	7	By MS/MS	By MS/MS	By MS/MS	By MS/MS	By MS/MS	By MS/MS		By MS/MS	By MS/MS	By MS/MS	By MS/MS		39,3	27,56	0	20,12
P43246	P43246	DNA mismatch repair protein Msh2	MSH2	12	12	12	By MS/MS	By MS/MS	By MS/MS	By MS/MS	By MS/MS	By MS/MS	By MS/MS	By MS/MS	By MS/MS	By MS/MS	By MS/MS	By MS/MS	20,4	104,74	0	235,32
P52701	P52701	DNA mismatch repair protein Msh6	MSH6	19	19	19	By MS/MS	By MS/MS	By MS/MS	By MS/MS	By MS/MS	By MS/MS	By MS/MS	By MS/MS	By MS/MS	By MS/MS	By MS/MS	By MS/MS	21,0	152,78	0	92,60
P26038	P26038	Moesin	MSN	34	34	25	By MS/MS	By MS/MS	By MS/MS	By MS/MS	By MS/MS	By MS/MS	By MS/MS	By MS/MS	By MS/MS	By MS/MS	By MS/MS	By MS/MS	61,2	67,82	0	323,31
P00403	P00403	Cytochrome c oxidase subunit 2	MT-CO2	6	6	6	By MS/MS	By MS/MS	By MS/MS	By MS/MS	By MS/MS	By MS/MS	By MS/MS	By MS/MS	By MS/MS	By MS/MS	By MS/MS	By MS/MS	32,2	25,57	0	180,49
O94776	O94776	Metastasis-associated protein MTA2	MTA2	10	10	10	By MS/MS	By MS/MS	By MS/MS		By MS/MS	By MS/MS	By MS/MS	By MS/MS		By MS/MS	By MS/MS	By MS/MS	20,2	75,02	0	162,57
Q9Y6C9	Q9Y6C9	Mitochondrial carrier homolog 2	MTCH2	13	13	13	By MS/MS	By MS/MS	By MS/MS	By MS/MS	By MS/MS	By MS/MS	By MS/MS	By MS/MS	By MS/MS	By MS/MS	By MS/MS	By MS/MS	56,1	33,33	0	323,31
P11586	P11586	C-1-tetrahydrofolate synthase, cytoplasmic;Methylenetetrahydrofolate dehydrogenase;Methenyltetrahydrofolate cyclohydrolase;Formyltetrahydrofolate synthetase;C-1-tetrahydrofolate synthase, cytoplasmic, N-terminally processed	MTHFD1	26	26	26	By MS/MS	By MS/MS	By MS/MS	By MS/MS	By MS/MS	By MS/MS	By MS/MS	By MS/MS	By MS/MS	By MS/MS	By MS/MS	By MS/MS	39,1	101,56	0	204,77

Q6UB35	Q6UB35	Monofunctional C1-tetrahydrofolate synthase, mitochondrial	MTHFD1L	13	13	13	By MS/MS	By MS/MS	By MS/MS	By MS/MS	By MS/MS	By MS/MS	By MS/MS	By MS/MS		By MS/MS	By MS/MS	By MS/MS	18,4	105,79	0	95,70
P13995	P13995	Bifunctional methylenetetrahydrofolate dehydrogenase/cyclohydrolase, mitochondrial;NAD-dependent methylenetetrahydrofolate dehydrogenase;Methenyltetrahydrofolate cyclohydrolase	MTHFD2	7	7	7	By MS/MS	By MS/MS	By MS/MS	By MS/MS	By MS/MS	By MS/MS	By MS/MS	By MS/MS	By MS/MS	By MS/MS	By MS/MS	By MS/MS	40,3	37,90	0	53,16
P42345	P42345	Serine/threonine-protein kinase mTOR	MTOR	14	14	14	By MS/MS	By MS/MS	By MS/MS	By MS/MS	By MS/MS	By MS/MS		By MS/MS				By MS/MS	9,8	288,89	0	40,76
P58546	P58546	Myotrophin	MTPN	8	8	8	By MS/MS	By MS/MS	By MS/MS	By MS/MS	By MS/MS	By MS/MS	By MS/MS	By MS/MS	By MS/MS	By MS/MS	By MS/MS	By MS/MS	68,6	12,90	0	323,31
Q13505	Q13505	Metaxin-1	MTX1	7	7	7	By MS/MS	By MS/MS	By MS/MS	By MS/MS	By MS/MS	By MS/MS	By MS/MS	By MS/MS	By MS/MS	By MS/MS	By MS/MS	By MS/MS	18,9	51,48	0	68,75
O75431	O75431	Metaxin-2	MTX2	5	5	5	By MS/MS	By MS/MS	By MS/MS	By MS/MS	By MS/MS	By MS/MS	By MS/MS	By MS/MS	By MS/MS	By MS/MS	By MS/MS	By MS/MS	33,8	29,76	0	51,55
P53602	P53602	Diphosphomevalonate decarboxylase	MVD	7	7	7	By MS/MS	By MS/MS	By MS/MS	By MS/MS	By MS/MS	By MS/MS	By MS/MS	By MS/MS	By MS/MS	By MS/MS			39,8	43,40	0	33,27
Q9BQG0	Q9BQG0	Myb-binding protein 1A	MYBBP1A	24	24	24	By MS/MS	By MS/MS	By MS/MS	By MS/MS	By MS/MS	By MS/MS	By MS/MS	By MS/MS	By MS/MS	By MS/MS	By MS/MS	By MS/MS	26,8	148,85	0	62,78
Q969H8	Q969H8	Myeloid-derived growth factor	MYDGF	6	6	6	By MS/MS	By MS/MS	By MS/MS	By MS/MS	By MS/MS	By MS/MS	By MS/MS		By MS/MS	By MS/MS	By MS/MS	By MS/MS	42,8	18,80	0	22,99
P35580	P35580	Myosin-10	MYH10	52	52	40	By MS/MS	By MS/MS	By MS/MS	By MS/MS	By MS/MS	By MS/MS	By MS/MS	By MS/MS	By MS/MS	By MS/MS	By MS/MS	By MS/MS	35,3	229,00	0	323,31
Q7Z406	Q7Z406	Myosin-14	MYH14	6	2	2	By MS/MS	By MS/MS	By matchin	By matchin	By MS/MS	By matchin	By matchin	By MS/MS	By MS/MS	By matchin	By matchin	By MS/MS	3,6	227,87	0	3,21
P35579	P35579	Myosin-9	MYH9	40	32	29	By MS/MS	By MS/MS	By MS/MS	By MS/MS	By MS/MS	By MS/MS	By MS/MS	By MS/MS	By MS/MS	By MS/MS	By MS/MS	By MS/MS	26,6	226,53	0	323,31
P19105;O14950;P24844	P19105;O14950	Myosin regulatory light chain 12A;Myosin regulatory light chain 12B	MYL12A;MYL12B	9	9	9	By MS/MS	By MS/MS	By MS/MS	By MS/MS	By MS/MS	By MS/MS	By MS/MS	By MS/MS	By MS/MS	By MS/MS	By MS/MS	By MS/MS	63,2	19,79	0	323,31
P12829;P08590	P12829	Myosin light chain 4	MYL4	11	11	11	By MS/MS	By MS/MS	By MS/MS	By MS/MS	By MS/MS	By MS/MS	By MS/MS	By MS/MS	By MS/MS	By MS/MS	By MS/MS	By MS/MS	65,5	21,56	0	39,02
P60660;P14649	P60660	Myosin light polypeptide 6	MYL6	8	8	8	By MS/MS	By MS/MS	By MS/MS	By MS/MS	By MS/MS	By MS/MS	By MS/MS	By MS/MS	By MS/MS	By MS/MS	By MS/MS	By MS/MS	55,6	16,93	0	38,58
P41227;Q9BSU3	P41227	N-alpha-acetyltransferase 10	NAA10	8	8	8	By MS/MS	By MS/MS	By MS/MS	By MS/MS	By MS/MS	By MS/MS	By MS/MS	By MS/MS	By MS/MS	By MS/MS	By MS/MS	By MS/MS	43,8	26,46	0	138,21
Q9BXJ9;Q6N069	Q9BXJ9	N-alpha-acetyltransferase 15, NatA auxiliary subunit	NAA15	24	24	24	By MS/MS	By MS/MS	By MS/MS	By MS/MS	By MS/MS	By MS/MS	By MS/MS	By MS/MS	By MS/MS	By MS/MS	By MS/MS	By MS/MS	39,6	101,27	0	124,47
Q14CX7	Q14CX7	N-alpha-acetyltransferase 25, NatB auxiliary subunit	NAA25	5	5	5	By MS/MS	By MS/MS	By MS/MS				By MS/MS	By MS/MS	By MS/MS	By MS/MS	By MS/MS	By MS/MS	5,6	112,29	0	6,48
Q9GZZ1	Q9GZZ1	N-alpha-acetyltransferase 50	NAA50	14	14	14	By MS/MS	By MS/MS	By MS/MS	By MS/MS	By MS/MS	By MS/MS	By MS/MS	By MS/MS	By MS/MS	By MS/MS	By MS/MS	By MS/MS	69,2	19,40	0	51,32
Q13765;E9PAV3	Q13765;E9PAV3	Nascent polypeptide-associated complex subunit alpha;Nascent polypeptide-associated complex subunit alpha, muscle-specific form	NACA	8	8	6	By MS/MS	By MS/MS	By MS/MS	By MS/MS	By MS/MS	By MS/MS	By MS/MS	By MS/MS	By MS/MS	By MS/MS	By MS/MS	By MS/MS	34,9	23,38	0	323,31
Q4G0N4	Q4G0N4	NAD kinase 2, mitochondrial	NADK2	8	8	8	By MS/MS	By MS/MS	By MS/MS	By MS/MS	By MS/MS	By MS/MS	By MS/MS	By MS/MS	By MS/MS	By MS/MS			25,3	49,43	0	158,85
Q13564	Q13564	NEDD8-activating enzyme E1 regulatory subunit	NAE1	6	6	6	By MS/MS	By MS/MS	By MS/MS	By MS/MS	By MS/MS	By MS/MS	By MS/MS	By MS/MS			By MS/MS	By MS/MS	21,7	60,25	0	82,17
P43490	P43490	Nicotinamide phosphoribosyltransferase	NAMPT	20	20	20	By MS/MS	By MS/MS	By MS/MS	By MS/MS	By MS/MS	By MS/MS		By MS/MS			By MS/MS	By MS/MS	60,5	55,52	0	168,63
Q9NR45	Q9NR45	Sialic acid synthase	NANS	9	9	9	By MS/MS	By MS/MS	By MS/MS	By MS/MS	By MS/MS	By MS/MS	By MS/MS	By MS/MS	By MS/MS	By MS/MS			47,4	40,31	0	80,78
P55209	P55209	Nucleosome assembly protein 1-like 1	NAP1L1	14	14	12	By MS/MS	By MS/MS	By MS/MS	By MS/MS	By MS/MS	By MS/MS	By MS/MS	By MS/MS	By MS/MS		By MS/MS	By MS/MS	53,5	45,37	0	256,84
Q99733	Q99733	Nucleosome assembly protein 1-like 4	NAP1L4	12	10	10	By MS/MS	By MS/MS	By MS/MS	By MS/MS	By MS/MS	By MS/MS	By MS/MS	By MS/MS			By MS/MS	By MS/MS	32,8	42,82	0	323,31
P54920;Q9H115	P54920	Alpha-soluble NSF attachment protein	NAPA	19	19	19	By MS/MS	By MS/MS	By MS/MS	By MS/MS	By MS/MS	By MS/MS		By MS/MS	By MS/MS	By MS/MS	By MS/MS	By MS/MS	79,7	33,23	0	241,01
O43776	O43776	Asparagine--tRNA ligase, cytoplasmic	NARS	21	21	21	By MS/MS	By MS/MS	By MS/MS	By MS/MS	By MS/MS	By MS/MS	By MS/MS	By MS/MS			By MS/MS	By MS/MS	42,2	62,94	0	235,64
P49321	P49321	Nuclear autoantigenic sperm protein	NASP	29	29	29	By MS/MS	By MS/MS	By MS/MS	By MS/MS	By MS/MS	By MS/MS	By MS/MS	By MS/MS	By MS/MS	By MS/MS	By MS/MS	By MS/MS	57,6	85,24	0	323,31
Q9H0A0	Q9H0A0	N-acetyltransferase 10	NAT10	18	18	18	By MS/MS	By MS/MS	By MS/MS	By MS/MS	By MS/MS	By MS/MS	By MS/MS	By MS/MS	By MS/MS	By MS/MS	By MS/MS	By MS/MS	25,2	115,73	0	97,94
Q15021	Q15021	Condensin complex subunit 1	NCAPD2	11	11	11	By MS/MS	By MS/MS	By MS/MS	By MS/MS	By MS/MS	By MS/MS	By MS/MS	By MS/MS	By MS/MS		By MS/MS	By MS/MS	14,2	157,18	0	83,31
Q09161	Q09161	Nuclear cap-binding protein subunit 1	NCBP1	12	12	12	By MS/MS	By MS/MS		By MS/MS	By MS/MS	By MS/MS	By MS/MS	By MS/MS	By MS/MS	By MS/MS	By MS/MS	By MS/MS	23,3	91,84	0	15,89
Q69YL0	Q69YL0	Uncharacterized protein NCBP2-AS2	NCBP2-AS2	4	4	4	By MS/MS				By MS/MS				By MS/MS	By MS/MS			60,6	10,89	0	9,13
P19338	P19338	Nucleolin	NCL	32	32	32	By MS/MS	By MS/MS	By MS/MS	By MS/MS	By MS/MS	By MS/MS	By MS/MS	By MS/MS	By MS/MS	By MS/MS	By MS/MS	By MS/MS	36,1	76,61	0	323,31
Q969V3	Q969V3	Nicalin	NCLN	5	5	5	By MS/MS	By MS/MS	By MS/MS	By MS/MS	By MS/MS	By MS/MS						By MS/MS	12,4	62,97	0	11,98
Q92542	Q92542	Nicastrin	NCSTN	5	5	5	By MS/MS	By MS/MS	By MS/MS	By MS/MS	By MS/MS	By MS/MS	By MS/MS				By MS/MS	By MS/MS	8,5	78,41	0	9,65
Q9UI09	Q9UI09	NADH dehydrogenase [ubiquinone] 1 alpha subcomplex subunit 12	NDUFA12	5	5	5	By MS/MS	By MS/MS	By MS/MS	By MS/MS	By MS/MS	By MS/MS	By MS/MS	By MS/MS	By MS/MS	By MS/MS	By MS/MS	By MS/MS	40,0	17,11	0	54,14
Q9P0J0	Q9P0J0	NADH dehydrogenase [ubiquinone] 1 alpha subcomplex subunit 13	NDUFA13	6	6	6	By MS/MS	By MS/MS	By MS/MS	By MS/MS	By MS/MS	By MS/MS	By MS/MS	By MS/MS		By MS/MS	By MS/MS	By MS/MS	36,8	16,70	0	13,81
O43678	O43678	NADH dehydrogenase [ubiquinone] 1 alpha subcomplex subunit 2	NDUFA2	5	5	5	By MS/MS	By MS/MS	By MS/MS		By MS/MS	By MS/MS		By MS/MS		By MS/MS		By MS/MS	56,6	10,92	0	30,41
Q16718	Q16718	NADH dehydrogenase [ubiquinone] 1 alpha subcomplex subunit 5	NDUFA5	6	6	6	By MS/MS	By MS/MS	By MS/MS	By MS/MS	By MS/MS		By MS/MS	By MS/MS	By MS/MS	By MS/MS	By MS/MS	By MS/MS	70,7	13,46	0	28,51
P51970	P51970	NADH dehydrogenase [ubiquinone] 1 alpha subcomplex subunit 8	NDUFA8	5	5	5	By MS/MS	By MS/MS		By MS/MS	By MS/MS	By MS/MS	By MS/MS	By MS/MS	By MS/MS	By MS/MS			35,5	20,11	0	16,96
Q16795	Q16795	NADH dehydrogenase [ubiquinone] 1 alpha subcomplex subunit 9, mitochondrial	NDUFA9	12	12	12	By MS/MS	By MS/MS	By MS/MS	By MS/MS	By MS/MS	By MS/MS	By MS/MS	By MS/MS		By MS/MS	By MS/MS	By MS/MS	37,1	42,51	0	323,31
Q8N183	Q8N183	Mimitin, mitochondrial	NDUFAF2	7	7	7	By MS/MS	By MS/MS	By MS/MS	By MS/MS	By MS/MS	By MS/MS	By MS/MS	By MS/MS	By MS/MS	By MS/MS	By MS/MS	By MS/MS	40,8	19,86	0	14,71
Q9BU61	Q9BU61	NADH dehydrogenase [ubiquinone] 1 alpha subcomplex assembly factor 3	NDUFAF3	4	4	4	By MS/MS	By MS/MS	By MS/MS	By MS/MS	By MS/MS	By MS/MS				By MS/MS	By MS/MS	By MS/MS	32,1	20,35	0	21,49
O96000	O96000	NADH dehydrogenase [ubiquinone] 1 beta subcomplex subunit 10	NDUFB10	12	12	12	By MS/MS	By MS/MS	By MS/MS	By MS/MS	By MS/MS	By MS/MS	By MS/MS	By MS/MS	By MS/MS	By MS/MS	By MS/MS	By MS/MS	54,7	20,78	0	28,24
O95139	O95139	NADH dehydrogenase [ubiquinone] 1 beta subcomplex subunit 6	NDUFB6	4	4	4	By MS/MS	By MS/MS	By MS/MS	By MS/MS	By MS/MS	By MS/MS			By MS/MS	By MS/MS		By MS/MS	39,8	15,49	0	15,39
Q9Y6M9	Q9Y6M9	NADH dehydrogenase [ubiquinone] 1 beta subcomplex subunit 9	NDUFB9	6	6	6	By MS/MS	By MS/MS	By MS/MS	By MS/MS	By MS/MS	By MS/MS	By MS/MS	By MS/MS	By MS/MS	By MS/MS	By MS/MS	By MS/MS	43,0	21,83	0	285,25
O95298;E9PQ53	O95298	NADH dehydrogenase [ubiquinone] 1 subunit C2	NDUFC2	5	5	5	By MS/MS	By MS/MS		By MS/MS		By MS/MS	By MS/MS			By MS/MS			31,9	14,19	0	4,20
P28331	P28331	NADH-ubiquinone oxidoreductase 75 kDa subunit, mitochondrial	NDUFS1	15	15	15	By MS/MS	By MS/MS	By MS/MS	By MS/MS	By MS/MS	By MS/MS	By MS/MS	By MS/MS	By MS/MS	By MS/MS	By MS/MS	By MS/MS	36,6	79,47	0	164,05
O75306	O75306	NADH dehydrogenase [ubiquinone] iron-sulfur protein 2, mitochondrial	NDUFS2	8	8	8	By MS/MS	By MS/MS	By MS/MS	By MS/MS	By MS/MS	By MS/MS					By MS/MS	By MS/MS	22,7	52,55	0	43,00
O75489	O75489	NADH dehydrogenase [ubiquinone] iron-sulfur protein 3, mitochondrial	NDUFS3	12	12	12	By MS/MS	By MS/MS	By MS/MS	By MS/MS	By MS/MS	By MS/MS	By MS/MS	By MS/MS	By MS/MS	By MS/MS	By MS/MS	By MS/MS	43,2	30,24	0	44,06
O43920	O43920	NADH dehydrogenase [ubiquinone] iron-sulfur protein 5	NDUFS5	4	4	4	By MS/MS	By MS/MS			By MS/MS	By MS/MS	By MS/MS	By MS/MS	By MS/MS	By MS/MS	By MS/MS	By MS/MS	36,8	12,52	0	12,43
O00217	O00217	NADH dehydrogenase [ubiquinone] iron-sulfur protein 8, mitochondrial	NDUFS8	4	4	4	By MS/MS	By MS/MS	By MS/MS	By MS/MS	By MS/MS	By MS/MS	By MS/MS	By MS/MS	By MS/MS	By MS/MS	By MS/MS	By MS/MS	23,3	23,71	0	33,51
P49821	P49821	NADH dehydrogenase [ubiquinone] flavoprotein 1, mitochondrial	NDUFV1	4	4	4	By MS/MS	By MS/MS	By MS/MS	By MS/MS	By MS/MS	By MS/MS					By MS/MS	By MS/MS	19,0	50,82	0	19,68

P19404	P19404	NADH dehydrogenase [ubiquinone] flavoprotein 2, mitochondrial	NDUFV2	5	5	5	By MS/MS	By MS/MS	By MS/MS	By MS/MS	By MS/MS	By MS/MS	By MS/MS	By MS/MS	By MS/MS		By MS/MS	By MS/MS	33,3	27,39	0	9,79
Q9NVZ3	Q9NVZ3	Adaptin ear-binding coat-associated protein 2	NECAP2	5	5	5	By MS/MS	By MS/MS	By MS/MS	By MS/MS	By MS/MS	By MS/MS	By MS/MS	By MS/MS	By MS/MS	By MS/MS	By MS/MS	By MS/MS	33,8	28,34	0	48,30
Q8WX92	Q8WX92	Negative elongation factor B	NELFB	6	6	6	By MS/MS	By MS/MS	By MS/MS			By MS/MS					By MS/MS	By MS/MS	17,2	65,70	0	7,14
Q9Y697	Q9Y697	Cysteine desulfurase, mitochondrial	NFS1	4	4	4		By MS/MS	By MS/MS		By MS/MS	By MS/MS						By MS/MS	13,8	50,20	0	23,48
Q9NX24	Q9NX24	H/ACA ribonucleoprotein complex subunit 2	NHP2	4	4	4	By MS/MS	By MS/MS	By MS/MS	By MS/MS	By MS/MS	By MS/MS	By MS/MS	By MS/MS	By MS/MS	By MS/MS	By MS/MS	By MS/MS	31,4	17,20	0	91,62
P55769	P55769	NHP2-like protein 1;NHP2-like protein 1, N-terminally processed	NHP2L1	7	7	7	By MS/MS	By MS/MS	By MS/MS	By MS/MS	By MS/MS	By MS/MS	By MS/MS	By MS/MS	By MS/MS	By MS/MS	By MS/MS	By MS/MS	45,3	14,17	0	85,94
Q9GZT8	Q9GZT8	NIF3-like protein 1	NIF3L1	4	4	4				By MS/MS				By MS/MS	By MS/MS	By MS/MS			15,9	41,97	0	5,48
Q9UFN0	Q9UFN0	Protein NipSnap homolog 3A	NIPSNAP3A	5	5	5	By MS/MS	By MS/MS	By MS/MS	By MS/MS	By MS/MS	By MS/MS		By MS/MS		By MS/MS	By MS/MS	By MS/MS	28,7	28,47	0	26,02
P15531	P15531	Nucleoside diphosphate kinase A	NME1	14	7	7	By MS/MS	By MS/MS	By MS/MS	By MS/MS	By MS/MS	By MS/MS	By MS/MS	By MS/MS	By MS/MS	By MS/MS	By MS/MS	By MS/MS	72,4	17,15	0	139,88
P22392;O60361	P22392;O60361	Nucleoside diphosphate kinase B;Putative nucleoside diphosphate kinase	NME2;NME2P1	17	17	10	By MS/MS	By MS/MS	By MS/MS	By MS/MS	By MS/MS	By MS/MS	By MS/MS	By MS/MS	By MS/MS	By MS/MS	By MS/MS	By MS/MS	86,2	17,30	0	323,31
P30419;O60551	P30419	Glycylpeptide N-tetradecanoyltransferase 1	NMT1	9	9	9	By MS/MS	By MS/MS	By MS/MS	By MS/MS	By MS/MS	By MS/MS	By MS/MS	By MS/MS			By MS/MS	By MS/MS	28,4	56,81	0	85,56
Q13423	Q13423	NAD(P) transhydrogenase, mitochondrial	NNT	13	13	13	By MS/MS	By MS/MS	By MS/MS	By MS/MS	By MS/MS	By MS/MS		By MS/MS	By MS/MS		By MS/MS	By MS/MS	17,5	113,89	0	101,26
Q9Y3T9	Q9Y3T9	Nucleolar complex protein 2 homolog	NOC2L	4	4	4		By MS/MS	By MS/MS	By MS/MS	By MS/MS	By MS/MS				By MS/MS		By MS/MS	8,3	84,92	0	17,38
Q9BVI4	Q9BVI4	Nucleolar complex protein 4 homolog	NOC4L	4	4	4	By MS/MS	By MS/MS	By MS/MS	By MS/MS	By MS/MS	By MS/MS					By MS/MS	By MS/MS	15,5	58,47	0	10,73
Q9H6R4	Q9H6R4	Nucleolar protein 6	NOL6	7	7	7	By MS/MS		By MS/MS	By MS/MS	By MS/MS	By MS/MS	By MS/MS	By MS/MS	By MS/MS	By MS/MS	By MS/MS	By MS/MS	9,9	127,59	0	42,20
Q15155;P69849;Q5JPE7	Q15155;P69849;Q5JPE7	Nodal modulator 1;Nodal modulator 3;Nodal modulator 2	NOMO1;NOMO3;NOMO2	16	16	16	By MS/MS	By MS/MS	By MS/MS	By MS/MS	By MS/MS	By MS/MS	By MS/MS	By MS/MS		By MS/MS	By MS/MS	By MS/MS	21,3	134,32	0	81,06
Q15233	Q15233	Non-POU domain-containing octamer-binding protein	NONO	11	10	10		By MS/MS	By MS/MS	By MS/MS	By MS/MS	By MS/MS	By MS/MS	By MS/MS	By MS/MS	By MS/MS	By MS/MS	By MS/MS	32,9	54,23	0	133,88
P46087	P46087	Probable 28S rRNA (cytosine(4447)-C(5))-methyltransferase	NOP2	6	6	6	By MS/MS	By MS/MS	By MS/MS	By MS/MS	By MS/MS	By MS/MS	By MS/MS		By MS/MS	By MS/MS	By MS/MS	By MS/MS	9,4	89,30	0	16,27
O00567	O00567	Nucleolar protein 56	NOP56	7	7	7	By MS/MS	By MS/MS		By MS/MS	By MS/MS	By MS/MS		By MS/MS			By MS/MS	By MS/MS	20,4	66,05	0	14,80
Q9Y2X3	Q9Y2X3	Nucleolar protein 58	NOP58	5	5	5		By MS/MS	By MS/MS	By MS/MS		By MS/MS		By MS/MS			By MS/MS	By MS/MS	13,0	59,58	0	24,25
Q9Y314	Q9Y314	Nitric oxide synthase-interacting protein	NOSIP	5	5	5	By MS/MS	By MS/MS	By MS/MS	By MS/MS	By MS/MS	By MS/MS	By MS/MS	By MS/MS	By MS/MS	By MS/MS	By MS/MS	By MS/MS	27,9	33,17	0	82,37
P55786;A6NEC2	P55786	Puromycin-sensitive aminopeptidase	NPEPPS	25	25	25	By MS/MS	By MS/MS	By MS/MS	By MS/MS	By MS/MS	By MS/MS	By MS/MS	By MS/MS	By MS/MS	By MS/MS	By MS/MS	By MS/MS	33,8	103,28	0	76,30
Q8TAT6	Q8TAT6	Nuclear protein localization protein 4 homolog	NPLOC4	6	6	6	By MS/MS	By MS/MS	By MS/MS	By MS/MS	By MS/MS	By MS/MS	By MS/MS	By MS/MS	By MS/MS	By MS/MS	By MS/MS	By MS/MS	18,8	68,12	0	251,50
P06748	P06748	Nucleophosmin	NPM1	15	15	15	By MS/MS	By MS/MS	By MS/MS	By MS/MS	By MS/MS	By MS/MS	By MS/MS	By MS/MS	By MS/MS	By MS/MS	By MS/MS	By MS/MS	58,8	32,58	0	323,31
O75607	O75607	Nucleoplasmin-3	NPM3	5	5	5	By MS/MS	By MS/MS	By MS/MS	By MS/MS	By MS/MS	By MS/MS	By MS/MS	By MS/MS	By MS/MS	By MS/MS	By MS/MS	By MS/MS	36,5	19,34	0	26,20
P15559	P15559	NAD(P)H dehydrogenase [quinone] 1	NQO1	6	6	6	By MS/MS	By MS/MS	By MS/MS	By MS/MS	By MS/MS	By MS/MS	By MS/MS	By MS/MS		By MS/MS	By MS/MS	By MS/MS	28,1	30,87	0	10,62
P16083	P16083	Ribosyldihydronicotinamide dehydrogenase [quinone]	NQO2	7	7	7	By MS/MS	By MS/MS	By MS/MS	By MS/MS	By MS/MS	By MS/MS	By MS/MS	By MS/MS	By MS/MS	By MS/MS	By MS/MS	By MS/MS	46,3	25,92	0	104,69
P01111	P01111	GTPase NRas	NRAS	5	5	2	By MS/MS	By MS/MS	By MS/MS	By MS/MS	By MS/MS	By MS/MS	By MS/MS	By MS/MS	By MS/MS	By MS/MS		By MS/MS	31,2	21,23	0	30,81
O43847	O43847	Nardilysin	NRD1	21	21	21	By MS/MS	By MS/MS	By MS/MS	By MS/MS	By MS/MS	By MS/MS	By MS/MS	By MS/MS		By MS/MS	By MS/MS	By MS/MS	23,2	131,57	0	304,97
Q15738	Q15738	Sterol-4-alpha-carboxylate 3-dehydrogenase, decarboxylating	NSDHL	9	9	9	By MS/MS	By MS/MS	By MS/MS	By MS/MS	By MS/MS	By MS/MS	By MS/MS	By MS/MS	By MS/MS	By MS/MS			41,6	41,90	0	28,46
P46459	P46459	Vesicle-fusing ATPase	NSF	6	6	6	By MS/MS	By MS/MS	By MS/MS	By MS/MS					By MS/MS		By MS/MS		9,0	82,59	0	13,16
Q9UNZ2	Q9UNZ2	NSFL1 cofactor p47	NSFL1C	9	9	9	By MS/MS	By MS/MS	By MS/MS	By MS/MS	By MS/MS	By MS/MS		By MS/MS		By MS/MS	By MS/MS	By MS/MS	32,7	40,57	0	193,52
Q08J23	Q08J23	tRNA (cytosine(34)-C(5))-methyltransferase	NSUN2	15	15	15	By MS/MS	By MS/MS	By MS/MS	By MS/MS	By MS/MS	By MS/MS	By MS/MS	By MS/MS	By MS/MS	By MS/MS	By MS/MS	By MS/MS	28,4	86,47	0	141,06
Q8TCD5	Q8TCD5	5(3)-deoxyribonucleotidase, cytosolic type	NT5C	5	5	5	By MS/MS	By MS/MS		By MS/MS	By MS/MS	By MS/MS			By MS/MS				43,3	23,38	0	21,95
P49902	P49902	Cytosolic purine 5-nucleotidase	NT5C2	7	7	7	By MS/MS	By MS/MS	By MS/MS	By MS/MS	By MS/MS	By MS/MS					By MS/MS	By MS/MS	22,5	64,97	0	29,18
Q5TFE4	Q5TFE4	5-nucleotidase domain-containing protein 1	NT5DC1	5	5	5	By MS/MS	By MS/MS	By MS/MS	By MS/MS	By MS/MS	By MS/MS					By MS/MS	By MS/MS	22,4	51,84	0	14,62
Q9BV86	Q9BV86	N-terminal Xaa-Pro-Lys N-methyltransferase 1;N-terminal Xaa-Pro-Lys N-methyltransferase 1, N-terminally processed	NTMT1	7	7	7	By MS/MS	By MS/MS	By MS/MS	By MS/MS	By MS/MS	By MS/MS	By MS/MS	By MS/MS	By MS/MS	By MS/MS	By MS/MS	By MS/MS	46,2	25,39	0	180,92
Q9BSD7	Q9BSD7	Cancer-related nucleoside-triphosphatase	NTPCR	6	6	6	By MS/MS	By MS/MS	By MS/MS	By MS/MS	By MS/MS	By MS/MS	By MS/MS	By MS/MS	By MS/MS	By MS/MS	By MS/MS	By MS/MS	36,3	20,71	0	45,72
Q9Y266	Q9Y266	Nuclear migration protein nudC	NUDC	18	18	18	By MS/MS	By MS/MS	By MS/MS	By MS/MS	By MS/MS	By MS/MS	By MS/MS	By MS/MS	By MS/MS	By MS/MS			50,8	38,24	0	80,48
Q96RS6	Q96RS6	NudC domain-containing protein 1	NUDCD1	4	4	4	By MS/MS	By MS/MS	By MS/MS		By MS/MS	By MS/MS		By MS/MS			By MS/MS	By MS/MS	11,0	66,76	0	11,22
Q8WVJ2	Q8WVJ2	NudC domain-containing protein 2	NUDCD2	7	7	7	By MS/MS	By MS/MS	By MS/MS	By MS/MS	By MS/MS	By MS/MS	By MS/MS	By MS/MS	By MS/MS	By MS/MS	By MS/MS	By MS/MS	43,9	17,68	0	242,00
O43809	O43809	Cleavage and polyadenylation specificity factor subunit 5	NUDT21	13	13	13	By MS/MS	By MS/MS	By MS/MS	By MS/MS	By MS/MS	By MS/MS	By MS/MS	By MS/MS	By MS/MS	By MS/MS	By MS/MS	By MS/MS	51,5	26,23	0	60,39
O95989	O95989	Diphosphoinositol polyphosphate phosphohydrolase 1	NUDT3	4	4	4	By MS/MS	By MS/MS		By MS/MS	By MS/MS	By MS/MS			By MS/MS	By MS/MS			35,5	19,47	0	11,90
Q9UKK9	Q9UKK9	ADP-sugar pyrophosphatase	NUDT5	13	13	13	By MS/MS	By MS/MS	By MS/MS	By MS/MS	By MS/MS	By MS/MS	By MS/MS	By MS/MS	By MS/MS	By MS/MS	By MS/MS	By MS/MS	56,6	24,33	0	111,31
Q14980	Q14980	Nuclear mitotic apparatus protein 1	NUMA1	19	19	19	By MS/MS	By MS/MS	By MS/MS	By MS/MS	By MS/MS	By MS/MS	By MS/MS	By MS/MS	By MS/MS	By MS/MS	By MS/MS	By MS/MS	15,8	238,26	0	76,94
P57740	P57740	Nuclear pore complex protein Nup107	NUP107	9	9	9	By MS/MS	By MS/MS	By MS/MS	By MS/MS	By MS/MS	By MS/MS	By MS/MS						16,2	106,37	0	126,38
Q8WUM0	Q8WUM0	Nuclear pore complex protein Nup133	NUP133	14	14	14	By MS/MS	By MS/MS	By MS/MS	By MS/MS	By MS/MS	By MS/MS	By MS/MS	By MS/MS		By MS/MS	By MS/MS	By MS/MS	17,0	128,98	0	62,85
O75694	O75694	Nuclear pore complex protein Nup155	NUP155	23	23	23	By MS/MS	By MS/MS	By MS/MS	By MS/MS	By MS/MS	By MS/MS	By MS/MS	By MS/MS		By MS/MS	By MS/MS	By MS/MS	26,7	155,20	0	225,71
Q12769	Q12769	Nuclear pore complex protein Nup160	NUP160	8	8	8	By MS/MS	By MS/MS	By MS/MS	By MS/MS	By MS/MS	By MS/MS	By MS/MS	By MS/MS	By MS/MS	By MS/MS	By MS/MS	By MS/MS	8,9	162,12	0	26,48
Q5SRE5	Q5SRE5	Nucleoporin NUP188 homolog	NUP188	11	11	11	By MS/MS	By MS/MS	By MS/MS	By MS/MS	By MS/MS	By MS/MS	By MS/MS		By MS/MS				9,3	196,04	0	34,03
Q92621	Q92621	Nuclear pore complex protein Nup205	NUP205	34	34	34	By MS/MS	By MS/MS	By MS/MS	By MS/MS	By MS/MS	By MS/MS	By MS/MS	By MS/MS	By MS/MS	By MS/MS	By MS/MS	By MS/MS	24,8	227,92	0	323,31
Q8TEM1	Q8TEM1	Nuclear pore membrane glycoprotein 210	NUP210	23	23	23	By MS/MS	By MS/MS	By MS/MS	By MS/MS	By MS/MS	By MS/MS	By MS/MS	By MS/MS	By MS/MS	By MS/MS	By MS/MS	By MS/MS	19,3	205,11	0	101,26
P35658	P35658	Nuclear pore complex protein Nup214	NUP214	32	32	32	By MS/MS	By MS/MS	By MS/MS	By MS/MS	By MS/MS	By MS/MS		By MS/MS			By MS/MS	By MS/MS	28,3	213,62	0	191,98
Q8NFH4	Q8NFH4	Nucleoporin Nup37	NUP37	4	4	4	By MS/MS	By MS/MS	By MS/MS	By MS/MS	By MS/MS	By MS/MS	By MS/MS		By MS/MS				19,9	36,71	0	16,18
Q8NFH3	Q8NFH3	Nucleoporin Nup43	NUP43	7	7	7	By MS/MS	By MS/MS	By MS/MS	By MS/MS	By MS/MS	By MS/MS	By MS/MS	By MS/MS			By MS/MS	By MS/MS	34,5	42,15	0	108,07
Q7Z3B4	Q7Z3B4	Nucleoporin p54	NUP54	5	5	5		By MS/MS	By MS/MS	By MS/MS	By MS/MS	By MS/MS					By MS/MS	By MS/MS	14,8	55,44	0	62,63
P37198	P37198	Nuclear pore glycoprotein p62	NUP62	5	5	5	By MS/MS	By MS/MS	By MS/MS	By MS/MS	By MS/MS	By MS/MS	By MS/MS	By MS/MS	By MS/MS	By MS/MS	By MS/MS	By MS/MS	16,7	53,25	0	22,21
Q9BW27	Q9BW27	Nuclear pore complex protein Nup85	NUP85	4	4	4	By MS/MS	By MS/MS				By MS/MS					By MS/MS	By MS/MS	10,8	75,02	0	20,99
Q8N1F7	Q8N1F7	Nuclear pore complex protein Nup93	NUP93	19	19	19	By MS/MS	By MS/MS	By MS/MS	By MS/MS	By MS/MS	By MS/MS	By MS/MS	By MS/MS	By MS/MS	By MS/MS	By MS/MS	By MS/MS	37,5	93,49	0	147,25
P52948	P52948	Nuclear pore complex protein Nup98-Nup96;Nuclear pore complex protein Nup98;Nuclear pore complex protein Nup96	NUP98	10	10	10	By MS/MS	By MS/MS	By MS/MS		By MS/MS	By MS/MS	By MS/MS	By MS/MS	By MS/MS	By MS/MS	By MS/MS	By MS/MS	8,1	197,58	0	57,76
P61970	P61970	Nuclear transport factor 2	NUTF2	7	7	7	By MS/MS	By MS/MS	By MS/MS	By MS/MS	By MS/MS	By MS/MS	By MS/MS	By MS/MS	By MS/MS	By MS/MS	By MS/MS	By MS/MS	79,5	14,48	0	50,26
Q9UBU9	Q9UBU9	Nuclear RNA export factor 1	NXF1	4	4	4	By MS/MS	By MS/MS	By MS/MS	By MS/MS	By MS/MS	By MS/MS	By MS/MS	By MS/MS	By MS/MS	By MS/MS	By MS/MS	By MS/MS	10,3	70,18	0	17,17
Q9Y530	Q9Y530	O-acetyl-ADP-ribose deacetylase 1	OARD1	5	5	5	By MS/MS	By MS/MS	By MS/MS	By MS/MS	By MS/MS	By MS/MS	By MS/MS	By MS/MS	By MS/MS	By MS/MS	By MS/MS	By MS/MS	36,2	17,03	0	136,33
P04181	P04181	Ornithine aminotransferase, mitochondrial;Ornithine aminotransferase, hepatic form;Ornithine aminotransferase, renal form	OAT	23	23	23	By MS/MS	By MS/MS	By MS/MS	By MS/MS	By MS/MS	By MS/MS	By MS/MS	By MS/MS	By MS/MS	By MS/MS	By MS/MS	By MS/MS	67,4	48,53	0	222,34
Q02218;Q9ULD0	Q02218	2-oxoglutarate dehydrogenase, mitochondrial	OGDH	8	8	8	By MS/MS	By MS/MS	By MS/MS	By MS/MS	By MS/MS	By MS/MS	By MS/MS	By MS/MS	By MS/MS	By MS/MS	By MS/MS	By MS/MS	10,9	115,93	0	20,00

O15294	O15294	UDP-N-acetylglucosamine--peptide N-acetylglucosaminyltransferase 110 kDa subunit	OGT	4	4	4	By MS/MS	By MS/MS	By MS/MS	By MS/MS	By MS/MS	By MS/MS		By MS/MS		By MS/MS	By MS/MS	By MS/MS	7,0	116,92	0	24,14
Q9NTK5	Q9NTK5	Obg-like ATPase 1	OLA1	24	24	24	By MS/MS	By MS/MS	By MS/MS	By MS/MS	By MS/MS	By MS/MS	By MS/MS	By MS/MS	By MS/MS		By MS/MS	By MS/MS	71,5	44,74	0	132,90
O60313	O60313	Dynamin-like 120 kDa protein, mitochondrial;Dynamin-like 120 kDa protein, form S1	OPA1	7	7	7	By MS/MS	By MS/MS	By MS/MS	By MS/MS	By MS/MS	By MS/MS		By MS/MS					12,3	111,63	0	41,59
Q9NPF4	Q9NPF4	Probable tRNA N6-adenosine threonylcarbamoyltransferase	OSGEP	4	4	4	By MS/MS	By MS/MS	By MS/MS	By MS/MS	By MS/MS	By MS/MS	By MS/MS	By MS/MS	By MS/MS	By MS/MS	By MS/MS		23,6	36,43	0	34,77
Q92882	Q92882	Osteoclast-stimulating factor 1	OSTF1	5	5	5	By MS/MS	By MS/MS	By MS/MS	By MS/MS	By MS/MS	By MS/MS		By MS/MS	By MS/MS	By MS/MS	By MS/MS	By MS/MS	27,1	23,79	0	28,51
Q96FW1	Q96FW1	Ubiquitin thioesterase OTUB1	OTUB1	8	8	8	By MS/MS	By MS/MS	By MS/MS	By MS/MS	By MS/MS	By MS/MS	By MS/MS	By MS/MS	By MS/MS	By MS/MS	By MS/MS	By MS/MS	40,6	31,28	0	115,95
Q8N6M0	Q8N6M0	OTU domain-containing protein 6B	OTUD6B	5	5	5	By MS/MS	By MS/MS	By MS/MS	By MS/MS	By MS/MS	By MS/MS	By MS/MS		By MS/MS		By MS/MS	By MS/MS	24,9	33,81	0	31,29
P55809	P55809	Succinyl-CoA:3-ketoacid coenzyme A transferase 1, mitochondrial	OXCT1	4	4	4	By MS/MS	By MS/MS	By MS/MS		By MS/MS	By MS/MS					By MS/MS	By MS/MS	16,9	56,16	0	323,31
O95747	O95747	Serine/threonine-protein kinase OSR1	OXSR1	8	8	8	By MS/MS	By MS/MS	By MS/MS	By MS/MS	By MS/MS	By MS/MS		By MS/MS	By MS/MS		By MS/MS	By MS/MS	21,6	58,02	0	34,09
P07237	P07237	Protein disulfide-isomerase	P4HB	31	31	31	By MS/MS	By MS/MS	By MS/MS	By MS/MS	By MS/MS	By MS/MS	By MS/MS	By MS/MS			By MS/MS	By MS/MS	61,6	57,12	0	323,31
Q9UQ80	Q9UQ80	Proliferation-associated protein 2G4	PA2G4	26	26	26	By MS/MS	By MS/MS	By MS/MS	By MS/MS	By MS/MS	By MS/MS	By MS/MS	By MS/MS	By MS/MS	By MS/MS	By MS/MS	By MS/MS	69,0	43,79	0	323,31
P11940;Q9H361;Q4VXU2;Q5JQF8;Q96DU9	P11940;Q9H361	Polyadenylate-binding protein 1;Polyadenylate-binding protein 3	PABPC1;PABPC3	26	26	17	By MS/MS	By MS/MS	By MS/MS	By MS/MS	By MS/MS	By MS/MS	By MS/MS	By MS/MS	By MS/MS	By MS/MS	By MS/MS	By MS/MS	42,3	70,67	0	323,31
Q13310	Q13310	Polyadenylate-binding protein 4	PABPC4	16	7	7	By MS/MS	By MS/MS	By MS/MS	By MS/MS	By MS/MS	By MS/MS	By matchin	By matchin	By matchin	By matchin	By MS/MS	By matchin	29,7	70,78	0	8,16
Q86U42;A6NDY0	Q86U42	Polyadenylate-binding protein 2	PABPN1	4	4	4	By MS/MS	By MS/MS	By MS/MS	By MS/MS		By MS/MS	By MS/MS	By MS/MS	By MS/MS	By MS/MS	By MS/MS	By MS/MS	27,8	32,75	0	23,72
P68402	P68402	Platelet-activating factor acetylhydrolase IB subunit beta	PAFAH1B2	4	4	4	By MS/MS	By MS/MS	By MS/MS	By MS/MS	By MS/MS	By MS/MS	By MS/MS	By MS/MS	By MS/MS	By MS/MS	By MS/MS		30,6	25,57	0	97,81
Q15102	Q15102	Platelet-activating factor acetylhydrolase IB subunit gamma	PAFAH1B3	8	8	8	By MS/MS	By MS/MS	By MS/MS	By MS/MS	By MS/MS	By MS/MS	By MS/MS	By MS/MS	By MS/MS	By MS/MS	By MS/MS	By MS/MS	46,3	25,73	0	93,37
P22234	P22234	Multifunctional protein ADE2;Phosphoribosylaminoimidazole-succinocarboxamide synthase;Phosphoribosylaminoimidazole carboxylase	PAICS	31	31	31	By MS/MS	By MS/MS	By MS/MS	By MS/MS	By MS/MS	By MS/MS	By MS/MS	By MS/MS	By MS/MS	By MS/MS	By MS/MS	By MS/MS	81,2	47,08	0	323,31
Q13177;O75914;Q13153	Q13177	Serine/threonine-protein kinase PAK 2;PAK-2p27;PAK-2p34	PAK2	17	17	17	By MS/MS	By MS/MS	By MS/MS	By MS/MS	By MS/MS	By MS/MS	By MS/MS	By MS/MS	By MS/MS	By MS/MS	By MS/MS	By MS/MS	51,0	58,04	0	280,54
Q9Y3D7	Q9Y3D7	Mitochondrial import inner membrane translocase subunit TIM16	PAM16	4	4	4	By MS/MS	By MS/MS	By MS/MS	By MS/MS	By MS/MS	By MS/MS	By MS/MS	By MS/MS		By MS/MS	By MS/MS	By MS/MS	51,2	13,83	0	163,28
Q99497	Q99497	Protein deglycase DJ-1	PARK7	18	18	18	By MS/MS	By MS/MS	By MS/MS	By MS/MS	By MS/MS	By MS/MS	By MS/MS	By MS/MS	By MS/MS	By MS/MS	By MS/MS	By MS/MS	81,5	19,89	0	263,91
P09874	P09874	Poly [ADP-ribose] polymerase 1	PARP1	31	31	31	By MS/MS	By MS/MS	By MS/MS	By MS/MS	By MS/MS	By MS/MS	By MS/MS	By MS/MS	By MS/MS	By MS/MS	By MS/MS	By MS/MS	41,5	113,08	0	260,06
P61457	P61457	Pterin-4-alpha-carbinolamine dehydratase	PCBD1	5	5	5	By MS/MS	By MS/MS			By MS/MS		By MS/MS	By MS/MS	By MS/MS	By MS/MS	By MS/MS	By MS/MS	57,7	12,00	0	38,33
Q15365	Q15365	Poly(rC)-binding protein 1	PCBP1	14	14	12	By MS/MS	By MS/MS	By MS/MS	By MS/MS	By MS/MS	By MS/MS	By MS/MS	By MS/MS	By MS/MS	By MS/MS	By MS/MS	By MS/MS	80,1	37,50	0	323,31
Q15366	Q15366	Poly(rC)-binding protein 2	PCBP2	14	12	11	By MS/MS	By MS/MS	By MS/MS	By MS/MS	By MS/MS	By MS/MS	By MS/MS	By MS/MS	By MS/MS	By MS/MS	By MS/MS	By MS/MS	61,9	38,58	0	120,15
Q16822	Q16822	Phosphoenolpyruvate carboxykinase [GTP], mitochondrial	PCK2	5	5	5	By MS/MS	By MS/MS	By MS/MS	By MS/MS	By MS/MS	By MS/MS		By MS/MS		By MS/MS			9,8	70,73	0	11,81
P22061	P22061	Protein-L-isoaspartate(D-aspartate) O-methyltransferase	PCMT1	14	14	14	By MS/MS	By MS/MS	By MS/MS	By MS/MS	By MS/MS	By MS/MS	By MS/MS	By MS/MS	By MS/MS	By MS/MS	By MS/MS	By MS/MS	70,5	24,64	0	265,63
P12004	P12004	Proliferating cell nuclear antigen	PCNA	14	14	14	By MS/MS	By MS/MS	By MS/MS	By MS/MS	By MS/MS	By MS/MS	By MS/MS	By MS/MS	By MS/MS	By MS/MS	By MS/MS	By MS/MS	74,7	28,77	0	323,31
Q99447	Q99447	Ethanolamine-phosphate cytidylyltransferase	PCYT2	5	5	5	By MS/MS	By MS/MS	By MS/MS	By MS/MS	By MS/MS	By MS/MS	By MS/MS	By MS/MS	By MS/MS				20,3	43,84	0	14,99
Q13442	Q13442	28 kDa heat- and acid-stable phosphoprotein	PDAP1	5	5	5	By MS/MS	By MS/MS	By MS/MS	By MS/MS	By MS/MS	By MS/MS	By MS/MS			By MS/MS	By MS/MS	By MS/MS	32,0	20,63	0	174,24
Q9BUL8	Q9BUL8	Programmed cell death protein 10	PDCD10	5	5	5	By MS/MS		By MS/MS	By MS/MS	By MS/MS	By MS/MS			By MS/MS				26,4	24,70	0	7,20
Q14690	Q14690	Protein RRP5 homolog	PDCD11	7	7	7	By MS/MS	By MS/MS	By MS/MS	By MS/MS	By MS/MS	By MS/MS	By MS/MS	By MS/MS	By MS/MS	By MS/MS	By MS/MS	By MS/MS	6,6	208,70	0	39,88
O14737	O14737	Programmed cell death protein 5	PDCD5	7	7	7	By MS/MS	By MS/MS	By MS/MS	By MS/MS	By MS/MS	By MS/MS	By MS/MS	By MS/MS	By MS/MS	By MS/MS	By MS/MS	By MS/MS	51,2	14,29	0	105,52
O75340	O75340	Programmed cell death protein 6	PDCD6	9	9	9	By MS/MS	By MS/MS	By MS/MS	By MS/MS	By MS/MS	By MS/MS	By MS/MS	By MS/MS	By MS/MS	By MS/MS	By MS/MS	By MS/MS	63,4	21,87	0	54,40
Q8WUM4	Q8WUM4	Programmed cell death 6-interacting protein	PDCD6IP	40	40	40	By MS/MS	By MS/MS	By MS/MS	By MS/MS	By MS/MS	By MS/MS	By MS/MS	By MS/MS	By MS/MS	By MS/MS	By MS/MS	By MS/MS	65,6	96,02	0	323,31
Q9H2J4	Q9H2J4	Phosducin-like protein 3	PDCL3	6	6	6	By MS/MS	By MS/MS	By MS/MS		By MS/MS				By MS/MS	By MS/MS		By MS/MS	36,0	27,61	0	10,04
Q8NB37	Q8NB37	Parkinson disease 7 domain-containing protein 1	PDDC1	4	4	4	By MS/MS	By MS/MS	By MS/MS	By MS/MS	By MS/MS	By MS/MS			By MS/MS	By MS/MS	By MS/MS	By MS/MS	32,7	23,30	0	45,19
P08559;P29803	P08559	Pyruvate dehydrogenase E1 component subunit alpha, somatic form, mitochondrial	PDHA1	5	5	5	By MS/MS	By MS/MS	By MS/MS	By MS/MS	By MS/MS	By MS/MS	By MS/MS		By MS/MS	By MS/MS			16,9	43,30	0	12,77
P11177	P11177	Pyruvate dehydrogenase E1 component subunit beta, mitochondrial	PDHB	12	12	12	By MS/MS	By MS/MS	By MS/MS	By MS/MS	By MS/MS	By MS/MS		By MS/MS	By MS/MS	By MS/MS	By MS/MS	By MS/MS	47,6	39,23	0	139,85
P30101	P30101	Protein disulfide-isomerase A3	PDIA3	29	29	29	By MS/MS	By MS/MS	By MS/MS	By MS/MS	By MS/MS	By MS/MS	By MS/MS	By MS/MS			By MS/MS	By MS/MS	63,0	56,78	0	189,58
P13667	P13667	Protein disulfide-isomerase A4	PDIA4	31	31	31	By MS/MS	By MS/MS	By MS/MS	By MS/MS	By MS/MS	By MS/MS	By MS/MS	By MS/MS	By MS/MS	By MS/MS	By MS/MS	By MS/MS	49,9	72,93	0	323,31
Q15084	Q15084	Protein disulfide-isomerase A6	PDIA6	17	17	17	By MS/MS	By MS/MS	By MS/MS	By MS/MS	By MS/MS	By MS/MS	By MS/MS	By MS/MS	By MS/MS	By MS/MS	By MS/MS	By MS/MS	49,5	48,12	0	267,19
O00151	O00151	PDZ and LIM domain protein 1	PDLIM1	11	11	11	By MS/MS	By MS/MS	By MS/MS	By MS/MS	By MS/MS	By MS/MS	By MS/MS	By MS/MS	By MS/MS	By MS/MS	By MS/MS	By MS/MS	60,5	36,07	0	67,38
Q96HC4	Q96HC4	PDZ and LIM domain protein 5	PDLIM5	6	6	6		By MS/MS	By MS/MS	By MS/MS	By MS/MS	By MS/MS	By MS/MS		By MS/MS	By MS/MS	By MS/MS	By MS/MS	14,8	63,94	0	9,79
O00764	O00764	Pyridoxal kinase	PDXK	9	9	9	By MS/MS	By MS/MS	By MS/MS	By MS/MS	By MS/MS	By MS/MS	By MS/MS	By MS/MS	By MS/MS	By MS/MS	By MS/MS	By MS/MS	29,2	35,10	0	96,75
P30086	P30086	Phosphatidylethanolamine-binding protein 1;Hippocampal cholinergic neurostimulating peptide	PEBP1	17	17	17	By MS/MS	By MS/MS	By MS/MS	By MS/MS	By MS/MS	By MS/MS	By MS/MS	By MS/MS	By MS/MS	By MS/MS	By MS/MS	By MS/MS	85,6	21,06	0	323,31
Q8IZL8	Q8IZL8	Proline-, glutamic acid- and leucine-rich protein 1	PELP1	8	8	8	By MS/MS	By MS/MS	By MS/MS	By MS/MS	By MS/MS	By MS/MS	By MS/MS	By MS/MS			By MS/MS	By MS/MS	11,1	119,70	0	19,79
O15067	O15067	Phosphoribosylformylglycinamidine synthase	PFAS	33	33	33	By MS/MS	By MS/MS	By MS/MS	By MS/MS	By MS/MS	By MS/MS	By MS/MS	By MS/MS	By MS/MS	By MS/MS	By MS/MS	By MS/MS	35,5	144,73	0	284,88
Q9UHV9	Q9UHV9	Prefoldin subunit 2	PFDN2	5	5	5	By MS/MS	By MS/MS	By MS/MS	By MS/MS	By MS/MS	By MS/MS	By MS/MS	By MS/MS	By MS/MS	By MS/MS	By MS/MS	By MS/MS	32,5	16,65	0	301,93
Q9NQP4	Q9NQP4	Prefoldin subunit 4	PFDN4	5	5	5	By MS/MS	By MS/MS	By MS/MS	By MS/MS	By MS/MS	By MS/MS	By MS/MS	By MS/MS	By MS/MS	By MS/MS	By MS/MS	By MS/MS	38,1	15,31	0	94,29
Q99471	Q99471	Prefoldin subunit 5	PFDN5	9	9	9	By MS/MS	By MS/MS	By MS/MS	By MS/MS	By MS/MS	By MS/MS	By MS/MS	By MS/MS	By MS/MS	By MS/MS	By MS/MS	By MS/MS	68,2	17,33	0	323,31
O15212	O15212	Prefoldin subunit 6	PFDN6	9	9	9	By MS/MS	By MS/MS	By MS/MS		By MS/MS	By MS/MS	By MS/MS	By MS/MS	By MS/MS	By MS/MS		By MS/MS	58,1	14,58	0	53,38
P17858;P08237	P17858	ATP-dependent 6-phosphofructokinase, liver type	PFKL	13	12	12	By MS/MS	By MS/MS	By MS/MS	By MS/MS	By MS/MS	By MS/MS	By MS/MS	By MS/MS	By MS/MS	By MS/MS	By MS/MS	By matchin	27,4	85,02	0	33,40
Q01813	Q01813	ATP-dependent 6-phosphofructokinase, platelet type	PFKP	14	14	13	By MS/MS	By MS/MS	By MS/MS	By MS/MS	By MS/MS	By MS/MS	By MS/MS	By MS/MS	By MS/MS	By MS/MS	By MS/MS	By MS/MS	23,5	85,60	0	323,31
P07737	P07737	Profilin-1	PFN1	16	16	12	By MS/MS	By MS/MS	By MS/MS	By MS/MS	By MS/MS	By MS/MS	By MS/MS	By MS/MS	By MS/MS	By MS/MS	By MS/MS	By MS/MS	96,4	15,05	0	323,31
P18669;Q8N0Y7;P15259	P18669	Phosphoglycerate mutase 1	PGAM1	16	16	16	By MS/MS	By MS/MS	By MS/MS	By MS/MS	By MS/MS	By MS/MS	By MS/MS	By MS/MS	By MS/MS	By MS/MS	By MS/MS	By MS/MS	72,4	28,80	0	323,31
Q96HS1	Q96HS1	Serine/threonine-protein phosphatase PGAM5, mitochondrial	PGAM5	9	9	9	By MS/MS	By MS/MS	By MS/MS	By MS/MS	By MS/MS	By MS/MS	By MS/MS	By MS/MS	By MS/MS	By MS/MS	By MS/MS	By MS/MS	31,1	32,00	0	13,04
P52209	P52209	6-phosphogluconate dehydrogenase, decarboxylating	PGD	30	30	30	By MS/MS	By MS/MS	By MS/MS	By MS/MS	By MS/MS	By MS/MS	By MS/MS	By MS/MS			By MS/MS	By MS/MS	80,3	53,14	0	323,31
P00558;P07205	P00558	Phosphoglycerate kinase 1	PGK1	35	35	35	By MS/MS	By MS/MS	By MS/MS	By MS/MS	By MS/MS	By MS/MS	By MS/MS	By MS/MS	By MS/MS	By MS/MS	By MS/MS	By MS/MS	79,9	44,61	0	323,31
O95336	O95336	6-phosphogluconolactonase	PGLS	14	14	14	By MS/MS	By MS/MS	By MS/MS	By MS/MS	By MS/MS	By MS/MS	By MS/MS	By MS/MS	By MS/MS	By MS/MS	By MS/MS	By MS/MS	74,0	27,55	0	131,98

Q96G03	Q96G03	Phosphoglucomutase-2	PGM2	9	9	9	By MS/MS	By MS/MS	By MS/MS	By MS/MS	By MS/MS	By MS/MS	By MS/MS	By MS/MS	By MS/MS	By MS/MS	By MS/MS	By MS/MS	22,7	68,28	0	197,13
O95394	O95394	Phosphoacetylglucosamine mutase	PGM3	6	6	6	By MS/MS	By MS/MS	By MS/MS	By MS/MS	By MS/MS	By MS/MS	By MS/MS	By MS/MS	By MS/MS	By MS/MS	By MS/MS	By MS/MS	18,8	59,85	0	155,56
A6NDG6	A6NDG6	Phosphoglycolate phosphatase	PGP	6	6	6	By MS/MS	By MS/MS	By MS/MS	By MS/MS	By MS/MS	By MS/MS	By MS/MS	By MS/MS	By MS/MS	By MS/MS	By MS/MS	By MS/MS	27,1	34,01	0	136,83
O00264	O00264	Membrane-associated progesterone receptor component 1	PGRMC1	11	11	10	By MS/MS	By MS/MS	By MS/MS	By MS/MS	By MS/MS	By MS/MS	By MS/MS	By MS/MS	By MS/MS	By MS/MS	By MS/MS	By MS/MS	55,4	21,67	0	96,98
O15173	O15173	Membrane-associated progesterone receptor component 2	PGRMC2	5	4	4	By MS/MS	By MS/MS	By MS/MS	By MS/MS	By MS/MS	By MS/MS					By MS/MS	By MS/MS	22,9	23,82	0	80,54
P35232	P35232	Prohibitin	PHB	14	14	14	By MS/MS	By MS/MS	By MS/MS	By MS/MS	By MS/MS	By MS/MS	By MS/MS	By MS/MS	By MS/MS	By MS/MS	By MS/MS	By MS/MS	68,0	29,80	0	323,31
Q99623	Q99623	Prohibitin-2	PHB2	16	16	16	By MS/MS	By MS/MS	By MS/MS	By MS/MS	By MS/MS	By MS/MS	By MS/MS	By MS/MS	By MS/MS	By MS/MS	By MS/MS	By MS/MS	55,5	33,30	0	299,81
Q7RTV0	Q7RTV0	PHD finger-like domain-containing protein 5A	PHF5A	5	5	5	By MS/MS	By MS/MS	By MS/MS	By MS/MS	By MS/MS	By MS/MS	By MS/MS	By MS/MS	By MS/MS	By MS/MS	By MS/MS	By MS/MS	54,5	12,41	0	36,49
O43175	O43175	D-3-phosphoglycerate dehydrogenase	PHGDH	17	17	17	By MS/MS	By MS/MS	By MS/MS	By MS/MS	By MS/MS	By MS/MS	By MS/MS	By MS/MS	By MS/MS	By MS/MS	By MS/MS	By MS/MS	46,5	56,65	0	323,31
Q9NRX4	Q9NRX4	14 kDa phosphohistidine phosphatase	PHPT1	5	5	5	By MS/MS	By MS/MS			By MS/MS	By MS/MS	By MS/MS	By MS/MS	By MS/MS	By MS/MS	By MS/MS	By MS/MS	59,2	13,83	0	20,24
Q13492;O60641	Q13492	Phosphatidylinositol-binding clathrin assembly protein	PICALM	10	10	10	By MS/MS	By MS/MS	By MS/MS	By MS/MS	By MS/MS	By MS/MS	By MS/MS	By MS/MS	By MS/MS	By MS/MS	By MS/MS	By MS/MS	25,0	70,75	0	190,41
Q13526;O15428	Q13526	Peptidyl-prolyl cis-trans isomerase NIMA-interacting 1	PIN1	6	6	6	By MS/MS	By MS/MS	By MS/MS	By MS/MS	By MS/MS	By MS/MS	By MS/MS	By MS/MS	By MS/MS	By MS/MS	By MS/MS	By MS/MS	57,1	18,24	0	124,69
Q9Y237	Q9Y237	Peptidyl-prolyl cis-trans isomerase NIMA-interacting 4	PIN4	4	4	4	By MS/MS	By MS/MS	By MS/MS	By MS/MS	By MS/MS	By MS/MS	By MS/MS	By MS/MS	By MS/MS	By MS/MS	By MS/MS	By MS/MS	42,0	13,81	0	61,44
Q9GZP4	Q9GZP4	PITH domain-containing protein 1	PITHD1	10	10	10	By MS/MS	By MS/MS	By MS/MS	By MS/MS	By MS/MS	By MS/MS	By MS/MS	By MS/MS	By MS/MS	By MS/MS	By MS/MS	By MS/MS	57,3	24,18	0	82,74
P48739	P48739	Phosphatidylinositol transfer protein beta isoform	PITPNB	7	7	7	By MS/MS	By MS/MS	By MS/MS	By MS/MS	By MS/MS	By MS/MS		By MS/MS	By MS/MS	By MS/MS		By MS/MS	35,8	31,54	0	26,51
Q5JRX3	Q5JRX3	Presequence protease, mitochondrial	PITRM1	4	4	4	By MS/MS		By MS/MS	By MS/MS		By MS/MS						By MS/MS	5,9	117,41	0	3,29
P30613	P30613	Pyruvate kinase PKLR	PKLR	13	12	12	By MS/MS	By MS/MS	By MS/MS	By MS/MS	By MS/MS	By MS/MS	By matchin	By MS/MS			By MS/MS	By MS/MS	33,8	61,83	0	50,09
P14618	P14618	Pyruvate kinase PKM	PKM	39	39	38	By MS/MS	By MS/MS	By MS/MS	By MS/MS	By MS/MS	By MS/MS	By MS/MS	By MS/MS	By MS/MS	By MS/MS	By MS/MS	By MS/MS	75,5	57,94	0	323,31
Q9Y263	Q9Y263	Phospholipase A-2-activating protein	PLAA	7	7	7	By MS/MS	By MS/MS	By MS/MS	By MS/MS	By MS/MS	By MS/MS	By MS/MS	By MS/MS	By MS/MS	By MS/MS	By MS/MS	By MS/MS	14,6	87,16	0	52,67
Q15149	Q15149	Plectin	PLEC	14	14	14	By MS/MS	By MS/MS	By MS/MS	By MS/MS	By MS/MS	By MS/MS			By MS/MS				5,0	531,78	0	42,58
O60664	O60664	Perilipin-3	PLIN3	18	18	18	By MS/MS	By MS/MS	By MS/MS	By MS/MS	By MS/MS	By MS/MS	By MS/MS	By MS/MS	By MS/MS	By MS/MS	By MS/MS	By MS/MS	65,9	47,07	0	323,31
Q02809	Q02809	Procollagen-lysine,2-oxoglutarate 5-dioxygenase 1	PLOD1	4	4	4	By MS/MS	By MS/MS	By MS/MS	By MS/MS	By MS/MS	By MS/MS						By MS/MS	8,1	83,55	0	21,47
O15305	O15305	Phosphomannomutase 2	PMM2	6	6	6	By MS/MS	By MS/MS	By MS/MS	By MS/MS	By MS/MS	By MS/MS	By MS/MS	By MS/MS	By MS/MS	By MS/MS	By MS/MS	By MS/MS	26,0	28,08	0	11,74
Q10713	Q10713	Mitochondrial-processing peptidase subunit alpha	PMPCA	10	10	10	By MS/MS	By MS/MS	By MS/MS	By MS/MS	By MS/MS	By MS/MS		By MS/MS			By MS/MS	By MS/MS	30,3	58,25	0	26,05
O75439	O75439	Mitochondrial-processing peptidase subunit beta	PMPCB	11	11	11	By MS/MS	By MS/MS	By MS/MS	By MS/MS	By MS/MS	By MS/MS		By MS/MS	By MS/MS	By MS/MS		By MS/MS	31,5	54,37	0	71,49
Q15126	Q15126	Phosphomevalonate kinase	PMVK	6	6	6	By MS/MS	By MS/MS	By MS/MS	By MS/MS	By MS/MS	By MS/MS	By MS/MS	By MS/MS	By MS/MS	By MS/MS	By MS/MS	By MS/MS	37,0	22,00	0	23,16
Q9NRX1	Q9NRX1	RNA-binding protein PNO1	PNO1	4	4	4	By MS/MS	By MS/MS		By MS/MS	By MS/MS		By MS/MS				By MS/MS	By MS/MS	22,2	27,92	0	11,57
P00491	P00491	Purine nucleoside phosphorylase	PNP	17	17	17	By MS/MS	By MS/MS	By MS/MS	By MS/MS	By MS/MS	By MS/MS	By MS/MS	By MS/MS	By MS/MS	By MS/MS	By MS/MS	By MS/MS	74,0	32,12	0	220,90
Q8TCS8	Q8TCS8	Polyribonucleotide nucleotidyltransferase 1, mitochondrial	PNPT1	6	6	6	By MS/MS	By MS/MS	By MS/MS	By MS/MS		By MS/MS	By MS/MS	By MS/MS	By MS/MS	By MS/MS	By MS/MS	By MS/MS	13,4	85,95	0	21,38
P28340	P28340	DNA polymerase delta catalytic subunit	POLD1	12	12	12	By MS/MS	By MS/MS	By MS/MS	By MS/MS	By MS/MS	By MS/MS					By MS/MS	By MS/MS	15,8	123,63	0	28,09
Q9Y2S7	Q9Y2S7	Polymerase delta-interacting protein 2	POLDIP2	8	8	8	By MS/MS	By MS/MS	By MS/MS	By MS/MS	By MS/MS	By MS/MS		By MS/MS	By MS/MS	By MS/MS			30,4	42,03	0	62,81
Q07864	Q07864	DNA polymerase epsilon catalytic subunit A	POLE	5	5	5		By MS/MS	By MS/MS	By MS/MS	By MS/MS	By MS/MS	By MS/MS						3,9	261,51	0	17,06
Q9NRF9	Q9NRF9	DNA polymerase epsilon subunit 3	POLE3	5	5	5	By MS/MS	By MS/MS	By MS/MS	By MS/MS	By MS/MS	By MS/MS	By MS/MS	By MS/MS	By MS/MS	By MS/MS	By MS/MS	By MS/MS	45,6	16,86	0	44,20
O15160	O15160	DNA-directed RNA polymerases I and III subunit RPAC1	POLR1C	7	7	7	By MS/MS	By MS/MS	By MS/MS	By MS/MS	By MS/MS	By MS/MS	By MS/MS	By MS/MS	By MS/MS	By MS/MS			28,3	39,25	0	134,14
Q9Y2S0	Q9Y2S0	DNA-directed RNA polymerases I and III subunit RPAC2	POLR1D	4	4	4	By MS/MS	By MS/MS	By MS/MS	By MS/MS	By MS/MS	By MS/MS	By MS/MS	By MS/MS	By MS/MS				45,9	15,24	0	53,48
P30876	P30876	DNA-directed RNA polymerase II subunit RPB2	POLR2B	16	16	16	By MS/MS	By MS/MS	By MS/MS	By MS/MS	By MS/MS	By MS/MS	By MS/MS	By MS/MS	By MS/MS	By MS/MS	By MS/MS		17,5	133,90	0	30,42
P62487	P62487	DNA-directed RNA polymerase II subunit RPB7	POLR2G	5	5	5	By MS/MS	By MS/MS	By MS/MS	By MS/MS	By MS/MS	By MS/MS	By MS/MS	By MS/MS	By MS/MS	By MS/MS			39,0	19,29	0	11,07
P52434	P52434	DNA-directed RNA polymerases I, II, and III subunit RPABC3	POLR2H	6	6	6	By MS/MS	By MS/MS	By MS/MS	By MS/MS	By MS/MS	By MS/MS	By MS/MS	By MS/MS	By MS/MS	By MS/MS	By MS/MS	By MS/MS	45,3	17,14	0	17,97
O14802	O14802	DNA-directed RNA polymerase III subunit RPC1	POLR3A	4	4	4	By MS/MS	By MS/MS	By MS/MS	By MS/MS	By MS/MS	By MS/MS	By MS/MS				By MS/MS		3,6	155,64	0	10,82
P16435	P16435	NADPH--cytochrome P450 reductase	POR	9	9	9	By MS/MS	By MS/MS	By MS/MS	By MS/MS	By MS/MS	By MS/MS	By MS/MS	By MS/MS	By MS/MS	By MS/MS	By MS/MS	By MS/MS	21,7	76,69	0	19,50
Q15181	Q15181	Inorganic pyrophosphatase	PPA1	22	22	22	By MS/MS	By MS/MS	By MS/MS	By MS/MS	By MS/MS	By MS/MS	By MS/MS	By MS/MS	By MS/MS	By MS/MS	By MS/MS	By MS/MS	79,2	32,66	0	323,31
Q06203	Q06203	Amidophosphoribosyltransferase	PPAT	10	10	10	By MS/MS	By MS/MS	By MS/MS	By MS/MS	By MS/MS	By MS/MS	By MS/MS	By MS/MS			By MS/MS	By MS/MS	25,7	57,40	0	57,55
P62937;P0DN26;A0A0B4J2A2;A0A075B759;Q9Y536;F5H284;P0DN37	P62937	Peptidyl-prolyl cis-trans isomerase A;Peptidyl-prolyl cis-trans isomerase A, N-terminally processed	PPIA	18	18	18	By MS/MS	By MS/MS	By MS/MS	By MS/MS	By MS/MS	By MS/MS	By MS/MS	By MS/MS	By MS/MS	By MS/MS	By MS/MS	By MS/MS	92,1	18,01	0	323,31
P23284	P23284	Peptidyl-prolyl cis-trans isomerase B	PPIB	18	18	18	By MS/MS	By MS/MS	By MS/MS	By MS/MS	By MS/MS	By MS/MS	By MS/MS	By MS/MS	By MS/MS	By MS/MS	By MS/MS	By MS/MS	61,1	23,74	0	186,59
Q08752	Q08752	Peptidyl-prolyl cis-trans isomerase D	PPID	10	10	10	By MS/MS	By MS/MS	By MS/MS	By MS/MS	By MS/MS	By MS/MS	By MS/MS	By MS/MS	By MS/MS	By MS/MS			34,1	40,76	0	40,54
P30405	P30405	Peptidyl-prolyl cis-trans isomerase F, mitochondrial	PPIF	10	10	10	By MS/MS	By MS/MS	By MS/MS	By MS/MS	By MS/MS	By MS/MS	By MS/MS	By MS/MS	By MS/MS	By MS/MS	By MS/MS	By MS/MS	62,8	22,04	0	70,07
O43447	O43447	Peptidyl-prolyl cis-trans isomerase H	PPIH	11	11	11	By MS/MS	By MS/MS	By MS/MS	By MS/MS	By MS/MS	By MS/MS	By MS/MS	By MS/MS	By MS/MS	By MS/MS	By MS/MS	By MS/MS	75,7	19,21	0	177,92
Q9Y3C6	Q9Y3C6	Peptidyl-prolyl cis-trans isomerase-like 1	PPIL1	6	6	6	By MS/MS	By MS/MS	By MS/MS	By MS/MS	By MS/MS	By MS/MS	By MS/MS	By MS/MS	By MS/MS	By MS/MS	By MS/MS	By MS/MS	37,3	18,24	0	23,73
O43314;Q6PFW1	O43314	Inositol hexakisphosphate and diphosphoinositol-pentakisphosphate kinase 2	PPIP5K2	13	13	13	By MS/MS	By MS/MS	By MS/MS	By MS/MS	By MS/MS	By MS/MS	By MS/MS	By MS/MS	By MS/MS	By MS/MS	By MS/MS	By MS/MS	14,7	140,41	0	68,56
P49593	P49593	Protein phosphatase 1F	PPM1F	15	15	15	By MS/MS	By MS/MS	By MS/MS	By MS/MS	By MS/MS	By MS/MS	By MS/MS	By MS/MS	By MS/MS	By MS/MS	By MS/MS	By MS/MS	53,1	49,83	0	118,36
O15355	O15355	Protein phosphatase 1G	PPM1G	6	6	6	By MS/MS	By MS/MS	By MS/MS	By MS/MS	By MS/MS	By MS/MS	By MS/MS	By MS/MS	By MS/MS	By MS/MS	By MS/MS	By MS/MS	17,2	59,27	0	21,86
Q9Y570	Q9Y570	Protein phosphatase methylesterase 1	PPME1	4	4	4	By MS/MS	By MS/MS	By MS/MS	By MS/MS	By MS/MS	By MS/MS		By MS/MS	By MS/MS			By MS/MS	16,3	42,32	0	14,34
P62136	P62136	Serine/threonine-protein phosphatase PP1-alpha catalytic subunit	PPP1CA	21	21	6	By MS/MS	By MS/MS	By MS/MS	By MS/MS	By MS/MS	By MS/MS	By MS/MS	By MS/MS	By MS/MS	By MS/MS	By MS/MS	By MS/MS	56,1	37,51	0	323,31
P62140	P62140	Serine/threonine-protein phosphatase PP1-beta catalytic subunit	PPP1CB	18	5	5	By MS/MS	By MS/MS	By MS/MS	By MS/MS	By MS/MS	By MS/MS	By matchin	By MS/MS	By MS/MS	By MS/MS	By MS/MS	By MS/MS	55,0	37,19	0	15,64
P36873	P36873	Serine/threonine-protein phosphatase PP1-gamma catalytic subunit	PPP1CC	17	2	2	By MS/MS	By MS/MS	By MS/MS	By MS/MS	By MS/MS	By MS/MS	By MS/MS	By MS/MS	By MS/MS	By MS/MS	By MS/MS	By MS/MS	48,3	36,98	0	22,02
P30153	P30153	Serine/threonine-protein phosphatase 2A 65 kDa regulatory subunit A alpha isoform	PPP2R1A	28	28	20	By MS/MS	By MS/MS	By MS/MS	By MS/MS	By MS/MS	By MS/MS	By MS/MS	By MS/MS	By MS/MS	By MS/MS	By MS/MS	By MS/MS	60,3	65,31	0	323,31
P30154	P30154	Serine/threonine-protein phosphatase 2A 65 kDa regulatory subunit A beta isoform	PPP2R1B	17	9	9	By MS/MS	By MS/MS	By MS/MS	By MS/MS	By MS/MS	By MS/MS	By matchin	By MS/MS	By matchin	By matchin	By matchin	By MS/MS	41,9	66,21	0	91,72
P63151;Q66LE6;Q00005;Q9Y2T4	P63151	Serine/threonine-protein phosphatase 2A 55 kDa regulatory subunit B alpha isoform	PPP2R2A	8	8	8	By MS/MS	By MS/MS	By MS/MS	By MS/MS	By MS/MS	By MS/MS	By MS/MS	By MS/MS			By MS/MS	By MS/MS	26,8	51,69	0	82,61
Q15257	Q15257	Serine/threonine-protein phosphatase 2A activator	PPP2R4	7	7	7	By MS/MS	By MS/MS	By MS/MS	By MS/MS	By MS/MS	By MS/MS	By MS/MS	By MS/MS	By MS/MS	By MS/MS		By MS/MS	26,0	40,67	0	37,96
Q14738	Q14738	Serine/threonine-protein phosphatase 2A 56 kDa regulatory subunit delta isoform	PPP2R5D	5	5	4		By MS/MS	By MS/MS	By MS/MS	By MS/MS	By MS/MS					By MS/MS	By MS/MS	8,0	69,99	0	17,63

Q08209;P16298;P48454	Q08209;P16298	Serine/threonine-protein phosphatase 2B catalytic subunit alpha isoform;Serine/threonine-protein phosphatase 2B catalytic subunit beta isoform	PPP3CA;PPP3CB	8	8	8	By MS/MS	By MS/MS	By MS/MS	By MS/MS	By MS/MS	By MS/MS	By MS/MS				By MS/MS	By MS/MS	21,9	58,69	0	16,32
P63098	P63098	Calcineurin subunit B type 1	PPP3R1	4	4	4	By MS/MS	By MS/MS		By MS/MS				By MS/MS	By MS/MS	By MS/MS	By MS/MS	By MS/MS	34,1	19,30	0	20,46
P60510	P60510	Serine/threonine-protein phosphatase 4 catalytic subunit	PPP4C	7	5	5	By MS/MS	By MS/MS	By MS/MS	By MS/MS	By MS/MS	By MS/MS				By MS/MS	By MS/MS		25,4	35,08	0	104,00
P53041	P53041	Serine/threonine-protein phosphatase 5	PPP5C	7	7	7	By MS/MS	By MS/MS	By MS/MS	By MS/MS	By MS/MS	By MS/MS	By MS/MS	By MS/MS			By MS/MS	By MS/MS	21,4	56,88	0	235,18
O00743	O00743	Serine/threonine-protein phosphatase 6 catalytic subunit;Serine/threonine-protein phosphatase 6 catalytic subunit, N-terminally processed	PPP6C	8	8	8	By MS/MS	By MS/MS	By MS/MS	By MS/MS	By MS/MS	By MS/MS			By MS/MS	By MS/MS	By MS/MS	By MS/MS	31,8	35,14	0	213,12
P50897	P50897	Palmitoyl-protein thioesterase 1	PPT1	7	7	7	By MS/MS	By MS/MS	By MS/MS	By MS/MS	By MS/MS	By MS/MS	By MS/MS	By MS/MS	By MS/MS	By MS/MS	By MS/MS	By MS/MS	33,0	34,19	0	259,12
Q06830	Q06830	Peroxiredoxin-1	PRDX1	24	24	21	By MS/MS	By MS/MS	By MS/MS	By MS/MS	By MS/MS	By MS/MS	By MS/MS	By MS/MS	By MS/MS	By MS/MS	By MS/MS	By MS/MS	76,4	22,11	0	323,31
P32119	P32119	Peroxiredoxin-2	PRDX2	17	16	16	By MS/MS	By MS/MS	By MS/MS	By MS/MS	By MS/MS	By MS/MS	By MS/MS	By MS/MS	By MS/MS	By MS/MS	By MS/MS	By MS/MS	73,7	21,89	0	117,57
P30048	P30048	Thioredoxin-dependent peroxide reductase, mitochondrial	PRDX3	9	9	9	By MS/MS	By MS/MS	By MS/MS	By MS/MS	By MS/MS	By MS/MS	By MS/MS	By MS/MS	By MS/MS	By MS/MS	By MS/MS	By MS/MS	43,8	27,69	0	116,20
Q13162	Q13162	Peroxiredoxin-4	PRDX4	15	13	13	By MS/MS	By MS/MS	By MS/MS	By MS/MS	By MS/MS	By MS/MS	By MS/MS	By MS/MS	By MS/MS	By MS/MS	By MS/MS	By MS/MS	63,1	30,54	0	181,24
P30044	P30044	Peroxiredoxin-5, mitochondrial	PRDX5	12	12	12	By MS/MS	By MS/MS	By MS/MS	By MS/MS	By MS/MS	By MS/MS	By MS/MS	By MS/MS	By MS/MS	By MS/MS	By MS/MS	By MS/MS	57,0	22,09	0	212,01
P30041	P30041	Peroxiredoxin-6	PRDX6	26	26	26	By MS/MS	By MS/MS	By MS/MS	By MS/MS	By MS/MS	By MS/MS	By MS/MS	By MS/MS	By MS/MS	By MS/MS	By MS/MS	By MS/MS	82,6	25,04	0	323,31
P48147	P48147	Prolyl endopeptidase	PREP	8	8	8	By MS/MS	By MS/MS	By MS/MS	By MS/MS	By MS/MS	By MS/MS	By MS/MS	By MS/MS	By MS/MS	By MS/MS			18,2	80,70	0	302,13
P54619	P54619	5-AMP-activated protein kinase subunit gamma-1	PRKAG1	6	6	5	By MS/MS	By MS/MS		By MS/MS	By MS/MS	By MS/MS	By MS/MS	By MS/MS	By MS/MS	By MS/MS			25,7	37,58	0	230,11
P31323	P31323	cAMP-dependent protein kinase type II-beta regulatory subunit	PRKAR2B	6	5	5		By MS/MS	By MS/MS	By matchin	By MS/MS			By MS/MS				By MS/MS	21,8	46,30	0	15,49
P05771;P05129	P05771	Protein kinase C beta type	PRKCB	8	8	5	By MS/MS	By MS/MS	By MS/MS	By MS/MS	By MS/MS	By MS/MS	By MS/MS	By MS/MS	By MS/MS	By MS/MS	By MS/MS	By MS/MS	17,6	76,87	0	142,90
P14314	P14314	Glucosidase 2 subunit beta	PRKCSH	11	11	11	By MS/MS	By MS/MS	By MS/MS	By MS/MS	By MS/MS	By MS/MS	By MS/MS	By MS/MS	By MS/MS	By MS/MS	By MS/MS	By MS/MS	25,6	59,43	0	42,06
P78527	P78527	DNA-dependent protein kinase catalytic subunit	PRKDC	164	164	164	By MS/MS	By MS/MS	By MS/MS	By MS/MS	By MS/MS	By MS/MS	By MS/MS	By MS/MS	By MS/MS	By MS/MS	By MS/MS	By MS/MS	48,3	469,08	0	323,31
Q99873;Q9NR22	Q99873	Protein arginine N-methyltransferase 1	PRMT1	17	17	17	By MS/MS	By MS/MS	By MS/MS	By MS/MS	By MS/MS	By MS/MS	By MS/MS	By MS/MS	By MS/MS	By MS/MS	By MS/MS	By MS/MS	49,9	41,52	0	181,46
O14744	O14744	Protein arginine N-methyltransferase 5;Protein arginine N-methyltransferase 5, N-terminally processed	PRMT5	12	12	12	By MS/MS	By MS/MS	By MS/MS	By MS/MS	By MS/MS	By MS/MS	By MS/MS	By MS/MS	By MS/MS	By MS/MS	By MS/MS	By MS/MS	30,6	72,68	0	60,28
O94903	O94903	Proline synthase co-transcribed bacterial homolog protein	PROSC	5	5	5	By MS/MS	By MS/MS	By MS/MS	By MS/MS	By MS/MS	By MS/MS	By MS/MS	By MS/MS	By MS/MS	By MS/MS	By MS/MS	By MS/MS	28,0	30,34	0	16,77
Q9UMS4	Q9UMS4	Pre-mRNA-processing factor 19	PRPF19	13	13	13	By MS/MS	By MS/MS	By MS/MS	By MS/MS	By MS/MS	By MS/MS	By MS/MS	By MS/MS	By MS/MS	By MS/MS	By MS/MS	By MS/MS	47,8	55,18	0	104,92
Q8WWY3	Q8WWY3	U4/U6 small nuclear ribonucleoprotein Prp31	PRPF31	4	4	4	By MS/MS		By MS/MS	By MS/MS		By MS/MS				By MS/MS			15,8	55,46	0	11,15
O43172	O43172	U4/U6 small nuclear ribonucleoprotein Prp4	PRPF4	7	7	7				By MS/MS		By MS/MS					By MS/MS	By MS/MS	20,1	58,45	0	25,57
Q6P2Q9	Q6P2Q9	Pre-mRNA-processing-splicing factor 8	PRPF8	52	52	52	By MS/MS	By MS/MS	By MS/MS	By MS/MS	By MS/MS	By MS/MS	By MS/MS	By MS/MS	By MS/MS	By MS/MS	By MS/MS	By MS/MS	32,2	273,60	0	323,31
P60891	P60891	Ribose-phosphate pyrophosphokinase 1	PRPS1	14	14	4	By MS/MS	By MS/MS	By MS/MS	By MS/MS	By MS/MS	By MS/MS	By MS/MS	By MS/MS	By MS/MS	By MS/MS	By MS/MS	By MS/MS	47,2	34,83	0	90,96
P11908	P11908	Ribose-phosphate pyrophosphokinase 2	PRPS2	14	6	5	By matchin	By MS/MS	By MS/MS	By MS/MS	By MS/MS	By MS/MS		By MS/MS	By MS/MS	By MS/MS	By matchin	By MS/MS	48,4	34,77	0	12,09
Q14558	Q14558	Phosphoribosyl pyrophosphate synthase-associated protein 1	PRPSAP1	6	6	6	By MS/MS	By MS/MS	By MS/MS	By MS/MS	By MS/MS	By MS/MS	By MS/MS	By MS/MS	By MS/MS	By MS/MS	By MS/MS	By MS/MS	24,7	39,39	0	13,19
Q96M27	Q96M27	Protein PRRC1	PRRC1	4	4	4	By MS/MS	By MS/MS	By MS/MS		By MS/MS	By MS/MS			By MS/MS	By MS/MS	By MS/MS		13,3	46,70	0	78,26
Q9Y617	Q9Y617	Phosphoserine aminotransferase	PSAT1	19	19	19	By MS/MS	By MS/MS	By MS/MS	By MS/MS	By MS/MS	By MS/MS	By MS/MS	By MS/MS	By MS/MS	By MS/MS			58,1	40,42	0	148,18
P25786	P25786	Proteasome subunit alpha type-1	PSMA1	11	11	11	By MS/MS	By MS/MS	By MS/MS	By MS/MS	By MS/MS	By MS/MS	By MS/MS	By MS/MS	By MS/MS	By MS/MS	By MS/MS	By MS/MS	43,3	29,56	0	144,85
P25787	P25787	Proteasome subunit alpha type-2	PSMA2	12	12	12	By MS/MS	By MS/MS	By MS/MS	By MS/MS	By MS/MS	By MS/MS	By MS/MS	By MS/MS	By MS/MS	By MS/MS	By MS/MS	By MS/MS	59,4	25,90	0	114,87
P25788	P25788	Proteasome subunit alpha type-3	PSMA3	10	10	10	By MS/MS	By MS/MS	By MS/MS	By MS/MS	By MS/MS	By MS/MS	By MS/MS	By MS/MS	By MS/MS	By MS/MS	By MS/MS	By MS/MS	43,9	28,43	0	174,74
P25789	P25789	Proteasome subunit alpha type-4	PSMA4	10	10	10	By MS/MS	By MS/MS	By MS/MS	By MS/MS	By MS/MS	By MS/MS	By MS/MS	By MS/MS	By MS/MS	By MS/MS	By MS/MS	By MS/MS	51,7	29,48	0	189,42
P28066	P28066	Proteasome subunit alpha type-5	PSMA5	13	13	13	By MS/MS	By MS/MS	By MS/MS	By MS/MS	By MS/MS	By MS/MS	By MS/MS	By MS/MS	By MS/MS	By MS/MS	By MS/MS	By MS/MS	71,4	26,41	0	323,31
P60900	P60900	Proteasome subunit alpha type-6	PSMA6	13	13	13	By MS/MS	By MS/MS	By MS/MS	By MS/MS	By MS/MS	By MS/MS	By MS/MS	By MS/MS	By MS/MS	By MS/MS	By MS/MS	By MS/MS	54,1	27,40	0	323,31
O14818;Q8TAA3	O14818	Proteasome subunit alpha type-7	PSMA7	17	17	17	By MS/MS	By MS/MS	By MS/MS	By MS/MS	By MS/MS	By MS/MS	By MS/MS	By MS/MS	By MS/MS	By MS/MS	By MS/MS	By MS/MS	64,5	27,89	0	323,31
P20618	P20618	Proteasome subunit beta type-1	PSMB1	15	15	15	By MS/MS	By MS/MS	By MS/MS	By MS/MS	By MS/MS	By MS/MS	By MS/MS	By MS/MS	By MS/MS	By MS/MS	By MS/MS	By MS/MS	61,4	26,49	0	323,31
P49721	P49721	Proteasome subunit beta type-2	PSMB2	13	13	13	By MS/MS	By MS/MS	By MS/MS	By MS/MS	By MS/MS	By MS/MS	By MS/MS	By MS/MS	By MS/MS	By MS/MS	By MS/MS	By MS/MS	68,7	22,84	0	259,15
P49720	P49720	Proteasome subunit beta type-3	PSMB3	11	11	11	By MS/MS	By MS/MS	By MS/MS	By MS/MS	By MS/MS	By MS/MS	By MS/MS	By MS/MS	By MS/MS	By MS/MS	By MS/MS	By MS/MS	53,7	22,95	0	151,56
P28070	P28070	Proteasome subunit beta type-4	PSMB4	9	9	9	By MS/MS	By MS/MS	By MS/MS	By MS/MS	By MS/MS	By MS/MS	By MS/MS	By MS/MS	By MS/MS	By MS/MS	By MS/MS	By MS/MS	50,4	29,20	0	229,25
P28074	P28074	Proteasome subunit beta type-5	PSMB5	16	16	16	By MS/MS	By MS/MS	By MS/MS	By MS/MS	By MS/MS	By MS/MS	By MS/MS	By MS/MS	By MS/MS	By MS/MS	By MS/MS	By MS/MS	68,4	28,48	0	323,31
P28072	P28072	Proteasome subunit beta type-6	PSMB6	5	5	5	By MS/MS	By MS/MS	By MS/MS	By MS/MS	By MS/MS	By MS/MS	By MS/MS	By MS/MS	By MS/MS	By MS/MS	By MS/MS	By MS/MS	26,8	25,36	0	10,12
Q99436	Q99436	Proteasome subunit beta type-7	PSMB7	8	8	7	By MS/MS	By MS/MS	By MS/MS	By MS/MS	By MS/MS	By MS/MS	By MS/MS	By MS/MS	By MS/MS	By MS/MS	By MS/MS	By MS/MS	30,7	29,97	0	209,47
P62191	P62191	26S protease regulatory subunit 4	PSMC1	14	14	14	By MS/MS	By MS/MS	By MS/MS	By MS/MS	By MS/MS	By MS/MS	By MS/MS	By MS/MS			By MS/MS	By MS/MS	40,0	49,18	0	323,31
P35998	P35998	26S protease regulatory subunit 7	PSMC2	23	23	23	By MS/MS	By MS/MS	By MS/MS	By MS/MS	By MS/MS	By MS/MS	By MS/MS	By MS/MS			By MS/MS	By MS/MS	60,3	48,63	0	150,92
P17980	P17980	26S protease regulatory subunit 6A	PSMC3	19	19	19	By MS/MS	By MS/MS	By MS/MS	By MS/MS	By MS/MS	By MS/MS	By MS/MS	By MS/MS	By MS/MS		By MS/MS	By MS/MS	44,9	49,20	0	248,50
P43686	P43686	26S protease regulatory subunit 6B	PSMC4	16	16	16	By MS/MS	By MS/MS	By MS/MS	By MS/MS	By MS/MS	By MS/MS		By MS/MS			By MS/MS	By MS/MS	56,7	47,37	0	277,39
P62195	P62195	26S protease regulatory subunit 8	PSMC5	19	19	18	By MS/MS	By MS/MS	By MS/MS	By MS/MS	By MS/MS	By MS/MS	By MS/MS	By MS/MS	By MS/MS	By MS/MS	By MS/MS	By MS/MS	61,6	45,63	0	157,40
P62333	P62333	26S protease regulatory subunit 10B	PSMC6	19	19	19	By MS/MS	By MS/MS	By MS/MS	By MS/MS	By MS/MS	By MS/MS	By MS/MS	By MS/MS	By MS/MS	By MS/MS	By MS/MS	By MS/MS	64,5	44,17	0	119,47
Q99460	Q99460	26S proteasome non-ATPase regulatory subunit 1	PSMD1	29	29	29	By MS/MS	By MS/MS	By MS/MS	By MS/MS	By MS/MS	By MS/MS	By MS/MS	By MS/MS	By MS/MS	By MS/MS	By MS/MS	By MS/MS	44,6	105,84	0	287,31
O75832	O75832	26S proteasome non-ATPase regulatory subunit 10	PSMD10	9	9	9	By MS/MS	By MS/MS	By MS/MS	By MS/MS	By MS/MS	By MS/MS	By MS/MS	By MS/MS	By MS/MS	By MS/MS	By MS/MS	By MS/MS	48,7	24,43	0	90,81
O00231	O00231	26S proteasome non-ATPase regulatory subunit 11	PSMD11	24	24	24	By MS/MS	By MS/MS	By MS/MS	By MS/MS	By MS/MS	By MS/MS	By MS/MS	By MS/MS	By MS/MS	By MS/MS	By MS/MS	By MS/MS	59,5	47,46	0	142,61
O00232	O00232	26S proteasome non-ATPase regulatory subunit 12	PSMD12	16	16	16	By MS/MS	By MS/MS	By MS/MS	By MS/MS	By MS/MS	By MS/MS	By MS/MS	By MS/MS	By MS/MS	By MS/MS	By MS/MS	By MS/MS	48,5	52,90	0	310,83
Q9UNM6	Q9UNM6	26S proteasome non-ATPase regulatory subunit 13	PSMD13	25	25	25	By MS/MS	By MS/MS	By MS/MS	By MS/MS	By MS/MS	By MS/MS	By MS/MS	By MS/MS	By MS/MS	By MS/MS			76,1	42,95	0	323,31
O00487	O00487	26S proteasome non-ATPase regulatory subunit 14	PSMD14	10	10	10	By MS/MS	By MS/MS	By MS/MS	By MS/MS	By MS/MS	By MS/MS	By MS/MS	By MS/MS	By MS/MS	By MS/MS	By MS/MS	By MS/MS	53,9	34,58	0	94,05
Q13200	Q13200	26S proteasome non-ATPase regulatory subunit 2	PSMD2	29	29	29	By MS/MS	By MS/MS	By MS/MS	By MS/MS	By MS/MS	By MS/MS	By MS/MS	By MS/MS	By MS/MS	By MS/MS	By MS/MS	By MS/MS	47,1	100,20	0	323,31
O43242	O43242	26S proteasome non-ATPase regulatory subunit 3	PSMD3	12	12	12	By MS/MS	By MS/MS	By MS/MS	By MS/MS	By MS/MS	By MS/MS	By MS/MS	By MS/MS	By MS/MS	By MS/MS	By MS/MS	By MS/MS	30,3	60,98	0	97,84
P55036	P55036	26S proteasome non-ATPase regulatory subunit 4	PSMD4	5	5	5	By MS/MS	By MS/MS	By MS/MS	By MS/MS	By MS/MS	By MS/MS	By MS/MS	By MS/MS			By MS/MS	By MS/MS	25,5	40,74	0	20,20
Q16401	Q16401	26S proteasome non-ATPase regulatory subunit 5	PSMD5	21	21	21	By MS/MS	By MS/MS	By MS/MS	By MS/MS	By MS/MS	By MS/MS	By MS/MS	By MS/MS	By MS/MS	By MS/MS	By MS/MS	By MS/MS	54,8	56,20	0	266,74
Q15008	Q15008	26S proteasome non-ATPase regulatory subunit 6	PSMD6	25	25	25	By MS/MS	By MS/MS	By MS/MS	By MS/MS	By MS/MS	By MS/MS	By MS/MS	By MS/MS	By MS/MS	By MS/MS	By MS/MS	By MS/MS	66,8	45,53	0	84,29
P51665	P51665	26S proteasome non-ATPase regulatory subunit 7	PSMD7	14	14	14	By MS/MS	By MS/MS	By MS/MS	By MS/MS	By MS/MS	By MS/MS	By MS/MS	By MS/MS	By MS/MS	By MS/MS			50,6	37,03	0	168,76
P48556	P48556	26S proteasome non-ATPase regulatory subunit 8	PSMD8	12	12	12	By MS/MS	By MS/MS	By MS/MS	By MS/MS	By MS/MS	By MS/MS		By MS/MS	By MS/MS	By MS/MS		By MS/MS	35,1	39,61	0	47,12
O00233	O00233	26S proteasome non-ATPase regulatory subunit 9	PSMD9	6	6	6	By MS/MS	By MS/MS	By MS/MS	By MS/MS	By MS/MS	By MS/MS			By MS/MS	By MS/MS		By MS/MS	31,4	24,68	0	10,60
Q06323	Q06323	Proteasome activator complex subunit 1	PSME1	10	10	10	By MS/MS	By MS/MS	By MS/MS	By MS/MS	By MS/MS	By MS/MS	By MS/MS	By MS/MS	By MS/MS	By MS/MS	By MS/MS	By MS/MS	41,4	28,72	0	74,88
Q9UL46	Q9UL46	Proteasome activator complex subunit 2	PSME2	8	8	8	By MS/MS	By MS/MS	By MS/MS	By MS/MS	By MS/MS	By MS/MS		By MS/MS		By MS/MS			51,0	27,40	0	83,10
P61289	P61289	Proteasome activator complex subunit 3	PSME3	12	12	12	By MS/MS	By MS/MS	By MS/MS	By MS/MS	By MS/MS	By MS/MS	By MS/MS	By MS/MS	By MS/MS	By MS/MS	By MS/MS	By MS/MS	51,6	29,51	0	314,00

Q14997	Q14997	Proteasome activator complex subunit 4	PSME4	8	8	8	By MS/MS	By MS/MS	By MS/MS	By MS/MS	By MS/MS	By MS/MS	By MS/MS	By MS/MS	By MS/MS	By MS/MS			7,3	211,33	0	25,14
O95456	O95456	Proteasome assembly chaperone 1	PSMG1	7	7	7	By MS/MS	By MS/MS	By MS/MS	By MS/MS	By MS/MS	By MS/MS	By MS/MS	By MS/MS	By MS/MS	By MS/MS	By MS/MS		33,7	32,85	0	36,54
Q969U7	Q969U7	Proteasome assembly chaperone 2	PSMG2	6	6	6	By MS/MS	By MS/MS	By MS/MS	By MS/MS	By MS/MS	By MS/MS	By MS/MS		By MS/MS	By MS/MS	By MS/MS	By MS/MS	20,5	29,40	0	42,46
Q9BT73	Q9BT73	Proteasome assembly chaperone 3	PSMG3	6	6	6	By MS/MS	By MS/MS	By MS/MS		By MS/MS	By MS/MS		By MS/MS	By MS/MS	By MS/MS	By MS/MS	By MS/MS	59,8	13,10	0	17,94
Q8WXF1	Q8WXF1	Paraspeckle component 1	PSPC1	6	6	6	By MS/MS	By MS/MS	By MS/MS	By MS/MS	By MS/MS	By MS/MS	By MS/MS	By MS/MS	By MS/MS	By MS/MS	By MS/MS	By MS/MS	16,8	58,74	0	21,78
P26599	P26599	Polypyrimidine tract-binding protein 1	PTBP1	20	20	19	By MS/MS	By MS/MS	By MS/MS	By MS/MS	By MS/MS	By MS/MS	By MS/MS	By MS/MS	By MS/MS	By MS/MS	By MS/MS	By MS/MS	59,7	57,22	0	323,31
Q96EY7	Q96EY7	Pentatricopeptide repeat domain-containing protein 3, mitochondrial	PTCD3	9	9	9	By MS/MS	By MS/MS		By MS/MS	By MS/MS	By MS/MS	By MS/MS	By MS/MS	By MS/MS	By MS/MS	By MS/MS	By MS/MS	23,1	78,55	0	34,78
Q9H7Z7	Q9H7Z7	Prostaglandin E synthase 2;Prostaglandin E synthase 2 truncated form	PTGES2	4	4	4	By MS/MS	By MS/MS	By MS/MS	By MS/MS	By MS/MS	By MS/MS			By MS/MS	By MS/MS	By MS/MS	By MS/MS	21,2	41,94	0	73,28
Q15185	Q15185	Prostaglandin E synthase 3	PTGES3	9	9	9	By MS/MS	By MS/MS	By MS/MS	By MS/MS	By MS/MS	By MS/MS	By MS/MS	By MS/MS	By MS/MS	By MS/MS	By MS/MS	By MS/MS	52,5	18,70	0	323,31
P06454	P06454	Prothymosin alpha;Prothymosin alpha, N-terminally processed;Thymosin alpha-1	PTMA	5	5	5	By MS/MS	By MS/MS	By MS/MS	By MS/MS	By MS/MS	By MS/MS	By MS/MS	By MS/MS	By MS/MS	By MS/MS	By MS/MS	By MS/MS	38,7	12,20	0	323,31
Q12974;Q93096	Q12974	Protein tyrosine phosphatase type IVA 2	PTP4A2	5	5	5		By MS/MS	By MS/MS	By MS/MS	By MS/MS		By MS/MS	By MS/MS	By MS/MS	By MS/MS			36,5	19,13	0	12,46
Q8WUK0	Q8WUK0	Phosphatidylglycerophosphatase and protein-tyrosine phosphatase 1	PTPMT1	5	5	5	By MS/MS	By MS/MS	By MS/MS	By MS/MS	By MS/MS	By MS/MS	By MS/MS				By MS/MS	By MS/MS	33,8	22,84	0	15,69
P18031	P18031	Tyrosine-protein phosphatase non-receptor type 1	PTPN1	5	5	5	By MS/MS	By MS/MS	By MS/MS	By MS/MS	By MS/MS	By MS/MS	By MS/MS		By MS/MS	By MS/MS			13,8	49,97	0	22,35
P08575	P08575	Receptor-type tyrosine-protein phosphatase C	PTPRC	7	7	7	By MS/MS	By MS/MS	By MS/MS	By MS/MS	By MS/MS	By MS/MS		By MS/MS	By MS/MS	By MS/MS			8,2	147,25	0	11,18
Q6NZI2	Q6NZI2	Polymerase I and transcript release factor	PTRF	7	7	7	By MS/MS	By MS/MS			By MS/MS	By MS/MS		By MS/MS				By MS/MS	24,4	43,48	0	153,12
Q9Y3E5	Q9Y3E5	Peptidyl-tRNA hydrolase 2, mitochondrial	PTRH2	7	7	7	By MS/MS	By MS/MS	By MS/MS	By MS/MS	By MS/MS	By MS/MS	By MS/MS	By MS/MS	By MS/MS	By MS/MS	By MS/MS	By MS/MS	53,1	19,19	0	97,27
Q6GMV3	Q6GMV3	Putative peptidyl-tRNA hydrolase PTRHD1	PTRHD1	5	5	5	By MS/MS	By MS/MS	By MS/MS	By MS/MS	By MS/MS	By MS/MS	By MS/MS			By MS/MS			48,6	15,81	0	10,30
Q9UHX1	Q9UHX1	Poly(U)-binding-splicing factor PUF60	PUF60	15	15	15	By MS/MS	By MS/MS	By MS/MS	By MS/MS	By MS/MS	By MS/MS	By MS/MS	By MS/MS	By MS/MS	By MS/MS	By MS/MS	By MS/MS	42,8	59,88	0	230,10
Q9Y606	Q9Y606	tRNA pseudouridine synthase A, mitochondrial	PUS1	7	7	7	By MS/MS	By MS/MS	By MS/MS	By MS/MS	By MS/MS	By MS/MS	By MS/MS	By MS/MS	By MS/MS	By MS/MS			24,1	47,47	0	15,71
Q96PZ0	Q96PZ0	Pseudouridylate synthase 7 homolog	PUS7	4	4	4		By MS/MS		By MS/MS		By MS/MS	By MS/MS						13,2	75,04	0	15,53
P32322	P32322	Pyrroline-5-carboxylate reductase 1, mitochondrial	PYCR1	14	13	13	By MS/MS	By MS/MS	By MS/MS	By MS/MS	By MS/MS	By MS/MS	By MS/MS	By MS/MS	By MS/MS	By MS/MS	By MS/MS	By MS/MS	49,8	33,36	0	175,53
Q96C36	Q96C36	Pyrroline-5-carboxylate reductase 2	PYCR2	14	14	13	By MS/MS	By MS/MS	By MS/MS	By MS/MS	By MS/MS	By MS/MS	By MS/MS	By MS/MS	By MS/MS	By MS/MS	By MS/MS	By MS/MS	48,8	33,64	0	323,31
P47897	P47897	Glutamine--tRNA ligase	QARS	14	14	14	By MS/MS	By MS/MS	By MS/MS	By MS/MS	By MS/MS	By MS/MS	By MS/MS	By MS/MS	By MS/MS	By MS/MS	By MS/MS	By MS/MS	22,7	87,80	0	124,87
P09417	P09417	Dihydropteridine reductase	QDPR	8	8	8	By MS/MS	By MS/MS	By MS/MS	By MS/MS	By MS/MS	By MS/MS	By MS/MS	By MS/MS			By MS/MS	By MS/MS	43,9	25,79	0	323,31
Q5XKP0	Q5XKP0	Protein QIL1	QIL1	4	4	4	By MS/MS	By MS/MS			By MS/MS		By MS/MS	By MS/MS	By MS/MS	By MS/MS	By MS/MS	By MS/MS	72,0	13,09	0	102,59
Q96PU8	Q96PU8	Protein quaking	QKI	4	4	4	By MS/MS	By MS/MS	By MS/MS	By MS/MS	By MS/MS	By MS/MS				By MS/MS	By MS/MS	By MS/MS	17,9	37,67	0	5,99
Q15274	Q15274	Nicotinate-nucleotide pyrophosphorylase [carboxylating]	QPRT	5	5	5			By MS/MS	By MS/MS	By MS/MS	By MS/MS	By MS/MS	By MS/MS	By MS/MS	By MS/MS	By MS/MS	By MS/MS	24,9	30,85	0	17,43
Q9BXR0	Q9BXR0	Queuine tRNA-ribosyltransferase	QTRT1	4	4	4	By MS/MS	By MS/MS		By MS/MS		By MS/MS			By MS/MS				20,8	44,05	0	4,95
P61026	P61026	Ras-related protein Rab-10	RAB10	12	11	11	By MS/MS	By MS/MS	By MS/MS	By MS/MS	By MS/MS	By MS/MS	By MS/MS	By MS/MS	By MS/MS	By MS/MS	By MS/MS	By MS/MS	55,0	22,54	0	63,53
Q15907	Q15907	Ras-related protein Rab-11B	RAB11B	12	12	2	By MS/MS	By MS/MS	By MS/MS	By MS/MS	By MS/MS	By MS/MS	By MS/MS	By MS/MS	By MS/MS	By MS/MS	By MS/MS	By MS/MS	59,2	24,49	0	204,46
P51153;P59190	P51153	Ras-related protein Rab-13	RAB13	4	3	3	By MS/MS	By MS/MS	By MS/MS	By MS/MS	By MS/MS	By MS/MS	By matchin	By matchin	By matchin	By matchin	By matchin	By matchin	25,1	22,77	0	10,14
P61106	P61106	Ras-related protein Rab-14	RAB14	14	14	14	By MS/MS	By MS/MS	By MS/MS	By MS/MS	By MS/MS	By MS/MS	By MS/MS	By MS/MS	By MS/MS	By MS/MS	By MS/MS	By MS/MS	78,1	23,90	0	133,90
Q9NP72	Q9NP72	Ras-related protein Rab-18	RAB18	7	7	7	By MS/MS	By MS/MS	By MS/MS	By MS/MS	By MS/MS	By MS/MS	By MS/MS	By MS/MS	By MS/MS	By MS/MS	By MS/MS	By MS/MS	46,1	22,98	0	93,05
P62820	P62820	Ras-related protein Rab-1A	RAB1A	10	5	5	By MS/MS	By MS/MS	By MS/MS	By MS/MS	By MS/MS	By MS/MS	By MS/MS	By MS/MS	By MS/MS	By MS/MS	By MS/MS	By MS/MS	64,9	22,68	0	274,46
Q9H0U4;Q92928	Q9H0U4;Q92928	Ras-related protein Rab-1B;Putative Ras-related protein Rab-1C	RAB1B;RAB1C	12	12	7	By MS/MS	By MS/MS	By MS/MS	By MS/MS	By MS/MS	By MS/MS	By MS/MS	By MS/MS	By MS/MS	By MS/MS	By MS/MS	By MS/MS	72,6	22,17	0	281,71
Q9UL25	Q9UL25	Ras-related protein Rab-21	RAB21	9	9	9	By MS/MS	By MS/MS	By MS/MS	By MS/MS	By MS/MS	By MS/MS	By MS/MS	By MS/MS	By MS/MS	By MS/MS	By MS/MS	By MS/MS	46,2	24,35	0	56,68
Q9UL26	Q9UL26	Ras-related protein Rab-22A	RAB22A	4	2	2	By MS/MS	By MS/MS	By MS/MS	By MS/MS	By matching		By matchin	By matching					29,4	21,86	0	12,93
P51159	P51159	Ras-related protein Rab-27A	RAB27A	7	7	5	By MS/MS	By MS/MS	By MS/MS	By MS/MS	By MS/MS	By MS/MS	By MS/MS	By MS/MS				By MS/MS	36,2	24,87	0	23,26
P61019	P61019	Ras-related protein Rab-2A	RAB2A	16	16	8	By MS/MS	By MS/MS	By MS/MS	By MS/MS	By MS/MS	By MS/MS	By MS/MS	By MS/MS	By MS/MS	By MS/MS	By MS/MS	By MS/MS	74,1	23,55	0	227,77
Q13636	Q13636	Ras-related protein Rab-31	RAB31	8	8	6	By MS/MS	By MS/MS		By MS/MS	By MS/MS	By MS/MS	By MS/MS	By MS/MS					43,3	21,57	0	38,44
P20339	P20339	Ras-related protein Rab-5A	RAB5A	8	5	5	By MS/MS	By MS/MS	By MS/MS	By MS/MS	By MS/MS	By MS/MS	By MS/MS	By MS/MS	By MS/MS	By MS/MS	By MS/MS	By MS/MS	53,5	23,66	0	323,31
P61020	P61020	Ras-related protein Rab-5B	RAB5B	6	3	3	By MS/MS	By MS/MS	By MS/MS	By MS/MS	By MS/MS	By MS/MS	By matchin	By matchin	By MS/MS	By matchin	By matchin	By MS/MS	36,7	23,71	0	102,61
P51148	P51148	Ras-related protein Rab-5C	RAB5C	8	8	5	By MS/MS	By MS/MS	By MS/MS	By MS/MS	By MS/MS	By MS/MS	By MS/MS	By MS/MS	By MS/MS	By MS/MS	By MS/MS	By MS/MS	53,2	23,48	0	323,31
P20340	P20340	Ras-related protein Rab-6A	RAB6A	10	10	5	By MS/MS	By MS/MS	By MS/MS	By MS/MS	By MS/MS	By MS/MS	By MS/MS	By MS/MS	By MS/MS	By MS/MS	By MS/MS	By MS/MS	50,0	23,59	0	136,29
P51149	P51149	Ras-related protein Rab-7a	RAB7A	16	16	16	By MS/MS	By MS/MS	By MS/MS	By MS/MS	By MS/MS	By MS/MS	By MS/MS	By MS/MS	By MS/MS	By MS/MS	By MS/MS	By MS/MS	75,4	23,49	0	323,31
P61006	P61006	Ras-related protein Rab-8A	RAB8A	5	4	3	By MS/MS	By MS/MS	By MS/MS	By MS/MS	By MS/MS	By MS/MS	By matchin	By MS/MS	By MS/MS	By MS/MS	By matchin	By matchin	29,0	23,67	0	122,62
P63000	P63000	Ras-related C3 botulinum toxin substrate 1	RAC1	10	9	3	By MS/MS	By MS/MS	By MS/MS	By MS/MS	By MS/MS	By MS/MS	By MS/MS	By MS/MS	By MS/MS	By MS/MS	By MS/MS	By MS/MS	54,2	21,45	0	48,03
P15153	P15153	Ras-related C3 botulinum toxin substrate 2	RAC2	7	2	2	By MS/MS	By MS/MS	By MS/MS	By matchin	By MS/MS	By MS/MS	By MS/MS	By MS/MS	By matchin	By MS/MS	By MS/MS	By matchin	44,8	21,43	0	3,92
P60763	P60763	Ras-related C3 botulinum toxin substrate 3	RAC3	8	2	2	By MS/MS	By MS/MS	By matchin	By MS/MS	By MS/MS	By MS/MS	By matchin	By matchin	By matchin	By matchin	By matchin	By matchin	46,4	21,38	0	3,39
P54725	P54725	UV excision repair protein RAD23 homolog A	RAD23A	6	5	5	By MS/MS	By MS/MS	By MS/MS		By MS/MS	By MS/MS	By MS/MS	By MS/MS			By MS/MS	By MS/MS	20,4	39,61	0	37,18
P54727	P54727	UV excision repair protein RAD23 homolog B	RAD23B	12	12	11	By MS/MS	By MS/MS	By MS/MS	By MS/MS	By MS/MS	By MS/MS	By MS/MS	By MS/MS	By MS/MS	By MS/MS	By MS/MS	By MS/MS	46,0	43,17	0	197,69
Q92878	Q92878	DNA repair protein RAD50	RAD50	15	15	15	By MS/MS	By MS/MS	By MS/MS	By MS/MS	By MS/MS	By MS/MS	By MS/MS	By MS/MS	By MS/MS	By MS/MS	By MS/MS	By MS/MS	14,9	153,89	0	68,14
P78406	P78406	mRNA export factor	RAE1	7	7	7		By MS/MS	By MS/MS	By MS/MS	By MS/MS	By MS/MS	By MS/MS	By MS/MS	By MS/MS	By MS/MS			22,8	40,97	0	19,05
P11233	P11233	Ras-related protein Ral-A	RALA	4	4	3	By MS/MS	By MS/MS	By MS/MS	By MS/MS	By MS/MS	By MS/MS		By MS/MS	By MS/MS	By MS/MS	By MS/MS	By MS/MS	20,4	23,57	0	20,54
P62826	P62826	GTP-binding nuclear protein Ran	RAN	16	16	16	By MS/MS	By MS/MS	By MS/MS	By MS/MS	By MS/MS	By MS/MS	By MS/MS	By MS/MS	By MS/MS	By MS/MS	By MS/MS	By MS/MS	50,5	24,42	0	323,31
P43487	P43487	Ran-specific GTPase-activating protein	RANBP1	11	11	11	By MS/MS	By MS/MS	By MS/MS	By MS/MS	By MS/MS	By MS/MS	By MS/MS	By MS/MS	By MS/MS	By MS/MS	By MS/MS	By MS/MS	66,7	23,31	0	214,07
P49792;O14715;Q99666;P0DJD0;P0DJD1	P49792	E3 SUMO-protein ligase RanBP2	RANBP2	24	24	16	By MS/MS	By MS/MS	By MS/MS	By MS/MS	By MS/MS	By MS/MS		By MS/MS		By MS/MS			13,2	358,20	0	166,95
P46060	P46060	Ran GTPase-activating protein 1	RANGAP1	20	20	20	By MS/MS	By MS/MS	By MS/MS	By MS/MS	By MS/MS	By MS/MS	By MS/MS	By MS/MS	By MS/MS	By MS/MS	By MS/MS	By MS/MS	46,2	63,54	0	309,67
P62834	P62834	Ras-related protein Rap-1A	RAP1A	12	2	2	By MS/MS	By matchin	By matchin	By MS/MS	By MS/MS	By MS/MS	By MS/MS	By matchin	By MS/MS	By matchin	By matchin	By matchin	66,3	20,99	0	5,10
P61224;A6NIZ1	P61224;A6NIZ1	Ras-related protein Rap-1b;Ras-related protein Rap-1b-like protein	RAP1B	14	14	4	By MS/MS	By MS/MS	By MS/MS	By MS/MS	By MS/MS	By MS/MS	By MS/MS	By MS/MS	By MS/MS	By MS/MS	By MS/MS	By MS/MS	80,4	20,83	0	263,05
P52306	P52306	Rap1 GTPase-GDP dissociation stimulator 1	RAP1GDS1	8	8	8	By MS/MS	By MS/MS		By MS/MS	By MS/MS	By MS/MS						By MS/MS	22,1	66,32	0	70,40
P61225	P61225	Ras-related protein Rap-2b	RAP2B	6	6	4	By MS/MS	By MS/MS	By MS/MS	By MS/MS	By MS/MS	By MS/MS	By MS/MS	By MS/MS	By MS/MS				51,9	20,50	0	15,25
Q9Y3L5;P10114	Q9Y3L5	Ras-related protein Rap-2c	RAP2C	5	3	3		By MS/MS	By matchin	By MS/MS		By MS/MS	By matchin	By matching					32,8	20,75	0	3,82
P54136	P54136	Arginine--tRNA ligase, cytoplasmic	RARS	25	25	25	By MS/MS	By MS/MS	By MS/MS	By MS/MS	By MS/MS	By MS/MS	By MS/MS	By MS/MS		By MS/MS	By MS/MS	By MS/MS	45,5	75,38	0	226,76
Q8IY67	Q8IY67	Ribonucleoprotein PTB-binding 1	RAVER1	4	4	4	By MS/MS		By MS/MS	By MS/MS	By MS/MS	By MS/MS					By MS/MS	By MS/MS	10,6	63,88	0	20,29
Q09028	Q09028	Histone-binding protein RBBP4	RBBP4	13	13	8	By MS/MS	By MS/MS	By MS/MS	By MS/MS	By MS/MS	By MS/MS	By MS/MS	By MS/MS			By MS/MS	By MS/MS	42,4	47,66	0	182,15
Q16576	Q16576	Histone-binding protein RBBP7	RBBP7	13	8	8	By MS/MS	By MS/MS	By MS/MS	By MS/MS	By MS/MS	By MS/MS	By matchin	By matching			By MS/MS	By MS/MS	41,2	47,82	0	22,92
Q96PK6	Q96PK6	RNA-binding protein 14	RBM14	5	5	5	By MS/MS	By MS/MS	By MS/MS	By MS/MS	By MS/MS	By MS/MS	By MS/MS	By MS/MS	By MS/MS	By MS/MS	By MS/MS	By MS/MS	10,0	69,49	0	132,03

P49756	P49756	RNA-binding protein 25	RBM25	6	6	6	By MS/MS	By MS/MS	By MS/MS	By MS/MS	By MS/MS	By MS/MS	By MS/MS	By MS/MS	By MS/MS	By MS/MS	By MS/MS	By MS/MS	9,6	100,18	0	9,96
Q9P2N5;Q5T8P6	Q9P2N5	RNA-binding protein 27	RBM27	7	7	7	By MS/MS	By MS/MS	By MS/MS	By MS/MS	By MS/MS	By MS/MS			By MS/MS			By MS/MS	10,8	118,72	0	37,46
Q9NW13	Q9NW13	RNA-binding protein 28	RBM28	5	5	5	By MS/MS	By MS/MS	By MS/MS	By MS/MS	By MS/MS	By MS/MS	By MS/MS	By MS/MS	By MS/MS	By MS/MS			7,9	85,74	0	23,71
Q14498	Q14498	RNA-binding protein 39	RBM39	7	7	7	By MS/MS	By MS/MS	By MS/MS	By MS/MS	By MS/MS	By MS/MS	By MS/MS	By MS/MS	By MS/MS	By MS/MS	By MS/MS	By MS/MS	18,7	59,38	0	184,86
Q9BWF3;Q9BQ04	Q9BWF3	RNA-binding protein 4	RBM4	5	5	5	By MS/MS	By MS/MS	By MS/MS	By MS/MS	By MS/MS	By MS/MS	By MS/MS	By MS/MS	By MS/MS	By MS/MS			26,4	40,31	0	248,83
Q9Y5S9	Q9Y5S9	RNA-binding protein 8A	RBM8A	6	6	6	By MS/MS	By MS/MS	By MS/MS	By MS/MS	By MS/MS	By MS/MS	By MS/MS	By MS/MS	By MS/MS	By MS/MS	By MS/MS	By MS/MS	41,4	19,89	0	88,23
P38159;Q96E39; O75526	P38159;Q96E39	RNA-binding motif protein, X chromosome;RNA-binding motif protein, X chromosome, N-terminally processed;RNA binding motif protein, X-linked-like-1	RBMX;RBMXL1	7	7	6	By MS/MS	By MS/MS	By MS/MS	By MS/MS	By MS/MS	By MS/MS	By MS/MS	By MS/MS	By MS/MS	By MS/MS			24,0	42,33	0	36,08
P18754	P18754	Regulator of chromosome condensation	RCC1	5	5	5	By MS/MS	By MS/MS	By MS/MS	By MS/MS	By MS/MS	By MS/MS	By MS/MS	By MS/MS	By MS/MS	By MS/MS	By MS/MS	By MS/MS	18,5	44,97	0	251,74
Q9P258	Q9P258	Protein RCC2	RCC2	17	17	17	By MS/MS	By MS/MS	By MS/MS	By MS/MS	By MS/MS	By MS/MS	By MS/MS	By MS/MS			By MS/MS	By MS/MS	41,4	56,08	0	174,33
Q8TC12	Q8TC12	Retinol dehydrogenase 11	RDH11	6	6	6	By MS/MS	By MS/MS	By MS/MS	By MS/MS	By MS/MS	By MS/MS			By MS/MS	By MS/MS	By MS/MS	By MS/MS	24,8	35,39	0	45,89
Q8NBN7	Q8NBN7	Retinol dehydrogenase 13	RDH13	8	8	8	By MS/MS	By MS/MS	By MS/MS	By MS/MS	By MS/MS	By MS/MS	By MS/MS	By MS/MS	By MS/MS	By MS/MS	By MS/MS	By MS/MS	35,6	35,93	0	166,74
P35241	P35241	Radixin	RDX	19	9	9	By MS/MS	By MS/MS	By MS/MS	By MS/MS	By MS/MS	By MS/MS	By matchin	By MS/MS	By MS/MS	By MS/MS	By MS/MS	By MS/MS	41,5	68,56	0	33,00
Q04206	Q04206	Transcription factor p65	RELA	5	5	5	By MS/MS	By MS/MS	By MS/MS	By MS/MS	By MS/MS	By MS/MS			By MS/MS	By MS/MS			13,1	60,22	0	5,42
Q9Y3B8	Q9Y3B8	Oligoribonuclease, mitochondrial	REXO2	7	7	7	By MS/MS	By MS/MS	By MS/MS	By MS/MS	By MS/MS	By MS/MS	By MS/MS	By MS/MS	By MS/MS	By MS/MS	By MS/MS	By MS/MS	38,0	26,83	0	60,84
P35250	P35250	Replication factor C subunit 2	RFC2	4	4	4	By MS/MS	By MS/MS	By MS/MS	By MS/MS	By MS/MS	By MS/MS	By MS/MS	By MS/MS	By MS/MS	By MS/MS			18,4	39,16	0	46,89
P40938	P40938	Replication factor C subunit 3	RFC3	4	4	4	By MS/MS	By MS/MS	By MS/MS	By MS/MS	By MS/MS	By MS/MS	By MS/MS	By MS/MS	By MS/MS	By MS/MS			18,5	40,56	0	84,96
P35249	P35249	Replication factor C subunit 4	RFC4	5	5	5	By MS/MS	By MS/MS	By MS/MS	By MS/MS	By MS/MS	By MS/MS	By MS/MS	By MS/MS	By MS/MS	By MS/MS	By MS/MS	By MS/MS	23,1	39,68	0	20,36
P40937	P40937	Replication factor C subunit 5	RFC5	13	13	13	By MS/MS	By MS/MS	By MS/MS	By MS/MS	By MS/MS	By MS/MS	By MS/MS	By MS/MS	By MS/MS	By MS/MS	By MS/MS	By MS/MS	54,7	38,50	0	94,96
Q15382	Q15382	GTP-binding protein Rheb	RHEB	4	4	4	By MS/MS	By MS/MS	By MS/MS	By MS/MS	By MS/MS	By MS/MS			By MS/MS	By MS/MS			20,7	20,50	0	16,84
P61586	P61586	Transforming protein RhoA	RHOA	11	11	4	By MS/MS	By MS/MS	By MS/MS	By MS/MS	By MS/MS	By MS/MS	By MS/MS	By MS/MS	By MS/MS	By MS/MS	By MS/MS	By MS/MS	58,5	21,77	0	323,31
P08134;P62745	P08134	Rho-related GTP-binding protein RhoC	RHOC	9	2	2	By MS/MS	By MS/MS	By MS/MS	By MS/MS	By MS/MS	By MS/MS	By MS/MS	By MS/MS	By MS/MS	By MS/MS	By MS/MS	By MS/MS	50,3	22,01	0	9,20
P84095	P84095	Rho-related GTP-binding protein RhoG	RHOG	12	12	11	By MS/MS	By MS/MS	By MS/MS	By MS/MS	By MS/MS	By MS/MS	By MS/MS	By MS/MS	By MS/MS	By MS/MS	By MS/MS	By MS/MS	59,2	21,31	0	254,71
Q96DB5	Q96DB5	Regulator of microtubule dynamics protein 1	RMDN1	4	4	4	By MS/MS	By MS/MS		By MS/MS			By MS/MS						12,1	35,81	0	4,51
Q8TDP1	Q8TDP1	Ribonuclease H2 subunit C	RNASEH2C	4	4	4	By MS/MS	By MS/MS		By MS/MS	By MS/MS	By MS/MS					By MS/MS		36,6	17,84	0	6,58
Q5VTR2	Q5VTR2	E3 ubiquitin-protein ligase BRE1A	RNF20	7	7	6	By MS/MS	By MS/MS	By MS/MS	By MS/MS	By MS/MS	By MS/MS					By MS/MS		10,5	113,66	0	12,79
O75150	O75150	E3 ubiquitin-protein ligase BRE1B	RNF40	7	6	6		By MS/MS	By matchin	By MS/MS	By MS/MS	By MS/MS					By matching		11,9	113,65	0	4,15
P13489	P13489	Ribonuclease inhibitor	RNH1	18	18	18	By MS/MS	By MS/MS	By MS/MS	By MS/MS	By MS/MS	By MS/MS	By MS/MS	By MS/MS	By MS/MS	By MS/MS	By MS/MS	By MS/MS	61,8	49,97	0	205,76
Q9H4A4	Q9H4A4	Aminopeptidase B	RNPEP	23	23	23	By MS/MS	By MS/MS	By MS/MS	By MS/MS	By MS/MS	By MS/MS	By MS/MS	By MS/MS	By MS/MS	By MS/MS	By MS/MS	By MS/MS	50,3	72,60	0	111,46
P27694	P27694	Replication protein A 70 kDa DNA-binding subunit;Replication protein A 70 kDa DNA-binding subunit, N-terminally processed	RPA1	10	10	10	By MS/MS	By MS/MS	By MS/MS	By MS/MS	By MS/MS	By MS/MS	By MS/MS	By MS/MS	By MS/MS	By MS/MS	By MS/MS	By MS/MS	29,7	68,14	0	158,03
P15927	P15927	Replication protein A 32 kDa subunit	RPA2	9	9	9	By MS/MS	By MS/MS	By MS/MS	By MS/MS	By MS/MS	By MS/MS			By MS/MS	By MS/MS		By MS/MS	69,6	29,25	0	223,88
P35244	P35244	Replication protein A 14 kDa subunit	RPA3	6	6	6	By MS/MS	By MS/MS	By MS/MS	By MS/MS	By MS/MS	By MS/MS	By MS/MS	By MS/MS	By MS/MS	By MS/MS	By MS/MS	By MS/MS	80,2	13,57	0	41,31
P49247	P49247	Ribose-5-phosphate isomerase	RPIA	8	8	8	By MS/MS	By MS/MS	By MS/MS	By MS/MS	By MS/MS	By MS/MS	By MS/MS	By MS/MS	By MS/MS	By MS/MS	By MS/MS	By MS/MS	34,4	33,27	0	27,15
P27635;Q96L21	P27635	60S ribosomal protein L10	RPL10	13	13	13	By MS/MS	By MS/MS	By MS/MS	By MS/MS	By MS/MS	By MS/MS	By MS/MS	By MS/MS	By MS/MS	By MS/MS	By MS/MS	By MS/MS	46,7	24,60	0	323,31
P62906	P62906	60S ribosomal protein L10a	RPL10A	12	12	12	By MS/MS	By MS/MS	By MS/MS	By MS/MS	By MS/MS	By MS/MS	By MS/MS	By MS/MS	By MS/MS	By MS/MS	By MS/MS	By MS/MS	42,4	24,83	0	98,98
P62913	P62913	60S ribosomal protein L11	RPL11	14	14	14	By MS/MS	By MS/MS	By MS/MS	By MS/MS	By MS/MS	By MS/MS	By MS/MS	By MS/MS	By MS/MS	By MS/MS	By MS/MS	By MS/MS	72,5	20,25	0	314,21
P30050	P30050	60S ribosomal protein L12	RPL12	10	10	10	By MS/MS	By MS/MS	By MS/MS	By MS/MS	By MS/MS	By MS/MS	By MS/MS	By MS/MS	By MS/MS	By MS/MS	By MS/MS	By MS/MS	70,9	17,82	0	323,31
P26373	P26373	60S ribosomal protein L13	RPL13	12	12	12	By MS/MS	By MS/MS	By MS/MS	By MS/MS	By MS/MS	By MS/MS	By MS/MS	By MS/MS	By MS/MS	By MS/MS	By MS/MS	By MS/MS	47,9	24,26	0	96,44
P40429;Q6NVV1	P40429	60S ribosomal protein L13a	RPL13A	14	14	14	By MS/MS	By MS/MS	By MS/MS	By MS/MS	By MS/MS	By MS/MS	By MS/MS	By MS/MS	By MS/MS	By MS/MS	By MS/MS	By MS/MS	49,8	23,58	0	56,81
P50914	P50914	60S ribosomal protein L14	RPL14	7	7	7	By MS/MS	By MS/MS	By MS/MS	By MS/MS	By MS/MS	By MS/MS	By MS/MS	By MS/MS	By MS/MS	By MS/MS	By MS/MS	By MS/MS	28,4	23,43	0	100,17
P61313	P61313	60S ribosomal protein L15	RPL15	9	9	9	By MS/MS	By MS/MS	By MS/MS	By MS/MS	By MS/MS	By MS/MS	By MS/MS	By MS/MS	By MS/MS	By MS/MS	By MS/MS	By MS/MS	39,7	24,15	0	139,27
P18621	P18621	60S ribosomal protein L17	RPL17	13	13	13	By MS/MS	By MS/MS	By MS/MS	By MS/MS	By MS/MS	By MS/MS	By MS/MS	By MS/MS	By MS/MS	By MS/MS	By MS/MS	By MS/MS	50,5	21,40	0	60,60
Q07020	Q07020	60S ribosomal protein L18	RPL18	9	9	9	By MS/MS	By MS/MS	By MS/MS	By MS/MS	By MS/MS	By MS/MS	By MS/MS	By MS/MS	By MS/MS	By MS/MS	By MS/MS	By MS/MS	35,1	21,63	0	323,31
Q02543	Q02543	60S ribosomal protein L18a	RPL18A	19	19	19	By MS/MS	By MS/MS	By MS/MS	By MS/MS	By MS/MS	By MS/MS	By MS/MS	By MS/MS	By MS/MS	By MS/MS	By MS/MS	By MS/MS	61,9	20,76	0	228,76
P84098	P84098	60S ribosomal protein L19	RPL19	13	13	13	By MS/MS	By MS/MS	By MS/MS	By MS/MS	By MS/MS	By MS/MS	By MS/MS	By MS/MS	By MS/MS	By MS/MS	By MS/MS	By MS/MS	42,9	23,47	0	323,31
P46778	P46778	60S ribosomal protein L21	RPL21	11	11	11	By MS/MS	By MS/MS	By MS/MS	By MS/MS	By MS/MS	By MS/MS	By MS/MS	By MS/MS	By MS/MS	By MS/MS	By MS/MS	By MS/MS	48,1	18,57	0	127,35
P35268	P35268	60S ribosomal protein L22	RPL22	7	7	6	By MS/MS	By MS/MS	By MS/MS	By MS/MS	By MS/MS	By MS/MS	By MS/MS	By MS/MS	By MS/MS	By MS/MS	By MS/MS	By MS/MS	39,1	14,79	0	323,31
P62829	P62829	60S ribosomal protein L23	RPL23	10	10	10	By MS/MS	By MS/MS	By MS/MS	By MS/MS	By MS/MS	By MS/MS	By MS/MS	By MS/MS	By MS/MS	By MS/MS	By MS/MS	By MS/MS	61,4	14,87	0	157,81
P62750	P62750	60S ribosomal protein L23a	RPL23A	14	14	14	By MS/MS	By MS/MS	By MS/MS	By MS/MS	By MS/MS	By MS/MS	By MS/MS	By MS/MS	By MS/MS	By MS/MS	By MS/MS	By MS/MS	47,4	17,70	0	38,14
P83731	P83731	60S ribosomal protein L24	RPL24	10	10	10	By MS/MS	By MS/MS	By MS/MS	By MS/MS	By MS/MS	By MS/MS	By MS/MS	By MS/MS	By MS/MS	By MS/MS	By MS/MS	By MS/MS	45,2	17,78	0	138,87
P61254;Q9UNX3	P61254;Q9UNX3	60S ribosomal protein L26;60S ribosomal protein L26-like 1	RPL26;RPL26L1	11	11	11	By MS/MS	By MS/MS	By MS/MS	By MS/MS	By MS/MS	By MS/MS	By MS/MS	By MS/MS	By MS/MS	By MS/MS	By MS/MS	By MS/MS	44,8	17,26	0	24,09
P61353	P61353	60S ribosomal protein L27	RPL27	11	11	11	By MS/MS	By MS/MS	By MS/MS	By MS/MS	By MS/MS	By MS/MS	By MS/MS	By MS/MS	By MS/MS	By MS/MS	By MS/MS	By MS/MS	54,4	15,80	0	25,81
P46776	P46776	60S ribosomal protein L27a	RPL27A	9	9	9	By MS/MS	By MS/MS	By MS/MS	By MS/MS	By MS/MS	By MS/MS	By MS/MS	By MS/MS	By MS/MS	By MS/MS	By MS/MS	By MS/MS	45,3	16,56	0	220,36
P46779	P46779	60S ribosomal protein L28	RPL28	8	8	8	By MS/MS	By MS/MS	By MS/MS	By MS/MS	By MS/MS	By MS/MS	By MS/MS	By MS/MS	By MS/MS	By MS/MS	By MS/MS	By MS/MS	40,9	15,75	0	46,81
P47914	P47914	60S ribosomal protein L29	RPL29	4	4	4	By MS/MS	By MS/MS	By MS/MS	By MS/MS	By MS/MS	By MS/MS	By MS/MS	By MS/MS	By MS/MS	By MS/MS	By MS/MS	By MS/MS	20,8	17,75	0	20,18
P39023;Q92901	P39023	60S ribosomal protein L3	RPL3	13	13	13	By MS/MS	By MS/MS	By MS/MS	By MS/MS	By MS/MS	By MS/MS	By MS/MS	By MS/MS	By MS/MS	By MS/MS	By MS/MS	By MS/MS	36,0	46,11	0	120,79
P62888	P62888	60S ribosomal protein L30	RPL30	9	9	9	By MS/MS	By MS/MS	By MS/MS	By MS/MS	By MS/MS	By MS/MS	By MS/MS	By MS/MS	By MS/MS	By MS/MS	By MS/MS	By MS/MS	68,7	12,78	0	284,01
P62899	P62899	60S ribosomal protein L31	RPL31	7	7	7	By MS/MS	By MS/MS	By MS/MS	By MS/MS	By MS/MS	By MS/MS	By MS/MS	By MS/MS	By MS/MS	By MS/MS	By MS/MS	By MS/MS	46,4	14,46	0	244,84
P62910	P62910	60S ribosomal protein L32	RPL32	13	13	13	By MS/MS	By MS/MS	By MS/MS	By MS/MS	By MS/MS	By MS/MS	By MS/MS	By MS/MS	By MS/MS	By MS/MS	By MS/MS	By MS/MS	54,8	15,86	0	259,68
P49207	P49207	60S ribosomal protein L34	RPL34	6	6	6	By MS/MS	By MS/MS	By MS/MS	By MS/MS	By MS/MS	By MS/MS	By MS/MS	By MS/MS	By MS/MS	By MS/MS	By MS/MS	By MS/MS	32,5	13,29	0	9,44
P42766	P42766	60S ribosomal protein L35	RPL35	6	6	6	By MS/MS	By MS/MS	By MS/MS	By MS/MS	By MS/MS	By MS/MS	By MS/MS	By MS/MS	By MS/MS	By MS/MS	By MS/MS	By MS/MS	35,8	14,55	0	323,31
P18077	P18077	60S ribosomal protein L35a	RPL35A	12	12	12	By MS/MS	By MS/MS	By MS/MS	By MS/MS	By MS/MS	By MS/MS	By MS/MS	By MS/MS	By MS/MS	By MS/MS	By MS/MS	By MS/MS	49,1	12,54	0	78,80
Q9Y3U8	Q9Y3U8	60S ribosomal protein L36	RPL36	6	6	6	By MS/MS	By MS/MS	By MS/MS	By MS/MS	By MS/MS	By MS/MS	By MS/MS	By MS/MS	By MS/MS	By MS/MS	By MS/MS	By MS/MS	33,3	12,25	0	35,57
P83881;Q969Q0	P83881;Q969Q0	60S ribosomal protein L36a;60S ribosomal protein L36a-like	RPL36A;RPL36AL	6	6	6		By MS/MS	By MS/MS			By MS/MS	By MS/MS	By MS/MS	By MS/MS	By MS/MS	By MS/MS	By MS/MS	48,1	12,44	0	6,74
P61513;A6NKH3	P61513	60S ribosomal protein L37a	RPL37A	4	4	4	By MS/MS	By MS/MS	By MS/MS	By MS/MS	By MS/MS	By MS/MS	By MS/MS	By MS/MS	By MS/MS	By MS/MS	By MS/MS	By MS/MS	51,1	10,28	0	233,39
P63173	P63173	60S ribosomal protein L38	RPL38	8	8	8	By MS/MS	By MS/MS	By MS/MS	By MS/MS	By MS/MS	By MS/MS	By MS/MS	By MS/MS	By MS/MS	By MS/MS	By MS/MS	By MS/MS	57,1	8,22	0	35,41
P36578	P36578	60S ribosomal protein L4	RPL4	23	23	23	By MS/MS	By MS/MS	By MS/MS	By MS/MS	By MS/MS	By MS/MS	By MS/MS	By MS/MS	By MS/MS	By MS/MS	By MS/MS	By MS/MS	46,4	47,70	0	199,97
P46777	P46777	60S ribosomal protein L5	RPL5	14	14	14	By MS/MS	By MS/MS	By MS/MS	By MS/MS	By MS/MS	By MS/MS	By MS/MS	By MS/MS	By MS/MS	By MS/MS	By MS/MS	By MS/MS	40,4	34,36	0	78,31
Q02878	Q02878	60S ribosomal protein L6	RPL6	18	18	18	By MS/MS	By MS/MS	By MS/MS	By MS/MS	By MS/MS	By MS/MS	By MS/MS	By MS/MS	By MS/MS	By MS/MS	By MS/MS	By MS/MS	52,8	32,73	0	193,06
P18124	P18124	60S ribosomal protein L7	RPL7	18	18	18	By MS/MS	By MS/MS	By MS/MS	By MS/MS	By MS/MS	By MS/MS	By MS/MS	By MS/MS	By MS/MS	By MS/MS	By MS/MS	By MS/MS	48,4	29,23	0	211,87
P62424	P62424	60S ribosomal protein L7a	RPL7A	17	17	17	By MS/MS	By MS/MS	By MS/MS	By MS/MS	By MS/MS	By MS/MS	By MS/MS	By MS/MS	By MS/MS	By MS/MS	By MS/MS	By MS/MS	51,1	30,00	0	323,31
Q6DKI1	Q6DKI1	60S ribosomal protein L7-like 1	RPL7L1	4	4	4	By MS/MS	By MS/MS	By MS/MS	By MS/MS	By MS/MS	By MS/MS				By MS/MS			17,5	28,66	0	4,53

P62917	P62917	60S ribosomal protein L8	RPL8	11	11	11	By MS/MS	By MS/MS	By MS/MS	By MS/MS	By MS/MS	By MS/MS	By MS/MS	By MS/MS	By MS/MS	By MS/MS	By MS/MS	By MS/MS	42,4	28,02	0	155,46
P32969	P32969	60S ribosomal protein L9	RPL9	15	15	15	By MS/MS	By MS/MS	By MS/MS	By MS/MS	By MS/MS	By MS/MS	By MS/MS	By MS/MS	By MS/MS	By MS/MS	By MS/MS	By MS/MS	72,4	21,86	0	323,31
P05388;Q8NHW5	P05388;Q8NHW5	60S acidic ribosomal protein P0;60S acidic ribosomal protein P0-like	RPLP0;RPLP0P6	16	16	16	By MS/MS	By MS/MS	By MS/MS	By MS/MS	By MS/MS	By MS/MS	By MS/MS	By MS/MS	By MS/MS	By MS/MS	By MS/MS	By MS/MS	64,0	34,27	0	323,31
P05386	P05386	60S acidic ribosomal protein P1	RPLP1	6	5	5	By MS/MS	By MS/MS	By MS/MS	By MS/MS	By MS/MS	By MS/MS	By MS/MS	By MS/MS	By MS/MS	By MS/MS	By MS/MS	By MS/MS	95,6	11,51	0	115,18
P05387	P05387	60S acidic ribosomal protein P2	RPLP2	11	11	10	By MS/MS	By MS/MS	By MS/MS	By MS/MS	By MS/MS	By MS/MS	By MS/MS	By MS/MS	By MS/MS	By MS/MS	By MS/MS	By MS/MS	97,4	11,67	0	323,31
P04843	P04843	Dolichyl-diphosphooligosaccharide--protein glycosyltransferase subunit 1	RPN1	26	26	26	By MS/MS	By MS/MS	By MS/MS	By MS/MS	By MS/MS	By MS/MS	By MS/MS	By MS/MS	By MS/MS	By MS/MS	By MS/MS	By MS/MS	59,5	68,57	0	162,86
P04844	P04844	Dolichyl-diphosphooligosaccharide--protein glycosyltransferase subunit 2	RPN2	22	22	22	By MS/MS	By MS/MS	By MS/MS	By MS/MS	By MS/MS	By MS/MS	By MS/MS	By MS/MS	By MS/MS	By MS/MS	By MS/MS	By MS/MS	54,0	69,28	0	323,31
P78346	P78346	Ribonuclease P protein subunit p30	RPP30	7	7	7	By MS/MS	By MS/MS	By MS/MS	By MS/MS	By MS/MS	By MS/MS			By MS/MS	By MS/MS			36,9	29,32	0	30,56
Q9NQG5;Q96P16	Q9NQG5	Regulation of nuclear pre-mRNA domain-containing protein 1B	RPRD1B	7	7	7	By MS/MS	By MS/MS	By MS/MS	By MS/MS	By MS/MS	By MS/MS	By MS/MS	By MS/MS	By MS/MS	By MS/MS	By MS/MS	By MS/MS	32,8	36,90	0	148,70
P46783;Q9NQ39	P46783	40S ribosomal protein S10	RPS10	13	13	13	By MS/MS	By MS/MS	By MS/MS	By MS/MS	By MS/MS	By MS/MS	By MS/MS	By MS/MS	By MS/MS	By MS/MS	By MS/MS	By MS/MS	65,5	18,90	0	323,31
P62280	P62280	40S ribosomal protein S11	RPS11	17	17	17	By MS/MS	By MS/MS	By MS/MS	By MS/MS	By MS/MS	By MS/MS	By MS/MS	By MS/MS	By MS/MS	By MS/MS	By MS/MS	By MS/MS	65,8	18,43	0	180,96
P25398	P25398	40S ribosomal protein S12	RPS12	13	13	13	By MS/MS	By MS/MS	By MS/MS	By MS/MS	By MS/MS	By MS/MS	By MS/MS	By MS/MS	By MS/MS	By MS/MS	By MS/MS	By MS/MS	81,8	14,52	0	323,31
P62277	P62277	40S ribosomal protein S13	RPS13	14	14	14	By MS/MS	By MS/MS	By MS/MS	By MS/MS	By MS/MS	By MS/MS	By MS/MS	By MS/MS	By MS/MS	By MS/MS	By MS/MS	By MS/MS	62,9	17,22	0	83,77
P62263	P62263	40S ribosomal protein S14	RPS14	10	10	10	By MS/MS	By MS/MS	By MS/MS	By MS/MS	By MS/MS	By MS/MS	By MS/MS	By MS/MS	By MS/MS	By MS/MS	By MS/MS	By MS/MS	41,7	16,27	0	313,91
P62841	P62841	40S ribosomal protein S15	RPS15	6	6	6	By MS/MS	By MS/MS	By MS/MS		By MS/MS	By MS/MS	By MS/MS	By MS/MS	By MS/MS	By MS/MS	By MS/MS	By MS/MS	33,8	17,04	0	165,63
P62244	P62244	40S ribosomal protein S15a	RPS15A	13	13	13	By MS/MS	By MS/MS	By MS/MS	By MS/MS	By MS/MS	By MS/MS	By MS/MS	By MS/MS	By MS/MS	By MS/MS	By MS/MS	By MS/MS	76,2	14,84	0	125,38
P62249	P62249	40S ribosomal protein S16	RPS16	20	20	20	By MS/MS	By MS/MS	By MS/MS	By MS/MS	By MS/MS	By MS/MS	By MS/MS	By MS/MS	By MS/MS	By MS/MS	By MS/MS	By MS/MS	79,5	16,45	0	60,44
P08708	P08708	40S ribosomal protein S17	RPS17	11	11	11	By MS/MS	By MS/MS	By MS/MS	By MS/MS	By MS/MS	By MS/MS	By MS/MS	By MS/MS	By MS/MS	By MS/MS	By MS/MS	By MS/MS	66,7	15,55	0	270,67
P62269	P62269	40S ribosomal protein S18	RPS18	20	20	20	By MS/MS	By MS/MS	By MS/MS	By MS/MS	By MS/MS	By MS/MS	By MS/MS	By MS/MS	By MS/MS	By MS/MS	By MS/MS	By MS/MS	68,4	17,72	0	323,31
P39019	P39019	40S ribosomal protein S19	RPS19	15	15	15	By MS/MS	By MS/MS	By MS/MS	By MS/MS	By MS/MS	By MS/MS	By MS/MS	By MS/MS	By MS/MS	By MS/MS	By MS/MS	By MS/MS	63,4	16,06	0	99,63
P15880	P15880	40S ribosomal protein S2	RPS2	14	14	14	By MS/MS	By MS/MS	By MS/MS	By MS/MS	By MS/MS	By MS/MS	By MS/MS	By MS/MS	By MS/MS	By MS/MS	By MS/MS	By MS/MS	44,0	31,32	0	276,96
P60866	P60866	40S ribosomal protein S20	RPS20	7	7	7	By MS/MS	By MS/MS	By MS/MS	By MS/MS	By MS/MS	By MS/MS	By MS/MS	By MS/MS	By MS/MS	By MS/MS	By MS/MS	By MS/MS	49,6	13,37	0	148,19
P63220	P63220	40S ribosomal protein S21	RPS21	9	9	9	By MS/MS	By MS/MS	By MS/MS	By MS/MS	By MS/MS	By MS/MS	By MS/MS	By MS/MS	By MS/MS	By MS/MS	By MS/MS	By MS/MS	71,1	9,11	0	81,36
P62266	P62266	40S ribosomal protein S23	RPS23	7	7	7	By MS/MS	By MS/MS		By MS/MS	By MS/MS	By MS/MS	By MS/MS	By MS/MS	By MS/MS	By MS/MS	By MS/MS	By MS/MS	49,7	15,81	0	158,04
P62847	P62847	40S ribosomal protein S24	RPS24	6	6	6	By MS/MS	By MS/MS	By MS/MS	By MS/MS	By MS/MS	By MS/MS	By MS/MS	By MS/MS	By MS/MS	By MS/MS	By MS/MS	By MS/MS	36,1	15,42	0	56,42
P62851	P62851	40S ribosomal protein S25	RPS25	9	9	9	By MS/MS	By MS/MS	By MS/MS	By MS/MS	By MS/MS	By MS/MS	By MS/MS	By MS/MS	By MS/MS	By MS/MS	By MS/MS	By MS/MS	41,6	13,74	0	158,94
P62854;Q5JNZ5	P62854;Q5JNZ5	40S ribosomal protein S26;Putative 40S ribosomal protein S26-like 1	RPS26;RPS26P11	4	4	4	By MS/MS	By MS/MS	By MS/MS	By MS/MS	By MS/MS	By MS/MS	By MS/MS	By MS/MS	By MS/MS	By MS/MS	By MS/MS	By MS/MS	37,4	13,02	0	52,81
P42677	P42677	40S ribosomal protein S27	RPS27	7	7	4	By MS/MS	By MS/MS	By MS/MS	By MS/MS	By MS/MS	By MS/MS	By MS/MS	By MS/MS	By MS/MS	By MS/MS	By MS/MS	By MS/MS	46,4	9,46	0	54,27
P62979;P62987;P0CG47;P0CG48	P62979;P62987;P0CG47;P0CG48	Ubiquitin-40S ribosomal protein S27a;Ubiquitin;40S ribosomal protein S27a;Ubiquitin-60S ribosomal protein L40;Ubiquitin;60S ribosomal protein L40;Polyubiquitin-B;Ubiquitin;Polyubiquitin-C;Ubiquitin	RPS27A;UBA52;UBB;UBC	12	12	12	By MS/MS	By MS/MS	By MS/MS	By MS/MS	By MS/MS	By MS/MS	By MS/MS	By MS/MS	By MS/MS	By MS/MS	By MS/MS	By MS/MS	62,8	17,97	0	323,31
P62857	P62857	40S ribosomal protein S28	RPS28	8	8	8	By MS/MS	By MS/MS	By MS/MS	By MS/MS	By MS/MS	By MS/MS	By MS/MS	By MS/MS	By MS/MS	By MS/MS	By MS/MS	By MS/MS	79,7	7,84	0	161,58
P23396	P23396	40S ribosomal protein S3	RPS3	21	21	21	By MS/MS	By MS/MS	By MS/MS	By MS/MS	By MS/MS	By MS/MS	By MS/MS	By MS/MS	By MS/MS	By MS/MS	By MS/MS	By MS/MS	69,1	26,69	0	323,31
P61247	P61247	40S ribosomal protein S3a	RPS3A	19	19	19	By MS/MS	By MS/MS	By MS/MS	By MS/MS	By MS/MS	By MS/MS	By MS/MS	By MS/MS	By MS/MS	By MS/MS	By MS/MS	By MS/MS	61,0	29,95	0	323,31
P62701;Q8TD47;P22090	P62701	40S ribosomal protein S4, X isoform	RPS4X	22	22	22	By MS/MS	By MS/MS	By MS/MS	By MS/MS	By MS/MS	By MS/MS	By MS/MS	By MS/MS	By MS/MS	By MS/MS	By MS/MS	By MS/MS	60,8	29,60	0	323,31
P46782	P46782	40S ribosomal protein S5;40S ribosomal protein S5, N-terminally processed	RPS5	10	10	10	By MS/MS	By MS/MS	By MS/MS	By MS/MS	By MS/MS	By MS/MS	By MS/MS	By MS/MS	By MS/MS	By MS/MS	By MS/MS	By MS/MS	48,0	22,88	0	323,31
P62753	P62753	40S ribosomal protein S6	RPS6	11	11	11	By MS/MS	By MS/MS	By MS/MS	By MS/MS	By MS/MS	By MS/MS	By MS/MS	By MS/MS	By MS/MS	By MS/MS	By MS/MS	By MS/MS	38,2	28,68	0	323,31
P62081	P62081	40S ribosomal protein S7	RPS7	18	18	18	By MS/MS	By MS/MS	By MS/MS	By MS/MS	By MS/MS	By MS/MS	By MS/MS	By MS/MS	By MS/MS	By MS/MS	By MS/MS	By MS/MS	68,6	22,13	0	49,96
P62241	P62241	40S ribosomal protein S8	RPS8	15	15	15	By MS/MS	By MS/MS	By MS/MS	By MS/MS	By MS/MS	By MS/MS	By MS/MS	By MS/MS	By MS/MS	By MS/MS	By MS/MS	By MS/MS	60,1	24,21	0	323,31
P46781	P46781	40S ribosomal protein S9	RPS9	22	22	22	By MS/MS	By MS/MS	By MS/MS	By MS/MS	By MS/MS	By MS/MS	By MS/MS	By MS/MS	By MS/MS	By MS/MS	By MS/MS	By MS/MS	51,5	22,59	0	100,83
P08865	P08865	40S ribosomal protein SA	RPSA	19	19	19	By MS/MS	By MS/MS	By MS/MS	By MS/MS	By MS/MS	By MS/MS	By MS/MS	By MS/MS	By MS/MS	By MS/MS	By MS/MS	By MS/MS	64,4	32,85	0	323,31
Q92600	Q92600	Cell differentiation protein RCD1 homolog	RQCD1	7	7	7	By MS/MS	By MS/MS	By MS/MS	By MS/MS	By MS/MS	By MS/MS	By MS/MS	By MS/MS	By MS/MS	By MS/MS	By MS/MS	By MS/MS	34,4	33,63	0	30,43
P23921	P23921	Ribonucleoside-diphosphate reductase large subunit	RRM1	22	22	22	By MS/MS	By MS/MS	By MS/MS	By MS/MS	By MS/MS	By MS/MS	By MS/MS	By MS/MS	By MS/MS	By MS/MS	By MS/MS	By MS/MS	43,7	90,07	0	233,84
P31350	P31350	Ribonucleoside-diphosphate reductase subunit M2	RRM2	6	6	5	By MS/MS	By MS/MS	By MS/MS	By MS/MS	By MS/MS	By MS/MS		By MS/MS	By MS/MS	By MS/MS	By MS/MS	By MS/MS	22,4	44,88	0	20,62
Q5JTH9	Q5JTH9	RRP12-like protein	RRP12	12	12	12	By MS/MS	By MS/MS	By MS/MS	By MS/MS	By MS/MS	By MS/MS	By MS/MS	By MS/MS	By MS/MS	By MS/MS	By MS/MS	By MS/MS	15,0	143,70	0	171,52
O76021	O76021	Ribosomal L1 domain-containing protein 1	RSL1D1	14	14	14	By MS/MS	By MS/MS	By MS/MS	By MS/MS	By MS/MS	By MS/MS	By MS/MS	By MS/MS	By MS/MS	By MS/MS	By MS/MS	By MS/MS	36,3	54,97	0	61,63
Q15404	Q15404	Ras suppressor protein 1	RSU1	5	5	5	By MS/MS	By MS/MS	By MS/MS	By MS/MS	By MS/MS	By MS/MS	By MS/MS	By MS/MS	By MS/MS			By MS/MS	24,5	31,54	0	17,91
Q9Y3I0	Q9Y3I0	tRNA-splicing ligase RtcB homolog	RTCB	14	14	14	By MS/MS	By MS/MS	By MS/MS	By MS/MS	By MS/MS	By MS/MS	By MS/MS	By MS/MS			By MS/MS	By MS/MS	37,8	55,21	0	209,99
O95197	O95197	Reticulon-3	RTN3	4	4	4	By MS/MS	By MS/MS	By MS/MS	By MS/MS	By MS/MS	By MS/MS	By MS/MS	By MS/MS	By MS/MS	By MS/MS	By MS/MS	By MS/MS	6,2	112,61	0	41,86
Q9NQC3	Q9NQC3	Reticulon-4	RTN4	7	7	7	By MS/MS	By MS/MS	By MS/MS	By MS/MS	By MS/MS	By MS/MS	By MS/MS	By MS/MS	By MS/MS	By MS/MS	By MS/MS	By MS/MS	9,3	129,93	0	92,34
Q9Y265	Q9Y265	RuvB-like 1	RUVBL1	18	18	18	By MS/MS	By MS/MS	By MS/MS	By MS/MS	By MS/MS	By MS/MS	By MS/MS	By MS/MS			By MS/MS	By MS/MS	45,6	50,23	0	143,42
Q9Y230	Q9Y230	RuvB-like 2	RUVBL2	22	22	22	By MS/MS	By MS/MS	By MS/MS	By MS/MS	By MS/MS	By MS/MS	By MS/MS	By MS/MS			By MS/MS	By MS/MS	59,8	51,16	0	160,45
P31949	P31949	Protein S100-A11;Protein S100-A11, N-terminally processed	S100A11	4	4	4	By MS/MS	By MS/MS	By MS/MS	By MS/MS	By MS/MS	By MS/MS	By MS/MS		By MS/MS	By MS/MS	By MS/MS	By MS/MS	38,1	11,74	0	157,48
Q9NTJ5	Q9NTJ5	Phosphatidylinositide phosphatase SAC1	SACM1L	5	5	5			By MS/MS	By MS/MS	By MS/MS	By MS/MS	By MS/MS	By MS/MS			By MS/MS		12,6	66,97	0	18,02
Q9UBE0	Q9UBE0	SUMO-activating enzyme subunit 1;SUMO-activating enzyme subunit 1, N-terminally processed	SAE1	14	14	14	By MS/MS	By MS/MS	By MS/MS	By MS/MS	By MS/MS	By MS/MS	By MS/MS	By MS/MS	By MS/MS	By MS/MS	By MS/MS	By MS/MS	54,0	38,45	0	323,31
Q9Y512	Q9Y512	Sorting and assembly machinery component 50 homolog	SAMM50	8	8	8	By MS/MS	By MS/MS	By MS/MS	By MS/MS	By MS/MS	By MS/MS					By MS/MS	By MS/MS	23,9	51,98	0	12,87
Q9NR31	Q9NR31	GTP-binding protein SAR1a	SAR1A	13	13	10	By MS/MS	By MS/MS	By MS/MS	By MS/MS	By MS/MS	By MS/MS	By MS/MS	By MS/MS	By MS/MS	By MS/MS	By MS/MS	By MS/MS	57,1	22,37	0	96,76
Q9Y6B6	Q9Y6B6	GTP-binding protein SAR1b	SAR1B	7	4	4	By MS/MS	By MS/MS	By matchin	By MS/MS	By MS/MS	By MS/MS	By matchin	By MS/MS	By MS/MS	By MS/MS	By MS/MS	By MS/MS	40,9	22,41	0	23,20
P82979	P82979	SAP domain-containing ribonucleoprotein	SARNP	5	5	5	By MS/MS	By MS/MS	By MS/MS	By MS/MS	By MS/MS	By MS/MS	By MS/MS	By MS/MS	By MS/MS	By MS/MS	By MS/MS	By MS/MS	28,1	23,67	0	308,69
P49591	P49591	Serine--tRNA ligase, cytoplasmic	SARS	21	21	21	By MS/MS	By MS/MS	By MS/MS	By MS/MS	By MS/MS	By MS/MS	By MS/MS	By MS/MS			By MS/MS	By MS/MS	47,5	58,78	0	323,31
Q9NP81	Q9NP81	Serine--tRNA ligase, mitochondrial	SARS2	5	5	5	By MS/MS			By MS/MS	By MS/MS	By MS/MS					By MS/MS	By MS/MS	16,4	58,28	0	24,60
Q15020	Q15020	Squamous cell carcinoma antigen recognized by T-cells 3	SART3	13	13	13	By MS/MS	By MS/MS	By MS/MS	By MS/MS	By MS/MS	By MS/MS		By MS/MS	By MS/MS	By MS/MS	By MS/MS	By MS/MS	19,6	109,93	0	58,34
Q9Y3A5	Q9Y3A5	Ribosome maturation protein SBDS	SBDS	8	8	8	By MS/MS	By MS/MS	By MS/MS	By MS/MS	By MS/MS	By MS/MS		By MS/MS	By MS/MS	By MS/MS	By MS/MS	By MS/MS	48,4	28,76	0	20,94
Q9UPN6	Q9UPN6	Protein SCAF8	SCAF8	4	4	3	By MS/MS	By MS/MS	By MS/MS	By MS/MS	By MS/MS	By MS/MS	By MS/MS	By MS/MS	By MS/MS	By MS/MS	By MS/MS	By MS/MS	5,7	140,52	0	12,18
Q8NBX0	Q8NBX0	Saccharopine dehydrogenase-like oxidoreductase	SCCPDH	11	11	11	By MS/MS	By MS/MS	By MS/MS	By MS/MS	By MS/MS	By MS/MS	By MS/MS	By MS/MS	By MS/MS	By MS/MS			39,2	47,15	0	44,69
Q8WVM8	Q8WVM8	Sec1 family domain-containing protein 1	SCFD1	11	11	11		By MS/MS	By MS/MS	By MS/MS	By MS/MS	By MS/MS	By MS/MS	By MS/MS	By MS/MS	By MS/MS	By MS/MS	By MS/MS	32,6	72,38	0	19,54

P22307	P22307	Non-specific lipid-transfer protein	SCP2	6	6	6	By MS/MS	By MS/MS	By MS/MS	By MS/MS	By MS/MS	By MS/MS			By MS/MS				9,7	58,99	0	18,72
Q6P3W7	Q6P3W7	SCY1-like protein 2	SCYL2	5	5	5						By MS/MS			By MS/MS	By MS/MS	By MS/MS		9,8	103,71	0,000344	2,67
O00560	O00560	Syntenin-1	SDCBP	5	5	5	By MS/MS	By MS/MS	By MS/MS	By MS/MS	By MS/MS	By MS/MS					By MS/MS		33,2	32,44	0	49,89
Q9HCN8	Q9HCN8	Stromal cell-derived factor 2-like protein 1	SDF2L1	5	5	5	By MS/MS	By MS/MS	By MS/MS	By MS/MS	By MS/MS	By MS/MS	By MS/MS	By MS/MS	By MS/MS	By MS/MS	By MS/MS	By MS/MS	43,9	23,60	0	122,21
P31040	P31040	Succinate dehydrogenase [ubiquinone] flavoprotein subunit, mitochondrial	SDHA	17	17	17	By MS/MS	By MS/MS	By MS/MS	By MS/MS	By MS/MS	By MS/MS	By MS/MS	By MS/MS	By MS/MS	By MS/MS	By MS/MS	By MS/MS	38,4	72,69	0	243,54
P21912	P21912	Succinate dehydrogenase [ubiquinone] iron-sulfur subunit, mitochondrial	SDHB	8	8	8	By MS/MS	By MS/MS	By MS/MS	By MS/MS	By MS/MS	By MS/MS	By MS/MS	By MS/MS	By MS/MS	By MS/MS	By MS/MS	By MS/MS	28,6	31,63	0	21,31
P67812	P67812	Signal peptidase complex catalytic subunit SEC11A	SEC11A	7	7	7	By MS/MS	By MS/MS	By MS/MS	By MS/MS	By MS/MS	By MS/MS	By MS/MS	By MS/MS		By MS/MS		By MS/MS	34,1	20,63	0	30,05
P55735	P55735	Protein SEC13 homolog	SEC13	9	9	9	By MS/MS	By MS/MS	By MS/MS	By MS/MS	By MS/MS	By MS/MS	By MS/MS	By MS/MS	By MS/MS	By MS/MS			51,2	35,54	0	274,37
O75396	O75396	Vesicle-trafficking protein SEC22b	SEC22B	10	10	10	By MS/MS	By MS/MS	By MS/MS	By MS/MS	By MS/MS	By MS/MS	By MS/MS	By MS/MS	By MS/MS	By MS/MS	By MS/MS	By MS/MS	43,7	24,59	0	192,75
Q15436	Q15436	Protein transport protein Sec23A	SEC23A	4	3	3		By MS/MS	By matchin	By MS/MS	By matchin	By MS/MS	By MS/MS		By MS/MS	By MS/MS	By MS/MS	By MS/MS	7,2	86,16	0	7,79
Q15437	Q15437	Protein transport protein Sec23B	SEC23B	11	11	10	By MS/MS	By MS/MS	By MS/MS	By MS/MS	By MS/MS	By MS/MS	By MS/MS	By MS/MS	By MS/MS	By MS/MS	By MS/MS	By MS/MS	21,6	86,48	0	142,36
P53992	P53992	Protein transport protein Sec24C	SEC24C	17	17	17	By MS/MS	By MS/MS	By MS/MS	By MS/MS	By MS/MS	By MS/MS	By MS/MS	By MS/MS	By MS/MS	By MS/MS	By MS/MS	By MS/MS	22,5	118,32	0	109,51
O94979	O94979	Protein transport protein Sec31A	SEC31A	15	15	15	By MS/MS	By MS/MS	By MS/MS	By MS/MS	By MS/MS	By MS/MS		By MS/MS	By MS/MS	By MS/MS	By MS/MS		17,5	133,01	0	127,38
Q9UGP8	Q9UGP8	Translocation protein SEC63 homolog	SEC63	4	4	4			By MS/MS	By MS/MS				By MS/MS					6,2	88,00	0,000346	2,72
P49903	P49903	Selenide, water dikinase 1	SEPHS1	4	4	4	By MS/MS	By MS/MS	By MS/MS	By MS/MS	By MS/MS	By MS/MS	By MS/MS	By MS/MS	By MS/MS	By MS/MS			15,6	42,91	0	234,49
Q9NVA2	Q9NVA2	Septin-11	SEPTIN11	8	8	6	By MS/MS	By MS/MS	By MS/MS	By MS/MS	By MS/MS	By MS/MS		By MS/MS			By MS/MS	By MS/MS	22,1	49,40	0	323,31
Q15019	Q15019	Septin-2	SEPTIN2	13	13	13	By MS/MS	By MS/MS	By MS/MS	By MS/MS	By MS/MS	By MS/MS	By MS/MS	By MS/MS	By MS/MS	By MS/MS			58,7	41,49	0	278,84
Q16181;Q6ZU15	Q16181	Septin-7	SEPTIN7	13	13	13	By MS/MS	By MS/MS	By MS/MS	By MS/MS	By MS/MS	By MS/MS	By MS/MS	By MS/MS			By MS/MS	By MS/MS	36,6	50,68	0	112,73
Q8NC51	Q8NC51	Plasminogen activator inhibitor 1 RNA-binding protein	SERBP1	9	9	9	By MS/MS	By MS/MS	By MS/MS	By MS/MS	By MS/MS	By MS/MS	By MS/MS	By MS/MS	By MS/MS	By MS/MS	By MS/MS	By MS/MS	25,2	44,97	0	135,89
P30740;O75830	P30740	Leukocyte elastase inhibitor	SERPINB1	15	14	14	By MS/MS	By MS/MS	By MS/MS	By MS/MS	By MS/MS	By MS/MS	By MS/MS		By MS/MS	By MS/MS			50,4	42,74	0	55,76
P35237	P35237	Serpin B6	SERPINB6	17	17	17	By MS/MS	By MS/MS	By MS/MS	By MS/MS	By MS/MS	By MS/MS	By MS/MS	By MS/MS	By MS/MS	By MS/MS	By MS/MS	By MS/MS	60,4	42,62	0	235,84
P50453;P50452	P50453	Serpin B9	SERPINB9	18	18	17	By MS/MS	By MS/MS	By MS/MS	By MS/MS	By MS/MS	By MS/MS	By MS/MS	By MS/MS	By MS/MS	By MS/MS			60,6	42,40	0	77,89
P50454	P50454	Serpin H1	SERPINH1	15	15	15	By MS/MS	By MS/MS	By MS/MS	By MS/MS	By MS/MS	By MS/MS	By MS/MS	By MS/MS	By MS/MS	By MS/MS	By MS/MS	By MS/MS	48,1	46,44	0	118,36
Q01105;P0DME0	Q01105;P0DME0	Protein SET;Protein SETSIP	SET;SETSIP	10	10	10	By MS/MS	By MS/MS	By MS/MS	By MS/MS	By MS/MS	By MS/MS	By MS/MS	By MS/MS	By MS/MS	By MS/MS			39,3	33,49	0	323,31
Q15637	Q15637	Splicing factor 1	SF1	10	10	10	By MS/MS	By MS/MS	By MS/MS	By MS/MS	By MS/MS	By MS/MS	By MS/MS	By MS/MS	By MS/MS	By MS/MS	By MS/MS	By MS/MS	18,3	68,33	0	29,01
Q15459	Q15459	Splicing factor 3A subunit 1	SF3A1	19	19	19	By MS/MS	By MS/MS	By MS/MS	By MS/MS	By MS/MS	By MS/MS	By MS/MS	By MS/MS	By MS/MS	By MS/MS	By MS/MS	By MS/MS	35,7	88,89	0	68,37
Q12874	Q12874	Splicing factor 3A subunit 3	SF3A3	12	12	12	By MS/MS	By MS/MS	By MS/MS	By MS/MS	By MS/MS	By MS/MS		By MS/MS			By MS/MS	By MS/MS	39,9	58,85	0	284,96
O75533	O75533	Splicing factor 3B subunit 1	SF3B1	32	32	32	By MS/MS	By MS/MS	By MS/MS	By MS/MS	By MS/MS	By MS/MS	By MS/MS		By MS/MS	By MS/MS	By MS/MS	By MS/MS	37,2	145,83	0	150,86
Q15393	Q15393	Splicing factor 3B subunit 3	SF3B3	34	34	34	By MS/MS	By MS/MS	By MS/MS	By MS/MS	By MS/MS	By MS/MS	By MS/MS	By MS/MS	By MS/MS	By MS/MS	By MS/MS	By MS/MS	38,7	135,58	0	323,31
Q9BWJ5	Q9BWJ5	Splicing factor 3B subunit 5	SF3B5	4	4	4	By MS/MS	By MS/MS	By MS/MS	By MS/MS	By MS/MS	By MS/MS	By MS/MS		By MS/MS	By MS/MS	By MS/MS	By MS/MS	46,5	10,14	0	21,08
Q9Y3B4	Q9Y3B4	Splicing factor 3B subunit 6	SF3B6	5	5	5	By MS/MS	By MS/MS	By MS/MS	By MS/MS	By MS/MS	By MS/MS	By MS/MS	By MS/MS	By MS/MS	By MS/MS	By MS/MS	By MS/MS	48,0	14,59	0	109,34
P23246	P23246	Splicing factor, proline- and glutamine-rich	SFPQ	16	16	15	By MS/MS	By MS/MS	By MS/MS	By MS/MS	By MS/MS	By MS/MS	By MS/MS	By MS/MS	By MS/MS	By MS/MS	By MS/MS	By MS/MS	30,3	76,15	0	315,13
Q9H9B4	Q9H9B4	Sideroflexin-1	SFXN1	12	12	11	By MS/MS	By MS/MS	By MS/MS	By MS/MS	By MS/MS	By MS/MS	By MS/MS	By MS/MS	By MS/MS	By MS/MS	By MS/MS	By MS/MS	55,3	35,62	0	146,89
O43765	O43765	Small glutamine-rich tetratricopeptide repeat-containing protein alpha	SGTA	10	10	10	By MS/MS	By MS/MS	By MS/MS	By MS/MS	By MS/MS	By MS/MS	By MS/MS	By MS/MS	By MS/MS	By MS/MS	By MS/MS	By MS/MS	35,1	34,06	0	36,23
O75368	O75368	SH3 domain-binding glutamic acid-rich-like protein	SH3BGRL	5	5	5	By MS/MS	By MS/MS	By MS/MS	By MS/MS	By MS/MS	By MS/MS	By MS/MS	By MS/MS	By MS/MS	By MS/MS	By MS/MS	By MS/MS	60,5	12,77	0	53,60
Q9H299	Q9H299	SH3 domain-binding glutamic acid-rich-like protein 3	SH3BGRL3	4	4	4	By MS/MS	By MS/MS			By MS/MS	By MS/MS	By MS/MS	By MS/MS	By MS/MS	By MS/MS	By MS/MS	By MS/MS	35,5	10,44	0	32,59
Q99961;Q99962	Q99961	Endophilin-A2	SH3GL1	5	5	5	By MS/MS	By MS/MS	By MS/MS	By MS/MS		By MS/MS	By MS/MS	By MS/MS	By MS/MS	By MS/MS			21,7	41,49	0	10,45
P34897;P34896	P34897	Serine hydroxymethyltransferase, mitochondrial	SHMT2	19	19	19	By MS/MS	By MS/MS	By MS/MS	By MS/MS	By MS/MS	By MS/MS	By MS/MS	By MS/MS			By MS/MS	By MS/MS	56,3	55,99	0	172,84
P42285	P42285	Superkiller viralicidic activity 2-like 2	SKIV2L2	22	22	22	By MS/MS	By MS/MS	By MS/MS	By MS/MS	By MS/MS	By MS/MS	By MS/MS	By MS/MS	By MS/MS	By MS/MS	By MS/MS	By MS/MS	28,3	117,80	0	95,90
P63208	P63208	S-phase kinase-associated protein 1	SKP1	11	11	11	By MS/MS	By MS/MS	By MS/MS	By MS/MS	By MS/MS	By MS/MS	By MS/MS	By MS/MS	By MS/MS	By MS/MS	By MS/MS	By MS/MS	71,8	18,66	0	145,95
P53985	P53985	Monocarboxylate transporter 1	SLC16A1	8	8	8	By MS/MS	By MS/MS	By MS/MS	By MS/MS	By MS/MS	By MS/MS		By MS/MS	By MS/MS	By MS/MS			23,0	53,94	0	30,52
Q15758	Q15758	Neutral amino acid transporter B(0)	SLC1A5	10	10	10	By MS/MS	By MS/MS	By MS/MS	By MS/MS	By MS/MS	By MS/MS	By MS/MS	By MS/MS	By MS/MS	By MS/MS	By MS/MS	By MS/MS	22,7	56,60	0	50,87
P53007	P53007	Tricarboxylate transport protein, mitochondrial	SLC25A1	9	9	9	By MS/MS	By MS/MS	By MS/MS	By MS/MS	By MS/MS	By MS/MS	By MS/MS	By MS/MS	By MS/MS	By MS/MS	By MS/MS	By MS/MS	32,2	34,01	0	27,56
Q9UBX3	Q9UBX3	Mitochondrial dicarboxylate carrier	SLC25A10	4	4	4	By MS/MS	By MS/MS	By MS/MS	By MS/MS	By MS/MS	By MS/MS		By MS/MS	By MS/MS	By MS/MS		By MS/MS	22,6	31,28	0	59,06
Q02978	Q02978	Mitochondrial 2-oxoglutarate/malate carrier protein	SLC25A11	10	10	10	By MS/MS	By MS/MS	By MS/MS	By MS/MS	By MS/MS	By MS/MS	By MS/MS	By MS/MS	By MS/MS	By MS/MS	By MS/MS	By MS/MS	47,8	34,06	0	60,27
O75746	O75746	Calcium-binding mitochondrial carrier protein Aralar1	SLC25A12	6	2	2	By matchin	By matchin	By matchin	By matchin	By MS/MS	By matching				By matchin	By matchin	By matchin	12,2	74,76	0	25,83
Q9UJS0	Q9UJS0	Calcium-binding mitochondrial carrier protein Aralar2	SLC25A13	14	14	10	By MS/MS	By MS/MS	By MS/MS	By MS/MS	By MS/MS	By MS/MS	By MS/MS	By MS/MS	By MS/MS	By MS/MS	By MS/MS	By MS/MS	33,6	74,18	0	274,29
Q9BQT8	Q9BQT8	Mitochondrial 2-oxodicarboxylate carrier	SLC25A21	4	4	4	By MS/MS	By MS/MS	By MS/MS	By MS/MS	By MS/MS	By MS/MS			By MS/MS	By MS/MS			17,4	33,30	0	49,89
Q9H936;Q9H1K4	Q9H936;Q9H1K4	Mitochondrial glutamate carrier 1;Mitochondrial glutamate carrier 2	SLC25A22;SLC25A18	4	4	4	By MS/MS	By MS/MS	By MS/MS	By MS/MS	By MS/MS	By MS/MS	By MS/MS	By MS/MS					13,6	34,47	0	52,57
Q6NUK1	Q6NUK1	Calcium-binding mitochondrial carrier protein SCaMC-1	SLC25A24	6	6	6		By MS/MS		By MS/MS	By MS/MS	By MS/MS			By MS/MS	By MS/MS			21,8	53,35	0	29,49
Q00325	Q00325	Phosphate carrier protein, mitochondrial	SLC25A3	10	10	10	By MS/MS	By MS/MS	By MS/MS	By MS/MS	By MS/MS	By MS/MS	By MS/MS	By MS/MS	By MS/MS	By MS/MS	By MS/MS	By MS/MS	35,9	40,09	0	203,21
P12235	P12235	ADP/ATP translocase 1	SLC25A4	13	3	3	By MS/MS	By matchin	By MS/MS	By matchin	By matchin	By matchin	By matchin	By matchin	By MS/MS	By MS/MS	By MS/MS	By matchin	44,6	33,06	0	9,56
P05141;Q9H0C2	P05141	ADP/ATP translocase 2;ADP/ATP translocase 2, N-terminally processed	SLC25A5	20	20	10	By MS/MS	By MS/MS	By MS/MS	By MS/MS	By MS/MS	By MS/MS	By MS/MS	By MS/MS	By MS/MS	By MS/MS	By MS/MS	By MS/MS	48,7	32,85	0	145,05
P12236	P12236	ADP/ATP translocase 3;ADP/ATP translocase 3, N-terminally processed	SLC25A6	17	7	4	By MS/MS	By MS/MS	By MS/MS	By MS/MS	By MS/MS	By MS/MS	By MS/MS	By MS/MS	By MS/MS	By MS/MS	By MS/MS	By MS/MS	52,3	32,87	0	28,77
P11166	P11166	Solute carrier family 2, facilitated glucose transporter member 1	SLC2A1	4	4	4	By MS/MS	By MS/MS	By MS/MS	By MS/MS	By MS/MS	By MS/MS	By MS/MS		By MS/MS	By MS/MS	By MS/MS		7,5	54,08	0	13,26
L0R6Q1	L0R6Q1		SLC35A4	8	8	8	By MS/MS	By MS/MS	By MS/MS		By MS/MS			By MS/MS	By MS/MS	By MS/MS	By MS/MS	By MS/MS	84,5	11,13	0	27,32
P08195	P08195	4F2 cell-surface antigen heavy chain	SLC3A2	25	25	25	By MS/MS	By MS/MS	By MS/MS	By MS/MS	By MS/MS	By MS/MS	By MS/MS	By MS/MS	By MS/MS	By MS/MS	By MS/MS	By MS/MS	43,8	67,99	0	323,31
O14745	O14745	Na(+)/H(+) exchange regulatory cofactor NHE-RF1	SLC9A3R1	4	4	4	By MS/MS	By MS/MS	By MS/MS			By MS/MS	By MS/MS			By MS/MS	By MS/MS		14,2	38,87	0	5,21
Q9GZT3	Q9GZT3	SRA stem-loop-interacting RNA-binding protein, mitochondrial	SLIRP	10	10	10	By MS/MS	By MS/MS	By MS/MS	By MS/MS	By MS/MS	By MS/MS	By MS/MS	By MS/MS	By MS/MS	By MS/MS	By MS/MS	By MS/MS	89,9	12,35	0	65,15
P51532;P51531	P51532	Transcription activator BRG1	SMARCA4	13	13	12	By MS/MS	By MS/MS	By MS/MS	By MS/MS	By MS/MS	By MS/MS	By MS/MS		By MS/MS	By MS/MS		By MS/MS	12,8	184,64	0	20,70
O60264	O60264	SWI/SNF-related matrix-associated actin-dependent regulator of chromatin subfamily A member 5	SMARCA5	14	14	10	By MS/MS	By MS/MS	By MS/MS	By MS/MS	By MS/MS	By MS/MS	By MS/MS		By MS/MS	By MS/MS	By MS/MS	By MS/MS	17,2	121,90	0	112,13
Q92922	Q92922	SWI/SNF complex subunit SMARCC1	SMARCC1	8	8	6	By MS/MS	By MS/MS	By MS/MS	By MS/MS	By MS/MS	By MS/MS	By MS/MS	By MS/MS	By MS/MS	By MS/MS			12,1	122,87	0	257,76
Q8TAQ2	Q8TAQ2	SWI/SNF complex subunit SMARCC2	SMARCC2	6	4	4		By MS/MS	By MS/MS	By MS/MS	By MS/MS	By MS/MS		By MS/MS	By matching				7,0	132,88	0	108,45
Q14683	Q14683	Structural maintenance of chromosomes protein 1A	SMC1A	11	11	11	By MS/MS	By MS/MS	By MS/MS	By MS/MS		By MS/MS	By MS/MS	By MS/MS	By MS/MS	By MS/MS	By MS/MS	By MS/MS	11,9	143,23	0	39,54
O95347	O95347	Structural maintenance of chromosomes protein 2	SMC2	14	14	14	By MS/MS	By MS/MS	By MS/MS	By MS/MS	By MS/MS	By MS/MS	By MS/MS	By MS/MS	By MS/MS	By MS/MS	By MS/MS	By MS/MS	16,3	135,65	0	30,90
Q9UQE7	Q9UQE7	Structural maintenance of chromosomes protein 3	SMC3	14	14	14	By MS/MS	By MS/MS	By MS/MS	By MS/MS		By MS/MS	By MS/MS	By MS/MS	By MS/MS	By MS/MS	By MS/MS	By MS/MS	16,4	141,54	0	43,66
Q9NTJ3	Q9NTJ3	Structural maintenance of chromosomes protein 4	SMC4	10	10	10		By MS/MS	By MS/MS	By MS/MS	By MS/MS	By MS/MS	By MS/MS		By MS/MS	By MS/MS	By MS/MS	By MS/MS	11,6	147,18	0	10,20
A6NHR9	A6NHR9	Structural maintenance of chromosomes flexible hinge domain-containing protein 1	SMCHD1	5	5	5	By MS/MS		By MS/MS	By MS/MS			By MS/MS	By MS/MS	By MS/MS	By MS/MS	By MS/MS	By MS/MS	3,5	226,37	0	11,66

P52788	P52788	Spermine synthase	SMS	9	9	9	By MS/MS	By MS/MS	By MS/MS	By MS/MS	By MS/MS	By MS/MS	By MS/MS	By MS/MS	By MS/MS	By MS/MS			38,8	41,27	0	59,21
Q2TAY7	Q2TAY7	WD40 repeat-containing protein SMU1;WD40 repeat-containing protein SMU1, N-terminally processed	SMU1	4	4	4				By MS/MS		By MS/MS	By MS/MS	By MS/MS			By MS/MS	By MS/MS	10,5	57,54	0	5,68
O95721	O95721	Synaptosomal-associated protein 29	SNAP29	7	7	7	By MS/MS	By MS/MS	By MS/MS	By MS/MS	By MS/MS	By MS/MS	By MS/MS	By MS/MS	By MS/MS	By MS/MS	By MS/MS	By MS/MS	38,4	28,97	0	17,07
O95295	O95295	SNARE-associated protein Snapin	SNAPIN	4	4	4	By MS/MS	By MS/MS	By MS/MS	By MS/MS	By MS/MS	By MS/MS	By MS/MS	By MS/MS	By MS/MS	By MS/MS	By MS/MS	By MS/MS	33,8	14,87	0	229,61
Q7KZF4	Q7KZF4	Staphylococcal nuclease domain-containing protein 1	SND1	41	41	41	By MS/MS	By MS/MS	By MS/MS	By MS/MS	By MS/MS	By MS/MS	By MS/MS	By MS/MS	By MS/MS	By MS/MS	By MS/MS	By MS/MS	53,3	102,00	0	323,31
O75643	O75643	U5 small nuclear ribonucleoprotein 200 kDa helicase	SNRNP200	58	58	58	By MS/MS	By MS/MS	By MS/MS	By MS/MS	By MS/MS	By MS/MS	By MS/MS	By MS/MS	By MS/MS	By MS/MS	By MS/MS	By MS/MS	38,2	244,50	0	323,31
P09012	P09012	U1 small nuclear ribonucleoprotein A	SNRPA	6	6	5	By MS/MS	By MS/MS	By MS/MS		By MS/MS	By MS/MS	By MS/MS	By MS/MS	By MS/MS		By MS/MS	By MS/MS	29,4	31,28	0	32,84
P09661	P09661	U2 small nuclear ribonucleoprotein A	SNRPA1	11	11	11	By MS/MS	By MS/MS	By MS/MS	By MS/MS	By MS/MS	By MS/MS	By MS/MS	By MS/MS	By MS/MS	By MS/MS	By MS/MS	By MS/MS	38,8	28,42	0	150,75
P14678;P63162	P14678;P63162	Small nuclear ribonucleoprotein-associated proteins B and B;Small nuclear ribonucleoprotein-associated protein N	SNRPB;SNRPN	9	9	9	By MS/MS	By MS/MS	By MS/MS	By MS/MS	By MS/MS	By MS/MS	By MS/MS	By MS/MS	By MS/MS	By MS/MS	By MS/MS	By MS/MS	32,1	24,61	0	47,18
P08579	P08579	U2 small nuclear ribonucleoprotein B	SNRPB2	5	4	4	By MS/MS	By MS/MS	By MS/MS	By MS/MS	By MS/MS	By MS/MS	By matching		By matching		By matchin	By matchin	35,6	25,49	0	21,73
P62314	P62314	Small nuclear ribonucleoprotein Sm D1	SNRPD1	5	5	5	By MS/MS	By MS/MS	By MS/MS	By MS/MS	By MS/MS	By MS/MS	By MS/MS	By MS/MS	By MS/MS	By MS/MS	By MS/MS	By MS/MS	54,6	13,28	0	56,98
P62316	P62316	Small nuclear ribonucleoprotein Sm D2	SNRPD2	13	13	13	By MS/MS	By MS/MS	By MS/MS	By MS/MS	By MS/MS	By MS/MS	By MS/MS	By MS/MS	By MS/MS	By MS/MS	By MS/MS	By MS/MS	74,6	13,53	0	323,31
P62318	P62318	Small nuclear ribonucleoprotein Sm D3	SNRPD3	7	7	7	By MS/MS	By MS/MS	By MS/MS	By MS/MS	By MS/MS	By MS/MS	By MS/MS	By MS/MS	By MS/MS	By MS/MS	By MS/MS	By MS/MS	56,3	13,92	0	179,67
P62304	P62304	Small nuclear ribonucleoprotein E	SNRPE	6	6	6	By MS/MS	By MS/MS	By MS/MS	By MS/MS	By MS/MS	By MS/MS	By MS/MS	By MS/MS	By MS/MS	By MS/MS	By MS/MS	By MS/MS	72,8	10,80	0	231,95
P62306	P62306	Small nuclear ribonucleoprotein F	SNRPF	6	6	6	By MS/MS	By MS/MS	By MS/MS	By MS/MS	By MS/MS	By MS/MS	By MS/MS	By MS/MS	By MS/MS	By MS/MS	By MS/MS	By MS/MS	48,8	9,73	0	29,30
P62308;A8MWD9	P62308;A8MWD9	Small nuclear ribonucleoprotein G;Putative small nuclear ribonucleoprotein G-like protein 15	SNRPG;SNRPGP15	4	4	4	By MS/MS	By MS/MS			By MS/MS		By MS/MS	By MS/MS	By MS/MS	By MS/MS	By MS/MS	By MS/MS	36,8	8,50	0	33,98
O60749	O60749	Sorting nexin-2	SNX2	4	4	3	By MS/MS	By MS/MS	By MS/MS	By MS/MS	By MS/MS	By MS/MS				By MS/MS	By MS/MS	By MS/MS	9,2	58,47	0	52,76
O60493	O60493	Sorting nexin-3	SNX3	11	11	10	By MS/MS	By MS/MS	By MS/MS	By MS/MS	By MS/MS	By MS/MS	By MS/MS	By MS/MS	By MS/MS	By MS/MS	By MS/MS	By MS/MS	65,4	18,76	0	21,44
Q9Y5X3	Q9Y5X3	Sorting nexin-5	SNX5	5	5	5	By MS/MS	By MS/MS	By MS/MS	By MS/MS	By MS/MS	By MS/MS		By MS/MS			By MS/MS	By MS/MS	13,9	46,82	0	37,33
Q9UNH7;Q86XE0	Q9UNH7	Sorting nexin-6;Sorting nexin-6, N-terminally processed	SNX6	7	7	7	By MS/MS	By MS/MS	By MS/MS	By MS/MS	By MS/MS	By MS/MS		By MS/MS	By MS/MS	By MS/MS		By MS/MS	27,3	46,65	0	167,00
Q9Y5X1	Q9Y5X1	Sorting nexin-9	SNX9	4	4	4		By MS/MS		By MS/MS		By MS/MS	By MS/MS	By MS/MS			By MS/MS	By MS/MS	11,8	66,59	0	12,91
P00441;P00442	P00441	Superoxide dismutase [Cu-Zn]	SOD1	10	10	10	By MS/MS	By MS/MS	By MS/MS	By MS/MS	By MS/MS	By MS/MS	By MS/MS	By MS/MS	By MS/MS	By MS/MS	By MS/MS	By MS/MS	84,4	15,94	0	179,76
P04179	P04179	Superoxide dismutase [Mn], mitochondrial	SOD2	9	9	9	By MS/MS	By MS/MS	By MS/MS	By MS/MS	By MS/MS	By MS/MS		By MS/MS	By MS/MS		By MS/MS	By MS/MS	35,1	24,72	0	73,08
Q00796	Q00796	Sorbitol dehydrogenase	SORD	4	4	4		By MS/MS	By MS/MS	By MS/MS	By MS/MS		By MS/MS	By MS/MS	By MS/MS	By MS/MS		By MS/MS	22,1	38,32	0	23,71
Q99523	Q99523	Sortilin	SORT1	4	4	4	By MS/MS	By MS/MS	By MS/MS	By MS/MS	By MS/MS	By MS/MS	By MS/MS	By MS/MS	By MS/MS	By MS/MS			8,1	92,07	0	16,37
O60271	O60271	C-Jun-amino-terminal kinase-interacting protein 4	SPAG9	7	7	7	By MS/MS	By MS/MS	By MS/MS	By MS/MS	By MS/MS	By MS/MS	By MS/MS		By MS/MS	By MS/MS	By MS/MS	By MS/MS	9,1	146,20	0	133,66
Q8NBT2	Q8NBT2	Kinetochore protein Spc24	SPC24	5	5	5	By MS/MS	By MS/MS	By MS/MS	By MS/MS	By MS/MS	By MS/MS		By MS/MS	By MS/MS	By MS/MS		By MS/MS	46,2	22,48	0	34,11
Q9HBM1	Q9HBM1	Kinetochore protein Spc25	SPC25	4	4	4	By MS/MS	By MS/MS	By MS/MS	By MS/MS	By MS/MS	By MS/MS	By MS/MS			By MS/MS		By MS/MS	25,0	26,15	0	12,16
Q15005	Q15005	Signal peptidase complex subunit 2	SPCS2	8	8	8	By MS/MS	By MS/MS	By MS/MS	By MS/MS	By MS/MS	By MS/MS	By MS/MS	By MS/MS	By MS/MS	By MS/MS	By MS/MS	By MS/MS	35,8	25,00	0	42,08
P35270	P35270	Sepiapterin reductase	SPR	8	8	8	By MS/MS	By MS/MS	By MS/MS	By MS/MS	By MS/MS	By MS/MS	By MS/MS	By MS/MS	By MS/MS	By MS/MS	By MS/MS	By MS/MS	49,8	28,05	0	60,57
P02549	P02549	Spectrin alpha chain, erythrocytic 1	SPTA1	17	17	17	By MS/MS	By MS/MS	By MS/MS	By MS/MS	By MS/MS	By MS/MS		By MS/MS		By MS/MS			12,8	280,01	0	122,71
O15269	O15269	Serine palmitoyltransferase 1	SPTLC1	7	7	7	By MS/MS	By MS/MS	By MS/MS	By MS/MS	By MS/MS	By MS/MS					By MS/MS	By MS/MS	26,6	52,74	0	119,29
Q13501	Q13501	Sequestosome-1	SQSTM1	4	4	4	By MS/MS		By MS/MS	By MS/MS	By MS/MS	By MS/MS					By MS/MS		13,4	47,69	0	49,64
P30626	P30626	Sorcin	SRI	10	10	10	By MS/MS	By MS/MS	By MS/MS	By MS/MS	By MS/MS	By MS/MS	By MS/MS	By MS/MS	By MS/MS	By MS/MS	By MS/MS	By MS/MS	59,1	21,68	0	77,46
P19623	P19623	Spermidine synthase	SRM	12	12	12	By MS/MS	By MS/MS	By MS/MS	By MS/MS	By MS/MS	By MS/MS	By MS/MS	By MS/MS	By MS/MS	By MS/MS	By MS/MS	By MS/MS	56,6	33,82	0	60,25
P37108	P37108	Signal recognition particle 14 kDa protein	SRP14	8	8	8	By MS/MS	By MS/MS	By MS/MS	By MS/MS	By MS/MS	By MS/MS	By MS/MS	By MS/MS	By MS/MS	By MS/MS	By MS/MS	By MS/MS	47,1	14,57	0	53,07
P09132	P09132	Signal recognition particle 19 kDa protein	SRP19	9	9	9	By MS/MS	By MS/MS	By MS/MS	By MS/MS		By MS/MS	By MS/MS	By MS/MS	By MS/MS	By MS/MS	By MS/MS	By MS/MS	52,8	16,16	0	63,99
P61011	P61011	Signal recognition particle 54 kDa protein	SRP54	5	5	5	By MS/MS	By MS/MS	By MS/MS	By MS/MS	By MS/MS	By MS/MS						By MS/MS	13,9	55,70	0	32,05
Q9UHB9	Q9UHB9	Signal recognition particle subunit SRP68	SRP68	12	12	12	By MS/MS	By MS/MS	By MS/MS	By MS/MS	By MS/MS	By MS/MS			By MS/MS		By MS/MS	By MS/MS	23,4	70,73	0	133,60
O76094	O76094	Signal recognition particle subunit SRP72	SRP72	11	11	11	By MS/MS	By MS/MS	By MS/MS	By MS/MS	By MS/MS	By MS/MS	By MS/MS		By MS/MS	By MS/MS	By MS/MS	By MS/MS	26,1	74,61	0	52,65
P49458	P49458	Signal recognition particle 9 kDa protein	SRP9	8	8	8	By MS/MS	By MS/MS	By MS/MS	By MS/MS	By MS/MS		By MS/MS	By MS/MS	By MS/MS	By MS/MS	By MS/MS	By MS/MS	64,0	10,11	0	24,79
Q96SB4	Q96SB4	SRSF protein kinase 1	SRPK1	7	7	7	By MS/MS	By MS/MS	By MS/MS	By MS/MS	By MS/MS	By MS/MS	By MS/MS	By MS/MS					14,4	74,32	0	24,52
P08240	P08240	Signal recognition particle receptor subunit alpha	SRPR	7	7	7		By MS/MS	By MS/MS	By MS/MS		By MS/MS		By MS/MS			By MS/MS	By MS/MS	17,6	69,81	0	10,88
Q9Y5M8	Q9Y5M8	Signal recognition particle receptor subunit beta	SRPRB	9	9	9	By MS/MS	By MS/MS	By MS/MS	By MS/MS	By MS/MS	By MS/MS	By MS/MS	By MS/MS	By MS/MS	By MS/MS	By MS/MS	By MS/MS	43,9	29,70	0	234,48
Q9BXP5	Q9BXP5	Serrate RNA effector molecule homolog	SRRT	11	11	11	By MS/MS	By MS/MS	By MS/MS	By MS/MS	By MS/MS	By MS/MS	By MS/MS	By MS/MS	By MS/MS	By MS/MS	By MS/MS	By MS/MS	15,9	100,67	0	21,75
Q07955	Q07955	Serine/arginine-rich splicing factor 1	SRSF1	12	12	11	By MS/MS	By MS/MS	By MS/MS	By MS/MS	By MS/MS	By MS/MS	By MS/MS	By MS/MS	By MS/MS	By MS/MS	By MS/MS	By MS/MS	48,0	27,74	0	68,62
Q01130;Q9BRL6	Q01130	Serine/arginine-rich splicing factor 2	SRSF2	5	5	5	By MS/MS	By MS/MS	By MS/MS	By MS/MS	By MS/MS	By MS/MS	By MS/MS	By MS/MS	By MS/MS	By MS/MS	By MS/MS	By MS/MS	24,0	25,48	0	305,53
P84103	P84103	Serine/arginine-rich splicing factor 3	SRSF3	6	5	5	By MS/MS	By MS/MS	By MS/MS	By MS/MS	By MS/MS	By MS/MS	By MS/MS	By MS/MS	By MS/MS	By MS/MS	By MS/MS	By MS/MS	34,8	19,33	0	21,67
Q13247	Q13247	Serine/arginine-rich splicing factor 6	SRSF6	4	4	3	By MS/MS	By MS/MS	By MS/MS	By MS/MS	By MS/MS	By MS/MS	By MS/MS	By MS/MS	By MS/MS	By MS/MS	By MS/MS	By MS/MS	11,0	39,59	0	3,90
Q16629	Q16629	Serine/arginine-rich splicing factor 7	SRSF7	7	7	6	By MS/MS	By MS/MS	By MS/MS	By MS/MS	By MS/MS	By MS/MS	By MS/MS	By MS/MS	By MS/MS	By MS/MS	By MS/MS	By MS/MS	29,8	27,37	0	43,39
Q13242	Q13242	Serine/arginine-rich splicing factor 9	SRSF9	5	4	4	By MS/MS	By MS/MS	By MS/MS	By MS/MS	By MS/MS	By MS/MS	By MS/MS	By MS/MS			By MS/MS	By MS/MS	24,4	25,54	0	6,67
P05455	P05455	Lupus La protein	SSB	21	21	21	By MS/MS	By MS/MS	By MS/MS	By MS/MS	By MS/MS	By MS/MS	By MS/MS	By MS/MS	By MS/MS	By MS/MS	By MS/MS	By MS/MS	47,1	46,84	0	286,99
Q04837	Q04837	Single-stranded DNA-binding protein, mitochondrial	SSBP1	15	15	15	By MS/MS	By MS/MS	By MS/MS	By MS/MS	By MS/MS	By MS/MS	By MS/MS	By MS/MS	By MS/MS	By MS/MS	By MS/MS	By MS/MS	75,0	17,26	0	323,31
P43307	P43307	Translocon-associated protein subunit alpha	SSR1	4	4	4	By MS/MS	By MS/MS	By MS/MS	By MS/MS	By MS/MS	By MS/MS			By MS/MS	By MS/MS	By MS/MS		21,0	32,24	0	103,64
P51571	P51571	Translocon-associated protein subunit delta	SSR4	6	6	6	By MS/MS	By MS/MS	By MS/MS	By MS/MS	By MS/MS	By MS/MS	By MS/MS	By MS/MS	By MS/MS	By MS/MS	By MS/MS	By MS/MS	37,0	19,00	0	114,08
Q08945	Q08945	FACT complex subunit SSRP1	SSRP1	5	5	5	By MS/MS	By MS/MS	By MS/MS	By MS/MS	By MS/MS	By MS/MS	By MS/MS	By MS/MS	By MS/MS	By MS/MS	By MS/MS	By MS/MS	10,9	81,07	0	28,61
P50502;Q8IZP2;Q8NFI4	P50502;Q8IZP2;Q8NFI4	Hsc70-interacting protein;Putative protein FAM10A4;Putative protein FAM10A5	ST13;ST13P4;ST13P5	7	7	7	By MS/MS	By MS/MS	By MS/MS	By MS/MS	By MS/MS	By MS/MS	By MS/MS	By MS/MS	By MS/MS	By MS/MS	By MS/MS	By MS/MS	23,0	41,33	0	75,22
P42224	P42224	Signal transducer and activator of transcription 1-alpha/beta	STAT1	4	4	4		By MS/MS	By MS/MS		By MS/MS	By MS/MS	By MS/MS	By MS/MS					7,9	87,33	0	22,17
P40763	P40763	Signal transducer and activator of transcription 3	STAT3	9	9	9	By MS/MS	By MS/MS	By MS/MS	By MS/MS	By MS/MS	By MS/MS	By MS/MS	By MS/MS	By MS/MS	By MS/MS		By MS/MS	20,3	88,07	0	105,87
P42229	P42229	Signal transducer and activator of transcription 5A	STAT5A	24	24	9	By MS/MS	By MS/MS	By MS/MS	By MS/MS	By MS/MS	By MS/MS	By MS/MS	By MS/MS	By MS/MS	By MS/MS	By MS/MS	By MS/MS	37,4	90,65	0	323,31
P51692	P51692	Signal transducer and activator of transcription 5B	STAT5B	20	5	5	By MS/MS	By MS/MS	By MS/MS	By MS/MS	By MS/MS	By MS/MS	By MS/MS	By MS/MS	By MS/MS	By MS/MS	By MS/MS	By MS/MS	33,7	89,87	0	83,41
P31948	P31948	Stress-induced-phosphoprotein 1	STIP1	37	37	37	By MS/MS	By MS/MS	By MS/MS	By MS/MS	By MS/MS	By MS/MS	By MS/MS	By MS/MS			By MS/MS	By MS/MS	54,1	62,64	0	323,31
P16949;Q9H169	P16949	Stathmin	STMN1	11	11	8	By MS/MS	By MS/MS	By MS/MS	By MS/MS	By MS/MS	By MS/MS	By MS/MS	By MS/MS	By MS/MS	By MS/MS	By MS/MS	By MS/MS	44,3	17,30	0	315,06
Q9UJZ1	Q9UJZ1	Stomatin-like protein 2, mitochondrial	STOML2	14	14	14	By MS/MS	By MS/MS	By MS/MS	By MS/MS	By MS/MS	By MS/MS	By MS/MS	By MS/MS	By MS/MS	By MS/MS	By MS/MS	By MS/MS	64,9	38,53	0	323,31
Q9Y3F4	Q9Y3F4	Serine-threonine kinase receptor-associated protein	STRAP	13	13	13	By MS/MS	By MS/MS	By MS/MS	By MS/MS	By MS/MS	By MS/MS	By MS/MS	By MS/MS	By MS/MS	By MS/MS			47,7	38,44	0	323,31
P46977	P46977	Dolichyl-diphosphooligosaccharide--protein glycosyltransferase subunit STT3A	STT3A	13	13	13	By MS/MS	By MS/MS	By MS/MS	By MS/MS	By MS/MS	By MS/MS	By MS/MS	By MS/MS	By MS/MS	By MS/MS	By MS/MS	By MS/MS	22,8	80,53	0	67,86
Q9UNE7	Q9UNE7	E3 ubiquitin-protein ligase CHIP	STUB1	4	4	4	By MS/MS	By MS/MS	By MS/MS	By MS/MS	By MS/MS	By MS/MS	By MS/MS	By MS/MS	By MS/MS	By MS/MS	By MS/MS	By MS/MS	17,2	34,86	0	17,78
Q15833	Q15833	Syntaxin-binding protein 2	STXBP2	9	9	9	By MS/MS	By MS/MS		By MS/MS	By MS/MS	By MS/MS	By MS/MS	By MS/MS	By MS/MS	By MS/MS	By MS/MS	By MS/MS	22,1	66,45	0	20,23
P53999	P53999	Activated RNA polymerase II transcriptional coactivator p15	SUB1	6	6	6	By MS/MS	By MS/MS	By MS/MS	By MS/MS	By MS/MS	By MS/MS	By MS/MS	By MS/MS	By MS/MS	By MS/MS	By MS/MS	By MS/MS	37,8	14,40	0	287,20

Q9P2R7	Q9P2R7	Succinyl-CoA ligase [ADP-forming] subunit beta, mitochondrial	SUCLA2	5	5	5	By MS/MS	By MS/MS	By MS/MS	By MS/MS	By MS/MS	By MS/MS					By MS/MS		14,7	50,32	0	5,54
P53597	P53597	Succinyl-CoA ligase [ADP/GDP-forming] subunit alpha, mitochondrial	SUCLG1	5	5	5	By MS/MS	By MS/MS	By MS/MS	By MS/MS	By MS/MS	By MS/MS	By MS/MS	By MS/MS	By MS/MS	By MS/MS	By MS/MS	By MS/MS	21,4	36,25	0	91,59
Q96I99	Q96I99	Succinyl-CoA ligase [GDP-forming] subunit beta, mitochondrial	SUCLG2	8	8	8	By MS/MS	By MS/MS	By MS/MS	By MS/MS	By MS/MS	By MS/MS		By MS/MS			By MS/MS	By MS/MS	25,7	46,51	0	28,59
Q9Y2Z0	Q9Y2Z0	Suppressor of G2 allele of SKP1 homolog	SUGT1	8	8	8	By MS/MS	By MS/MS	By MS/MS	By MS/MS	By MS/MS	By MS/MS	By MS/MS	By MS/MS	By MS/MS	By MS/MS		By MS/MS	29,0	41,02	0	84,46
Q9Y5B9	Q9Y5B9	FACT complex subunit SPT16	SUPT16H	5	5	5		By MS/MS	By MS/MS	By MS/MS	By MS/MS	By MS/MS	By MS/MS	By MS/MS	By MS/MS	By MS/MS	By MS/MS	By MS/MS	6,4	119,91	0	12,93
O00267	O00267	Transcription elongation factor SPT5	SUPT5H	11	11	11	By MS/MS	By MS/MS	By MS/MS	By MS/MS	By MS/MS	By MS/MS				By MS/MS			16,3	121,00	0	66,58
Q8IYB8	Q8IYB8	ATP-dependent RNA helicase SUPV3L1, mitochondrial	SUPV3L1	5	5	5		By MS/MS				By MS/MS			By MS/MS	By MS/MS	By MS/MS	By MS/MS	9,7	87,99	0	10,96
O15260	O15260	Surfeit locus protein 4	SURF4	6	6	6	By MS/MS	By MS/MS	By MS/MS	By MS/MS	By MS/MS	By MS/MS	By MS/MS	By MS/MS	By MS/MS	By MS/MS	By MS/MS	By MS/MS	27,5	30,39	0	180,99
Q92797	Q92797	Symplekin	SYMPK	12	12	12	By MS/MS	By MS/MS	By MS/MS	By MS/MS	By MS/MS	By MS/MS	By MS/MS				By MS/MS	By MS/MS	18,6	141,15	0	54,41
O60506	O60506	Heterogeneous nuclear ribonucleoprotein Q	SYNCRIP	24	24	19	By MS/MS	By MS/MS	By MS/MS	By MS/MS	By MS/MS	By MS/MS	By MS/MS	By MS/MS	By MS/MS	By MS/MS	By MS/MS	By MS/MS	40,8	69,60	0	312,65
Q92804	Q92804	TATA-binding protein-associated factor 2N	TAF15	4	2	2		By MS/MS		By matchin	By matching		By MS/MS	By MS/MS	By matchin	By matchin	By matchin	By matchin	9,8	61,83	0	9,74
P37802;Q9UI15	P37802	Transgelin-2	TAGLN2	22	22	22	By MS/MS	By MS/MS	By MS/MS	By MS/MS	By MS/MS	By MS/MS	By MS/MS	By MS/MS	By MS/MS	By MS/MS	By MS/MS	By MS/MS	90,5	22,39	0	323,31
P37837	P37837	Transaldolase	TALDO1	22	22	22	By MS/MS	By MS/MS	By MS/MS	By MS/MS	By MS/MS	By MS/MS	By MS/MS	By MS/MS	By MS/MS	By MS/MS	By MS/MS	By MS/MS	52,5	37,54	0	323,31
Q96BW9	Q96BW9	Phosphatidate cytidylyltransferase, mitochondrial	TAMM41	5	5	5	By MS/MS	By MS/MS	By MS/MS	By MS/MS	By MS/MS	By MS/MS		By MS/MS	By MS/MS	By MS/MS	By MS/MS	By MS/MS	12,4	51,07	0	83,78
Q13148	Q13148	TAR DNA-binding protein 43	TARDBP	8	8	8	By MS/MS	By MS/MS	By MS/MS	By MS/MS	By MS/MS	By MS/MS	By MS/MS	By MS/MS	By MS/MS	By MS/MS		By MS/MS	35,3	44,74	0	307,49
P26639;A2RTX5	P26639	Threonine--tRNA ligase, cytoplasmic	TARS	31	31	31	By MS/MS	By MS/MS	By MS/MS	By MS/MS	By MS/MS	By MS/MS	By MS/MS	By MS/MS	By MS/MS	By MS/MS	By MS/MS	By MS/MS	46,9	83,43	0	205,24
O75347	O75347	Tubulin-specific chaperone A	TBCA	10	10	10	By MS/MS	By MS/MS	By MS/MS	By MS/MS	By MS/MS	By MS/MS	By MS/MS	By MS/MS		By MS/MS	By MS/MS		62,0	12,86	0	154,28
Q9BTW9	Q9BTW9	Tubulin-specific chaperone D	TBCD	13	13	13	By MS/MS	By MS/MS	By MS/MS	By MS/MS	By MS/MS	By MS/MS	By MS/MS				By MS/MS		16,4	132,60	0	24,54
Q15813	Q15813	Tubulin-specific chaperone E	TBCE	9	9	9	By MS/MS	By MS/MS	By MS/MS	By MS/MS	By MS/MS	By MS/MS		By MS/MS		By MS/MS	By MS/MS	By MS/MS	31,1	59,35	0	55,06
Q9BZK7;O60907;Q9BQ87	Q9BZK7;O60907	F-box-like/WD repeat-containing protein TBL1XR1;F-box-like/WD repeat-containing protein TBL1X	TBL1XR1;TBL1X	5	5	5	By MS/MS	By MS/MS	By MS/MS	By MS/MS	By MS/MS	By MS/MS			By MS/MS		By MS/MS	By MS/MS	18,1	55,59	0	17,13
Q9Y4P3	Q9Y4P3	Transducin beta-like protein 2	TBL2	4	4	4	By MS/MS		By MS/MS				By MS/MS	By MS/MS	By MS/MS	By MS/MS		By MS/MS	13,6	49,80	0	40,58
Q12788	Q12788	Transducin beta-like protein 3	TBL3	5	5	5					By MS/MS	By MS/MS	By MS/MS	By MS/MS	By MS/MS	By MS/MS	By MS/MS	By MS/MS	10,1	89,03	0	11,16
Q969Z0	Q969Z0	Protein TBRG4	TBRG4	17	17	17	By MS/MS	By MS/MS	By MS/MS	By MS/MS	By MS/MS	By MS/MS		By MS/MS			By MS/MS	By MS/MS	50,6	70,74	0	60,81
P23193;Q15560	P23193	Transcription elongation factor A protein 1	TCEA1	9	9	9	By MS/MS		By MS/MS	By MS/MS	By MS/MS	By MS/MS	By MS/MS	By MS/MS	By MS/MS	By MS/MS			37,5	33,97	0	24,11
Q15369	Q15369	Transcription elongation factor B polypeptide 1	TCEB1	6	6	6	By MS/MS	By MS/MS	By MS/MS	By MS/MS	By MS/MS	By MS/MS	By MS/MS	By MS/MS	By MS/MS	By MS/MS	By MS/MS	By MS/MS	54,5	12,47	0	84,56
Q15370	Q15370	Transcription elongation factor B polypeptide 2	TCEB2	8	8	8	By MS/MS	By MS/MS	By MS/MS	By MS/MS	By MS/MS	By MS/MS	By MS/MS	By MS/MS	By MS/MS	By MS/MS	By MS/MS	By MS/MS	59,3	13,13	0	19,29
O14776	O14776	Transcription elongation regulator 1	TCERG1	10	10	10	By MS/MS	By MS/MS	By MS/MS	By MS/MS	By MS/MS	By MS/MS	By MS/MS				By MS/MS	By MS/MS	11,9	123,90	0	16,01
P17987	P17987	T-complex protein 1 subunit alpha	TCP1	39	39	39	By MS/MS	By MS/MS	By MS/MS	By MS/MS	By MS/MS	By MS/MS	By MS/MS	By MS/MS	By MS/MS	By MS/MS	By MS/MS	By MS/MS	82,6	60,34	0	323,31
Q9NZ01	Q9NZ01	Very-long-chain enoyl-CoA reductase	TECR	8	8	8	By MS/MS	By MS/MS	By MS/MS	By MS/MS	By MS/MS	By MS/MS	By MS/MS	By MS/MS	By MS/MS	By MS/MS	By MS/MS	By MS/MS	23,7	36,03	0	16,21
Q9NXF1	Q9NXF1	Testis-expressed sequence 10 protein	TEX10	4	4	4	By MS/MS	By MS/MS	By MS/MS	By MS/MS		By MS/MS	By MS/MS		By MS/MS	By MS/MS	By MS/MS	By MS/MS	6,6	105,67	0	9,42
Q00059	Q00059	Transcription factor A, mitochondrial	TFAM	8	8	8	By MS/MS	By MS/MS	By MS/MS	By MS/MS	By MS/MS	By MS/MS	By MS/MS	By MS/MS	By MS/MS	By MS/MS	By MS/MS	By MS/MS	33,3	29,10	0	59,68
Q92734	Q92734	Protein TFG	TFG	5	5	5	By MS/MS	By MS/MS	By MS/MS	By MS/MS	By MS/MS	By MS/MS	By MS/MS	By MS/MS	By MS/MS	By MS/MS	By MS/MS	By MS/MS	16,5	43,45	0	15,83
P02786	P02786	Transferrin receptor protein 1;Transferrin receptor protein 1, serum form	TFRC	28	28	28	By MS/MS	By MS/MS	By MS/MS	By MS/MS	By MS/MS	By MS/MS	By MS/MS	By MS/MS	By MS/MS	By MS/MS	By MS/MS	By MS/MS	43,3	84,87	0	323,31
P21980	P21980	Protein-glutamine gamma-glutamyltransferase 2	TGM2	6	6	6	By MS/MS	By MS/MS	By MS/MS	By MS/MS	By MS/MS	By MS/MS	By MS/MS	By MS/MS	By MS/MS		By MS/MS		12,2	77,33	0	14,55
Q8NI27	Q8NI27	THO complex subunit 2	THOC2	6	6	6	By MS/MS	By MS/MS	By MS/MS	By MS/MS	By MS/MS	By MS/MS	By MS/MS	By MS/MS	By MS/MS	By MS/MS		By MS/MS	4,3	182,77	0	11,96
Q86W42	Q86W42	THO complex subunit 6 homolog	THOC6	5	5	5	By MS/MS	By MS/MS	By MS/MS	By MS/MS	By MS/MS	By MS/MS	By MS/MS	By MS/MS	By MS/MS	By MS/MS	By MS/MS	By MS/MS	25,8	37,54	0	33,81
P52888	P52888	Thimet oligopeptidase	THOP1	5	5	5	By MS/MS	By MS/MS	By MS/MS	By MS/MS	By MS/MS	By MS/MS	By MS/MS	By MS/MS	By MS/MS	By MS/MS	By MS/MS		11,2	78,84	0	81,80
Q9NXG2	Q9NXG2	THUMP domain-containing protein 1	THUMPD1	5	5	5	By MS/MS	By MS/MS	By MS/MS	By MS/MS	By MS/MS	By MS/MS		By MS/MS	By MS/MS	By MS/MS	By MS/MS	By MS/MS	24,9	39,32	0	25,24
P31483	P31483	Nucleolysin TIA-1 isoform p40	TIA1	4	4	2		By MS/MS		By MS/MS	By MS/MS	By MS/MS	By MS/MS	By MS/MS	By MS/MS	By MS/MS			12,4	42,96	0	15,38
Q9Y5L4	Q9Y5L4	Mitochondrial import inner membrane translocase subunit Tim13	TIMM13	5	5	5	By MS/MS	By MS/MS	By MS/MS	By MS/MS	By MS/MS	By MS/MS	By MS/MS	By MS/MS	By MS/MS	By MS/MS	By MS/MS	By MS/MS	62,1	10,50	0	126,51
O60830	O60830	Mitochondrial import inner membrane translocase subunit Tim17-B	TIMM17B	4	4	3	By MS/MS	By MS/MS	By MS/MS	By MS/MS	By MS/MS	By MS/MS	By MS/MS	By MS/MS	By MS/MS	By MS/MS			37,2	18,27	0	7,01
O14925;Q5SRD1	O14925;Q5SRD1	Mitochondrial import inner membrane translocase subunit Tim23;Putative mitochondrial import inner membrane translocase subunit Tim23B	TIMM23;TIMM23B	5	5	5	By MS/MS	By MS/MS	By MS/MS	By MS/MS	By MS/MS	By MS/MS	By MS/MS	By MS/MS	By MS/MS	By MS/MS	By MS/MS	By MS/MS	27,3	21,94	0	95,94
O43615	O43615	Mitochondrial import inner membrane translocase subunit TIM44	TIMM44	16	16	16	By MS/MS	By MS/MS	By MS/MS	By MS/MS	By MS/MS	By MS/MS	By MS/MS	By MS/MS	By MS/MS	By MS/MS	By MS/MS	By MS/MS	33,4	51,36	0	148,50
Q3ZCQ8	Q3ZCQ8	Mitochondrial import inner membrane translocase subunit TIM50	TIMM50	7	7	7	By MS/MS	By MS/MS	By MS/MS	By MS/MS	By MS/MS	By MS/MS	By MS/MS	By MS/MS	By MS/MS	By MS/MS	By MS/MS	By MS/MS	23,5	39,65	0	79,48
O60220	O60220	Mitochondrial import inner membrane translocase subunit Tim8 A	TIMM8A	4	4	4	By MS/MS	By MS/MS	By MS/MS	By MS/MS	By MS/MS	By MS/MS	By MS/MS					By MS/MS	63,9	11,00	0	18,94
O75663	O75663	TIP41-like protein	TIPRL	7	7	7	By MS/MS	By MS/MS	By MS/MS		By MS/MS	By MS/MS			By MS/MS	By MS/MS			41,9	31,44	0	13,18
P04183	P04183	Thymidine kinase, cytosolic	TK1	11	11	11	By MS/MS	By MS/MS	By MS/MS	By MS/MS	By MS/MS	By MS/MS	By MS/MS	By MS/MS	By MS/MS	By MS/MS		By MS/MS	65,0	25,47	0	42,34
P29401	P29401	Transketolase	TKT	29	29	29	By MS/MS	By MS/MS	By MS/MS	By MS/MS	By MS/MS	By MS/MS	By MS/MS	By MS/MS	By MS/MS	By MS/MS	By MS/MS	By MS/MS	64,7	67,88	0	323,31
Q9Y490	Q9Y490	Talin-1	TLN1	100	100	91	By MS/MS	By MS/MS	By MS/MS	By MS/MS	By MS/MS	By MS/MS	By MS/MS	By MS/MS	By MS/MS	By MS/MS	By MS/MS	By MS/MS	61,3	269,76	0	323,31
Q9Y4G6	Q9Y4G6	Talin-2	TLN2	12	3	3	By matchin	By matchin	By matchin	By MS/MS	By matchin	By matchin	By matchin	By matchin	By MS/MS	By matchin	By matchin	By matchin	5,3	271,61	0,000343	2,65
Q9HD45	Q9HD45	Transmembrane 9 superfamily member 3	TM9SF3	4	4	4				By MS/MS		By MS/MS	By MS/MS		By MS/MS	By MS/MS		By MS/MS	6,8	67,89	0	5,75
P49755	P49755	Transmembrane emp24 domain-containing protein 10	TMED10	12	12	12	By MS/MS	By MS/MS	By MS/MS	By MS/MS	By MS/MS	By MS/MS	By MS/MS	By MS/MS	By MS/MS	By MS/MS	By MS/MS	By MS/MS	42,5	24,98	0	307,84
Q15363	Q15363	Transmembrane emp24 domain-containing protein 2	TMED2	4	4	4	By MS/MS	By MS/MS	By MS/MS	By MS/MS	By MS/MS	By MS/MS	By MS/MS	By MS/MS	By MS/MS	By MS/MS	By MS/MS	By MS/MS	24,9	22,76	0	16,46
Q9Y3B3	Q9Y3B3	Transmembrane emp24 domain-containing protein 7	TMED7	4	4	4	By MS/MS	By MS/MS	By MS/MS	By MS/MS	By MS/MS	By MS/MS	By MS/MS	By MS/MS	By MS/MS				23,2	25,17	0	5,22
Q9BVK6	Q9BVK6	Transmembrane emp24 domain-containing protein 9	TMED9	6	6	5	By MS/MS	By MS/MS	By MS/MS	By MS/MS	By MS/MS	By MS/MS		By MS/MS	By MS/MS	By MS/MS			26,8	27,28	0	323,31
P57088	P57088	Transmembrane protein 33	TMEM33	8	8	8	By MS/MS	By MS/MS	By MS/MS	By MS/MS	By MS/MS	By MS/MS	By MS/MS	By MS/MS	By MS/MS	By MS/MS	By MS/MS	By MS/MS	25,5	27,98	0	56,60
Q9BTV4	Q9BTV4	Transmembrane protein 43	TMEM43	8	8	8	By MS/MS	By MS/MS	By MS/MS	By MS/MS	By MS/MS	By MS/MS	By MS/MS	By MS/MS	By MS/MS	By MS/MS			34,2	44,88	0	45,37
P42166	P42166	Lamina-associated polypeptide 2, isoform alpha;Thymopoietin;Thymopentin	TMPO	9	9	3	By MS/MS	By MS/MS	By MS/MS	By MS/MS	By MS/MS	By MS/MS	By MS/MS	By MS/MS	By MS/MS	By MS/MS	By MS/MS	By MS/MS	23,3	75,49	0	242,88
P42167	P42167	Lamina-associated polypeptide 2, isoforms beta/gamma;Thymopoietin;Thymopentin	TMPO	9	3	3	By matchin	By matchin	By matchin	By matchin	By matchin	By MS/MS	By MS/MS	By matchin	By matchin	By matchin	By MS/MS	By MS/MS	34,8	50,67	0	27,12
Q9H3N1	Q9H3N1	Thioredoxin-related transmembrane protein 1	TMX1	4	4	4	By MS/MS	By MS/MS	By MS/MS	By MS/MS	By MS/MS	By MS/MS	By MS/MS	By MS/MS	By MS/MS	By MS/MS	By MS/MS	By MS/MS	20,0	31,79	0	10,21
Q9Y320	Q9Y320	Thioredoxin-related transmembrane protein 2	TMX2	4	4	4	By MS/MS	By MS/MS	By MS/MS	By MS/MS	By MS/MS	By MS/MS			By MS/MS	By MS/MS			17,6	34,04	0	15,59
Q92973	Q92973	Transportin-1	TNPO1	23	23	21	By MS/MS	By MS/MS	By MS/MS	By MS/MS	By MS/MS	By MS/MS	By MS/MS	By MS/MS	By MS/MS	By MS/MS	By MS/MS	By MS/MS	34,6	102,35	0	289,96
Q9Y5L0	Q9Y5L0	Transportin-3	TNPO3	11	11	11	By MS/MS	By MS/MS	By MS/MS	By MS/MS	By MS/MS	By MS/MS	By MS/MS	By MS/MS	By MS/MS	By MS/MS	By MS/MS	By MS/MS	22,1	104,20	0	104,29

Q9NS69	Q9NS69	Mitochondrial import receptor subunit TOM22 homolog	TOMM22	7	7	7	By MS/MS	By MS/MS	By MS/MS	By MS/MS	By MS/MS	By MS/MS	By MS/MS	By MS/MS	By MS/MS	By MS/MS	By MS/MS	By MS/MS	78,9	15,52	0	235,19
Q15785	Q15785	Mitochondrial import receptor subunit TOM34	TOMM34	9	9	9	By MS/MS	By MS/MS	By MS/MS	By MS/MS	By MS/MS	By MS/MS	By MS/MS	By MS/MS	By MS/MS	By MS/MS	By MS/MS	By MS/MS	43,7	34,56	0	323,31
O96008	O96008	Mitochondrial import receptor subunit TOM40 homolog	TOMM40	14	14	14	By MS/MS	By MS/MS	By MS/MS	By MS/MS	By MS/MS	By MS/MS	By MS/MS	By MS/MS	By MS/MS	By MS/MS			77,3	37,89	0	137,16
O94826	O94826	Mitochondrial import receptor subunit TOM70	TOMM70A	7	7	7	By MS/MS	By MS/MS	By MS/MS	By MS/MS	By MS/MS	By MS/MS	By MS/MS	By MS/MS			By MS/MS	By MS/MS	18,9	67,45	0	40,59
P11387	P11387	DNA topoisomerase 1	TOP1	7	7	7	By MS/MS	By MS/MS	By MS/MS	By MS/MS	By MS/MS	By MS/MS	By MS/MS	By MS/MS	By MS/MS	By MS/MS	By MS/MS	By MS/MS	13,3	90,73	0	11,15
P11388	P11388	DNA topoisomerase 2-alpha	TOP2A	14	14	10		By MS/MS	By MS/MS	By MS/MS	By MS/MS	By MS/MS	By MS/MS	By MS/MS	By MS/MS	By MS/MS	By MS/MS	By MS/MS	12,1	174,38	0	59,16
Q02880	Q02880	DNA topoisomerase 2-beta	TOP2B	6	2	2		By matchin	By matchin	By matching		By MS/MS	By MS/MS	By MS/MS	By MS/MS	By MS/MS	By matchin	By MS/MS	4,7	183,26	0	11,20
O43399	O43399	Tumor protein D54	TPD52L2	11	11	11	By MS/MS	By MS/MS	By MS/MS	By MS/MS	By MS/MS	By MS/MS	By MS/MS	By MS/MS	By MS/MS	By MS/MS	By MS/MS	By MS/MS	66,0	22,24	0	178,85
P60174	P60174	Triosephosphate isomerase	TPI1	23	23	23	By MS/MS	By MS/MS	By MS/MS	By MS/MS	By MS/MS	By MS/MS	By MS/MS	By MS/MS	By MS/MS	By MS/MS	By MS/MS	By MS/MS	78,0	30,79	0	323,31
P06753;P09493	P06753	Tropomyosin alpha-3 chain	TPM3	16	16	8	By MS/MS	By MS/MS	By MS/MS	By MS/MS	By MS/MS	By MS/MS	By MS/MS	By MS/MS	By MS/MS	By MS/MS	By MS/MS	By MS/MS	41,8	32,95	0	133,62
P67936;P07951	P67936	Tropomyosin alpha-4 chain	TPM4	15	7	7	By MS/MS	By MS/MS	By MS/MS	By MS/MS	By MS/MS	By MS/MS	By matchin	By MS/MS	By MS/MS	By MS/MS	By matchin	By MS/MS	50,0	28,52	0	143,40
O14773	O14773	Tripeptidyl-peptidase 1	TPP1	5	5	5	By MS/MS	By MS/MS	By MS/MS	By MS/MS	By MS/MS	By MS/MS		By MS/MS					14,7	61,25	0	39,03
P29144	P29144	Tripeptidyl-peptidase 2	TPP2	23	23	23	By MS/MS	By MS/MS	By MS/MS	By MS/MS	By MS/MS	By MS/MS	By MS/MS	By MS/MS	By MS/MS	By MS/MS	By MS/MS	By MS/MS	25,4	138,35	0	276,94
P12270	P12270	Nucleoprotein TPR	TPR	48	48	48	By MS/MS	By MS/MS	By MS/MS	By MS/MS	By MS/MS	By MS/MS	By MS/MS	By MS/MS	By MS/MS	By MS/MS	By MS/MS	By MS/MS	24,8	267,29	0	323,31
P13693;Q56UQ5	P13693	Translationally-controlled tumor protein	TPT1	6	6	6	By MS/MS	By MS/MS	By MS/MS	By MS/MS	By MS/MS	By MS/MS	By MS/MS	By MS/MS	By MS/MS	By MS/MS	By MS/MS	By MS/MS	35,5	19,60	0	63,58
P62995	P62995	Transformer-2 protein homolog beta	TRA2B	6	6	6	By MS/MS	By MS/MS	By MS/MS	By MS/MS	By MS/MS	By MS/MS	By MS/MS	By MS/MS	By MS/MS	By MS/MS			29,5	33,67	0	57,68
Q12931	Q12931	Heat shock protein 75 kDa, mitochondrial	TRAP1	24	24	24	By MS/MS	By MS/MS	By MS/MS	By MS/MS	By MS/MS	By MS/MS	By MS/MS	By MS/MS	By MS/MS	By MS/MS	By MS/MS	By MS/MS	49,7	80,11	0	323,31
Q9Y5R8	Q9Y5R8	Trafficking protein particle complex subunit 1	TRAPPC1	4	4	4	By MS/MS	By MS/MS		By MS/MS		By MS/MS				By MS/MS		By MS/MS	33,8	16,83	0	20,61
O43617	O43617	Trafficking protein particle complex subunit 3	TRAPPC3	6	6	6	By MS/MS	By MS/MS	By MS/MS	By MS/MS	By MS/MS	By MS/MS	By MS/MS	By MS/MS	By MS/MS	By MS/MS		By MS/MS	38,3	20,27	0	17,25
Q9Y296	Q9Y296	Trafficking protein particle complex subunit 4	TRAPPC4	6	6	6		By MS/MS		By MS/MS	By MS/MS	By MS/MS		By MS/MS	By MS/MS	By MS/MS	By MS/MS	By MS/MS	30,6	24,34	0	11,96
Q13263	Q13263	Transcription intermediary factor 1-beta	TRIM28	26	26	26	By MS/MS	By MS/MS	By MS/MS	By MS/MS	By MS/MS	By MS/MS	By MS/MS	By MS/MS	By MS/MS	By MS/MS	By MS/MS	By MS/MS	49,7	88,55	0	323,31
Q15645	Q15645	Pachytene checkpoint protein 2 homolog	TRIP13	10	10	10	By MS/MS	By MS/MS	By MS/MS	By MS/MS	By MS/MS	By MS/MS					By MS/MS	By MS/MS	31,0	48,55	0	29,22
Q7L0Y3	Q7L0Y3	Mitochondrial ribonuclease P protein 1	TRMT10C	11	11	11	By MS/MS	By MS/MS	By MS/MS	By MS/MS	By MS/MS	By MS/MS	By MS/MS	By MS/MS	By MS/MS	By MS/MS			36,0	47,35	0	28,23
Q9UI30	Q9UI30	Multifunctional methyltransferase subunit TRM112-like protein	TRMT112	6	6	6	By MS/MS	By MS/MS	By MS/MS	By MS/MS	By MS/MS	By MS/MS	By MS/MS	By MS/MS	By MS/MS	By MS/MS	By MS/MS	By MS/MS	55,2	14,20	0	187,55
P10155	P10155	60 kDa SS-A/Ro ribonucleoprotein	TROVE2	9	9	9	By MS/MS	By MS/MS	By MS/MS	By MS/MS	By MS/MS	By MS/MS		By MS/MS			By MS/MS	By MS/MS	25,5	60,67	0	57,96
P43897	P43897	Elongation factor Ts, mitochondrial	TSFM	5	5	5	By MS/MS	By MS/MS	By MS/MS	By MS/MS	By MS/MS	By MS/MS	By MS/MS	By MS/MS	By MS/MS	By MS/MS	By MS/MS	By MS/MS	27,7	35,39	0	41,90
Q15631	Q15631	Translin	TSN	14	14	14	By MS/MS	By MS/MS	By MS/MS	By MS/MS	By MS/MS	By MS/MS	By MS/MS	By MS/MS	By MS/MS	By MS/MS	By MS/MS	By MS/MS	50,9	26,18	0	66,79
Q99598	Q99598	Translin-associated protein X	TSNAX	12	12	12	By MS/MS	By MS/MS	By MS/MS	By MS/MS	By MS/MS	By MS/MS	By MS/MS	By MS/MS	By MS/MS	By MS/MS	By MS/MS	By MS/MS	52,4	33,11	0	143,04
Q2NL82	Q2NL82	Pre-rRNA-processing protein TSR1 homolog	TSR1	9	9	9	By MS/MS	By MS/MS	By MS/MS	By MS/MS	By MS/MS	By MS/MS	By MS/MS	By MS/MS	By MS/MS	By MS/MS	By MS/MS	By MS/MS	16,3	91,81	0	58,69
Q16762	Q16762	Thiosulfate sulfurtransferase	TST	5	5	5	By MS/MS		By MS/MS		By MS/MS	By MS/MS	By MS/MS		By MS/MS				28,6	33,43	0	10,73
Q99614	Q99614	Tetratricopeptide repeat protein 1	TTC1	6	6	6	By MS/MS				By MS/MS	By MS/MS			By MS/MS	By MS/MS			29,1	33,53	0	11,53
Q6P3X3	Q6P3X3	Tetratricopeptide repeat protein 27	TTC27	5	5	5	By MS/MS	By MS/MS	By MS/MS	By MS/MS	By MS/MS	By MS/MS		By MS/MS	By MS/MS	By MS/MS	By MS/MS		9,3	96,63	0	26,39
O95801	O95801	Tetratricopeptide repeat protein 4	TTC4	4	4	4	By MS/MS	By MS/MS	By MS/MS				By MS/MS	By MS/MS	By MS/MS	By MS/MS			13,7	44,68	0	10,07
Q14166	Q14166	Tubulin--tyrosine ligase-like protein 12	TTLL12	17	17	17	By MS/MS	By MS/MS	By MS/MS	By MS/MS	By MS/MS	By MS/MS	By MS/MS	By MS/MS	By MS/MS	By MS/MS	By MS/MS	By MS/MS	39,4	74,40	0	137,70
Q8WZ42	Q8WZ42	Titin	TTN	10	10	10	By MS/MS	By MS/MS	By MS/MS	By MS/MS	By MS/MS	By MS/MS	By MS/MS		By MS/MS				0,4	3816,00	0	5,92
Q9BQE3	Q9BQE3	Tubulin alpha-1C chain	TUBA1C	31	4	4	By MS/MS	By MS/MS	By MS/MS	By MS/MS	By MS/MS	By MS/MS	By MS/MS	By MS/MS	By MS/MS	By MS/MS	By MS/MS	By MS/MS	72,2	49,90	0	323,31
P68366	P68366	Tubulin alpha-4A chain	TUBA4A	25	4	4	By MS/MS	By MS/MS	By MS/MS	By MS/MS	By MS/MS	By MS/MS	By MS/MS	By MS/MS	By MS/MS	By MS/MS	By MS/MS	By MS/MS	65,4	49,92	0	10,60
P07437;A6NNZ2;Q9H4B7	P07437	Tubulin beta chain	TUBB	27	6	5	By MS/MS	By MS/MS	By MS/MS	By MS/MS	By MS/MS	By MS/MS	By MS/MS	By MS/MS	By MS/MS	By MS/MS	By MS/MS	By MS/MS	64,0	49,67	0	323,31
Q13885;Q9BVA1	Q13885;Q9BVA1	Tubulin beta-2A chain;Tubulin beta-2B chain	TUBB2A;TUBB2B	26	6	5	By MS/MS	By MS/MS	By MS/MS	By MS/MS	By MS/MS	By MS/MS	By MS/MS	By MS/MS	By matchin	By MS/MS	By MS/MS	By MS/MS	63,6	49,91	0	63,90
Q13509	Q13509	Tubulin beta-3 chain	TUBB3	19	4	4	By MS/MS	By MS/MS	By MS/MS	By MS/MS	By MS/MS	By MS/MS	By MS/MS	By MS/MS	By MS/MS	By MS/MS	By MS/MS	By MS/MS	45,8	50,43	0	158,21
Q9BUF5	Q9BUF5	Tubulin beta-6 chain	TUBB6	24	16	12	By MS/MS	By MS/MS	By MS/MS	By MS/MS	By MS/MS	By MS/MS	By MS/MS	By MS/MS	By matchin	By MS/MS	By MS/MS	By MS/MS	63,5	49,86	0	323,31
Q3ZCM7	Q3ZCM7	Tubulin beta-8 chain	TUBB8	11	2	2	By MS/MS	By matchin	By MS/MS	By matchin	By MS/MS	By MS/MS	By matchin	By MS/MS	By matchin	By matchin	By MS/MS	By MS/MS	24,5	49,78	0	24,11
P49411	P49411	Elongation factor Tu, mitochondrial	TUFM	27	27	27	By MS/MS	By MS/MS	By MS/MS	By MS/MS	By MS/MS	By MS/MS	By MS/MS	By MS/MS	By MS/MS	By MS/MS	By MS/MS	By MS/MS	66,4	49,54	0	303,15
Q6IBS0	Q6IBS0	Twinfilin-2	TWF2	5	5	5	By MS/MS	By MS/MS	By MS/MS	By MS/MS	By MS/MS	By MS/MS	By MS/MS	By MS/MS		By MS/MS			26,6	39,55	0	48,93
P10599	P10599	Thioredoxin	TXN	11	11	11	By MS/MS	By MS/MS	By MS/MS	By MS/MS	By MS/MS	By MS/MS	By MS/MS	By MS/MS	By MS/MS	By MS/MS	By MS/MS	By MS/MS	66,7	11,74	0	151,47
O95881	O95881	Thioredoxin domain-containing protein 12	TXNDC12	4	4	4	By MS/MS	By MS/MS	By MS/MS	By MS/MS	By MS/MS	By MS/MS	By MS/MS	By MS/MS	By MS/MS	By MS/MS		By MS/MS	30,8	19,21	0	13,72
Q9BRA2	Q9BRA2	Thioredoxin domain-containing protein 17	TXNDC17	5	5	5	By MS/MS	By MS/MS	By MS/MS	By MS/MS	By MS/MS	By MS/MS	By MS/MS	By MS/MS	By MS/MS	By MS/MS	By MS/MS	By MS/MS	53,7	13,94	0	32,28
Q8NBS9	Q8NBS9	Thioredoxin domain-containing protein 5	TXNDC5	8	8	8	By MS/MS	By MS/MS	By MS/MS	By MS/MS	By MS/MS	By MS/MS	By MS/MS	By MS/MS			By MS/MS	By MS/MS	22,2	47,63	0	12,15
O43396	O43396	Thioredoxin-like protein 1	TXNL1	12	12	12	By MS/MS	By MS/MS	By MS/MS	By MS/MS	By MS/MS	By MS/MS	By MS/MS	By MS/MS	By MS/MS	By MS/MS	By MS/MS	By MS/MS	58,8	32,25	0	118,62
Q16881	Q16881	Thioredoxin reductase 1, cytoplasmic	TXNRD1	17	17	16	By MS/MS	By MS/MS	By MS/MS	By MS/MS	By MS/MS	By MS/MS	By MS/MS	By MS/MS			By MS/MS	By MS/MS	43,8	70,91	0	323,31
Q9NNW7	Q9NNW7	Thioredoxin reductase 2, mitochondrial	TXNRD2	7	6	6	By matchin	By MS/MS	By MS/MS	By MS/MS	By MS/MS	By MS/MS	By matchin	By matching			By MS/MS	By MS/MS	25,4	56,51	0	37,21
P04818	P04818	Thymidylate synthase	TYMS	10	10	10	By MS/MS	By MS/MS	By MS/MS	By MS/MS	By MS/MS	By MS/MS	By MS/MS	By MS/MS	By MS/MS	By MS/MS	By MS/MS	By MS/MS	41,5	35,72	0	323,31
P0DN76;Q01081;Q8WU68	P0DN76;Q01081;Q8WU68	Splicing factor U2AF 35 kDa subunit;Splicing factor U2AF 26 kDa subunit	U2AF1;U2AF1L4	6	6	6	By MS/MS	By MS/MS	By MS/MS	By MS/MS	By MS/MS	By MS/MS	By MS/MS	By MS/MS	By MS/MS	By MS/MS		By MS/MS	32,1	27,87	0	44,98
P26368	P26368	Splicing factor U2AF 65 kDa subunit	U2AF2	12	12	12	By MS/MS	By MS/MS	By MS/MS	By MS/MS	By MS/MS	By MS/MS	By MS/MS	By MS/MS	By MS/MS	By MS/MS	By MS/MS	By MS/MS	44,8	53,50	0	144,83
O15042	O15042	U2 snRNP-associated SURP motif-containing protein	U2SURP	9	9	9		By MS/MS	By MS/MS	By MS/MS	By MS/MS		By MS/MS	By MS/MS	By MS/MS	By MS/MS	By MS/MS	By MS/MS	12,0	118,29	0	15,79
Q16222;Q3KQV9	Q16222	UDP-N-acetylhexosamine pyrophosphorylase;UDP-N-acetylgalactosamine pyrophosphorylase;UDP-N-acetylglucosamine pyrophosphorylase	UAP1	10	10	10	By MS/MS	By MS/MS	By MS/MS	By MS/MS	By MS/MS	By MS/MS						By MS/MS	23,0	58,77	0	22,12
P22314	P22314	Ubiquitin-like modifier-activating enzyme 1	UBA1	37	37	37	By MS/MS	By MS/MS	By MS/MS	By MS/MS	By MS/MS	By MS/MS	By MS/MS	By MS/MS	By MS/MS	By MS/MS	By MS/MS	By MS/MS	49,2	117,85	0	323,31
Q9UBT2	Q9UBT2	SUMO-activating enzyme subunit 2	UBA2	14	14	14	By MS/MS	By MS/MS	By MS/MS	By MS/MS	By MS/MS	By MS/MS	By MS/MS	By MS/MS	By MS/MS	By MS/MS	By MS/MS	By MS/MS	33,6	71,22	0	59,00
Q8TBC4	Q8TBC4	NEDD8-activating enzyme E1 catalytic subunit	UBA3	7	7	7	By MS/MS	By MS/MS	By MS/MS	By MS/MS	By MS/MS	By MS/MS					By MS/MS	By MS/MS	27,9	51,85	0	55,93
A0AVT1	A0AVT1	Ubiquitin-like modifier-activating enzyme 6	UBA6	25	25	25	By MS/MS	By MS/MS	By MS/MS	By MS/MS	By MS/MS	By MS/MS	By MS/MS	By MS/MS	By MS/MS	By MS/MS	By MS/MS	By MS/MS	32,9	117,97	0	214,43
Q9BSL1	Q9BSL1	Ubiquitin-associated domain-containing protein 1	UBAC1	9	9	9	By MS/MS	By MS/MS	By MS/MS	By MS/MS	By MS/MS	By MS/MS	By MS/MS	By MS/MS	By MS/MS	By MS/MS	By MS/MS	By MS/MS	33,6	45,34	0	93,43
Q14157	Q14157	Ubiquitin-associated protein 2-like	UBAP2L	7	7	7	By MS/MS	By MS/MS	By MS/MS	By MS/MS	By MS/MS	By MS/MS						By MS/MS	11,2	114,53	0	229,74
P61077;P62837	P61077;P62837	Ubiquitin-conjugating enzyme E2 D3;Ubiquitin-conjugating enzyme E2 D2	UBE2D3;UBE2D2	4	4	3	By MS/MS	By MS/MS	By MS/MS	By MS/MS	By MS/MS		By MS/MS	By MS/MS			By MS/MS	By MS/MS	42,9	16,69	0	16,84
P62253	P62253	Ubiquitin-conjugating enzyme E2 G1;Ubiquitin-conjugating enzyme E2 G1, N-terminally processed	UBE2G1	4	4	4	By MS/MS	By MS/MS	By MS/MS	By MS/MS	By MS/MS		By MS/MS	By MS/MS	By MS/MS	By MS/MS		By MS/MS	21,2	19,51	0	109,39
P62256	P62256	Ubiquitin-conjugating enzyme E2 H	UBE2H	4	4	4	By MS/MS	By MS/MS		By MS/MS	By MS/MS	By MS/MS	By MS/MS	By MS/MS	By MS/MS			By MS/MS	23,0	20,66	0	16,17
P63279	P63279	SUMO-conjugating enzyme UBC9	UBE2I	8	8	8	By MS/MS	By MS/MS	By MS/MS	By MS/MS	By MS/MS	By MS/MS	By MS/MS	By MS/MS	By MS/MS	By MS/MS	By MS/MS	By MS/MS	39,2	18,01	0	43,74
P61086	P61086	Ubiquitin-conjugating enzyme E2 K	UBE2K	12	12	12	By MS/MS	By MS/MS	By MS/MS	By MS/MS	By MS/MS	By MS/MS	By MS/MS	By MS/MS	By MS/MS	By MS/MS	By MS/MS	By MS/MS	63,0	22,41	0	105,10
P68036	P68036	Ubiquitin-conjugating enzyme E2 L3	UBE2L3	14	14	14	By MS/MS	By MS/MS	By MS/MS	By MS/MS	By MS/MS	By MS/MS	By MS/MS	By MS/MS	By MS/MS	By MS/MS	By MS/MS	By MS/MS	76,6	17,86	0	230,24

P61081	P61081	NEDD8-conjugating enzyme Ubc12	UBE2M	6	6	6	By MS/MS	By MS/MS	By MS/MS	By MS/MS	By MS/MS	By MS/MS	By MS/MS	By MS/MS	By MS/MS	By MS/MS	By MS/MS	By MS/MS	33,3	20,90	0	93,79
P61088;Q5JXB2	P61088;Q5JXB2	Ubiquitin-conjugating enzyme E2 N;Putative ubiquitin-conjugating enzyme E2 N-like	UBE2N;UBE2NL	12	12	12	By MS/MS	By MS/MS	By MS/MS	By MS/MS	By MS/MS	By MS/MS	By MS/MS	By MS/MS	By MS/MS	By MS/MS	By MS/MS	By MS/MS	65,8	17,14	0	227,95
Q9C0C9	Q9C0C9	E2/E3 hybrid ubiquitin-protein ligase UBE2O	UBE2O	6	6	6	By MS/MS	By MS/MS	By MS/MS	By MS/MS	By MS/MS	By MS/MS					By MS/MS	By MS/MS	7,9	141,29	0	9,66
Q16763	Q16763	Ubiquitin-conjugating enzyme E2 S	UBE2S	7	7	7	By MS/MS	By MS/MS	By MS/MS	By MS/MS	By MS/MS	By MS/MS	By MS/MS		By MS/MS	By MS/MS	By MS/MS	By MS/MS	46,4	23,85	0	22,34
Q9NPD8	Q9NPD8	Ubiquitin-conjugating enzyme E2 T	UBE2T	6	6	6	By MS/MS	By MS/MS	By MS/MS	By MS/MS	By MS/MS	By MS/MS					By MS/MS	By MS/MS	30,5	22,52	0	7,17
Q13404	Q13404	Ubiquitin-conjugating enzyme E2 variant 1	UBE2V1	12	6	6	By MS/MS	By MS/MS	By MS/MS	By MS/MS	By MS/MS	By MS/MS	By MS/MS	By MS/MS	By MS/MS	By MS/MS	By MS/MS	By MS/MS	78,9	16,50	0	16,66
Q15819	Q15819	Ubiquitin-conjugating enzyme E2 variant 2	UBE2V2	12	12	6	By MS/MS	By MS/MS	By MS/MS	By MS/MS	By MS/MS	By MS/MS	By MS/MS	By MS/MS	By MS/MS	By MS/MS	By MS/MS	By MS/MS	78,6	16,36	0	140,15
Q14139	Q14139	Ubiquitin conjugation factor E4 A	UBE4A	10	10	10	By MS/MS	By MS/MS	By MS/MS	By MS/MS	By MS/MS	By MS/MS						By MS/MS	14,3	122,56	0	25,11
O95155	O95155	Ubiquitin conjugation factor E4 B	UBE4B	6	6	6	By MS/MS	By MS/MS	By MS/MS	By MS/MS	By MS/MS	By MS/MS						By MS/MS	7,6	146,18	0	28,84
P11441	P11441	Ubiquitin-like protein 4A	UBL4A	7	7	7	By MS/MS	By MS/MS	By MS/MS	By MS/MS	By MS/MS	By MS/MS	By MS/MS	By MS/MS		By MS/MS	By MS/MS	By MS/MS	51,6	17,78	0	21,87
Q8WVY7	Q8WVY7	Ubiquitin-like domain-containing CTD phosphatase 1	UBLCP1	7	7	7	By MS/MS	By MS/MS	By MS/MS		By MS/MS	By MS/MS	By MS/MS						22,6	36,80	0	8,40
Q9NRR5	Q9NRR5	Ubiquilin-4	UBQLN4	4	4	2	By MS/MS	By MS/MS	By MS/MS	By MS/MS	By MS/MS	By MS/MS	By MS/MS	By MS/MS	By MS/MS			By MS/MS	12,1	63,85	0	6,57
Q5T4S7	Q5T4S7	E3 ubiquitin-protein ligase UBR4	UBR4	54	54	54	By MS/MS	By MS/MS	By MS/MS	By MS/MS	By MS/MS	By MS/MS	By MS/MS	By MS/MS	By MS/MS	By MS/MS	By MS/MS	By MS/MS	18,2	573,83	0	313,05
Q04323	Q04323	UBX domain-containing protein 1	UBXN1	5	5	5	By MS/MS	By MS/MS	By MS/MS	By MS/MS	By MS/MS	By MS/MS			By MS/MS	By MS/MS			27,9	33,33	0	22,06
P15374	P15374	Ubiquitin carboxyl-terminal hydrolase isozyme L3	UCHL3	12	12	12	By MS/MS	By MS/MS	By MS/MS	By MS/MS	By MS/MS	By MS/MS	By MS/MS	By MS/MS	By MS/MS	By MS/MS	By MS/MS	By MS/MS	67,8	26,18	0	88,11
Q9Y5K5	Q9Y5K5	Ubiquitin carboxyl-terminal hydrolase isozyme L5	UCHL5	12	12	12	By MS/MS	By MS/MS	By MS/MS	By MS/MS	By MS/MS	By MS/MS	By MS/MS	By MS/MS	By MS/MS	By MS/MS	By MS/MS	By MS/MS	46,5	37,61	0	56,58
Q9BZX2;Q9HA47	Q9BZX2	Uridine-cytidine kinase 2	UCK2	5	5	5	By MS/MS	By MS/MS			By MS/MS		By MS/MS	By MS/MS					21,8	29,30	0	7,75
Q92890	Q92890	Ubiquitin fusion degradation protein 1 homolog	UFD1L	6	6	6	By MS/MS	By MS/MS	By MS/MS	By MS/MS	By MS/MS	By MS/MS	By MS/MS	By MS/MS	By MS/MS	By MS/MS	By MS/MS		30,3	34,50	0	8,46
O94874	O94874	E3 UFM1-protein ligase 1	UFL1	7	7	7	By MS/MS	By MS/MS	By MS/MS	By MS/MS	By MS/MS	By MS/MS	By MS/MS	By MS/MS					13,6	89,59	0	65,89
O60701	O60701	UDP-glucose 6-dehydrogenase	UGDH	5	5	5	By MS/MS	By MS/MS	By MS/MS	By MS/MS	By MS/MS	By MS/MS					By MS/MS		18,0	55,02	0	15,05
Q9NYU2	Q9NYU2	UDP-glucose:glycoprotein glucosyltransferase 1	UGGT1	22	22	22	By MS/MS	By MS/MS	By MS/MS	By MS/MS	By MS/MS	By MS/MS	By MS/MS	By MS/MS	By MS/MS	By MS/MS	By MS/MS	By MS/MS	23,3	177,19	0	305,13
Q16851	Q16851	UTP--glucose-1-phosphate uridylyltransferase	UGP2	6	6	6	By MS/MS	By MS/MS	By MS/MS	By MS/MS	By MS/MS	By MS/MS					By MS/MS	By MS/MS	17,3	56,94	0	127,31
P11172	P11172	Uridine 5-monophosphate synthase;Orotate phosphoribosyltransferase;Orotidine 5-phosphate decarboxylase	UMPS	4	4	4	By MS/MS	By MS/MS	By MS/MS		By MS/MS	By MS/MS					By MS/MS	By MS/MS	12,1	52,22	0	21,97
Q9H3U1	Q9H3U1	Protein unc-45 homolog A	UNC45A	4	4	4	By MS/MS	By MS/MS	By MS/MS	By MS/MS		By MS/MS						By MS/MS	7,5	103,08	0	37,41
Q92900	Q92900	Regulator of nonsense transcripts 1	UPF1	28	28	28	By MS/MS	By MS/MS	By MS/MS	By MS/MS	By MS/MS	By MS/MS	By MS/MS	By MS/MS	By MS/MS	By MS/MS	By MS/MS	By MS/MS	32,6	124,34	0	256,83
Q9NVA1	Q9NVA1	Ubiquinol-cytochrome-c reductase complex assembly factor 1	UQCC1	5	5	5	By MS/MS	By MS/MS	By MS/MS	By MS/MS		By MS/MS		By MS/MS		By MS/MS		By MS/MS	14,0	34,60	0	9,89
Q9BRT2	Q9BRT2	Ubiquinol-cytochrome-c reductase complex assembly factor 2	UQCC2	5	5	5	By MS/MS	By MS/MS	By MS/MS	By MS/MS	By MS/MS	By MS/MS		By MS/MS	By MS/MS		By MS/MS		47,6	14,88	0	10,11
P14927	P14927	Cytochrome b-c1 complex subunit 7	UQCRB	9	9	9	By MS/MS	By MS/MS	By MS/MS	By MS/MS	By MS/MS	By MS/MS	By MS/MS	By MS/MS	By MS/MS	By MS/MS	By MS/MS	By MS/MS	59,5	13,53	0	293,53
P31930	P31930	Cytochrome b-c1 complex subunit 1, mitochondrial	UQCRC1	15	15	15	By MS/MS	By MS/MS	By MS/MS	By MS/MS	By MS/MS	By MS/MS	By MS/MS	By MS/MS	By MS/MS	By MS/MS	By MS/MS	By MS/MS	45,8	52,65	0	323,31
P22695	P22695	Cytochrome b-c1 complex subunit 2, mitochondrial	UQCRC2	17	17	17	By MS/MS	By MS/MS	By MS/MS	By MS/MS	By MS/MS	By MS/MS	By MS/MS	By MS/MS	By MS/MS	By MS/MS	By MS/MS	By MS/MS	47,7	48,44	0	323,31
P47985;P0C7P4	P47985;P0C7P4	Cytochrome b-c1 complex subunit Rieske, mitochondrial;Cytochrome b-c1 complex subunit 11;Putative cytochrome b-c1 complex subunit Rieske-like protein 1	UQCRFS1;UQCRFS1P1	9	9	9	By MS/MS	By MS/MS	By MS/MS	By MS/MS	By MS/MS	By MS/MS	By MS/MS	By MS/MS	By MS/MS	By MS/MS	By MS/MS	By MS/MS	41,2	29,67	0	213,20
O14949	O14949	Cytochrome b-c1 complex subunit 8	UQCRQ	4	4	4	By MS/MS	By MS/MS			By MS/MS		By MS/MS	By MS/MS	By MS/MS	By MS/MS	By MS/MS	By MS/MS	36,6	9,91	0	11,83
O60287	O60287	Nucleolar pre-ribosomal-associated protein 1	URB1	5	5	5	By MS/MS	By MS/MS	By MS/MS	By MS/MS		By MS/MS	By MS/MS	By MS/MS					3,7	254,39	0	8,34
P06132	P06132	Uroporphyrinogen decarboxylase	UROD	17	17	17	By MS/MS	By MS/MS	By MS/MS	By MS/MS	By MS/MS	By MS/MS	By MS/MS	By MS/MS	By MS/MS	By MS/MS			75,7	40,79	0	175,50
Q96IX5	Q96IX5	Up-regulated during skeletal muscle growth protein 5	USMG5	4	4	4	By MS/MS	By MS/MS	By MS/MS	By MS/MS	By MS/MS	By MS/MS	By MS/MS	By MS/MS	By MS/MS	By MS/MS	By MS/MS	By MS/MS	44,8	6,46	0	323,31
O60763	O60763	General vesicular transport factor p115	USO1	12	12	12	By MS/MS	By MS/MS	By MS/MS	By MS/MS	By MS/MS	By MS/MS	By MS/MS	By MS/MS	By MS/MS	By MS/MS	By MS/MS	By MS/MS	17,6	107,89	0	147,63
Q14694	Q14694	Ubiquitin carboxyl-terminal hydrolase 10	USP10	13	13	13	By MS/MS	By MS/MS	By MS/MS	By MS/MS	By MS/MS	By MS/MS		By MS/MS		By MS/MS	By MS/MS	By MS/MS	24,8	87,13	0	33,13
P54578	P54578	Ubiquitin carboxyl-terminal hydrolase 14	USP14	20	20	20	By MS/MS	By MS/MS	By MS/MS	By MS/MS	By MS/MS	By MS/MS	By MS/MS	By MS/MS			By MS/MS	By MS/MS	51,2	56,07	0	260,42
Q9Y4E8	Q9Y4E8	Ubiquitin carboxyl-terminal hydrolase 15	USP15	27	27	26	By MS/MS	By MS/MS	By MS/MS	By MS/MS	By MS/MS	By MS/MS	By MS/MS	By MS/MS	By MS/MS	By MS/MS	By MS/MS	By MS/MS	34,8	112,42	0	195,61
Q53GS9	Q53GS9	U4/U6.U5 tri-snRNP-associated protein 2	USP39	6	6	6	By MS/MS	By MS/MS	By MS/MS	By MS/MS	By MS/MS	By MS/MS	By MS/MS	By MS/MS			By MS/MS	By MS/MS	19,1	65,38	0	25,04
P45974	P45974	Ubiquitin carboxyl-terminal hydrolase 5	USP5	10	10	9	By MS/MS	By MS/MS	By MS/MS	By MS/MS	By MS/MS	By MS/MS	By MS/MS	By MS/MS	By MS/MS	By MS/MS	By MS/MS	By MS/MS	18,1	95,79	0	143,47
Q93009	Q93009	Ubiquitin carboxyl-terminal hydrolase 7	USP7	20	20	20	By MS/MS	By MS/MS	By MS/MS	By MS/MS	By MS/MS	By MS/MS	By MS/MS	By MS/MS	By MS/MS	By MS/MS	By MS/MS	By MS/MS	26,9	128,30	0	191,58
P63027;P23763	P63027	Vesicle-associated membrane protein 2	VAMP2	5	5	2	By MS/MS	By MS/MS	By MS/MS	By MS/MS	By MS/MS	By MS/MS	By MS/MS	By MS/MS	By MS/MS	By MS/MS	By MS/MS	By MS/MS	54,3	12,66	0	67,99
P51809	P51809	Vesicle-associated membrane protein 7	VAMP7	7	7	7	By MS/MS	By MS/MS	By MS/MS	By MS/MS	By MS/MS	By MS/MS	By MS/MS	By MS/MS	By MS/MS	By MS/MS		By MS/MS	39,5	24,94	0	23,89
Q9P0L0	Q9P0L0	Vesicle-associated membrane protein-associated protein A	VAPA	9	9	8	By MS/MS	By MS/MS	By MS/MS	By MS/MS	By MS/MS	By MS/MS	By MS/MS	By MS/MS	By MS/MS	By MS/MS	By MS/MS	By MS/MS	36,5	27,89	0	37,20
O95292	O95292	Vesicle-associated membrane protein-associated protein B/C	VAPB	5	4	4	By MS/MS	By MS/MS	By MS/MS	By MS/MS	By MS/MS	By MS/MS	By MS/MS	By MS/MS	By MS/MS	By MS/MS	By matchin	By MS/MS	23,5	27,23	0	17,91
P26640	P26640	Valine--tRNA ligase	VARS	53	53	53	By MS/MS	By MS/MS	By MS/MS	By MS/MS	By MS/MS	By MS/MS	By MS/MS	By MS/MS	By MS/MS	By MS/MS	By MS/MS	By MS/MS	48,7	140,47	0	323,31
P50552	P50552	Vasodilator-stimulated phosphoprotein	VASP	6	6	6	By MS/MS	By MS/MS	By MS/MS	By MS/MS	By MS/MS	By MS/MS					By MS/MS	By MS/MS	22,1	39,83	0	21,90
Q99536	Q99536	Synaptic vesicle membrane protein VAT-1 homolog	VAT1	18	18	18	By MS/MS	By MS/MS	By MS/MS	By MS/MS	By MS/MS	By MS/MS	By MS/MS	By MS/MS	By MS/MS	By MS/MS	By MS/MS	By MS/MS	68,4	41,92	0	103,73
P61758	P61758	Prefoldin subunit 3	VBP1	10	10	10	By MS/MS	By MS/MS	By MS/MS	By MS/MS	By MS/MS	By MS/MS	By MS/MS	By MS/MS	By MS/MS	By MS/MS	By MS/MS	By MS/MS	47,7	22,66	0	20,13
P18206	P18206	Vinculin	VCL	60	60	60	By MS/MS	By MS/MS	By MS/MS	By MS/MS	By MS/MS	By MS/MS	By MS/MS	By MS/MS	By MS/MS	By MS/MS	By MS/MS	By MS/MS	58,4	123,80	0	323,31
P55072	P55072	Transitional endoplasmic reticulum ATPase	VCP	47	47	47	By MS/MS	By MS/MS	By MS/MS	By MS/MS	By MS/MS	By MS/MS	By MS/MS	By MS/MS	By MS/MS	By MS/MS	By MS/MS	By MS/MS	68,1	89,32	0	323,31
Q96JH7	Q96JH7	Deubiquitinating protein VCIP135	VCPIP1	4	4	4	By MS/MS	By MS/MS	By MS/MS	By MS/MS	By MS/MS	By MS/MS					By MS/MS		4,9	134,32	0	41,06
P21796	P21796	Voltage-dependent anion-selective channel protein 1	VDAC1	20	20	20	By MS/MS	By MS/MS	By MS/MS	By MS/MS	By MS/MS	By MS/MS	By MS/MS	By MS/MS	By MS/MS	By MS/MS	By MS/MS	By MS/MS	84,1	30,77	0	323,31
P45880	P45880	Voltage-dependent anion-selective channel protein 2	VDAC2	17	17	17	By MS/MS	By MS/MS	By MS/MS	By MS/MS	By MS/MS	By MS/MS	By MS/MS	By MS/MS	By MS/MS	By MS/MS	By MS/MS	By MS/MS	61,6	31,57	0	323,31
Q9Y277	Q9Y277	Voltage-dependent anion-selective channel protein 3	VDAC3	15	15	15	By MS/MS	By MS/MS	By MS/MS	By MS/MS	By MS/MS	By MS/MS	By MS/MS	By MS/MS	By MS/MS	By MS/MS	By MS/MS	By MS/MS	58,3	30,66	0	226,98
P08670;P17661;P41219	P08670	Vimentin	VIM	28	27	27	By MS/MS	By MS/MS	By MS/MS	By MS/MS	By MS/MS	By MS/MS	By MS/MS	By MS/MS	By MS/MS	By MS/MS	By MS/MS	By MS/MS	66,1	53,65	0	323,31
O75436	O75436	Vacuolar protein sorting-associated protein 26A	VPS26A	7	7	6	By MS/MS	By MS/MS	By MS/MS	By MS/MS	By MS/MS	By MS/MS	By MS/MS	By MS/MS	By MS/MS	By MS/MS			30,0	38,17	0	37,74
Q9UK41	Q9UK41	Vacuolar protein sorting-associated protein 28 homolog	VPS28	4	4	4	By MS/MS	By MS/MS		By MS/MS	By MS/MS	By MS/MS	By MS/MS	By MS/MS	By MS/MS	By MS/MS	By MS/MS	By MS/MS	31,2	25,43	0	28,53
Q9UBQ0	Q9UBQ0	Vacuolar protein sorting-associated protein 29	VPS29	7	7	7	By MS/MS	By MS/MS	By MS/MS	By MS/MS	By MS/MS	By MS/MS	By MS/MS	By MS/MS	By MS/MS	By MS/MS	By MS/MS	By MS/MS	36,8	20,51	0	33,86
Q96QK1	Q96QK1	Vacuolar protein sorting-associated protein 35	VPS35	24	24	24	By MS/MS	By MS/MS	By MS/MS	By MS/MS	By MS/MS	By MS/MS	By MS/MS	By MS/MS	By MS/MS	By MS/MS	By MS/MS	By MS/MS	37,3	91,71	0	323,31
Q9UID3	Q9UID3	Vacuolar protein sorting-associated protein 51 homolog	VPS51	4	4	4		By MS/MS					By MS/MS	By MS/MS	By MS/MS	By MS/MS	By MS/MS	By MS/MS	10,0	86,04	0	12,77
Q9NP79	Q9NP79	Vacuolar protein sorting-associated protein VTA1 homolog	VTA1	6	6	6	By MS/MS	By MS/MS	By MS/MS	By MS/MS	By MS/MS	By MS/MS	By MS/MS	By MS/MS	By MS/MS	By MS/MS	By MS/MS	By MS/MS	30,9	33,88	0	248,65
P23381	P23381	Tryptophan--tRNA ligase, cytoplasmic;T1-TrpRS;T2-TrpRS	WARS	27	27	27	By MS/MS	By MS/MS	By MS/MS	By MS/MS	By MS/MS	By MS/MS	By MS/MS	By MS/MS			By MS/MS	By MS/MS	65,0	53,17	0	323,31
O75083	O75083	WD repeat-containing protein 1	WDR1	16	16	16	By MS/MS	By MS/MS	By MS/MS	By MS/MS	By MS/MS	By MS/MS	By MS/MS	By MS/MS		By MS/MS	By MS/MS	By MS/MS	48,7	66,19	0	105,21

Q8NI36	Q8NI36	WD repeat-containing protein 36	WDR36	5	5	5	By MS/MS	By MS/MS	By MS/MS	By MS/MS	By MS/MS	By MS/MS	By MS/MS			By MS/MS	By MS/MS	By MS/MS	7,5	105,32	0	12,21
P61964;Q86VZ2	P61964	WD repeat-containing protein 5	WDR5	7	7	7	By MS/MS	By MS/MS	By MS/MS	By MS/MS	By MS/MS	By MS/MS			By MS/MS	By MS/MS	By MS/MS	By MS/MS	27,5	36,59	0	12,90
Q9GZS3	Q9GZS3	WD repeat-containing protein 61;WD repeat-containing protein 61, N-terminally processed	WDR61	5	5	5	By MS/MS	By MS/MS	By MS/MS	By MS/MS	By MS/MS	By MS/MS	By MS/MS	By MS/MS	By MS/MS	By MS/MS	By MS/MS	By MS/MS	31,5	33,58	0	94,75
Q9BQA1	Q9BQA1	Methylosome protein 50	WDR77	7	7	7	By MS/MS	By MS/MS	By MS/MS	By MS/MS	By MS/MS	By MS/MS	By MS/MS	By MS/MS	By MS/MS	By MS/MS			25,1	36,72	0	62,92
Q6UXN9	Q6UXN9	WD repeat-containing protein 82	WDR82	6	6	6	By MS/MS	By MS/MS	By MS/MS	By MS/MS	By MS/MS	By MS/MS	By MS/MS	By MS/MS	By MS/MS	By MS/MS	By MS/MS	By MS/MS	25,9	35,08	0	96,82
Q9NQW7	Q9NQW7	Xaa-Pro aminopeptidase 1	XPNPEP1	5	5	5	By MS/MS	By MS/MS	By MS/MS	By MS/MS	By MS/MS	By MS/MS	By MS/MS	By MS/MS	By MS/MS	By MS/MS	By MS/MS	By MS/MS	12,2	69,92	0	37,83
O14980	O14980	Exportin-1	XPO1	36	36	36	By MS/MS	By MS/MS	By MS/MS	By MS/MS	By MS/MS	By MS/MS	By MS/MS	By MS/MS	By MS/MS	By MS/MS	By MS/MS	By MS/MS	44,4	123,38	0	323,31
Q9HAV4	Q9HAV4	Exportin-5	XPO5	35	35	35	By MS/MS	By MS/MS	By MS/MS	By MS/MS	By MS/MS	By MS/MS	By MS/MS	By MS/MS	By MS/MS	By MS/MS	By MS/MS	By MS/MS	36,5	136,31	0	323,31
Q9UIA9	Q9UIA9	Exportin-7	XPO7	17	17	16	By MS/MS	By MS/MS	By MS/MS	By MS/MS	By MS/MS	By MS/MS	By MS/MS	By MS/MS	By MS/MS	By MS/MS	By MS/MS	By MS/MS	25,2	123,91	0	261,99
O43592	O43592	Exportin-T	XPOT	11	11	11	By MS/MS	By MS/MS	By MS/MS	By MS/MS	By MS/MS	By MS/MS	By MS/MS	By MS/MS	By MS/MS	By MS/MS	By MS/MS	By MS/MS	17,0	109,96	0	35,39
P13010	P13010	X-ray repair cross-complementing protein 5	XRCC5	37	37	37	By MS/MS	By MS/MS	By MS/MS	By MS/MS	By MS/MS	By MS/MS	By MS/MS	By MS/MS	By MS/MS	By MS/MS	By MS/MS	By MS/MS	70,4	82,70	0	323,31
P12956	P12956	X-ray repair cross-complementing protein 6	XRCC6	26	26	26	By MS/MS	By MS/MS	By MS/MS	By MS/MS	By MS/MS	By MS/MS	By MS/MS	By MS/MS	By MS/MS	By MS/MS	By MS/MS	By MS/MS	49,8	69,84	0	323,31
P54577	P54577	Tyrosine--tRNA ligase, cytoplasmic;Tyrosine--tRNA ligase, cytoplasmic, N-terminally processed	YARS	35	35	35	By MS/MS	By MS/MS	By MS/MS	By MS/MS	By MS/MS	By MS/MS	By MS/MS	By MS/MS	By MS/MS	By MS/MS	By MS/MS	By MS/MS	68,6	59,14	0	221,91
Q9Y2Z4	Q9Y2Z4	Tyrosine--tRNA ligase, mitochondrial	YARS2	5	5	5	By MS/MS	By MS/MS	By MS/MS	By MS/MS	By MS/MS	By MS/MS	By MS/MS	By MS/MS	By MS/MS	By MS/MS			15,3	53,20	0	50,09
P67809;Q9Y2T7	P67809	Nuclease-sensitive element-binding protein 1	YBX1	12	12	7	By MS/MS	By MS/MS	By MS/MS	By MS/MS	By MS/MS	By MS/MS	By MS/MS	By MS/MS	By MS/MS	By MS/MS	By MS/MS	By MS/MS	51,2	35,92	0	323,31
P16989	P16989	Y-box-binding protein 3	YBX3	8	3	3	By MS/MS	By MS/MS	By MS/MS	By MS/MS	By MS/MS	By MS/MS	By MS/MS	By MS/MS	By MS/MS	By MS/MS	By MS/MS	By MS/MS	28,2	40,09	0	22,70
P07947;P12931;P06241;P06239;P08631	P07947;P12931;P06241	Tyrosine-protein kinase Yes;Proto-oncogene tyrosine-protein kinase Src;Tyrosine-protein kinase Fyn	YES1;SRC;FYN	4	4	3		By MS/MS		By MS/MS		By MS/MS					By MS/MS		9,8	60,80	0	3,66
Q86U90	Q86U90	YrdC domain-containing protein, mitochondrial	YRDC	7	7	7	By MS/MS	By MS/MS	By MS/MS	By MS/MS	By MS/MS	By MS/MS	By MS/MS	By MS/MS	By MS/MS	By MS/MS	By MS/MS	By MS/MS	36,2	29,33	0	33,38
Q9Y5A9	Q9Y5A9	YTH domain-containing family protein 2	YTHDF2	4	4	4	By MS/MS	By MS/MS		By MS/MS	By MS/MS	By MS/MS				By MS/MS			9,2	62,33	0	9,96
P31946	P31946	14-3-3 protein beta/alpha;14-3-3 protein beta/alpha, N-terminally processed	YWHAB	20	14	13	By MS/MS	By MS/MS	By MS/MS	By MS/MS	By MS/MS	By MS/MS	By MS/MS	By MS/MS	By MS/MS	By MS/MS	By MS/MS	By MS/MS	65,0	28,08	0	323,31
P62258	P62258	14-3-3 protein epsilon	YWHAE	22	20	20	By MS/MS	By MS/MS	By MS/MS	By MS/MS	By MS/MS	By MS/MS	By MS/MS	By MS/MS	By MS/MS	By MS/MS	By MS/MS	By MS/MS	72,5	29,17	0	323,31
P61981	P61981	14-3-3 protein gamma;14-3-3 protein gamma, N-terminally processed	YWHAG	15	10	10	By MS/MS	By MS/MS	By MS/MS	By MS/MS	By MS/MS	By MS/MS	By MS/MS	By MS/MS	By MS/MS	By MS/MS	By MS/MS	By MS/MS	72,1	28,30	0	323,31
Q04917	Q04917	14-3-3 protein eta	YWHAH	14	10	10	By MS/MS	By MS/MS	By MS/MS	By MS/MS	By MS/MS	By MS/MS	By MS/MS	By MS/MS	By MS/MS	By MS/MS	By MS/MS	By MS/MS	55,7	28,22	0	323,31
P27348	P27348	14-3-3 protein theta	YWHAQ	19	15	15	By MS/MS	By MS/MS	By MS/MS	By MS/MS	By MS/MS	By MS/MS	By MS/MS	By MS/MS	By MS/MS	By MS/MS	By MS/MS	By MS/MS	66,5	27,76	0	323,31
P63104	P63104	14-3-3 protein zeta/delta	YWHAZ	26	26	20	By MS/MS	By MS/MS	By MS/MS	By MS/MS	By MS/MS	By MS/MS	By MS/MS	By MS/MS	By MS/MS	By MS/MS	By MS/MS	By MS/MS	73,5	27,75	0	323,31
Q8WU90	Q8WU90	Zinc finger CCCH domain-containing protein 15	ZC3H15	6	6	6	By MS/MS	By MS/MS	By MS/MS	By MS/MS	By MS/MS	By MS/MS		By MS/MS			By MS/MS	By MS/MS	19,0	48,60	0	26,76
Q86VM9	Q86VM9	Zinc finger CCCH domain-containing protein 18	ZC3H18	5	5	5	By MS/MS	By MS/MS	By MS/MS	By MS/MS	By MS/MS	By MS/MS				By MS/MS			7,5	106,38	0	12,01
Q7Z2W4	Q7Z2W4	Zinc finger CCCH-type antiviral protein 1	ZC3HAV1	11	11	10		By MS/MS	By MS/MS	By MS/MS		By MS/MS	By MS/MS	By MS/MS	By MS/MS	By MS/MS	By MS/MS	By MS/MS	16,4	101,43	0	12,89
Q86WB0	Q86WB0	Nuclear-interacting partner of ALK	ZC3HC1	5	5	5	By MS/MS	By MS/MS	By MS/MS	By MS/MS		By MS/MS					By MS/MS	By MS/MS	15,3	55,26	0	24,42
O75844	O75844	CAAX prenyl protease 1 homolog	ZMPSTE24	4	4	4	By MS/MS	By MS/MS	By MS/MS	By MS/MS		By MS/MS	By MS/MS	By MS/MS	By MS/MS	By MS/MS	By MS/MS	By MS/MS	12,0	54,81	0	15,22
Q969S3	Q969S3	Zinc finger protein 622	ZNF622	4	4	4	By MS/MS	By MS/MS	By MS/MS		By MS/MS	By MS/MS	By MS/MS						13,8	54,27	0	7,84
O75312	O75312	Zinc finger protein ZPR1	ZPR1	4	4	4	By MS/MS	By MS/MS	By MS/MS		By MS/MS	By MS/MS		By MS/MS			By MS/MS	By MS/MS	11,8	50,93	0	187,71
O43264	O43264	Centromere/kinetochore protein zw10 homolog	ZW10	4	4	4	By MS/MS	By MS/MS	By MS/MS	By MS/MS	By MS/MS	By MS/MS	By MS/MS	By MS/MS	By MS/MS	By MS/MS	By MS/MS	By MS/MS	6,9	88,83	0	81,59
P0DP25;P0DP24;P0DP23	P0DP25;P0DP24;P0DP23			7	7	7	By MS/MS	By MS/MS	By MS/MS	By MS/MS	By MS/MS	By MS/MS	By MS/MS	By MS/MS	By MS/MS	By MS/MS	By MS/MS	By MS/MS	55,7	16,84	0	157,42

a	Herein, and for all other columns, we followed the definitions and parameters as in MaxQuant (ver. 1.6.0.16). Identifier(s) of protein(s) contained in the Protein Group created by the built-in Andromeda searching
b	When the same peptide sequences is assigned to diverse proteins in the reference database, here only the ID of proteins with a more confident identification is reported for sake of clarity
c	Total number of peptide sequences associated with all proteins in the group. With the sequence coverage, can be used to qualitatively estimate the level of confidence in protein(s) identification(s). In this study, we<br>accepted from the original data set only identifications achieved with at least 4 peptides
d	Sum of the number of peptide sequences exclusively assigned to the protein group (unique peptides), and that of the peptide sequences shared with other protein groups ("razor peptides"). This parameter is used in quantitative analysis
e	Total number of peptide sequences exclusively assigned to the protein group. With the sequence coverage, can be used to qualitatively estimate the level of confidence in protein(s) identification(s). In this study, we accepted from the original data set only identification achieved with at least 2 unique peptide.
f	Indicates whether the peptide sequence assignment was obtained by analysis of daughter ions (by MS/MS) or only by matching the mass of a parent ion between runs (blank column). Data are reported for each experiment: FRW is for "forward" and REV is for "reverse" experiments; numers refers to the biological replicates, whereas letters to the technical replicates.
g	Sequence coverage of the best protein sequence contained in the group by the identified peptides, expressed as %.
h	Molecular weight of the leading protein sequence contained in the protein group.
i	In the MaxQuant algorithms, Q-values of a protein group are the name given to the adjusted p-values found using an optimised FDR approach. It ranges between 0 and 1, with higher values referring for an high probability to be an fault positive hit.
l	Score for the protein identification, determined by multiplying each peptide posterior error probabilities (PEPs) calculated by the Andromeda search engine for assigning statistical significance to the peptide identifications. The larger this score, the more certain is the identification of a protein.
