# Supplementary material for: Proteomic Analysis of Quercetin-Treated K562 Cells
Source: Int J Mol Sci. 2019 Dec 19;21(1):32. doi: 10.3390/ijms21010032 (PMC6981597; doi:10.3390/ijms21010032)
Supplement: Supplementary file 1 [file ijms-21-00032-s001.zip › jms-665671_Supplemetary materials/Table S2.pdf]

Table S2

Protein names	Gene names	Intensity L[a]	Intensity H[b]	Ratio H/L[c]	Ratio H/L normalized[d]	Log2_Ratio H/L normalized[e]	Ratio H/L variability [%][f]	Relative Abundance of proteins over all experiments (quercetin-treated/-untreated)[g]												T-test p-value[h]	-Log10 T-test p-value[i]	T-test Difference[l]	T-test Significant[m]
								Log2_Ratio H/L normalized FRW1	Log2_Ratio H/L normalized FRW1a	Log2_Ratio H/L normalized FRW2	Log2_H/L normalized FRW2a	Log2_H/L normalized FRW3	Log2_H/L normalized FRW3a	Log2_H/L normalized REV1	Log2_H/L normalized REV1a	Log2_H/L normalized REV2	Log2_H/L normalized REV2a	Log2_H/L normalized REV3	Log2_H/L normalized REV3a				
4F2 cell-surface antigen heavy chain	SLC3A2	596460000	1507000000	2,74	2,10	1,07	13,508	1,082294	1,244096	0,9989898	1,098958	0,9866658	1,177344	1,03977	0,9776829	0,9805372	0,8962331	1,038577	0,8869793	1,65E-12	11,78	1,034010534	+
Glutamate--cysteine ligase regulatory subunit	GCLM	34234000	84820000	2,66	1,96	0,97	4,6343	0,9776096	0,9930584	0,8949148	NaN	0,8665519	NaN	0,7445917	0,8584585	NaN	NaN	NaN	NaN	2,32E-06	5,64	0,889197489	+
Thioredoxin reductase 1, cytoplasmic	TXNRD1	209800000	475510000	2,33	1,90	0,93	23,167	0,9140286	0,9591025	0,7540323	0,8384274	0,965027	0,9616233	0,5941664	0,5727927	NaN	NaN	0,4487953	0,6195538	5,17E-07	6,29	0,762754923	+
Acid ceramidase;Acid ceramidase subunit alpha;Acid ceramidase subunit beta	ASAH1	21055000	48720000	2,57	1,78	0,84	14,994	NaN	0,3810618	NaN	NaN	0,4213715	1,034638	0,8637799	0,5949307	0,7880174	0,1388144	0,8420537	0,7583885	1,37E-04	3,86	0,647006229	+
Regulation of nuclear pre-mRNA domain-containing protein 1B	RPRD1B	17483000	31967000	1,94	1,67	0,74	8,2864	0,4538599	0,615228	0,773068	0,9595476	0,7277027	0,7538612	NaN	0,6573657	NaN	0,1021878	0,5792767	0,4533332	1,80E-05	4,74	0,607543079	+
Glutathione reductase, mitochondrial	GSR	573030000	1264600000	1,95	1,67	0,74	9,0476	0,7038095	0,7320521	0,7643462	0,6516832	0,7152784	0,7216789	0,7242137	0,8001648	NaN	NaN	0,715542	0,7390704	5,40E-13	12,27	0,726783901	+
Cystathionine gamma-lyase	CTH	46126000	96381000	2,09	1,61	0,69	21,89	0,81106	0,7571939	0,6948357	0,8189323	0,7446778	0,7934381	0,254594	NaN	0,5395311	0,5157124	NaN	NaN	5,88E-06	5,23	0,658886145	+
Replication protein A 70 kDa DNA-binding subunit	RPA1	95614000	194020000	1,89	1,60	0,68	10,035	0,7998334	1,038577	0,4635179	0,5962673	0,6280737	0,6277936	NaN	NaN	NaN	NaN	0,2068932	0,3215818	4,03E-04	3,39	0,58531717	+
Synaptic vesicle membrane protein VAT-1 homolog	VAT1	435970000	800050000	2,05	1,58	0,66	14,542	0,4232016	0,6215244	0,4661311	0,6724706	0,5934972	0,6179567	0,8033097	0,7941871	0,6005552	0,7634116	0,6089984	0,5519835	1,40E-09	8,85	0,626435588	+
Ferritin heavy chain	FTH1	27502000	63184000	2,16	1,58	0,66	29,313	1,547845	1,164078	0,8735771	NaN	1,214933	1,30906	0,3448285	0,2287264	0,2266318	0,4101239	0,06653696	NaN	1,96E-03	2,71	0,73863404	+
Annexin A5	ANXA5	196420000	363330000	2,04	1,54	0,62	15,592	0,7817374	0,6174865	0,5917751	0,6262054	0,8071076	0,6796039	0,4763298	0,3017047	0,424922	0,3594087	0,4115348	0,4467856	1,86E-07	6,73	0,543716798	+
D-3-phosphoglycerate dehydrogenase	PHGDH	526020000	995710000	1,89	1,54	0,62	18,546	0,9078905	0,7647708	0,8034751	0,8094145	0,8867452	0,7657045	-0,05289497	0,05227683	0,2329063	0,2282337	-0,005825792	-0,05388305	3,01E-03	2,52	0,444901133	+
Carbonyl reductase [NADPH] 1	CBR1	31390000	66675000	2,09	1,50	0,59	18,173	1,03991	0,7406254	0,7397617	0,8294436	0,3336527	0,7515494	0,5796629	0,4481609	0,5887087	0,5595407	0,5359534	0,4515408	2,23E-07	6,65	0,633209206	+
Coronin-1C	CORO1C	191260000	382020000	1,92	1,49	0,57	22,09	0,885262	0,7734057	0,9024981	0,8715283	0,9519607	0,6841903	0,5812066	0,4923147	0,4308206	0,497638	0,3769571	-0,0289403	1,22E-05	4,91	0,618236806	+
Hexokinase-1	HK1	265090000	411690000	1,67	1,48	0,57	23,051	0,6029319	0,6221806	0,5561105	0,6157929	0,60046	0,6682099	-0,03324471	0,08841301	0,09288389	0,06873958	0,01564032	0,104605	2,30E-03	2,64	0,333560248	+
Ferrochelatase, mitochondrial	FECH	59629000	124600000	2,22	1,48	0,57	22,666	0,7080548	1,052694	1,053111	0,9607341	0,7599228	1,060877	NaN	NaN	0,2178511	0,3880241	NaN	NaN	2,62E-04	3,58	0,77515872	+
Glycine--tRNA ligase	GARS	968460000	1773300000	1,96	1,48	0,57	27,851	0,6742799	0,705049	0,5520819	0,6559929	0,7329203	0,7448499	0,3800644	0,3769571	0,3563692	0,3025237	0,269512	0,3193867	1,47E-06	5,83	0,505832252	+
Sorting nexin-6	SNX6	34408000	57742000	1,90	1,47	0,56	12,499	0,9806103	0,6595592	0,6820339	0,4330663	0,5441886	0,5747313	NaN	NaN	0,1944653	0,0533895	NaN	0,1878943	1,19E-03	2,92	0,478882085	+
Annexin A1	ANXA1	3077300000	5689400000	1,89	1,46	0,55	15,842	0,6010309	0,5845778	0,6915342	0,6626612	0,7571086	0,7319652	0,3070788	0,3128975	0,2242873	0,2154923	0,2613503	0,3155663	1,08E-05	4,97	0,472129188	+
Adipocyte plasma membrane-associated protein	APMAP	47847000	86527000	1,85	1,46	0,55	18,878	0,7865964	0,4592217	NaN	0,5524754	0,5571895	0,4646683	NaN	NaN	NaN	NaN	0,3540007	0,4054478	7,53E-05	4,12	0,511371404	+
Protein phosphatase 1F	PPM1F	204510000	366170000	1,65	1,45	0,53	17,286	0,6035017	0,5097465	0,6047356	0,5386375	0,6200233	0,5591491	-0,08382914	-0,1254312	0,5572876	0,4063189	NaN	-0,2186319	4,63E-03	2,33	0,361046178	+
Selenide, water dikinase 1	SEPHS1	19428000	34608000	2,00	1,44	0,53	12,344	0,6366363	NaN	0,3689359	NaN	0,566182	0,5463635	0,144699	NaN	0,399554	0,5882289	NaN	NaN	3,80E-04	3,42	0,464371353	+
Glutamine--fructose-6-phosphate aminotransferase [isomerizing] 1	GFPT1	161180000	278680000	1,82	1,44	0,53	11,283	0,5586595	0,5383394	0,5047729	0,503247	0,5185351	0,5254675	0,4374408	0,6303123	0,4270697	0,345737	0,4614238	0,5489298	9,48E-11	10,02	0,499994566	+
Transaldolase	TALDO1	1747800000	3247900000	1,90	1,44	0,52	13,038	0,7463128	0,5965536	0,6698431	0,6596506	0,5745375	0,5912006	0,2156165	0,2734562	0,31823	0,3056787	0,3862592	0,3968171	1,76E-06	5,76	0,477846321	+
Alanine--tRNA ligase, cytoplasmic	AARS	793680000	1306700000	1,69	1,43	0,52	13,681	0,4916992	0,566182	0,5175276	0,5455731	0,6010309	0,6329195	0,2723816	0,3064956	0,417596	0,3526456	0,3295255	0,2966629	1,32E-07	6,88	0,444186638	+
Carbonic anhydrase-related protein	CA8	21667000	39808000	1,68	1,43	0,52	8,8991	0,4256741	0,4575418	0,5834228	0,5393326	0,5935928	0,6396026	0,4104497	0,4210482	0,068602	0,3860384	NaN	NaN	9,72E-06	5,01	0,452530488	+
Porphobilinogen deaminase	HMBS	368720000	636310000	1,66	1,42	0,51	20,599	0,4172719	0,5081244	0,569005	0,5573856	0,4652954	0,5298709	0,01863419	0,1583371	0,2192151	0,04194384	-0,0319019	0,02772026	1,50E-03	2,82	0,290075139	+
AFG3-like protein 2	AFG3L2	63177000	110350000	1,65	1,42	0,50	29,008	NaN	0,4256741	0,620211	0,5750218	NaN	0,4624712	0,02417773	0,5087328	0,458277	0,5103543	0,1619522	0,09464151	2,71E-04	3,57	0,384151375	+
Beta-lactamase-like protein 2	LACTB2	21013000	38558000	1,67	1,41	0,50	19,439	0,5845778	NaN	0,6514995	0,514905	0,539928	0,2422062	NaN	NaN	0,005040595	0,009060428	NaN	NaN	1,29E-02	1,89	0,363888212	+
Methylcrotonoyl-CoA carboxylase beta chain, mitochondrial	MCCC2	63323000	77805000	1,67	1,40	0,49	8,6763	0,5043661	0,5116704	0,261591	0,3939653	0,6146628	0,4894402	NaN	NaN	NaN	NaN	-0,6997707	-0,7630972	4,36E-01	0,36	0,164103467	
Carboxymethylenebutenolidase homolog	CMBL	158380000	279890000	1,73	1,40	0,48	13,643	0,4985572	0,4296427	0,4902621	0,5382402	0,5964581	0,5899552	0,1492593	0,1076879	0,1322477	0,02814484	0,1350094	0,1584662	3,48E-04	3,46	0,321160908	+
Glucosamine-6-phosphate isomerase 1	GNPDA1	52367000	84982000	1,73	1,40	0,48	12,428	0,4323181	0,540424	0,5164186	0,5624744	0,4688439	0,4922121	0,1488691	0,2501126	0,1245928	0,2194628	0,2881813	0,3256167	6,21E-06	5,21	0,364127216	+
Catalase	CAT	353030000	555930000	1,65	1,40	0,48	22,033	0,6291934	0,5308698	0,6849084	0,4824353	0,5721136	0,5768607	-0,2939072	-0,2434891	NaN	NaN	-0,08286615	-0,1568844	6,52E-02	1,19	0,269923451	
Replication protein A 14 kDa subunit	RPA3	18277000	32301000	1,89	1,39	0,48	8,5946	0,7591558	NaN	NaN	NaN	0,4889262	NaN	0,2843957	0,4363751	0,4212637	0,271067	0,1269729	0,2878269	7,27E-04	3,14	0,384497913	+
Cytochrome b-c1 complex subunit 8	UQCRQ	17367000	28374000	1,78	1,39	0,48	8,0068	0,4567011	0,4193231	NaN	NaN	0,6573657	NaN	NaN	0,230203	NaN	0,2071432	NaN	0,2123825	4,35E-03	2,36	0,363853092	+
ADP-ribosylation factor-like protein 8B	ARL8B	32276000	55008000	1,77	1,38	0,46	4,2462	0,4902621	0,4476321	0,5350576	0,46634	0,4431822	0,5504081	0,3112706	0,4616333	0,3731749	0,3214664	0,2907782	0,3782897	3,22E-09	8,49	0,422457943	+
Serine/threonine-protein phosphatase CPPED1	CPPED1	27004000	47400000	1,87	1,38	0,46	17,569	0,446468	0,7601783	0,7500061	0,7892702	0,9411814	0,8205671	NaN	NaN	0,2897164	0,1079557	0,00805652	0,2414741	9,14E-04	3,04	0,515487373	+
Dolichyl-diphosphooligosaccharide--protein glycosyltransferase subunit STT3A	STT3A	125770000	194260000	1,65	1,37	0,46	18,884	0,7422649	0,5331647	0,2224336	0,3878037	0,3319345	0,6053995	0,2146222	0,1919416	0,3331947	0,3186928	0,373843	0,3481191	6,40E-06	5,19	0,383617867	+
26S proteasome non-ATPase regulatory subunit 10	PSMD10	39159000	66878000	1,76	1,37	0,46	13,279	0,4439248	0,4972291	0,4900566	0,5129854	0,4683226	0,6542522	0,2772706	0,1696685	0,1012467	NaN	0,169412	0,03689012	2,08E-04	3,68	0,34738715	+
Palmitoyl-protein thioesterase 1	PPT1	44999000	74239000	1,66	1,37	0,45	9,6948	0,4033768	0,2547149	0,23842	0,123931	0,4340276	0,3325074	0,2977197	0,229834	0,2991274	-0,1380764	0,2665168	NaN	3,10E-04	3,51	0,249281751	+
Isocitrate dehydrogenase [NADP], mitochondrial	IDH2	185370000	306460000	1,71	1,37	0,45	10,496	0,5219553	0,4719678	0,5108607	0,4522791	0,349705	0,5824597	0,1722314	0,2963106	-0,1084408	0,3832757	0,4692609	0,4336005	2,48E-05	4,61	0,377955473	+
Nuclear autoantigenic sperm protein	NASP	532860000	796440000	1,57	1,36	0,45	22,68	0,2730981	0,3784007	0,4935448	0,5156115	0,3425547	0,4062101	0,5190386	0,9553872	0,9077367	1,071282	0,7964316	1,051303	1,09E-05	4,96	0,642549897	+
Macrophage-capping protein	CAPG	123540000	184800000	1,90	1,36	0,44	7,3163	0,2327835	0,3221589	0,7577913	NaN	0,4158668	0,4522791	NaN	0,3547907	0,2860532	0,3345683	NaN	NaN	2,35E-04	3,63	0,39453648	+
Hemoglobin subunit epsilon	HBE1	6670600	13915000	2,23	1,36	0,44	15,591	-0,1037825	0,03393393	NaN	NaN	0,1515991	0,4318903	0,6879565	0,5617904	0,4428639	0,520045	0,4978422	0,487795	1,28E-03	2,89	0,3711934	+
Cytochrome b-c1 complex subunit 7	UQCRB	33388000	54788000	1,79	1,36	0,44	10,369	0,5061957	0,4358419	0,3805078	1,254896	0,6495695	0,7583031	0,10742	0,004465491	NaN	0,2152437	0,1989969	0,2621926	2,29E-03	2,64	0,433966643	+
Glyoxylate reductase/hydroxypyruvate reductase	GRHPR	141890000	232490000	1,64	1,36	0,44	29,993	0,5463635	0,2445218	0,3914378	0,3951724	0,5054845	0,3836075	0,232538	0,215368	0,1195554	0,04096256	0,1759396	0,05672221	1,39E-04	3,86	0,275639449	+
Glucose-6-phosphate 1-dehydrogenase	G6PD	1685900000	2335800000	1,66	1,35	0,44	13,478	0,3983503	0,4339209	0,4868688	0,4943644	0,5135918	0,4577518	0,2107629	0,1402552	NaN	NaN	0,1938348	0,1611782	5,01E-05	4,30	0,349087915	+
Ornithine aminotransferase, mitochondrial;Ornithine aminotransferase, hepatic form;Ornithine aminotransferase, renal form	OAT	927590000	1546800000	1,69	1,35	0,43	16,82	0,5702686	0,5886127	0,5999842	0,5157124	0,4661311	0,5479433	-0,1857859	-0,1287399	0,09653179	0,1508197	-0,06916424	-0,06250495	1,71E-02	1,77	0,257484072	+
Phosphoacetylglucosamine mutase	PGM3	79851000	122880000	1,68	1,35	0,43	7,8387	0,1604039	0,4374408	0,6172044	0,5363513	0,4830547	0,5367492	NaN	0,2841587	NaN	0,2747686	NaN	NaN	1,49E-04	3,83	0,416266464	+
Peroxiredoxin-5, mitochondrial	PRDX5	96236000	186610000	1,90	1,34	0,43	20,968	0,5302706	0,3823906	0,4522791	0,5673507	0,4842929	0,6524177	0,3595212	0,2997136	0,2773897	0,3015876	0,4266405	0,3878037	6,13E-08	7,21	0,426804813	+
V-type proton ATPase subunit C 1	ATP6V1C1	31911000	54687000	2,03	1,34	0,43	10,627	0,5437928	NaN	0,2785796	0,4230941	0,4094724	NaN	NaN	0,4910835	0,4091466	0,28889	NaN	NaN	3,22E-05	4,49	0,406294146	+
Beta-hexosaminidase subunit beta;Beta-hexosaminidase subunit beta chain B;Beta-hexosaminidase subunit beta chain A	HEXB	21380000	36109000	1,75	1,33	0,41	9,755	0,3005338	0,3214664	NaN	0,3649085	0,3828332	0,5865967	NaN	0,679694	NaN	NaN	0,8368937	0,8446267	2,77E-04	3,56	0,539694127	+
Dihydropteridine reductase	QDPR	21697000	37711000	1,69	1,33	0,41	8,409	0,6106057	0,4381864	0,4184599	NaN	0,449535	0,4408458	0,5005982	0,2476845	NaN	NaN	NaN	-0,4056999	1,95E-02	1,71	0,33752694	+
Replication protein A 32 kDa subunit	RPA2	26448000	45273000	1,65	1,33	0,41	18,47	0,5687131	0,7794699	0,3103401	0,4708232	0,6498454	0,2881813	NaN	NaN	0,1688987	0,1298765	NaN	-0,06023178	3,63E-03	2,44	0,367324053	+
Pyruvate kinase PKLR	PKLR	78248000	130790000	1,70	1,33	0,41	19,361	0,2678354	0,4672795	0,2940766	0,4311415	0,5620837	0,5217544	NaN	0,3945141	NaN	NaN	0,3613192	0,2913677	3,22E-06	5,49	0,399041351	+
Protein kinase C beta type	PRKCB	78838000	139600000	1,84	1,33	0,41	13,092	0,2237931	0,3268825	0,4122938	0,3736205	0,5311693	0,5863084	0,2533843	0,3892364	0,3241193	0,4481609	0,1851053	0,296428	4,76E-07	6,32	0,362541802	+
Single-stranded DNA-binding protein, mitochondrial	SSBP1	485150000	776220000	1,65	1,32	0,41	25,999	0,4682183	0,5136929	0,6041662	0,5829413	0,3757346	0,4775738	0,0354831	0,03731201	0,04558271	0,1145003	0,1386835	0,1292171	9,92E-04	3,00	0,29359215	+
Isocitrate dehydrogenase [NADP] cytoplasmic	IDH1	200180000	311760000	1,71	1,32	0,40	14,578	0,4061012	0,466862	0,5440896	0,4177041	0,3087104	0,3956113	0,2454957	0,2395203	NaN	NaN	0,143524	0,1244606	3,84E-05	4,42	0,329207904	+
Tyrosine--tRNA ligase, cytoplasmic;Tyrosine--tRNA ligase, cytoplasmic, N-terminally processed	YARS	688770000	1192000000	1,60	1,32	0,40	14,722	0,4708232	0,4591168	0,3758458	0,3766238	0,6039764	0,5388361	-0,09075686	-0,08098795	0,0416635	0,1068843	-0,00492797	0,2376861	4,94E-03	2,31	0,252898596	+
Nuclear protein localization protein 4 homolog	NPLOC4	35999000	45331000	1,68	1,32	0,40	24,863	0,3598585	0,2979544	0,3655806	NaN	0,3951724	0,3146386	0,4969225	0,2956054	NaN	NaN	NaN	-1,0713	3,47E-01	0,46	0,181804076	
NADH dehydrogenase [ubiquinone] 1 alpha subcomplex subunit 12	NDUFA12	20899000	32550000	1,59	1,31	0,39	13,078	0,342441	0,7260464	0,329181	0,5035523	0,5296711	0,3983503	0,3203114	0,477781	NaN	NaN	0,4966157	NaN	5,31E-06	5,27	0,45821669	+
14 kDa phosphohistidine phosphatase	PHPT1	39779000	60657000	1,61	1,31	0,38	16,563	0,4474205	0,3480057	NaN	NaN	NaN	NaN	0,007769512	0,01392662	NaN	-0,01732945	-0,09249397	-0,2185816	4,70E-01	0,33	0,069816768	
Lon protease homolog, mitochondrial	LONP1	308910000	451770000	1,51	1,30	0,38	17,568	0,2817873	0,3299846	0,4013028	0,5836154	0,2635152	0,3997727	NaN	-0,2507833	-0,01361091	0,07477944	0,2492632	0,2072682	7,18E-03	2,14	0,229717696	+
Enoyl-CoA delta isomerase 1, mitochondrial	ECI1	127880000	216090000	1,74	1,29	0,37	13,905	0,4659222	0,389016	0,4853237	0,4353086	0,4364817	0,4774701	0,009633875	0,0353423	0,02304246	NaN	-0,06502312	-0,04686145	8,00E-03	2,10	0,240514215	+
Cystatin-B	CSTB	139770000	225330000	1,65	1,29	0,37	5,6324	0,3483458	0,4603756	0,484499	0,4547023	0,4331732	0,6721085	-0,01177707	0,2834477	0,07189995	NaN	0,06281248	NaN	1,19E-03	2,92	0,325958748	+
Malectin	MLEC	20369000	29995000	1,69	1,29	0,37	5,49	0,3680419	0,4239545	0,2608687	0,2487777	0,3837181	0,4161913	NaN	NaN	0,1448295	0,2042659	NaN	NaN	7,60E-05	4,12	0,306330953	+
Histone H2A.V;Histone H2A.Z	H2AFV;H2AFZ	97360000	173100000	1,96	1,29	0,37	10,364	0,2102642	0,6010309	NaN	NaN	NaN	NaN	0,3944044	0,5325662	0,4900566	0,5054845	0,1966071	0,4642501	8,48E-05	4,07	0,424332997	+
Thioredoxin	TXN	120020000	219600000	1,95	1,29	0,37	6,7089	0,3253864	0,2775088	0,3115032	0,4652954	0,4327456	0,5965536	0,4608998	0,5056878	0,3770682	0,429857	0,5470549	0,3939653	8,70E-09	8,06	0,426960488	+
Transportin-1	TNPO1	343250000	509120000	1,57	1,29	0,37	15,332	0,2152437	0,4158668	0,4277135	0,26123	0,3810618	0,3261922	0,1491292	0,1079557	0,1253867	0,1399933	0,4152178	0,3900073	1,04E-05	4,98	0,279583154	+
Malate dehydrogenase, cytoplasmic	MDH1	329390000	515450000	1,57	1,29	0,36	14,738	0,3639	0,2193389	0,3752896	0,2543522	0,3606452	0,3018217	0,06419311	0,1835818	0,1107641	0,1389455	0,08909126	0,0938305	5,54E-05	4,26	0,212979496	+
Erlin-2	ERLIN2	98320000	151790000	1,64	1,29	0,36	14,368	0,3109217	0,4129441	0,2741722	NaN	0,4140272	0,4706149	0,3982408	0,1685137	0,3315905	0,3834969	NaN	NaN	2,84E-06	5,55	0,351613584	+
Probable ATP-dependent RNA helicase DDX6	DDX6	330430000	506790000	1,41	1,28	0,36	16,714	0,2326607	0,1807844	0,5020251	0,4265331	0,2288495	0,117163	0,8620347	0,1285573	NaN	NaN	0,4142438	0,5295712	8,16E-04	3,09	0,362242284	+
Acyl-coenzyme A thioesterase 13;Acyl-coenzyme A thioesterase 13, N-terminally processed	ACOT13	62354000	105100000	1,72	1,28	0,36	16,037	0,3964884	0,3946239	0,3982408	0,7030121	0,4869717	0,5413166	0,2492632	0,2607483	0,1721035	0,3993352	0,2838032	0,05672221	1,61E-05	4,79	0,361885758	+
Histone H4	HIST1H4A	1478500000	2893300000	2,06	1,28	0,35	24,4	0,9160179	0,9523335	1,415813	1,520498	0,2224336	0,372172	0,353662	0,4901594	0,06777637	0,1571731	0,3263073	0,3637879	1,39E-03	2,86	0,596511135	+
Aminopeptidase B	RNPEP	195990000	309110000	1,59	1,28	0,35	12,052	0,3139424	0,4140272	0,3257317	0,3181144	0,2723816	0,3727293	0,4803685	0,4463621	0,5278711	0,2834477	0,4768483	0,4478436	7,49E-09	8,13	0,389972332	+
	SLC35A4	13726000	20985000	1,53	1,28	0,35	24,107	0,3774015	0,4235244	NaN	NaN	0,5302706	NaN	NaN	0,021764	-0,3796763	-0,01807427	-0,3378857	-0,05198236	5,79E-01	0,24	0,070667719	
Glutathione S-transferase omega-1	GSTO1	763880000	1364300000	1,66	1,28	0,35	13,6	0,3041605	0,3441468	0,3144065	0,3733977	0,3246955	0,4022855	0,3576083	0,3466449	0,3829439	0,3889059	0,3931965	0,364012	4,11E-13	12,39	0,35803366	+
4-trimethylaminobutyraldehyde dehydrogenase	ALDH9A1	117090000	175350000	1,60	1,27	0,35	15,498	0,582845	0,3354833	NaN	0,2678354	0,7670615	0,3384529	0,05130255	NaN	NaN	NaN	0,4676968	0,2341334	1,85E-03	2,73	0,380601363	+
Acylglycerol kinase, mitochondrial	AGK	66143000	106510000	1,73	1,27	0,35	6,3205	0,2504765	0,2465906	0,3368547	NaN	0,1676149	0,3173041	NaN	NaN	NaN	NaN	0,3383388	0,008486926	1,79E-03	2,75	0,237952368	+
Surfeit locus protein 4	SURF4	112110000	174230000	1,63	1,27	0,35	13,08	0,5005982	0,5096452	0,4758112	0,4693651	0,7018595	0,6096604	0,1392075	0,1852322	0,1440464	0,2147466	0,09071802	0,203765	1,19E-04	3,93	0,35372128	+
Cysteine--tRNA ligase, cytoplasmic	CARS	104350000	168500000	1,67	1,27	0,35	17,931	0,5526721	0,9054279	0,4636224	0,7125518	0,4915966	0,4633086	0,08120319	0,1610493	0,3083608	-0,1477774	-0,34828	0,0439044	1,35E-02	1,87	0,307303309	+
Glyoxalase domain-containing protein 4	GLOD4	65418000	107040000	1,70	1,27	0,34	12,81	0,3431235	0,3596336	0,3278023	NaN	0,5475485	0,3258469	0,2142491	0,1610493	0,113167	0,2198345	0,1944653	0,181675	2,61E-05	4,58	0,27167228	+
Metastasis-associated protein MTA2	MTA2	47074000	69578000	1,90	1,27	0,34	15,017	0,4152178	0,7512066	NaN	NaN	NaN	NaN	NaN	0,2081427	NaN	0,3075451	0,2585793	0,2347465	7,32E-03	2,14	0,362573	+
Asparagine synthetase [glutamine-hydrolyzing]	ASNS	560370000	943160000	1,59	1,27	0,34	25,435	0,4683226	0,5011079	0,4869717	0,3853759	0,5657921	0,4828483	-0,2961729	-0,214123	NaN	NaN	-0,2002495	-0,1986426	1,24E-01	0,91	0,198123057	
Phosphoserine aminotransferase	PSAT1	694280000	1144200000	1,61	1,27	0,34	19,13	0,4494293	0,5539502	0,444243	0,4108837	0,3643483	0,4905701	-0,1572383	-0,1139884	-0,06703179	-0,02001858	NaN	NaN	2,86E-02	1,54	0,235514749	+
Cytochrome b-c1 complex subunit 2, mitochondrial	UQCRC2	814980000	1269200000	1,53	1,27	0,34	10,223	0,372172	0,4087119	0,4687397	0,3633393	0,3535491	0,3081279	0,03195977	0,240009	0,1580785	0,1515991	0,320658	0,2507191	6,94E-06	5,16	0,285638611	+

Sepiapterin reductase	SPR	42664000	62722000	1,82	1,26	0,34	12,472	0,4361619	0,3204269	0,1301402	0,4075159	0,4324249	0,4172719	0,04012096	0,324004	0,09477648	0,2548359	0,01063669	0,177854	1,72E-04	3,76	0,253847484	+
UDP-glucose:glycoprotein glucosyltransferase 1	UGGT1	148140000	194460000	1,35	1,26	0,34	12,042	0,5844815	0,363115	0,2618317	0,395721	0,4312485	0,4463621	-0,2663333	0,1158324	0,0446039	0,2717842	NaN	NaN	7,73E-03	2,11	0,264864697	+
Sialic acid synthase	NANS	174760000	313490000	1,63	1,26	0,34	22,03	0,5028398	0,6363578	0,5545398	0,5478446	0,5107595	0,7270927	-0,1692092	0,1627257	0,1821838	0,1624679	NaN	NaN	2,02E-03	2,70	0,381760241	+
Casein kinase II subunit alpha;Casein kinase II subunit alpha 3	CSNK2A1;CSNK2A3	197020000	333670000	1,75	1,26	0,34	17,887	0,4816089	0,533962	0,2406196	0,4948763	0,281906	0,3407331	0,2086421	0,2046416	0,1723594	0,231801	NaN	NaN	3,97E-05	4,40	0,319114998	+
Superoxide dismutase [Mn], mitochondrial	SOD2	102020000	172120000	1,65	1,26	0,33	8,7452	0,3605329	0,3774015	0,5645245	0,2858165	0,466862	0,3260771	NaN	-0,1784857	-0,5439299	NaN	0,000721115	0,02828617	1,51E-01	0,82	0,168780599	
Ragulator complex protein LAMTOR2	LAMTOR2	11799000	20413000	1,66	1,26	0,33	6,553	0,3034593	0,3893466	NaN	0,2565276	0,3956113	0,3067288	0,5681294	0,09868897	0,418136	0,1754288	0,325962	0,2716648	7,61E-06	5,12	0,319062135	+
Hepatoma-derived growth factor	HDGF	64561000	111690000	1,57	1,25	0,33	17,386	0,3836075	0,1709506	0,2681949	0,3263073	0,3047447	0,2720232	0,1649149	0,1469159	0,4766409	0,458172	NaN	NaN	2,19E-05	4,66	0,297247188	+
Dolichyl-diphosphooligosaccharide--protein glycosyltransferase subunit 1	RPN1	607620000	933510000	1,62	1,25	0,32	12,376	0,3470986	0,2839217	0,3041605	0,3218127	0,2347465	0,366476	0,3819478	0,2963106	0,2406196	0,1703097	0,2781037	0,3298698	2,97E-09	8,53	0,296281445	+
Transmembrane protein 43	TMEM43	132970000	184400000	1,47	1,25	0,32	9,8721	0,3983503	0,4825385	0,3913278	0,2487777	0,05227683	0,3936358	0,1578198	NaN	0,4856329	0,4534386	NaN	NaN	1,58E-04	3,80	0,34042203	+
Diablo homolog, mitochondrial	DIABLO	125970000	186690000	1,81	1,25	0,32	7,5217	0,2967804	0,3650205	0,2918393	0,4138107	0,3470986	0,4228789	0,1048734	0,1579492	0,2600257	0,008630365	0,1208827	0,2172308	3,74E-05	4,43	0,250585034	+
Glutathione synthetase	GSS	85269000	139640000	1,54	1,25	0,32	7,4241	0,3378824	0,2936059	0,5818814	0,4534386	0,4244922	0,3792882	NaN	NaN	NaN	NaN	-0,1703453	-0,002729279	1,41E-02	1,85	0,287189264	+
Dihydrolipoyllysine-residue succinyltransferase component of 2-oxoglutarate dehydrogenase complex, mitochondrial	DLST	31240000	50625000	1,62	1,25	0,32	2,1099	NaN	0,2972502	NaN	NaN	0,02219017	0,3455099	NaN	NaN	0,3279173	NaN	0,3272275	0,2614706	3,20E-03	2,49	0,263594272	+
Succinate dehydrogenase [ubiquinone] flavoprotein subunit, mitochondrial	SDHA	340080000	512750000	1,53	1,25	0,32	14,669	0,3253864	0,2418402	0,212258	0,3113869	0,3635636	0,3596336	0,1473066	0,1386835	NaN	0,539928	0,3460776	0,0790197	3,54E-05	4,45	0,278644017	+
Annexin A7	ANXA7	371330000	588260000	1,64	1,25	0,32	17,04	0,5089356	0,5206485	0,3286067	0,4141355	0,372172	0,4683226	0,3078948	0,01221106	-0,2052473	-0,09370968	-0,2273035	-0,08275924	4,08E-02	1,39	0,193658925	
Histone H2B type 1-M;Histone H2B type 1-N;Histone H2B type 1-H;Histone H2B type 2-F;Histone H2B type 1-C/E/F/G/I;Histone H2B type 1-D;Histone H2B type 1-K;Histone H2B type 1-L;Histone H2B type F-S	H;HIST2H2BF;HIST1H2BC;HIST1H	1489800000	2901500000	1,97	1,25	0,32	16,444	0,342441	0,3328511	0,6052098	0,7030121	-0,1894335	-0,006753202	0,3911079	0,5011079	0,1547128	0,2369518	0,5619859	0,733094	9,41E-04	3,03	0,363857297	+
E3 ubiquitin-protein ligase UBR4	UBR4	249190000	359930000	1,39	1,24	0,32	17,896	0,2452522	0,4355219	0,2183473	0,3351402	0,3530974	0,3149864	0,06584801	0,3637879	0,3979125	0,1842168	0,09342498	0,0408223	4,15E-05	4,38	0,254029817	+
Nicotinate-nucleotide pyrophosphorylase [carboxylating]	QPRT	20190000	35308000	1,74	1,24	0,31	20,09	NaN	NaN	NaN	0,6369146	0,05935536	0,485736	0,1115655	0,08038475	0,3170725	0,2953704	-0,005217574	0,2002533	9,19E-03	2,04	0,242381646	+
Acylphosphatase-1	ACYP1	7436500	12346000	1,82	1,24	0,31	19,858	0,06087747	0,3751785	NaN	NaN	NaN	NaN	0,123931	0,2810751	0,2795307	0,3184614	0,3426685	-0,01595754	3,49E-03	2,46	0,220720654	+
Endoplasmic reticulum resident protein 44	ERP44	250150000	355170000	1,48	1,24	0,31	14,475	0,3422133	0,3381105	0,370499	0,3045111	0,2777467	0,3307878	0,07614867	0,4520683	-0,189598	-0,165127	NaN	NaN	1,56E-02	1,81	0,213736037	+
Very long-chain specific acyl-CoA dehydrogenase, mitochondrial	ACADVL	121390000	185670000	1,41	1,24	0,31	8,5807	0,1263122	0,2142491	0,4492181	0,2474415	0,3096419	0,1466552	0,7207162	NaN	NaN	NaN	0,3223896	0,2703495	8,66E-04	3,06	0,311885935	+
Exportin-7	XPO7	82678000	123150000	1,46	1,24	0,31	14,089	0,2679552	0,3463045	0,1768333	0,3398215	0,4087119	0,4363751	0,0647449	NaN	0,2360947	0,05990899	NaN	0,2158649	1,66E-04	3,78	0,25526148	+
Transmembrane emp24 domain-containing protein 2	TMED2	17296000	26637000	1,85	1,24	0,31	14,575	0,1504297	NaN	0,190678	0,2486562	0,1655583	0,1889072	0,2519311	0,3725064	0,296428	0,2665168	NaN	-0,3176604	1,36E-02	1,87	0,181395127	+
Apoptosis inhibitor 5	API5	118480000	202080000	1,57	1,23	0,30	7,5572	0,2551986	0,2853431	0,3296403	0,3401635	0,2995964	0,1569143	0,1804026	0,4300712	NaN	NaN	0,3378824	0,2795307	1,12E-06	5,95	0,289474301	+
Programmed cell death 6-interacting protein	PDCD6IP	1117200000	1623300000	1,51	1,23	0,30	15,068	0,2211965	0,3278023	0,3057954	0,3360548	0,3319345	0,311038	0,2403754	0,2072682	0,2258919	0,2635152	0,09868897	0,1923205	8,72E-08	7,06	0,255156811	+
Coatomer subunit beta	COPB1	247420000	340190000	1,50	1,23	0,30	23,841	0,3049782	0,3328511	0,3039269	0,3317052	0,3414165	0,3366261	0,2862897	0,3036931	0,3336527	0,4193231	0,262794	0,4420146	1,52E-10	9,82	0,333272599	+
Cat eye syndrome critical region protein 5	CECR5	63375000	94739000	1,73	1,23	0,30	21,766	NaN	0,2600257	NaN	0,6926057	0,3377682	0,4643545	NaN	-0,2460019	-0,8696225	NaN	NaN	NaN	6,66E-01	0,18	0,106521619	
Apolipoprotein O	APOO	21953000	33563000	1,57	1,23	0,30	7,9953	0,2860532	0,4257815	0,3761792	NaN	0,4441369	0,3663642	-0,03308228	NaN	0,05616735	0,03562387	-0,03038346	NaN	1,33E-02	1,87	0,214093396	+
Ubiquitin-like modifier-activating enzyme 1	UBA1	1315700000	1900000000	1,47	1,23	0,30	11,099	0,3595212	0,3292958	0,3175356	0,3354833	0,4165155	0,4088205	0,01692422	0,01506926	0,06460693	0,02133754	0,102994	0,06846439	1,41E-03	2,85	0,204714022	+
Ras-related protein Rab-27A	RAB27A	70189000	105680000	1,56	1,23	0,30	17,688	0,3054453	0,533663	0,3762904	0,1759396	0,286408	0,4119685	NaN	-0,3134053	NaN	NaN	NaN	-0,2903741	1,43E-01	0,85	0,185741933	
Pyrroline-5-carboxylate reductase 1, mitochondrial	PYCR1	134420000	209530000	1,50	1,23	0,30	12,438	0,3478923	0,345737	0,4826418	0,3869213	0,3128975	0,3936358	0,2343786	0,006333955	-0,02478071	-0,01568047	-0,01798668	0,0292761	3,72E-03	2,43	0,206772217	+
Histone H3.2;Histone H3.1t;Histone H3.3;Histone H3.1;Histone H3.3C	H3A;HIST3H3;H3F3A;HIST1H3A;H	373130000	590860000	1,98	1,23	0,30	25,566	NaN	0,9377963	1,023184	1,325847	0,2686739	0,5584636	0,4701985	0,4331732	0,1994997	0,153935	0,1870075	0,1901724	1,51E-03	2,82	0,522541016	+
WD repeat-containing protein 1	WDR1	401130000	512470000	1,53	1,23	0,30	6,9109	0,3624418	0,2856981	0,2341334	0,3660284	0,3117356	0,3723949	0,1439158	0,215368	NaN	0,1365852	0,24732	0,1139672	4,76E-06	5,32	0,253598947	+
Ras-related protein Rab-31	RAB31	5248600	6715700	1,53	1,23	0,29	18,426	0,2017593	0,418028	NaN	0,1772162	NaN	0,2380531	-0,1362835	0,2348692	NaN	NaN	NaN	NaN	5,05E-02	1,30	0,188940361	
Succinyl-CoA ligase [ADP/GDP-forming] subunit alpha, mitochondrial	SUCLG1	21066000	38898000	1,69	1,23	0,29	14,145	-0,2230369	0,28889	NaN	NaN	-0,2334303	0,4047941	NaN	0,4875892	NaN	0,186247	0,2729787	0,3222744	9,13E-02	1,04	0,188288271	
Filamin-A	FLNA	4326600000	5916400000	1,47	1,22	0,29	11,016	0,2795307	0,256286	0,1781091	0,2157407	0,3210045	0,3193867	0,279293	0,2679552	0,3101074	0,3083608	0,2803624	0,3402774	3,45E-10	9,46	0,27970116	+
Septin-2	SEPTIN2	268920000	395600000	1,57	1,22	0,29	23,001	0,4620524	0,4493236	0,2515676	0,2533843	0,2172308	0,3696061	-0,1375685	-0,05101046	-0,0124754	0,001153714	NaN	NaN	2,74E-02	1,56	0,180326418	+
Cation-independent mannose-6-phosphate receptor	IGF2R	163520000	237700000	1,38	1,22	0,29	13,694	0,4233092	0,4350952	0,2418402	0,3389092	0,2999479	0,4970246	0,4969225	0,1086248	0,1075539	0,05088473	0,2078928	0,2510827	4,53E-05	4,34	0,288257321	+
Ribonuclease inhibitor	RNH1	556450000	856790000	1,56	1,22	0,29	10,271	0,4468915	0,3663642	0,4061012	0,3234277	0,2558028	0,3801753	0,04124304	0,07217448	0,06115411	0,1163647	0,07942928	0,1025909	4,72E-04	3,33	0,220976599	+
High mobility group protein B2	HMGB2	90192000	161760000	1,78	1,22	0,29	9,4696	0,4901594	0,4818156	0,1740229	NaN	0,1859933	0,2608687	0,3632271	0,3178829	0,2419622	0,2928995	0,3926472	0,2588203	2,07E-06	5,68	0,314572651	+
Lysine-specific histone demethylase 1A	KDM1A	89196000	112300000	1,44	1,22	0,29	14,859	0,2833292	0,3175356	0,3578334	0,1478277	0,2403754	0,4243847	NaN	NaN	0,5129854	0,2766753	0,1558787	-0,2324638	3,75E-03	2,43	0,248436169	+
Cytoplasmic aconitate hydratase	ACO1	117270000	170250000	1,58	1,22	0,29	13,972	0,2236697	0,3219281	0,1558787	0,2753647	0,2171066	0,3336527	0,2016338	0,1638852	0,3702759	0,07942928	0,000576887	0,2229281	2,34E-05	4,63	0,213860816	+
Coatomer subunit delta	ARCN1	100510000	155530000	1,51	1,22	0,29	8,4152	0,1814206	0,3369689	0,335369	0,2567692	0,2671163	0,2507191	NaN	0,1388144	NaN	0,2158649	NaN	NaN	1,94E-05	4,71	0,247880286	+
Dihydrolipoyl dehydrogenase, mitochondrial	DLD	481740000	792540000	1,57	1,22	0,29	10,617	0,3117356	0,2341334	0,2258919	0,2263851	0,2816686	0,3141744	0,3230819	0,3476657	NaN	NaN	0,2378085	0,3798428	5,01E-08	7,30	0,288238783	+
Nicotinamide phosphoribosyltransferase	NAMPT	274200000	422180000	1,58	1,22	0,29	11,309	0,3272275	0,3442604	0,2878269	0,2822618	0,4077334	0,3280323	NaN	NaN	NaN	NaN	-0,3540771	0,2236697	3,09E-02	1,51	0,230866875	
Protein transport protein Sec23B	SEC23B	78588000	114080000	1,57	1,22	0,28	14,262	0,3318198	0,7143115	0,4658178	0,533962	0,2032638	0,4437128	0,3858175	0,2807188	0,1502997	0,2381754	-0,02426711	0,3607576	7,51E-05	4,12	0,340365801	+
Ester hydrolase C11orf54	C11orf54	13101000	22867000	1,69	1,22	0,28	1,2227	0,2933705	0,2944296	0,2916035	0,445091	0,4564908	0,4277135	NaN	NaN	0,1471764	0,1818022	0,09261344	0,09071802	1,77E-04	3,75	0,272100895	+
Peptidyl-prolyl cis-trans isomerase FKBP1A	FKBP1A	41659000	63038000	1,49	1,21	0,28	8,7464	0,1306673	0,3330801	0,1384213	0,08868441	0,3691593	0,1820566	0,09585687	0,3148704	0,2702299	0,1749178	0,1305355	0,2053925	1,55E-05	4,81	0,20282268	+
Glutamate dehydrogenase 1, mitochondrial;Glutamate dehydrogenase 2, mitochondrial	GLUD1;GLUD2	288380000	439770000	1,65	1,21	0,28	11,453	0,3286067	0,2057679	0,2247812	0,3029917	0,3789555	0,3725064	0,2490205	0,5545398	NaN	NaN	0,07819984	0,1076879	1,36E-04	3,87	0,280305737	+
Vesicle-trafficking protein SEC22b	SEC22B	61594000	100960000	1,68	1,21	0,28	7,952	0,1669724	0,2496274	0,3634514	0,24245	0,3227358	0,3111543	0,1278974	0,2842772	0,3290661	NaN	0,3233124	0,3242345	2,02E-07	6,69	0,276834457	+
Transferrin receptor protein 1;Transferrin receptor protein 1, serum form	TFRC	1840200000	2600300000	1,55	1,21	0,28	18,186	0,1323794	0,1479579	0,2168583	0,1604039	0,2407417	0,2987756	0,4134858	0,3934163	0,2880631	0,1469159	0,2441563	0,3614315	2,36E-06	5,63	0,253715483	+
Ubiquitin carboxyl-terminal hydrolase 14	USP14	379160000	579060000	1,46	1,21	0,28	8,5785	0,2956054	0,2756031	0,3428961	0,2554403	0,2946649	0,2692725	0,2352368	NaN	NaN	NaN	0,249506	0,1574319	2,94E-07	6,53	0,263961895	+
Calpain small subunit 1	CAPNS1	205720000	319910000	1,59	1,21	0,28	11,215	0,4250295	0,2856981	0,2045164	0,3166091	0,2894804	0,2945473	0,05450115	-0,08213291	0,03955951	0,005184378	-0,000995779	-0,119965	1,90E-02	1,72	0,14266935	+
Sideroflexin-1	SFXN1	108700000	172810000	1,57	1,21	0,28	13,613	0,3338817	0,2444	0,3790664	0,3756233	0,2924284	0,381837	-0,03162166	0,2419622	-0,03956246	-0,02605815	-0,08489984	0,1894135	4,46E-03	2,35	0,188039208	+
Ubiquitin thioesterase OTUB1	OTUB1	57128000	88456000	1,60	1,21	0,28	10,644	0,3731749	0,3203114	0,4971269	0,4029404	0,3198492	0,2760797	-0,5374869	NaN	0,1908044	0,1329059	-0,02525047	0,2693923	3,93E-02	1,41	0,201804324	
Dihydrolipoyllysine-residue acetyltransferase component of pyruvate dehydrogenase complex, mitochondrial	DLAT	246340000	381440000	1,54	1,21	0,28	14,426	0,1851053	0,2146222	0,1035311	0,1809117	0,4713435	0,384713	0,2938414	0,2645965	0,3639	0,2924284	0,0729976	0,3500446	8,90E-06	5,05	0,264836282	+
Cytochrome b-c1 complex subunit 1, mitochondrial	UQCRC1	475840000	729560000	1,55	1,21	0,27	14,278	0,3986787	0,4274989	0,3703876	0,3423271	0,2429378	0,3799537	-0,09172513	-0,04660813	-0,01067203	-0,07289237	0,2246577	0,006908315	1,16E-02	1,94	0,180954347	+
Mitogen-activated protein kinase 14	MAPK14	37251000	52112000	1,50	1,21	0,27	13,192	0,3359405	0,1206174	-0,03701426	0,1755566	0,1001703	0,3649085	NaN	0,3163775	0,07052668	0,1749178	NaN	NaN	3,95E-03	2,40	0,18022234	+
Transcription factor A, mitochondrial	TFAM	30645000	49301000	1,99	1,21	0,27	24,094	1,267176	0,6877774	NaN	0,1851053	0,5918708	0,7336146	0,2121336	0,2939589	0,2002533	-0,03510605	0,3212354	0,1282935	3,97E-03	2,40	0,416937543	+
BRO1 domain-containing protein BROX	BROX	117840000	167870000	1,41	1,21	0,27	16,749	0,1971105	0,3186928	0,2382977	0,3278023	0,009633875	0,3895667	NaN	NaN	NaN	NaN	0,1885276	0,2331518	6,57E-04	3,18	0,237847925	+
Adenylate kinase isoenzyme 1	AK1	16198000	28318000	1,77	1,21	0,27	13,635	0,319618	0,3520805	0,3367404	0,398022	0,111432	0,6828429	0,2475629	0,2636354	0,2194628	0,2732175	0,3608699	0,2168583	8,26E-06	5,08	0,315195214	+
ATP synthase subunit alpha, mitochondrial	ATP5A1	3912700000	5762300000	1,43	1,20	0,27	8,7289	0,2689134	0,3029917	0,2277411	0,2667565	0,2984237	0,2983063	0,07833651	0,03885755	0,2401311	0,2458607	-0,09154058	-0,0023102	7,87E-04	3,10	0,181038986	+
WD repeat-containing protein 5	WDR5	16569000	23065000	1,69	1,20	0,27	17,038	NaN	0,07217448	0,03646811	-0,09475698	-0,07818285	0,02105333	NaN	NaN	0,2606279	0,2245343	-0,05685135	0,05797008	2,72E-01	0,57	0,049226335	
Tryptophan--tRNA ligase, cytoplasmic;T1-TrpRS;T2-TrpRS	WARS	784220000	1219600000	1,59	1,20	0,27	16,343	0,363115	0,261591	0,3382247	0,3596336	0,4324249	0,3384529	0,1354035	0,03843611	NaN	NaN	0,0878702	0,09815002	3,94E-04	3,40	0,245330201	+
Pyruvate dehydrogenase E1 component subunit alpha, somatic form, mitochondrial	PDHA1	74254000	114810000	1,55	1,20	0,27	9,4937	NaN	0,01720924	NaN	-0,1982784	0,3981315	0,2723816	0,04194384	NaN	0,3419858	0,06515872	NaN	NaN	1,45E-01	0,84	0,134076038	
Tubulin beta-6 chain	TUBB6	287430000	446480000	1,47	1,20	0,26	15,306	0,2856981	0,2440345	0,2484133	0,2813124	0,3589589	0,3286067	NaN	-0,045491	NaN	0,2941943	0,009347181	-0,03178392	3,12E-03	2,51	0,197329052	+
Retinol dehydrogenase 13	RDH13	21499000	33253000	1,52	1,20	0,26	8,3851	0,18954	0,1551016	0,359971	0,2007554	0,2152437	0,4636224	0,009920342	NaN	0,03506084	0,04628156	0,04725923	NaN	5,28E-03	2,28	0,172275599	+
Cathepsin D;Cathepsin D light chain;Cathepsin D heavy chain	CTSD	28516000	47676000	1,69	1,20	0,26	24,319	-0,01610345	0,3106892	-0,07378799	-0,0555763	0,188021	-0,1485288	NaN	NaN	0,5946441	0,4327456	0,455439	0,5469561	3,31E-02	1,48	0,223449848	
Lamina-associated polypeptide 2, isoform alpha;Thymopoietin;Thymopentin	TMPO	88706000	143840000	1,96	1,20	0,26	19,301	0,3448285	0,485736	0,03660879	0,01449814	-0,2121496	0,1645289	0,2509616	0,4822288	0,264116	-0,1190873	0,4715517	0,4800582	9,80E-03	2,01	0,221989974	+
Histone-arginine methyltransferase CARM1	CARM1	25625000	32187000	1,32	1,20	0,26	3,8043	0,07272322	0,2555611	NaN	NaN	NaN	-0,4399112	0,1950955	0,08270261	0,1947175	NaN	NaN	NaN	5,89E-01	0,23	0,060148125	
Bifunctional coenzyme A synthase;Phosphopantetheine adenylyltransferase;Dephospho-CoA kinase	COASY	26149000	33841000	1,52	1,20	0,26	10,956	0,3321638	NaN	NaN	0,4427578	NaN	0,3581712	NaN	NaN	NaN	NaN	-0,09157137	-0,4195843	4,92E-01	0,31	0,124387412	
Ragulator complex protein LAMTOR3	LAMTOR3	5453700	8762500	1,45	1,20	0,26	10,981	0,3774015	0,2591821	NaN	NaN	NaN	NaN	-0,08813206	NaN	0,4299641	NaN	0,2709474	NaN	5,07E-02	1,30	0,2498726	
Thymidylate kinase	DTYMK	36999000	58782000	1,54	1,20	0,26	13,872	0,4499575	0,3071954	0,1273692	0,2614706	0,2324152	0,3542265	-0,2518651	NaN	NaN	NaN	NaN	-0,01126808	5,43E-02	1,27	0,183687659	
Translin-associated protein X	TSNAX	43901000	73165000	1,50	1,20	0,26	11,484	0,2446436	0,3634514	0,1988712	0,2757223	0,3367404	0,3893466	0,2458607	0,04586229	0,2817873	-0,08640019	-0,07943225	-0,1330999	8,19E-03	2,09	0,173612781	+
Ras-related protein Rab-21	RAB21	41674000	62875000	1,69	1,19	0,26	9,946	0,4021764	0,3609822	NaN	0,2265085	0,249142	0,6291001	NaN	-0,3645335	0,05436236	0,09788039	NaN	0,1541943	5,98E-02	1,22	0,201090294	
Enolase-phosphatase E1	ENOPH1	14439000	20981000	1,54	1,19	0,25	14,578	0,3071954	0,206143	NaN	0,1825652	NaN	0,3309025	NaN	NaN	NaN	0,02630426	-0,1770659	NaN	1,21E-01	0,92	0,146007408	
55 kDa erythrocyte membrane protein	MPP1	196830000	301090000	1,43	1,19	0,25	16,751	0,2204538	0,3089433	0,261591	0,2251515	0,3872522	0,2492632	-0,1298918	0,08188502	-0,07501847	-0,1595085	-0,1578337	-0,03608202	1,18E-01	0,93	0,098017122	
Actin-related protein 2/3 complex subunit 2	ARPC2	24130000	34950000	1,40	1,19	0,25	7,7023	0,2389091	0,2532632	0,4709273	0,3469853	0,2851063	0,3096419	NaN	NaN	0,1154328	0,1569143	NaN	NaN	2,22E-04	3,65	0,272147522	+
HD domain-containing protein 2	HDDC2	39553000	58548000	1,44	1,19	0,25	24,563	0,3234277	0,3339961	0,3503841	0,292075	0,5936884	0,3925373	NaN	-0,2756292	NaN	NaN	-0,2575798	NaN	8,88E-02	1,05	0,219112433	
Phospholipase A-2-activating protein	PLAA	14374000	20987000	1,59	1,19	0,25	12,866	-0,02766022	0,3483458	0,197488	0,1584662	0,1810389	0,5212518	0,08651205	0,04753833	0,156526	0,2904245	0	0,442227	1,95E-03	2,71	0,200179863	+
Vinculin	VCL	2083200000	2890500000	1,46	1,19	0,25	10,378	0,2579761	0,2671163	0,3034593	0,3319345	0,3327366	0,351063	0,1749178	0,1758121	0,1292171	0,1347467	0,2132539	0,1976139	4,60E-07	6,34	0,239153922	+
Mitochondrial import receptor subunit TOM34	TOMM34	28129000	38239000	1,26	1,19	0,25	15,177	0,1646576	0,187261	0,1248574	0,2161133	0,1978653	0,2157407	NaN	-0,4074784	-0,3052	-0,130397	-0,285372	-0,3891866	6,50E-01	0,19	-0,03737624	
Phenylalanine--tRNA ligase beta subunit	FARSB	297680000	409200000	1,54	1,19	0,25	12,358	0,3191554	0,2582174	0,08038475	0,2167342	0,2459823	0,1598875	0,2729787	0,2504765	0,3389092	0,2035144	0,2953704	0,4396763	9,20E-07	6,04	0,256773928	+
Dolichyl-diphosphooligosaccharide--protein glycosyltransferase subunit 2	RPN2	430220000	585470000	1,49	1,19	0,25	10,164	0,2422062	0,2154923	0,246712	0,1897929	0,3379965	0,2594232	0,2456174	0,3189242	0,3243498	0,2936059	0,1339581	0,1645289	3,94E-08	7,40	0,24771729	+
DNA replication licensing factor MCM5	MCM5	364310000	520850000	1,66	1,19	0,25	17,592	0,04600206	0,1174291	0,1713349	0,1624679	0,07696952	0,2332746	0,3716146	0,2816686	0,2617113	0,2080177	0,3698294	0,3255016	2,30E-05	4,64	0,218818431	+
Electron transfer flavoprotein subunit alpha, mitochondrial	ETFA	198870000	343350000	1,67	1,19	0,25	11,457	0,3330801	0,3418719	0,3397075	0,4309275	0,3764015	0,3381105	0,1420866	0,1811662	0,1641426	0,1489992	0,1335635	0,1436546	7,23E-06	5,14	0,256142655	+

Sorcin	SRI	35287000	56489000	1,65	1,19	0,25	6,0529	0,2143735	0,1561377	0,2075181	0,3419858	0,4148932	0,3883549	0,2141248	0,3474389	0,2852247	0,2106382	0,2875905	0,2823805	1,25E-07	6,90	0,279221732	+
Bifunctional glutamate/proline--tRNA ligase;Glutamate--tRNA ligase;Proline--tRNA ligase	EPRS	1112900000	1508600000	1,38	1,19	0,24	15,845	0,3189242	0,315798	0,2161133	0,2543522	0,1955996	0,3637879	0,06115411	0,07983892	0,07491644	0,06653696	0,1579492	-0,1214708	1,79E-03	2,75	0,165291669	+
DNA-(apurinic or apyrimidinic site) lyase;DNA-(apurinic or apyrimidinic site) lyase, mitochondrial	APEX1	42540000	74991000	1,70	1,18	0,24	21,057	0,09707135	0,1674864	0,2702299	0,2786984	0,1767058	0,1309309	0,2735755	0,2237931	0,1311943	0,2211965	NaN	NaN	5,73E-06	5,24	0,197088225	+
UPF0553 protein C9orf64	C9orf64	5253600	8600300	1,66	1,18	0,24	4,7652	NaN	0,363115	NaN	NaN	NaN	0,3090597	NaN	NaN	0,1491292	0,2273714	NaN	NaN	1,13E-02	1,95	0,262168825	+
Rab GDP dissociation inhibitor alpha	GDI1	36953000	62815000	1,38	1,18	0,24	11,658	0,05990899	0,3010022	NaN	NaN	0,3269975	0,30381	NaN	NaN	NaN	NaN	0,4652954	0,4138107	2,80E-03	2,55	0,311804137	+
Heterogeneous nuclear ribonucleoprotein U-like protein 2	HNRNPUL2	78883000	100100000	1,38	1,18	0,24	15,996	-0,1543453	0,09922788	0,03365203	0,3420996	0,1152996	0,1553607	0,1331689	0,445409	-0,2577868	-0,1847197	0,1035311	0,54962	1,32E-01	0,88	0,115043082	
Alpha/beta hydrolase domain-containing protein 14B	ABHD14B	135290000	201460000	1,56	1,18	0,24	7,145	0,2251515	0,2541102	0,2418402	0,3377682	0,2741722	0,1973622	-0,1741167	-0,1775554	-0,2626074	-0,1276361	-0,0261169	-0,2421574	5,29E-01	0,28	0,043351217	
Ribonuclease UK114	HRSP12	53409000	75778000	1,52	1,18	0,24	9,8038	0,227125	0,2270017	0,144177	0,1769609	0,1346153	0,3481191	NaN	0,3919876	0,2748878	NaN	0,4349884	0,346191	2,01E-05	4,70	0,270605372	+
Stathmin	STMN1	476550000	752840000	1,62	1,18	0,24	15,016	0,05755419	0,3672592	0,06460693	0,2144979	0,2551986	0,2284801	0,12618	0,2168583	0,2417182	0,2785796	0,10916	0,06942715	4,31E-05	4,37	0,185793345	+
Structural maintenance of chromosomes protein 3	SMC3	29079000	47773000	1,73	1,18	0,24	27,351	NaN	0,3310172	NaN	0,1840899	NaN	1,494467	0,6194599	0,8273312	0,2082676	0,2412301	0,01178193	0,4508021	1,21E-02	1,92	0,485382971	+
Reticulon-4	RTN4	243790000	364420000	1,51	1,18	0,24	18,3	0,3298698	0,1439158	0,1573025	0,0439044	0,2872358	0,1444379	0,2699907	0,06722574	0,206018	0,2267551	NaN	0,1471764	4,65E-05	4,33	0,183984743	+
Putative RNA-binding protein Luc7-like 2	LUC7L2	346520000	526040000	1,59	1,18	0,24	22,494	0,1815478	0,1977397	-0,004450373	0,1805297	0,04739887	0,144699	0,351063	0,3056787	0,6885833	0,5750218	0,2380531	0,3179986	7,08E-04	3,15	0,268655259	+
Isocitrate dehydrogenase [NAD] subunit beta, mitochondrial	IDH3B	68177000	97468000	1,52	1,18	0,24	9,1304	0,4146767	0,2509616	0,2292187	0,1416943	0,2484133	0,2244108	NaN	NaN	0,02133754	0,07601179	NaN	NaN	2,26E-03	2,65	0,200840604	+
Serine/threonine-protein kinase OSR1	OXSR1	61470000	89113000	1,30	1,18	0,24	7,4229	0,1044708	0,1230039	0,1997508	0,232538	0,261952	0,2927818	NaN	NaN	NaN	NaN	0,6042612	0,1742785	2,87E-03	2,54	0,249129629	+
Flavin reductase (NADPH)	BLVRB	1108100000	1878200000	1,76	1,18	0,24	13,866	0,3805078	0,4155423	0,458172	0,5521803	0,3802861	0,4455148	-0,02594065	-0,0391473	0,0311129	0,01020692	0,008343471	-0,02445785	9,18E-03	2,04	0,216026739	+
ADP-ribosylation factor 5	ARF5	6732800	9461900	1,40	1,18	0,24	5,0832	0,2869995	0,2705888	NaN	0,2013829	NaN	0,1318529	0,01963077	NaN	0,167358	NaN	NaN	NaN	6,54E-03	2,18	0,179635449	+
Casein kinase II subunit alpha	CSNK2A2	18097000	29902000	1,47	1,18	0,23	12,86	0,2937236	0,3808402	NaN	0,3828332	0,0650208	0,2923105	0,2123825	0,2821432	0,09545191	0,3822799	NaN	NaN	1,60E-04	3,79	0,265220663	+
V-type proton ATPase subunit G 1	ATP6V1G1	31497000	48301000	1,42	1,18	0,23	14,523	0,1773438	0,2660369	0,1636276	0,07038926	0,168257	0,1935825	-0,008087322	NaN	-0,004146521	NaN	0,121148	0,04292445	2,52E-03	2,60	0,119107561	+
Mitochondrial fission 1 protein	FIS1	13383000	21060000	1,60	1,17	0,23	10,141	0,4227712	0,329181	NaN	NaN	NaN	NaN	0,0116388	0,1994997	-0,195963	0,1791288	NaN	0,1515991	8,75E-02	1,06	0,156836524	
Phosphoribosyl pyrophosphate synthase-associated protein 1	PRPSAP1	31039000	36436000	1,60	1,17	0,23	14,287	0,4101239	0,4032677	0,4135941	0,1875144	0,6339427	0,4323181	-0,2652406	-0,1720184	NaN	-0,1055353	NaN	NaN	7,83E-02	1,11	0,215329625	
Protein diaphanous homolog 1	DIAPH1	455240000	591630000	1,28	1,17	0,23	15,045	0,2974849	0,2644764	0,19131	0,2178511	0,2639958	0,3373114	0,09328973	-0,09933989	0,0182068	-0,09589748	-0,1840148	0,3680419	2,63E-02	1,58	0,139392985	+
Transmembrane emp24 domain-containing protein 10	TMED10	159660000	255770000	1,58	1,17	0,23	14,717	0,1031283	0,2760797	0,1977397	0,160533	0,3024067	0,2433034	0,2081427	0,2274946	0,186247	0,2648367	0,2082676	0,1943392	2,77E-08	7,56	0,214376546	+
Signal peptidase complex catalytic subunit SEC11A	SEC11A	41592000	37750000	1,32	1,17	0,23	12,655	0,4404207	0,1023222	NaN	0,07778991	NaN	NaN	-0,02749851	-0,07286207	NaN	NaN	NaN	NaN	3,13E-01	0,50	0,104034458	
NADH-ubiquinone oxidoreductase 75 kDa subunit, mitochondrial	NDUFS1	134480000	208390000	1,31	1,17	0,23	23,477	-0,02560283	-0,07002721	0,3934163	0,2797684	0,1386835	0,2025117	0,4111008	0,3248107	0,3688243	0,294312	0,2650768	0,2998307	2,25E-04	3,65	0,240225418	+
Sec1 family domain-containing protein 1	SCFD1	75168000	88576000	1,41	1,17	0,23	5,8315	NaN	0,1924466	0,2066433	0,1830736	0,09531686	0,2335199	-0,3240757	-0,03346621	0,4529117	-0,1058148	NaN	NaN	2,21E-01	0,66	0,100061705	
26S proteasome non-ATPase regulatory subunit 7	PSMD7	631120000	879820000	1,52	1,17	0,23	10,021	0,3352546	0,2951353	0,2928995	0,143524	0,2329063	0,2956054	NaN	-0,1742469	0,05380643	0,003746074	NaN	NaN	2,12E-02	1,67	0,164292292	+
Alcohol dehydrogenase class-3	ADH5	52774000	73342000	1,47	1,17	0,23	12,284	-0,0296175	0,1058121	0,001874253	0,1347467	0,3382247	0,2363395	0,04893365	-0,06774226	0,3481191	0,08052122	0,02941749	NaN	2,56E-02	1,59	0,111511724	+
26S proteasome non-ATPase regulatory subunit 1	PSMD1	500510000	668870000	1,32	1,17	0,23	10,507	0,2295879	0,2202061	0,2126316	0,2817873	0,28889	0,3191554	0,2368294	0,04963072	0,1138339	0,06873958	0,1001703	0,2216915	1,17E-05	4,93	0,195262814	+
Large proline-rich protein BAG6	BAG6	159940000	203270000	1,25	1,17	0,23	25,275	0,222681	0,2535053	0,06653696	0,2042659	0,32112	0,1307991	NaN	0,1615652	NaN	NaN	-0,01864412	0,3033423	1,11E-03	2,95	0,182796859	+
Trifunctional enzyme subunit alpha, mitochondrial;Long-chain enoyl-CoA hydratase;Long chain 3-hydroxyacyl-CoA dehydrogenase	HADHA	576810000	837060000	1,52	1,17	0,22	8,1816	0,1786191	0,2862897	0,2454957	0,2968979	0,2446436	0,2885358	0,1541943	0,1950955	0,1501697	0,212009	0,2006298	0,1025909	1,14E-07	6,94	0,212930912	+
SWI/SNF complex subunit SMARCC1	SMARCC1	139840000	108440000	1,29	1,17	0,22	14,675	NaN	0,4097983	0,02389408	0,1986199	-0,1752077	0,007339063	-2,387223	-1,768371	0,1159654	-0,2180612	NaN	NaN	2,29E-01	0,64	-0,421471799	
Aspartate--tRNA ligase, cytoplasmic	DARS	1023400000	1500500000	1,43	1,17	0,22	7,6523	0,09004036	0,2130051	0,1723594	0,1749178	0,2418402	0,1910572	0,347212	0,3083608	0,4900566	0,2499914	0,205017	0,206018	5,55E-06	5,26	0,240823001	+
NAD(P)H dehydrogenase [quinone] 1	NQO1	57061000	88468000	1,56	1,17	0,22	9,288	0,2823805	0,3486856	0,2335199	0,2076431	0,2802438	0,2279875	NaN	NaN	NaN	0,6298462	NaN	0,7292699	1,23E-03	2,91	0,367447056	+
DAZ-associated protein 1	DAZAP1	91718000	108620000	1,61	1,17	0,22	14,391	0,2358496	NaN	0,2446436	0,3468719	-0,03225588	-0,05120486	0,4565959	0,5668638	0,5842891	0,3813941	NaN	0,4852206	1,44E-03	2,84	0,321826793	+
Ribose-phosphate pyrophosphokinase 1	PRPS1	55685000	84503000	1,47	1,17	0,22	18,532	0,1987455	0,1003049	0,1339581	0,2613503	0,3130136	0,2580967	NaN	0,03927886	-0,02012095	-0,02365107	-0,1001748	-0,1370131	7,25E-02	1,14	0,093071657	
Heterogeneous nuclear ribonucleoprotein H3	HNRNPH3	57429000	90988000	1,99	1,17	0,22	14,764	-0,5547818	-0,5139962	0,2623128	0,1013812	-0,379958	-0,4538933	0,446468	0,4695734	0,1253867	0,169027	0,7135198	0,6059683	5,41E-01	0,27	0,082583995	
MICOS complex subunit MIC60	IMMT	645910000	964320000	1,51	1,16	0,22	14,882	0,2560445	0,2574934	0,102994	0,2289726	0,3101074	0,3469853	0,1444379	0,23842	0,1286893	0,185486	0,1515991	0,1642714	1,10E-06	5,96	0,209625088	+
Nuclear transport factor 2	NUTF2	33043000	52309000	1,58	1,16	0,22	8,8113	0,2168583	0,2190911	0,247806	NaN	NaN	NaN	0,1216786	0,2561654	0,2091414	0,1708224	0,2016338	0,08351974	8,05E-06	5,09	0,191857422	+
Superoxide dismutase [Cu-Zn]	SOD1	197250000	337050000	1,70	1,16	0,22	11,213	0,2881813	0,3986787	0,2579761	0,2501126	0,3718376	0,3017047	0,165944	0,1437852	0,1835818	0,2457391	0,1017845	0,2103888	1,26E-06	5,90	0,243309538	+
Retinol dehydrogenase 11	RDH11	23649000	36686000	1,49	1,16	0,22	15,8	0,3755121	0,2234226	0,4714476	0,05547326	0,3672592	0,2924284	NaN	NaN	-0,3371747	-0,003336449	-0,245027	NaN	1,98E-01	0,70	0,133333882	
Electron transfer flavoprotein subunit beta	ETFB	343400000	519770000	1,56	1,16	0,22	8,7959	0,2801249	0,2439128	0,1887806	0,2595437	0,2189671	0,2462257	0,168642	0,2068932	0,1887806	0,1697967	0,2378085	0,2292187	2,77E-10	9,56	0,219891217	+
Probable tRNA N6-adenosine threonylcarbamoyltransferase	OSGEP	14812000	21724000	1,49	1,16	0,22	2,7562	0,203765	0,02998291	0,2088917	-0,04639952	0,1281614	0,3374256	NaN	NaN	0,6683915	0,3163775	NaN	NaN	2,09E-02	1,68	0,230824534	+
Pachytene checkpoint protein 2 homolog	TRIP13	46174000	68295000	1,49	1,16	0,22	15,487	0,2420842	0,232538	NaN	0,08691968	0,2498701	0,1315895	NaN	NaN	NaN	NaN	-0,006434354	0,1541943	4,81E-03	2,32	0,155823064	+
E3 ubiquitin-protein ligase HUWE1	HUWE1	258670000	336500000	1,27	1,16	0,21	15,757	0,2905423	0,1777266	0,144177	0,1219437	0,351063	0,2892443	0,07189995	0,2218153	0,2352368	0,2451305	-0,1074614	0,2420842	2,17E-04	3,66	0,190283516	+
Mitochondrial carrier homolog 2	MTCH2	369310000	481350000	1,36	1,16	0,21	16,441	0,2758415	0,1096949	0,2065182	0,2278643	0,2937236	-0,009437132	-0,1470263	-0,2365884	-0,05015878	0,04320455	-0,1877236	0,000144286	4,21E-01	0,38	0,043838091	
Squamous cell carcinoma antigen recognized by T-cells 3	SART3	127060000	152530000	1,27	1,16	0,21	24,895	-0,1151442	0,2404974	0,3391374	0,1909309	0,0911243	0,008486926	0,0692898	NaN	NaN	NaN	0,1286893	0,2672362	2,01E-02	1,70	0,135583107	+
Asparagine--tRNA ligase, cytoplasmic	NARS	391090000	564450000	1,51	1,16	0,21	13,784	0,2329063	0,1176951	0,158854	0,205017	0,3117356	0,1760674	0,08256635	0,1047392	NaN	NaN	0,1968587	0,09761087	3,90E-05	4,41	0,168405049	+
Ras GTPase-activating-like protein IQGAP1	IQGAP1	431810000	536770000	1,29	1,16	0,21	23,913	0,296428	0,1502997	0,1703097	0,1654296	0,311038	0,2503552	0,2623128	0,1645289	0,10742	0,2719037	0,2241638	0,1264442	4,87E-07	6,31	0,208386139	+
Heat shock 70 kDa protein 1B;Heat shock 70 kDa protein 1A	HSPA1B;HSPA1A	3507600000	5082600000	1,43	1,16	0,21	13,74	0,3671474	0,349705	0,3238889	0,2761988	0,2951353	0,2758415	-0,1352057	-0,07949331	-0,1629609	-0,2099954	-0,1601694	-0,1355543	2,60E-01	0,58	0,083711473	
Endoplasmic reticulum resident protein 29	ERP29	345760000	532750000	1,58	1,16	0,21	5,9772	0,2365845	0,2321696	0,1473066	0,2380531	0,2106382	0,2878269	0,07642223	0,04697992	0,1845977	0,08174868	0,1843437	0,4135941	3,52E-05	4,45	0,195022099	+
Eukaryotic translation initiation factor 3 subunit J	EIF3J	78768000	112760000	1,45	1,16	0,21	12,224	0,3025237	0,2407417	0,3791774	NaN	0,1987455	NaN	-0,08623166	-0,116958	0,05283319	-0,04908374	-0,06414803	-0,000476127	1,65E-01	0,78	0,085712401	
Aspartate aminotransferase, cytoplasmic	GOT1	554810000	820110000	1,46	1,16	0,21	12,588	0,2914856	0,3294107	0,2988929	0,3015876	0,1606621	0,3029917	0,02828617	-0,01417899	0,03998062	0,05255495	NaN	NaN	2,86E-03	2,54	0,179167331	+
Threonine--tRNA ligase, cytoplasmic	TARS	1064100000	1424300000	1,55	1,16	0,21	18,589	0,2579761	0,345737	0,3294107	0,2838032	0,400975	0,3287216	0,06915226	0,1138339	0,05990899	0,09518178	0,07313477	0,1781091	1,20E-04	3,92	0,211328694	+
Cytosol aminopeptidase	LAP3	345190000	485910000	1,49	1,15	0,21	10,719	0,2068932	0,1812935	0,2083924	0,09058251	0,3008851	0,1556197	0,1146336	0,08420031	NaN	NaN	0,2617113	0,122474	3,67E-05	4,44	0,172668555	+
Stress-70 protein, mitochondrial	HSPA9	3331300000	4945500000	1,57	1,15	0,21	6,832	0,1593708	0,1585955	0,2734562	0,190678	0,1515991	0,1482182	0,2599051	0,2431815	0,1935825	0,188401	0,2693923	0,2446436	1,31E-08	7,88	0,206751981	+
Annexin A2;Putative annexin A2-like protein	ANXA2;ANXA2P2	703140000	1030800000	1,46	1,15	0,21	11,307	0,2450088	0,1188912	0,2516888	0,2121336	0,242572	0,2635152	0,09410089	0,1493894	0,1072862	0,1637564	0,1875144	0,1200864	7,33E-07	6,13	0,179661946	+
Minor histocompatibility antigen H13	HM13	18049000	23309000	1,36	1,15	0,21	5,2204	NaN	NaN	NaN	0,07970238	0,4139189	0,2055176	NaN	NaN	0,2121336	0,3159139	NaN	NaN	1,21E-02	1,92	0,245437296	+
Aspartate aminotransferase, mitochondrial	GOT2	1089800000	1673500000	1,54	1,15	0,21	13,107	0,4020673	0,444667	0,3693827	0,3708338	0,3397075	0,3504972	-0,06362023	0,02304246	-0,05012895	-0,05923934	NaN	NaN	1,32E-02	1,88	0,212720952	+
Perilipin-3	PLIN3	746060000	1096500000	1,48	1,15	0,21	14,237	0,2733369	0,2726204	0,3226204	0,2337653	0,2798873	0,3622174	-0,1649814	0,02828617	-0,05595116	-0,04361613	0,1932041	0,07860982	1,28E-02	1,89	0,148333253	+
Splicing factor 3A subunit 1	SF3A1	298390000	385930000	1,27	1,15	0,20	23,95	0,2525368	0,1641426	0,2032638	0,1796384	0,1476974	0,0244615	0,4440309	0,3042774	0,3955016	-0,1308867	0,2058929	0,2568899	7,62E-04	3,12	0,203953871	+
NADH dehydrogenase [ubiquinone] 1 beta subcomplex subunit 9	NDUFB9	65553000	101130000	1,48	1,15	0,20	20,806	0,0494913	0,08515262	-0,05544136	0,1052758	-0,0713151	-0,02252223	0,4430762	0,3731749	0,2161133	0,2593026	0,163241	0,2197106	9,82E-03	2,01	0,147104984	+
Very-long-chain 3-oxoacyl-CoA reductase	HSD17B12	32363000	50573000	1,55	1,15	0,20	8,25	0,2063932	0,1331689	0,4127274	0,29125	0,2733369	0,3802861	0,03153631	0,1443075	-0,001818917	0,2828548	0,1363226	0,2422062	1,33E-04	3,88	0,211047585	+
Destrin	DSTN	101760000	148520000	1,43	1,15	0,20	12,209	0,2514464	0,2330291	0,2535053	0,3619929	0,3787335	0,3198492	-0,1786816	-0,1687551	-0,2636635	-0,2543238	-0,2623998	-0,134477	5,81E-01	0,24	0,044687962	
Acyl-coenzyme A synthetase ACSM3, mitochondrial	ACSM3	54060000	72458000	1,47	1,15	0,20	29,901	-0,2953227	NaN	0,009777201	NaN	0,3939653	0,09693644	NaN	NaN	NaN	NaN	0,5585616	-0,3193696	6,32E-01	0,20	0,074091353	
NAD kinase 2, mitochondrial	NADK2	73517000	82127000	1,38	1,15	0,20	5,476	0,2918393	0,2116352	0,2648367	0,04208399	0,08746284	0,1989969	NaN	NaN	0,02431962	0,2397646	NaN	NaN	2,40E-03	2,62	0,170117405	+
Proline synthase co-transcribed bacterial homolog protein	PROSC	13384000	21575000	1,60	1,15	0,20	14,93	0,3375398	0,167358	0,5861161	0,3108054	0,3266523	0,2359721	NaN	NaN	0,3449421	0,03365203	NaN	-0,2481079	1,75E-02	1,76	0,232770006	+
Methionine--tRNA ligase, cytoplasmic	MARS	539730000	731130000	1,47	1,15	0,20	9,5855	0,2715452	0,2063932	0,1633699	0,1998764	0,3145225	0,2363395	0,01292609	-0,01652655	-0,01214072	0,08024827	0,07778991	0,08243007	1,64E-03	2,78	0,134731156	+
NADH dehydrogenase [ubiquinone] iron-sulfur protein 5	NDUFS5	10799000	18635000	1,62	1,15	0,20	5,7375	0,1527676	0,4075159	NaN	NaN	0,1505597	NaN	NaN	0,2578555	-0,02485411	0,3592962	NaN	-0,2617769	1,40E-01	0,85	0,148766281	
Oligoribonuclease, mitochondrial	REXO2	45118000	62175000	1,41	1,15	0,20	15,715	0,2380531	0,3765127	0,2734562	0,2356045	0,166587	NaN	NaN	-0,3613978	-0,5520929	-0,4235843	NaN	NaN	9,66E-01	0,02	-0,005857699	
DnaJ homolog subfamily C member 9	DNAJC9	55815000	75363000	1,55	1,15	0,20	17,298	0,14496	0,2759607	0,3204269	0,205017	0,3106892	0,188021	NaN	NaN	0,1158324	0,1402552	0,1829465	0,05366741	5,88E-05	4,23	0,193777628	+
m7GpppX diphosphatase	DCPS	22371000	34430000	1,38	1,15	0,20	17,932	0,03703076	0,2797684	0,06143053	0,2936059	0,2626737	0,3893466	NaN	0,3309025	0,2468337	0,0734089	NaN	NaN	9,15E-04	3,04	0,219444549	+
28S ribosomal protein S35, mitochondrial	MRPS35	22218000	27404000	1,49	1,15	0,20	16,883	NaN	NaN	NaN	0,2685541	NaN	-0,2193372	NaN	NaN	0,3717261	0,2657969	NaN	NaN	2,86E-01	0,54	0,17168498	
Importin-9	IPO9	127380000	156690000	1,26	1,15	0,20	13,631	0,1583371	0,1578198	-0,07239174	0,05935536	0,05477901	0,1736392	0,08976937	0,1386835	0,1248574	0,2208253	0,05838585	0,08501676	6,28E-04	3,20	0,104089741	+
Phosphate carrier protein, mitochondrial	SLC25A3	549200000	795950000	1,50	1,14	0,19	13,99	0,2392758	0,2447654	0,2143735	0,1556197	0,3284918	0,2593026	0,2625534	0,0692898	0,07970238	0,10742	0,1043367	0,1382902	1,23E-05	4,91	0,183618447	+
Saccharopine dehydrogenase-like oxidoreductase	SCCPDH	94308000	129110000	1,36	1,14	0,19	19,279	0,1437852	0,1625968	0,1624679	0,2531421	0,2293418	0,0745054	NaN	-0,1513139	-0,4648031	-0,2432158	NaN	NaN	8,28E-01	0,08	0,018500711	
Histone-binding protein RBBP7	RBBP7	85356000	131930000	1,64	1,14	0,19	14,029	0,3642362	0,4346683	0,4202937	0,4353086	0,3737317	NaN	NaN	NaN	NaN	NaN	-0,1209686	-0,001948933	2,11E-02	1,68	0,272188699	+
Pyruvate dehydrogenase E1 component subunit beta, mitochondrial	PDHB	47239000	70636000	1,69	1,14	0,19	24,803	0,1285573	0,09342498	0,1335635	0,00302647	0,41186	0,398022	NaN	NaN	0,1897929	0,1932041	0,2678354	0,1828194	8,21E-04	3,09	0,200210613	+
Bcl-2-like protein 13	BCL2L13	22508000	33044000	1,58	1,14	0,19	23,095	0,4027222	NaN	0,4699901	NaN	0,1821838	NaN	NaN	0,08324736	-0,136236	0,08052122	NaN	NaN	1,07E-01	0,97	0,180404798	
Low molecular weight phosphotyrosine protein [illegible]	ACP1	117100000	189230000	1,55	1,14	0,19	9,2344	0,2678354	0,1712069	0,01306909	0,1107641	0,05935536	0,1315895	0,2726204	0,2723816	0,2801249	0,2584586	0,3345683	0,7680784	1,07E-03	2,97	0,245004383	+
Probable ATP-dependent RNA helicase DDX23	DDX23	19670000	24691000	1,52	1,14	0,18	19,386	NaN	0,07354603	NaN	0,4318903	NaN	NaN	0,2385423	0,103397	-0,3032049	-0,2012609	0,3075451	-0,159428	5,29E-01	0,28	0,061378371	
Ubiquitin carboxyl-terminal hydrolase isozyme L5	UCHL5	99509000	135950000	1,42	1,14	0,18	14,21	0,04376442	0,1465249	0,07162537	0,1139672	0,2100148	0,1940871	NaN	0,006333955	0,2027625	0,2131294	-0,03351048	0,1389455	1,05E-03	2,98	0,118876784	+
Talin-1	TLN1	3203400000	4061200000	1,25	1,13	0,18	13,246	0,09922788	0,02800333	0,07368314	0,095722	0,1579492	0,1096949	0,3341105	0,3581712	0,4042492	0,4198625	0,3498182	0,3940751	2,31E-04	3,64	0,235380592	+
Lamin-B receptor	LBR	205980000	282870000	1,37	1,13	0,18	17,654	0,2068932	0,1851053	0,04040144	0,1718473	0,3912178	0,357946	0,1194226	0,1208827	0,06805168	0,1416943	0,3427823	0,09450638	1,81E-04	3,74	0,186729252	+
U6 snRNA-associated Sm-like protein LSm4	LSM4	20070000	27866000	1,41	1,13	0,18	8,8088	0,2215678	0,244887	NaN	0,249142	0,1346153	NaN	0,5181323	NaN	NaN	NaN	0,4065367	NaN	3,53E-03	2,45	0,295813493	+
DNA damage-binding protein 1	DDB1	446320000	559300000	1,23	1,13	0,18	14,265	0,1894135	0,144699	0,09976643	0,1875144	0,08202134	0,2580967	0,07436837	0,09369545	0,2859348	0,1482182	0,1934563	0,2322924	5,59E-06	5,25	0,165789748	+
Leucine--tRNA ligase, cytoplasmic	LARS	1055100000	1460800000	1,37	1,13	0,18	12,801	0,2561654	0,2458607	0,2716648	0,2991274	0,2904245	0,27453	-0,02384173	-0,1213453	-0,01283923	0,01464098	-0,127778	-0,188973	1,03E-01	0,99	0,098136367	
Alpha-adducin	ADD1	76542000	89862000	1,18	1,13	0,18	13,944	0,01535482	0,1561377	0,1282935	0,07751644	-0,1473778	0,1741506	NaN	NaN	NaN	NaN	-0,1426877	-0,2031199	8,96E-01	0,05	0,007283454	
Lactoylglutathione lyase	GLO1	622070000	1121800000	1,77	1,13	0,18	10,789	0,1798932	0,2600257	0,1763228	0,2880631	0,295723	0,3251561	0,2105135	0,18954	0,1779815	0,1713349	0,1786191	0,1509495	6,08E-08	7,22	0,217010191	+
Platelet-activating factor acetylhydrolase IB subunit beta	PAFAH1B2	74875000	107790000	1,52	1,13	0,18	4,4818	0,1553607	0,1290852	NaN	0,08283886	0,2881813	0,1772162	NaN	0,06170706	0,2102642	0,230572	NaN	NaN	4,41E-04	3,36	0,16690319	+
Malate dehydrogenase, mitochondrial	MDH2	2512000000	3827400000	1,57	1,13	0,18	11,665	0,2611096	0,2313095	0,3494785	0,3311319	0,3357119	0,3047447	0,05561211	0,06129241	0,05227683	0,07860982	0,08038475	0,07382025	3,83E-04	3,42	0,184623519	+
14-3-3 protein eta	YWHAH	293500000	406150000	1,52	1,13	0,18	15,018	0,1609202	0,1126334	0,1875144	0,1518587	0,215368	0,1357976	-0,06419326	-0,1463396	-0,001342347	-0,0132323	-0,09354039	-0,0692097	2,12E-01	0,67	0,048019566	
Dynamin-1-like protein	DNM1L	131080000	177060000	1,37	1,13	0,18	17,613	0,2444	0,2197106	0,2370741	0,2027625	0,0439044	0,2505978	0,2657969	-0,1698745	NaN	NaN	-0,03662948	0,07162537	1,99E-02	1,70	0,132936774	+
Adenylyl cyclase-associated protein 1	CAP1	651470000	848160000	1,52	1,13	0,17	9,8005	-0,04312555	0,05352838	0,105678	0,1397313	0,1388144	0,1584662	0,2403754	0,3098746	0,3068455	0,2823805	0,4676968	0,3958306	3,95E-04	3,40	0,213008029	+
Stomatin-like protein 2, mitochondrial	STOML2	564380000	755210000	1,46	1,13	0,17	9,2546	0,1764505	0,2661569	0,1723594	0,13711	0,0880058	0,2228046	0,05769283	0,06046242	0,04404436	0,1580785	0,262794	0,2235462	3,01E-05	4,52	0,155792115	+

Tumor protein D54	TPD52L2	116830000	172860000	1,73	1,13	0,17	19,567	0,2261386	0,144699	0,1212807	0,1596292	0,2253984	0,1723594	0,1843437	0,1629835	0,07983892	0,1777266	0,07751644	0,181675	1,72E-07	6,77	0,159465788	+
Annexin A11	ANXA11	257750000	367410000	1,38	1,13	0,17	9,1498	0,2501126	0,1428708	0,2832106	0,2716648	0,2657969	0,2326607	0,2744107	0,120352	NaN	NaN	0,1139672	0,1277653	7,73E-06	5,11	0,208281159	+
Proteasome subunit alpha type-1	PSMA1	236410000	357410000	1,54	1,13	0,17	8,7017	0,2131294	0,1450905	0,2126316	0,1463945	0,2699907	0,215368	-0,02069169	0,1453513	0,1747899	0,13711	0,2697513	0,1937087	7,49E-06	5,13	0,175218693	+
Cancer-related nucleoside-triphosphatase	NTPCR	41672000	64991000	1,65	1,12	0,17	9,5818	0,2539892	0,2402533	0,2063932	0,0610158	0,1723594	0,1314578	0,2132539	0,3781787	0,3126652	0,3272275	0,008630365	0,279293	2,74E-05	4,56	0,215393118	+
Prostaglandin E synthase 2;Prostaglandin E synthase 2 truncated form	PTGES2	25329000	34262000	1,46	1,12	0,17	9,9256	0,06032421	-0,1434684	0,06584801	0,2343786	0,4059924	0,1935825	NaN	NaN	0,1582078	0,2131294	0,1406477	NaN	1,83E-02	1,74	0,14762693	+
Proteasome assembly chaperone 3	PSMG3	12596000	19575000	1,46	1,12	0,17	7,5406	0,2342561	0,1962293	-0,03695507	NaN	0,2146222	NaN	NaN	0,1483484	0,1514692	-0,008972576	0,1004395	0,1344838	3,91E-03	2,41	0,12599122	+
Translation initiation factor eIF-2B subunit delta	EIF2B4	89030000	127890000	1,35	1,12	0,17	8,997	0,1916889	0,1910572	0,1593708	0,1432628	0,4160831	0,1199537	NaN	NaN	NaN	0,4198625	NaN	-0,1244087	1,76E-02	1,76	0,189608802	+
14-3-3 protein epsilon	YWHAE	1383500000	2070900000	1,52	1,12	0,17	9,3068	0,1606621	0,1463945	0,2408638	0,292075	0,1938348	0,270828	0,07080147	0,01720924	0,03026536	0,06322686	0,1235338	0,04432424	4,13E-04	3,38	0,137834936	+
Aminoacyl tRNA synthase complex-interacting multifunctional protein 2	AIMP2	81344000	116800000	1,39	1,12	0,17	13,846	0,1649149	0,169925	0,08814155	0,09922788	0,3004166	0,3317052	0,3054453	0,258338	0,06584801	0,3154503	0,1736392	NaN	4,13E-05	4,38	0,206641091	+
NudC domain-containing protein 2	NUDCD2	39398000	55670000	1,55	1,12	0,17	11,782	0,165944	0,1704378	0,1160985	0,2081427	0,1952215	0,2025117	0,06267433	0,06750099	0,268075	-0,07711712	0,1311943	-0,2519682	2,81E-02	1,55	0,104892965	+
Mitochondrial import inner membrane translocase subunit TIM44	TIMM44	147870000	207570000	1,40	1,12	0,17	12,027	0,1688987	0,2851063	0,2632748	0,21661	0,1198209	0,1561377	0,3617683	0,2970153	0,0353423	0,04865466	0,2429378	0,2794119	2,23E-05	4,65	0,20624823	+
DNA replication licensing factor MCM3	MCM3	647120000	857970000	1,34	1,12	0,17	12,504	0,1160985	0,1769609	0,1453513	0,2363395	0,1623389	0,2549568	0,1428708	0,04194384	0,1314578	0,09220742	0,09828482	0,1463945	3,53E-06	5,45	0,145433764	+
Translational activator GCN1	GCN1L1	869440000	1020100000	1,24	1,12	0,17	17,07	0,1955996	0,1870075	0,1208827	0,1092936	0,196733	0,1952215	-0,02492751	-0,0544073	-0,08952827	-0,04180318	-0,1178343	-0,08873027	2,15E-01	0,67	0,048958924	
Stress-induced-phosphoprotein 1	STIP1	1498300000	2285500000	1,55	1,12	0,16	9,9233	0,2894804	0,1518587	0,2290956	0,1986199	0,3141744	0,2062681	0,05907837	0,068602	NaN	NaN	0,07080147	0,0500489	5,24E-04	3,28	0,163802798	+
Phenylalanine--tRNA ligase alpha subunit	FARSA	213380000	283670000	1,38	1,12	0,16	10,036	0,2284801	0,2240403	0,03492	0,1384213	0,06032421	0,02147971	NaN	0,3029917	NaN	NaN	0,3354833	0,08270261	3,74E-03	2,43	0,158760353	+
Charged multivesicular body protein 5	CHMP5	16902000	21966000	1,57	1,12	0,16	11,847	NaN	0,2630345	0,1220763	0,1996252	0,2194628	0,1954736	NaN	NaN	-0,2644951	-0,06680508	NaN	0,06073913	1,91E-01	0,72	0,091138907	
Vacuolar protein sorting-associated protein VTA1 homolog	VTA1	55481000	77784000	1,47	1,12	0,16	17,242	0,1428708	0,1709506	-0,04009629	0,2299569	-0,03819892	-0,01905342	0,2867629	0,2189671	0,3617683	0,4142438	NaN	NaN	8,56E-03	2,07	0,172817185	+
Aldehyde dehydrogenase, mitochondrial	ALDH2	33613000	49301000	1,69	1,12	0,16	21,922	NaN	0,2589409	NaN	NaN	0,4360552	0,1853591	NaN	NaN	NaN	NaN	0,3188085	-0,01254818	3,37E-02	1,47	0,237323126	
Elongator complex protein 1	IKBKAP	101620000	123600000	1,27	1,12	0,16	12,214	0,2203299	0,24245	0,08705537	0,1663298	0,1867541	0,182438	-0,06759103	-0,1292447	NaN	-0,1134733	0,03026536	-0,04101628	1,29E-01	0,89	0,069481573	
ATP synthase subunit beta, mitochondrial	ATP5B	6233600000	8792900000	1,40	1,12	0,16	6,4964	0,1541943	0,2082676	0,1736392	0,1958516	0,1377659	0,1534163	0,2385423	0,2924284	0,167101	0,196733	-0,1364262	-0,06193259	1,84E-03	2,74	0,143298386	+
High mobility group protein B1	HMGB1	609990000	975590000	1,58	1,12	0,16	18,592	0,335369	0,3411888	0,1180938	0,1475672	0,10916	0,07860982	0,08406419	0,09761087	0,1959774	0,1870075	0,2005043	0,3436921	5,31E-05	4,27	0,186570419	+
Trifunctional enzyme subunit beta, mitochondrial;3-ketoacyl-CoA thiolase	HADHB	263470000	387200000	1,49	1,12	0,16	12,693	0,3208889	0,2033891	0,5634511	0,2775088	0,2075181	0,08637615	0,1048734	-0,2832462	NaN	NaN	0,09922788	0,1589833	3,04E-02	1,52	0,173897047	
SUMO-activating enzyme subunit 2	UBA2	113800000	153690000	1,43	1,12	0,16	15,543	0,1461339	0,2834477	0,1329059	0,1835818	0,2951353	0,1681285	0,1216786	0,2360947	0,1570437	0,1548424	-0,2542894	-0,3008748	6,93E-02	1,16	0,110319021	
6-phosphogluconate dehydrogenase, decarboxylating	PGD	1729800000	2490400000	1,44	1,12	0,16	12,473	0,2395203	0,2447654	0,2315553	0,1820566	0,1909309	0,215368	0,02389408	0,04026129	NaN	NaN	0,0116388	0,0610158	1,15E-03	2,94	0,144100646	+
Dolichyl-diphosphooligosaccharide--protein glycosyltransferase 48 kDa subunit	DDOST	446540000	607870000	1,40	1,12	0,16	12,636	0,340961	0,1662012	0,2941943	0,1629835	0,1758121	0,1410403	NaN	NaN	NaN	NaN	-0,02384173	-0,0173002	1,15E-02	1,94	0,155006307	+
Luc7-like protein 3	LUC7L3	17891000	19568000	1,28	1,12	0,16	19,149	NaN	NaN	NaN	NaN	-0,06812029	-0,03386486	-0,03732512	-0,1233083	NaN	NaN	0,3459641	0,2065182	5,50E-01	0,26	0,048310628	
Far upstream element-binding protein 2	KHSRP	720680000	1018700000	1,59	1,12	0,16	16,717	0,2331518	0,1772162	-0,002714805	0,03562387	-0,02344575	0,09166604	0,168642	0,1597583	0,2368294	0,3307878	0,3831652	0,2179751	7,45E-04	3,13	0,167387932	+
Ras-related protein Rab-18	RAB18	24579000	37451000	1,52	1,12	0,16	8,6213	0,2236697	0,1210154	0,08038475	0,1079557	0,1971105	0,09017591	0,1510794	-0,0129994	0,06639916	0,1708224	0,1573025	-0,0482332	6,84E-04	3,17	0,108723568	+
26S protease regulatory subunit 7	PSMC2	553440000	671440000	1,33	1,12	0,16	14,63	0,03717139	0,1500396	0,01120934	0,01235413	0,1894135	0,1544536	-0,041046	0,1240634	NaN	NaN	0,02204824	0,1223414	1,16E-02	1,93	0,078204866	+
Peroxiredoxin-2	PRDX2	894350000	1465600000	1,61	1,11	0,16	11,958	0,1790014	0,2717842	0,2923105	0,3344539	0,3306731	0,2880631	-0,002367994	0,03294719	0,0321009	-0,02670465	0,08283886	0,004609331	3,90E-03	2,41	0,151642482	+
Alpha-soluble NSF attachment protein	NAPA	55335000	73576000	1,38	1,11	0,16	16,978	0,1610493	0,1301402	0,1783641	0,1901724	0,1719754	-0,08814742	NaN	-0,2475254	0,007626157	0,03745244	0,0551957	-0,08425727	2,97E-01	0,53	0,046549597	
NADH-cytochrome b5 reductase 3;NADH-cytochrome b5 reductase 3 membrane-bound form;NADH-cytochrome b5 reductase 3 soluble form	CYB5R3	42927000	62655000	1,39	1,11	0,16	19,252	0,1561377	0,1801479	NaN	0,3124328	0,1802752	0,4368015	NaN	-0,008290378	0,06460693	-0,04377975	-0,007246082	0,1280294	1,66E-02	1,78	0,139911524	+
Actin-related protein 3	ACTR3	263840000	379020000	1,36	1,11	0,16	14,343	0,1422174	0,1252544	0,1302719	0,168257	0,196733	0,09936258	-0,01976993	0,1311943	NaN	NaN	0,1130337	0,06350289	1,78E-04	3,75	0,115005714	+
Proteasome subunit beta type-6	PSMB6	187350000	273450000	1,63	1,11	0,15	3,8969	0,1418251	0,1527676	0,09247811	0,1432628	0,1119661	0,2115106	0,1471764	0,1544536	0,1021878	NaN	0,08270261	0,2470768	2,19E-06	5,66	0,144309789	+
26S proteasome non-ATPase regulatory subunit 12	PSMD12	368490000	543270000	1,49	1,11	0,15	11,28	0,2017593	0,3144065	0,3251561	0,1911836	0,2404974	0,2752454	0,07710631	0,1071523	0,1184926	-0,1747517	0,08827728	0,05408442	3,06E-03	2,51	0,151550803	+
Cleavage and polyadenylation specificity factor subunit 7	CPSF7	49206000	60185000	1,49	1,11	0,15	24,534	-0,1718234	0,09855437	NaN	-0,1283456	NaN	-0,2590984	NaN	0,5272706	0,2423281	0,242572	0,2651968	0,3148704	1,87E-01	0,73	0,125724983	
Sodium/potassium-transporting ATPase subunit alpha-1	ATP1A1	358310000	448060000	1,31	1,11	0,15	14,288	0,262794	0,2320467	0,1267086	0,2278643	0,1978653	0,2097652	-0,0536135	-0,1139572	-0,01762146	-0,01651203	-0,09335561	-0,09367894	1,21E-01	0,92	0,072358778	
26S protease regulatory subunit 4	PSMC1	352220000	500600000	1,47	1,11	0,15	15,936	0,1732553	0,07231157	0,2462257	0,02531228	0,3651326	0,1593708	-0,02620508	0,1001703	NaN	NaN	-0,1276361	-0,04046724	7,18E-02	1,14	0,094747006	
Malignant T-cell-amplified sequence 1	MCTS1	72925000	107310000	1,54	1,11	0,15	9,4955	0,1290852	0,102994	0,04628156	0,244887	-0,09722405	-0,05235628	0,1471764	0,2835663	0,1007085	0,1545833	0,2314324	0,1656869	3,48E-03	2,46	0,121401771	+
Cytochrome c1, heme protein, mitochondrial	CYC1	91405000	117700000	1,37	1,11	0,15	12,112	0,222681	0,1151664	0,2899525	0,1938348	0,2125071	0,1958516	NaN	NaN	-0,0155054	-0,1812803	NaN	-0,2098285	1,73E-01	0,76	0,091486562	
Cofilin-1	CFL1	2916000000	4254300000	1,42	1,11	0,15	8,7753	0,1368475	0,09207205	0,08283886	0,1918153	0,07587489	0,1424789	0,2360947	0,1678717	0,1458731	0,1758121	0,0938305	0,09301919	1,41E-06	5,85	0,136202386	+
Poly(rC)-binding protein 1	PCBP1	1370600000	1942400000	1,56	1,11	0,15	17,185	0,2838032	0,2389091	0,2151194	0,2000021	0,06460693	0,09922788	0,108491	0,1460035	0,09193667	0,04194384	0,2433034	0,1649149	2,26E-05	4,65	0,15818849	+
SH3 domain-binding glutamic acid-rich-like protein 3	SH3BGRL3	17892000	19892000	1,40	1,11	0,15	3,1522	NaN	0,05602856	NaN	NaN	0,2808376	NaN	-0,1267222	-1,525206	NaN	0,04208399	NaN	NaN	4,76E-01	0,32	-0,25459559	
14-3-3 protein beta/alpha;14-3-3 protein beta/alpha, N-terminally processed	YWHAB	463760000	711640000	1,51	1,11	0,15	12,233	0,06543438	0,1137005	0,1646576	0,1152996	0,1285573	0,1103632	0,2881813	0,2681949	0,1758121	0,1478277	0,138028	0,1849785	3,78E-06	5,42	0,158419597	+
26S protease regulatory subunit 10B	PSMC6	476930000	681420000	1,45	1,11	0,15	17,667	0,01449814	0,2236697	-0,03044239	0,01621099	0,1082234	0,1096949	-0,1109782	-0,1052094	0,07751644	-0,03268384	NaN	NaN	4,34E-01	0,36	0,02704997	
Methionine adenosyltransferase 2 subunit beta	MAT2B	50278000	72606000	1,36	1,10	0,14	2,4643	-0,01938971	-0,02916106	0,1624679	0,09895846	NaN	0,2417182	NaN	0,2220626	0,1247251	0,1255188	NaN	NaN	1,31E-02	1,88	0,11586252	+
ATP synthase F(0) complex subunit B1, mitochondrial	ATP5F1	365790000	410960000	1,42	1,10	0,14	10,811	0,1037997	0,1540647	0,1858666	0,1454818	0,1651723	0,07956584	-0,101366	-0,2589948	-0,09694628	-0,102171	-0,1077722	-0,118304	9,27E-01	0,03	0,004033058	
D-dopachrome decarboxylase;D-dopachrome decarboxylase-like protein	DDT;DDTL	51049000	74882000	1,38	1,10	0,14	7,1192	0,2590615	0,1443075	0,1512094	NaN	0,2771516	NaN	-0,08482332	0,1098286	-0,09983458	0,04600206	-0,09520379	-0,006332865	1,50E-01	0,82	0,07013661	
Alpha-actinin-4	ACTN4	3799300000	5283500000	1,47	1,10	0,14	9,6502	0,169027	0,1664584	0,2502339	0,2276179	0,1731275	0,2133784	0,09922788	0,1302719	0,09504669	0,1376347	0,03969989	0,02970023	1,85E-05	4,73	0,144285367	+
Sarcoplasmic/endoplasmic reticulum calcium ATPase 2	ATP2A2	272820000	330400000	1,29	1,10	0,14	9,4337	0,07477944	0,1489992	-0,0429621	0,07915612	0,2083924	0,1991226	-0,06096904	-0,1526444	-0,0511301	-0,0224783	-0,07793921	0,1269729	3,20E-01	0,49	0,035774967	
Heat shock 70 kDa protein 4	HSPA4	2064900000	2810000000	1,34	1,10	0,14	10,659	0,1687703	0,1902988	0,2092662	0,2073932	0,2485348	0,1953476	0,04404436	0,04977013	0,05616735	0,005615641	0,07833651	0,06598585	2,77E-04	3,56	0,126627564	+
Coatomer subunit gamma-2	COPG2	222190000	321530000	1,33	1,10	0,14	11,614	0,2088917	0,1373724	0,2182232	0,1810389	0,1259155	0,2137517	0,1989969	0,07258602	0,2921927	0,3573831	0,2620723	0,3351402	2,35E-06	5,63	0,216963739	+
RNA-binding protein 25	RBM25	32010000	50335000	1,09	1,10	0,14	13,216	-0,2204629	0,1076879	0,1493894	0,1133004	-0,2759784	NaN	0,2952528	0,3368547	0,4016304	0,3014705	NaN	0,4392508	6,33E-02	1,20	0,164839563	
26S proteasome non-ATPase regulatory subunit 2	PSMD2	487270000	688290000	1,40	1,10	0,14	13,509	0,1522483	0,09207205	0,1826923	0,06433112	0,1654296	0,1781091	0,1579492	0,2006298	0,2075181	0,1443075	0,1361914	0,182438	6,35E-08	7,20	0,155326377	+
Programmed cell death protein 6	PDCD6	77056000	120670000	1,55	1,10	0,14	4,5685	0,1782366	0,279293	NaN	0,1809117	0,2326607	0,243791	0,1112984	0,1667154	0,01349801	-0,001241217	0,08270261	0,2053925	2,46E-04	3,61	0,153932602	+
26S proteasome non-ATPase regulatory subunit 13	PSMD13	774070000	1063700000	1,48	1,10	0,14	11,869	0,1875144	0,2231754	0,2073932	0,1628546	0,1768333	0,2183473	-0,03117934	-0,03768053	-0,004638512	-0,02450185	NaN	NaN	1,62E-02	1,79	0,107811788	+
Alcohol dehydrogenase [NADP(+)]	AKR1A1	47703000	68579000	1,52	1,10	0,14	14,825	0,001874253	0,221444	0,000288558	0,2321696	0,3576083	0,3416442	-0,1032244	-0,3711797	0,2182232	0,01663897	NaN	NaN	2,32E-01	0,63	0,091548697	
28S ribosomal protein S5, mitochondrial	MRPS5	21124000	25726000	1,64	1,10	0,14	20,401	-0,1044182	-0,07260414	-0,2328877	NaN	0,0265876	0,06198353	NaN	NaN	0,1186256	0,02856912	NaN	NaN	6,01E-01	0,22	-0,024877737	
Eukaryotic translation initiation factor 4E	EIF4E	66536000	87265000	1,45	1,10	0,14	20,213	0,1436546	0,05408442	0,1629835	0,1866274	0,01891905	-0,06821108	0,3643483	0,2505978	0,2369518	0,1040682	0,186247	0,3176514	8,10E-04	3,09	0,163160194	+
Peptidyl-prolyl cis-trans isomerase-like 1	PPIL1	48006000	68273000	1,53	1,10	0,14	16,264	NaN	0,3971458	0,3304437	0,4349884	0,2697513	0,2261386	0,02361021	0,1367164	0,09922788	0,1712069	0,05032762	0,2293418	3,61E-04	3,44	0,215354427	+
Bifunctional methylenetetrahydrofolate dehydrogenase/cyclohydrolase, mitochondrial;NAD-dependent methylenetetrahydrofolate dehydrogenase;Methenyltetrahydrofolate cyclohydrolase	MTHFD2	24412000	34864000	1,29	1,10	0,14	7,6668	0,038998	0,2515676	NaN	0,1059462	0,1514692	0,3028747	0,1881477	-0,04662296	0,1050075	0,1649149	0,09180144	0,2158649	7,29E-04	3,14	0,142724478	+
Fumarate hydratase, mitochondrial	FH	1136500000	1642200000	1,44	1,10	0,14	7,3336	0,1928254	0,1899194	0,2136273	0,1393385	0,04684024	0,1306673	0,1043367	0,1044708	0,009060428	-0,00371246	0,09734114	0,114367	1,63E-04	3,79	0,111590149	+
NADH dehydrogenase [ubiquinone] flavoprotein 1, mitochondrial	NDUFV1	32046000	42590000	1,30	1,10	0,14	12,835	0,001442041	0,06295063	0,2274946	0,3283769	0,07052668	-0,04052658	NaN	NaN	NaN	NaN	0,2382977	-0,2689037	2,85E-01	0,54	0,077457291	
Neutral alpha-glucosidase AB	GANAB	1525600000	1905700000	1,29	1,10	0,14	9,17	0,1288213	0,116764	0,1200864	0,177599	0,1894135	0,1830736	0,127105	0,1317212	0,06543438	0,1020534	0,166587	0,1104968	7,37E-08	7,13	0,134929633	+
Proliferating cell nuclear antigen	PCNA	382420000	593080000	1,52	1,10	0,14	10,632	0,1480882	0,0586628	0,1672295	0,2188431	0,1579492	0,1492593	0,1818022	0,1054098	0,1418251	0,1180938	0,05144163	0,1019189	1,28E-06	5,89	0,13337697	+
Serine--tRNA ligase, cytoplasmic	SARS	727090000	1041800000	1,31	1,10	0,14	7,1137	0,138028	0,1756843	0,2459823	0,196733	0,1228714	0,1175621	0,09815002	-0,02343107	NaN	NaN	0,03969989	0,12075	6,48E-04	3,19	0,123203	+
ADP-sugar pyrophosphatase	NUDT5	136040000	178200000	1,23	1,10	0,14	7,0541	0,06350289	0,002018352	0,1717192	0,06708801	0,08311115	0,1433935	0,04054174	-0,1050698	0,2141248	0,05158086	-0,02007709	-0,06996664	7,49E-02	1,13	0,05349725	
Septin-7	SEPTIN7	236930000	356370000	1,59	1,10	0,13	11,899	0,5060941	0,4143521	0,322505	0,1513393	0,1141004	0,2045164	0,07724293	-0,146643	NaN	NaN	0,01777946	0,1008431	1,82E-02	1,74	0,176212965	+
Kinesin-1 heavy chain	KIF5B	308620000	345280000	1,22	1,10	0,13	13,005	0,167101	0,2352368	0,0938305	0,1636276	0,1859933	0,1234014	0,06446896	0,004033884	-0,09164824	-0,2848095	-0,07172444	0,1625968	1,75E-01	0,76	0,062675666	
Ubiquitin-associated domain-containing protein 1	UBAC1	128320000	149190000	1,34	1,10	0,13	13,083	0,2451305	0,138028	-0,002367994	-0,004059682	0,002738459	0,0889556	NaN	NaN	-0,2361975	-0,1727988	-0,2163171	-0,2344992	4,84E-01	0,31	-0,039138773	
Coatomer subunit zeta-1	COPZ1	136670000	204640000	1,37	1,10	0,13	11,184	0,103934	0,08052122	0,06143053	0,1023222	0,1557492	0,1443075	0,2976023	0,169925	0,1184926	0,2237931	0,298541	0,3043942	3,24E-05	4,49	0,171751085	+
Ras-related protein Rab-5A	RAB5A	60189000	94790000	1,50	1,10	0,13	12,373	0,4618429	0,3335382	0,315798	0,1749178	0,2015084	0,4077334	0,2221863	0,06433112	0,1646576	-0,01851268	0,2547149	0,1910572	1,04E-04	3,98	0,231147761	+
Protein canopy homolog 2	CNPY2	43865000	60606000	1,31	1,10	0,13	16,014	0,1818022	0,1847246	0,02119536	0,09274875	0,1198209	0,1259155	0,1375035	0,2486562	0,1878943	0,2542312	0,1606621	0,1959774	3,90E-06	5,41	0,159261022	+
Spermine synthase	SMS	97478000	121320000	1,33	1,10	0,13	13,583	0,1904253	0,1930779	0,08841301	0,01934605	0,1963552	-0,1208903	NaN	0,5018213	-0,248862	-0,1650623	NaN	NaN	3,73E-01	0,43	0,07273602	
Cytosolic acyl coenzyme A thioester hydrolase	ACOT7	55299000	73111000	1,17	1,09	0,13	17,385	0,1398623	0,3552418	0,2492632	0,1305355	-0,12699	0,3253864	0,02899346	0,09180144	0,1439158	NaN	0,2729787	0,3503841	2,74E-03	2,56	0,178306611	+
Bifunctional purine biosynthesis protein PURH;Phosphoribosylaminoimidazolecarboxamide formyltransferase;IMP cyclohydrolase	ATIC	1293000000	1869800000	1,34	1,09	0,13	8,3667	0,09180144	0,111432	0,1047392	0,1202192	0,1419559	0,1646576	0,243791	0,2678354	NaN	NaN	0,2148708	0,1858666	1,41E-05	4,85	0,164716904	+
Long-chain-fatty-acid--CoA ligase 3	ACSL3	140220000	171260000	1,36	1,09	0,13	14,109	-0,25515	0,002738459	-0,1058458	0,07176267	0,03843611	-0,05419756	0,1054098	0,1658154	0,3370831	0,1116991	0,06419311	0,2135028	2,18E-01	0,66	0,057953937	
V-type proton ATPase subunit H	ATP6V1H	11880000	14941000	1,31	1,09	0,13	2,246	0,1528972	0,07464243	NaN	NaN	-0,01187885	-0,02947023	NaN	NaN	NaN	NaN	0,09518178	0,2705888	9,78E-02	1,01	0,091993517	
Heat shock protein 75 kDa, mitochondrial	TRAP1	891760000	1189600000	1,32	1,09	0,13	13,855	0,1615652	0,1228714	0,1267086	0,1273692	0,110096	0,1264442	-0,002440175	0,1253867	-0,04482113	0,05227683	0,001586012	-0,05944982	8,99E-03	2,05	0,070632756	+
26S proteasome non-ATPase regulatory subunit 14	PSMD14	143260000	209330000	1,31	1,09	0,13	8,1074	0,1120997	0,1017845	0,1252544	0,1518587	0,1082234	0,1623389	-0,0500393	0,01592577	0,1354035	-0,112506	0,05199848	-0,1142851	7,23E-02	1,14	0,057338085	
Clathrin heavy chain 1	CLTC	3491000000	4431100000	1,25	1,09	0,13	9,8184	0,09031146	0,08610431	0,1959774	0,1966071	0,1416943	0,2123825	-0,01737322	0,008343471	0,03717139	0,06626152	0,02899346	0,03927886	2,15E-03	2,67	0,090479381	+
Histidine triad nucleotide-binding protein 1	HINT1	57407000	84154000	1,42	1,09	0,13	20,158	0,2163618	0,3068455	0,1031283	0,8281441	0,1047392	0,01521204	0,04977013	0,07546414	0,09976643	0,03026536	-0,2790562	NaN	1,14E-01	0,94	0,14096735	
Tropomyosin alpha-4 chain	TPM4	33220000	34343000	1,66	1,09	0,13	15,152	NaN	0,4474205	NaN	0,1792563	-0,1842606	0,503247	NaN	NaN	0,05533457	0,09125975	NaN	-0,07966096	1,84E-01	0,73	0,144656641	
Tricarboxylate transport protein, mitochondrial	SLC25A1	216470000	314120000	1,44	1,09	0,12	10,323	-0,07987443	0,04012096	0,2446436	0,251204	0,0618453	0,005328146	0,1701815	0,1248574	0,07942928	0,1191569	0,1255188	0,151729	2,33E-03	2,63	0,10784504	+
NEDD8-conjugating enzyme Ubc12	UBE2M	73653000	101340000	1,40	1,09	0,12	5,7867	0,07888308	0,100574	0,1867541	0,1278974	0,2073932	0,1317212	-0,02916106	-0,06117976	-0,04223398	-0,3869777	0,1051417	NaN	4,67E-01	0,33	0,038073828	
Nucleolysin TIA-1 isoform p40	TIA1	30079000	38302000	1,36	1,09	0,12	13,164	NaN	0,3034593	NaN	0,1978653	NaN	NaN	NaN	NaN	-0,1047594	-0,2342277	NaN	NaN	7,69E-01	0,11	0,040584382	
Histone acetyltransferase type B catalytic subunit	HAT1	400330000	530640000	1,40	1,09	0,12	11,523	0,1509495	0,1905516	0,1281614	0,03829577	0,07724293	0,1615652	0,1496496	0,06170706	-0,04723405	0,6473606	0,04334458	-0,02366567	2,71E-02	1,57	0,131494048	+
PITH domain-containing protein 1	PITHD1	88782000	114430000	1,40	1,09	0,12	8,7883	0,0729976	0,1381591	0,07093885	0,2637556	0,2732175	0,1372412	NaN	-0,01938971	0,1296127	0,1619522	-0,07067864	0,08488071	4,77E-03	2,32	0,11297156	+

NADH dehydrogenase [ubiquinone] iron-sulfur protein 2, mitochondrial	NDUFS2	62102000	89966000	1,32	1,09	0,12	14,45	NaN	0,001297885	-0,1411596	NaN	0,123931	0,2159891	NaN	NaN	NaN	NaN	0,5237627	0,4439248	1,21E-01	0,92	0,194624317	
Monocarboxylate transporter 1	SLC16A1	192680000	244780000	1,19	1,09	0,12	7,7599	0,1496496	0,2934882	0,0533895	0,1521185	0,4757075	0,1578198	NaN	0,6242412	0,2553195	0,1502997	NaN	NaN	2,99E-03	2,52	0,256892611	+
Protein FAM49B	FAM49B	89672000	137430000	1,46	1,09	0,12	10,156	0,1496496	0,1892869	0,2215678	0,2311866	0,2228046	0,2722622	0,01449814	0,1513393	0,08229379	0,1642714	0,000721115	0,06391706	1,28E-04	3,89	0,146983208	+
Peptidyl-prolyl cis-trans isomerase FKBP5;Peptidyl-prolyl cis-trans isomerase FKBP5, N-terminally processed	FKBP5	211480000	272960000	1,30	1,09	0,12	17,591	0,02375215	0,1035311	0,1021878	0,06391706	0,06295063	0,1304037	NaN	NaN	0,3111543	-0,007942225	NaN	NaN	2,35E-02	1,63	0,098744328	+
Ubiquitin-conjugating enzyme E2 K	UBE2K	194830000	292710000	1,44	1,09	0,12	12,942	-0,06321318	-0,1196671	0,1963552	0,03689012	0,1407786	-0,02866064	0,1731275	0,08569661	0,319618	0,286408	0,007482617	0,3426685	2,52E-02	1,60	0,114790357	+
26S protease regulatory subunit 6B	PSMC4	204320000	290070000	1,42	1,09	0,12	17,283	0,1175621	0,1310627	0,1842168	0,09234277	0,141302	0,1587248	NaN	NaN	NaN	NaN	0,1870075	0,2892443	1,24E-04	3,91	0,162682864	+
S-formylglutathione hydrolase	ESD	172730000	261140000	1,48	1,09	0,12	9,5767	0,120352	-0,005782364	0,1430015	-0,09703889	0,2071432	0,01035019	-0,006129998	0,07231157	0,1992483	0,1107641	0,1003049	0,1149001	1,01E-02	2,00	0,080785388	+
Ras-related protein Rab-10	RAB10	73459000	116610000	1,47	1,09	0,12	15,399	0,09801521	0,2008809	0,1382902	0,1945914	0,1445685	0,2952528	0,1759396	-0,01543256	0,1934563	0,1137005	0,1719754	0,0441843	5,89E-05	4,23	0,146285228	+
Transketolase	TKT	2104800000	2752700000	1,40	1,09	0,12	6,4679	0,06915226	0,06212175	0,09301919	0,09504669	0,1023222	0,1047392	0,2090166	0,1783641	0,2284801	0,113167	0,1035311	0,10916	6,97E-06	5,16	0,122343354	+
Proteasome subunit alpha type-3	PSMA3	442530000	636090000	1,54	1,09	0,12	4,1447	0,1650436	0,1150332	0,05325044	0,1004395	0,1044708	0,167101	0,07819984	0,02856912	0,0446039	0,06225996	0,06529664	0,07710631	2,41E-05	4,62	0,088447857	+
DNA replication complex GINS protein PSF1	GINS1	9025100	10339000	1,65	1,09	0,12	3,6885	NaN	0,1280294	-0,003452207	0,03506084	0,02644594	NaN	NaN	NaN	NaN	NaN	0,2654369	-0,104899	3,13E-01	0,50	0,05777031	
Ras-related protein Rab-1B;Putative Ras-related protein Rab-1C	RAB1B;RAB1C	550940000	809400000	1,47	1,09	0,12	18,099	0,1187585	0,1158324	0,1265764	0,1882743	0,1763228	0,2152437	-0,01214072	0,05130255	0,004177767	0,05102402	0,04404436	0,04879416	1,05E-03	2,98	0,094017513	+
78 kDa glucose-regulated protein	HSPA5	3809200000	5027400000	1,35	1,09	0,12	11,433	0,05366741	0,04376442	0,03745244	0,03885755	-0,02616103	-0,01613266	0,2126316	0,2773897	0,1296127	0,1914362	0,1981169	0,2570107	3,16E-03	2,50	0,116470497	+
Cytochrome b-c1 complex subunit Rieske, mitochondrial;Cytochrome b-c1 complex subunit 11;Putative cytochrome b-c1 complex subunit Rieske-like protein 1	UQCRFS1;UQCRFS1P1	72769000	111130000	1,50	1,09	0,12	9,7324	0,05380643	0,115566	0,3102238	0,3774015	0,3155663	0,2149951	-0,1991229	0,03068919	0,03618686	0,03435652	0,02219017	-0,1546826	9,05E-02	1,04	0,096431353	
Aconitate hydratase, mitochondrial	ACO2	477130000	652460000	1,42	1,09	0,12	18,363	0,203765	0,1540647	0,2258919	0,205017	0,13711	0,1994997	-0,06136038	-0,09015781	-0,01661415	-0,12636	-0,03393872	0,08610431	7,76E-02	1,11	0,07358512	
Ezrin	EZR	526420000	750580000	1,39	1,08	0,12	13,258	0,2595437	0,249506	0,3414165	0,2515676	0,4059924	0,2917214	-0,09400225	-0,05727155	-0,001934467	0,01977311	-0,05716651	-0,02997102	3,00E-02	1,52	0,131597914	
Oxygen-dependent coproporphyrinogen-III oxidase, mitochondrial	CPOX	28653000	42062000	1,26	1,08	0,12	13,643	0,01635366	-0,1841459	0,2239167	NaN	0,06460693	0,07778991	0,2865263	NaN	0,2284801	0,2464689	0,2313095	0,2601461	1,32E-02	1,88	0,145145233	+
Aminoacyl tRNA synthase complex-interacting multifunctional protein 1;Endothelial monocyte-activating polypeptide 2	AIMP1	46091000	61342000	1,35	1,08	0,11	15,161	0,201132	NaN	0,1797658	0,1428708	0,108491	0,2459823	-0,02598469	-0,2235085	-0,1616371	-0,395549	NaN	-0,112272	9,53E-01	0,02	-0,004070946	
Sorting nexin-5	SNX5	52481000	67351000	1,24	1,08	0,11	23,242	0,09990108	0,1195554	0,1138339	0,05380643	-0,1080831	0,284751	NaN	NaN	NaN	NaN	0,009777201	-0,09294018	2,22E-01	0,65	0,060075212	
Histone-binding protein RBBP4	RBBP4	516760000	759040000	1,44	1,08	0,11	25,033	0,1585955	0,1491292	0,2374413	0,2251515	0,05144163	0,1576905	0,07436837	0,1423481	NaN	NaN	0,00805652	0,0244615	8,93E-04	3,05	0,122868423	+
Vesicle-associated membrane protein 2	VAMP2	25368000	32505000	1,34	1,08	0,11	28,803	-0,2658822	0,6683007	0,1519886	0,3438057	0,2279875	0,2860532	-0,1485448	-0,05896888	NaN	NaN	-0,0680901	NaN	2,30E-01	0,64	0,126294414	
Protein transport protein Sec24C	SEC24C	317590000	389850000	1,28	1,08	0,11	6,6259	0,08243007	0,1478277	0,07491644	0,08678382	0,0878702	0,08147596	0,1800205	0,1120997	0,1527676	0,0745054	-0,08974321	0,1774714	6,13E-04	3,21	0,097368796	+
ATP synthase subunit f, mitochondrial	ATP5J2	51984000	95726000	1,55	1,08	0,11	12,692	0,1500396	0,0278618	-0,04610153	0,2063932	0,1562672	0,1304037	0,0640551	0,2602666	0,1036654	0,1359287	0,04907312	-0,009422677	2,15E-03	2,67	0,102369192	+
Ras-related protein Rab-2A	RAB2A	194260000	228600000	1,48	1,08	0,11	5,1607	0,06681251	0,09788039	0,02913487	0,1232689	0,1384213	0,1265764	0,1381591	0,06433112	0,05797008	0,02361021	0,003889986	-0,008072794	6,30E-04	3,20	0,071831847	+
NADH dehydrogenase [ubiquinone] iron-sulfur protein 8, mitochondrial	NDUFS8	8041200	12610000	1,54	1,08	0,11	9,983	0,07601179	0,1096949	NaN	NaN	NaN	NaN	0,6358008	0,02119536	0,4436067	NaN	NaN	NaN	9,88E-02	1,01	0,257261893	
Ras-related protein Rab-1A	RAB1A	47915000	73642000	1,51	1,08	0,11	8,5658	0,1994997	0,1624679	0,1043367	0,2589409	0,2108876	0,1844707	0,1082234	0,2584586	0,2508404	-0,01384391	-0,06837742	-0,1155662	5,60E-03	2,25	0,128361533	+
Ras-related protein Rap-2b	RAP2B	5730200	8816800	1,47	1,08	0,11	12,864	0,222681	0,2021356	NaN	0,4215869	0,100574	NaN	0,2337653	0,05311137	0,3554674	NaN	NaN	NaN	3,60E-03	2,44	0,227045944	+
Eukaryotic translation elongation factor 1 epsilon-1	EEF1E1	124790000	174350000	1,48	1,08	0,11	9,2271	0,167358	0,2052674	0,1051417	0,07477944	0,351063	0,2406196	-0,08800939	-0,05740666	0,003602319	-0,0339978	-0,02783677	-0,04485093	9,21E-02	1,04	0,074644147	
Apoptosis regulator BAX	BAX	26075000	38574000	1,55	1,08	0,11	16,754	0,1292171	0,1008431	0,2258919	0,0745054	0,09774555	0,1035311	NaN	-0,1613467	-0,1488646	-0,2800368	-0,04966597	-0,5516699	5,56E-01	0,25	-0,041804526	
2-oxoglutarate dehydrogenase, mitochondrial	OGDH	24024000	28507000	1,31	1,08	0,11	15,218	0,08501676	0,08093054	0,0353423	0,2023863	0,1463945	0,23008	NaN	NaN	NaN	NaN	0,05547326	-0,03928072	1,60E-02	1,80	0,099542865	+
Calcium-binding mitochondrial carrier protein Aralar2	SLC25A13	223720000	299580000	1,31	1,08	0,11	14,549	0,08079411	0,1080895	0,2036397	0,1950955	-0,0842879	0,01292609	0,4692609	0,5260689	NaN	0,1068843	0,2213203	-0,106234	2,60E-02	1,59	0,157596123	+
FACT complex subunit SSRP1	SSRP1	55510000	63258000	1,26	1,08	0,11	13,767	NaN	0,1307991	NaN	0,351176	-0,4529055	-0,549789	-0,2128183	-0,3566055	-0,3918909	-0,2660037	NaN	0,1062144	1,13E-01	0,95	-0,182424834	
Delta-1-pyrroline-5-carboxylate synthase;Glutamate 5-kinase;Gamma-glutamyl phosphate reductase	ALDH18A1	214780000	253420000	1,27	1,08	0,11	14,381	-0,09669945	0,07696952	0,07942928	0,1373724	0,01178193	0,1832007	NaN	0,03125411	-0,100747	-0,1620567	-0,2780063	-0,1604112	5,74E-01	0,24	-0,02526479	
Translocon-associated protein subunit delta	SSR4	92500000	135270000	1,51	1,08	0,11	7,2091	0,103397	0,05018827	0,2526579	0,297837	0,144699	0,19131	0,2803624	0,1210154	0,04837578	0,08161233	0,0160683	0,1393385	2,46E-04	3,61	0,143905166	+
Actin-related protein 2/3 complex subunit 3	ARPC3	36898000	51212000	1,48	1,08	0,11	14,27	0,2866446	-0,03821367	NaN	0,09693644	0,06391706	0,1067503	-0,01473279	0,21661	0,1103632	0,1544536	NaN	NaN	1,28E-02	1,89	0,109192086	+
Transitional endoplasmic reticulum ATPase	VCP	2780700000	3770800000	1,43	1,08	0,11	10,099	0,1071523	0,09788039	0,1480882	0,07683272	0,08202134	0,1342209	0,1306673	0,1196882	0,07929271	0,09761087	0,1202192	0,1135672	4,33E-09	8,36	0,108936772	+
Hypoxia up-regulated protein 1	HYOU1	1263500000	1584500000	1,24	1,08	0,11	19,477	0,1458731	0,09450638	0,104605	0,1460035	0,1182268	0,08936255	0,0699771	0,09369545	0,05921687	0,06322686	0,0766959	0,07642223	2,34E-07	6,63	0,094817641	+
Protein disulfide-isomerase	P4HB	1914100000	2607900000	1,37	1,08	0,11	9,1691	0,1021878	0,05838585	0,1243282	0,08256635	0,08868441	0,09274875	0,2260152	0,1523782	NaN	NaN	-0,009640465	0,003602319	2,12E-03	2,67	0,09212566	+
Calreticulin	CALR	1280900000	1927000000	1,45	1,08	0,11	15,811	0,2055176	0,2083924	0,09504669	0,1102295	0,1764505	0,1547128	0,06073913	0,08963377	NaN	NaN	0,2070182	0,05533457	6,07E-05	4,22	0,136307518	+
Proteasome subunit beta type-4	PSMB4	209890000	312910000	1,56	1,08	0,10	5,5051	0,2193389	0,1453513	0,1712069	0,1348781	0,112233	0,3011194	0,02899346	0,04628156	-0,01554921	0,0182068	0,1984942	0,01278325	1,95E-03	2,71	0,114444801	+
26S proteasome non-ATPase regulatory subunit 6	PSMD6	627630000	796980000	1,36	1,07	0,10	14,364	0,2276179	0,1483484	0,01877663	0,09761087	0,07560106	0,1174291	-0,2528618	-0,2428572	-0,1284402	-0,2128684	0,1508197	0,3663642	6,06E-01	0,22	0,030461681	
Signal transducer and activator of transcription 3	STAT3	72485000	113780000	1,35	1,07	0,10	19,102	0,1094274	0,2727398	0,04278438	0,3517414	0,03280615	0,1102295	-0,1848673	NaN	-0,7285266	-0,6849903	NaN	NaN	5,77E-01	0,24	-0,075406174	
Crk-like protein	CRKL	815730000	1050300000	1,34	1,07	0,10	8,806	0,06018582	-0,06345443	0,04656085	0,03506084	0,06143053	0,09491159	-0,01687687	0,06777637	-0,007289554	0,0451634	0,4531224	0,2702299	5,49E-02	1,26	0,087235067	
Peptidyl-prolyl cis-trans isomerase F, mitochondrial	PPIF	93997000	139710000	1,35	1,07	0,10	12,472	0,09125975	0,1701815	0,08610431	NaN	-0,007608591	0,07519039	0,294312	0,1964812	0,2502339	0,2961931	0,3360548	0,3572705	3,39E-04	3,47	0,195061168	+
Myotrophin	MTPN	133670000	191650000	1,48	1,07	0,10	8,7326	0,1292171	0,09098883	0,2236697	0,1660727	0,12618	0,1696685	0,1390765	0,1646576	0,1662012	0,187261	0,06832678	-0,03837668	3,18E-05	4,50	0,132745271	+
39S ribosomal protein L1, mitochondrial	MRPL1	14984000	20193000	1,41	1,07	0,10	7,7421	0,04278438	0,1263122	0,05060612	0,247806	-0,04449375	0,1426094	NaN	NaN	0,2513252	0,1722314	0,04054174	0,03941911	7,13E-03	2,15	0,106914182	+
Apoptosis-inducing factor 1, mitochondrial	AIFM1	143370000	187870000	1,35	1,07	0,10	17,43	0,1953476	0,1668439	0,1984942	0,3139424	-0,02268348	0,04236425	0,08922691	0,1329059	0,1357976	0,1688987	-0,03725109	0,1731275	8,55E-04	3,07	0,129751195	+
Rab GDP dissociation inhibitor beta	GDI2	3046600000	4169100000	1,36	1,07	0,10	9,4737	0,120352	0,1527676	0,1440464	0,08474465	0,07135073	0,1410403	0,05227683	0,1119661	NaN	NaN	-0,01489308	0,01421241	8,96E-04	3,05	0,087786397	+
Cleavage and polyadenylation specificity factor subunit 5	NUDT21	220370000	323690000	1,34	1,07	0,10	11,153	0,1636276	0,1585955	0,08243007	0,02431962	-0,1977323	-0,1333847	0,1732553	0,3124328	0,2313095	-0,04168439	0,2023863	0,2257685	4,92E-02	1,31	0,100110308	
Fermitin family homolog 3	FERMT3	336350000	407340000	1,32	1,07	0,10	15,645	0,0441843	0,02091112	0,04110281	0,114367	0,1076879	0,1257833	0,1591125	0,2808376	0,3779566	0,3350258	0,2757223	0,3398215	4,26E-04	3,37	0,185209389	+
V-type proton ATPase subunit B, brain isoform	ATP6V1B2	199060000	273300000	1,34	1,07	0,10	15,027	0,1440464	0,1664584	0,2375637	0,2299569	0,121546	0,1103632	0,2756031	0,240009	NaN	NaN	-0,006072053	0,06736329	3,15E-04	3,50	0,158683798	+
ATP synthase subunit O, mitochondrial	ATP5O	510580000	704100000	1,42	1,07	0,10	6,0843	0,05755419	0,06253617	0,07778991	0,1492593	0,07560106	0,03337007	0,292664	0,06046242	0,05102402	0,03998062	0,02517066	-0,02102828	7,07E-03	2,15	0,075365346	+
Glutathione S-transferase P	GSTP1	4079300000	5815300000	1,49	1,07	0,10	10,007	0,06681251	0,07505342	0,09545191	0,0938305	0,1075539	0,0439044	0,05352838	0,04348443	0,06004741	0,1182268	0,07710631	0,04068203	1,05E-06	5,98	0,072973501	+
Creatine kinase B-type	CKB	1369000000	1807400000	1,41	1,07	0,10	7,011	0,0729976	-0,06564207	0,1317212	0,05436236	0,104605	0,1212807	0,1807844	0,2408638	0,1886542	0,0416635	0,06887715	0,0321009	1,75E-03	2,76	0,097689058	+
Voltage-dependent anion-selective channel protein 1	VDAC1	907390000	1317300000	1,40	1,07	0,10	11,617	0,06308875	0,114367	0,1106305	0,06460693	0,1633699	0,09545191	-0,03244768	0,02616273	-0,03537217	-0,03793228	-0,05954006	-0,06155609	1,57E-01	0,80	0,034235787	
NADH dehydrogenase [ubiquinone] iron-sulfur protein 3, mitochondrial	NDUFS3	106360000	161500000	1,38	1,07	0,10	8,5076	-0,1022948	-0,0422191	0,008200003	0,003458378	-0,04299178	0,122474	0,2869995	0,2018848	0,2447654	0,2329063	0,2236697	0,2076431	1,68E-02	1,78	0,112041278	+
Golgi resident protein GCP60	ACBD3	40835000	48879000	1,25	1,07	0,09	3,7023	-0,06274599	-0,08912922	-0,003220786	-0,06564207	0,2330291	0,04558271	0,3186928	0,2379307	0,7557422	0,7483754	NaN	0,2709474	3,86E-02	1,41	0,217232935	
Heterogeneous nuclear ribonucleoprotein H2	HNRNPH2	18162000	28612000	2,06	1,07	0,09	11,573	NaN	NaN	NaN	NaN	0,2551986	-0,07501847	NaN	NaN	NaN	NaN	0,09477648	0,05658348	3,10E-01	0,51	0,08288503	
F-actin-capping protein subunit alpha-1	CAPZA1	378890000	544500000	1,37	1,07	0,09	12,611	0,0275787	0,06046242	0,1414329	0,1361914	0,3226204	0,06046242	0,01749438	0,06198353	0,09410089	-0,03320045	0,07313477	0,1534163	4,46E-03	2,35	0,092973127	+
Filamin-C	FLNC	594780000	723290000	1,23	1,07	0,09	14,123	0,03984026	0,111165	-0,05865324	0,02460336	0,1968587	0,1178281	0,1293489	0,07519039	0,0650208	0,1273692	0,2190911	0,2584586	1,34E-03	2,87	0,108843436	+
Protein disulfide-isomerase A4	PDIA4	674780000	863610000	1,37	1,07	0,09	13,472	-0,0783656	-0,08557326	-0,1188053	-0,0714819	-0,04562502	0,01178193	0,1354035	0,1571731	0,143524	0,1280294	0,1728715	0,2398868	2,03E-01	0,69	0,049068271	
Deoxycytidylate deaminase	DCTD	24729000	35244000	1,40	1,07	0,09	5,5643	0,1660727	0,1606621	0,1352722	0,1102295	0,211386	0,2796496	0,06529664	0,1736392	0,03195977	0,04502354	NaN	NaN	3,21E-04	3,49	0,137919108	+
Peptidyl-prolyl cis-trans isomerase H	PPIH	84195000	127870000	1,37	1,07	0,09	5,8756	-0,1253525	0,07272322	0,04990952	0,1306673	-0,01070114	0,02842765	0,08065767	0,2474415	0,1678717	0,1961033	0,09531686	0,1075539	1,09E-02	1,96	0,086718255	+
3(2),5-bisphosphate nucleotidase 1	BPNT1	101340000	145490000	1,38	1,07	0,09	10,596	-0,1032709	0,2457391	0,3535491	0,1712069	0,08270261	0,1486088	-0,2276244	-0,02644013	0,1281614	0,04907312	NaN	NaN	1,59E-01	0,80	0,082170558	
DNA replication licensing factor MCM2	MCM2	1097800000	1357700000	1,30	1,07	0,09	6,743	0,1023222	0,1377659	0,05893986	0,08106696	0,09936258	0,1870075	-0,00878387	0,1032627	0,292075	0,2218153	0,009920342	0,1068843	6,31E-04	3,20	0,115969897	+
Proteasome subunit beta type-5	PSMB5	578350000	822540000	1,55	1,07	0,09	23,166	0,1055439	0,05255495	0,09936258	0,1763228	0,08691968	0,177854	0,01206797	0,008200003	0,02729555	0,05311137	-0,1190559	-0,06145073	6,55E-02	1,18	0,051560513	
ATPase ASNA1	ASNA1	67969000	82518000	1,51	1,07	0,09	13,091	0,02332628	0,1359287	NaN	0,0416635	NaN	0,2420842	0,02261638	0,08215749	NaN	NaN	NaN	NaN	4,75E-02	1,32	0,0912961	
Nucleosome assembly protein 1-like 4	NAP1L4	340920000	547260000	1,32	1,06	0,09	5,4498	0,04068203	0,06873958	0,03054793	0,04990952	0,1192898	0,1409095	0,03969989	NaN	NaN	NaN	0,03167748	0,05949366	1,20E-03	2,92	0,064549925	+
Bleomycin hydrolase	BLMH	220450000	288540000	1,15	1,06	0,09	13,181	0,09125975	0,03393393	0,1158324	0,02332628	0,1257833	0,1260478	NaN	NaN	NaN	NaN	0,08011194	0,1071523	4,37E-04	3,36	0,087930947	+
ATP-binding cassette sub-family E member 1	ABCE1	522540000	713570000	1,31	1,06	0,09	12,565	0,06722574	0,003889986	0,07806332	0,005615641	-0,001501157	0,000865329	0,122474	0,04012096	0,1782366	0,5953127	0,1377659	0,1365852	3,51E-02	1,45	0,113721182	
Importin subunit beta-1	KPNB1	1415900000	1897800000	1,35	1,06	0,09	9,3699	0,09477648	0,08379207	0,1260478	0,09125975	0,04334458	0,1004395	0,03815525	0,08732703	0,05644473	0,06225996	0,03773361	0,09491159	1,41E-06	5,85	0,076374363	+
Ribosome maturation protein SBDS	SBDS	48745000	64499000	1,28	1,06	0,09	8,5382	0,127105	-0,0216872	-0,1389498	0,1216786	0,08569661	0,2358496	NaN	NaN	-0,1427993	-0,09839744	-0,04668264	-0,2066451	8,57E-01	0,07	-0,008483161	
Regulator of nonsense transcripts 1	UPF1	566470000	647550000	1,19	1,06	0,09	11,844	-0,004653005	0,005902907	0,09734114	0,169412	-0,07404613	-0,01004722	0,03407487	0,241108	0,1756843	0,2016338	0,3053285	0,2440345	7,83E-03	2,11	0,115481148	+
Calnexin	CANX	1120500000	1498400000	1,39	1,06	0,08	14,729	-0,0302656	0,09855437	-0,01737322	-0,07632596	-0,04367569	-0,03836184	0,08949816	0,167358	0,1861201	0,2474415	0,3319345	0,2600257	3,36E-02	1,47	0,097910831	
Protein LZIC	LZIC	17380000	26856000	1,58	1,06	0,08	5,7254	0,229834	0,2006298	NaN	0,190678	0,2717842	0,241108	-0,04574413	0,07107621	NaN	NaN	0,04879416	0,04977013	5,27E-03	2,28	0,139770048	+
Glucosamine 6-phosphate N-acetyltransferase	GNPNAT1	23003000	33368000	1,52	1,06	0,08	8,3669	0,1979911	0,1804026	NaN	0,07038926	0,068602	0,1988712	-0,08969718	-0,1070883	-0,09169434	0,0275787	0,02346825	NaN	2,35E-01	0,63	0,047882331	
		106650000	158510000	1,38	1,06	0,08	20,891	0,1275012	0,07942928	0,07847318	0,2333973	0,1627257	0,2524157	0,09153062	0,1011122	0,04488368	0,01763692	0,08868441	0,01564032	4,47E-04	3,35	0,107785871	+
26S proteasome non-ATPase regulatory subunit 11	PSMD11	1140500000	1489300000	1,39	1,06	0,08	10,009	0,1845977	0,1636276	0,1138339	0,1259155	0,06377902	0,1184926	-0,08603258	-0,005289984	-0,07325665	0,03308822	0,1315895	-0,01057033	3,45E-02	1,46	0,06331454	
Isoleucine--tRNA ligase, cytoplasmic	IARS	1077000000	1325700000	1,25	1,06	0,08	11,024	0,07765318	0,1199537	0,09058251	0,08637615	0,1342209	0,1168971	-0,05749677	-0,1304287	-0,06704684	-0,07117868	0,06571014	-0,07485126	3,99E-01	0,40	0,024199287	
mRNA export factor	RAE1	73310000	83371000	1,72	1,06	0,08	17,57	NaN	-0,2444799	NaN	NaN	-0,04273913	-0,5098612	NaN	-0,02332847	0,09774555	0,1667154	NaN	NaN	4,02E-01	0,40	-0,092657974	
Acidic leucine-rich nuclear phosphoprotein 32 family member B	ANP32B	805720000	1208400000	1,56	1,06	0,08	21,049	0,1825652	0,175301	0,07354603	0,1102295	0,1722314	0,1644001	-0,03990347	-0,1119602	-0,06621589	-0,1364896	-0,02478071	-0,03515046	2,90E-01	0,54	0,038647742	
Serpin H1	SERPINH1	546290000	722970000	1,34	1,06	0,08	11,722	0,121148	0,1142337	0,1298765	0,09180144	0,02290045	0,111165	-0,3571783	-0,08638485	-0,1320719	0,09450638	0,1331689	0,1685137	4,55E-01	0,34	0,034306586	
26S protease regulatory subunit 8	PSMC5	330340000	450340000	1,36	1,06	0,08	26,557	0,1407786	0,2358496	0,08501676	0,1536757	0,02715413	0,1674864	0,2188431	-0,06923993	-0,09901539	-0,3715717	0,004033884	0,001730139	4,17E-01	0,38	0,041228451	
Prohibitin-2	PHB2	397740000	526150000	1,27	1,06	0,08	4,9935	0,06750099	-0,02550007	0,1575611	0,1178281	0,09477648	0,06818932	-0,06309262	-0,289263	-0,1970706	-0,03834701	0,2101395	0,004033884	8,35E-01	0,08	0,008896337	
Ubiquitin-conjugating enzyme E2 variant 2	UBE2V2	225590000	318570000	1,40	1,06	0,08	21,588	0,1418251	0,07573799	0,2609891	0,1115655	0,1032627	0,2382977	0,01264022	-0,001125722	0,04628156	0,05491792	-0,0662763	0,05074544	9,28E-03	2,03	0,085738439	+
Translin	TSN	272970000	389860000	1,36	1,06	0,08	6,2047	0,05672221	0,09774555	0,05755419	0,1463945	-0,01483479	0,1887806	0,02955887	-0,02397375	0,03365203	0,01877663	0,08338356	0,01049345	9,91E-03	2,00	0,057021089	+
SH3 domain-binding glutamic acid-rich-like protein	SH3BGRL	27966000	38053000	1,26	1,06	0,08	6,2204	-0,01655578	-0,01819122	0,08243007	0,1172961	0,1028596	0,165301	0,09518178	0,3300994	-0,1254942	0,1540647	0,2370741	0,3915478	1,22E-02	1,91	0,12630111	+
Cytoplasmic dynein 1 heavy chain 1	DYNC1H1	1100200000	1260400000	1,15	1,06	0,08	12,014	-0,01702287	0,08624023	-0,004030707	0,08188502	0,1138339	0,09949727	-0,02629326	0,02133754	0,06281248	0,05352838	-0,0151555	-0,06759103	8,05E-02	1,09	0,032420118	
Pyrroline-5-carboxylate reductase 2	PYCR2	187610000	263580000	1,33	1,06	0,08	10,789	0,2125071	0,0439044	0,06818932	0,1981169	0,1175621	0,1023222	0,0167816	-0,1412232	0,0984196	0,01235413	0,1063484	-0,003683579	2,80E-02	1,55	0,069299914	+
Protein-L-isoaspartate(D-aspartate) O-methyltransferase	PCMT1	469830000	686010000	1,51	1,06	0,08	6,7864	0,08379207	0,06681251	0,01792198	0,08133958	0,08990479	0,09828482	0,1301402	0,05686093	-0,00229573	0,01321208	0,06253617	0,05630613	1,40E-04	3,85	0,062901293	+
DNA replication licensing factor MCM4	MCM4	745620000	933080000	1,28	1,06	0,08	12,686	0,1103632	0,09410089	0,04474379	-0,006898093	0,01877663	0,08678382	0,01192487	0,1668439	0,06873958	0,03280615	0,04977013	0,1289532	9,15E-04	3,04	0,067242342	+
Protein SET;Protein SETSIP	SET;SETSIP	1531000000	2098600000	1,47	1,06	0,08	4,0993	0,05255495	0,2240403	0,02856912	0,2051422	0,009347181	0,1471764	0,03913844	0,06942715	0,05783146	0,07354603	NaN	NaN	4,04E-03	2,39	0,090677315	+

Synaptosomal-associated protein 29	SNAP29	39434000	49295000	1,31	1,05	0,08	11,931	-0,01281016	-0,04039304	-0,03583058	0,03520166	0,1749178	0,07313477	0,3561438	NaN	0,03674946	-0,7850794	0,16221	NaN	9,71E-01	0,01	-0,00357558	
Splicing factor 3A subunit 3	SF3A3	162700000	233110000	1,37	1,05	0,08	8,2331	0,255682	0,1445685	0,2683145	NaN	0,02885203	-0,0713151	NaN	NaN	NaN	NaN	0,04012096	0,08827728	6,11E-02	1,21	0,107785738	
Sorting nexin-3	SNX3	58210000	74630000	1,33	1,05	0,08	6,3656	0,02204824	0,04990952	-0,05505162	0,02034214	0,008343471	0,1060803	-0,358269	-0,07055745	NaN	-0,01696449	-0,001298968	-0,161621	2,99E-01	0,52	-0,041548988	
Ras-related protein Ral-A	RALA	22953000	35881000	1,42	1,05	0,08	15,754	0,03407487	NaN	-0,1659522	-0,01773837	0,06777637	-0,02602882	NaN	NaN	0,2340108	NaN	NaN	-0,21682	8,27E-01	0,08	-0,012953918	
Rho GDP-dissociation inhibitor 1	ARHGDIA	636250000	853330000	1,38	1,05	0,08	10,409	-0,01033776	0,03449741	0,06308875	0	-0,02227314	0,005184378	0,01735182	0,4079509	0,06322686	0,1259155	-0,06807495	0,2839217	8,67E-02	1,06	0,07503763	
Transcription elongation factor A protein 1	TCEA1	26969000	49088000	1,67	1,05	0,08	8,508	NaN	NaN	0,4627853	NaN	0,02290045	0,2872358	0,07038926	0,1805297	0,04502354	0,1950955	NaN	NaN	2,23E-02	1,65	0,180565666	+
Fascin	FSCN1	2010200000	2689300000	1,36	1,05	0,08	13,214	0,002450562	0,0880058	0,08678382	0,05185929	0,09855437	0,1339581	0,06018582	-0,01423726	NaN	NaN	0,005902907	0,108491	3,47E-03	2,46	0,062195435	+
Protein Hikeshi	C11orf73	9729000	12206000	1,60	1,05	0,08	12,448	NaN	NaN	-0,1501928	NaN	0,06488286	NaN	-0,05841288	0,1378969	0,04334458	0,1473066	0,196733	-0,2042829	6,81E-01	0,17	0,022159412	
Leucine-rich PPR motif-containing protein, mitochondrial	LRPPRC	3041200000	3659200000	1,24	1,05	0,08	8,7802	0,0615688	0,08963377	0,07272322	0,06295063	0,05241581	0,01578305	0,05699965	0,07327176	0,1222089	0,09815002	0,111432	0,08297509	1,96E-06	5,71	0,075009387	+
Diphosphomevalonate decarboxylase	MVD	82193000	98668000	1,20	1,05	0,07	20,729	0,1583371	-0,07614347	0,3283769	0,2327835	0,2235462	-0,2547713	NaN	NaN	-0,2400772	-0,2990987	NaN	NaN	9,22E-01	0,04	0,00911913	
Actin-related protein 2/3 complex subunit 4	ARPC4	195600000	267320000	1,39	1,05	0,07	11,047	0,01135251	0,09166604	0,170566	0,08188502	0,02275842	0,08243007	0,07135073	0,1166309	0,01049345	0,0278618	0,1095611	0,02219017	6,54E-04	3,18	0,068228856	+
Thimet oligopeptidase	THOP1	68704000	86179000	1,33	1,05	0,07	24,726	0,0265876	0,04781753	0,04306451	0,0500489	0,2253984	0,04544289	0,1527676	NaN	NaN	-0,05154864	NaN	NaN	5,82E-02	1,23	0,067447349	
Signal recognition particle receptor subunit beta	SRPRB	85844000	110080000	1,40	1,05	0,07	4,1607	-0,02947023	0,06087747	0,009490535	0,03576464	-0,03112038	-0,02843987	0,3278023	0,3807295	0,2679552	0,3452829	0,4049031	0,3363976	7,27E-03	2,14	0,173347733	+
Tubulin-specific chaperone E	TBCE	60701000	86777000	1,31	1,05	0,07	9,9209	0,04725923	0,1414329	-0,0602619	0,1194226	NaN	0,1036654	NaN	NaN	NaN	NaN	0,1263122	-0,1040614	2,00E-01	0,70	0,053395565	
RuvB-like 2	RUVBL2	736740000	1198200000	1,32	1,05	0,07	6,0723	0,1382902	0,1194226	0,145221	0,08854871	-0,04999452	-0,007057603	0,09071802	-0,00050503	NaN	NaN	0,0321009	-0,0456101	5,84E-02	1,23	0,051113421	
Proteasome subunit beta type-1	PSMB1	415230000	568070000	1,41	1,05	0,07	8,6539	0,05283319	0,08773442	-0,04980037	0,004752986	0,03885755	0,07107621	0,1357976	-0,05153375	-0,01490768	0,08215749	-0,02155539	0,07231157	7,52E-02	1,12	0,033976982	
Transportin-3	TNPO3	75220000	97785000	1,27	1,05	0,07	14,45	0,100574	0,1755566	0,1660727	0,1015157	0,1296127	0,2159891	0,133432	0,006333955	NaN	0,01192487	NaN	0,1192898	3,74E-04	3,43	0,116030128	+
Aldehyde dehydrogenase X, mitochondrial	ALDH1B1	141870000	196470000	1,31	1,05	0,07	11,563	0,1141004	0,04026129	0,02091112	0,04572251	0,1278974	0,05783146	NaN	0,3000651	NaN	NaN	0,2828548	0,3080114	6,86E-03	2,16	0,144183934	+
Platelet-activating factor acetylhydrolase IB subunit gamma	PAFAH1B3	163920000	222090000	1,45	1,05	0,07	9,3226	0,05241581	-0,000202008	0,03054793	0,07327176	0,07942928	0,005328146	0,1915626	0,04907312	-0,01997472	0,2566484	0,05311137	0,4560701	2,52E-02	1,60	0,102273481	+
Proteasome activator complex subunit 1	PSME1	87499000	122360000	1,43	1,05	0,07	10,832	0,193961	-0,08710491	0,06763869	0,1107641	0,0766959	0,1959774	NaN	-0,1447916	-0,07349949	-0,1096078	0,05505682	-0,05860819	5,85E-01	0,23	0,020589268	
14-3-3 protein zeta/delta	YWHAZ	3469200000	5082100000	1,55	1,05	0,07	11,247	0,06708801	0,1199537	0,09855437	0,1414329	0,1062144	0,165301	0,0292761	0,04781753	0,04292445	0,004896797	-0,00955338	0,0207689	1,28E-03	2,89	0,06955622	+
Phosphatidylethanolamine-binding protein 1;Hippocampal cholinergic neurostimulating peptide	PEBP1	642900000	942940000	1,52	1,05	0,07	7,1033	0,100574	0,07929271	0,05852425	0,038998	0,1146336	0,01920368	0,07956584	-0,02145292	0,01735182	0,08147596	0,1566554	0,02800333	1,04E-03	2,98	0,062735468	+
Lupus La protein	SSB	1401400000	1813300000	1,30	1,05	0,07	11,527	0,06060078	0,02814484	0,1176951	0,09166604	0,07532719	0,02701252	0,1115655	0,09193667	0,1977397	0,09356022	-0,03717705	-0,04400288	5,09E-03	2,29	0,067839052	+
Serine/threonine-protein phosphatase 5	PPP5C	84459000	111270000	1,33	1,05	0,07	7,989	0,2773897	0,1130337	0,1326427	0,03083044	0,05769283	0,1194226	NaN	-0,2441724	NaN	NaN	-0,5798356	-0,4666758	5,47E-01	0,26	-0,062185766	
Metaxin-2	MTX2	26887000	39968000	1,61	1,05	0,07	10,454	0,1257833	0,08732703	NaN	0,1815478	0,03407487	0,1513393	NaN	NaN	0,1616942	NaN	-0,0963292	NaN	4,55E-02	1,34	0,092205332	
Peptidyl-prolyl cis-trans isomerase A;Peptidyl-prolyl cis-trans isomerase A, N-terminally processed	PPIA	4544200000	6859400000	1,37	1,05	0,07	17,729	0,004033884	0,05408442	0,0494913	0,09734114	0,02856912	0,01278325	0,07860982	0,1346153	0,0426443	0,1055439	0,1106305	0,1198209	1,82E-04	3,74	0,069847318	+
Flap endonuclease 1	FEN1	390230000	533810000	1,28	1,05	0,07	6,4841	0,1234014	0,06060078	0,2789363	0,1116991	-0,02240502	0,02842765	0,01991543	0,1938348	0,1175621	0,1746621	0,1060803	0,1227389	7,63E-04	3,12	0,109621154	+
TAR DNA-binding protein 43	TARDBP	202950000	262140000	1,51	1,05	0,07	16,818	0,09139519	0,1282935	0,1467856	0,04292445	-0,01722728	0,03731201	0,1781091	-0,1036119	0,01106615	-0,02985311	NaN	NaN	1,15E-01	0,94	0,048519363	
39S ribosomal protein L44, mitochondrial	MRPL44	13213000	19517000	1,27	1,05	0,07	6,3834	-0,01959445	0,09599187	0,0734089	0,0278618	0,02941749	NaN	NaN	0,1031283	1,082089	NaN	0,08133958	NaN	1,97E-01	0,71	0,18420537	
Ubiquitin-conjugating enzyme E2 N;Putative ubiquitin-conjugating enzyme E2 N-like	UBE2N;UBE2NL	267760000	356750000	1,34	1,05	0,07	12,833	-0,01266459	0,07628538	0,1072862	0,2729787	0,0586628	0,02715413	0,05949366	-0,07477527	-0,03655548	-0,05072657	-0,166616	-0,1010874	6,96E-01	0,16	0,013286296	
Protein NipSnap homolog 3A	NIPSNAP3A	27804000	39924000	1,53	1,05	0,07	10,977	0,1259155	0,2901885	NaN	0,1547128	0,5633534	0,2407417	NaN	NaN	NaN	-0,4042291	NaN	-0,5854747	7,32E-01	0,14	0,055029729	
Calcyclin-binding protein	CACYBP	877810000	1208700000	1,45	1,05	0,07	9,7699	0,163241	0,0586628	0,01077991	0,09504669	0,07217448	0,09437123	0,115566	0,03280615	-0,002367994	-0,000476127	-0,1030075	-0,05586124	9,20E-02	1,04	0,040077957	
THO complex subunit 6 homolog	THOC6	52628000	85464000	1,57	1,05	0,07	7,2386	0,05949366	0,1914362	0,2459823	0,2393981	-0,173238	NaN	0,08379207	0,2022611	0,07860982	0,2115106	0,18954	0,02587931	8,52E-03	2,07	0,123151385	+
Nucleoprotein TPR	TPR	492330000	536770000	1,17	1,05	0,07	16,043	-0,01528672	0,05185929	0,1501697	0,2032638	-0,01347983	-0,01043953	-0,009567836	0,1142337	-0,1581718	0,01849174	-0,04192198	0,002738459	3,96E-01	0,40	0,024324078	
U6 snRNA-associated Sm-like protein LSm8	LSM8	45558000	51105000	1,53	1,05	0,07	6,3191	0,2892443	0,1108978	NaN	NaN	NaN	NaN	0,1260478	-0,01184981	-0,04622077	-0,08718155	-0,005304398	0,05255495	2,49E-01	0,60	0,053523532	
Lysine-specific demethylase 3B	KDM3B	27941000	26993000	1,11	1,05	0,07	15,289	NaN	-0,1197926	0,02772026	-0,4096423	-0,01349442	0,06915226	1,578504	NaN	NaN	0,5069066	NaN	0,3102238	2,92E-01	0,54	0,243697192	
Moesin	MSN	2993000000	4040300000	1,38	1,05	0,07	18,849	-0,02902857	0,000576887	0,1050075	0,1055439	0,002306507	-0,01664337	0,1134338	0,1483484	0,169925	0,1003049	0,05533457	0,1119661	3,44E-03	2,46	0,072256314	+
Small glutamine-rich tetratricopeptide repeat-containing protein alpha	SGTA	75242000	112110000	1,38	1,05	0,07	12,215	0,04753833	0,1062144	0,07121356	0,1470462	0,3154503	0,03604614	0,0878702	0,009777201	0,1863738	-0,02000393	0,2782227	0,02587931	4,75E-03	2,32	0,107635681	+
Ras-related protein Rab-14	RAB14	197600000	265460000	1,36	1,05	0,07	9,6751	-0,02913166	0,04208399	0,09963177	-0,01228624	0,08460858	0,177599	-0,3245272	-0,6427746	-0,9574677	-0,3433114	NaN	-0,7545287	6,75E-02	1,17	-0,241827665	
C-terminal-binding protein 1	CTBP1	28483000	35018000	1,59	1,05	0,07	13,734	NaN	-0,01884875	NaN	NaN	NaN	0,2208253	-0,04818847	NaN	0,01006364	0,1098286	NaN	NaN	3,29E-01	0,48	0,05473605	
Proteasome subunit alpha type-7	PSMA7	718890000	1013900000	1,45	1,05	0,07	7,6807	0,1142337	0,07997543	0,06736329	0,07409441	0,1519886	0,1479579	-0,01797205	-0,07520081	-0,06609508	-0,01662876	-0,06208313	0,06695026	1,34E-01	0,87	0,038715318	
10 kDa heat shock protein, mitochondrial	HSPE1	258130000	362620000	1,44	1,05	0,07	8,6951	0,07354603	0,03266509	0,1240634	0,08759864	0,0984196	-0,01565126	0,265677	0,1798932	-0,007811666	0,05185929	0,03731201	0,02941749	5,56E-03	2,25	0,079749065	+
Exportin-2	CSE1L	2003500000	3046000000	1,28	1,05	0,07	9,7814	0,08909126	0,1156992	0,02800333	0,0745054	0,09423606	0,13711	-0,04578878	-0,04574413	-0,05677631	-0,02592594	-0,05265548	-0,04462767	3,27E-01	0,49	0,022260575	
Leucine-rich repeat-containing protein 47	LRRC47	114480000	145230000	1,45	1,05	0,07	11,441	-0,3464263	-0,09946353	-0,1080987	-0,08679849	-0,3331525	-0,04511876	-0,01218434	0,09558696	0,05241581	0,1508197	0,09504669	0,1164979	3,80E-01	0,42	-0,043406303	
Nuclear cap-binding protein subunit 1	NCBP1	34737000	45425000	1,30	1,05	0,07	20,577	0,3742884	0,09369545	NaN	0,2294649	NaN	0,127105	NaN	-0,2136043	0,06322686	0,03689012	-0,7029847	-1,409317	4,30E-01	0,37	-0,155692764	
F-actin-capping protein subunit beta	CAPZB	146590000	194370000	1,37	1,05	0,07	23,586	0,1476974	-0,02212661	-0,1281721	0,09234277	0,1214134	0,1532865	0,02970023	0,1001703	0,2502339	0,1523782	0,005040595	0,7917724	5,49E-02	1,26	0,14114475	
SNARE-associated protein Snapin	SNAPIN	14188000	21437000	1,48	1,05	0,07	0,62383	0,07792662	0,03857661	0,09855437	0,1378969	-0,02724856	0,004752986	0,1304037	0,2751263	0,1428708	0,3162616	0,2843957	0,2525368	1,14E-03	2,94	0,144337829	+
Triosephosphate isomerase	TPI1	2753400000	3982400000	1,41	1,05	0,06	7,6272	0,02133754	-0,01816196	0,0615688	0,07176267	0,09356022	0,08460858	0,07162537	0,06087747	0,03984026	0,02247432	0,122474	0,1306673	3,23E-04	3,49	0,063552882	+
Heterogeneous nuclear ribonucleoprotein U-like protein 1	HNRNPUL1	75599000	93712000	1,10	1,05	0,06	12,485	0,02147971	-0,01362549	0,2726204	0,0551957	0,02800333	0,01206797	NaN	0,1601458	NaN	NaN	0,2717842	0,4306065	2,91E-02	1,54	0,137586444	
3-mercaptopyruvate sulfurtransferase	MPST	16480000	24607000	1,52	1,05	0,06	18,033	0,2555611	NaN	0,3210045	0,4946716	0,5618882	NaN	NaN	NaN	-0,203668	-0,235433	-0,2102457	-0,2371494	4,75E-01	0,32	0,093328655	
Alpha-enolase	ENO1	17465000000	2,323E+10	1,33	1,05	0,06	6,5149	-0,0117189	0,007482617	-0,006550274	-0,01305762	-0,04712974	-0,0151701	0,1806571	0,1792563	0,3052118	0,2000021	0,1158324	0,1009776	2,79E-02	1,55	0,082982763	+
NAD(P)H-hydrate epimerase	APOA1BP	68104000	104480000	1,53	1,05	0,06	4,0081	0,02375215	0,07915612	0,1092936	0,1187585	0,08093054	0,12075	0,03280615	0,06571014	0,07778991	0,09193667	0,08664794	0,03703076	4,95E-06	5,31	0,07704687	+
ATP synthase subunit g, mitochondrial	ATP5L	144490000	208960000	1,47	1,04	0,06	9,9093	0,04809668	0,1463945	-0,1413506	0,1125	-0,07591531	-0,0580073	0,1367164	0,131326	0,06267433	0,06777637	0,05644473	0,07354603	1,05E-01	0,98	0,046683488	
Dynamin-2	DNM2	88945000	120000000	1,29	1,04	0,06	11,125	0,2584586	0,2286032	0,2498701	0,07038926	0,1125	0,009060428	0,09788039	NaN	NaN	NaN	0,1647863	-0,141271	2,66E-02	1,58	0,11669748	+
RuvB-like 1	RUVBL1	527590000	733410000	1,31	1,04	0,06	12,566	0,1351408	0,1071523	0,1749178	0,153935	0,0446039	0,0116388	0,1162316	0,1163647	NaN	NaN	0,07464243	0,05241581	1,95E-04	3,71	0,098704306	+
Serine/threonine-protein phosphatase 2A activator	PPP2R4	33702000	56025000	1,56	1,04	0,06	19,019	0,03731201	0,2433034	NaN	NaN	0,1236661	0,1310627	-0,1035809	0,2276179	-0,07684319	0,01278325	NaN	NaN	1,48E-01	0,83	0,074415156	
ATP synthase subunit d, mitochondrial	ATP5H	147790000	209210000	1,38	1,04	0,06	5,2828	0,08732703	0,116764	0,09004036	0,07025199	0,04474379	0,1484786	0,05450115	-0,1671991	-0,01721266	0	-0,06217354	0,07601179	1,70E-01	0,77	0,036794453	
C-1-tetrahydrofolate synthase, cytoplasmic;Methylenetetrahydrofolate dehydrogenase;Methenyltetrahydrofolate cyclohydrolase;Formyltetrahydrofolate synthetase;C-1-tetrahydrofolate synthase, cytoplasmic, N-terminally processed	MTHFD1	638570000	818980000	1,28	1,04	0,06	10,695	0,09031146	0,153027	0,0244615	0,10165	0,003602319	0,1025909	0,0650208	0,08637615	0,08392822	-0,007753654	-0,09141763	-0,2075611	2,67E-01	0,57	0,033686333	
Tropomyosin alpha-3 chain	TPM3	265380000	347460000	1,13	1,04	0,06	10,545	0,02559582	-0,03054558	0,04684024	-0,01203892	-0,005666505	0,02162186	0,1548424	0,1104968	0,05616735	0,1460035	0,07888308	0,07915612	8,26E-03	2,08	0,055946347	+
Elongation factor Tu, mitochondrial	TUFM	1855100000	2479200000	1,35	1,04	0,06	15,509	0,1600167	0,1216786	0,1519886	0,1589833	0,04558271	0,0911243	-0,1547469	-0,1816403	0,3645725	0,3561438	-0,05448222	0,05464008	8,54E-02	1,07	0,092821763	
Ataxin-10	ATXN10	97638000	129230000	1,35	1,04	0,06	9,4167	0,009347181	0,08705537	-0,1928925	0,0311129	-0,03337759	0,005471901	0,2950176	0,2415961	0,3148704	0,199374	0,04278438	-0,1458926	1,59E-01	0,80	0,071205603	
Eukaryotic initiation factor 4A-III;Eukaryotic initiation factor 4A-III, N-terminally processed	EIF4A3	209110000	293520000	1,33	1,04	0,06	8,737	0,2657969	0,1851053	0,3937456	0,2972502	-0,01198073	-0,0120971	-0,1469784	-0,4252106	NaN	NaN	0,01991543	0,06004741	4,30E-01	0,37	0,062559392	
Cytosolic non-specific dipeptidase	CNDP2	202710000	280240000	1,26	1,04	0,06	16,755	-0,1089386	-0,1294972	-0,05311942	0,01877663	0,05283319	-0,0391473	0,03181863	-0,04181805	NaN	NaN	0,215368	0,1218111	8,42E-01	0,07	0,006808696	
ATP-dependent 6-phosphofructokinase, platelet type	PFKP	125710000	160670000	1,27	1,04	0,06	13,588	-0,09642179	0,08990479	-0,000302979	0,1724875	-0,1044337	0,1282935	0,005184378	-0,5293642	0,1373724	0,0247452	NaN	0,1180938	9,35E-01	0,03	-0,004949194	
Ubiquitin carboxyl-terminal hydrolase 7	USP7	136430000	160150000	1,10	1,04	0,05	9,573	0,03660879	0,0504668	0,08174868	0,1071523	-0,04902405	0,08624023	-0,05856314	-0,06214337	-0,01234443	0,03040665	0,06446896	-0,05641617	3,38E-01	0,47	0,018216767	
Casein kinase II subunit beta	CSNK2B	56662000	78848000	1,51	1,04	0,05	15,872	0,08583257	-0,009872919	0,108491	NaN	-0,0740006	-0,0106139	0,02941749	0,1200864	NaN	0,01963077	0,02885203	0,07956584	8,18E-02	1,09	0,037738868	
Obg-like ATPase 1	OLA1	983410000	1323700000	1,35	1,04	0,05	12,297	0,177599	0,1280294	0,07409441	-0,03913245	0,04544289	0,03139513	-0,07339326	-0,06069816	NaN	NaN	0,0182068	0,09410089	1,62E-01	0,79	0,039564466	
26S proteasome non-ATPase regulatory subunit 3	PSMD3	427010000	556200000	1,30	1,04	0,05	6,0082	0,0615688	0,04935203	0,1600167	0,07860982	0,1735112	0,08773442	0,000865329	-0,04495508	-0,1773922	-0,1386322	-0,08952827	-0,1375526	9,55E-01	0,02	0,001966497	
Peroxiredoxin-1	PRDX1	2774000000	4162600000	1,40	1,04	0,05	10,084	-0,05997609	0,005184378	-0,04896435	0,01206797	-0,003466602	0,0451634	0,1908044	0,06018582	0,1861201	0,03913844	0,2001277	0,2235462	3,48E-02	1,46	0,070827614	
Cystathionine beta-synthase	CBS	135360000	203200000	1,27	1,04	0,05	17,837	0,004465491	0,003170453	0,02460336	0,01906128	0,07819984	0,108491	-1,081431	-0,7793976	NaN	NaN	-0,2761357	-0,1082386	1,52E-01	0,82	-0,20072111	
Tubulin-specific chaperone A	TBCA	315670000	382170000	1,23	1,04	0,05	11,044	-0,01119537	-0,04504441	-0,08598667	0,2106382	-0,02810146	0,1090262	-0,1153787	0,04194384	NaN	NaN	0,09855437	NaN	5,94E-01	0,23	0,019384007	
COP9 signalosome complex subunit 4	COPS4	179260000	233660000	1,32	1,04	0,05	10,923	0,1656869	0,2032638	0,08011194	0,1071523	0,07423148	0,1102295	-0,1371241	NaN	-0,1667455	-0,2229528	NaN	NaN	6,61E-01	0,18	0,023761487	
GTP-binding protein SAR1a	SAR1A	223580000	321600000	1,40	1,04	0,05	5,9025	0,08596853	0,0939657	0,03449741	0,152508	0,03717139	0,06873958	-0,04009629	-0,06239953	0,002738459	-0,004522744	-0,05409274	-0,01282465	2,13E-01	0,67	0,025137758	
Purine nucleoside phosphorylase	PNP	255080000	358080000	1,40	1,04	0,05	11,82	0,1728715	0,162081	0,1285573	0,1355349	0,1547128	0,1471764	-0,1004685	-0,1874443	-0,1483689	-0,1632517	-0,2592365	-0,1160977	9,01E-01	0,05	-0,00616114	
Acetyl-CoA acetyltransferase, mitochondrial	ACAT1	854230000	1313600000	1,54	1,04	0,05	26,783	0,4532278	0,4483725	0,4188915	0,2867629	0,3355975	0,3636757	-0,08555793	-0,1535909	-0,08223987	-0,1178343	NaN	NaN	4,98E-02	1,30	0,186730487	
Calcium-binding protein 39	CAB39	44178000	57238000	1,30	1,03	0,05	16,746	-0,06170668	0,2309407	NaN	0,001730139	-0,006898093	0,04963072	NaN	NaN	0,08691968	0,007195495	NaN	NaN	2,65E-01	0,58	0,043973142	
tRNA pseudouridine synthase A, mitochondrial	PUS1	81234000	108540000	1,46	1,03	0,05	5,4383	0,2348692	-0,02277135	0,02687089	-0,04766648	0,0542234	0,04921259	NaN	NaN	-0,06872541	-0,1396331	NaN	NaN	7,92E-01	0,10	0,010797469	
Ubiquitin carboxyl-terminal hydrolase isozyme L3	UCHL3	68982000	99837000	1,43	1,03	0,05	11,315	0,005040595	0,1190241	0,05352838	0,03689012	0,3491388	0,04502354	NaN	0,1735112	0,1579492	0,07038926	0,0615688	0,1020534	4,07E-03	2,39	0,10673794	+
U6 snRNA-associated Sm-like protein LSm2	LSM2	44552000	61143000	1,32	1,03	0,05	7,4355	0,1386835	0,09450638	0,000144286	0,1103632	-0,1676527	-0,04199625	0,008486926	0,1300083	-0,03808034	-0,05159355	-0,03098771	-0,08462442	8,42E-01	0,07	0,005604802	
Dolichyl-diphosphooligosaccharide--protein glycosyltransferase subunit DAD1	DAD1	68681000	94811000	1,39	1,03	0,05	10,391	0,1589833	-0,1218789	0,1216786	0,08351974	0,1826923	0,1703097	-0,2771143	0,1741506	0,09626186	0,133432	0,07601179	0,1192898	8,06E-02	1,09	0,07644471	
WD repeat-containing protein 82	WDR82	15833000	25356000	1,40	1,03	0,05	8,2634	0,3312466	0,04809668	NaN	NaN	0,103397	NaN	NaN	NaN	0,08705537	0,03717139	0,1290852	0,1096949	1,76E-02	1,76	0,12082102	+
Serine/threonine-protein phosphatase 2A 65 kDa regulatory subunit A alpha isoform	PPP2R1A	868300000	1082800000	1,34	1,03	0,05	10,33	0,1092936	0,0359054	-0,008217905	-0,002873853	0,07354603	0,01991543	-0,02198018	0,08556063	0,05741553	0,09558696	-0,0519524	-0,07860939	1,62E-01	0,79	0,026132489	
Importin subunit alpha-7	KPNA6	43662000	54561000	1,24	1,03	0,05	12,985	0,1347467	0,05144163	0,08841301	0,02715413	0,2102642	0,06322686	NaN	NaN	NaN	NaN	-0,09483393	0,1740229	4,52E-02	1,34	0,081804442	
Alpha-actinin-1	ACTN1	41459000	54467000	1,38	1,03	0,05	19,105	0,04586229	0,3354833	0,230449	0,2308178	NaN	0,1623389	-0,2180948	0,01435528	-0,1057681	-0,2522431	-0,1577371	-0,3509825	9,33E-01	0,03	-0,005956274	
Delta(3,5)-Delta(2,4)-dienoyl-CoA isomerase, mitochondrial	ECH1	46859000	58315000	1,29	1,03	0,05	9,7036	0,04404436	0,01863419	0,1674864	0,153027	0,1678717	-0,2510065	NaN	-0,2566488	-0,08402792	-0,1082698	0,003602319	0,05102402	8,56E-01	0,07	-0,008569359	
SAP domain-containing ribonucleoprotein	SARNP	19032000	24411000	1,42	1,03	0,05	11,47	NaN	-0,2036847	NaN	0,2897164	NaN	NaN	0,03632757	NaN	-0,1887756	-0,06843793	0,1025909	0,1403861	8,28E-01	0,08	0,015446099	
DNA replication licensing factor MCM7	MCM7	903360000	1129000000	1,25	1,03	0,05	15,989	0,02019986	0,05672221	0,1198209	-0,01534504	0,04502354	0,05741553	0,05630613	0,1359287	-0,3852054	0,12075	0,07997543	0,262794	3,18E-01	0,50	0,046198822	
Cleavage and polyadenylation specificity factor subunit 6	CPSF6	87866000	110830000	1,23	1,03	0,04	23,209	0,3296403	0,3627785	NaN	0,2203299	NaN	-0,103922	NaN	NaN	NaN	NaN	0,1103632	0,1747899	4,59E-02	1,34	0,182329973	
Actin-like protein 6A	ACTL6A	107150000	140240000	1,43	1,03	0,04	9,3142	0,3248107	0,2335199	0,1585955	0,1419559	0,01792198	0,02389408	0,09585687	0,1595	NaN	NaN	0,05158086	-0,191442	4,76E-02	1,32	0,101619376	

Splicing factor U2AF 35 kDa subunit;Splicing factor U2AF 26 kDa subunit	U2AF1;U2AF1L4	59908000	93660000	1,34	1,03	0,04	2,6543	0,06557227	0,116764	0,1613072	0,1839629	-0,1172396	-0,02445785	-0,01073016	0,1697967	0,005471901	0,01120934	NaN	NaN	1,09E-01	0,96	0,056165674	
Peroxiredoxin-6	PRDX6	2329200000	3358800000	1,42	1,03	0,04	10,904	0,02743714	0,03773361	0,03322923	0,02389408	0,05018827	0,06004741	0,07066408	0,03829577	0,02247432	0,0551957	0,05893986	0,05380643	1,04E-06	5,98	0,044325492	+
Proteasome assembly chaperone 1	PSMG1	54435000	51902000	1,26	1,03	0,04	8,5339	0,04823624	-0,02727792	0,03040665	0,06695026	0,1419559	0,1071523	-0,06280626	-0,02351908	-1,431365	-2,336865	NaN	NaN	2,21E-01	0,65	-0,348713157	
SUMO-conjugating enzyme UBC9	UBE2I	86640000	106840000	1,30	1,03	0,04	8,0234	0,1042025	-0,01305762	0,221939	0,120352	-0,1050852	-0,0750488	0,1811662	0,2589409	NaN	NaN	0,2097652	0,06832678	3,89E-02	1,41	0,097150098	
Ras-related protein Rab-7a	RAB7A	483070000	742740000	1,44	1,03	0,04	7,4081	-0,06069816	-0,002266791	0,01920368	0,02332628	0,0542234	0,02587931	0,1012467	0,1488691	0,07737969	0,02715413	0,08474465	0,01035019	2,12E-02	1,67	0,042451013	+
Protein phosphatase methylesterase 1	PPME1	86765000	108100000	1,28	1,03	0,04	10,481	-0,05913416	0,245374	-0,1785836	0,0416635	0,01106615	0,03421579	NaN	0,14496	0,3613192	NaN	NaN	NaN	2,54E-01	0,59	0,075110108	
Uroporphyrinogen decarboxylase	UROD	377780000	481210000	1,30	1,03	0,04	16,531	0,09585687	0,03576464	0,0939657	0,1013812	0,05283319	0,09612688	-0,3404772	-0,5208936	-0,3138715	-0,2946148	NaN	NaN	2,22E-01	0,65	-0,099392871	
Phosphoglycerate kinase 1	PGK1	4930600000	6000700000	1,52	1,03	0,04	11,745	-0,1552608	-0,1318506	-0,06831692	-0,03361381	-0,1031625	-0,09966448	0,2065182	0,2268784	0,270828	0,2576141	NaN	0,2703495	3,18E-01	0,50	0,058210837	
Profilin-1	PFN1	2462700000	3486600000	1,45	1,03	0,04	5,7167	-0,04990488	-0,04181805	-0,002960535	0,03435652	0,02517066	0,1191569	0,0494913	0,1112984	0,01292609	0,04194384	0,05907837	0,01849174	5,75E-02	1,24	0,031435866	
Acidic leucine-rich nuclear phosphoprotein 32 family member A	ANP32A	494700000	706080000	1,35	1,03	0,04	8,9997	0,09301919	0,02941749	0,05713834	0,05158086	0,09693644	0,05505682	-0,05658124	-0,04791998	-0,03953276	0,05575094	-0,03922143	-0,01222805	2,37E-01	0,62	0,020284719	
Signal recognition particle 14 kDa protein	SRP14	65421000	94356000	1,22	1,03	0,04	15,738	-0,2895274	-0,3353897	-0,2350764	-0,01355266	0,05699965	-0,1495206	0,3069621	0,2723816	0,2696317	0,2110122	0,3537749	0,2516888	4,44E-01	0,35	0,058282019	
DNA replication licensing factor MCM6	MCM6	917940000	1099200000	1,19	1,03	0,04	11,197	0,09369545	0,0292761	0,007626157	-0,01374199	-0,02102828	0,1164979	-0,1550681	-0,1314713	0,1241958	0,06073913	-0,01065748	-0,1200904	9,52E-01	0,02	-0,001668915	
Serine hydroxymethyltransferase, mitochondrial	SHMT2	760140000	863000000	1,28	1,03	0,04	24,702	0,08447251	-0,04620585	0,1749178	0,03562387	0,131326	-0,01167521	-0,2797566	-0,1664865	NaN	NaN	-0,1339229	-0,1401735	4,63E-01	0,33	-0,035188032	
Splicing factor U2AF 65 kDa subunit	U2AF2	189400000	249480000	1,18	1,03	0,04	18,244	0,2027625	-0,1010565	0,1708224	-0,01506799	-0,08859219	-0,08776401	0,5173261	0,3999913	0,4342412	0,262794	0,347212	0,2224336	1,22E-02	1,91	0,188758541	+
14-3-3 protein gamma;14-3-3 protein gamma, N-terminally processed	YWHAG	478490000	671670000	1,32	1,03	0,04	6,0371	0,02984157	0,008917032	-0,007956751	0,09193667	-0,06589875	0,04670055	0,1071523	0,1847246	0,1929516	0,1019189	0,1701815	0,1802752	4,85E-03	2,31	0,086728712	+
Alpha-centractin;Beta-centractin	ACTR1A;ACTR1B	182340000	220620000	1,30	1,03	0,04	12,073	0,163241	0,1513393	0,1241958	-0,05029322	-0,02116004	0,1096949	-0,101041	NaN	NaN	NaN	-0,01976993	-0,008812854	2,73E-01	0,56	0,038599328	
Heme oxygenase 2	HMOX2	20955000	21545000	1,23	1,03	0,04	14,876	0,04278438	0,09423606	0,2444	-0,09643723	NaN	0,07970238	-0,2330572	-0,2341937	-0,2198243	-0,1356653	NaN	NaN	4,02E-01	0,40	-0,050894984	
Very-long-chain enoyl-CoA reductase	TECR	58514000	69672000	1,21	1,03	0,04	17,564	NaN	0,08963377	0,3442604	-0,0706483	0,1212807	-0,04495508	NaN	0,11703	0,06598585	0,08488071	NaN	NaN	8,73E-02	1,06	0,088433511	
2-deoxynucleoside 5-phosphate N-hydrolase 1	DNPH1	105890000	146390000	1,36	1,03	0,04	13,16	0,07066408	-0,1659846	0,3852654	NaN	0,1471764	0,1513393	-0,1821476	-0,1003912	-0,1248176	NaN	NaN	-0,2713741	8,90E-01	0,05	-0,010030008	
DNA polymerase epsilon subunit 3	POLE3	40727000	43106000	1,09	1,02	0,04	16,524	0,2167342	-0,2486391	-0,269008	-0,3259191	0,1376347	-0,1158944	-0,03584539	0,005759195	NaN	0,0321009	NaN	NaN	3,15E-01	0,50	-0,06700855	
BRCA2 and CDKN1A-interacting protein	BCCIP	136210000	165710000	1,31	1,02	0,03	4,8561	0,0441843	0,03125411	0,03745244	-0,01168977	-0,09101805	-0,02152615	-0,04968092	-0,03460356	NaN	-0,1937337	-0,08479267	NaN	1,38E-01	0,86	-0,037415396	
Cytochrome c	CYCS	375450000	524380000	1,35	1,02	0,03	12,358	0,1257833	0,1496496	0,1573025	0,1138339	-0,001385642	0,1007085	-0,152885	-0,05755682	-0,1649814	-0,05470692	-0,0614056	-0,07287727	8,45E-01	0,07	0,006789929	
Phosphatidylinositol transfer protein beta isoform	PITPNB	27061000	38452000	1,26	1,02	0,03	10,514	-0,006144506	-0,05707642	0,04320455	0,1037997	0,1329059	0,05810868	NaN	-0,1434684	0,1779815	0,02275842	NaN	-0,03717705	3,55E-01	0,45	0,029489243	
Microtubule-associated protein RP/EB family member 1	MAPRE1	201820000	266380000	1,24	1,02	0,03	13,241	0,103397	0,1344838	0,2529	0,1685137	-0,08013366	0,01621099	-0,134778	-0,04589308	0,003602319	-0,07740634	0,004321636	-0,06845307	5,11E-01	0,29	0,023063778	
MICOS complex subunit MIC19	CHCHD3	160780000	207030000	1,34	1,02	0,03	15,113	0,04572251	0,07189995	-0,042858	0,05533457	0,03083044	0,03393393	NaN	NaN	0,0278618	-0,02104294	-0,1968722	NaN	9,85E-01	0,01	0,000534451	
Proteasome subunit alpha type-5	PSMA5	674290000	902880000	1,39	1,02	0,03	4,8838	0,006477609	0,01849174	-0,05820255	0,000865329	0,01877663	0,04851514	-0,01454325	-0,2710956	-0,2048482	-0,04510394	-0,04444915	-0,0719974	8,80E-02	1,06	-0,051426133	
COP9 signalosome complex subunit 6	COPS6	17625000	22363000	1,28	1,02	0,03	17,926	0,1662012	-0,133907	-0,2509034	NaN	-0,1952525	NaN	NaN	NaN	0,06170706	-0,07678234	NaN	-0,2726284	1,56E-01	0,81	-0,100223631	
Protein deglycase DJ-1	PARK7	624350000	877580000	1,36	1,02	0,03	7,6024	0,03506084	-0,02514765	-0,0139896	0,001442041	-0,01192247	-0,008435511	0,09234277	0,01521204	-0,01692064	0,02587931	0,07025199	0,0618453	1,21E-01	0,92	0,018801536	
3-hydroxyacyl-CoA dehydrogenase type-2	HSD17B10	563090000	793050000	1,48	1,02	0,03	5,434	0,09545191	0,009347181	0,06750099	0,02941749	0,06681251	0,03421579	0,01891905	-0,04663788	-0,02004788	-0,02926408	0,04628156	-0,03173962	1,52E-01	0,82	0,020021419	
Peptidyl-prolyl cis-trans isomerase NIMA-interacting 1	PIN1	34140000	39522000	1,26	1,02	0,03	14,331	-0,01218434	0,1164979	NaN	0,2808376	NaN	0,2205776	NaN	-0,2353821	NaN	0,000721115	NaN	-0,06760617	5,41E-01	0,27	0,043351655	
Tubulin beta-3 chain	TUBB3	76636000	97121000	1,37	1,02	0,03	22,603	NaN	0,6167341	-0,08499168	NaN	0,2714257	0,1118326	-0,3102178	-0,4841665	NaN	NaN	-0,4843682	-0,5766047	4,59E-01	0,34	-0,117544563	
Leucine-rich repeat-containing protein 59	LRRC59	331310000	500390000	1,14	1,02	0,03	9,5922	-0,1538798	-0,1170831	-0,04595264	-0,07284687	-0,14264	-0,0987218	0,2058929	0,1554903	0,09234277	0,08379207	0,1062144	0,09153062	8,16E-01	0,09	0,008678225	
Golgi to ER traffic protein 4 homolog	GET4	19044000	31085000	1,42	1,02	0,03	20,006	0,1278974	0,000721115	NaN	0,09247811	0,2215678	-0,1059389	NaN	NaN	0,04935203	-0,06282134	-0,05160844	-0,02983845	4,66E-01	0,33	0,026867693	
Ubiquitin-conjugating enzyme E2 L3	UBE2L3	359190000	532690000	1,43	1,02	0,03	9,3635	-0,04452355	0,04488368	-0,05706139	-0,07043619	-0,1183197	-0,03563833	0,1206174	0,07956584	0,06267433	0,04376442	0,1298765	0,05144163	4,70E-01	0,33	0,017237051	
Tubulin--tyrosine ligase-like protein 12	TTLL12	283830000	356620000	1,37	1,02	0,03	16,289	0,3189242	0,2783417	0,0984196	0,1409095	0,005184378	0,2067682	-0,1002985	-0,0161618	-0,1279829	0,09342498	-0,134097	-0,001732303	1,78E-01	0,75	0,063474997	
Pre-mRNA-splicing factor ATP-dependent RNA helicase DHX15	DHX15	342060000	429130000	1,50	1,02	0,03	14,946	-0,1130831	-0,07978291	0,2471984	0,06681251	-0,2316505	-0,1525643	0,125651	-0,03166587	0,1025909	0,02941749	0,09747601	0,09761087	7,46E-01	0,13	0,013167547	
Calpain-1 catalytic subunit	CAPN1	410400000	536510000	1,25	1,02	0,03	11,22	-0,09260171	0,08420031	0,1244606	0,04012096	0,005759195	0,1356662	0,2002533	0,1582078	-0,245027	-0,07915797	-0,1549074	-0,2874306	8,47E-01	0,07	-0,009204696	
Proteasome subunit alpha type-2	PSMA2	369620000	494080000	1,32	1,02	0,03	10,313	0,02531228	0,01092296	-0,000634928	0,02630426	0,01849174	0,005902907	0,09261344	-0,02801326	0,0640551	0,08596853	0,08814155	0,07573799	7,08E-03	2,15	0,038733547	+
39S ribosomal protein L13, mitochondrial	MRPL13	27277000	35690000	1,25	1,02	0,03	19,337	-0,05102542	-0,05398785	1,187388	0,04907312	-0,2016923	0,05060612	NaN	NaN	0,1484786	0,165944	NaN	NaN	3,24E-01	0,49	0,161848008	
Spermidine synthase	SRM	122330000	157520000	1,21	1,02	0,02	11,664	0,03195977	0,02460336	0,08188502	-0,1768377	-0,07173962	-0,1357445	-0,0544223	0,1557492	-0,1246131	-0,09974179	-0,6255113	-0,2609987	9,87E-02	1,01	-0,104617638	
Cellular nucleic acid-binding protein	CNBP	61501000	72682000	1,54	1,02	0,02	16,574	-0,6305279	-0,6248437	-0,4007965	-0,3243286	-0,6466089	-0,5762604	0,08052122	0,04781753	0,0745054	0,04921259	0,1249898	0,06225996	3,45E-02	1,46	-0,230338306	
Isocitrate dehydrogenase [NAD] subunit alpha, mitochondrial	IDH3A	49888000	73727000	1,45	1,02	0,02	7,8303	0,312549	0,2631546	0,2263851	0,2368294	0,3530974	0,1244606	NaN	0,1008431	-0,05270043	0,0551957	NaN	NaN	3,45E-03	2,46	0,179979376	+
Heat shock protein beta-1	HSPB1	887500000	1177600000	1,44	1,02	0,02	10,908	0,003170453	-0,04529746	-0,01308669	-0,06346944	-0,0205892	-0,03603767	0,1971105	0,1683854	0,3507235	0,3305584	0,3076617	0,2041406	3,04E-02	1,52	0,115272511	
Proteasome subunit beta type-3	PSMB3	350800000	473940000	1,38	1,02	0,02	10,637	0,05450115	0,06598585	-0,07365134	-0,16882	0,03731201	-0,0581124	0,1390765	0,0724488	0,02147971	0,01421241	0,1025909	-0,01570959	5,28E-01	0,28	0,015942835	
Mitochondrial import inner membrane translocase subunit TIM50	TIMM50	96625000	123530000	1,21	1,02	0,02	12,028	-0,1756965	0,06129241	0,1186256	0,02970023	0,04040144	0,1055439	-0,1992222	-0,07217937	-0,158381	-0,04669748	0,07970238	NaN	5,88E-01	0,23	-0,019719155	
Host cell factor 1;HCF N-terminal chain 1;HCF N-terminal chain 2;HCF N-terminal chain 3;HCF N-terminal chain 4;HCF N-terminal chain 5;HCF N-terminal chain 6;HCF C-terminal chain 1;HCF C-terminal chain 2;HCF C-terminal chain 3;HCF C-terminal chain 4;HCF C-terminal chain 5;HCF C-terminal chain 6	HCFC1	91754000	103260000	1,20	1,01	0,02	22,394	0,2783417	0,2311866	-0,127778	0,0504668	-0,2140059	-0,2822632	NaN	0,3520805	NaN	NaN	0,559345	0,381837	2,00E-01	0,70	0,136578937	
Thioredoxin-like protein 1	TXNL1	161300000	200650000	1,22	1,01	0,02	9,6681	-0,01235901	0,02531228	0,05144163	0,003889986	-0,0612851	0,03646811	0,001009529	0,04432424	0,009347181	-0,07371206	0,02417773	0,1691554	3,27E-01	0,49	0,018147493	
Fructose-bisphosphate aldolase C	ALDOC	912540000	940720000	1,43	1,01	0,02	22,937	-0,5833764	-0,6968914	-0,3317175	-0,3584909	-0,5035684	-0,4436148	0,1844707	0,2135028	0,4261037	0,4603756	0,4086031	0,002162436	4,29E-01	0,37	-0,101870067	
Adenosine kinase	ADK	91818000	114870000	1,20	1,01	0,02	5,8108	0,2148708	0,05727703	0,2841587	0,04781753	-0,1076789	0,05088473	NaN	NaN	NaN	NaN	-0,0408382	0,02375215	1,85E-01	0,73	0,066280483	
Ribose-5-phosphate isomerase	RPIA	84973000	97731000	1,37	1,01	0,02	11,8	0,000721115	0,121148	-0,1039374	0,1538053	-0,02566156	0,1443075	-0,3138895	0,05172008	-0,1199023	-0,1490086	-0,127384	-0,482936	2,23E-01	0,65	-0,070918101	
T-complex protein 1 subunit zeta	CCT6A	2783900000	3688800000	1,29	1,01	0,02	5,2153	0,01106615	0,02800333	0,008630365	0,01020692	0,03224201	0,04893365	-0,08124738	-0,02099905	NaN	NaN	-0,08297317	-0,09488022	4,23E-01	0,37	-0,014101739	
Dolichol-phosphate mannosyltransferase subunit 1	DPM1	45285000	52295000	1,40	1,01	0,02	18,756	0,1393385	0,03083044	NaN	0,1870075	0,2496274	0,03703076	-0,1005923	NaN	0,4513299	-0,2765201	-0,9709218	-1,091172	4,28E-01	0,37	-0,134404136	
DnaJ homolog subfamily C member 7	DNAJC7	157310000	175400000	1,25	1,01	0,02	10,984	0,06681251	-0,08731949	-0,1075701	-0,1453021	-0,008653237	-0,02686637	0,3226204	-0,2513498	NaN	NaN	0,2228046	NaN	9,79E-01	0,01	-0,001647072	
Replication factor C subunit 5	RFC5	39057000	55438000	1,20	1,01	0,02	15,496	0,04432424	-0,09364811	0,2316782	0,2332746	-0,03880637	-0,001746681	NaN	NaN	0,001442041	-0,1300813	0,1086248	0,05880134	3,14E-01	0,50	0,041386281	
Protein ERGIC-53	LMAN1	106790000	147590000	1,28	1,01	0,02	9,5286	0,1200864	-0,0225369	0,02630426	0,2669964	0,02545406	0,06391706	NaN	NaN	NaN	NaN	-0,1892361	-0,1437393	7,29E-01	0,14	0,018405742	
Poly(U)-binding-splicing factor PUF60	PUF60	132780000	204680000	1,33	1,01	0,02	13,533	-0,04058593	0,1129003	0,000721115	-0,143102	-0,2199587	-0,09589748	0,03703076	0,05699965	0,1923205	0,1548424	0,1307991	0,1737671	4,51E-01	0,35	0,029986403	
Mitotic checkpoint protein BUB3	BUB3	302020000	334300000	1,18	1,01	0,02	11,097	-0,3128676	-0,04256084	-0,07911219	-0,2789337	-0,4347733	-0,171011	0,03857661	0,01991543	0,0610158	0,07956584	NaN	NaN	7,92E-02	1,10	-0,112018499	
Up-regulated during skeletal muscle growth protein 5	USMG5	59970000	79314000	1,39	1,01	0,02	9,3025	0,08976937	0,08065767	-0,08002695	0,001442041	0,05755419	0,1830736	0,05949366	0,07038926	-0,08972783	-0,1194947	-0,1179439	0,07601179	5,45E-01	0,26	0,017599853	
O-acetyl-ADP-ribose deacetylase 1	OARD1	25663000	35528000	1,21	1,01	0,01	12,421	-0,2137549	-0,006492313	-0,04957635	-0,09703889	-0,2013936	-0,1312026	0,3054453	0,4088205	0,3253864	0,4927248	0,01321208	0,3394795	2,09E-01	0,68	0,098800839	
CDGSH iron-sulfur domain-containing protein 2	CISD2	68476000	88748000	1,30	1,01	0,01	22,131	-0,05685135	0,07272322	-0,0169061	0,1232689	0,06942715	0,08311115	0,1672295	-0,1882823	NaN	-0,03393872	0,06073913	NaN	4,13E-01	0,38	0,028052052	
Rho-related GTP-binding protein RhoG	RHOG	104240000	130760000	1,31	1,01	0,01	16,392	0,3572705	-0,07149699	-0,08955901	-0,09722405	-0,04778576	-0,07072407	0,0500489	0,1000357	0,03153631	-0,06678995	0,05644473	-0,0995563	9,10E-01	0,04	0,004350003	
Ubiquitin carboxyl-terminal hydrolase 15	USP15	140070000	158000000	1,15	1,01	0,01	14,601	0,04068203	0,05158086	0,001153714	-0,02591123	0,06018582	0,0207689	-0,3304652	-0,3450512	-0,04128348	-0,3438241	-0,4276536	-0,3318264	3,07E-02	1,51	-0,139303659	
Proteasome subunit alpha type-4	PSMA4	449850000	635120000	1,42	1,01	0,01	5,5157	0,1186256	0,0911243	0,04879416	0,03026536	0,0618453	0,1210154	-0,01543256	0,001586012	-0,09924714	-0,08807073	0,02970023	-0,02061851	2,85E-01	0,54	0,023298947	
Translation initiation factor eIF-2B subunit alpha	EIF2B1	77141000	103620000	1,33	1,01	0,01	9,0355	0,02885203	0,1641426	0,1184926	-0,08454792	0,2221863	0,06598585	0,248292	0,0650208	NaN	NaN	-0,004363514	NaN	3,44E-02	1,46	0,091562307	
Cyclin-dependent kinase 2	CDK2	16850000	24456000	1,48	1,01	0,01	25,233	0,04795711	-0,008522615	-0,02450185	-0,07154253	-0,006231471	0,06018582	NaN	0,3192711	0,2940766	0,1979911	NaN	0,2446436	4,68E-02	1,33	0,105332686	
Ferritin light chain	FTL	100870000	133810000	1,40	1,01	0,01	12,175	0,1479579	0,1819295	0,6618408	-0,06691082	0,07942928	0,3068455	-0,9837033	-0,212718	-0,06205306	0,000144286	-1,064133	-0,4084354	4,29E-01	0,37	-0,118317196	
Basigin	BSG	303100000	397590000	1,22	1,01	0,01	11,145	-0,0752616	-0,08075912	0,05977056	-0,06757589	0,1350094	-0,02239035	NaN	NaN	NaN	NaN	0,1023222	0,2953704	3,84E-01	0,42	0,043310706	
Prefoldin subunit 4	PFDN4	31656000	36333000	1,36	1,01	0,01	14,352	-0,09525	-0,171336	0,1971105	-0,115488	0,02133754	-0,01903878	NaN	-0,1857695	NaN	0,1809117	NaN	NaN	6,69E-01	0,17	-0,023440321	
Chloride intracellular channel protein 2	CLIC2	27146000	34223000	1,37	1,01	0,01	19,689	-0,06606483	-0,0628967	0,01863419	-0,1016136	0,02559582	-0,2120995	NaN	NaN	0,1589833	0,08949816	-0,08877627	0,2504765	9,79E-01	0,01	0,001173708	
Nucleoporin Nup43	NUP43	108610000	137270000	1,30	1,01	0,01	12,385	0,2263851	0,02091112	-0,06457045	0,1945914	-0,203369	-0,009640465	0,006621249	NaN	NaN	NaN	-0,4094316	-0,1372987	5,40E-01	0,27	-0,041755696	
V-type proton ATPase catalytic subunit A	ATP6V1A	169280000	221650000	1,29	1,01	0,01	13,475	0,002594432	0,1602748	0,1830736	0,1066163	-0,09280171	0,004752986	-0,005304398	-0,03000043	0,2188431	0,2386647	0,1087586	0,01763692	3,24E-02	1,49	0,076092408	
NADH dehydrogenase [ubiquinone] 1 beta subcomplex subunit 10	NDUFB10	80266000	115090000	1,47	1,01	0,01	14,702	0,09207205	0,07121356	0,05713834	0,2498701	-0,2092279	0,1087586	-0,1254627	-0,08578761	0,1066163	0,01578305	0,08229379	-0,000692654	4,12E-01	0,39	0,030214576	
Proteasome subunit alpha type-6	PSMA6	779250000	1059700000	1,35	1,01	0,01	4,7468	0,003602319	0,08678382	0,05102402	0,03040665	0,1072862	0,02871058	0,005328146	-0,03929557	-0,06283643	-0,07880746	0,02814484	-0,01941891	4,84E-01	0,32	0,011744019	
ADP-ribosylation factor-like protein 2	ARL2	28674000	42407000	1,24	1,00	0,01	12,222	0,0880058	0,03266509	0,002450562	0,05436236	0,05074544	-0,02535321	NaN	NaN	1,140517	NaN	0,000576887	NaN	2,68E-01	0,57	0,16799624	
Inosine triphosphate pyrophosphatase	ITPA	26407000	33285000	1,37	1,00	0,01	9,3744	0,1243282	0,005902907	NaN	0,008917032	0,05797008	NaN	-0,3392721	-0,2239129	-0,1330367	0,1745343	0,02133754	-0,1174117	4,26E-01	0,37	-0,042064338	
Protein-glutamine gamma-glutamyltransferase 2	TGM2	28985000	39361000	1,37	1,00	0,01	24,651	0,2346239	0,03787417	0,4957975	0,2251515	0,2629142	0,4213715	-0,2618116	-0,3466281	NaN	NaN	NaN	NaN	2,53E-01	0,60	0,133661634	
DnaJ homolog subfamily B member 1	DNAJB1	46050000	69775000	1,48	1,00	0,01	10,215	0,1210154	0,2947824	0,2566484	0,3089433	0,1331689	0,1977397	-0,1129427	0,2151194	0,0766959	0,01663897	NaN	0,3062623	2,21E-03	2,66	0,164915628	+
Protein CutA	CUTA	94513000	135890000	1,33	1,00	0,00	7,2012	0,08133958	0,03012422	NaN	0,01049345	0,02247432	0,04656085	-0,01320315	0,08420031	-0,1647712	NaN	0,06364096	-0,01492228	5,38E-01	0,27	0,014593709	
T-complex protein 1 subunit eta	CCT7	2903300000	3855800000	1,30	1,00	0,00	6,829	0,01378388	0,01292609	-0,04626543	0,005759195	0,05325044	0,01592577	0,03941911	-0,01511179	NaN	NaN	-0,03713267	-0,02344575	8,55E-01	0,07	0,001910884	
BolA-like protein 2	BOLA2	35634000	48175000	1,39	1,00	0,00	6,0441	0,100574	0,07093885	0,008200003	-0,07243722	0,0408223	0,09653179	-0,05514159	NaN	-0,06529482	0,04558271	0,01749438	-0,08130842	6,50E-01	0,19	0,009632903	
Enoyl-CoA delta isomerase 2, mitochondrial	ECI2	38619000	43002000	1,52	1,00	0,00	28,028	0,2005043	0,5210507	NaN	NaN	0,4635179	0,4386122	-0,5349345	-0,5884436	-0,3225413	-0,4758761	NaN	NaN	8,35E-01	0,08	-0,037263809	
ATP synthase subunit gamma, mitochondrial	ATP5C1	105630000	137840000	1,34	1,00	0,00	11,662	0,1281614	0,01249718	0,125651	0,2016338	0,06377902	0,09450638	NaN	-0,03851003	-0,08975858	-0,2215391	-0,1341604	-0,2194211	8,76E-01	0,06	-0,007014572	
Ubiquitin-like modifier-activating enzyme 6	UBA6	173600000	190310000	1,13	1,00	0,00	14,099	0,000432815	0,007482617	0,07833651	-0,01214072	0,1188912	0,06018582	-0,2309223	-0,1614112	0,06281248	0,09868897	-0,1989904	-0,1398079	4,75E-01	0,32	-0,026370179	
Cytochrome b5 type B	CYB5B	32447000	44705000	1,39	1,00	0,00	5,6059	-0,1403484	0,0413831	NaN	NaN	-0,07646286	-0,05653617	-0,0229619	-0,04648894	-0,05659618	0,009490535	0,06073913	-0,04708496	1,05E-01	0,98	-0,033486671	
T-complex protein 1 subunit epsilon	CCT5	2408700000	3129400000	1,25	1,00	0,00	9,649	-0,01394593	-0,04854648	-0,03587492	-0,04948682	-0,054677	-0,003943961	0,05394543	0,01292609	0,2281107	NaN	0,008630365	0,05699965	5,79E-01	0,24	0,014012466	
BH3-interacting domain death agonist;BH3-interacting domain death agonist p15;BH3-interacting domain death agonist p13;BH3-interacting domain death agonist p11	BID	46983000	63218000	1,33	1,00	0,00	14,889	0,03660879	0,2270017	-0,006579213	0,02275842	-0,02914632	0,02970023	-0,1035809	-0,2431475	0,05213766	-0,3756464	0,4096896	-0,0223024	9,97E-01	0,00	-0,000208857	
Polymerase delta-interacting protein 2	POLDIP2	27794000	28719000	1,15	1,00	0,00	8,3368	0,2022611	-0,000880304	0,08732703	0,0633648	-0,1384099	-0,01715427	NaN	NaN	-1,224284	-1,212501	NaN	NaN	2,19E-01	0,66	-0,280034476	
Ras-related protein Rab-6A	RAB6A	220130000	307620000	1,31	1,00	0,00	9,2209	-0,0128538	-0,02171653	-0,1071505	-0,01842497	-0,03797669	-0,05689634	0,09707135	0,08556063	0,1194226	0,2025117	0,1055439	-0,02927882	3,27E-01	0,49	0,027151051	
Proteasome subunit beta type-7	PSMB7	110690000	113210000	1,43	1,00	0,00	12,798	-0,07609778	-0,1770497	0,04963072	-0,1183824	NaN	-0,6223543	-0,04550591	0,05436236	-0,2295346	-0,03379109	0,1292171	-0,01027965	1,39E-01	0,86	-0,098162283	
Importin subunit alpha-3	KPNA4	89967000	129100000	1,40	1,00	0,00	3,9748	0,4153261	0,03252402	0,05144163	0,01049345	-0,01182068	-0,2239297	NaN	NaN	NaN	NaN	-0,01131167	0,02559582	5,81E-01	0,24	0,03603987	
Nucleoside diphosphate kinase A	NME1	381480000	535120000	1,42	1,00	0,00	2,9888	-0,003770397	0,007051912	-0,01455784	-0,04666772	0,02204824	-0,008406535	0,01706665	-0,02621979	-0,006390821	0,008200003	-0,01215529	-0,04311067	1,82E-01	0,74	-0,008909355	

T-complex protein 1 subunit beta	CCT2	3680200000	4815600000	1,27	1,00	0,00	6,8598	0,02772026	-0,0151701	-0,000808115	0,01564032	0,0413831	-0,02456054	0,003458378	-0,06435923	0,6610199	0,2380531	-0,05917922	-0,02504493	2,80E-01	0,55	0,066512737	
Eukaryotic translation initiation factor 6	EIF6	322710000	470380000	1,54	1,00	0,00	7,3302	-0,02617566	-0,05419756	0,2447654	0,1457427	-0,04960625	-0,02310851	0,08188502	0,06598585	0,1245928	0,07942928	0,1852322	0,1008431	2,33E-02	1,63	0,07294902	+
UTP--glucose-1-phosphate uridylyltransferase	UGP2	79472000	114600000	1,22	1,00	0,00	8,1345	0,08488071	0,04628156	-0,03144473	NaN	-0,0493076	-0,05949498	NaN	NaN	NaN	NaN	-0,1963762	0,06529664	6,04E-01	0,22	-0,020023516	
60 kDa heat shock protein, mitochondrial	HSPD1	7517100000	9915200000	1,28	1,00	0,00	5,4325	0,05060612	0,02304246	0,01720924	0,03421579	0,04278438	0,01535482	-0,06880108	-0,084808	-0,04651869	-0,04456816	-0,0522516	-0,03135623	3,95E-01	0,40	-0,012090913	
Adenylosuccinate lyase	ADSL	81951000	108210000	1,30	1,00	0,00	10,387	0,2126316	-0,01941891	0,6201171	0,06653696	0,4269625	-0,001544543	NaN	NaN	NaN	NaN	0,07915612	-0,1513139	1,31E-01	0,88	0,154140865	
Nuclear mitotic apparatus protein 1	NUMA1	32648000	34938000	1,16	1,00	-0,01	24,014	NaN	-0,009989116	0,06846439	0,2499914	-0,1473459	-0,3731777	-0,08231617	0,09707135	0,1191569	0,1294808	NaN	NaN	9,29E-01	0,03	0,005703994	
SUMO-activating enzyme subunit 1;SUMO-activating enzyme subunit 1, N-terminally processed	SAE1	191080000	259230000	1,35	1,00	-0,01	11,44	0,1013812	0,05018827	0,07874646	-0,03799152	0,02417773	0,01464098	-0,1117419	-0,176267	-0,1609434	-0,1904045	NaN	0,2156165	6,66E-01	0,18	-0,017508823	
Adenylate kinase 2, mitochondrial;Adenylate kinase 2, mitochondrial, N-terminally processed	AK2	463850000	530930000	1,34	1,00	-0,01	10,744	-0,1979971	-0,2143741	-0,11868	-0,1587997	-0,1492966	-0,1642699	0,1401242	0,1681285	0,003746074	0,06970215	0,1269729	0,08351974	4,34E-01	0,36	-0,034268648	
Eukaryotic translation initiation factor 2 subunit 1	EIF2S1	159990000	229670000	1,23	1,00	-0,01	9,6961	-0,04753228	0,01877663	-0,1224285	-0,06330366	-0,09180192	-0,07892934	-0,07308965	0,06832678	-0,03876194	-0,03800636	0,1983684	0,1954736	8,48E-01	0,07	-0,00607568	
ATP-citrate synthase	ACLY	1828500000	2115000000	1,16	1,00	-0,01	12,984	-0,03308228	-0,04324446	0,03562387	0,05713834	0,06736329	0,05283319	-0,197418	-0,1766746	-0,131171	-0,1267852	0,02587931	-0,009669553	1,75E-01	0,76	-0,039933925	
Hippocalcin-like protein 1;Neuron-specific calcium-binding protein hippocalcin	HPCAL1;HPCA	15334000	20384000	1,39	1,00	-0,01	3,3118	-0,2348557	-0,3081264	NaN	NaN	NaN	NaN	0,1037997	NaN	0,1135672	0,04474379	NaN	NaN	5,64E-01	0,25	-0,056174272	
Protein SEC13 homolog	SEC13	85510000	121500000	1,35	1,00	-0,01	7,649	0,09410089	0,1368475	-0,02132113	0,2576141	-0,08326348	-0,01388767	-0,06830177	-0,0665331	-0,1232925	0,02616273	NaN	NaN	7,19E-01	0,14	0,013812559	
Serpin B6	SERPINB6	503240000	632300000	1,30	1,00	-0,01	6,9805	0,04963072	0,05921687	0,0415233	0,05255495	-0,03129726	0,05436236	-0,01339243	-0,08925197	-0,2567005	-0,211615	0,2208253	0,2699907	7,86E-01	0,10	0,01215392	
Ras-related protein Rap-1b;Ras-related protein Rap-1b-like protein	RAP1B	352270000	470480000	1,38	0,99	-0,01	6,3775	-0,09446426	-0,04739808	-0,02395906	-0,02139436	-0,05395794	-0,005753356	0,1793837	0,1364539	0,1321162	0,1989969	0,158854	0,07587489	1,08E-01	0,97	0,052896058	
Hsp90 co-chaperone Cdc37;Hsp90 co-chaperone Cdc37, N-terminally processed	CDC37	221310000	292970000	1,38	0,99	-0,01	11,565	-0,2493763	-0,07929515	-0,2466351	-0,1528048	-0,165483	-0,03042763	0,1825652	0,2200823	NaN	NaN	0,1672295	0,02048441	5,64E-01	0,25	-0,033366062	
Macrophage migration inhibitory factor	MIF	395540000	513850000	1,47	0,99	-0,01	21,409	-0,2088611	-0,2125676	-0,1622828	-0,1641082	-0,2034354	-0,2197739	0,08011194	0,2067682	0,09301919	0,0724488	0,07710631	0,08868441	3,41E-01	0,47	-0,046074185	
NAD(P) transhydrogenase, mitochondrial	NNT	76602000	87999000	1,22	0,99	-0,01	14,521	0,2526579	NaN	-0,02089653	-0,04273913	0,1491292	0,095722	NaN	-0,1154412	-0,05249094	NaN	NaN	NaN	4,73E-01	0,33	0,037991625	
T-complex protein 1 subunit gamma	CCT3	3553300000	4631900000	1,28	0,99	-0,01	5,8523	0,02147971	0,02842765	0	-0,03157745	0,01120934	0,003889986	-0,01642444	-0,05126464	-0,0946029	-0,06638201	-0,0169061	0,01549758	1,64E-01	0,78	-0,016387773	
Isochorismatase domain-containing protein 2, mitochondrial	ISOC2	25750000	35910000	1,35	0,99	-0,01	5,5565	-0,04550591	0,0311129	NaN	NaN	-0,006782144	0,08854871	NaN	NaN	0,10742	-0,06389166	NaN	NaN	5,46E-01	0,26	0,018483649	
Far upstream element-binding protein 1	FUBP1	253590000	339910000	1,36	0,99	-0,01	14,171	0,07162537	0,1023222	-0,1859336	-0,08071329	-0,1940473	-0,08256063	-0,05794723	0,1048734	0,03604614	0,1521185	0,2332746	0,3219281	4,65E-01	0,33	0,035082183	
Cell division control protein 42 homolog	CDC42	86996000	138640000	1,33	0,99	-0,01	7,6652	-0,03438189	-0,005666505	-0,1084098	-0,0292052	-0,1757942	-0,03760647	0,06529664	0,1019189	0,2836848	0,07860982	0,044464	-0,0507116	7,50E-01	0,12	0,011016547	
Radixin	RDX	116330000	141730000	1,11	0,99	-0,01	8,8847	-0,1392358	-0,03670348	-0,1463396	0,02048441	0,003314423	-0,02995627	NaN	NaN	NaN	NaN	-0,2533261	-0,02799853	5,57E-02	1,25	-0,076220117	
Ras-related protein Rab-11B	RAB11B	271370000	363890000	1,39	0,99	-0,01	10,908	-0,002671471	0,01306909	-0,01080285	-0,030914	0,02984157	0,004609331	-0,06550618	-0,1416529	-0,1526444	-0,0122571	-0,02416445	-0,07308965	4,23E-02	1,37	-0,03884858	
Copine-3	CPNE3	180210000	247000000	1,34	0,99	-0,01	16,05	-0,1208118	0,07560106	0,1004395	0,1216786	0,1285573	0,143524	NaN	-0,2398557	NaN	NaN	0,009060428	-0,2305837	9,79E-01	0,01	-0,001376698	
Protein disulfide-isomerase A3	PDIA3	1152300000	1488500000	1,23	0,99	-0,01	18,957	-0,1423215	-0,003640216	-0,01827883	-0,003061786	-0,05784214	-0,1567558	0,1052758	0,144177	NaN	NaN	0,01891905	-0,01302846	6,80E-01	0,17	-0,012655688	
Prothymosin alpha;Prothymosin alpha, N-terminally processed;Thymosin alpha-1	PTMA	189820000	257870000	1,33	0,99	-0,01	4,365	-0,005362311	-0,1475057	-0,1946911	-0,05452714	-0,1128803	0,03843611	-0,02304988	0,03759303	-0,000837024	-0,02161396	0,06598585	-0,02357773	1,33E-01	0,88	-0,036835844	
Citrate synthase, mitochondrial	CS	1097200000	1360800000	1,31	0,99	-0,01	7,4818	-0,01482019	0,1009776	0,005902907	-0,0204868	-0,04218935	0,01521204	-0,08499168	-0,04055621	-0,05214684	-0,04235281	NaN	-0,3681041	1,89E-01	0,72	-0,049414129	
Hsc70-interacting protein;Putative protein FAM10A4;Putative protein FAM10A5	ST13;ST13P4;ST13P5	515840000	689350000	1,28	0,99	-0,01	10,555	0,02233225	0,114367	-0,01479109	-0,01356724	-0,04619094	-0,04629518	-0,01362549	0,04474379	0,3827226	0,2487777	-0,06984551	-0,05862323	2,77E-01	0,56	0,045833718	
Dynein assembly factor 5, axonemal	DNAAF5	54852000	65987000	1,22	0,99	-0,01	9,6577	-0,1750937	-0,02497152	0,08229379	-0,1772454	-0,2020739	0,1241958	NaN	0,2603871	0,07560106	0,03703076	0,2973675	-0,05580126	6,76E-01	0,17	0,021971836	
Transgelin-2	TAGLN2	915410000	1296000000	1,36	0,99	-0,01	9,342	-0,1305708	-0,1857695	-0,07263444	-0,05168321	-0,1317874	-0,09155598	0,01464098	0,06818932	0,05394543	0,02275842	0,03195977	-0,008392004	1,22E-01	0,91	-0,040074948	
Ubiquitin-conjugating enzyme E2 D3;Ubiquitin-conjugating enzyme E2 D2	UBE2D3;UBE2D2	24650000	29634000	1,12	0,99	-0,02	15,304	0,02460336	0,6072001	0,2266318	0,03857661	-0,2249578	NaN	NaN	NaN	NaN	NaN	0,1206174	0,1887806	1,94E-01	0,71	0,140207447	
Proteasome subunit beta type-2	PSMB2	152850000	196810000	1,33	0,99	-0,02	13,744	-0,03925103	-0,04997956	-0,04400288	-0,06763636	-0,001472321	-0,02820449	-0,01911189	-0,09592827	-0,0843797	-0,01412072	NaN	0,1483484	2,00E-01	0,70	-0,026885347	
Complement component 1 Q subcomponent-binding protein, mitochondrial	C1QBP	183960000	242490000	1,17	0,99	-0,02	8,7518	-0,123654	-0,02610219	0,0160683	-0,02730736	-0,04701051	-0,08692104	0,06515872	0,1286893	-0,0282486	-0,01276644	-0,04681677	-0,104046	2,54E-01	0,59	-0,024413053	
Voltage-dependent anion-selective channel protein 2	VDAC2	1154100000	1548800000	1,31	0,99	-0,02	15,607	0,07313477	0,03745244	0,1486088	0,09220742	0,1004395	0,1300083	-0,254909	-0,196558	-0,2761357	-0,2774641	-0,2481935	-0,3513505	1,59E-01	0,80	-0,085229957	
Arginine--tRNA ligase, cytoplasmic	RARS	469370000	583910000	1,16	0,99	-0,02	13,972	-0,1322616	-0,07652371	-0,07999641	-0,06199282	-0,09463365	-0,01183524	-0,07771078	-0,007231562	NaN	NaN	0,03913844	-0,00510176	1,32E-02	1,88	-0,050814913	+
Splicing factor 3B subunit 3	SF3B3	633870000	724930000	1,16	0,99	-0,02	18,329	-0,1735796	-0,05142904	-0,0785027	0,09058251	-0,1368386	-0,1584293	0,0451634	0,1106305	0,06129241	0,01991543	0,01720924	0,1971105	8,90E-01	0,05	-0,004739608	
T-complex protein 1 subunit alpha	TCP1	3410800000	4425700000	1,29	0,99	-0,02	7,1833	0,008343471	-0,02717504	-0,03197566	-0,02582316	0,01834928	-0,01458703	-0,03451487	0,03618686	0,0292761	0,02644594	-0,009393595	-4,33E-05	7,83E-01	0,11	-0,002075913	
Glycylpeptide N-tetradecanoyltransferase 1	NMT1	154680000	202350000	1,13	0,99	-0,02	13,714	0,006477609	-0,2556494	0,04502354	-0,1475856	-0,06907348	-0,07748247	0,2811937	0,5933059	NaN	NaN	0,2075181	0,2894804	3,06E-01	0,51	0,087320842	
Replication factor C subunit 3	RFC3	34710000	44758000	1,23	0,99	-0,02	16,791	NaN	0,02531228	NaN	0,1885276	NaN	-0,04741292	-0,2787411	-0,1350631	-0,221741	-0,2131025	NaN	NaN	1,68E-01	0,77	-0,097460115	
Biliverdin reductase A	BLVRA	248830000	299760000	1,18	0,99	-0,02	9,4792	-0,05208702	-0,1447119	-0,1456213	-0,1157069	-0,005347897	-0,04052658	-0,2755767	-0,2523977	-0,2089278	-0,1589608	NaN	NaN	8,12E-04	3,09	-0,139986457	+
Elongation factor Ts, mitochondrial	TSFM	31314000	39631000	1,21	0,99	-0,02	8,3482	-0,172246	-0,1303812	0,03407487	-0,004392495	-0,0920326	0,06722574	NaN	NaN	0,1180938	0,1162316	NaN	NaN	8,47E-01	0,07	-0,007928279	
T-complex protein 1 subunit delta	CCT4	2986700000	3912600000	1,35	0,99	-0,02	6,2437	-0,00513067	-0,01601588	-0,0752616	-0,07734546	-0,02272741	-0,03701426	-0,05644621	-0,04138746	0,4731115	0,009060428	-0,02849872	-0,009001562	8,30E-01	0,08	0,009445227	
COP9 signalosome complex subunit 3	COPS3	71136000	100590000	1,35	0,99	-0,02	5,6773	NaN	0,3329657	0,4322112	0,1293489	-0,1035964	0,03195977	NaN	-0,0929555	-0,08208712	-0,1652402	NaN	NaN	4,64E-01	0,33	0,060325797	
Endoplasmin	HSP90B1	1879100000	2289200000	1,25	0,99	-0,02	16,197	0,002162436	0,002162436	-0,04698066	0,01049345	-0,06390676	0,005184378	-0,00105352	-0,07781741	-0,003625733	-0,0226688	-0,02799853	0,001153714	5,27E-02	1,28	-0,018574583	
UMP-CMP kinase	CMPK1	179150000	253740000	1,43	0,99	-0,02	11,378	0,03463829	0,07286042	0,006046604	0,01592577	0,1376347	0,1106305	-0,2021902	-0,1316451	-0,09194031	-0,07034535	-0,1581074	-0,1750937	2,85E-01	0,54	-0,037632141	
Ankyrin-1	ANK1	381030000	404120000	1,08	0,99	-0,02	12,996	-0,05742169	-0,003249744	-0,2369454	-0,09467994	-0,05434738	0,01906128	-0,233142	-0,2351953	-0,06799932	NaN	-0,2758562	-0,05283511	4,35E-03	2,36	-0,117510073	+
Far upstream element-binding protein 3	FUBP3	30845000	61727000	1,15	0,99	-0,02	29,675	0,3141744	NaN	0,08732703	0,2559237	0,01991543	0,1075539	NaN	NaN	NaN	NaN	-0,5155835	0,2267551	5,26E-01	0,28	0,070866591	
Eukaryotic peptide chain release factor GTP-binding subunit ERF3A	GSPT1	158490000	179720000	1,12	0,99	-0,02	23,714	0,2349918	-0,2373705	NaN	0,0745054	-0,2477995	0,1286893	-0,1369338	-0,198742	NaN	0,06956466	NaN	NaN	5,73E-01	0,24	-0,039136824	
ATP-dependent 6-phosphofructokinase, liver type	PFKL	113980000	142960000	1,22	0,98	-0,02	14,08	0,07052668	-0,002613665	-0,1282352	0,1645289	0,004752986	0,1007085	-0,4296342	NaN	-0,1560805	NaN	NaN	NaN	5,04E-01	0,30	-0,047005804	
GTPase NRas	NRAS	23955000	30788000	1,38	0,98	-0,02	5,6378	0,005328146	-0,07129993	-0,00991647	0,06308875	-0,06958812	0,01863419	-0,1329417	-0,2619672	NaN	-0,04372028	NaN	NaN	1,21E-01	0,92	-0,055820293	
Hypoxanthine-guanine phosphoribosyltransferase	HPRT1	131910000	172400000	1,43	0,98	-0,02	12,384	-0,02346035	0,05018827	0,02431962	0,07710631	0,1420866	0,1194226	-0,2691297	-0,3237326	-0,2669234	-0,136236	-0,2715831	NaN	1,67E-01	0,78	-0,079812874	
Mitogen-activated protein kinase 1	MAPK1	765000000	915560000	1,23	0,98	-0,02	8,4455	0,1172961	0,02133754	-0,03203465	-0,06248986	-0,1226013	-0,006361886	-0,1357128	-0,1060321	-0,07249789	-0,1264703	NaN	NaN	6,74E-02	1,17	-0,052556715	
Suppressor of G2 allele of SKP1 homolog	SUGT1	36496000	49231000	1,22	0,98	-0,02	10,909	-0,000533933	NaN	-0,04950168	0,3580586	0,09207205	0,03689012	-0,1694202	0,02616273	-0,1071505	-0,03754725	NaN	NaN	7,49E-01	0,13	0,016558889	
Spliceosome RNA helicase DDX39B	DDX39B	713510000	872510000	1,16	0,98	-0,02	11,345	-0,06798417	-0,05731664	0,1050075	0,03632757	-0,1851953	-0,166616	0,06087747	-0,07255857	0,07025199	0,0494913	0,03913844	-0,01361091	5,47E-01	0,26	-0,016848939	
Activated RNA polymerase II transcriptional coactivator p15	SUB1	124130000	175240000	1,53	0,98	-0,02	5,7423	-0,2196227	-0,1221144	-0,05866828	-0,05949498	-0,289792	-0,1904374	-0,01025064	0,2481705	0,09058251	0,01920368	0,1352722	0,231801	7,14E-01	0,15	-0,018779223	
39S ribosomal protein L12, mitochondrial	MRPL12	48297000	70034000	1,21	0,98	-0,03	16,304	-0,625222	-0,0998191	-0,1322775	-0,0713909	-0,04236769	-0,09183271	0,2033891	0,1243282	0,03125411	0,1116991	-0,1741655	NaN	3,17E-01	0,50	-0,069673176	
Prefoldin subunit 6	PFDN6	32062000	40021000	1,14	0,98	-0,03	26,917	0	-0,1105109	-0,2307022	NaN	-0,215094	-0,02034045	NaN	0,258338	-0,5962306	0,206018	NaN	-0,02661653	3,64E-01	0,44	-0,081682078	
NADH dehydrogenase [ubiquinone] 1 alpha subcomplex subunit 5	NDUFA5	18085000	23188000	1,28	0,98	-0,03	12,965	-0,1759246	-0,1284875	-0,03193135	NaN	-0,1151286	NaN	0,2023863	NaN	-0,0394883	NaN	0,2010065	0,1658154	8,65E-01	0,06	0,009780983	
Nuclear pore glycoprotein p62	NUP62	54644000	61712000	1,06	0,98	-0,03	8,7214	-0,2292471	-0,2480736	-0,1315345	0,009633875	-0,5404424	-0,4174055	-0,1922	0,003314423	0,001730139	0,007339063	0,2855797	0,2487777	1,83E-01	0,74	-0,100210678	
Serpin B9	SERPINB9	363980000	409270000	1,26	0,98	-0,03	10,815	-0,3167617	-0,2851435	0,09139519	0,1031283	-0,00459512	0,07519039	0,2960756	-0,1084098	0,1151664	0,1943392	NaN	NaN	8,04E-01	0,09	0,016038504	
ADP-ribosylation factor 1;ADP-ribosylation factor 3	ARF1;ARF3	582780000	765340000	1,27	0,98	-0,03	8,1245	0,01891905	0,03815525	0,03520166	0,05255495	0,06364096	0,03689012	-0,1482889	-0,101722	-0,1281563	-0,1111028	-0,06629143	-0,0661102	1,98E-01	0,70	-0,031359141	
Eukaryotic translation initiation factor 5A-1;Eukaryotic translation initiation factor 5A-1-like;Eukaryotic translation initiation factor 5A-2	EIF5A;EIF5AL1;EIF5A2	821280000	1012800000	1,28	0,98	-0,03	6,548	0,004752986	-0,0661102	0	-0,04094214	-0,1734007	-0,1518107	-0,05026339	0,0618453	-0,0163953	0,04026129	0,04432424	0,03393393	2,54E-01	0,60	-0,026150392	
Multifunctional protein ADE2;Phosphoribosylaminoimidazole-succinocarboxamide synthase;Phosphoribosylaminoimidazole carboxylase	PAICS	2658500000	3422700000	1,30	0,98	-0,03	9,5257	0,02729555	0,07066408	0,02998291	-0,06659353	0,02034214	0,000432815	0,06956466	0,006477609	-0,03603767	-0,04418135	-0,01864412	0,03181863	5,53E-01	0,26	0,007593477	
Protein disulfide-isomerase A6	PDIA6	1196000000	1514200000	1,25	0,98	-0,03	11,238	-0,1451745	-0,02849872	0,0353423	0,04656085	-0,01157343	0,004321636	-0,04453836	0,03308822	-0,1765441	-0,09609793	0,02899346	0,01278325	2,10E-01	0,68	-0,028444781	
UV excision repair protein RAD23 homolog B	RAD23B	170700000	210470000	1,23	0,98	-0,03	12,972	0,1881477	0,02147971	0,1393385	0,01249718	0,2653169	-0,04516348	NaN	0,5232608	0,779722	NaN	0,3677065	NaN	2,37E-02	1,62	0,250256198	+
Tubulin alpha-1C chain	TUBA1C	383850000	487750000	1,29	0,98	-0,03	4,5253	0,007195495	-0,06777245	0,04222413	0,05921687	0,02318429	-0,1297024	-0,1543453	-0,2405544	-0,1019078	-0,2284863	-0,3291415	-0,1110561	1,46E-02	1,84	-0,102595448	+
X-ray repair cross-complementing protein 6	XRCC6	949300000	1115300000	1,16	0,98	-0,03	12,241	-0,1515543	-0,1813948	-0,1310604	-0,1826223	-0,2066617	-0,151378	0,1969846	0,1830736	0,1501697	0,1467856	0,0615688	0,100574	7,78E-01	0,11	-0,013792952	
Nodal modulator 1;Nodal modulator 3;Nodal modulator 2	NOMO1;NOMO3;NOMO2	65123000	69804000	1,10	0,98	-0,03	17,989	-0,06294189	-0,1455734	-0,1527567	-0,1360299	-0,02942608	0,01564032	0,1120997	-0,4404788	NaN	NaN	0,1062144	-0,3394729	8,95E-02	1,05	-0,107272531	
Vacuolar protein sorting-associated protein 28 homolog	VPS28	20513000	26190000	1,36	0,98	-0,03	7,8699	0,2849878	-0,1313923	NaN	-0,03424891	-0,1976662	-0,02019411	0,2216915	-0,1281721	NaN	-0,1328468	0,0729976	-0,1970045	6,38E-01	0,20	-0,026184792	
Nuclear pore complex protein Nup205	NUP205	172200000	195060000	1,11	0,98	-0,04	10,482	-0,07561126	-0,06970923	0,2263851	0,2613503	-0,2791962	-0,1936677	-0,2329215	-0,04427055	-0,2202949	-0,1365848	-0,1918539	-0,1105732	9,98E-02	1,00	-0,088912315	
Midasin	MDN1	14979000	14662000	0,94	0,98	-0,04	11,82	NaN	NaN	0,1115655	0,01092296	-0,6574225	-0,1275415	-0,7188775	NaN	-0,665775	NaN	NaN	NaN	7,93E-02	1,10	-0,341188006	
Regulator of chromosome condensation	RCC1	120500000	177130000	1,49	0,97	-0,04	19,353	0,6371929	NaN	0,444773	0,4962067	0,07464243	0,044464	-0,2372515	-0,2451638	-0,1642375	NaN	-0,02302053	-0,1879044	4,39E-01	0,36	0,083970136	
Ras suppressor protein 1	RSU1	26184000	33697000	1,23	0,97	-0,04	9,6351	-0,03956246	-0,05172813	-0,05292485	-0,1070417	-0,2173063	-0,1875429	NaN	NaN	0,02729555	NaN	NaN	0,1340894	1,65E-01	0,78	-0,061840173	
Sterol-4-alpha-carboxylate 3-dehydrogenase, decarboxylating	NSDHL	72449000	93583000	1,25	0,97	-0,04	6,8076	-0,1108224	-0,01584086	0,005902907	0,003889986	-0,02060385	-0,01584086	-0,1686254	-0,01552	-0,07561126	-0,08978924	NaN	NaN	2,30E-02	1,64	-0,050286096	+
Leukotriene A-4 hydrolase	LTA4H	29644000	34604000	1,25	0,97	-0,04	18,33	-0,4743119	-0,0505024	NaN	-0,02508894	NaN	NaN	0,04977013	-0,003538924	NaN	NaN	NaN	0,1519886	5,36E-01	0,27	-0,058613906	
Actin-related protein 2	ACTR2	216550000	260240000	1,26	0,97	-0,04	15,556	-0,02854292	0,08990479	-0,1954673	-0,08491516	-0,03309705	-0,03147417	-0,2424305	-0,02322578	-0,1101527	-0,01734407	NaN	NaN	5,21E-02	1,28	-0,067674487	
Elongation factor G, mitochondrial	GFM1	57477000	72176000	1,32	0,97	-0,04	13,375	0,2023863	-0,2302791	NaN	-0,08318704	0,1259155	0,06364096	-0,2658822	-0,03311184	-0,04412186	-0,2042165	NaN	-0,0297942	3,31E-01	0,48	-0,049864982	
Peroxiredoxin-4	PRDX4	277400000	372770000	1,30	0,97	-0,04	9,1362	0,01192487	-0,0566112	-0,03853962	-0,03411604	-0,1110561	-0,03893976	-0,1766582	-0,06036716	-0,1656448	-0,1118198	-0,1357286	-0,1124591	3,41E-04	3,47	-0,085834636	+
L-xylulose reductase	DCXR	55764000	70043000	1,31	0,97	-0,04	10,897	-0,02222922	-0,1519069	0,095722	0,1265764	-0,08708956	-0,01741709	-0,2743548	NaN	-0,09768709	NaN	NaN	-0,3383416	1,36E-01	0,87	-0,085191982	
14-3-3 protein theta	YWHAQ	348260000	491160000	1,42	0,97	-0,04	13,662	-0,0766454	0,03689012	-0,01387308	-0,0598558	-0,04883003	-0,03455926	-0,03367287	-0,03723626	-0,04260539	-0,06612524	-0,03367287	-0,1142382	1,59E-03	2,80	-0,043702022	+
Thioredoxin-dependent peroxide reductase, mitochondrial	PRDX3	333910000	427960000	1,39	0,97	-0,04	13,289	0,002162436	-0,05530643	-0,0173002	-0,01680388	-0,1690308	-0,0650986	-0,08326348	-0,1416848	-0,02318183	-0,07828952	0,005615641	-0,1354117	2,87E-03	2,54	-0,064799427	+
AP-1 complex subunit gamma-1	AP1G1	53025000	71836000	1,25	0,97	-0,04	15,392	-0,05706139	0,05852425	NaN	0,002162436	-0,07093623	0,2353594	-0,07559603	0,2580967	-0,1792533	-0,02702801	NaN	0,06488286	6,44E-01	0,19	0,020915075	
ATP-dependent RNA helicase DDX19A	DDX19A	47571000	64002000	1,15	0,97	-0,04	12,898	-0,1180222	-0,08486919	-0,05343385	-0,2136546	-0,1816403	0,3913278	NaN	0,2147466	NaN	NaN	NaN	-0,1140352	7,97E-01	0,10	-0,019947616	
Exportin-5	XPO5	565880000	633000000	1,13	0,97	-0,04	10,46	-0,03597851	0,04362443	-0,0544073	0,005615641	0,01077991	-0,05114498	-0,1510096	-0,1632033	-0,2313626	-0,1071971	-0,1135357	-0,1125371	5,67E-03	2,25	-0,080029679	+

Chromodomain-helicase-DNA-binding protein 4	CHD4	19321000	22448000	1,37	0,97	-0,04	18,852	NaN	NaN	0,534958	NaN	-0,0407937	0,1423481	0,4070807	NaN	0,01349801	-0,05784214	-0,3138177	0,1938348	2,90E-01	0,54	0,109908269	
CAD protein;Glutamine-dependent carbamoyl-phosphate synthase;Aspartate carbamoyltransferase;Dihydroorotase	CAD	663960000	748200000	1,13	0,97	-0,04	10,979	0,001153714	-0,009408136	-0,1784204	-0,1150036	0,008200003	-0,02070626	-0,1046818	-0,1066844	-0,0450146	-0,001847847	-0,05356861	-0,04672732	6,68E-03	2,17	-0,056059109	+
Activator of 90 kDa heat shock protein ATPase homolog 1	AHSA1	165370000	194260000	1,26	0,97	-0,04	6,9275	0,05380643	0,01635366	0,02005774	-0,1202315	-0,04981533	0,0116388	NaN	-0,3756839	-0,3083408	-0,147282	-0,01584086	-0,006043124	7,89E-02	1,10	-0,083761898	
Signal transducer and activator of transcription 5A	STAT5A	388680000	448280000	1,20	0,97	-0,04	13,595	0,02219017	0,008343471	-0,02535321	-0,03979969	-0,07293796	-0,08089645	-0,1637366	-0,3738132	-0,3882421	-0,2769221	-0,203668	-0,3051822	3,53E-03	2,45	-0,158334819	+
6-phosphogluconolactonase	PGLS	171690000	235980000	1,39	0,97	-0,04	10,587	0,07628538	-0,0302656	-0,06630655	0,007051912	0,004752986	-0,05845792	-0,0920633	-0,118398	0,05380643	-0,08340105	-0,06398217	0,07066408	2,19E-01	0,66	-0,025026155	
Splicing factor 1	SF1	45447000	47072000	1,42	0,97	-0,04	27,664	0,03492	NaN	-0,1077878	-0,1312974	NaN	-0,1202315	NaN	0,4233092	0,2157407	0,3144065	0,4269625	NaN	1,68E-01	0,78	0,132002773	
Ras-related C3 botulinum toxin substrate 1	RAC1	230900000	288390000	1,33	0,97	-0,05	5,0969	-0,2186487	-0,1060631	-0,01518461	-0,07673672	0,01035019	-0,1353483	-0,06904317	0,1309309	-0,002975011	0,1681285	-0,07617383	0,1914362	6,55E-01	0,18	-0,016610637	
Histidine--tRNA ligase, cytoplasmic	HARS	86553000	110710000	1,31	0,97	-0,05	18,001	0,007195495	0,03745244	0,1428708	-0,04568457	-0,1720184	0,008486926	NaN	-0,1795963	NaN	NaN	-0,07271031	0,1355349	7,01E-01	0,15	-0,015385452	
T-complex protein 1 subunit theta	CCT8	5171400000	6653900000	1,23	0,97	-0,05	7,6995	-0,08228566	-0,07515515	-0,08016421	-0,1154724	-0,05957008	-0,1221458	-0,04355666	-0,007782617	NaN	NaN	-0,03568267	-0,05139919	1,94E-04	3,71	-0,06732144	+
Phosphoribosylformylglycinamidine synthase	PFAS	498400000	525370000	1,08	0,97	-0,05	14,862	-0,07346918	-0,02287401	-0,2099787	-0,1036119	-0,1309815	-0,01482019	-0,1711248	-0,1005614	-0,1326095	-0,1281721	-0,08130842	-0,040126	9,52E-05	4,02	-0,100803138	+
Transcription elongation factor B polypeptide 1	TCEB1	53803000	66931000	1,20	0,97	-0,05	7,9362	-0,0951113	-0,09978814	-0,1695337	-0,1365055	-0,1399192	-0,09141763	-0,2877652	0,05505682	NaN	0,1149001	0,1015157	0,03941911	1,21E-01	0,92	-0,064468087	
Mitochondrial import inner membrane translocase subunit TIM14	DNAJC19	39143000	54754000	1,33	0,97	-0,05	14,689	-0,08169013	0,08065767	NaN	0,08691968	0,1483484	0,169412	-0,000966953	0,1285573	0,08814155	NaN	-0,04017048	0,05810868	3,58E-02	1,45	0,063731777	
General vesicular transport factor p115	USO1	87771000	111750000	1,26	0,97	-0,05	18,191	0,09288389	0,1978653	0,09653179	0,2253984	0,06322686	0,2479275	-0,160379	-0,1607176	-0,0967766	-0,2277765	-0,1597665	-0,3195496	7,69E-01	0,11	-0,016760988	
Pentatricopeptide repeat domain-containing protein 3, mitochondrial	PTCD3	71409000	90685000	1,36	0,97	-0,05	11,345	NaN	-0,09280171	NaN	0,08038475	NaN	-0,03612646	-0,2160992	0,0247452	0,144177	0,158854	-0,1806588	0,1585955	9,27E-01	0,03	0,004563368	
Acyl-protein thioesterase 1	LYPLA1	139020000	171890000	1,29	0,96	-0,05	7,4711	-0,1106822	-0,08814742	0,04334458	-0,1069796	-0,01206797	-0,1668751	0,2327835	-0,0966532	-0,2363334	NaN	0,0766959	-0,03659984	2,66E-01	0,57	-0,045592252	
L-lactate dehydrogenase B chain	LDHB	2079100000	2748900000	1,31	0,96	-0,05	6,1976	-0,09343258	-0,09924714	0,01720924	-0,1628156	-0,08031666	-0,1114457	-0,1130051	-0,1251322	-0,07334775	-0,1283456	-0,04983019	-0,09360196	1,97E-05	4,70	-0,092775935	+
Transducin beta-like protein 3	TBL3	11440000	12757000	1,39	0,96	-0,05	19,545	NaN	NaN	NaN	NaN	-0,802663	-0,4958984	-0,3358085	0,03506084	-0,05935967	-0,01835189	-0,8677259	NaN	4,19E-02	1,38	-0,363535217	
Deoxyuridine 5-triphosphate nucleotidohydrolase, mitochondrial	DUT	159210000	224630000	1,31	0,96	-0,05	13,566	-0,07570242	-0,05207204	-0,1766093	-0,1542169	-0,1132079	-0,1855726	0,2088917	0,04865466	0,1088924	0,2005043	0,04278438	0,05144163	8,45E-01	0,07	-0,008017676	
Vacuolar protein sorting-associated protein 35	VPS35	379610000	447270000	1,22	0,96	-0,05	8,9093	-0,1111497	-0,1183354	-0,04174378	-0,06577796	0,003889986	-0,05404775	-0,1230411	-0,02404702	0,01364095	-0,08785604	0,02048441	-0,01074471	6,72E-03	2,17	-0,049894004	+
Heterogeneous nuclear ribonucleoprotein H;Heterogeneous nuclear ribonucleoprotein H, N-terminally processed	HNRNPH1	1099900000	1372900000	1,38	0,96	-0,05	22,286	-0,3122942	-0,2661598	-0,02149682	-0,1835231	-0,2910447	-0,4089717	-0,02137969	0,1579492	0,2537472	0,0275787	-0,02676343	0,1844707	2,49E-01	0,60	-0,075657317	
Inosine-5-monophosphate dehydrogenase 2	IMPDH2	963700000	1281300000	1,22	0,96	-0,05	8,995	-0,01160256	-0,08327878	-0,06318308	-0,1097945	0,001586012	-0,02997102	-0,1892196	-0,1485608	NaN	NaN	-0,1047749	-0,07719323	1,96E-03	2,71	-0,081599259	+
Peptidyl-prolyl cis-trans isomerase FKBP4;Peptidyl-prolyl cis-trans isomerase FKBP4, N-terminally processed	FKBP4	1102000000	1364000000	1,23	0,96	-0,05	14,592	-0,0312383	0,002162436	-0,04990488	-0,09819667	0,01421241	-0,01898031	-0,1761365	-0,1120693	NaN	NaN	-0,08903711	-0,04400288	9,78E-03	2,01	-0,060319106	+
Basic leucine zipper and W2 domain-containing protein 1	BZW1	61869000	82903000	1,29	0,96	-0,05	18,205	NaN	NaN	NaN	0,04809668	NaN	-0,1131768	NaN	NaN	NaN	NaN	-0,456326	-0,10293	2,40E-01	0,62	-0,156084009	
Replication factor C subunit 2	RFC2	67155000	88867000	1,28	0,96	-0,05	3,1678	0,08637615	0,0244615	0,1629835	0,0734089	-0,09312481	0,05686093	-0,2404521	-0,05584622	-0,1915573	-0,03667384	NaN	NaN	6,09E-01	0,22	-0,02135633	
Tubulin beta chain	TUBB	4877300000	6267900000	1,31	0,96	-0,05	5,6953	-0,07126965	-0,1163948	-0,1537514	-0,1695661	-0,05632621	-0,1471542	0,03139513	0,07642223	0,08011194	0,1290852	0,108491	0,1407786	7,26E-01	0,14	-0,0123482	
Mitochondrial import receptor subunit TOM40 homolog	TOMM40	244960000	292040000	1,32	0,96	-0,05	11,975	0,3160298	-0,006840117	0,1267086	-0,02476601	-0,1147848	0,01777946	-0,3097528	0,00302647	-0,1778328	-0,2341258	NaN	NaN	4,98E-01	0,30	-0,040455809	
Lanosterol synthase	LSS	36634000	38629000	1,08	0,96	-0,06	20,256	-0,232396	-0,2828072	-0,1643508	NaN	-0,08850012	NaN	NaN	0,03506084	NaN	0,2418402	NaN	NaN	3,49E-01	0,46	-0,081858852	
Heat shock protein HSP 90-alpha	HSP90AA1	8240300000	9963000000	1,17	0,96	-0,06	7,5676	-0,085956	-0,03137099	-0,04230827	-0,04701051	-0,09118704	-0,0628967	-0,07073925	-0,07705625	-0,04572922	-0,04832265	-0,0377101	0,02573757	1,36E-04	3,87	-0,051212451	+
Puromycin-sensitive aminopeptidase	NPEPPS	376660000	440360000	1,25	0,96	-0,06	23,538	-0,05821759	-0,05797731	-0,1975503	-0,02181902	0,09044699	0,05325044	-0,005927245	-0,03070764	0,068602	-0,04328912	-0,05934462	-0,05090587	2,56E-01	0,59	-0,02611994	
Chloride intracellular channel protein 1	CLIC1	2805200000	3796100000	1,29	0,96	-0,06	9,392	-0,1074147	-0,2163171	-0,09545036	-0,09754815	-0,06065305	-0,119965	-0,04790505	-0,07051194	-0,02696922	-0,04587816	-0,04348239	-0,05844288	1,60E-04	3,80	-0,082544827	+
26S protease regulatory subunit 6A	PSMC3	320910000	414770000	1,22	0,96	-0,06	11,46	0,01806431	-0,07433474	-0,1776696	-0,09370968	-0,09901539	0,000865329	NaN	-0,05517152	NaN	NaN	-0,02304988	-0,06245978	1,26E-02	1,90	-0,062942326	+
Transcription elongation factor B polypeptide 2	TCEB2	71151000	87116000	1,26	0,96	-0,06	23,701	0,2220626	-0,1020316	-0,1683821	-0,1887262	0,2141248	-0,05847296	-0,104263	-0,05703142	-0,1252738	-0,01693525	-0,3546672	-0,4453621	1,04E-01	0,98	-0,098746538	
Elongation factor 1-delta	EEF1D	341080000	381360000	1,20	0,96	-0,06	14,305	-0,07605218	-0,02240502	-0,2298391	-0,02885196	-0,1670695	-0,2188166	-0,06868005	-0,03729556	-0,4285853	-0,4861252	-0,0909566	0,09828482	1,30E-02	1,89	-0,146366021	+
39S ribosomal protein L22, mitochondrial	MRPL22	28737000	32856000	1,29	0,96	-0,06	18,395	NaN	-0,1501608	-0,5216183	NaN	-0,3257744	-0,4008155	0,4150014	0,5920624	0,7304009	NaN	NaN	0,1394695	7,35E-01	0,13	0,059820645	
Phosphoglucomutase-2	PGM2	103310000	112520000	1,11	0,96	-0,06	25,768	-0,2536529	NaN	-0,2185311	0,01421241	-0,3194956	0,05907837	-0,06101425	-0,1942618	0,1440464	-0,09995826	-0,05902898	NaN	6,31E-02	1,20	-0,098860572	
Peptidyl-tRNA hydrolase 2, mitochondrial	PTRH2	37346000	41361000	1,28	0,96	-0,06	7,042	-0,2238118	-0,2544787	NaN	-0,06537031	0,06060078	NaN	-0,05742169	-0,09014243	-0,01877566	-0,1011648	NaN	NaN	3,68E-02	1,43	-0,093820572	
NudC domain-containing protein 1	NUDCD1	23633000	31291000	1,22	0,96	-0,06	18,461	NaN	-0,1973022	0,0938305	NaN	NaN	-0,0196383	NaN	0,2663968	NaN	NaN	NaN	0,1737671	4,74E-01	0,32	0,063410781	
Signal recognition particle subunit SRP72	SRP72	126140000	147020000	1,18	0,96	-0,06	14,428	-0,2683475	0,001874253	-0,1852773	0,1393385	-0,0804845	-0,2321588	-0,07552001	NaN	0,40523	0,1067503	-0,2848797	0,01077991	5,16E-01	0,29	-0,042063163	
Ras-related protein Rab-5C	RAB5C	187480000	251470000	1,41	0,96	-0,06	6,1631	0,0321009	0,08556063	0,1376347	0,000432815	0,05644473	-0,000187559	-0,1225227	-0,1926451	-0,09938624	-0,04049687	-0,5369635	-0,1078499	2,24E-01	0,65	-0,065656511	
Cullin-associated NEDD8-dissociated protein 1	CAND1	848760000	952290000	1,14	0,96	-0,06	10,73	-0,06706197	-0,01680388	-0,09003497	0,02062666	0,009203812	0,03337007	-0,1909478	-0,2037013	-0,2574246	-0,2057464	-0,2089278	-0,1668103	3,59E-03	2,44	-0,112021534	+
Cytochrome c oxidase subunit 5A, mitochondrial	COX5A	82626000	103550000	1,27	0,96	-0,06	11,157	0,10742	-0,06980012	0,01135251	-0,0819191	0,02460336	-0,000966953	-0,1164105	-0,02805736	-0,3004839	-0,2627113	0,04739887	-0,05823264	1,08E-01	0,97	-0,06065059	
UBX domain-containing protein 1	UBXN1	67651000	75113000	1,09	0,96	-0,06	10,465	NaN	NaN	NaN	-0,1896473	NaN	-0,04132808	NaN	NaN	-0,4049547	-0,04086792	NaN	NaN	1,44E-01	0,84	-0,16919952	
Leukocyte elastase inhibitor	SERPINB1	392580000	477130000	1,12	0,96	-0,06	13,406	-0,2535152	-0,09549666	-0,03873226	0,01035019	-0,06447989	-0,01359632	-0,1289133	NaN	0,1764505	-0,02785141	NaN	NaN	2,44E-01	0,61	-0,048420493	
DNA-dependent protein kinase catalytic subunit	PRKDC	3566200000	4002400000	1,15	0,96	-0,06	11,604	-0,03191658	-0,07620429	-0,06482694	-0,01168977	-0,03674785	-0,03393872	-0,102202	-0,11313	-0,05374826	-0,07694977	-0,08603258	-0,03340707	3,88E-05	4,41	-0,060066149	+
Acetyl-CoA acetyltransferase, cytosolic	ACAT2	582900000	802860000	1,28	0,96	-0,06	10,669	-0,06185728	-0,001356807	0,068602	0,0647449	-0,06215845	0,03632757	-0,1162072	-0,1606854	-0,1518107	-0,06576284	NaN	NaN	1,27E-01	0,90	-0,04501642	
Sodium/potassium-transporting ATPase subunit beta-3	ATP1B3	113880000	145760000	1,28	0,96	-0,06	9,074	0,2759607	0,1368475	0,02672926	-0,04158048	-0,01298482	0,08420031	-0,2786011	NaN	-0,1897133	-0,1250378	NaN	NaN	8,14E-01	0,09	-0,013797746	
Coiled-coil domain-containing protein 47	CCDC47	87137000	113130000	1,19	0,96	-0,06	27,872	-0,02619037	0,0790197	-0,02657239	NaN	-0,06541557	-0,1218004	NaN	NaN	NaN	NaN	-0,2614484	-0,1704589	9,01E-02	1,05	-0,084695183	
Insulin-like growth factor 2 mRNA-binding protein 1	IGF2BP1	377770000	381740000	1,24	0,96	-0,07	21,189	-0,4789493	-0,5207073	-0,01861494	-0,2022566	-0,3782318	-0,4841866	0,1285573	0,2087669	0,3064956	0,1719754	0,2903065	-0,03053083	3,78E-01	0,42	-0,083947969	
Calcineurin B homologous protein 1	CHP1	21340000	46626000	1,33	0,96	-0,07	10,981	-0,1099969	-0,1209686	NaN	0,02856912	0,9137989	NaN	NaN	0,1318529	0,09936258	0,03139513	-0,227388	-0,002844902	4,76E-01	0,32	0,082642238	
Lysine--tRNA ligase	KARS	841390000	961520000	1,12	0,96	-0,07	10,059	-0,08558859	-0,1628641	-0,1665998	-0,1472181	0,01221106	-0,1309182	-0,200863	-0,02826333	-0,1464993	-0,1378065	-0,02757204	-0,02299125	3,36E-04	3,47	-0,103747762	+
Myosin-10	MYH10	496230000	552200000	1,10	0,96	-0,07	19,251	-0,4137492	-0,3945057	0,1152996	0,07505342	-0,44906	-0,2921925	-0,08682908	-0,03311184	-0,02114538	0,009633875	0,003602319	-0,08074384	4,56E-02	1,34	-0,130645696	
Heterogeneous nuclear ribonucleoproteins C1/C2;Heterogeneous nuclear ribonucleoprotein C-like 4;Heterogeneous nuclear ribonucleoprotein C-like 1;Heterogeneous nuclear ribonucleoprotein C-like 3;Heterogeneous nuclear ribonucleoprotein C-like 2	NRNPCL4;HNRNPCL1;HNRNPCL3;	358610000	465610000	1,42	0,96	-0,07	12,007	-0,07620429	0,05352838	0,2593026	0,3145225	-0,1703615	-0,1463876	0	-0,04791998	-0,104108	-0,09438723	0,5869809	0,5881329	2,46E-01	0,61	0,0969249	
Basic leucine zipper and W2 domain-containing protein 2	BZW2	205630000	255130000	1,21	0,95	-0,07	6,1689	0,03871708	-0,02017955	-0,09768709	-0,1540404	-0,1032709	-0,06188735	-0,09131008	-0,10967	0,2806	0,1567849	-0,09899992	-0,1816566	3,64E-01	0,44	-0,036883319	
Glycerol-3-phosphate dehydrogenase, mitochondrial	GPD2	21725000	30008000	1,19	0,95	-0,07	24,626	0,07778991	NaN	-0,1019542	0,4206172	-0,3493825	NaN	0,154324	NaN	NaN	NaN	-0,6860576	NaN	6,36E-01	0,20	-0,080777198	
Ubiquitin carboxyl-terminal hydrolase 5	USP5	125650000	146090000	1,29	0,95	-0,07	17,135	-0,1398079	0,04432424	-0,05727155	-0,05760183	-0,001876691	-0,03373193	-0,08873027	0,04823624	-0,197964	-0,05252081	0,003889986	-0,1719371	2,66E-02	1,58	-0,05874931	+
Serine/threonine-protein phosphatase PGAM5, mitochondrial	PGAM5	33920000	44643000	1,18	0,95	-0,07	11,962	-0,2293993	-0,1606531	0,1497797	0,21661	NaN	-0,5401068	-0,1930244	-0,1709948	-0,05295482	-0,05273031	NaN	0,04488368	1,80E-01	0,75	-0,098859007	
Branched-chain-amino-acid aminotransferase, cytosolic	BCAT1	158390000	185600000	1,16	0,95	-0,07	10,21	-0,1116172	0,06350289	-0,0407937	0,02814484	-0,01254818	-0,001992332	-0,1791063	-0,05488672	-0,0208673	-0,06689578	NaN	NaN	1,04E-01	0,98	-0,039705973	
Prolyl endopeptidase	PREP	57070000	68318000	1,23	0,95	-0,07	7,7218	0,1194226	-0,1154412	0,09058251	-0,08474681	-0,05246097	-0,07590009	NaN	0,2231754	-0,239498	NaN	NaN	NaN	7,59E-01	0,12	-0,016858319	
Catechol O-methyltransferase	COMT	292970000	380270000	1,25	0,95	-0,07	10,12	-0,1561287	-0,1915738	-0,1606531	-0,1867708	-0,184064	-0,2258687	0,1487389	0,2657969	0,1226065	0,1024567	0,2854613	0,1043367	9,13E-01	0,04	-0,006305162	
Vesicle-associated membrane protein-associated protein A	VAPA	131660000	160760000	1,17	0,95	-0,07	25,21	-0,07286207	-0,1603951	-0,1057681	-0,07045136	-0,09089515	-0,1776369	0,2606279	0,1556197	0,07148806	-0,05121974	0,01421241	0,1433935	8,63E-01	0,06	-0,006990581	
Farnesyl pyrophosphate synthase	FDPS	193700000	231650000	1,21	0,95	-0,07	8,7164	-0,1965085	-0,06821108	0,1327743	0,03294719	0,06557227	-0,01431013	-0,3176964	-0,1124591	-0,2438819	-0,1963927	NaN	NaN	7,92E-02	1,10	-0,0918166	
Keratin, type I cytoskeletal 18	KRT18	676700000	809450000	1,16	0,95	-0,07	11,679	-0,1411915	-0,06772712	0,05990899	0,01706665	-0,192233	-0,0708301	-0,1355226	NaN	-0,1371717	0,07970238	-0,178502	-0,2206814	1,60E-02	1,79	-0,089743754	+
U4/U6.U5 tri-snRNP-associated protein 2	USP39	32220000	51160000	1,23	0,95	-0,07	13,074	NaN	-0,2594264	NaN	NaN	-0,6189824	-0,4683715	0,2826177	NaN	NaN	NaN	0,1606621	0,2035144	4,90E-01	0,31	-0,116664357	
39S ribosomal protein L15, mitochondrial	MRPL15	21482000	26225000	1,02	0,95	-0,07	17,348	0,2791741	-0,5106832	0,06639916	-0,187395	-0,3949608	0,2923105	-0,3345708	NaN	NaN	NaN	-0,09018847	NaN	3,39E-01	0,47	-0,10998931	
Hemoglobin subunit gamma-1	HBG1	135130000	164400000	1,28	0,95	-0,07	19,532	0,1791288	0,02955887	-0,2711478	-0,2266957	-0,1068708	-0,07233107	-0,2539453	-0,0920941	-0,5388277	-0,4897064	-0,2687125	-0,4265285	5,39E-03	2,27	-0,211514341	+
Fumarylacetoacetase	FAH	271050000	331210000	1,20	0,95	-0,08	6,1832	-0,1101527	-0,06568733	-0,02401773	-0,06122489	-0,1794329	-0,07594576	0,1427401	0,1264442	0,2462257	0,2532632	NaN	NaN	6,17E-01	0,21	0,025221189	
ADP-ribosylation factor 4	ARF4	66930000	82687000	1,28	0,95	-0,08	7,6787	NaN	0,0908535	0,01906128	-0,04187745	0,1063484	0,08133958	-0,2604975	-0,1853102	-0,1373781	-0,1923648	-0,1055353	-0,138791	1,02E-01	0,99	-0,069468319	
Uridine 5-monophosphate synthase;Orotate phosphoribosyltransferase;Orotidine 5-phosphate decarboxylase	UMPS	43765000	45217000	1,12	0,95	-0,08	28,339	-0,1468986	0,05380643	NaN	NaN	-0,5143877	-0,3606011	NaN	NaN	NaN	NaN	-0,04523792	-0,2752624	5,40E-02	1,27	-0,214763543	
Protein RCC2	RCC2	214800000	262590000	1,15	0,95	-0,08	12,429	-0,1222715	-0,07140609	-0,1335588	-0,1321035	-0,2687473	-0,3164742	0,2225573	-0,1125371	NaN	NaN	0,1779815	0,1231364	2,99E-01	0,53	-0,063342312	
U5 small nuclear ribonucleoprotein 200 kDa helicase	SNRNP200	406250000	455350000	1,15	0,95	-0,08	18,546	-0,1830972	-0,1119602	0,06942715	0,1202192	-0,3789445	-0,3812914	-0,01632233	-0,1197612	-0,1329575	-0,05160844	-0,06677482	0,01891905	4,31E-02	1,37	-0,102846018	
Exportin-1	XPO1	868980000	962000000	1,13	0,95	-0,08	17,844	-0,02139436	-0,06494766	-0,02306456	-0,04248653	0,02332628	0,01206797	-0,2815266	-0,2922985	-0,2901447	-0,2791962	-0,2429426	-0,2932352	3,24E-03	2,49	-0,149653555	+
Nuclear migration protein nudC	NUDC	735200000	907080000	1,28	0,95	-0,08	17,045	-0,4432223	-0,1747028	-0,02244895	-0,01893649	-0,1843918	0,02332628	-0,1459085	-0,04153587	-0,04951663	-0,1257933	NaN	NaN	2,16E-02	1,67	-0,118313029	+
Heterogeneous nuclear ribonucleoprotein D0	HNRNPD	1958900000	2380600000	1,25	0,95	-0,08	12,73	-0,2458479	-0,2136713	-0,05802234	-0,09300172	-0,195236	-0,1684957	0,2507191	0,02701252	0,2590615	0,1518587	0,05783146	0,05491792	7,82E-01	0,11	-0,014406143	
Importin-11	IPO11	94343000	99313000	1,02	0,95	-0,08	11,037	-0,1772127	-0,1036584	-0,09015781	-0,07570242	0,06281248	-0,06570245	-0,3177144	-0,2091946	NaN	NaN	NaN	-0,1149724	8,91E-03	2,05	-0,12127808	+
Ubiquitin-like protein 4A	UBL4A	34076000	42214000	1,22	0,95	-0,08	9,1828	-0,1589608	-0,0141644	0,01206797	0,05727703	0,1095611	-0,06233919	-0,1351581	0,05032762	NaN	NaN	NaN	NaN	6,15E-01	0,21	-0,017673598	
Coatomer subunit beta	COPB2	109100000	116730000	1,08	0,95	-0,08	10,233	-0,3965363	-0,1092654	-0,3643849	0,09261344	-0,2642526	-0,05196738	-0,1306497	0,04040144	-0,07935611	0,03618686	-0,03393872	0,1244606	7,99E-02	1,10	-0,094724071	
Monofunctional C1-tetrahydrofolate synthase, mitochondrial	MTHFD1L	55741000	58364000	1,12	0,95	-0,08	26,502	0,2015084	-0,1064359	0,06225996	-0,07888366	-0,05649119	-0,2676526	-0,1807897	0,09207205	NaN	-0,6326513	NaN	NaN	2,22E-01	0,65	-0,107451551	
Exosome complex component RRP41	EXOSC4	29171000	35974000	1,18	0,95	-0,08	11,567	-0,3562915	NaN	-0,02689571	-0,03808034	-0,1273367	NaN	NaN	0,4263184	NaN	-0,02149682	NaN	0,2953704	8,34E-01	0,08	0,02165539	
Transforming protein RhoA	RHOA	120290000	162620000	1,34	0,94	-0,08	7,4207	-0,01582626	-0,08164426	-0,09586661	-0,02579375	-0,1030539	0,04990952	-0,1893513	-0,2232222	-0,2048482	-0,1722135	-0,1999678	-0,2156635	6,82E-04	3,17	-0,123128472	+
S-adenosylmethionine synthase isoform type-2	MAT2A	453820000	531340000	1,13	0,94	-0,08	4,7761	0,0610158	0,05018827	-0,1665836	-0,08347749	-0,2474055	-0,1603306	0,03843611	-0,1017839	NaN	NaN	-0,3603233	-0,3717957	2,57E-02	1,59	-0,134205995	+
40S ribosomal protein S15	RPS15	47797000	64045000	1,35	0,94	-0,08	19,047	-1,014121	-0,9978952	NaN	NaN	NaN	NaN	-0,1328152	-0,08736544	0,01321208	-0,04264994	-0,05451214	0,08691968	1,26E-01	0,90	-0,278653358	
Mitochondrial ribonuclease P protein 1	TRMT10C	66156000	83708000	1,21	0,94	-0,08	8,632	NaN	-0,06393687	0,1677432	-0,4124813	0,02630426	-0,005680922	-0,1342553	-0,04818847	0,01506926	-0,1012731	NaN	NaN	2,74E-01	0,56	-0,06185547	
39S ribosomal protein L9, mitochondrial	MRPL9	19092000	23666000	1,30	0,94	-0,08	9,0426	-0,2512297	NaN	-0,270469	-0,01428103	-0,2873074	-0,08525184	-0,07717799	NaN	NaN	NaN	0,04795711	0,1047392	9,14E-02	1,04	-0,104127574	
Eukaryotic peptide chain release factor subunit 1	ETF1	355120000	466180000	1,24	0,94	-0,08	13,68	0,02034214	0,001297885	-0,05979566	0,01806431	-0,05285001	-0,1276519	-0,1712547	-0,2443261	NaN	NaN	-0,133448	-0,1828843	1,09E-02	1,96	-0,093250646	+
Protein phosphatase 1G	PPM1G	51292000	70469000	1,26	0,94	-0,08	16,734	NaN	0,1248574	-0,07192156	-0,0575868	-0,2703994	NaN	-0,02721912	NaN	NaN	-0,03413074	NaN	0,1445685	6,19E-01	0,21	-0,027404528	
Eukaryotic translation initiation factor 1A, X-chromosomal;Eukaryotic translation initiation factor 1A, Y-chromosomal	EIF1AX;EIF1AY	42800000	49155000	1,44	0,94	-0,09	17,056	-0,3252321	-0,3813479	-0,4413601	-0,3050039	-0,2727678	-0,4700691	-0,01700825	0,04278438	-0,0298237	0,1982427	0,1821838	0,07614867	6,49E-02	1,19	-0,145271108	
Hemoglobin subunit zeta	HBZ	1713400000	2318100000	1,36	0,94	-0,09	6,5505	0,06915226	0,03829577	0,06970215	0,06018582	-0,06255011	0,03167748	-0,1553251	-0,08800939	-0,172555	-0,1590091	-0,1341604	-0,1337171	9,17E-02	1,04	-0,053026061	

SRA stem-loop-interacting RNA-binding protein, mitochondrial	SLIRP	99252000	122880000	1,21	0,94	-0,09	5,6522	-0,0316954	0,02304246	-0,04965102	-0,1157069	-0,1787633	-0,08721215	0,01934605	-0,1181474	-0,003191915	-0,1758106	-0,06992124	0,07751644	2,70E-02	1,57	-0,05918291	+
Acylamino-acid-releasing enzyme	APEH	105040000	125800000	1,25	0,94	-0,09	17,403	-0,1608789	-0,3844143	0,196733	-0,1733356	-0,08489984	0,07888308	-0,05476685	-0,1714172	0,1142337	0,02899346	0,1422174	-0,1505451	3,12E-01	0,51	-0,05159976	
COP9 signalosome complex subunit 8	COPS8	49322000	66636000	1,23	0,94	-0,09	11,341	-0,1876414	-0,07760424	-0,2109135	-0,3065554	-0,04616119	-0,2579765	0,79693	0,4656089	0,1628546	0,22231	0,1685137	0,01834928	5,20E-01	0,28	0,062309524	
Serine/threonine-protein phosphatase 2B catalytic subunit alpha isoform;Serine/threonine-protein phosphatase 2B catalytic subunit beta isoform	PPP3CA;PPP3CB	65102000	87555000	1,28	0,94	-0,09	9,6215	-0,1737261	-0,07768038	NaN	-0,192777	0,09855437	-0,1449671	NaN	NaN	NaN	NaN	-0,3291235	-0,2440015	2,51E-02	1,60	-0,151960169	+
DnaJ homolog subfamily A member 2	DNAJA2	43203000	48899000	1,32	0,94	-0,09	15,468	-0,466636	-0,2496336	NaN	-0,252415	NaN	-0,3073764	0,05311137	0,1257833	0,02545406	NaN	NaN	NaN	1,17E-01	0,93	-0,15310176	
Vesicle-associated membrane protein 7	VAMP7	42148000	27927000	1,28	0,94	-0,09	12,896	-0,1475696	-0,03719188	NaN	-0,08411971	-0,1128335	-0,09069541	NaN	-0,1161602	-0,05238625	-0,05823264	NaN	-0,02929356	2,92E-04	3,53	-0,080942533	+
Small nuclear ribonucleoprotein Sm D3	SNRPD3	248210000	311370000	1,19	0,94	-0,09	13,998	-0,07599136	-0,1107757	0,1500396	0,1569143	-0,2443946	-0,1837689	-0,08462442	0,3700527	0,05185929	-0,01410613	-0,04382434	0,05783146	9,61E-01	0,02	0,002434323	
X-ray repair cross-complementing protein 5	XRCC5	1102400000	1242300000	1,07	0,94	-0,09	14,115	-0,2144578	-0,1469465	-0,1865574	-0,0925555	-0,2684865	-0,1413347	0,2332746	0,2218153	0,1597583	0,2249046	0,3381105	0,1460035	7,21E-01	0,14	0,022794034	
NADH dehydrogenase [ubiquinone] 1 beta subcomplex subunit 6	NDUFB6	46897000	55089000	1,22	0,94	-0,09	10,561	NaN	-0,05524648	NaN	-0,08586418	0,09017591	-0,03413074	NaN	NaN	0,08528864	-0,08903711	NaN	NaN	6,77E-01	0,17	-0,014802327	
Adenine phosphoribosyltransferase	APRT	575280000	734820000	1,32	0,94	-0,09	9,7495	-0,1671343	-0,1509295	-0,1736447	-0,1092031	-0,1464354	-0,1749797	0,03351105	-0,0232111	0,009347181	0,09626186	-0,01282465	0,05990899	5,22E-02	1,28	-0,063277789	
Glutaredoxin-3	GLRX3	564370000	704020000	1,26	0,94	-0,09	9,1479	-0,003871618	0,03337007	0,01535482	-0,1614919	-0,08727354	-0,08234675	-0,2373536	-0,08632359	-0,1627349	-0,1147068	0,131326	-0,1621536	2,68E-02	1,57	-0,076517119	+
39S ribosomal protein L14, mitochondrial	MRPL14	23480000	27189000	1,14	0,94	-0,09	2,1121	-0,1180222	-0,04720429	-0,2158646	-0,1424648	-0,05223662	-0,05232633	NaN	-0,2323113	-0,04215969	NaN	NaN	NaN	4,58E-03	2,34	-0,11282373	+
Heat shock protein HSP 90-beta	HSP90AB1	37995000000	4,2961E+10	1,17	0,94	-0,09	10,048	-0,1526926	-0,1427356	-0,1724574	-0,1682525	-0,1683821	-0,1480331	-0,07992015	-0,03089924	-0,05169819	-0,06781787	-0,02419374	-0,01006177	1,69E-04	3,77	-0,101428682	+
Parafibromin	CDC73	8462000	10004000	1,32	0,94	-0,09	15,969	-0,1773106	NaN	-0,3074478	NaN	-0,1957977	NaN	0,06942715	0,003458378	NaN	NaN	0,08447251	NaN	2,44E-01	0,61	-0,087199681	
Importin subunit alpha-4	KPNA3	65139000	75836000	1,13	0,94	-0,09	21,398	-0,1808387	-0,09252478	-0,3712544	-0,09451044	-0,09909266	-0,279669	NaN	0,3146386	NaN	NaN	0,1198209	0,1298765	4,17E-01	0,38	-0,061506011	
Core-binding factor subunit beta	CBFB	23226000	28588000	1,12	0,94	-0,09	12,37	-0,3503937	-0,2716005	-0,1395854	-0,2890514	-0,3402033	-0,2259193	0,1589833	0,1354035	0,0504668	0,05352838	0,02019986	NaN	9,55E-02	1,02	-0,10892471	
Prohibitin	PHB	1118300000	1370400000	1,25	0,94	-0,09	7,9375	-0,02817503	-0,1196201	-0,06041236	-0,1250221	-0,1812476	-0,1887756	-0,02136503	-0,08512932	0,06956466	-0,06514385	-0,1177248	-0,07867033	1,92E-03	2,72	-0,083476791	+
GTP-binding protein SAR1b	SAR1B	4901700	6660400	1,24	0,94	-0,10	2,1765	NaN	-0,07617383	NaN	-0,1441537	NaN	-0,03879162	NaN	0,2667565	0,09868897	-0,1359347	NaN	-0,2223804	5,90E-01	0,23	-0,035998393	
Lipopolysaccharide-responsive and beige-like anchor protein	LRBA	89852000	81525000	0,96	0,94	-0,10	10,744	-0,2253288	-0,1283456	-0,1517146	-0,2036182	-0,01499509	-0,09321709	NaN	NaN	-0,1435003	-0,1785183	NaN	NaN	5,08E-04	3,29	-0,142404758	+
Coronin-7	CORO7	63845000	72000000	1,07	0,94	-0,10	5,8385	-0,2038509	-0,1248963	-0,141446	-0,0493076	-0,05515651	-0,09674571	NaN	NaN	NaN	NaN	-0,04479132	-0,408378	1,34E-02	1,87	-0,140571534	+
Cell cycle and apoptosis regulator protein 2	CCAR2	436930000	472960000	1,07	0,93	-0,10	10,597	-0,1752077	-0,08138476	-0,1429427	-0,1016446	-0,1359823	-0,0860019	0,1842168	0,02389408	0,03294719	0,3502709	0,1873877	0,5242643	4,64E-01	0,33	0,048318089	
ADP/ATP translocase 3;ADP/ATP translocase 3, N-terminally processed	SLC25A6	133570000	146680000	1,08	0,93	-0,10	8,2858	-0,130397	-0,1203883	-0,2274048	-0,162751	-0,358306	-0,09750187	0,0878702	NaN	-0,2146755	-0,09768709	-0,1990897	-0,2321757	8,15E-04	3,09	-0,1593188	+
Prefoldin subunit 5	PFDN5	115970000	143870000	1,20	0,93	-0,10	6,1187	-0,05491674	-0,02195085	-0,06047253	-0,005521651	-0,04485093	-0,08664525	-0,2230875	-0,1468187	-0,07372727	-0,1575602	-0,08969718	-0,1233396	3,79E-04	3,42	-0,090715701	+
Tripeptidyl-peptidase 2	TPP2	148970000	151230000	1,01	0,93	-0,10	14,496	-0,2590292	-0,1570775	-0,1260609	-0,2062456	-0,0315037	-0,3662247	0,1094274	-0,1966407	-0,4029504	0,02389408	0,2136273	0,0408223	6,22E-02	1,21	-0,113163477	
Small nuclear ribonucleoprotein-associated proteins B and B;Small nuclear ribonucleoprotein-associated protein N	SNRPB;SNRPN	177860000	208350000	1,28	0,93	-0,10	19,22	-0,2392596	-0,3257744	-0,2860051	-0,1084875	-0,6868238	-0,3765826	0,1156992	0,201132	0,243791	0,2853431	0,4614238	0,1526378	6,42E-01	0,19	-0,046908862	
Complex III assembly factor LYRM7	LYRM7	16272000	22920000	1,21	0,93	-0,10	17,867	-0,02243428	-0,2532573	NaN	NaN	-0,07495759	NaN	0,271067	0,01249718	NaN	NaN	NaN	-0,003394284	8,72E-01	0,06	-0,011746542	
Splicing factor 3B subunit 5	SF3B5	14418000	16350000	1,14	0,93	-0,10	12,213	-0,2627805	-0,1049301	NaN	NaN	-0,6297241	-0,6398044	NaN	NaN	-0,08356924	0,04642113	NaN	NaN	6,66E-02	1,18	-0,279064533	
Transformer-2 protein homolog beta	TRA2B	35814000	41831000	0,97	0,93	-0,10	20,201	NaN	-0,3818554	0,1288213	-0,1246131	-0,984531	-0,4130767	NaN	-0,1213767	-0,1797434	0,04795711	NaN	NaN	7,90E-02	1,10	-0,25355224	
Programmed cell death protein 5	PDCD5	149730000	178000000	1,31	0,93	-0,10	11,127	-0,06190243	-0,1224285	-0,07807627	-0,130555	-0,06206814	-0,01633686	-0,1566914	-0,1066223	-0,2299745	-0,05920932	-0,1283771	-0,3003062	2,58E-04	3,59	-0,121045662	+
Glucose-6-phosphate isomerase	GPI	2035200000	2420100000	1,15	0,93	-0,10	10,667	-0,1629449	-0,2243004	-0,1259034	-0,08963569	-0,09048033	-0,1067931	-0,03637794	0,007626157	NaN	NaN	0,02261638	0,008630365	1,23E-02	1,91	-0,079756285	+
Signal peptidase complex subunit 2	SPCS2	57828000	80153000	1,36	0,93	-0,11	20,459	0,1853591	0,1120997	0,1195554	0,2503552	0,01049345	0,05783146	-0,485297	-0,5363357	-0,3465364	-0,3673781	-0,4349878	-0,3616018	1,06E-01	0,97	-0,149703538	
Transmembrane protein 33	TMEM33	80844000	106110000	1,35	0,93	-0,11	15,151	0,1692837	-0,1231982	0,007769512	0,1244606	-0,01259181	0,02842765	-0,3042734	-0,3133516	-0,2103626	-0,1690794	-0,2283172	-0,1683011	5,44E-02	1,26	-0,099961156	
Ribosyldihydronicotinamide dehydrogenase [quinone]	NQO2	84856000	103830000	1,24	0,93	-0,11	5,1267	-0,1719371	-0,1504491	0,1078218	-0,1761528	-0,04097178	-0,1049922	0,1096949	-0,0810338	NaN	-0,09850552	0,08909126	NaN	1,85E-01	0,73	-0,051743438	
Ubiquitin fusion degradation protein 1 homolog	UFD1L	42411000	49109000	1,14	0,93	-0,11	13,921	-0,188266	-0,01382941	0,09477648	0,005184378	-0,1848017	-0,04455326	NaN	NaN	-0,3930651	-0,1898449	NaN	NaN	7,45E-02	1,13	-0,114299935	
Dipeptidyl peptidase 3	DPP3	198100000	228120000	1,17	0,93	-0,11	6,7189	0,002306507	-0,1057992	-0,07298345	-0,02075023	-0,04990488	-0,04357155	-0,06285152	0,09680152	NaN	-0,005854803	0,05269407	0,08664794	5,86E-01	0,23	-0,011205964	
EH domain-containing protein 1	EHD1	90356000	118350000	1,19	0,93	-0,11	12,851	0,02630426	-0,1287557	-0,04896435	0,04684024	-0,2973424	-0,173238	NaN	NaN	NaN	NaN	0,6676651	-0,2658476	8,47E-01	0,07	-0,021667294	
Mitochondrial import inner membrane translocase subunit TIM16	PAM16	17651000	22074000	1,16	0,93	-0,11	1,7773	-0,01429554	-0,1134733	-0,04262027	NaN	-0,08946688	NaN	NaN	NaN	NaN	-0,3374846	0,6635723	NaN	9,40E-01	0,03	0,011038623	
Exportin-T	XPOT	91453000	104200000	1,19	0,93	-0,11	15,586	-0,3027778	-0,1272894	-0,05394294	-0,2998267	0,01206797	0,08936255	-0,2880295	-0,3544089	-0,3914557	-0,2371325	-0,184359	-0,2105796	8,48E-04	3,07	-0,195697628	+
Phosphoglycerate mutase 1	PGAM1	939380000	1166200000	1,19	0,93	-0,11	20,721	-0,3525473	-0,3248345	-0,2749133	-0,1853265	-0,3345345	-0,249222	0,0247452	0,07189995	0,05366741	0,04292445	0,1150332	0,07382025	6,56E-02	1,18	-0,111607301	
26S proteasome non-ATPase regulatory subunit 5	PSMD5	475280000	545640000	1,19	0,93	-0,11	8,2418	-0,001039149	-0,03621516	-0,02293263	-0,02359233	0,01506926	-0,07661502	NaN	-0,4930122	NaN	NaN	-0,36952	-0,2635076	5,26E-02	1,28	-0,141262766	
Vesicular integral-membrane protein VIP36	LMAN2	44698000	60831000	1,21	0,93	-0,11	11,36	-0,1033949	0,1634987	-0,1093276	-0,02577904	-0,07380319	-0,1501127	NaN	-0,08140004	-0,04465748	-0,01905342	NaN	NaN	1,39E-01	0,86	-0,049336628	
S-phase kinase-associated protein 1	SKP1	69391000	93111000	1,31	0,93	-0,11	9,3328	-0,03193135	0,02587931	-0,008479019	-0,2311932	-0,08408909	-0,119965	-0,2254301	-0,1776369	-0,08387498	0,007051912	-0,274826	-0,06215845	3,98E-03	2,40	-0,105554397	+
Serine-threonine kinase receptor-associated protein	STRAP	384060000	462130000	1,22	0,93	-0,11	9,522	-0,1708162	-0,2647204	-0,02127715	-0,07786307	-0,2851962	-0,2772542	-0,1380446	-0,09389441	-0,09595915	-0,07978291	NaN	NaN	7,22E-04	3,14	-0,150480824	+
Thioredoxin domain-containing protein 12	TXNDC12	31437000	37388000	1,26	0,93	-0,11	6,6314	NaN	-0,121722	NaN	-0,1290553	0,0354831	-0,1394741	NaN	-0,2653445	-0,04987505	-0,1641406	NaN	-0,5171311	2,33E-02	1,63	-0,168907458	+
Gamma-glutamylcyclotransferase	GGCT	63049000	52255000	1,36	0,93	-0,11	7,1311	-0,1187112	-0,1727501	-0,09091053	-0,1409687	-0,1029145	-0,08159849	0,01249718	0,1159654	0,0633648	0,1424789	0,06887715	0,1321162	6,75E-01	0,17	-0,014379493	
Peptidyl-prolyl cis-trans isomerase B	PPIB	403270000	525980000	1,11	0,93	-0,11	14,696	-0,2203621	-0,2809831	-0,3052	-0,2436769	-0,424223	-0,3870908	0,1692837	0,2287264	0,108491	0,1636276	0,2172308	0,1264442	3,63E-01	0,44	-0,070644367	
Glyceraldehyde-3-phosphate dehydrogenase	GAPDH	4912000000	6145600000	1,13	0,92	-0,11	7,8205	-0,2068449	-0,1659684	-0,1234496	-0,2459335	-0,2131862	-0,2028708	-0,07749762	-0,04651869	-0,07555045	-0,05737668	-0,07754336	-0,02422312	1,36E-04	3,87	-0,126413608	+
tRNA-splicing ligase RtcB homolog	RTCB	121360000	171460000	1,17	0,92	-0,11	13,909	-0,2717051	-0,08107954	-0,1177248	0,2225573	-0,4182149	-0,4139991	0,02856912	-0,139951	NaN	NaN	0,1473066	0,1243282	2,32E-01	0,64	-0,091991314	
Actin, alpha cardiac muscle 1;Actin, gamma-enteric smooth muscle;Actin, alpha skeletal muscle;Actin, aortic smooth muscle	ACTC1;ACTG2;ACTA1;ACTA2	668590000	859950000	1,14	0,92	-0,11	8,045	-0,1484009	-0,09946353	0,05741553	-0,06790855	-0,1454138	-0,1202002	-0,05707642	-0,04489555	-0,01941891	0,01934605	NaN	0,07655907	5,79E-02	1,24	-0,049950648	
Serine/threonine-protein phosphatase 6 catalytic subunit;Serine/threonine-protein phosphatase 6 catalytic subunit, N-terminally processed	PPP6C	30284000	36981000	1,25	0,92	-0,12	10,752	0,08011194	NaN	-0,1540885	-0,1655962	-0,07949331	-0,04595264	NaN	NaN	-0,1175526	-0,3677876	-0,2918216	-0,4211482	1,18E-02	1,93	-0,173703195	+
Cullin-1	CUL1	57822000	64984000	1,15	0,92	-0,12	10,305	-0,0284546	-0,06158624	NaN	-0,09220171	-0,04897921	-0,07979818	NaN	NaN	NaN	-0,1246131	-0,3246176	-0,1776859	1,06E-02	1,98	-0,117242073	+
Protein arginine N-methyltransferase 1	PRMT1	652420000	711210000	1,20	0,92	-0,12	12,527	-0,09118704	-0,05199724	-0,06259537	-0,1374573	-0,05289497	-0,1705564	0,1110314	-0,1827533	-0,1381399	-0,08144581	0,2072682	0,2148708	3,83E-01	0,42	-0,036321421	
Serrate RNA effector molecule homolog	SRRT	66152000	67004000	1,05	0,92	-0,12	10,521	-0,2784261	-0,2341428	-0,238085	-0,1714334	-0,6818625	-0,365481	0,1001703	NaN	NaN	NaN	0,3411888	-0,005536153	1,18E-01	0,93	-0,170400877	
Serine/arginine-rich splicing factor 2	SRSF2	237570000	319250000	1,52	0,92	-0,12	10,174	-0,4898685	-0,4221144	-0,1163635	-0,2697731	-0,4673938	-0,6403215	0,02162186	0,04670055	-0,03528351	-0,09892265	0,00302647	-0,04800941	1,09E-02	1,96	-0,209725115	+
U2 small nuclear ribonucleoprotein A	SNRPA1	98497000	118000000	1,17	0,92	-0,12	16,935	-0,1051939	-0,3002352	-0,06905833	-0,1477454	-0,2385104	-0,2044159	0,03139513	-0,1937832	-0,006376396	-0,1936512	-0,5796201	-0,2476796	1,74E-03	2,76	-0,187906198	+
Fatty acid-binding protein, epidermal	FABP5	77912000	91882000	1,19	0,92	-0,12	7,7299	-0,1382828	0,009203812	-0,4219404	-0,2647725	-0,1523879	-0,1377907	-0,4148836	-0,08851548	-0,1689497	-0,1194477	-0,2904623	-0,07996587	4,53E-04	3,34	-0,189016265	+
Heterogeneous nuclear ribonucleoprotein K	HNRNPK	2720500000	3336800000	1,12	0,92	-0,12	26,082	-0,1889565	-0,2208327	-0,1138791	-0,1206392	-0,3346618	-0,3153607	0,08215749	-0,05623617	0,3115032	0,3181144	0,08528864	0,2290956	6,92E-01	0,16	-0,027033919	
GTP-binding nuclear protein Ran	RAN	2451200000	3042100000	1,17	0,92	-0,12	6,8693	-0,1714984	-0,1744749	-0,1275573	-0,1195888	-0,2175075	-0,1614112	-0,110324	-0,06292688	-0,07580891	-0,1100281	-0,004363514	0,05018827	4,59E-04	3,34	-0,107108437	+
Elongation factor 1-gamma	EEF1G	3217000000	3810400000	1,23	0,92	-0,12	10,107	0,0426443	0,07327176	-0,08133899	-0,1170674	-0,1483209	-0,1111185	-0,2316674	-0,3354443	-0,3147147	-0,3631967	-0,3879401	-0,2520884	1,53E-03	2,82	-0,185581788	+
Nuclear pore complex protein Nup85	NUP85	13466000	19422000	1,13	0,92	-0,12	12,843	-0,06666908	-0,006318355	NaN	NaN	NaN	-0,2235085	NaN	NaN	NaN	NaN	0,1982427	0,1919416	8,27E-01	0,08	0,018737664	
N-alpha-acetyltransferase 10	NAA10	71175000	80182000	1,20	0,92	-0,12	4,6753	-0,07002721	-0,1492005	-0,1809368	-0,1741004	-0,04290257	-0,1440741	-0,2903388	-0,0764781	-0,07093623	-0,1265175	-0,2938895	0,07080147	1,13E-03	2,95	-0,129050022	+
Cell differentiation protein RCD1 homolog	RQCD1	22515000	32616000	1,29	0,92	-0,12	14,482	0,1331689	0,100574	0,06571014	-0,09720859	-0,2232727	0,1966071	-0,02416445	-0,03605248	NaN	-0,1104953	0,1112984	-0,08767208	9,48E-01	0,02	0,00259026	
Coatomer subunit alpha;Xenin;Proxenin	COPA	576600000	590480000	1,02	0,92	-0,13	18,66	-0,2074278	-0,1904209	-0,1392358	-0,08295787	-0,1315502	-0,06441956	-0,08534361	-0,1034879	0,1556197	0,1604039	0,000288558	0,01649632	1,33E-01	0,88	-0,056002946	
RNA-binding protein 8A	RBM8A	60736000	79019000	1,41	0,92	-0,13	12,057	-0,07060288	0,02573757	0,01749438	0,104605	-0,3605456	-0,2224645	-0,1370607	-0,08762603	0,04837578	-0,1226955	0,03097168	-0,138521	7,10E-02	1,15	-0,076027644	
28S ribosomal protein S6, mitochondrial	MRPS6	3774700	5531800	1,08	0,92	-0,13	19,281	-0,3155223	-0,2115816	NaN	NaN	NaN	NaN	NaN	0,2092662	NaN	NaN	0,1051417	NaN	6,99E-01	0,16	-0,053173985	
39S ribosomal protein L28, mitochondrial	MRPL28	8833700	11731000	1,34	0,92	-0,13	15,445	NaN	NaN	NaN	-0,2266618	-0,1670046	-0,1516345	NaN	NaN	0,03984026	0,04544289	-0,02354836	-0,0234017	1,27E-01	0,90	-0,072423982	
Dynein light chain Tctex-type 1	DYNLT1	11380000	15609000	1,33	0,92	-0,13	12,406	-0,1745563	0,0542234	NaN	0,05213766	-0,08626233	0,1003049	-0,2344992	-0,2251434	NaN	NaN	-0,2906035	-0,3392539	4,79E-02	1,32	-0,127072514	
Guanine nucleotide-binding protein G(s) subunit alpha isoforms short;Guanine nucleotide-binding protein G(s) subunit alpha isoforms XLas	GNAS	29571000	27032000	1,05	0,91	-0,13	10,122	NaN	-0,2424988	NaN	-0,1873129	NaN	-0,1160195	NaN	NaN	-0,9869023	-1,540317	NaN	NaN	9,30E-02	1,03	-0,614610007	
Heterogeneous nuclear ribonucleoprotein M	HNRNPM	1001100000	871010000	1,26	0,91	-0,13	28,795	-1,017943	-0,9809956	-0,233142	-0,2210177	-1,078228	-0,905872	0,01948842	0,0880058	0,0353423	0,09976643	0,152508	0,05130255	4,34E-02	1,36	-0,332565426	
Dihydrofolate reductase	DHFR	73558000	93443000	1,36	0,91	-0,13	15,734	-0,1982122	-0,1741655	-0,3507984	-0,4530833	-0,2884875	-0,2613446	0,01392662	0,1463945	0,07942928	0,0880058	-0,003929475	-0,106265	4,47E-02	1,35	-0,125710819	
Developmentally-regulated GTP-binding protein 1	DRG1	175370000	215390000	1,50	0,91	-0,13	19,744	-0,4738711	-0,3902829	-0,3488863	-0,3461878	-0,5961433	-0,4706488	0,1944653	0,06722574	0,1792563	0,09801521	NaN	NaN	5,95E-02	1,23	-0,208705766	
Dynein light chain roadblock-type 1;Dynein light chain roadblock-type 2	DYNLRB1;DYNLRB2	13396000	15984000	1,25	0,91	-0,13	3,025	-0,1667618	-0,06366543	NaN	NaN	-0,08629292	NaN	-0,2449415	-0,000750383	NaN	-0,07366654	NaN	-0,1195574	1,12E-02	1,95	-0,10794799	+
Voltage-dependent anion-selective channel protein 3	VDAC3	550900000	669370000	1,22	0,91	-0,13	20,686	0,04656085	0,03463829	-0,1240627	-0,2305159	-0,1256988	-0,1890552	-0,1437552	-0,0369106	0,1119661	0,1304037	0,04725923	0,105678	4,13E-01	0,38	-0,031124352	
N-alpha-acetyltransferase 15, NatA auxiliary subunit	NAA15	227590000	236260000	1,08	0,91	-0,13	16,423	-0,1923813	-0,1349205	-0,1125995	-0,01921428	-0,1414779	-0,2176753	-0,4683915	0,1315895	-0,08532838	0,01478381	NaN	-0,04201112	3,20E-02	1,50	-0,115238773	
Thioredoxin domain-containing protein 17	TXNDC17	63239000	71768000	1,13	0,91	-0,13	3,0354	-0,1153943	-0,1527887	-0,1017529	-0,07731498	-0,169907	-0,05725652	NaN	0,04935203	-1,412846	-0,01886339	NaN	NaN	1,65E-01	0,78	-0,228530236	
Eukaryotic translation initiation factor 2 subunit 2	EIF2S2	100070000	142370000	1,53	0,91	-0,13	13,239	NaN	-0,1556304	-0,3874494	NaN	NaN	-0,3736076	NaN	-0,1535588	-0,1941628	0,1606621	-0,2101456	-0,275245	1,35E-02	1,87	-0,198642183	+
Growth factor receptor-bound protein 2	GRB2	92139000	113370000	1,27	0,91	-0,13	12,831	-0,1414779	-0,1223186	-0,1535268	-0,1102617	-0,1594441	NaN	-0,09790324	NaN	0,0321009	-0,0259994	-0,1958473	-0,1115704	6,13E-04	3,21	-0,108624846	+
Ran-specific GTPase-activating protein	RANBP1	865170000	1064500000	1,21	0,91	-0,13	6,4752	-0,1027596	-0,1384575	-0,2023893	-0,1368862	-0,1766746	-0,1820984	-0,001356807	-0,08271334	-0,1233083	-0,08314123	-0,06756084	-0,05620613	3,54E-05	4,45	-0,112796019	+
Insulin-degrading enzyme	IDE	54438000	62053000	1,11	0,91	-0,14	15,505	-0,1572223	-0,1138479	-0,1401735	-0,2579248	0,000721115	-0,1649814	0,2096405	0,06087747	0,2441563	-0,1437075	0,05018827	NaN	4,64E-01	0,33	-0,037479425	
Thioredoxin-related transmembrane protein 1	TMX1	21401000	32756000	1,26	0,91	-0,14	2,6627	0,1473066	-0,02500092	NaN	-0,1924143	-0,01921428	-0,1101527	-0,1124123	-0,1073836	-0,1987254	0,08188502	NaN	NaN	1,66E-01	0,78	-0,059567984	
Nuclear pore complex protein Nup93	NUP93	148460000	156960000	1,14	0,91	-0,14	16,871	-0,1215336	-0,1993713	0,1025909	-0,02594065	-0,4193528	-0,3373934	-0,4501238	-0,1089853	-0,02501553	0,06018582	0,005902907	-0,1535268	2,25E-02	1,65	-0,139380293	+
Nucleoside diphosphate kinase B;Putative nucleoside diphosphate kinase	NME2;NME2P1	3084200000	3937100000	1,24	0,91	-0,14	14,535	-0,09705434	-0,0759609	-0,09691547	-0,06134539	-0,09858277	-0,06612524	-0,166697	-0,2075611	-0,1930738	-0,1216278	-0,1041701	-0,1256043	3,53E-06	5,45	-0,117893179	+
Ubiquitin-40S ribosomal protein S27a;Ubiquitin;40S ribosomal protein S27a;Ubiquitin-60S ribosomal protein L40;Ubiquitin;60S ribosomal protein L40;Polyubiquitin-B;Ubiquitin;Polyubiquitin-C;Ubiquitin	RPS27A;UBA52;UBB;UBC	1843900000	2208400000	1,29	0,91	-0,14	20,046	-0,1792206	-0,2255988	-0,4524909	-0,2476111	-0,2237949	-0,3278191	-0,08653805	0,05144163	-0,08016421	-0,08584885	-0,08138476	-0,07249789	1,36E-03	2,87	-0,167627291	+
Stromal cell-derived factor 2-like protein 1	SDF2L1	58683000	73024000	1,33	0,91	-0,14	16,888	-0,3042556	-0,4022638	-0,1994376	-0,2192532	-0,2019578	-0,2940309	-0,02749851	-0,04720429	-0,09035747	0,175301	-0,01109355	0,06543438	2,22E-02	1,65	-0,129718111	+

Acidic leucine-rich nuclear phosphoprotein 32 family member E	ANP32E	52369000	68560000	1,15	0,90	-0,14	6,71	-0,2091779	-0,05008417	-0,001876691	-0,1607499	-0,08811671	-0,002469117	0,2906603	0,6016966	-0,2199252	-0,3493457	NaN	-0,2074611	6,66E-01	0,18	-0,036077242	
Small nuclear ribonucleoprotein F	SNRPF	26973000	36474000	1,57	0,90	-0,15	7,1809	-0,547763	-0,353782	NaN	NaN	-0,4054323	-0,4067323	-0,07909693	0,05283319	-0,1088452	-0,06743983	-0,0871356	-0,2217747	5,67E-03	2,25	-0,222516878	+
RNA-binding protein 14	RBM14	50054000	44880000	1,49	0,90	-0,15	24,849	-0,4370762	NaN	-0,311954	-0,142624	-0,5903321	NaN	NaN	0,07038926	-0,2328199	0,07970238	0,4691566	0,1309309	3,55E-01	0,45	-0,107180784	
Protein MEMO1	MEMO1	19259000	22118000	1,10	0,90	-0,15	14,617	-0,2113811	-0,1284402	-0,1832283	-0,1835068	-0,4148451	-0,1223186	NaN	NaN	-0,180152	0,2617113	NaN	NaN	6,52E-02	1,19	-0,145270097	
Isochorismatase domain-containing protein 1	ISOC1	71770000	89392000	1,08	0,90	-0,15	14,675	-0,2869553	0,02119536	-0,1625411	-0,2876948	-0,1332106	-0,2410829	0,108491	-0,05761687	0,1183597	0,05699965	-0,004378005	0,1758121	2,45E-01	0,61	-0,05771848	
60S ribosomal protein L37a	RPL37A	85324000	92939000	1,51	0,90	-0,15	4,3847	-0,9749429	-0,9112343	-0,706865	-0,6609082	-0,9284021	-0,8439658	-0,2024723	-0,0929555	-0,1805444	-0,1556947	-0,1090631	-0,1346829	7,97E-04	3,10	-0,491810932	+
ELAV-like protein 1	ELAVL1	153390000	177970000	1,55	0,90	-0,15	25,996	-0,7494507	-0,7866465	-0,2617596	-0,1376161	-0,6436308	-0,7703383	0,1304037	0,1751732	0,05602856	-0,1668265	0,1278974	-0,1038599	3,55E-02	1,45	-0,260885465	
Transcription intermediary factor 1-beta	TRIM28	1467300000	1575600000	0,99	0,90	-0,15	8,4724	-0,2059627	-0,1466909	-0,2338034	-0,1826223	-0,1784694	-0,1306971	-0,07669102	-0,1206707	0,07642223	0,03449741	0,07436837	0,06364096	2,76E-02	1,56	-0,085556549	+
Pre-mRNA-splicing factor SPF27	BCAS2	38805000	48032000	1,44	0,90	-0,15	13,684	-0,4406942	NaN	0,01306909	-0,6690975	-0,4572367	-0,8715747	-0,1011184	-0,1947901	-0,1226798	-0,09903087	-0,07462331	-0,1359664	7,31E-03	2,14	-0,286703903	+
Trafficking protein particle complex subunit 3	TRAPPC3	36640000	43192000	1,03	0,90	-0,15	23,804	NaN	-0,1099659	-0,07857888	0,06032421	0,02431962	0,001009529	NaN	-0,358232	-0,5175027	-0,7536771	NaN	NaN	7,70E-02	1,11	-0,216537899	
Serine/threonine-protein phosphatase PP1-alpha catalytic subunit	PPP1CA	263790000	344240000	1,20	0,90	-0,15	17,106	-0,1757779	-0,197633	-0,03187236	0,03097168	-0,1525002	-0,177229	-0,1248176	-0,1704426	-0,2148596	-0,1972691	-0,1576728	-0,1869515	2,54E-05	4,59	-0,146337837	+
Nuclear pore complex protein Nup160	NUP160	72596000	83697000	1,00	0,90	-0,15	7,5962	-0,3625288	-0,2764327	0,07313477	0,08569661	-0,5686873	-0,3758336	-0,1210785	-0,2060126	-0,1542491	-0,011588	-0,1531738	-0,06264055	8,87E-03	2,05	-0,177782795	+
WD40 repeat-containing protein SMU1;WD40 repeat-containing protein SMU1, N-terminally processed	SMU1	14124000	15550000	1,42	0,90	-0,16	9,4902	NaN	NaN	NaN	-0,1243614	NaN	-0,424223	NaN	-0,05547139	NaN	NaN	-0,04205566	-0,2066617	7,10E-02	1,15	-0,170554648	
Serine/arginine-rich splicing factor 6	SRSF6	80929000	116600000	0,97	0,90	-0,16	27,101	-0,4306062	-0,4232748	-0,4056235	-0,1225541	-1,189878	NaN	0,1323794	0,6125885	NaN	NaN	0,07066408	0,1360601	3,21E-01	0,49	-0,180027141	
Prostaglandin E synthase 3	PTGES3	430370000	567080000	1,52	0,90	-0,16	4,6405	0,3139424	-0,001472321	0,2401311	-0,2089611	-0,1517306	-0,08036248	-0,1820166	-0,1635911	-0,1689822	-0,1147848	-0,1711897	-0,1986592	1,69E-01	0,77	-0,073973054	
Peptidyl-prolyl cis-trans isomerase FKBP8	FKBP8	60503000	65841000	1,21	0,90	-0,16	15,273	-0,05568122	-0,2422598	-0,1388863	-0,3638836	-0,04270937	-0,3391991	NaN	NaN	NaN	NaN	-0,2397194	-0,1417484	2,55E-03	2,59	-0,195510905	+
Exosome complex component RRP42	EXOSC7	8087400	10581000	1,08	0,89	-0,16	7,4059	NaN	-0,1601694	0,167101	NaN	NaN	-0,2702602	NaN	-0,0762499	-0,2557528	-0,1893513	NaN	NaN	1,05E-01	0,98	-0,130780434	
Serine/threonine-protein kinase PAK 2;PAK-2p27;PAK-2p34	PAK2	248220000	271070000	1,22	0,89	-0,16	14,374	-0,3524553	-0,1629771	-0,2924222	-0,3078406	-0,2767124	-0,1001129	NaN	NaN	NaN	NaN	-0,1055819	-0,04324446	1,55E-03	2,81	-0,205168362	+
Heat shock protein 105 kDa	HSPH1	603790000	656810000	1,19	0,89	-0,16	11,047	-0,1111341	-0,07052711	-0,2387147	-0,1688848	-0,1223814	-0,1358397	-0,1106822	-0,1931398	-0,1462598	-0,1871158	-0,1256516	-0,138521	2,34E-07	6,63	-0,145737649	+
GMP synthase [glutamine-hydrolyzing]	GMPS	677730000	749210000	1,09	0,89	-0,16	27,565	-0,1968225	-0,1978978	-0,1334639	-0,1488966	-0,1035499	-0,1289765	-0,2526039	-0,3104504	-0,3209186	-0,3453995	-0,269947	-0,2939779	1,60E-06	5,80	-0,225242051	+
Vacuolar protein sorting-associated protein 26A	VPS26A	56528000	71650000	1,27	0,89	-0,16	9,2761	NaN	0,01920368	NaN	-0,06816573	-0,5583252	NaN	NaN	NaN	-0,1181162	-0,1578176	NaN	NaN	1,52E-01	0,82	-0,176644212	
Mitochondrial import receptor subunit TOM22 homolog	TOMM22	127160000	148840000	1,31	0,89	-0,16	4,565	-0,1147537	-0,1851461	-0,3546304	-0,2026715	-0,2900213	-0,1847853	0,003746074	-0,1866887	-0,05150381	0,08392822	0,07724293	-0,1459245	7,21E-03	2,14	-0,129267343	+
Quinone oxidoreductase	CRYZ	58943000	73944000	1,11	0,89	-0,17	26,527	-0,1746539	-0,2339391	-0,086247	-0,3207023	-0,1308393	-0,0229619	-0,3017638	NaN	-0,1147848	-0,1195888	-0,1808714	-0,000346329	5,83E-04	3,23	-0,153336248	+
Inorganic pyrophosphatase	PPA1	611010000	729920000	1,14	0,89	-0,17	12,02	-0,126738	-0,2185647	-0,1313448	-0,1548272	-0,1212355	-0,09944805	-0,1865738	-0,1714009	-0,1176622	-0,208011	-0,1971533	-0,228013	5,75E-08	7,24	-0,163414375	+
Thioredoxin domain-containing protein 5	TXNDC5	68321000	85451000	1,15	0,89	-0,17	5,5537	-0,1873621	-0,03051607	-0,3567349	-0,2677742	-0,1906185	NaN	NaN	NaN	NaN	NaN	-0,1384099	-0,06630655	5,97E-03	2,22	-0,176817454	+
Lanosterol 14-alpha demethylase	CYP51A1	81037000	94677000	1,23	0,89	-0,17	13,113	-0,2545475	-0,3787757	-0,188973	-0,07110289	-0,3962514	-0,1285506	NaN	NaN	NaN	NaN	-0,0407937	-0,09754815	5,03E-03	2,30	-0,194567866	+
N-alpha-acetyltransferase 50	NAA50	90238000	103380000	1,16	0,89	-0,17	11,355	-0,2163003	-0,1760713	-0,4268582	-0,2482107	-0,2752799	-0,2450441	-0,1350473	-0,1666808	-0,1462598	0,02913487	NaN	-0,08228566	2,99E-04	3,52	-0,189900286	+
Staphylococcal nuclease domain-containing protein 1	SND1	1122100000	1156300000	0,97	0,89	-0,17	24,395	-0,3269502	-0,2569935	-0,1987254	-0,1674421	-0,3445566	-0,2358917	0,2711866	0,3680419	0,4216945	0,3963788	0,4557546	0,3716146	5,33E-01	0,27	0,062842626	
ADP/ATP translocase 2;ADP/ATP translocase 2, N-terminally processed	SLC25A5	1614500000	1858400000	1,14	0,89	-0,17	7,3391	-0,3148582	-0,325684	-0,2006971	-0,135491	-0,3174087	-0,3030803	-0,1370131	-0,1440581	-0,1379493	-0,08228566	-0,1572866	-0,1028991	1,38E-05	4,86	-0,196559273	+
28 kDa heat- and acid-stable phosphoprotein	PDAP1	21965000	21145000	1,12	0,89	-0,17	10,022	-0,1926451	-0,1654667	-0,0716638	-0,07897503	-0,1197142	-0,2681217	-0,2382552	NaN	NaN	NaN	0,02147971	NaN	4,54E-03	2,34	-0,139170247	+
Small nuclear ribonucleoprotein Sm D2	SNRPD2	225690000	284320000	1,12	0,89	-0,17	18,85	-0,3192796	-0,2841944	-0,2358067	-0,2162333	-0,4267612	-0,3706387	-0,2172728	-0,1560162	-0,1375367	-0,1714984	-0,1315345	-0,1461161	4,28E-06	5,37	-0,234407379	+
Vacuolar protein sorting-associated protein 29	VPS29	105900000	130260000	1,27	0,89	-0,17	9,8146	-0,1969879	-0,1808223	-0,2002329	-0,09733213	-0,1287557	-0,05364339	-0,1496807	-0,1021245	-0,3398929	-0,138394	-0,1020626	-0,1960621	1,46E-05	4,83	-0,157165929	+
Cilia- and flagella-associated protein 20	CFAP20	38326000	45379000	1,16	0,89	-0,17	11,585	-0,2293993	-0,1739539	-0,1767724	-0,20558	-0,3303564	-0,3283806	NaN	NaN	0,06722574	-0,04351208	NaN	-0,09937076	4,35E-03	2,36	-0,168899962	+
Galectin-1	LGALS1	102640000	112520000	1,11	0,89	-0,18	4,6978	-0,1798251	-0,07092106	-0,1353958	-0,06984551	-0,08695164	-0,01151527	-1,03115	-0,5168833	-0,5595367	-0,3601381	-0,5995496	-0,2750006	3,44E-03	2,46	-0,32472605	+
Guanine nucleotide-binding protein G(I)/G(S)/G(T) subunit beta-2	GNB2	28952000	32528000	1,02	0,89	-0,18	7,4944	-0,159557	-0,1761365	-0,3793948	-0,2629882	-0,01728567	-0,01646819	NaN	NaN	-0,4789895	-0,4087611	NaN	NaN	6,49E-03	2,19	-0,237447607	+
Nardilysin	NRD1	169320000	178620000	1,05	0,88	-0,18	13,456	-0,2183465	-0,1605079	-0,1078655	-0,1076479	-0,2263243	-0,2018748	-0,2633691	-0,02038432	NaN	-0,3106114	-0,4330778	-0,2243847	1,02E-04	3,99	-0,206763113	+
Matrin-3	MATR3	495730000	547820000	1,02	0,88	-0,18	12,984	-0,4867317	-0,2510922	-0,010454	0,1486088	-0,6285192	-0,5851068	0,0167816	0,02672926	0,07874646	0,1158324	-0,1415097	-0,1717909	7,31E-02	1,14	-0,1573755	
Dipeptidyl peptidase 1;Dipeptidyl peptidase 1 exclusion domain chain;Dipeptidyl peptidase 1 heavy chain;Dipeptidyl peptidase 1 light chain	CTSC	34377000	35501000	1,13	0,88	-0,18	10,255	-0,2655354	-0,2361975	-0,5630707	-0,4753346	-0,2962968	NaN	-0,07500324	NaN	NaN	NaN	NaN	-0,1995204	3,01E-03	2,52	-0,301565516	+
L-aminoadipate-semialdehyde dehydrogenase-phosphopantetheinyl transferase	AASDHPPT	20258000	22627000	1,01	0,88	-0,18	18,853	-0,2324469	-0,2582698	-0,0183957	-0,1207804	-0,0678935	NaN	-0,2736222	-0,4360414	-0,2539797	-0,2948979	NaN	-0,1214551	5,12E-04	3,29	-0,20777826	+
Polypyrimidine tract-binding protein 1	PTBP1	1905200000	2187500000	1,05	0,88	-0,19	17,289	-0,3241298	-0,3411533	-0,189631	-0,2024557	-0,3256297	-0,3505224	0,2128806	0,229834	0,26123	0,2452522	0,0451634	0,0439044	4,49E-01	0,35	-0,057938109	
Importin-5	IPO5	598820000	604840000	1,02	0,88	-0,19	7,9786	-0,1585098	-0,08352332	-0,2627632	-0,2002827	-0,2010287	-0,2202108	-0,3437508	-0,2249747	-0,1494086	-0,2120327	-0,3065019	-0,4483708	4,07E-06	5,39	-0,234279845	+
60 kDa SS-A/Ro ribonucleoprotein	TROVE2	231220000	254460000	1,09	0,88	-0,19	8,918	-0,1598469	-0,254427	-0,1680255	-0,1879372	-0,01017798	-0,1828188	NaN	NaN	NaN	NaN	-0,2054302	-0,3702471	9,90E-04	3,00	-0,192363843	+
Mitochondrial-processing peptidase subunit alpha	PMPCA	111720000	134850000	1,11	0,88	-0,19	13,219	-0,04080856	0,03759303	-0,01711041	-0,04689121	-0,05279016	-0,240401	NaN	NaN	NaN	NaN	-0,2119157	-0,1076479	4,11E-02	1,39	-0,084996476	
UPF0568 protein C14orf166	C14orf166	100230000	119970000	1,08	0,88	-0,19	11,359	-0,3839623	-0,4221917	-0,1979475	-0,1012886	-0,4472098	-0,3949798	0,05949366	0,0207689	0,06571014	-0,007362068	0,01649632	0,08283886	4,15E-02	1,38	-0,142469483	
Interleukin enhancer-binding factor 2	ILF2	1070200000	1154100000	1,02	0,88	-0,19	12,782	-0,4301395	-0,3561623	-0,1802501	-0,2067949	-0,4785673	-0,5181018	-0,1124123	-0,011588	-0,1240627	-0,06751543	NaN	NaN	1,96E-03	2,71	-0,248559442	+
Cysteine and histidine-rich domain-containing protein 1	CHORDC1	96284000	99444000	1,10	0,88	-0,19	19,316	0,1206174	-0,2416627	-0,1633649	-0,0110354	-0,2097284	-0,1108069	-0,3760957	-0,1558393	-0,1602177	-0,5320528	-0,0255441	-0,2726632	4,21E-03	2,38	-0,178199482	+
Guanine nucleotide-binding protein G(I)/G(S)/G(T) subunit beta-1	GNB1	28302000	34863000	1,23	0,88	-0,19	16,631	0,5960764	0,03815525	NaN	-0,1973188	0,02332628	-0,1621052	-0,2023229	NaN	-0,1469305	-0,2318877	-0,6674696	-0,382758	2,27E-01	0,64	-0,133323483	
Protein arginine N-methyltransferase 5;Protein arginine N-methyltransferase 5, N-terminally processed	PRMT5	244150000	267560000	1,21	0,88	-0,19	9,2632	-0,3003062	-0,2584079	-0,1546183	-0,2674443	-0,3542246	-0,258943	0,004752986	0,06198353	0,05935536	-0,05075641	-0,05934462	0,03787417	1,50E-02	1,82	-0,128339939	+
40S ribosomal protein S23	RPS23	291000000	309030000	1,38	0,88	-0,19	10,233	-1,053045	-0,8457537	NaN	-0,5267639	NaN	-0,8675153	-0,0837833	-0,1761203	-0,1113054	0,2375637	0,4506966	0,1877678	1,23E-01	0,91	-0,278825825	
Prefoldin subunit 2	PFDN2	117920000	153710000	1,25	0,88	-0,19	9,8699	NaN	-0,1183197	-0,1979143	-0,1615887	-0,1948231	-0,1665027	-0,242789	-0,159428	-0,2167529	-0,1878715	-0,1618953	-0,253051	2,29E-08	7,64	-0,187357843	+
Protein TBRG4	TBRG4	253400000	281230000	1,05	0,88	-0,19	14,027	-0,1907008	-0,1940968	-0,2322435	-0,180103	-0,2507833	-0,1279514	NaN	NaN	NaN	NaN	0,07888308	-0,1257302	4,13E-03	2,38	-0,152840732	+
Small nuclear ribonucleoprotein Sm D1	SNRPD1	281630000	294720000	1,07	0,88	-0,19	2,945	-0,1998683	-0,2697035	-0,07862465	-0,04751734	-0,3675643	-0,2344483	-0,01987228	-0,1506892	0,09450638	-0,1091565	-0,2275062	-0,1222086	2,07E-03	2,68	-0,144387735	+
Succinate dehydrogenase [ubiquinone] iron-sulfur subunit, mitochondrial	SDHB	152890000	194460000	1,16	0,88	-0,19	9,2398	-0,1415575	-0,220278	-0,177327	-0,1162072	-0,0740006	-0,2308376	-0,09284783	-0,09893812	-0,08311071	-0,3199098	-0,02563224	-0,1018768	1,06E-04	3,97	-0,140210278	+
CLIP-associating protein 2	CLASP2	10243000	10575000	1,29	0,88	-0,19	19,933	-0,5074801	NaN	-0,2443091	NaN	NaN	0,01392662	NaN	NaN	-0,6070671	-0,518887	NaN	NaN	3,09E-02	1,51	-0,372763357	
AH receptor-interacting protein	AIP	12342000	13737000	1,06	0,88	-0,19	9,4566	NaN	-0,09963361	NaN	-0,2100454	-0,2972538	-0,2739536	NaN	NaN	NaN	NaN	0,0353423	0,1354035	1,58E-01	0,80	-0,118356776	
Pre-mRNA-processing-splicing factor 8	PRPF8	284540000	273840000	1,10	0,88	-0,19	15,969	-0,4452442	-0,2531713	-0,09582038	0,06805168	-0,6365261	-0,5235665	-0,125746	-0,2053138	-0,1574475	-0,1710435	-0,155518	-0,2209168	1,31E-03	2,88	-0,243521867	+
CTP synthase 1	CTPS1	149640000	146320000	1,08	0,88	-0,19	16,046	-0,4884307	-0,08288145	-0,314948	-0,2824387	-0,2257337	-0,1179126	NaN	NaN	-0,1671991	NaN	NaN	0,1932041	3,37E-02	1,47	-0,185792531	
Coatomer subunit epsilon	COPE	59991000	66034000	1,00	0,88	-0,19	10,961	-0,1894006	-0,3460228	-0,006216962	-0,150465	-0,2111807	-0,1522275	0,06956466	NaN	-0,2063621	-0,04008153	0,1176951	0,02219017	4,25E-02	1,37	-0,09931884	
39S ribosomal protein L11, mitochondrial	MRPL11	49502000	58482000	1,12	0,88	-0,19	28,932	-0,2120327	-0,2292639	-0,2247556	-0,3276199	-0,192711	-0,2634384	0,05464008	0,0889556	0,05491792	0,03759303	0,02162186	-0,02228782	4,20E-02	1,38	-0,101198398	
Signal recognition particle 19 kDa protein	SRP19	19340000	21260000	1,16	0,88	-0,19	13,197	-0,1897626	0,1281614	NaN	-0,2678263	NaN	NaN	-0,2580455	-0,2247893	-0,3041487	-0,3501179	NaN	-0,4220951	4,77E-03	2,32	-0,236078	+
60S ribosomal protein L38	RPL38	224790000	237940000	1,39	0,87	-0,19	22,261	-0,7241824	-0,7264721	-0,7571597	-0,8751161	-0,8291519	-0,9297206	-0,05286499	0,01035019	-0,09369426	-0,08117106	-0,02733672	-0,03464787	3,64E-03	2,44	-0,426763962	+
Inositol-3-phosphate synthase 1	ISYNA1	468250000	503030000	1,07	0,87	-0,19	10,216	-0,1427356	-0,251659	-0,07743674	-0,2044824	-0,1299392	-0,2415603	-0,4056807	-0,2034853	NaN	NaN	-0,07970667	-0,05457206	4,67E-04	3,33	-0,179125801	+
Heterogeneous nuclear ribonucleoprotein L	HNRNPL	492360000	544910000	1,06	0,87	-0,20	13,644	-0,5053694	-0,3980753	0,1488691	-0,1021555	-0,278636	-0,2980163	-0,003336449	-0,09651437	NaN	0,2027625	0,1050075	0,01364095	1,47E-01	0,83	-0,110165751	
Testis-expressed sequence 10 protein	TEX10	10106000	11892000	1,16	0,87	-0,20	16,863	-0,3013726	NaN	NaN	0,0441843	NaN	-0,5050827	-0,2018251	NaN	NaN	NaN	NaN	0,01449814	1,36E-01	0,87	-0,189919579	
ATP-dependent RNA helicase DDX1	DDX1	168550000	181430000	1,03	0,87	-0,20	14,512	-0,3131365	-0,2620538	-0,2921219	-0,05509656	-0,1592185	-0,2869729	-0,1198396	-0,05293984	-0,03380579	-0,07172444	-0,1497126	-0,07915797	3,19E-04	3,50	-0,156315027	+
Alkyldihydroxyacetonephosphate synthase, peroxisomal	AGPS	155690000	158550000	1,13	0,87	-0,20	12,572	-0,2932175	-0,3960996	-0,3524736	-0,4398525	-0,4457943	-0,4044582	-0,1932223	-0,01382941	-0,1110094	-0,1546826	-0,1325621	-0,3023685	4,73E-05	4,33	-0,269964174	+
Eukaryotic translation initiation factor 4H	EIF4H	246780000	254200000	1,16	0,87	-0,20	15,595	-0,4895038	-0,4346173	-0,2348218	-0,2671317	-0,2973602	-0,3688118	-0,3723932	-0,214123	-0,1038445	-0,03110562	-0,1098568	-0,07646286	1,16E-04	3,94	-0,25000272	+
dCTP pyrophosphatase 1	DCTPP1	75902000	85294000	1,16	0,87	-0,20	8,3055	-0,2462243	-0,3320625	-0,1505131	-0,2106296	-0,2070614	-0,3047366	0,1834547	0,8281441	1,428625	-0,05163838	0,8004962	-0,08866891	4,20E-01	0,38	0,137432087	
39S ribosomal protein L40, mitochondrial	MRPL40	10757000	17928000	1,16	0,87	-0,20	14,305	-0,09084898	-0,09280171	NaN	0,2746493	-0,0258672	-0,08323296	0,2901885	0,3091761	0,1627257	NaN	NaN	NaN	1,96E-01	0,71	0,092998597	
Elongation factor 1-beta	EEF1B2	342560000	431360000	1,24	0,87	-0,20	8,9917	-0,007608591	-0,09901539	-0,1575923	-0,0597054	-0,1172396	-0,1081764	-0,4208584	-0,3163305	-0,1554215	-0,222313	-0,2983356	-0,295854	2,49E-04	3,60	-0,188204241	+
28S ribosomal protein S27, mitochondrial	MRPS27	48383000	56217000	1,40	0,87	-0,20	16,299	-0,489828	-0,2586323	NaN	NaN	0,0247452	-0,231244	-0,2519682	NaN	-0,1284244	-0,2504228	NaN	NaN	8,32E-03	2,08	-0,226539213	+
RNA-binding protein 39	RBM39	94788000	103150000	1,09	0,87	-0,20	25,63	-0,1884139	-0,3251959	-0,1946745	-0,3396555	-0,5587716	-0,2305159	-0,2159149	-0,02597007	-0,04376486	-0,04942711	0,01464098	-0,2182626	1,41E-03	2,85	-0,197993823	+
Eukaryotic translation initiation factor 5	EIF5	80854000	100290000	1,11	0,87	-0,20	14,038	-0,2143072	-0,09038813	-0,2490334	-0,2024889	-0,3226496	-0,3216756	NaN	NaN	NaN	NaN	0,5624744	0,3523065	6,22E-01	0,21	-0,060720233	
Heterogeneous nuclear ribonucleoproteins A2/B1	HNRNPA2B1	762190000	1083000000	0,91	0,87	-0,20	19,465	-0,5994183	-0,5580916	-0,1603306	-0,1259192	-0,7856763	-0,6489597	0,138028	0,1182268	0,05380643	0,1636276	0,5333639	0,3628907	3,34E-01	0,48	-0,125704366	
DNA topoisomerase 2-alpha	TOP2A	111960000	122300000	1,29	0,87	-0,21	22,358	NaN	-0,9538302	-0,1856218	-0,5168628	NaN	-1,062264	-0,2606703	-0,04324446	-0,1876086	-0,2347708	-0,1053336	-0,01387308	1,41E-02	1,85	-0,356407955	+
Protein S100-A11;Protein S100-A11, N-terminally processed	S100A11	24313000	28268000	1,03	0,87	-0,21	9,7481	-0,2183297	-0,3377216	-0,177588	-0,2498223	-0,1922	-0,1267065	-0,1114925	NaN	-0,6199134	NaN	-0,5698218	0,1634987	7,78E-03	2,11	-0,244009697	+
Heterogeneous nuclear ribonucleoprotein F	HNRNPF	684860000	838150000	1,07	0,87	-0,21	15,367	-0,1163948	-0,1628964	-0,06594402	-0,1346513	-0,318074	-0,2932529	0,006477609	0,09545191	0,2274946	0,1800205	0,1040682	0,2128806	6,94E-01	0,16	-0,022068321	
Acetyl-CoA carboxylase 1;Biotin carboxylase	ACACA	391530000	381440000	0,93	0,87	-0,21	16,758	-0,1893513	-0,2559423	-0,2931291	-0,3199818	-0,1575279	-0,2859699	-0,09264783	0,0311129	0,04488368	0,1905516	-0,1469784	0,06983963	3,33E-02	1,48	-0,117095067	
Putative heat shock protein HSP 90-beta 2	HSP90AB2P	1455000000	1581000000	1,03	0,87	-0,21	0,19785	-0,1812312	-0,1616533	-0,158526	-0,2180948	-0,2447706	-0,2457624	NaN	NaN	-0,2314133	NaN	NaN	-0,1584293	1,79E-06	5,75	-0,1999851	+
Nuclear pore complex protein Nup155	NUP155	219300000	208400000	0,92	0,87	-0,21	15,89	-0,2506288	-0,2023395	-0,0872888	-0,08735009	-0,433292	-0,4471901	NaN	NaN	NaN	-0,1444249	-0,4287601	NaN	2,15E-03	2,67	-0,260159274	+
Mimitin, mitochondrial	NDUFAF2	23879000	28172000	1,16	0,87	-0,21	23,65	-0,2960666	-0,2809831	-0,05757185	-0,2125174	-0,1988248	-0,08274394	NaN	NaN	0,03379299	-0,0235044	NaN	NaN	1,50E-02	1,82	-0,139802375	+
Splicing factor, proline- and glutamine-rich	SFPQ	431550000	487610000	1,07	0,86	-0,21	20,091	-0,6626657	-0,5217634	-0,1600726	-0,1209059	-0,9557598	-0,6834371	0,08038475	-0,1299076	0,1925729	0,152508	0,06239799	0,1025909	7,43E-02	1,13	-0,220338137	
Pyruvate kinase PKM	PKM	3889000000	4478200000	1,02	0,86	-0,21	17,822	-0,416712	-0,4006441	-0,2648418	-0,2849677	-0,2334472	-0,2274217	0,315798	0,244887	0,4376539	0,3615439	0,2413522	0,2734562	9,68E-01	0,01	0,003888053	
28S ribosomal protein S22, mitochondrial	MRPS22	163420000	177720000	1,15	0,86	-0,21	18,728	-0,1382669	-0,1388387	0,04684024	-0,3092165	-0,1537996	-0,2748085	NaN	-0,1849493	-0,1368228	-0,01115178	NaN	NaN	4,73E-03	2,33	-0,144557087	+
40S ribosomal protein SA	RPSA	4532200000	4942300000	1,21	0,86	-0,21	9,9119	-0,2451125	-0,2679479	-0,2911683	-0,2984244	-0,3901883	-0,3167617	-0,198593	-0,1793186	-0,104108	-0,07800012	0,2131294	0,1844707	1,26E-02	1,90	-0,164335225	+
L-lactate dehydrogenase A chain	LDHA	2838700000	3527600000	1,01	0,86	-0,21	16,558	-0,5364821	-0,4924033	-0,2493249	-0,3394912	-0,3800143	-0,3463529	0,0633648	0,07313477	0,140517	0,08732703	0,07272322	0,1036654	7,22E-02	1,14	-0,150278036	
Peroxisomal multifunctional enzyme type 2;(3R)-hydroxyacyl-CoA dehydrogenase;Enoyl-CoA hydratase 2	HSD17B4	112550000	120140000	1,13	0,86	-0,22	12,694	-0,2462928	-0,311381	-0,0526256	-0,1998849	-0,2506631	-0,4595561	-0,2015928	-0,1359664	-0,09923175	-0,3123481	-0,1400146	0,206018	2,64E-03	2,58	-0,183628258	+
B-cell receptor-associated protein 31	BCAP31	86945000	104080000	1,14	0,86	-0,22	10,597	-0,1172552	-0,07947804	-0,24732	-0,2015762	-0,3301931	-0,2158311	-0,09903087	NaN	-0,3348073	-0,125683	-0,09642179	-0,1092654	9,15E-05	4,04	-0,177896533	+
Adenosylhomocysteinase	AHCY	2022300000	2242300000	1,15	0,86	-0,22	8,5888	-0,2192867	-0,1413028	-0,2022233	-0,2318537	-0,2525524	-0,2513498	-0,1884797	-0,1958968	-0,1972691	-0,0788684	-0,1428789	-0,1123967	1,58E-07	6,80	-0,184529874	+
40S ribosomal protein S18	RPS18	788280000	860250000	1,18	0,86	-0,22	18,646	-0,7711505	-0,7070298	-0,7303206	-0,6121963	-0,6707271	-0,6670115	-0,1811004	-0,1546826	-0,01473279	-0,07690405	-0,1646903	-0,1709136	6,34E-04	3,20	-0,41012163	+
Methylosome subunit pICln	CLNS1A	84154000	84670000	0,93	0,86	-0,22	11,876	-0,1733518	-0,3631411	-0,1114769	-0,09086437	-0,2399409	-0,3265159	NaN	NaN	-0,6189604	-0,6734397	NaN	NaN	4,20E-03	2,38	-0,324711382	+

Prefoldin subunit 3	VBP1	49577000	63159000	1,16	0,86	-0,22	12,188	-0,07803053	-0,301497	0,01035019	-0,2108801	-0,04186257	-0,1141602	-0,08298838	-0,09472612	-0,1676852	-0,8666469	0,000144286	0,02984157	4,31E-02	1,37	-0,159845076	
tRNA (cytosine(34)-C(5))-methyltransferase	NSUN2	272010000	301750000	1,09	0,86	-0,22	13,966	-0,2148596	-0,2428232	-0,1817057	-0,2661598	-0,1391087	-0,2207823	-0,03501742	-0,1698907	-0,1114146	-0,139204	-0,2419527	-0,3100389	3,04E-06	5,52	-0,189413138	+
Valine--tRNA ligase	VARS	1249200000	1272500000	1,03	0,86	-0,22	15,713	-0,2999865	-0,2352971	-0,2695122	-0,243028	-0,3078764	-0,2801945	-0,0785027	-0,1227426	0,02005774	-0,02539723	0,07956584	-0,09254009	2,21E-03	2,66	-0,154621157	+
Cytochrome c oxidase subunit 4 isoform 1, mitochondrial	COX4I1	168310000	195800000	1,15	0,86	-0,22	22,863	-0,2026218	-0,1377113	-0,1623473	-0,2118155	-0,1225698	-0,1677175	-0,2712001	-0,2582526	-0,2499252	-0,2306006	-0,2861635	-0,2877652	5,22E-08	7,28	-0,215724201	+
Histone deacetylase 2	HDAC2	90748000	120380000	1,30	0,86	-0,23	21,745	0,3550163	0,1454818	0,1020534	NaN	0,01292609	-0,2845634	NaN	-0,2129187	NaN	0,2589409	-0,08231617	-0,2845634	9,89E-01	0,00	0,001117435	
AP-2 complex subunit beta	AP2B1	197580000	211910000	1,06	0,85	-0,23	16,559	-0,4424184	-0,2758562	-0,1759572	-0,1102461	-0,1283771	-0,5781762	-0,03289039	-0,1505291	-0,1514742	-0,04430035	-0,1906185	0,04530324	3,56E-03	2,45	-0,186295052	+
N-terminal Xaa-Pro-Lys N-methyltransferase 1;N-terminal Xaa-Pro-Lys N-methyltransferase 1, N-terminally processed	NTMT1	82017000	93865000	1,19	0,85	-0,23	7,6293	-0,1658552	-0,2551156	-0,08623166	-0,1542811	-0,000721562	-0,1806261	-0,2965626	-0,565716	-0,4407529	-0,4394807	-0,6324053	-0,5186183	2,56E-04	3,59	-0,311363926	+
NHP2-like protein 1;NHP2-like protein 1, N-terminally processed	NHP2L1	56704000	72502000	1,03	0,85	-0,23	16,755	-0,338141	-0,3098601	-0,005970677	-0,1188836	-0,4338571	-0,3415737	0,323197	0,3192711	0,2144979	0,4773665	0,03463829	0,1368475	9,69E-01	0,01	-0,003538992	
Small nuclear ribonucleoprotein E	SNRPE	251300000	316960000	1,25	0,85	-0,23	2,7097	-0,2589602	-0,2320741	-0,2321927	NaN	-0,4191213	NaN	-0,1595891	-0,1285348	-0,2258687	-0,2768347	-0,102775	-0,2287905	1,98E-05	4,70	-0,226474116	+
Nucleophosmin	NPM1	2273200000	2053800000	1,37	0,85	-0,23	25,292	-0,8599543	-0,7207069	-0,4118479	-0,5974088	-0,746035	-0,847518	0,007482617	0,1548424	0,03731201	-0,1158789	0,2801249	0,3252713	4,82E-02	1,32	-0,291193053	
GrpE protein homolog 1, mitochondrial	GRPEL1	126000000	144350000	1,12	0,85	-0,23	10,987	-0,3325529	-0,2526039	-0,1556304	-0,2760833	-0,2707648	-0,3502098	-0,0110354	-0,02607286	NaN	NaN	-0,06024684	-0,03634831	2,42E-03	2,62	-0,177154851	+
Methionine aminopeptidase 2	METAP2	48774000	50422000	1,31	0,85	-0,23	22,861	-0,7146333	-0,6224875	-0,6530374	NaN	-0,9432222	-0,8641237	-0,1095143	0,005040595	-0,06349963	-0,1970706	-0,07678234	-0,003350929	6,37E-03	2,20	-0,385698315	+
Serine/arginine-rich splicing factor 1	SRSF1	140540000	195050000	0,83	0,85	-0,23	26,494	-0,6883109	-0,6481455	-0,2062623	-0,1995702	-0,7941571	-0,7181416	-0,02861652	0,1230039	0,1463945	0,1966071	0,2055176	0,09653179	9,37E-02	1,03	-0,209595749	
Glutamine--tRNA ligase	QARS	246610000	256380000	1,01	0,85	-0,24	16,893	-0,1806425	-0,1137386	-0,178992	-0,231786	-0,2876771	-0,1975338	-0,3103966	-0,3735141	-0,3450879	-0,2811233	-0,4011204	-0,1923977	7,19E-07	6,14	-0,257834179	+
40S ribosomal protein S12	RPS12	697920000	858180000	1,02	0,85	-0,24	9,7977	-0,2788811	-0,2833338	-0,3077335	-0,1809859	-0,4120014	-0,2877652	-0,2271009	-0,1611531	-0,04814366	-0,1044802	-0,09264783	-0,09961813	4,34E-05	4,36	-0,206987064	+
Splicing factor 3B subunit 1	SF3B1	246240000	206010000	0,93	0,85	-0,24	18,774	-0,2045822	-0,3644963	0,05255495	-0,07723887	-0,485802	-0,5260572	-0,2012942	NaN	-0,2694427	-0,1371082	-0,153944	0,02048441	3,64E-03	2,44	-0,213356933	+
Interleukin enhancer-binding factor 3	ILF3	444420000	457240000	1,05	0,85	-0,24	15,928	-0,5846956	-0,359268	-0,04008153	-0,1202786	-0,506947	-0,5041207	-0,08566514	-0,03889532	-0,1132392	-0,09261702	0,07107621	0,05018827	1,42E-02	1,85	-0,193711972	+
ATP-dependent RNA helicase A	DHX9	1340300000	1285400000	0,88	0,85	-0,24	21,12	-0,7242301	-0,6548989	-0,1958473	-0,1958473	-0,8202596	-0,705947	0,116764	0,04753833	0,1095611	0,05255495	0,06695026	0,1373724	6,14E-02	1,21	-0,230524096	
Filamin-B	FLNB	858130000	830280000	0,96	0,85	-0,24	19,186	-0,4196615	-0,4238552	-0,4286824	-0,3845272	-0,3626587	-0,3200898	0,27453	0,2705888	0,1901724	0,2492632	0,2519311	0,1986199	4,47E-01	0,35	-0,075364119	
28S ribosomal protein S29, mitochondrial	DAP3	127100000	135480000	1,03	0,84	-0,24	10,54	-0,2782511	-0,2187495	-0,03255102	-0,2097618	-0,3183079	-0,3146608	-0,4812836	-0,2473884	-0,03580105	0,1553607	NaN	NaN	7,24E-03	2,14	-0,198139438	+
Heterogeneous nuclear ribonucleoprotein A/B	HNRNPAB	141180000	142930000	1,04	0,84	-0,25	4,3013	-0,3255213	-0,2642179	-0,2021403	-0,07419803	-0,262123	-0,3142481	NaN	NaN	-0,3480781	-0,3173009	NaN	NaN	7,05E-05	4,15	-0,263478451	+
ATP-binding cassette sub-family F member 1	ABCF1	96610000	83279000	0,79	0,84	-0,25	17,585	-0,122507	-0,3300117	-0,2507833	-0,3516082	-0,3138895	-0,293465	-0,1379336	-0,3172469	-0,02155539	-0,01086098	NaN	-0,6829043	1,06E-03	2,98	-0,257524165	+
40S ribosomal protein S14	RPS14	706200000	623060000	0,71	0,84	-0,25	14,904	-0,7629014	-0,8650168	-0,1977323	-0,6686846	-0,4786678	-0,5469619	-0,1497126	-0,1229154	-0,1120381	-0,01978458	-0,03042763	-0,08662991	3,00E-03	2,52	-0,336789421	+
Nucleosome assembly protein 1-like 1	NAP1L1	1195500000	1277500000	1,02	0,84	-0,25	8,2447	-0,2627805	-0,2587013	-0,1955829	-0,2679132	-0,2494278	-0,2692514	-0,4136724	-0,3601936	NaN	NaN	-0,3152889	-0,3964983	2,94E-07	6,53	-0,298931038	+
28S ribosomal protein S34, mitochondrial	MRPS34	36324000	33977000	1,11	0,84	-0,25	12,9	-0,09930895	-0,4619585	NaN	-0,2747562	NaN	-0,9217181	-0,1214551	NaN	0,1347467	NaN	NaN	NaN	1,10E-01	0,96	-0,290741686	
40S ribosomal protein S21	RPS21	144100000	157990000	1,03	0,84	-0,25	7,3724	-0,3051822	-0,3721877	-0,3197657	-0,4657989	-0,4206267	-0,3642735	-0,2713567	-0,1908819	-0,1140508	0,009633875	-0,03361381	-0,05298471	2,98E-04	3,53	-0,241757401	+
Gamma-aminobutyric acid receptor-associated protein-like 2	GABARAPL2	39659000	38846000	0,98	0,84	-0,25	14,189	-0,3669688	-0,164755	-0,2779363	0,09828482	-0,4279253	-0,1890058	NaN	-0,4065028	NaN	-0,2555289	NaN	NaN	4,39E-03	2,36	-0,248792239	+
2,4-dienoyl-CoA reductase, mitochondrial	DECR1	93895000	102750000	1,03	0,84	-0,26	3,9597	-0,3838306	-0,3923832	-0,0783656	-0,1397761	-0,1356494	-0,3663177	-0,1684794	-0,003828249	-0,1574475	-0,1756313	-0,3059133	-0,3139612	1,08E-04	3,97	-0,218465287	+
Peptidyl-prolyl cis-trans isomerase D	PPID	81556000	91720000	1,13	0,84	-0,26	11,284	-0,2651539	-0,1625411	-0,2789686	-0,3298667	-0,1247861	-0,2630748	-0,2360956	-0,07387913	-0,1879044	-0,2942786	NaN	NaN	1,25E-05	4,90	-0,221654888	+
Dynein light chain 1, cytoplasmic	DYNLL1	33725000	36864000	1,04	0,84	-0,26	8,861	-0,2998444	-0,2541345	NaN	-0,125683	-0,3891487	NaN	-0,2858468	-0,08810135	-0,3159892	-0,2518995	-0,1954342	-0,3557746	1,44E-05	4,84	-0,256185626	+
Eukaryotic translation initiation factor 3 subunit G	EIF3G	245460000	239350000	0,99	0,84	-0,26	17,575	-0,3302656	-0,3843954	-0,2789337	-0,3216035	-0,2530166	-0,5250604	-0,3615276	NaN	-0,1649329	-0,1411915	0,004752986	-0,2828424	6,52E-05	4,19	-0,276274235	+
Glutamate-rich WD repeat-containing protein 1	GRWD1	32857000	35343000	1,08	0,84	-0,26	20,946	-0,7987425	-0,4826336	-0,2171721	NaN	NaN	-0,4055088	NaN	NaN	NaN	NaN	-0,2637848	-0,3501915	4,39E-03	2,36	-0,419672211	+
Eukaryotic translation initiation factor 2 subunit 3;Putative eukaryotic translation initiation factor 2 subunit 3-like protein	EIF2S3;EIF2S3L	788660000	897700000	1,08	0,83	-0,26	16,644	-0,3538373	-0,331663	-0,4688107	-0,4196808	-0,4651615	-0,4976082	0,06915226	-0,02508894	NaN	NaN	0,08841301	0,09153062	1,83E-02	1,74	-0,231275469	+
Splicing factor 3B subunit 6	SF3B6	37280000	41716000	1,00	0,83	-0,26	2,3381	-0,2961019	-0,3445749	NaN	-0,1075857	-0,5677247	-0,5473202	-0,1160508	-0,114988	-0,252329	-0,1843262	-0,2140394	-0,09297091	4,95E-04	3,31	-0,258001075	+
40S ribosomal protein S24	RPS24	347390000	380650000	1,27	0,83	-0,27	16,602	-0,8102829	-0,8343388	-0,7214204	-0,7941321	-0,8319229	-0,7822233	-0,2275231	-0,1738888	-0,09277089	-0,05860819	-0,06606483	-0,1633003	8,14E-04	3,09	-0,463039713	+
Nuclease-sensitive element-binding protein 1	YBX1	881490000	929810000	1,18	0,83	-0,27	19,087	-0,5059838	-0,4742719	-0,3531184	-0,372636	-0,4249782	-0,4993815	0,04586229	0,05505682	0,04054174	0,1015157	0,03829577	0,1223414	3,58E-02	1,45	-0,185563015	
Serine/arginine-rich splicing factor 3	SRSF3	239980000	205910000	1,40	0,83	-0,27	28,985	-0,9835608	-0,9844168	-0,6132333	-0,5850202	-1,028587	-0,8924193	-0,1859501	NaN	-0,1845557	-0,2592537	-0,1104953	-0,1371082	7,97E-04	3,10	-0,542236413	+
Heterogeneous nuclear ribonucleoprotein Q	SYNCRIP	1301400000	1383600000	1,00	0,83	-0,27	12,922	-0,5134813	-0,5286356	-0,3649608	-0,3417017	-0,4394807	-0,5199211	0,0265876	-0,05078633	-0,1684794	-0,07998114	0,07833651	-0,001067978	4,21E-03	2,38	-0,241964321	+
ATPase family AAA domain-containing protein 3A	ATAD3A	125070000	142750000	0,99	0,83	-0,27	14,687	-0,4855798	-0,3251959	-0,2778138	-0,2209505	-0,2552878	-0,3059846	NaN	NaN	NaN	NaN	0,03351105	-0,1719534	1,93E-03	2,71	-0,251156843	+
Extended synaptotagmin-1	ESYT1	844750000	809030000	0,97	0,83	-0,28	14,16	-0,3240215	-0,283211	-0,3447214	-0,3335887	-0,3116137	-0,2637675	-0,228182	-0,1021865	-0,251951	-0,2407931	-0,1724248	-0,06825642	1,28E-06	5,89	-0,243726473	+
60S ribosomal protein L19	RPL19	230020000	213530000	1,31	0,82	-0,28	29,929	-1,034574	-0,9966856	-0,8022101	-0,7966109	-1,051459	-1,130839	-0,1215964	-0,1537675	-0,04960625	-0,125683	-0,100484	-0,1035034	1,86E-03	2,73	-0,538918278	+
Poly(rC)-binding protein 2	PCBP2	959530000	1013400000	1,01	0,82	-0,28	16,225	-0,4148451	-0,29108	-0,301746	-0,3082157	-0,4046682	-0,5089165	-0,1161602	-0,2674443	-0,1546023	-0,2836499	-0,05677631	-0,1334322	2,48E-05	4,61	-0,270128057	+
Osteoclast-stimulating factor 1	OSTF1	20546000	27997000	1,08	0,82	-0,28	7,3073	-0,2588911	NaN	-0,4276536	-0,2451125	-0,2790211	-0,2855654	NaN	0,08420031	NaN	0,1070183	NaN	0,03181863	6,14E-02	1,21	-0,159150807	
Ribonuclease P protein subunit p30	RPP30	9295100	9188200	1,06	0,82	-0,28	13,996	-0,2953404	-0,2912918	-0,3192436	-0,1499367	-0,3839247	-0,504223	NaN	NaN	-0,0874728	-0,09805768	NaN	NaN	1,30E-03	2,89	-0,266186344	+
Proteasome activator complex subunit 3	PSME3	178200000	199180000	0,99	0,82	-0,28	8,2751	-0,1999015	-0,3049683	-0,3480229	-0,3387064	-0,3722064	-0,227084	-0,2883113	-0,2886989	-0,2683475	-0,2681043	-0,2499596	-0,1710598	5,70E-09	8,24	-0,277114256	+
Methylosome protein 50	WDR77	156170000	139210000	0,99	0,82	-0,28	6,8676	-0,6610908	NaN	-0,2846338	-0,1938657	NaN	-0,4587033	-0,1881016	0,206143	-0,09879906	-0,02994152	NaN	NaN	5,62E-02	1,25	-0,213624087	
Heterogeneous nuclear ribonucleoprotein U	HNRNPU	2489900000	2368800000	0,89	0,82	-0,29	14,675	-0,4352024	-0,3620652	-0,2142402	-0,1663408	-0,4660779	-0,2940132	-0,1089075	-0,1082386	-0,2145582	-0,1826223	-0,1009791	-0,07257376	1,06E-04	3,97	-0,227151605	+
Replication factor C subunit 4	RFC4	44677000	49461000	1,07	0,82	-0,29	23,417	NaN	-0,2494792	-0,4344223	-0,262971	-0,3795637	-0,2489477	-0,04333369	-0,2293824	-0,3242202	-0,3630112	NaN	NaN	7,36E-05	4,13	-0,281703477	+
Eukaryotic initiation factor 4A-II;Eukaryotic initiation factor 4A-II, N-terminally processed	EIF4A2	187340000	194510000	1,02	0,82	-0,29	14,031	-0,1602983	-0,2874482	-0,004985869	-0,07096649	-0,2830354	-0,3083229	NaN	NaN	NaN	NaN	-0,5069061	-0,5782408	5,70E-03	2,24	-0,275025514	+
Mannosyl-oligosaccharide glucosidase	MOGS	55029000	67207000	1,03	0,82	-0,29	23,552	-0,8040477	-0,6586974	-0,5615368	-0,4063881	-0,6942512	-0,5639448	-0,03349569	0,09922788	-0,06650294	0,01692422	0,1781091	0,2086421	2,82E-02	1,55	-0,273830114	+
Eukaryotic initiation factor 4A-I	EIF4A1	4511000000	4606600000	1,09	0,82	-0,29	5,5148	-0,2992052	-0,2682607	-0,2718096	-0,3667083	-0,4538933	-0,4403418	-0,1687875	-0,210229	0,2179751	NaN	-0,2888399	-0,2289427	8,98E-04	3,05	-0,252640254	+
Proliferation-associated protein 2G4	PA2G4	2034400000	2486500000	1,00	0,82	-0,29	18,65	-0,4535772	-0,4238939	-0,3187756	-0,2785311	-0,4389525	-0,4520764	0,1355349	0,1129003	0,1576905	0,117163	0,03294719	0,07423148	8,79E-02	1,06	-0,144611618	
Pre-mRNA-processing factor 19	PRPF19	333300000	388260000	0,98	0,82	-0,29	23,966	-0,2873602	-0,3563839	-0,1748005	-0,2665242	-0,3381045	-0,455396	0,2006298	0,04348443	0,3029917	0,2202061	0,1658154	0,1356662	4,06E-01	0,39	-0,067481301	
General transcription factor II-I	GTF2I	112240000	108910000	0,85	0,82	-0,29	16,511	-0,07243722	-0,1254784	-0,5832251	-0,2752799	-0,2481935	-0,1678472	-0,1917221	0,1721035	0,1905516	NaN	0,5617904	-0,07804578	4,28E-01	0,37	-0,074343982	
Centromere/kinetochore protein zw10 homolog	ZW10	46875000	56311000	1,11	0,81	-0,30	13,555	0,1112984	0,3494785	-0,07863981	0,3318198	-0,2821931	NaN	-0,5280114	-0,5108682	-0,5297808	-0,2502855	-0,5743259	-0,5132753	6,10E-02	1,21	-0,224980308	
Mitochondrial import inner membrane translocase subunit Tim13	TIMM13	35469000	39627000	1,17	0,81	-0,30	13,66	-0,4082823	-0,4555146	-0,3383781	-0,4832989	-0,2949157	-0,4847316	-0,1724248	-0,1921175	-0,2771493	-0,3536346	-0,2018085	-0,3445016	4,67E-07	6,33	-0,333896454	+
WD repeat-containing protein 61;WD repeat-containing protein 61, N-terminally processed	WDR61	18011000	20722000	1,12	0,81	-0,30	8,8252	-0,3443185	-0,1764951	NaN	NaN	-0,3644406	-0,3066446	NaN	NaN	-0,2789337	NaN	-0,2247724	-0,2043661	5,79E-05	4,24	-0,271424432	+
Heterogeneous nuclear ribonucleoprotein R	HNRNPR	431750000	463230000	0,95	0,81	-0,30	17,332	-0,6713244	-0,5001158	-0,1453659	-0,1070417	-0,4914091	-0,444655	-0,1569166	-0,3553315	-0,05434738	0,1255188	0,1427401	0,3133619	4,65E-02	1,33	-0,195407223	
116 kDa U5 small nuclear ribonucleoprotein component	EFTUD2	606890000	550400000	0,89	0,81	-0,30	18,406	-0,4346173	-0,2689558	-0,3784943	-0,213136	-0,5789087	-0,5200038	-0,1631871	-0,2545475	-0,2208832	-0,1716446	-0,2509721	-0,249788	8,24E-06	5,08	-0,308761545	+
40S ribosomal protein S9	RPS9	1640200000	1507200000	1,27	0,81	-0,30	25,275	-0,9648265	-0,9281824	-0,7452124	-0,7104724	-0,870229	-0,7994455	-0,1689497	-0,2417821	-0,1450947	-0,1457649	-0,1004531	-0,1868858	5,24E-04	3,28	-0,50060821	+
Nucleoporin p54	NUP54	37988000	42589000	1,02	0,81	-0,31	18,565	NaN	-0,2260711	NaN	NaN	-0,5370264	-0,3915502	NaN	NaN	NaN	NaN	-0,2225992	-0,3814606	3,94E-03	2,40	-0,351741511	+
28S ribosomal protein S25, mitochondrial	MRPS25	19784000	21955000	0,97	0,81	-0,31	5,5988	-0,271113	-0,3907746	-0,3254309	-0,3058775	NaN	-0,172181	-0,04629518	NaN	NaN	-0,08178167	NaN	NaN	3,57E-03	2,45	-0,227636266	+
Heterogeneous nuclear ribonucleoprotein D-like	HNRNPDL	40127000	50969000	1,00	0,80	-0,32	9,0375	-0,3271492	-0,1962274	0,1651723	0,06281248	NaN	-0,5306349	NaN	NaN	-0,4808809	-0,4233134	NaN	NaN	5,24E-02	1,28	-0,247174445	
Importin-4	IPO4	246670000	220510000	0,88	0,80	-0,32	9,7008	-0,3325166	-0,3073585	-0,4718688	-0,3674153	-0,3467015	-0,2465667	-0,1516825	-0,210997	0,1064823	-0,06020174	-0,1838018	NaN	6,95E-04	3,16	-0,233875281	+
Glutathione S-transferase kappa 1	GSTK1	125540000	132210000	1,02	0,80	-0,32	8,6044	-0,2375066	-0,3451796	-0,3491252	-0,4131345	-0,4094507	-0,4056426	-0,1290553	0,04376442	NaN	-0,2526384	0,0426443	0,2112614	1,09E-02	1,96	-0,204005701	+
Importin-7	IPO7	392700000	368400000	0,91	0,80	-0,32	6,5948	-0,3496031	-0,2989745	-0,3540217	-0,2712697	-0,2965803	-0,316582	-0,3405321	-0,4206074	-0,3294134	-0,3184159	-0,3951506	-0,3804462	2,11E-11	10,68	-0,339299736	+
Eukaryotic translation initiation factor 3 subunit I	EIF3I	186910000	194090000	1,07	0,80	-0,32	9,0011	-0,2307868	-0,2051641	-0,1909478	-0,2707997	-0,4014633	-0,4707088	-0,4253656	-0,539184	-0,4696695	-0,4251331	NaN	-0,8472066	3,13E-05	4,50	-0,406948128	+
DNA-directed RNA polymerases I, II, and III subunit RPABC3	POLR2H	61014000	62159000	0,97	0,80	-0,33	17,525	-0,3684021	-0,3725799	0,3701643	-0,3204501	-0,2869553	-0,2832286	-0,5607492	0,2735755	-0,3907935	-0,6280509	0,095722	-0,05529143	4,10E-02	1,39	-0,21058659	
Fructose-bisphosphate aldolase A	ALDOA	7371100000	7803800000	0,93	0,80	-0,33	20,897	-0,5678531	-0,5740036	-0,3703403	-0,3951506	-0,4956339	-0,4505772	0,1431322	0,1237986	0,09747601	0,1182268	-0,01854186	0,1928254	6,89E-02	1,16	-0,183053473	
Serine/threonine-protein phosphatase PP1-gamma catalytic subunit	PPP1CC	47595000	54900000	1,08	0,80	-0,33	5,4093	NaN	NaN	NaN	-0,2636289	NaN	-0,3312455	NaN	NaN	-0,4143259	-0,3701352	-0,3653881	-0,297502	2,14E-05	4,67	-0,340370928	+
Nuclear pore membrane glycoprotein 210	NUP210	288770000	259280000	0,86	0,79	-0,33	11,143	-0,3357357	-0,3186857	-0,3328981	-0,3064127	-0,393122	-0,4117903	-0,3294678	-0,2644432	-0,1130519	-0,2255988	-0,2996313	-0,1766093	1,39E-07	6,86	-0,29228723	+
Hemoglobin subunit theta-1	HBQ1	18994000	21785000	1,16	0,79	-0,33	24,23	0,08229379	0,03689012	0,02998291	NaN	-0,03345142	NaN	-0,6431801	-0,5066602	-0,6626428	-0,6239097	-0,6076167	-0,565887	8,48E-03	2,07	-0,349418109	+
Clustered mitochondria protein homolog	CLUH	191970000	171340000	0,88	0,79	-0,33	8,8205	-0,3189736	-0,2577351	-0,433896	-0,4184848	-0,3562362	-0,3060915	-0,3713664	NaN	-0,360453	-0,3727854	-0,03246246	-0,2525868	2,55E-06	5,59	-0,316461025	+
Neutral amino acid transporter B(0)	SLC1A5	300290000	292700000	0,95	0,79	-0,34	10,459	-0,2786011	-0,08742674	-0,2364014	-0,3118644	-0,3018349	-0,3400937	-0,6025257	-0,4169239	-0,4851758	-0,4840252	-0,4882081	-0,5732307	2,58E-06	5,59	-0,383859307	+
40S ribosomal protein S2	RPS2	780240000	898630000	1,07	0,79	-0,34	17,844	-0,8590381	-0,7587699	-0,6439914	-0,5699288	-0,7580622	-0,6736927	-0,1825568	-0,1687065	-0,08840814	-0,1019697	-0,05162341	-0,1009172	9,43E-04	3,03	-0,41313875	+
Elongation factor 1-alpha 1;Putative elongation factor 1-alpha-like 3	EEF1A1;EEF1A1P5	22696000000	2,2445E+10	1,05	0,79	-0,34	28,397	-0,240435	-0,2269489	-0,335681	-0,3403677	-0,374823	-0,3439888	-0,4135571	-0,4215733	-0,3368285	-0,3288153	-0,4597941	-0,3737946	1,77E-09	8,75	-0,349717285	+
PHD finger-like domain-containing protein 5A	PHF5A	10900000	12462000	1,00	0,79	-0,34	7,4949	-0,3424149	-0,3395277	0,1772162	-0,009931016	-0,4170587	-0,3190816	0,01663897	NaN	-0,118821	-0,176805	-0,09366352	-0,02551478	2,36E-02	1,63	-0,149905716	+
Phosphatidylinositol-binding clathrin assembly protein	PICALM	157520000	188380000	1,04	0,79	-0,35	6,1653	-0,284458	-0,446148	-0,1199023	-0,4019207	-0,5254341	-0,2896861	-0,4055852	-0,2638715	-0,4065602	-0,3257021	-0,5611323	-0,5217012	5,91E-07	6,23	-0,379341799	+
28S ribosomal protein S7, mitochondrial	MRPS7	120920000	117200000	0,98	0,79	-0,35	13,314	-0,3447214	-0,5088549	-0,3804274	-0,3153607	-0,4163462	-0,3154505	0,2056428	-0,07302895	-0,2106797	-0,4099298	NaN	0,1231364	6,18E-03	2,21	-0,24054731	+
DNA mismatch repair protein Msh6	MSH6	83765000	79742000	0,93	0,78	-0,35	15,527	-0,285372	-0,2064786	-0,4293622	-0,4126734	-0,3216577	-0,3248706	-0,5403376	-0,2170044	-0,2815266	-0,3739254	-0,09474155	-0,9328844	8,84E-05	4,05	-0,368402869	+
60S ribosomal protein L22	RPL22	818250000	725930000	0,73	0,78	-0,35	29,149	-0,7431585	-0,7897857	-0,6514276	-0,7500572	-0,8182486	-0,9054666	-0,09455671	-0,06404249	-0,04766648	-0,02275668	-0,07859414	-0,09078754	2,54E-03	2,60	-0,421379008	+
Heterogeneous nuclear ribonucleoprotein A1;Heterogeneous nuclear ribonucleoprotein A1, N-terminally processed;Heterogeneous nuclear ribonucleoprotein A1-like 2	HNRNPA1;HNRNPA1L2	897940000	916480000	0,87	0,78	-0,35	18,255	-0,599637	-0,6736237	-0,2546336	-0,2509034	-0,6206896	-0,706606	-0,1352691	-0,1247861	-0,2056298	-0,2261218	-0,08545081	-0,1683173	4,54E-04	3,34	-0,337639026	+
Eukaryotic translation initiation factor 3 subunit B	EIF3B	865460000	788910000	0,96	0,78	-0,36	12,29	-0,3071443	-0,3076799	-0,354575	-0,360027	-0,5035888	-0,5207694	-0,3340797	-0,3622321	-0,32829	-0,265466	-0,2850555	-0,308841	9,24E-09	8,03	-0,353145726	+
Protein LSM12 homolog	LSM12	50439000	59337000	1,16	0,78	-0,36	7,74	-0,2974488	-0,2291117	-0,2993295	-0,1629609	-0,1597342	-0,2558734	-0,3813479	-0,4240294	-0,2772542	-0,2755243	-0,2736047	-0,2789162	6,74E-08	7,17	-0,276261264	+
60S ribosomal protein L32	RPL32	445510000	369110000	1,20	0,78	-0,36	29,982	-1,324491	-1,156499	-0,8642813	-0,9336283	-1,261777	-1,073545	-0,3421223	-0,2719489	-0,1878879	-0,181853	-0,2205469	-0,2281482	4,33E-04	3,36	-0,670560709	+
DNA-directed RNA polymerase II subunit RPB2	POLR2B	87746000	75774000	0,81	0,78	-0,36	16,712	-1,068877	-0,4081483	0,01178193	-0,3914935	-0,4505575	-0,5010548	0,1857398	-0,3434578	-0,3573631	-0,2180276	NaN	NaN	8,17E-03	2,09	-0,35414577	+
40S ribosomal protein S17	RPS17	349360000	403420000	1,15	0,78	-0,36	19,153	-0,7016231	-0,6672405	-0,5195488	-0,5897892	-0,7041124	-0,6710486	-0,2728375	-0,1231668	-0,08135428	-0,1090475	-0,02716032	-0,2275907	4,38E-04	3,36	-0,391209981	+
Guanine nucleotide-binding protein subunit beta-2-like 1;Guanine nucleotide-binding protein subunit beta-2-like 1, N-terminally processed	GNB2L1	1528900000	1566600000	0,94	0,78	-0,36	6,4721	-0,3878835	-0,4468361	-0,3864117	-0,412635	-0,554909	-0,505738	-0,3239132	-0,312563	-0,2181452	-0,2433695	-0,3127601	-0,3132619	6,77E-08	7,17	-0,368202171	+

60S ribosomal protein L28	RPL28	233920000	253130000	1,11	0,78	-0,36	22,602	-1,014616	-1,028617	-0,8333617	-0,8072252	-0,9978375	-1,011908	-0,3619169	-0,3501362	-0,1946415	-0,2292639	-0,189088	-0,1695012	1,81E-04	3,74	-0,5990094	+
60S ribosomal protein L36a;60S ribosomal protein L36a-like	RPL36A;RPL36AL	26266000	31173000	1,02	0,78	-0,36	27,202	NaN	-1,009205	NaN	NaN	NaN	-0,6330987	-0,2707474	-0,1490086	-0,08364569	-0,01979913	-0,1960126	-0,1055509	3,74E-02	1,43	-0,308383482	
DNA repair protein RAD50	RAD50	74447000	65127000	0,89	0,78	-0,37	12,357	-0,3129393	-0,2565453	-0,1737749	-0,3517739	-0,5256627	-0,4518199	-0,5787579	-0,5444978	-0,5302182	-0,6055956	-0,4377796	NaN	1,40E-06	5,85	-0,433578643	+
Cyclin-dependent kinase 1	CDK1	444420000	453070000	0,97	0,78	-0,37	10,561	-0,2570108	-0,3504673	-0,3064663	-0,3160431	-0,3904719	-0,3890544	-0,4292845	-0,3756651	-0,4718088	-0,4852768	-0,3308825	-0,3458211	9,20E-10	9,04	-0,370687711	+
PDZ and LIM domain protein 1	PDLIM1	53807000	49607000	0,94	0,78	-0,37	18,108	-0,5006465	-0,7219915	-0,3342798	-0,5518603	-0,5135636	-0,4393633	0,2041406	0,3149864	0,1934563	0,2974849	0,2579761	0,2338881	2,93E-01	0,53	-0,12998105	
HBS1-like protein	HBS1L	17972000	17563000	0,86	0,77	-0,37	8,0881	NaN	NaN	-0,4514252	-0,5139756	-0,6363692	-0,257597	NaN	NaN	-0,2572004	-0,3927053	NaN	NaN	9,80E-04	3,01	-0,418212111	+
Eukaryotic translation initiation factor 3 subunit L	EIF3L	231430000	237200000	0,92	0,77	-0,37	13,062	-0,3014792	-0,2948979	-0,2084276	-0,3365916	-0,5149443	-0,5179571	NaN	-0,6129904	NaN	NaN	-0,5403795	-0,3841882	1,91E-05	4,72	-0,412428432	+
Thymidine kinase, cytosolic	TK1	95248000	103530000	1,09	0,77	-0,37	17,177	-0,1336538	-0,3950557	-0,4235263	-0,2322265	-0,2690254	-0,3935199	NaN	-0,2718618	-0,8571814	-0,7985669	NaN	-0,07181545	1,14E-03	2,94	-0,384643302	+
Myosin regulatory light chain 12A;Myosin regulatory light chain 12B	MYL12A;MYL12B	55950000	55909000	1,04	0,77	-0,37	24,092	-0,7473906	-0,9248645	0,3236583	0,2565276	-0,7791005	-0,5175234	NaN	-0,252329	0,4418021	-0,3709745	-0,07734546	-0,217474	9,29E-02	1,03	-0,260455816	
Chromobox protein homolog 3	CBX3	185970000	200630000	1,20	0,77	-0,38	16,326	-0,8188593	-0,8559015	-0,409374	-0,4488236	-1,079173	-1,192447	0,02573757	0,0734089	-0,1722785	-0,2501483	-0,06579299	0,0610158	7,57E-03	2,12	-0,427719681	+
Cell division cycle 5-like protein	CDC5L	21979000	20793000	0,84	0,77	-0,38	21,834	-0,3343526	-0,751708	NaN	NaN	NaN	NaN	NaN	NaN	-0,1840311	0,02034214	NaN	NaN	1,52E-01	0,82	-0,312437393	
40S ribosomal protein S16	RPS16	1495300000	1370900000	0,82	0,77	-0,38	20,38	-0,7271646	-0,7760338	-0,6697858	-0,6035555	-0,7047471	-0,6399841	-0,1717746	-0,1103395	-0,09412542	-0,09217101	-0,08699767	-0,07931032	9,48E-04	3,02	-0,396332463	+
Guanine nucleotide-binding protein G(k) subunit alpha	GNAI3	109880000	112260000	0,96	0,77	-0,39	6,8277	-0,3107903	-0,3027956	-0,3372294	-0,4623958	-0,3981514	-0,3841506	-0,9867593	-0,2864625	-0,3803898	-0,3477843	NaN	NaN	1,19E-04	3,92	-0,419690886	+
40S ribosomal protein S5;40S ribosomal protein S5, N-terminally processed	RPS5	594300000	625460000	1,20	0,77	-0,39	27,71	-0,9099866	-0,9409216	-0,7961097	-0,7457204	-0,7981655	-0,8756983	-0,2119491	-0,3596752	-0,2435574	-0,1195104	-0,2218924	-0,1965085	1,63E-04	3,79	-0,534974597	+
Trifunctional purine biosynthetic protein adenosine-3;Phosphoribosylamine--glycine ligase;Phosphoribosylformylglycinamidine cyclo-ligase;Phosphoribosylglycinamide formyltransferase	GART	2348900000	2388000000	0,88	0,76	-0,39	13,528	-0,4156532	-0,2930937	-0,4338181	-0,3651651	-0,428197	-0,3393634	-0,2647898	-0,3180021	-0,6784183	-0,6682948	-0,5291768	-0,2439332	8,11E-07	6,09	-0,414825467	+
Insulin-like growth factor 2 mRNA-binding protein 3	IGF2BP3	193570000	175340000	0,91	0,76	-0,39	17,804	-0,785353	-0,7477781	-0,369986	-0,3634752	-0,7258995	-0,6800815	-0,1447119	-0,04911355	-0,1398079	-0,2985841	0,006908315	-0,01305762	1,79E-03	2,75	-0,359244991	+
Poly [ADP-ribose] polymerase 1	PARP1	1063800000	1038500000	0,86	0,76	-0,39	22,351	-0,1114925	-0,008696752	-0,7321886	-0,7050293	-0,5746052	-0,4974861	0,06115411	0,07737969	0,09153062	0,03646811	0,09585687	0,03829577	8,16E-02	1,09	-0,185734439	
Cold-inducible RNA-binding protein	CIRBP	36168000	39001000	0,89	0,76	-0,40	29,52	-0,8081594	-0,709151	NaN	-0,3503937	NaN	-0,6376703	-0,3618428	-0,4563062	-0,170329	-0,1697284	NaN	-0,08298838	1,27E-03	2,90	-0,416285475	+
PDZ and LIM domain protein 5	PDLIM5	30470000	28654000	1,04	0,76	-0,40	10,17	NaN	-0,5458241	0,03562387	-0,07116351	-0,3015681	-0,5290103	NaN	NaN	NaN	NaN	-0,7893867	-0,2267126	1,97E-02	1,71	-0,346863068	+
DNA-directed RNA polymerases I and III subunit RPAC1	POLR1C	146600000	154910000	1,01	0,76	-0,40	12,746	-0,3539849	-0,294951	-0,6124169	-0,5557994	-0,6502497	-0,5888342	-0,2745293	-0,2415945	-0,2478166	-0,2584424	NaN	NaN	3,59E-05	4,45	-0,407861879	+
39S ribosomal protein L41, mitochondrial	MRPL41	8495100	10379000	0,92	0,75	-0,41	9,3581	-0,358454	-0,4062352	NaN	NaN	-0,3057528	NaN	NaN	-0,1750448	NaN	-0,1633003	NaN	NaN	4,42E-03	2,35	-0,281757408	+
Hsp70-binding protein 1	HSPBP1	29657000	31383000	0,97	0,75	-0,41	15,805	-0,3583245	-0,1963431	-0,6125492	-0,2965449	-0,3083229	-0,2630748	NaN	NaN	-0,5696933	-0,3997112	NaN	-0,3214773	4,29E-05	4,37	-0,369560144	+
60S ribosomal protein L11	RPL11	777860000	664600000	1,16	0,75	-0,41	19,02	-1,107741	-1,128976	-0,9603001	-0,9035761	-1,081003	-1,158365	-0,3912475	-0,3278915	-0,2612581	-0,2327859	-0,1477614	-0,2004319	2,31E-04	3,64	-0,658444855	+
Cytochrome c oxidase subunit 6C	COX6C	15797000	15010000	0,96	0,75	-0,42	10,043	-0,4177908	-0,7255893	NaN	-0,4543479	-0,4348902	NaN	NaN	NaN	-0,1881838	-0,2148429	-0,2562696	NaN	1,58E-03	2,80	-0,384559225	+
Eukaryotic translation initiation factor 3 subunit F	EIF3F	243110000	230660000	0,93	0,75	-0,42	6,9918	-0,288276	-0,244839	-0,1021555	-0,2497537	-0,6718988	-0,6546037	-0,5134813	-0,3238049	-0,4565041	-0,493459	-0,1317874	NaN	8,17E-05	4,09	-0,375505752	+
Polyadenylate-binding protein 2	PABPN1	108790000	130010000	0,88	0,74	-0,43	11,061	-0,3504122	-0,4572566	NaN	-0,2624344	NaN	-0,4785472	NaN	-0,9536067	NaN	NaN	NaN	0,05921687	2,97E-02	1,53	-0,407173353	
60S ribosomal protein L31	RPL31	259030000	319870000	0,99	0,74	-0,43	25,131	-1,056761	-0,767462	-0,6734627	-0,6950218	-1,084089	-0,8406034	-0,2352122	-0,3300481	-0,3268416	-0,2377107	-0,3276019	-0,3373569	5,56E-05	4,25	-0,57601429	+
Mitochondrial import inner membrane translocase subunit Tim23;Putative mitochondrial import inner membrane translocase subunit Tim23B	TIMM23;TIMM23B	64122000	67688000	1,12	0,74	-0,43	8,1423	-0,1686254	-0,03398309	-0,4382486	-0,2641312	-0,5366287	-0,3722064	-0,316582	NaN	-0,3024218	-0,3456194	-0,4773817	-0,4876215	1,85E-05	4,73	-0,340313623	+
Cytoplasmic dynein 1 light intermediate chain 1	DYNC1LI1	123150000	131560000	0,93	0,74	-0,43	14,421	-0,4957966	-0,3425978	-0,5206453	-0,1742632	-0,4876215	-0,4510505	NaN	NaN	NaN	NaN	-0,07827426	-0,2871666	4,81E-04	3,32	-0,354676971	+
Enoyl-CoA hydratase, mitochondrial	ECHS1	42993000	44028000	0,96	0,74	-0,44	12,726	-0,553278	-0,4809816	-0,4881879	-0,5153978	-0,6614786	-0,4409096	-0,2725935	-0,4430654	-0,3133516	-0,3306648	-0,08260643	-0,2578731	2,39E-06	5,62	-0,403365688	+
Eukaryotic translation initiation factor 1	EIF1	47308000	53338000	1,04	0,74	-0,44	14,303	-0,4101981	-0,3444833	NaN	NaN	-0,7336512	NaN	-0,443811	-0,4633502	-0,4946172	-0,4742318	-0,3644777	-0,3236603	3,97E-06	5,40	-0,450275643	+
60S ribosomal protein L23	RPL23	700840000	739560000	0,87	0,74	-0,44	15,355	-0,7110391	-0,5903103	-0,6082983	-0,4857414	-0,7054293	-0,6461121	-0,08457856	-0,08225508	-0,07722362	-0,01990157	0,0182068	0,01564032	3,71E-03	2,43	-0,33142019	+
Heat shock cognate 71 kDa protein	HSPA8	19117000000	1,8204E+10	0,98	0,74	-0,44	8,4601	-0,4067323	-0,4554355	-0,5087523	-0,4977305	-0,5612814	-0,5566692	-0,4287213	-0,3667455	-0,4630519	-0,4513069	-0,4198738	-0,3713477	5,60E-11	10,25	-0,457304026	+
ATP-dependent RNA helicase DDX42	DDX42	31490000	26361000	0,84	0,74	-0,44	18,055	-0,2084776	NaN	-0,3185417	-0,118586	NaN	-0,5384295	NaN	NaN	NaN	-1,281632	-0,2750704	NaN	4,74E-02	1,32	-0,45678948	
DNA mismatch repair protein Msh2	MSH2	72536000	61936000	0,92	0,73	-0,44	13,982	-0,2935711	-0,4047828	-0,4475836	-0,3661131	-0,5778531	-0,4883498	-0,7347551	-0,8757249	-0,5376126	NaN	-0,5687729	-0,4787683	1,01E-06	6,00	-0,524898841	+
Eukaryotic translation initiation factor 3 subunit M	EIF3M	281440000	267790000	0,95	0,73	-0,45	18,611	-0,3245815	-0,2727329	-0,257666	-0,2677221	-0,5297808	-0,5403166	-0,6138291	-0,5170279	-0,4393242	-0,4766789	-1,332716	NaN	2,36E-04	3,63	-0,506579675	+
60S ribosomal protein L26;60S ribosomal protein L26-like 1	RPL26;RPL26L1	188460000	168270000	0,88	0,73	-0,45	25,427	-1,103767	-0,9841599	-0,6688681	-0,8652008	-1,082561	-0,9672784	-0,5290936	-0,3561623	-0,1533823	-0,1268483	-0,271409	-0,2922103	1,33E-04	3,88	-0,616745045	+
Eukaryotic translation initiation factor 5B	EIF5B	198720000	174280000	0,79	0,73	-0,45	16,895	-0,4911252	-0,4520173	-0,6665076	-0,688683	-0,6978272	-0,5700788	-0,1068087	-0,1454457	0,02034214	-0,2360616	-0,1886277	-0,05328412	7,19E-04	3,14	-0,356343716	+
40S ribosomal protein S26;Putative 40S ribosomal protein S26-like 1	RPS26;RPS26P11	350300000	373020000	1,04	0,73	-0,45	27,618	-0,8768635	-0,8114724	-0,7224199	-0,620135	-0,8625752	-0,7432308	0,1304037	-0,06745496	-0,0555763	0,09855437	0,309991	-0,2466864	1,42E-02	1,85	-0,37228879	+
Zinc finger CCCH-type antiviral protein 1	ZC3HAV1	29636000	27283000	0,70	0,73	-0,46	18,866	NaN	-0,6949517	NaN	-0,2553739	NaN	-0,6360328	-0,346463	NaN	-0,4031602	-0,3246899	-0,1569649	-0,4078803	4,14E-04	3,38	-0,403189564	+
DNA dC->dU-editing enzyme APOBEC-3C	APOBEC3C	32318000	26752000	1,06	0,73	-0,46	15,249	-0,715249	-0,7891124	NaN	-0,3127601	-0,7039244	-0,6093103	-0,2654833	-0,3914557	-0,384904	-0,2455571	NaN	NaN	1,22E-04	3,91	-0,490861807	+
40S ribosomal protein S11	RPS11	497830000	449920000	0,97	0,73	-0,46	24,813	-1,027204	-0,8467913	-0,7039715	-0,6324277	-0,9836178	-0,821483	-0,02952912	-0,01406246	-0,05523147	-0,07754336	-0,1318823	-0,1408096	2,81E-03	2,55	-0,455379493	+
Eukaryotic translation initiation factor 3 subunit A	EIF3A	630900000	544900000	0,84	0,72	-0,46	12,264	-0,4621772	-0,3941644	-0,4313063	-0,2447193	-0,651609	-0,5642861	-0,4934183	-0,5085881	-0,4697894	-0,4769399	-0,5422901	-0,3603974	7,88E-09	8,10	-0,466640451	+
Heterogeneous nuclear ribonucleoprotein A0	HNRNPA0	58237000	77130000	0,84	0,72	-0,46	24,118	-0,5761529	-0,7562335	-0,3171391	NaN	-0,5299683	-0,8146663	NaN	0,1437852	-0,1183824	-0,1324672	0,03745244	0,156526	3,19E-02	1,50	-0,290724596	
Trafficking protein particle complex subunit 4	TRAPPC4	9606400	9360700	1,09	0,72	-0,47	18,348	NaN	-0,4874394	NaN	-0,649548	NaN	-0,6064959	NaN	NaN	-0,448863	-0,432221	NaN	-0,4711887	3,27E-05	4,49	-0,515959327	+
60S ribosomal protein L34	RPL34	159590000	117610000	1,04	0,72	-0,47	25,052	-1,112631	-1,074851	-0,8536575	-0,7302009	-1,132167	-1,10479	-0,4688906	-0,2278948	-0,2390211	-0,1829498	-0,2089445	-0,3652767	1,69E-04	3,77	-0,64177292	+
Translationally-controlled tumor protein	TPT1	115190000	116840000	0,96	0,72	-0,47	10,886	-0,468611	-0,472229	-0,7267348	-0,558729	-0,7607974	-0,4725292	-0,4076315	-0,5015653	-0,5178332	-0,2852314	-0,5190731	-0,4031602	3,69E-08	7,43	-0,50784377	+
60S ribosomal protein L14	RPL14	962450000	694800000	1,07	0,72	-0,47	20,89	-1,229924	-1,261604	-0,9532995	-0,9336559	-1,249582	-1,243045	-0,4027406	-0,4204916	-0,2719315	-0,3718517	-0,3389984	-0,3766013	6,93E-05	4,16	-0,754477104	+
THO complex subunit 4	ALYREF	58862000	63340000	0,83	0,72	-0,47	21,74	-0,7243969	-0,7142072	-0,1828516	-0,2320571	-0,5822528	-0,654581	-0,1369338	-0,1866067	0,0790197	-0,02123325	-0,04868077	0,04725923	8,60E-03	2,07	-0,279793519	+
Hemoglobin subunit gamma-2	HBG2	1688100000	2062700000	1,20	0,72	-0,47	23,484	0,1894135	0,209391	0,1655583	0,1678717	0,1040682	0,1834547	-0,5408202	-0,5081366	-0,570443	-0,573746	-0,531594	-0,5554601	1,10E-01	0,96	-0,188370203	
60S ribosomal protein L15	RPL15	365140000	257480000	1,11	0,72	-0,47	25,866	-1,350081	-1,210797	-0,8173839	-0,918824	-1,164626	-1,225295	-0,4752944	-0,4357486	-0,3777068	-0,374636	-0,3593976	-0,3953023	3,76E-05	4,42	-0,758757676	+
Eukaryotic translation initiation factor 3 subunit C;Eukaryotic translation initiation factor 3 subunit C-like protein	EIF3C;EIF3CL	246440000	196590000	0,83	0,72	-0,48	12,967	-0,5355826	-0,3239132	-0,3818554	-0,368458	-0,7515867	-0,8027636	-0,6319804	-0,382852	-0,6051786	-0,6037089	-0,3009104	-0,4919162	3,91E-07	6,41	-0,51505883	+
Eukaryotic translation initiation factor 3 subunit H	EIF3H	138300000	117040000	0,89	0,72	-0,48	13,737	-0,3949988	-0,5222397	-0,3787006	-0,5101283	-0,7943321	-0,8056355	NaN	-0,3695014	-0,4384441	-0,3820433	NaN	NaN	2,11E-05	4,68	-0,510669324	+
Eukaryotic translation initiation factor 4 gamma 1	EIF4G1	630330000	499920000	0,73	0,72	-0,48	19,28	-0,4573755	-0,5072341	-0,388601	-0,4725093	-0,6236429	-0,6530147	-0,2940664	-0,4357682	-0,3557376	-0,2007137	-0,618916	-0,6601329	2,71E-07	6,57	-0,472309342	+
Nuclear pore complex protein Nup133	NUP133	78544000	60297000	0,82	0,71	-0,49	17,004	-0,7032196	-0,5859077	-0,1664217	-0,2019743	-1,009786	-0,6990212	0,04306451	-0,2886637	NaN	-0,3174627	-0,2935181	-0,07305935	2,26E-03	2,65	-0,390542694	+
60S acidic ribosomal protein P0;60S acidic ribosomal protein P0-like	RPLP0;RPLP0P6	1086400000	1050800000	1,11	0,71	-0,49	19,519	-0,9753683	-0,9961675	-0,6841556	-0,7662101	-0,881587	-0,9677863	-0,3648679	-0,282737	-0,2385104	-0,2798442	-0,130855	-0,1132704	1,97E-04	3,71	-0,556779973	+
Adenylosuccinate synthetase isozyme 2	ADSS	256030000	228390000	0,88	0,71	-0,50	9,6019	-0,5296141	-0,4995854	-0,548628	-0,6130787	-0,547004	-0,4891393	NaN	NaN	NaN	NaN	-0,3839247	-0,4662573	1,29E-07	6,89	-0,509653933	+
60S ribosomal protein L29	RPL29	84700000	89498000	1,28	0,71	-0,50	23,61	NaN	-1,261846	-0,9813944	-1,085405	-1,451386	NaN	-0,4691502	-0,3354625	-0,2722102	-0,1703128	-0,295854	-0,3359359	1,67E-03	2,78	-0,665895678	+
Structural maintenance of chromosomes protein 2	SMC2	86994000	62267000	0,79	0,71	-0,50	12,409	-0,4749133	-0,4627536	-0,6769413	-0,545445	-0,7637585	-0,4108693	NaN	NaN	-0,5663781	NaN	NaN	-0,2652232	3,04E-05	4,52	-0,520785291	+
60S ribosomal protein L35a	RPL35A	169500000	138740000	0,60	0,70	-0,51	24,194	-1,171661	-0,4536166	-0,7605531	-0,6065399	-1,138283	-1,123843	-0,3582505	-0,4788488	-0,4550201	-0,3844143	-0,4354755	NaN	4,48E-05	4,35	-0,669682289	+
Enhancer of mRNA-decapping protein 4	EDC4	19228000	16816000	0,87	0,70	-0,51	22,943	NaN	NaN	NaN	-0,5021373	-0,4838436	-0,8250056	NaN	NaN	NaN	NaN	-0,3819306	-0,5079725	1,95E-03	2,71	-0,540177917	+
Myb-binding protein 1A	MYBBP1A	144200000	116160000	0,75	0,70	-0,52	17,623	-0,6961199	-0,7330995	-0,6228651	-0,527741	-0,6860113	-0,5562024	-0,1887098	-0,2682607	NaN	NaN	-0,3115062	-0,05685135	1,72E-04	3,76	-0,464736722	+
Medium-chain specific acyl-CoA dehydrogenase, mitochondrial	ACADM	347220000	314860000	0,81	0,70	-0,52	18,511	-0,5860377	-0,5793398	-0,3580287	-0,6003149	-0,5571361	-0,5431939	-0,1052249	NaN	-0,04812872	-0,1255886	NaN	NaN	1,07E-03	2,97	-0,389221461	+
40S ribosomal protein S19	RPS19	450890000	438950000	0,77	0,69	-0,53	19,552	-0,8332589	-0,5597069	-0,7420721	-0,5990905	-0,7427962	-0,6581053	-0,1652564	-0,1159257	-0,1254155	-0,06629143	-0,08635417	-0,08034721	1,05E-03	2,98	-0,39788502	+
ATP-dependent RNA helicase DDX3X	DDX3X	505490000	370440000	0,99	0,69	-0,53	26,116	-1,298247	-1,11688	-0,7623874	-0,9275785	-1,319982	-1,210797	-0,482029	-0,4600125	-0,446384	-0,4471508	-0,4533598	-0,4312091	1,63E-05	4,79	-0,779668013	+
Heterogeneous nuclear ribonucleoprotein A3	HNRNPA3	321130000	300800000	0,77	0,69	-0,53	18,781	-0,4957966	-0,5923753	0,04488368	-0,1951699	-0,6089142	-0,7695019	-0,07187609	-0,02069169	-0,07178508	-0,02713097	NaN	NaN	1,66E-02	1,78	-0,280835811	+
DNA (cytosine-5)-methyltransferase 1	DNMT1	18683000	16779000	0,74	0,69	-0,53	18,761	NaN	NaN	-1,023358	-0,6199356	-0,9397035	-0,6655232	NaN	NaN	-0,3564209	-0,432221	NaN	NaN	1,61E-03	2,79	-0,672860309	+
Exosome complex component CSL4	EXOSC1	17334000	18951000	0,96	0,69	-0,53	8,4526	-0,5961651	-0,5916577	NaN	-0,166778	-0,5932239	-0,6646311	NaN	0,2446436	NaN	NaN	0,09585687	NaN	6,37E-02	1,20	-0,32456505	
Phosducin-like protein 3	PDCL3	8683600	9151200	0,94	0,69	-0,54	7,3585	-0,4250557	NaN	-0,5042025	NaN	-0,6368402	NaN	NaN	NaN	NaN	-0,4396372	NaN	-0,5300723	1,79E-04	3,75	-0,507161582	+
Ras GTPase-activating protein-binding protein 1	G3BP1	389830000	348080000	0,82	0,69	-0,54	29,585	-0,6562397	-0,6859416	-0,5399809	-0,4522935	-0,7640769	-0,82133	-0,1875921	0,01863419	-0,1863604	-0,05047256	-0,2224645	-0,06562694	1,07E-03	2,97	-0,384478755	+
Mitotic spindle assembly checkpoint protein MAD2A	MAD2L1	22015000	21314000	0,96	0,68	-0,55	5,9063	-0,571343	-0,5326788	NaN	-0,5460137	-0,5444347	-0,5836358	-0,382175	-0,5515007	NaN	-0,07590009	NaN	NaN	1,10E-04	3,96	-0,473460221	+
Inositol hexakisphosphate and diphosphoinositol-pentakisphosphate kinase 2	PPIP5K2	76109000	62914000	0,82	0,68	-0,55	20,127	-0,4973028	-0,6218658	-0,5024438	-0,4776027	-0,5598344	-0,588986	NaN	NaN	-0,7851043	-0,5717933	-0,845028	NaN	6,09E-07	6,22	-0,605551246	+
Isopentenyl-diphosphate Delta-isomerase 1	IDI1	85071000	82001000	0,91	0,68	-0,55	3,0115	-0,7126932	-0,6974763	-0,6916857	-0,5219705	-0,5892248	-0,5249359	-0,4291679	-0,5082803	-0,4685911	-0,4163269	-0,296474	-0,5035684	1,50E-08	7,82	-0,530032905	+
Multifunctional methyltransferase subunit TRM112-like protein	TRMT112	102280000	87071000	0,82	0,68	-0,55	5,0836	-0,5890945	-0,6489597	-0,8125617	-0,6940645	-0,6342403	-0,6182515	-0,524085	-0,6421891	-0,6082103	-0,6324053	-0,4174248	-0,6469252	1,16E-10	9,94	-0,622367658	+
40S ribosomal protein S3	RPS3	1412600000	1434700000	0,77	0,68	-0,56	18,986	-0,6929911	-0,7943572	-0,4980972	-0,6104112	-0,654808	-0,7802894	-0,109281	-0,1818202	-0,2063954	-0,1252896	-0,07152735	-0,09882991	6,07E-04	3,22	-0,402008129	+
60S ribosomal protein L5	RPL5	573180000	519760000	0,75	0,68	-0,56	21,813	-0,8353424	-0,7681007	-0,4915916	-0,5704858	-0,7575499	-0,7585991	-0,1191969	-0,1353007	-0,1608305	-0,1030539	-0,1501127	-0,226797	5,01E-04	3,30	-0,423080126	+
YrdC domain-containing protein, mitochondrial	YRDC	33332000	31135000	0,94	0,68	-0,56	14,799	-0,4819081	-0,4689904	-0,7668481	-0,7029378	-0,754042	-0,7062764	-0,4773215	-0,4457353	-0,3514425	-0,5091218	-0,3204861	-0,3998825	1,60E-07	6,79	-0,532082709	+
RNA-binding motif protein, X chromosome;RNA-binding motif protein, X chromosome, N-terminally processed;RNA binding motif protein, X-linked-like-1	RBMX;RBMXL1	149110000	116090000	1,02	0,68	-0,56	19,211	-1,081217	-0,2432329	-0,5957508	-0,1811494	-1,174361	-1,134287	-0,2648245	0,05644473	-0,4707488	-0,5317608	NaN	NaN	2,72E-03	2,57	-0,56208872	+
Nucleolin	NCL	3802400000	3113800000	0,73	0,68	-0,56	22,78	-0,757306	-0,7458414	-0,5048984	-0,4595362	-0,7549181	-0,67075	-0,08327878	-0,06435923	-0,3509089	-0,2720186	0,1150332	0,1307991	2,86E-03	2,54	-0,36816528	+
Plastin-2	LCP1	299410000	263620000	0,78	0,68	-0,56	22,721	-0,676849	-0,633703	-0,6627342	-0,4483511	-0,6441719	-0,542038	NaN	NaN	NaN	NaN	0,3062623	0,3988976	5,60E-02	1,25	-0,362835914	
Heat shock 70 kDa protein 4L	HSPA4L	121030000	108140000	0,82	0,68	-0,56	10,874	-0,5969505	-0,5452344	-0,6208006	-0,5840467	-0,6221988	-0,4134611	-0,7469547	-0,7319009	-0,7579647	-0,5509722	-0,7123386	-0,82309	7,91E-10	9,10	-0,642159425	+
60S ribosomal protein L27a	RPL27A	536020000	477590000	0,88	0,67	-0,58	21,079	-1,074213	-0,949366	-0,7739848	-0,5095531	-0,9588972	-0,9603001	-0,2842647	-0,2498223	-0,2181619	-0,009742186	-0,1343821	0,1426094	1,94E-03	2,71	-0,498339836	+
Ribonucleoside-diphosphate reductase large subunit	RRM1	416950000	344780000	0,86	0,67	-0,58	27,712	-0,4658388	-0,5258911	-0,6021314	-0,4957966	-0,6149335	-0,5678531	-0,5571361	-0,5566904	-0,4953695	-0,7718154	-0,7784322	-0,6929446	6,26E-10	9,20	-0,593736048	+
Elongation factor 2	EEF2	8490700000	7145900000	0,87	0,67	-0,58	11,294	-0,5849769	-0,531302	-0,6961433	-0,6445552	-0,6949984	-0,6674467	-0,6035336	-0,5731663	-0,5442875	-0,5768198	-0,559558	-0,5872943	7,04E-13	12,15	-0,605340178	+
Lamin-B1	LMNB1	95364000	113050000	1,12	0,67	-0,58	27,291	NaN	0,1467856	NaN	0,1915626	-0,5788871	-0,2253795	-0,7979397	-0,7491596	-0,5516065	-0,5466879	-0,6617525	-0,6577639	3,54E-03	2,45	-0,443082862	+
40S ribosomal protein S3a	RPS3A	557520000	511290000	0,78	0,67	-0,59	19,436	-0,812815	-0,885633	-0,6616612	-0,6457735	-0,7041595	-0,6580826	-0,2788111	-0,2822458	-0,2657782	-0,2195556	-0,2521228	-0,2374386	3,56E-05	4,45	-0,492006406	+
Nucleolar RNA helicase 2	DDX21	1691800000	1358600000	0,75	0,66	-0,59	24,313	-0,8847536	-0,7500086	-0,4257725	-0,4690903	-0,8518338	-0,7967863	0,04697992	0,1491292	0,1301402	0,01378388	-0,03739916	-0,01927275	2,01E-02	1,70	-0,324573645	+

Ras GTPase-activating protein-binding protein 2	G3BP2	121840000	106810000	0,71	0,66	-0,59	29,197	-0,7624853	-0,8372743	-0,6143149	-0,5746696	-0,9275785	-0,9512895	NaN	NaN	NaN	NaN	-0,03056025	-0,08421142	2,20E-03	2,66	-0,597797968	+
40S ribosomal protein S20	RPS20	766690000	638990000	0,70	0,66	-0,59	21,061	-0,8865929	-0,6746366	-0,624132	-0,5563721	-0,7570379	-0,7453334	-0,06239953	-0,007898733	-0,000591656	0,02955887	0,1255188	0,04781753	1,13E-02	1,95	-0,342674966	+
Superkiller viralicidic activity 2-like 2	SKIV2L2	161300000	119210000	0,67	0,66	-0,60	22,249	-0,8010283	-0,6933178	-0,6481004	-0,5549302	-0,9091465	-0,8758307	-0,529989	-0,4511294	-0,3636236	-0,4331752	-0,4879248	-0,290233	6,47E-07	6,19	-0,586535737	+
Fatty acid synthase;	FASN	9073700000	7198000000	0,82	0,66	-0,60	15,749	-0,6251998	-0,541723	-0,5757873	-0,5267845	-0,494292	-0,7013652	-0,435534	-1,707407	-0,9835892	-0,5497257	-0,7755892	-0,7484809	1,64E-05	4,79	-0,722123124	+
Myosin light polypeptide 6	MYL6	87255000	79967000	0,90	0,66	-0,60	13,352	-0,8941075	-0,7433274	-0,3964223	-0,209278	-0,7557218	-0,6584013	-0,4450281	-0,3164562	-0,5276162	-0,4221725	-0,3385787	-0,3206303	5,50E-06	5,26	-0,502311682	+
40S ribosomal protein S15a	RPS15A	583560000	523020000	0,87	0,66	-0,61	24,493	-0,9355034	-0,9035493	-0,6724046	-0,7152727	-0,8611593	-0,7099295	-0,1985598	-0,1324989	-0,1458767	-0,129876	-0,07172444	-0,02077945	1,21E-03	2,92	-0,458094511	+
60S ribosomal protein L35	RPL35	239670000	226330000	0,70	0,65	-0,61	29,909	-0,9601879	-0,7621672	-0,5500425	-0,4830772	-0,8218145	-0,7869701	0,02332628	-0,1393312	0,1538053	0,02389408	-0,1475057	-0,1261868	6,03E-03	2,22	-0,381354778	+
40S ribosomal protein S6	RPS6	453580000	506550000	0,70	0,65	-0,62	19,468	-0,8270005	-0,9960524	-0,6403438	-0,5458872	-0,8812948	-0,8666205	-0,1083476	-0,0565512	-0,03765087	-0,1365373	0,006046604	-0,1390452	2,68E-03	2,57	-0,43577373	+
Ubiquitin-conjugating enzyme E2 S	UBE2S	42414000	41908000	0,88	0,65	-0,62	8,5214	-0,1288818	-0,4341494	-0,4716487	-0,6440365	-0,5070291	-0,7126696	-0,3713104	NaN	-0,597387	NaN	NaN	NaN	1,37E-04	3,86	-0,483389053	+
Eukaryotic translation initiation factor 3 subunit D	EIF3D	121420000	112750000	1,00	0,65	-0,62	15,981	-0,3949229	-0,6120199	-0,3468116	-0,5838521	-0,6549216	-0,862208	-0,2281144	NaN	NaN	NaN	-0,6330093	-0,4610844	3,32E-05	4,48	-0,530771578	+
Importin subunit alpha-1	KPNA2	706490000	580150000	0,81	0,65	-0,62	15,832	-0,7170976	-0,5774225	-0,6992553	-0,5338689	-0,8157578	-0,8492843	-0,3101999	-0,4113873	-0,2628497	-0,1996364	-0,6075068	-0,329903	4,93E-06	5,31	-0,526180796	+
Nascent polypeptide-associated complex subunit alpha	NACA	520110000	465620000	0,78	0,65	-0,62	11,951	-0,6211555	-0,5308225	-0,6477838	-0,613189	-0,71345	-0,6635795	-0,3953023	-0,5571361	-0,6487787	-0,6775641	-0,1328784	-0,2525181	6,26E-07	6,20	-0,537846518	+
Eukaryotic translation initiation factor 4 gamma 2	EIF4G2	42215000	40797000	0,69	0,64	-0,63	24,218	-0,8158848	0,0415233	-0,54905	NaN	-1,255184	-0,4527871	NaN	NaN	-0,1735959	-0,355313	NaN	NaN	1,98E-02	1,70	-0,508613151	+
DNA topoisomerase 1	TOP1	91047000	65898000	0,65	0,64	-0,64	29,402	-0,8678048	-0,5407572	-0,8454427	-0,7448014	-0,8520421	-0,8781093	NaN	0,1882743	0,2697513	0,1849785	NaN	NaN	2,89E-02	1,54	-0,453994809	
Cytochrome c oxidase subunit 2	MT-CO2	182660000	163220000	0,99	0,64	-0,64	11,076	-0,8236261	-0,8018579	NaN	-0,793882	-0,6994194	-0,7947826	-0,5784562	-0,5608131	-0,5329292	-0,5524948	-0,5117112	-0,5466668	1,05E-08	7,98	-0,654239942	+
40S ribosomal protein S25	RPS25	858990000	809110000	0,77	0,64	-0,65	21,473	-0,7271884	-0,8036196	-0,9306553	-0,7621672	-0,7662346	-0,7620937	-0,1876908	-0,1420031	-0,1752077	-0,07313524	-0,1083786	-0,003683579	1,13E-03	2,95	-0,453504804	+
Eukaryotic translation initiation factor 3 subunit E	EIF3E	290720000	267910000	0,88	0,64	-0,65	22,525	-0,5007077	-0,5381991	-0,5240228	-0,3639949	-0,8543617	-0,8081847	NaN	NaN	NaN	NaN	-0,6279617	-0,7347072	1,67E-05	4,78	-0,619017474	+
Plasminogen activator inhibitor 1 RNA-binding protein	SERBP1	181840000	131160000	0,80	0,64	-0,65	26,034	-0,8666996	-0,7196376	-0,7678797	-0,7296265	-0,6579915	-0,8161134	-0,03578624	NaN	NaN	NaN	-0,5573059	-0,2146253	2,38E-04	3,62	-0,596185075	+
40S ribosomal protein S10	RPS10	395310000	344840000	0,81	0,63	-0,67	26,661	-0,7830424	-0,7873187	-0,719115	-0,6558305	-0,7027498	-0,7165049	-0,1579947	-0,2890514	-0,002830427	0,01720924	-0,1637042	-0,1441059	8,80E-04	3,06	-0,425419897	+
60S ribosomal protein L36	RPL36	255940000	197570000	0,61	0,61	-0,70	23,879	-1,134224	-1,157045	-0,8704137	-0,8318972	-1,120482	-1,002281	-0,5982385	-0,6765031	-0,4435754	-0,3193696	-0,3086445	-0,3787569	8,17E-06	5,09	-0,736785971	+
60S ribosomal protein L10a	RPL10A	809000000	617760000	0,85	0,61	-0,72	24,474	-1,155887	-1,125195	-0,9218547	-0,8878203	-1,126643	-1,13841	-0,4818275	-0,5032412	-0,3236965	-0,3836987	-0,2642526	-0,3021729	3,42E-05	4,47	-0,717891691	+
60S ribosomal protein L6	RPL6	625470000	515680000	0,64	0,60	-0,73	21,219	-1,037207	-0,9677017	-0,7754164	-0,8617884	-0,9727893	-1,07133	-0,3929515	-0,3404042	-0,3286342	-0,3144096	-0,1727501	-0,1778001	9,20E-05	4,04	-0,617765194	+
60S ribosomal protein L8	RPL8	263350000	234510000	0,63	0,60	-0,73	11,48	-1,00474	-1,007899	-0,8141334	-0,6750972	-0,9195061	-1,11391	-0,5583465	-0,5844577	-0,2880118	-0,3022262	-0,58072	-0,5332841	2,50E-06	5,60	-0,698527656	+
Eukaryotic translation initiation factor 3 subunit K	EIF3K	108850000	91222000	0,80	0,60	-0,73	9,8901	-0,7115824	-0,6589251	-0,6089362	-0,520252	-0,900395	-0,9733275	-0,9207892	NaN	-1,039073	-0,7673637	-0,8264375	-0,5446662	4,93E-08	7,31	-0,770158903	+
40S ribosomal protein S28	RPS28	54412000	64100000	0,71	0,60	-0,74	26,234	-0,86019	-0,8122575	-0,5596643	-0,8424646	-0,764567	-0,3551654	-0,2177592	-0,1444089	-0,1722785	-0,1143787	-0,110324	-0,09602082	8,64E-04	3,06	-0,420789915	+
Probable ATP-dependent RNA helicase DDX5	DDX5	1560800000	1277100000	0,88	0,59	-0,75	19,507	-1,292157	-1,210363	-0,8370165	-0,9785203	-1,404535	-1,090404	-0,3793948	-0,5867525	-0,963982	-0,7168131	-0,4352413	-0,4709486	3,39E-06	5,47	-0,863843923	+
Probable ATP-dependent RNA helicase DDX47	DDX47	11795000	12535000	0,73	0,59	-0,76	29,185	-0,9332977	-0,8257214	NaN	-0,4250944	-0,8128911	-0,7791499	NaN	NaN	NaN	NaN	-0,3310277	-0,5920056	2,25E-04	3,65	-0,671312549	+
40S ribosomal protein S8	RPS8	1845100000	1368200000	0,81	0,59	-0,77	21,865	-1,050413	-1,063228	-0,817562	-0,8674101	-1,044062	-0,9391779	-0,2436598	-0,2345331	-0,2797041	-0,1788939	-0,1981957	-0,2573728	2,45E-04	3,61	-0,597851064	+
60S ribosomal protein L23a	RPL23A	765200000	602970000	0,72	0,59	-0,77	19,84	-1,021746	-0,9832755	-0,7420721	-0,8199285	-0,9919435	-0,971742	-0,2950926	-0,3695759	-0,1790082	-0,1961117	-0,1568201	-0,2114479	2,17E-04	3,66	-0,578230313	+
40S ribosomal protein S13	RPS13	1128700000	983100000	0,68	0,58	-0,77	25,484	-1,044777	-1,041863	-0,7578427	-0,7470758	-0,9501463	-0,8809227	-0,1519871	-0,1756965	-0,1444089	-0,04595264	-0,153639	-0,1389022	1,13E-03	2,95	-0,519434423	+
60S ribosomal protein L3	RPL3	911760000	632680000	0,68	0,58	-0,79	18,976	-1,054512	-1,119009	-0,7468578	-0,7379037	-1,020487	-0,9710915	-0,3776131	-0,4646039	-0,1154255	-0,1125683	-0,1492486	-0,1010565	4,37E-04	3,36	-0,580864739	+
Polyadenylate-binding protein 1;Polyadenylate-binding protein 3	PABPC1;PABPC3	2056400000	1447700000	0,65	0,58	-0,79	18,338	-0,8737407	-0,8489724	-0,6650199	-0,6844106	-0,9443601	-0,9969447	-0,6331882	-0,5337644	-0,6223764	-0,6539451	-0,5186803	-0,5187837	1,43E-08	7,84	-0,707848892	+
40S ribosomal protein S4, X isoform	RPS4X	1393600000	1280300000	0,70	0,57	-0,80	24,553	-0,9690283	-0,976333	-0,9030365	-0,7787045	-0,9294733	-0,8278198	-0,09181732	-0,005289984	-0,03498781	0,03449741	-0,1248648	-0,1442972	3,20E-03	2,49	-0,479262929	+
Transcription factor BTF3	BTF3	121030000	97758000	0,80	0,57	-0,81	18,423	-0,6829275	-0,8451576	-1,043051	-0,9762194	-1,034338	-0,9136781	-0,7716923	-0,755113	-0,664128	-0,7228007	-0,8793296	-0,8199285	1,55E-10	9,81	-0,842363631	+
60S ribosomal protein L30	RPL30	582680000	468120000	0,68	0,57	-0,82	23,464	-1,069967	-1,002397	-0,8077554	-0,7423376	-1,043319	-1,002021	-0,3593605	-0,470189	-0,1824914	-0,2642872	-0,2686604	-0,3201258	6,54E-05	4,18	-0,627742564	+
40S ribosomal protein S7	RPS7	349430000	312060000	0,73	0,56	-0,82	24,59	-0,9337937	-0,8189865	-0,728168	-0,7778879	-0,9663197	-0,9358069	-0,1246131	-0,1643992	-0,1179596	-0,1212198	-0,2275569	-0,09309399	8,50E-04	3,07	-0,500817103	+
60S ribosomal protein L21	RPL21	752460000	593510000	0,69	0,56	-0,83	26,324	-1,159235	-1,16482	-0,9405063	-0,966348	-1,200117	-1,102589	-0,5944433	-0,3667455	-0,3613978	-0,3703403	-0,4025689	-0,3867324	1,93E-05	4,71	-0,75132026	+
1,2-dihydroxy-3-keto-5-methylthiopentene dioxygenase	ADI1	47821000	32685000	0,65	0,56	-0,84	16,387	-0,7550642	-0,6517222	-0,804728	-1,100824	-1,06362	-1,183998	-1,040497	NaN	-0,9053046	NaN	NaN	NaN	2,13E-06	5,67	-0,938219838	+
60S ribosomal protein L27	RPL27	676590000	497520000	0,58	0,56	-0,84	23,444	-1,253154	-1,142895	-0,838925	-0,7869701	-0,9589815	-0,9298854	-0,3096813	-0,2592192	-0,2188669	-0,1407301	-0,2671317	-0,1762507	3,65E-04	3,44	-0,606890902	+
60S ribosomal protein L7a	RPL7A	950900000	759170000	0,61	0,55	-0,85	14,657	-1,170979	-1,201742	-0,8159356	-0,8967106	-1,133147	-0,9665735	-0,5068241	-0,4616009	-0,3883176	-0,2503713	-0,4998506	-0,541618	1,02E-05	4,99	-0,736139181	+
Ribosome biogenesis protein BRX1 homolog	BRIX1	88794000	56882000	0,61	0,55	-0,86	18,843	-0,9723081	-1,198725	-0,5960998	-0,612483	NaN	-1,09542	-0,4636088	NaN	NaN	-0,3125093	NaN	NaN	1,08E-03	2,97	-0,750164862	+
Caprin-1	CAPRIN1	271060000	191860000	0,56	0,55	-0,86	16,008	-1,082041	-1,017475	-0,8599805	-0,9403679	-1,261431	-1,102651	-0,7170265	-0,6160387	-0,481203	-0,5346001	NaN	NaN	2,81E-06	5,55	-0,861281586	+
60S ribosomal protein L4	RPL4	2854200000	2013000000	0,63	0,55	-0,87	21,572	-1,058593	-1,029441	-0,8041233	-0,763538	-1,076067	-1,028705	-0,3838117	-0,2748609	0,2827364	0,7028348	-0,2205469	-0,2372685	1,30E-02	1,89	-0,490948714	+
Thymidylate synthase	TYMS	47024000	34353000	0,72	0,55	-0,87	13,18	-0,8208712	-0,941836	-0,803544	-1,072665	-0,7968865	-0,7877668	NaN	-0,9396483	-1,068756	-0,9455543	-1,077285	-1,07765	1,92E-10	9,72	-0,939314739	+
DnaJ homolog subfamily A member 1	DNAJA1	503920000	401830000	0,66	0,54	-0,88	24,871	-0,7647141	-0,8024868	-0,8798339	-0,8526151	-1,098042	-1,078046	NaN	-0,5904408	NaN	NaN	-0,8916161	-0,8691745	1,50E-07	6,82	-0,869663245	+
Nucleoplasmin-3	NPM3	116060000	89560000	0,70	0,54	-0,88	6,7783	-0,9656435	-1,121235	-0,6750972	-0,6027227	-0,9326091	-0,7684693	-0,4069618	-0,6337477	-0,4043054	NaN	-0,4447728	NaN	1,01E-05	5,00	-0,695556504	+
60S ribosomal protein L17	RPL17	336170000	232620000	0,70	0,54	-0,89	19,524	-1,189878	-1,065068	-0,9634194	-0,9787477	-1,183408	-1,109732	-0,5606216	-0,6254668	-0,3679366	-0,3867136	-0,47319	-0,6154859	3,07E-06	5,51	-0,7933057	+
60S ribosomal protein L10	RPL10	412380000	349240000	0,68	0,54	-0,89	21,537	-1,119416	-1,029029	-0,8251846	-0,8675153	-1,03771	-0,9947869	-0,1452223	-0,09495727	-0,1556143	-0,1731729	-0,1499847	-0,3218199	7,20E-04	3,14	-0,576201105	+
H/ACA ribonucleoprotein complex subunit 2	NHP2	21063000	19707000	0,69	0,54	-0,90	13,537	-1,006014	-0,9347309	-0,7054527	-0,816469	-1,007667	-1,053075	NaN	NaN	NaN	NaN	0,05297228	0,03040665	4,11E-03	2,39	-0,680003625	+
N-acetyltransferase 10	NAT10	176440000	108450000	0,55	0,54	-0,90	27,888	-1,199222	-1,095019	-0,8654636	-0,7209686	-0,9142219	-1,063228	-0,2127682	-0,3705082	-0,02127715	0,1288213	NaN	-0,1489606	2,15E-03	2,67	-0,589346928	+
60S ribosomal protein L7	RPL7	1197800000	965790000	0,62	0,53	-0,92	20,982	-1,170686	-1,059465	-0,9303527	-0,9064398	-1,139522	-1,012985	-0,4259275	-0,4492766	-0,2082609	-0,2824739	-0,2301268	-0,2690602	9,53E-05	4,02	-0,673714671	+
60S ribosomal protein L18a	RPL18A	727380000	588300000	0,72	0,53	-0,92	27,82	-1,167572	-1,157142	-0,8634411	-0,9324163	-1,16175	-1,136759	-0,4363928	-0,4541304	-0,2887518	-0,3945625	-0,5186803	-0,4078037	1,72E-05	4,77	-0,743283495	+
60S ribosomal protein L18	RPL18	1652800000	1244100000	0,64	0,53	-0,92	22,78	-1,19962	-1,159492	-0,8617098	-0,8921514	-1,151426	-1,187707	-0,3105219	-0,2842472	-0,2768172	-0,1656124	-0,266021	-0,3105219	2,35E-04	3,63	-0,672154025	+
Cold shock domain-containing protein E1	CSDE1	110200000	77589000	0,65	0,52	-0,95	15,828	-0,9474728	-0,9705825	NaN	-1,0635	-1,036674	-1,002021	-0,7804133	-0,7797195	-0,7916325	-0,9203795	-0,8523026	-0,7704121	1,14E-10	9,94	-0,901373576	+
60S ribosomal protein L9	RPL9	642330000	547410000	0,73	0,52	-0,95	27,81	-1,09798	-1,064555	-0,8230135	-0,8142602	-1,074426	-1,059134	-0,3392721	-0,3069123	-0,2897038	-0,2736745	-0,2539797	-0,2404181	1,18E-04	3,93	-0,636444158	+
60S acidic ribosomal protein P1	RPLP1	146410000	158830000	0,67	0,50	-1,00	20,84	-0,9941832	-1,054272	-0,8875	-0,8636774	-1,012112	-1,062505	0,1192898	-0,05584622	-0,02180435	0,153546	0,08474465	-0,04651869	1,15E-02	1,94	-0,470069873	+
60S ribosomal protein L13a	RPL13A	560000000	451700000	0,62	0,50	-1,00	24,349	-1,182917	-1,268643	-1,073393	-0,9667708	-1,198725	-1,170784	-0,4597346	-0,348225	-0,3481148	-0,2497365	-0,2851259	-0,2812285	1,08E-04	3,97	-0,736116532	+
60S acidic ribosomal protein P2	RPLP2	447670000	319040000	0,59	0,49	-1,02	19,585	-1,090189	-1,028852	-0,9695368	-0,8155038	-1,120953	-1,078655	-0,02135037	-0,1119757	-0,1494246	-0,1473139	-0,1193067	-0,1743121	1,64E-03	2,79	-0,568947713	+
Ribonucleoside-diphosphate reductase subunit M2	RRM2	53665000	28999000	0,61	0,48	-1,07	14,127	NaN	NaN	NaN	-1,356938	NaN	-1,334826	NaN	NaN	NaN	-0,746761	NaN	-0,9939822	4,78E-03	2,32	-1,108126715	+
Hydroxymethylglutaryl-CoA synthase, cytoplasmic	HMGCS1	498990000	266990000	0,54	0,41	-1,28	18,973	-1,228469	-1,535875	-1,339528	-1,374225	-1,388771	-1,354022	-1,508034	-1,539855	NaN	NaN	-1,356273	-1,393311	6,70E-12	11,17	-1,401836312	+

a	Summed up eXtracted Ion Current (XIC) of the isotopic cluster belonging to the light label partner of all peptide ions assigned to a Protein Group. It is proportional to the analyte concentration/abundance in the cell grown in the light medium
b	Summed up eXtracted Ion Current (XIC) of the isotopic cluster belonging to the heavy label partner of all peptide ions assigned to a Protein Group. It is proportional to the analyte concentration/abundance in the cell grown in the heavy medium
c	Median of all ratios between the intensities of each heavy and light label peptide partners. It is also called "SILAC ratio"
d	Assuming that most proteins do not change in a comparative analysis, the median of H/L ratio sub-populations was shifted to 1. Generally this valus is preferred to the previous one, for minimizing the effect of outliers and for correcting for unequal protein amounts.
e	We use the log2 for ratios because we want to represent up and down regulated proteins on the same scale
f	Coefficient of variability over all redundant quantifiable peptides. It is calculated as the standard deviation of the natural logarithm of ratios times 100.
g	These data are used for a statistical evaluation of SILAC ratios over all the twelve experiments. In "forward" experiments (FRW), cells treated with quercitin were grown in a medium containing Arg and Leu heavy labelled; consequentely original H/L normalised ratios, expressed as $\log_2$ were used. Conversely, in "reverse" experiments (REV) "heavy" cell coltures refers to the untreated status: for sake of clarity, in columns inverted values for H/L normalised ratios are shown. In the legend of single experiments, numbers refers to the biological replicates, whereas letters to the technical replicates.
h	*p* -value was obtained performing one sample T Test
i	Numerical column with the –log10 transformed *p* - value
l	Numerical column with the t-test difference corrisponding to average of expression value
m	+' shows statistically significant quatitative data with respect to the specified threshold < 0.05
