# Supplementary material for: Proteomic Analysis of Quercetin-Treated K562 Cells
Source: Int J Mol Sci. 2019 Dec 19;21(1):32. doi: 10.3390/ijms21010032 (PMC6981597; doi:10.3390/ijms21010032)
Supplement: Supplementary file 1 [file ijms-21-00032-s001.zip › jms-665671_Supplemetary materials/Table S3.pdf]

Table S3
Summary of enriched functional categories and their statistics

Change	GeneSet	size[a]	description	expected[b]	DEPs[c]	Gene Name	ratio[d]	pValue[e]	FDR[e]
up	GO:0006520	318	cellular amino acid metabolic process	0.55	7	GLCM;CTH;PHGDH;GARS;SEPHS1;GFPT1;AARS	12.53	9.14E-07	0.001
up	GO:0016053	401	organic acid biosynthetic process	0.70	6	CTH;PHGDH;CBR1;HK1;ANXA1;SEPHS1	8.52	5.52E-05	0.023
up	GO:0006091	469	generation of precursor metabolites and energy	0.82	6	TXNRD1;GSR;PHGDH;HK1;FECH;GFPT1	7.829	1.31E-04	0.037
down	GO:0022613	440	ribonucleoprotein complex biogenesis	2.47	33	RPL13A;RPL7;NAT10;NHP2;RPL10;NPM3;BRIX1;RPL7A;RPL27;RPS7;RPL3;RPL23A;RPS8;DDX47;RPS28;EIF3K;RPL6;RPL10A;RPS10;EIF3E;EIF3D;RPS6;RPL35;G3BP2;DDX21;RBMX;RPL5;TRMT112;G3BP1;DDX3X;RPS19;MYBBP1A;RPL35A	13.38	0	0
down	GO:0006605	412	protein targeting	2.31	37	RPLP2;RPL13A;RPLP1;RPL9;RPL18;RPL18A;RPL7;RPL10;RPL17;RPL4;RPL7A;RPL27;RPL21;RPS7;RPL30;RPS4X;RPL3;RPS13;RPL23A;RPS8;RPS28;RPL8;RPL6;RPL10A;RPL36;RPS10;RPS25;RPS6;RPL35;RPS15A;RPS20;RPS3A;RPL27A;RPL5;RPS3;RPS19;RPL35A	16.02	0	0
down	GO:0006401	341	RNA catabolic process	1.91	43	RPLP2;RPL13A;RPLP1;RPL9;CSDE1;RPL18;RPL18A;RPL7;RPL10;RPL17;RPL4;RPL7A;RPL27;RPL21;RPS7;RPL30;RPS4X;RPL3;RPS13;RPL23A;RPS8;DDX5;RPS28;RPL8;RPL6;RPL10A;RPL36;RPS10;SERBP1;EIF3E;RPS25;RPS6;RPL35;RPS15A;RPS20;RPS3A;RPL27A;RPL5;RPS3;RPS19;EDC4;RPL35A;RPL29	22.50	0	0
down	GO:0090150	313	establishment of protein localization to membrane	1.75	37	RPLP2;RPL13A;RPLP1;RPL9;RPL18;RPL18A;RPL7;RPL10;RPL17;RPL4;RPL7A;RPL27;RPL21;RPS7;RPL30;RPS4X;RPL3;RPS13;RPL23A;RPS8;RPS28;RPL8;RPL6;RPL10A;RPL36;RPS10;RPS25;RPS6;RPL35;RPS15A;RPS20;RPS3A;RPL27A;RPL5;RPS3;RPS19;RPL35A	21.09	0	0
down	GO:0016072	236	rRNA metabolic process	1.32	19	RPL7;NAT10;NHP2;NPM3;RPL7A;RPL27;RPS7;RPS8;DDX47;RPS28;RPL10A;RPS6;RPL35;DDX21;NCL;RPL5;TRMT112;RPS19;RPL35A	14.36	0	0
down	GO:0006413	192	translational initiation	1.08	43	RPLP2;RPL13A;RPLP1;RPL9;RPL18;RPL18A;RPL7;RPL10;RPL17;RPL4;RPL7A;RPL27;RPL21;RPS7;RPL30;RPS4X;RPL3;RPS13;RPL23A;RPS8;RPS28;EIF3K;RPL8;RPL6;RPL10A;RPL36;RPS10;EIF3E;RPS25;EIF4G2;EIF3D;RPS6;RPL35;RPS15A;RPS20;RPS3A;RPL27A;RPL5;RPS3;DDX3X;RPS19;RPL35A;RPL29	39.95	0	0
down	GO:0070972	137	protein localization to endoplasmic reticulum	0.77	37	RPLP2;RPL13A;RPLP1;RPL9;RPL18;RPL18A;RPL7;RPL10;RPL17;RPL4;RPL7A;RPL27;RPL21;RPS7;RPL30;RPS4X;RPL3;RPS13;RPL23A;RPS8;RPS28;RPL8;RPL6;RPL10A;RPL36;RPS10;RPS25;RPS6;RPL35;RPS15A;RPS20;RPS3A;RPL27A;RPL5;RPS3;RPS19;RPL35A	48.18	0	0
down	GO:0002181	92	cytoplasmic translation	0.52	17	RPL13A;RPLP1;RPL9;RPL18;RPL18A;RPL17;RPL30;RPS28;EIF3K;RPL8;RPL6;RPL10A;RPL36;EIF3E;EIF3D;EEF2;RPL35A	32.96	0	0

down	GO:0071826	245	ribonucleoprotein complex subunit organization	1.37	17	RPL13A;RPL10;BRIX1;RPL3;RPL23A;RPS28;EIF3K;RPL6;RPS10;EIF3E;EIF3D;G3BP2;RBMX;RPL5;G3BP1;DDX3X;RPS19	12.38	2.31E-14	2.18E-12
down	GO:0034470	367	ncRNA processing	2.06	18	RPL7;NAT10;NHP2;NPM3;RPL7A;RPL27;RPS7;RPS8;DDX47;RPS28;RPL10A;RPS6;RPL35;DDX21;RPL5;TRMT112;RPS19;RPL35A	8.75	1.42E-12	1.20E-10
down	GO:0010608	486	posttranscriptional regulation of gene expression	2.72	17	RPL13A;RPL10;TYMS;CAPRIN1;RPS4X;PABPC1;DDX5;EIF3K;SERBP1;EIF3E;EIF4G2;EIF3D;EEF2;NCL;RPL5;RPS3;DDX3X	6.24	1.24E-09	9.61E-08
down	GO:0034248	385	regulation of cellular amide metabolic process	2.16	15	RPL13A;RPL10;TYMS;CAPRIN1;RPS4X;PABPC1;EIF3K;EIF3E;EIF4G2;EIF3D;EEF2;NCL;RPL5;RPS3;DDX3X	6.95	3.11E-09	2.21E-07

[a] numbers of genes in the category

[b] expected number of genes for a statistically significant enrichment

[c] number of protein hits demonstrated by this study differentially expressed in quercetin-treated k562 cells

[d] enrichment ratio according to the WEBGestalt algorithm

[e] The p value of the new version WebGestalt is calculated from R. Zero means it is smaller than 2.220446E-16, the smallest positive floating-point number in R on the machine
