# Supplementary material for: Proteomic Analysis of Quercetin-Treated K562 Cells
Source: Int J Mol Sci. 2019 Dec 19;21(1):32. doi: 10.3390/ijms21010032 (PMC6981597; doi:10.3390/ijms21010032)
Supplement: Supplementary file 1 [file ijms-21-00032-s001.zip › jms-665671_Supplemetary materials/Table S4.pdf]

Table S4
Summary of enriched metabolic pathways and their statistics

Change	GeneSet	size[a]	description	expect[b]	DEPs[c]	Gene Symbol	ratio[d]	pValue[e]	FDR[e]
up	R-HSA-6791465	2	Pentose phosphate pathway disease	0.00	1	TALDO1	251.26	3.98E-03	7,19E-01
up	R-HSA-189451	11	Heme biosynthesis	0.02	2	FECH<br>HMBS	91.37	2.05E-04	2,04E-01
up	R-HSA-379716	24	Cytosolic tRNA Aminoacylation	0.05	2	GARS<br>AARS	41.88	1.01E-03	4.04E-01
up	R-HSA-71291	370	Metabolism of amino acids and derivatives	0.74	5	SLC3A2<br>TXNRD1<br>GSR<br>CTH<br>PHGDH	6.79	6,59E-04	3,28E-01
down	R-HSA-77348	5	Beta oxidation of octanoyl-CoA to hexanoyl-CoA	0.03	1	ACADM	30.15	3.27E-02	1,00E+00
down	R-HSA-72766	291	Translation	1.93	42	RPLP2<br>RPL13A<br>RPLP1<br>RPL9<br>RPL18<br>RPL18A<br>RPL7<br>RPL10<br>RPL17<br>RPL4<br>RPL7A<br>RPL27<br>RPL21<br>RPS7<br>RPL30<br>RPS4X<br>RPL3<br>RPS13<br>RPL23A<br>RPS8<br>RPS28<br>EIF3K<br>RPL8<br>RPL6<br>RPL10A<br>RPL36<br>RPS10	21.76	0,00E+00	0,00E+00

						EIF3E<br>RPS25<br>EIF3D<br>RPS6<br>RPL35<br>RPS15A<br>RPS20<br>RPS3A<br>EEF2<br>RPL27A<br>RPL5<br>RPS3<br>TRMT112<br>RPS19<br>RPL35A			
down	R-HSA-2426168	42	Activation of gene expression by SREBF	0.28	3	HMGCS1<br>FASN<br>IDI1	10.77	2.67E-03	1.25E-01
down	R-HSA-8953854	673	Metabolism of RNA	4.46	46	RPLP2<br>RPL13A<br>RPLP1<br>RPL9<br>RPL18<br>RPL18A<br>RPL7<br>NAT10<br>NHP2<br>RPL10<br>RPL17<br>RPL4<br>RPL7A<br>RPL27<br>RPL21<br>RPS7<br>RPL30<br>RPS4X<br>RPL3<br>RPS13<br>RPL23A<br>RPS8<br>DDX47<br>DDX5<br>RPS28	10.10	0,00E+00	0,00E+00

						RPL8<br>RPL6<br>RPL10A<br>RPL36<br>RPS10<br>RPS25<br>RPS6<br>RPL35<br>RPS15A<br>RPS20<br>DDX21<br>RPS3A<br>RPL27A<br>NCL<br>RPL5<br>RPS3<br>TRMT112<br>HNRNPA3<br>RPS19<br>EDC4<br>RPL35A			
down	R-HSA-1643685	1054	Disease	6.99	40	RPLP2<br>RPL13A<br>RPLP1<br>RPL9<br>RPL18<br>RPL18A<br>RPL7<br>RPL10<br>RPL17<br>RPL4<br>RPL7A<br>RPL27<br>RPL21<br>RPS7<br>RPL30<br>RPS4X<br>RPL3<br>RPS13<br>RPL23A<br>RPS8<br>RPS28<br>RPL8	5.72	0,00E+00	0,00E+00

						RPL6<br>RPL10A<br>RPL36<br>RPS10<br>RPS25<br>KPNA2<br>RPS6<br>RPL35<br>RPS15A<br>RPS20<br>RPS3A<br>LMNB1<br>EEF2<br>RPL27A<br>RPL5<br>RPS3<br>RPS19<br>RPL35A			
down	R-HSA-499943	30	Metabolism of nucleotides di- and triphosphates	0.20	3	TYMS<br>RRM1<br>RRM2	15.08	9.98E-04	4.79E-02
down	R-HSA-1266738	1074	Developmental Biology	7.14	38	RPLP2<br>RPL13A<br>RPLP1<br>RPL9<br>RPL18<br>RPL18A<br>RPL7<br>RPL10<br>RPL17<br>RPL4<br>RPL7A<br>RPL27<br>RPL21<br>RPS7<br>RPL30<br>RPS4X<br>RPL3<br>RPS13<br>RPL23A<br>RPS8<br>RPS28<br>RPL8	5.33	0	0

						RPL6<br>RPL10A<br>RPL36<br>RPS10<br>RPS25<br>RPS6<br>RPL35<br>RPS15A<br>MYL6<br>RPS20<br>RPS3A<br>RPL27A<br>RPL5<br>RPS3<br>RPS19<br>RPL35A			
down	R-HSA-71291	370	Metabolism of amino acids and derivatives	2.45	38	RPLP2<br>RPL13A<br>RPLP1<br>RPL9<br>RPL18<br>RPL18A<br>RPL7<br>RPL10<br>RPL17<br>RPL4<br>RPL7A<br>RPL27<br>ADI1<br>RPL21<br>RPS7<br>RPL30<br>RPS4X<br>RPL3<br>RPS13<br>RPL23A<br>RPS8<br>RPS28<br>RPL8<br>RPL6<br>RPL10A<br>RPL36<br>RPS10	15.58	0	0

						RPS25<br>RPS6<br>RPL35<br>RPS15A<br>RPS20<br>RPS3A<br>RPL27A<br>RPL5<br>RPS3<br>RPS19<br>RPL35A			

[a] numbers of genes in the category

[b] expected number of genes for a statistically significant enrichment

[c] number of protein hits demonstrated by this study differentially expressed in quercetin-treated k562 cells

[d] enrichment ratio according to the WEBGestalt algorithm

[e] The p value of the new version WebGestalt is calculated from R. Zero means it is smaller than 2.220446E-16, the smallest positive floating-point number in R on the machine
